# Supplementary material for: A diversity-oriented rhodamine library for wide-spectrum bactericidal agents with low inducible resistance against resistant pathogens
Source: Nat Commun. 2019 Jan 16;10:258. doi: 10.1038/s41467-018-08241-3 (PMC6335415; doi:10.1038/s41467-018-08241-3)
Supplement: Supplementary file 1 — Supplementary Information [file 41467_2018_8241_MOESM1_ESM.pdf]

Supplementary Information

A Diversity-Oriented Rhodamine Library for Wide-Spectrum Bactericidal Agents with Low Inducible Resistance against Resistant Pathogens

Luo *et al.*

Supplementary Figures

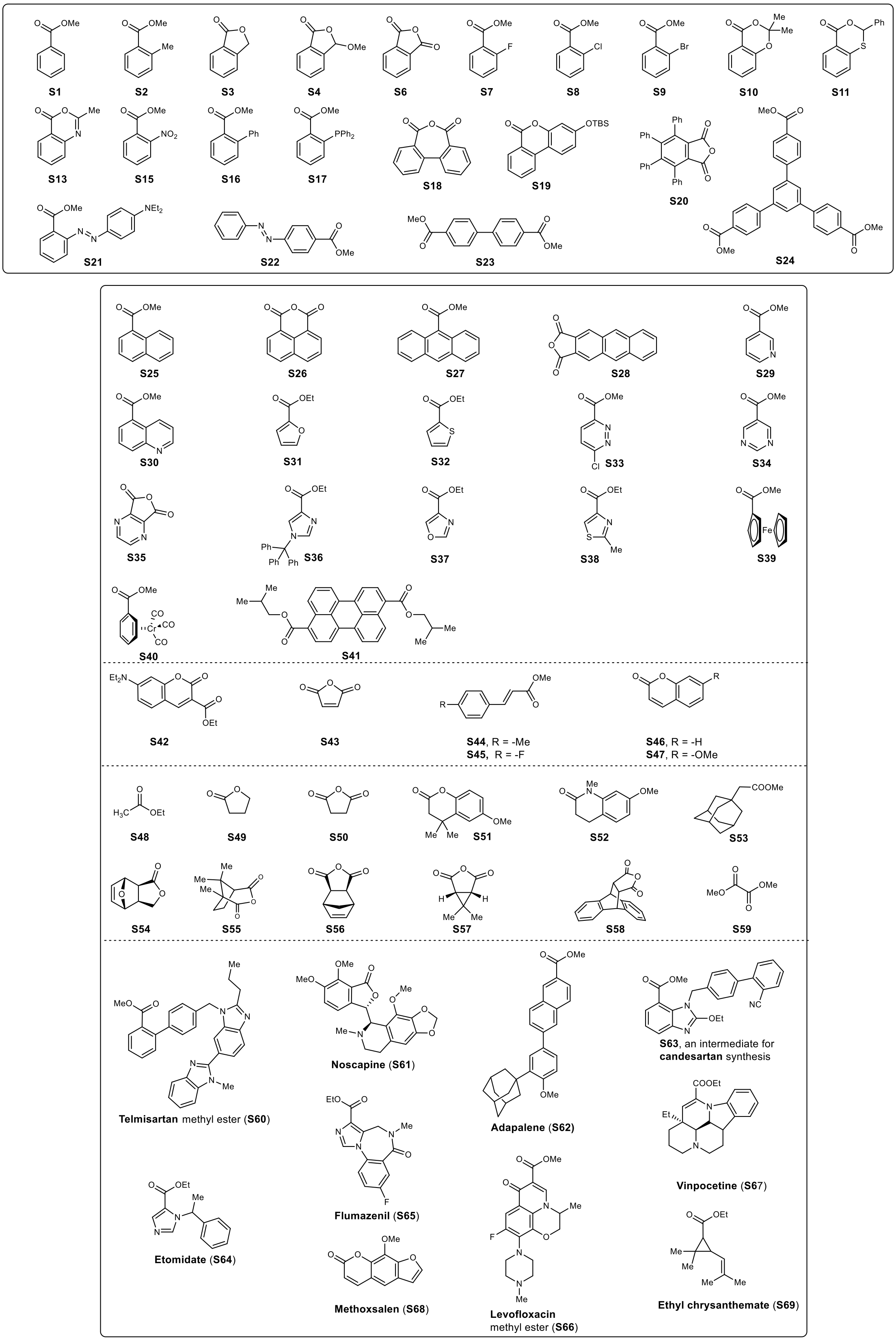

Supplementary Figure 1. Chemical structures of substrates **S1-S69**.

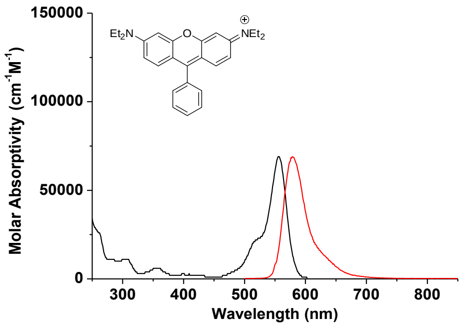

Supplementary Figure 2. The absorption (black) and fluorescence emission spectra (red) of **RD1** in pH=7.4 PBS with 1% DMSO as a co-solvent.

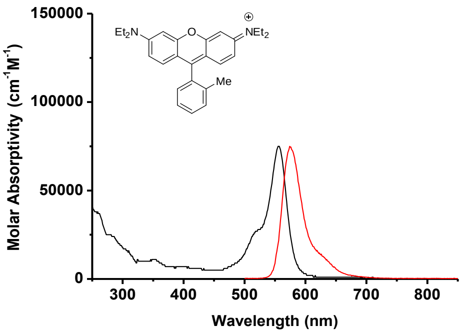

Supplementary Figure 3. The absorption (black) and fluorescence emission spectra (red) of **RD2** in pH=7.4 PBS with 1% DMSO as a co-solvent.

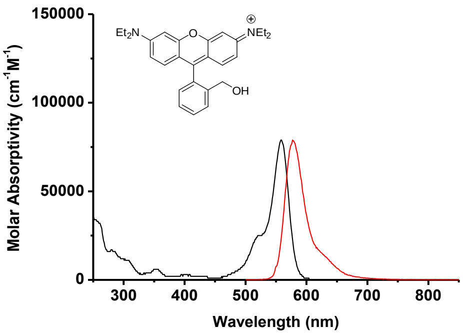

Supplementary Figure 4. The absorption (black) and fluorescence emission spectra (red) of **RD3** in pH=7.4 PBS with 1% DMSO as a co-solvent.

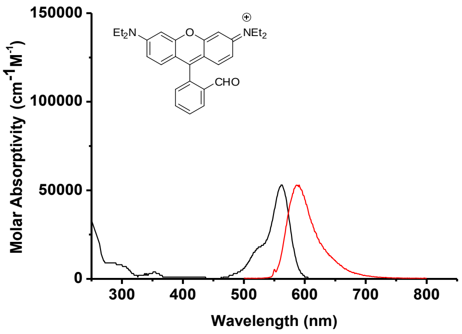

Supplementary Figure 5. The absorption (black) and fluorescence emission spectra (red) of **RD5** in pH=7.4 PBS with 1% DMSO as a co-solvent.

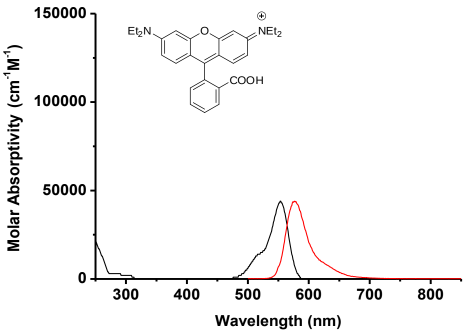

Supplementary Figure 6. The absorption (black) and fluorescence emission spectra (red) of **RD6** in pH=7.4 PBS with 1% DMSO as a co-solvent.

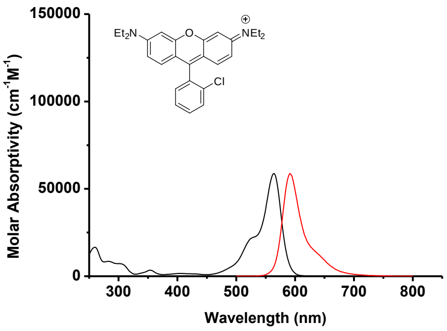

Supplementary Figure 7. The absorption (black) and fluorescence emission spectra (red) of **RD7** in pH=7.4 PBS with 1% DMSO as a co-solvent.

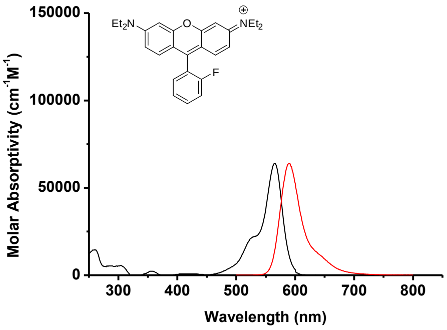

Supplementary Figure 8. The absorption (black) and fluorescence emission spectra (red) of **RD8** in pH=7.4 PBS with 1% DMSO as a co-solvent.

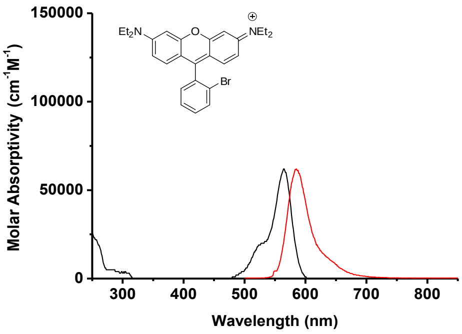

Supplementary Figure 9. The absorption (black) and fluorescence emission spectra (red) of **RD9** in pH=7.4 PBS with 1% DMSO as a co-solvent.

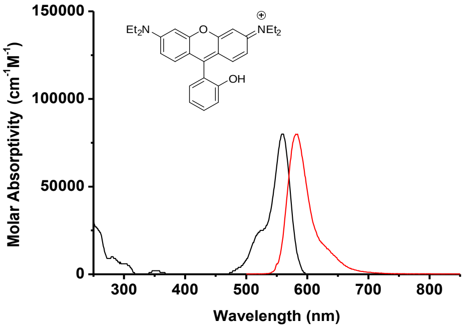

Supplementary Figure 10. The absorption (black) and fluorescence emission spectra (red) of **RD10** in pH=7.4 PBS with 1% DMSO as a co-solvent.

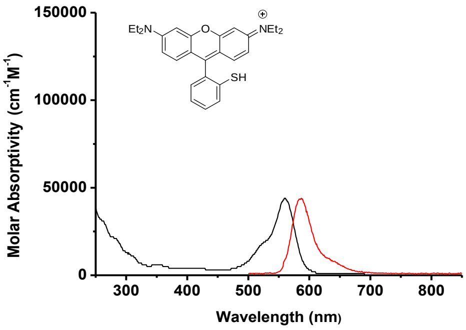

Supplementary Figure 11. The absorption (black) and fluorescence emission spectra (red) of **RD11** in pH=7.4 PBS with 1% DMSO as a co-solvent.

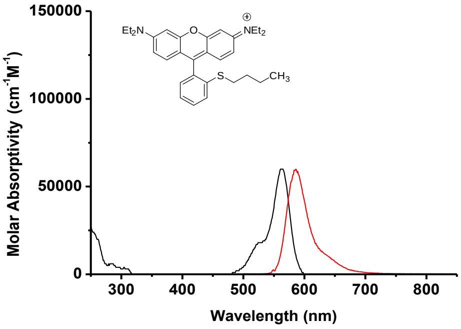

Supplementary Figure 12. The absorption (black) and fluorescence emission spectra (red) of **RD12** in pH=7.4 PBS with 1% DMSO as a co-solvent.

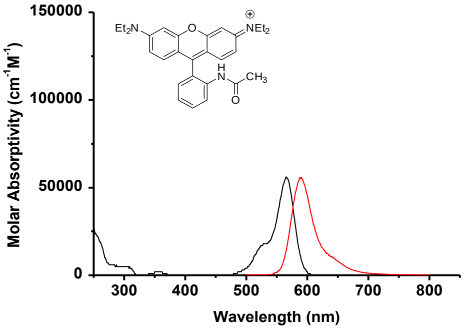

Supplementary Figure 13. The absorption (black) and fluorescence emission spectra (red) of **RD13** in pH=7.4 PBS with 1% DMSO as a co-solvent.

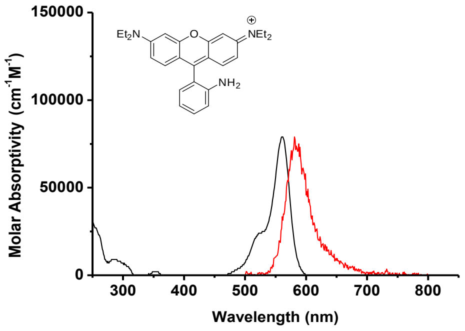

Supplementary Figure 14. The absorption (black) and fluorescence emission spectra (red) of **RD14** in pH=7.4 PBS with 1% DMSO as a co-solvent.

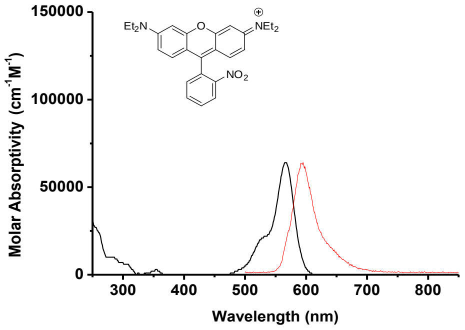

Supplementary Figure 15. The absorption (black) and fluorescence emission spectra (red) of **RD15** in pH=7.4 PBS with 1% DMSO as a co-solvent.

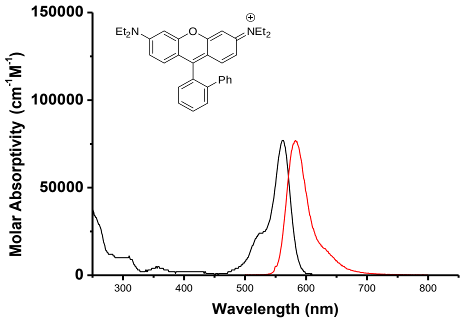

Supplementary Figure 16. The absorption (black) and fluorescence emission spectra (red) of **RD16** in pH=7.4 PBS with 1% DMSO as a co-solvent.

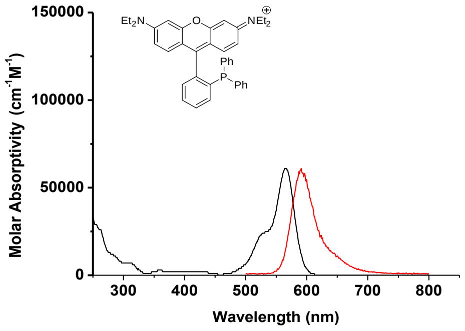

Supplementary Figure 17. The absorption (black) and fluorescence emission spectra (red) of **RD17** in pH=7.4 PBS with 1% DMSO as a co-solvent.

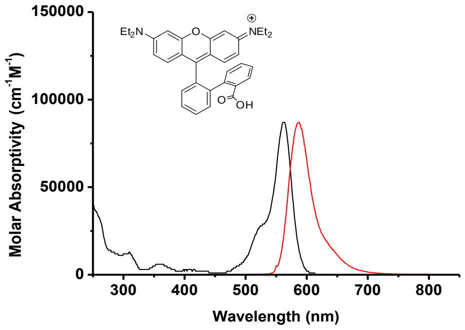

Supplementary Figure 18. The absorption (black) and fluorescence emission spectra (red) of **RD18** in pH=7.4 PBS with 1% DMSO as a co-solvent.

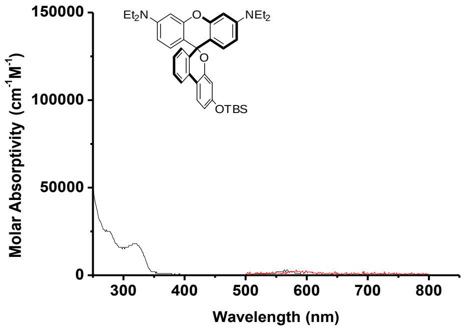

Supplementary Figure 19. The absorption (black) and fluorescence emission spectra (red) of **RD19** in pH=7.4 PBS with 1% DMSO as a co-solvent.

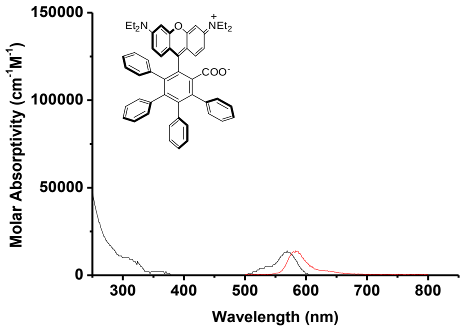

Supplementary Figure 20. The absorption (black) and fluorescence emission spectra (red) of **RD20** in pH=7.4 PBS with 1% DMSO as a co-solvent.

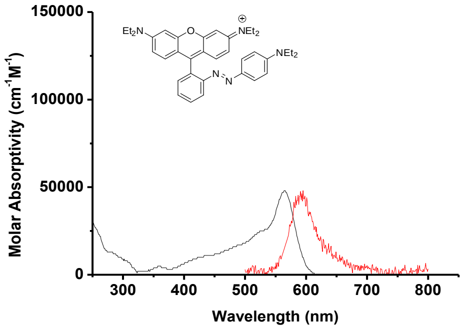

Supplementary Figure 21. The absorption (black) and fluorescence emission spectra (red) of **RD21** in pH=7.4 PBS with 1% DMSO as a co-solvent.

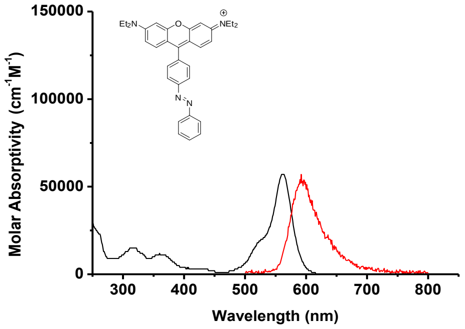

Supplementary Figure 22. The absorption (black) and fluorescence emission spectra (red) of **RD22** in pH=7.4 PBS with 1% DMSO as a co-solvent.

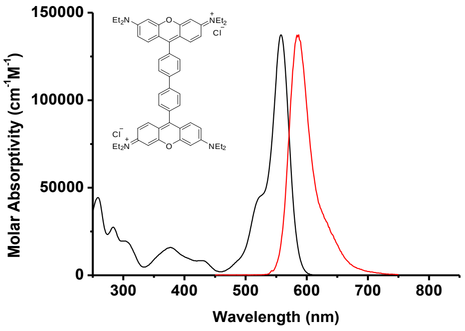

Supplementary Figure 23. The absorption (black) and fluorescence emission spectra (red) of **RD23** in ethanol.

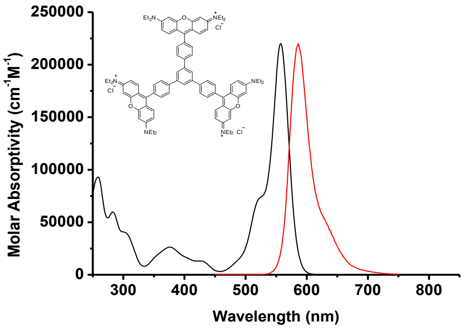

Supplementary Figure 24. The absorption (black) and fluorescence emission spectra (red) of **RD24** in ethanol.

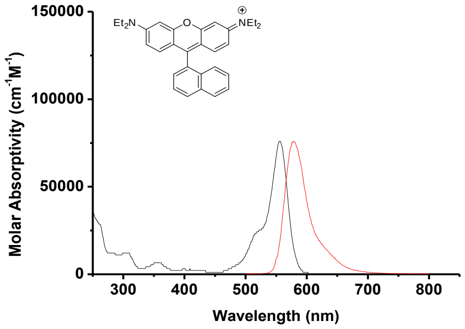

Supplementary Figure 25. The absorption (black) and fluorescence emission spectra (red) of **RD25** in pH=7.4 PBS with 1% DMSO as a co-solvent.

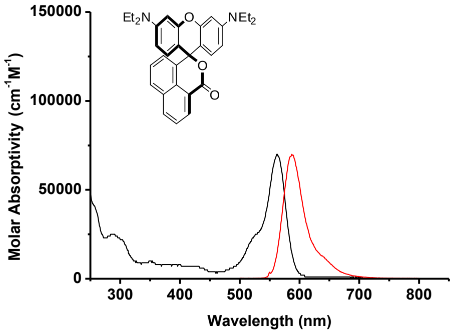

Supplementary Figure 26. The absorption (black) and fluorescence emission spectra (red) of **RD26** in pH=7.4 PBS with 1% DMSO as a co-solvent.

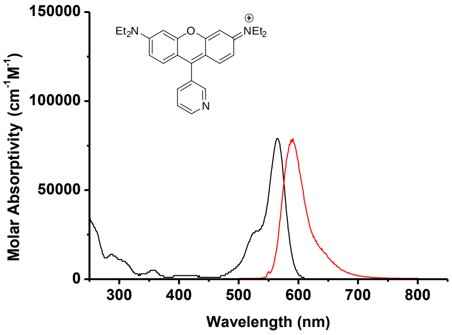

Supplementary Figure 29. The absorption (black) and fluorescence emission spectra (red) of **RD29** in pH=7.4 PBS with 1% DMSO as a co-solvent.

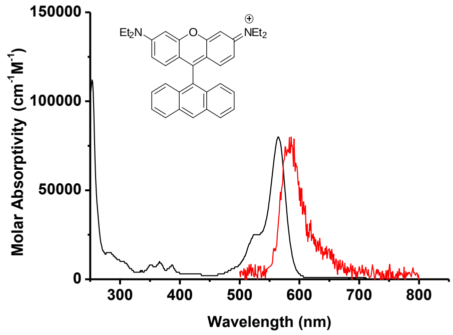

Supplementary Figure 27. The absorption (black) and fluorescence emission spectra (red) of **RD27** in pH=7.4 PBS with 1% DMSO as a co-solvent.

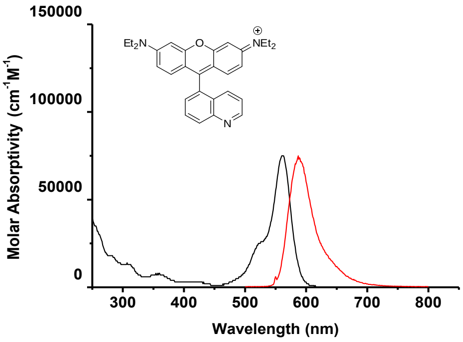

Supplementary Figure 30. The absorption (black) and fluorescence emission spectra (red) of **RD30** in pH=7.4 PBS with 1% DMSO as a co-solvent.

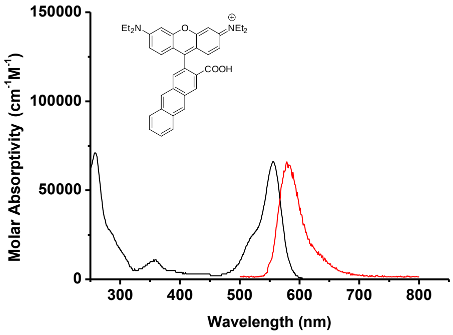

Supplementary Figure 28. The absorption (black) and fluorescence emission spectra (red) of **RD28** in pH=7.4 PBS with 1% DMSO as a co-solvent.

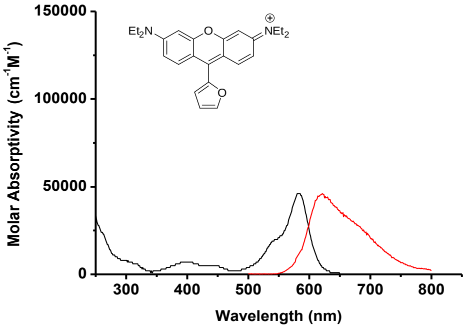

Supplementary Figure 31. The absorption (black) and fluorescence emission spectra (red) of **RD31** in pH=7.4 PBS with 1% DMSO as a co-solvent.

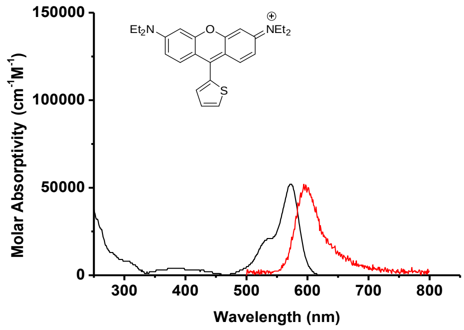

Supplementary Figure 32. The absorption (black) and fluorescence emission spectra (red) of **RD32** in pH=7.4 PBS with 1% DMSO as a co-solvent.

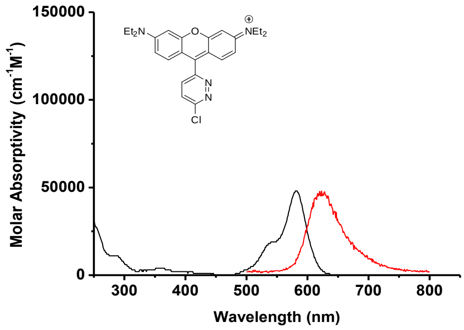

Supplementary Figure 33. The absorption (black) and fluorescence emission spectra (red) of **RD33** in pH=7.4 PBS with 1% DMSO as a co-solvent.

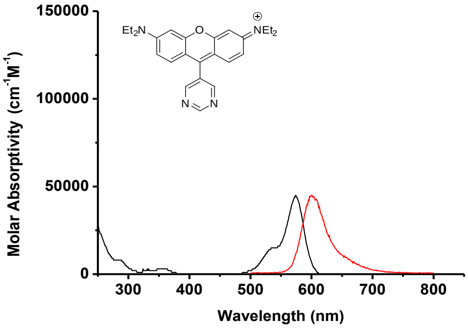

Supplementary Figure 34. The absorption (black) and fluorescence emission spectra (red) of **RD34** in pH=7.4 PBS with 1% DMSO as a co-solvent.

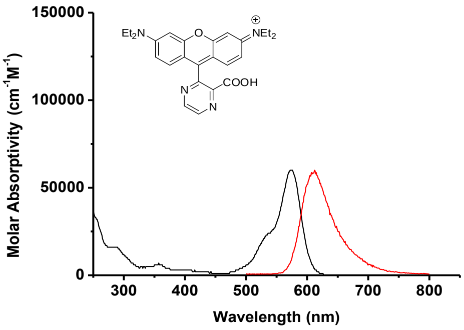

Supplementary Figure 35. The absorption (black) and fluorescence emission spectra (red) of **RD35** in pH=7.4 PBS with 1% DMSO as a co-solvent.

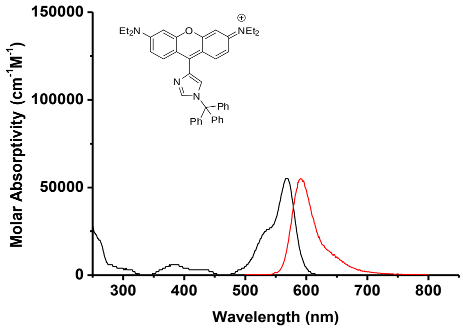

Supplementary Figure 36. The absorption (black) and fluorescence emission spectra (red) of **RD36** in pH=7.4 PBS with 1% DMSO as a co-solvent.

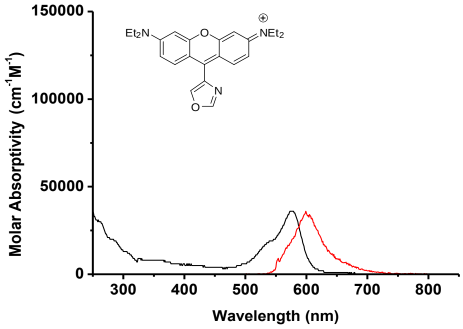

Supplementary Figure 37. The absorption (black) and fluorescence emission spectra (red) of **RD37** in pH=7.4 PBS with 1% DMSO as a co-solvent.

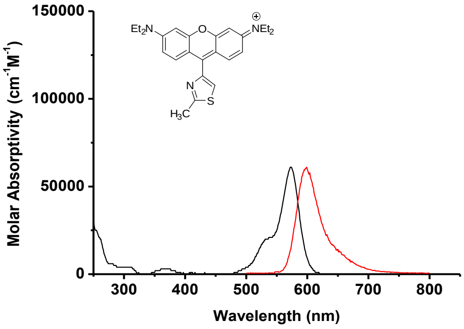

Supplementary Figure 38. The absorption (black) and fluorescence emission spectra (red) of **RD38** in pH=7.4 PBS with 1% DMSO as a co-solvent.

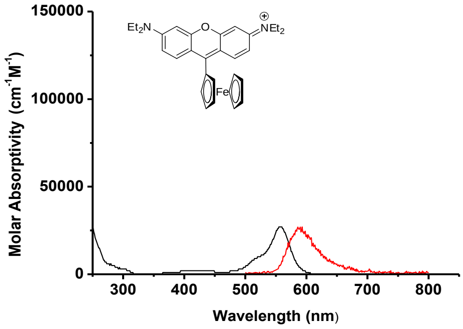

Supplementary Figure 39. The absorption (black) and fluorescence emission spectra (red) of **RD39** in pH=7.4 PBS with 1% DMSO as a co-solvent.

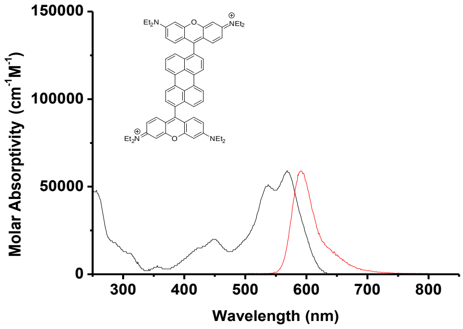

Supplementary Figure 40. The absorption (black) and fluorescence emission spectra (red) of **RD41** in pH=7.4 PBS with 1% DMSO as a co-solvent.

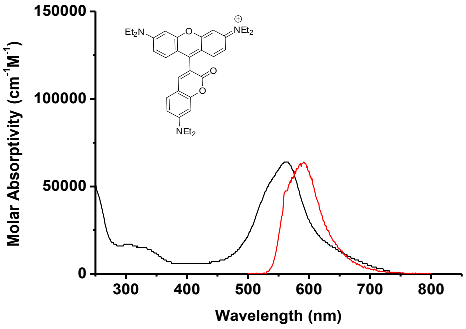

Supplementary Figure 41. The absorption (black) and fluorescence emission spectra (red) of **RD42** in pH=7.4 PBS with 1% DMSO as a co-solvent.

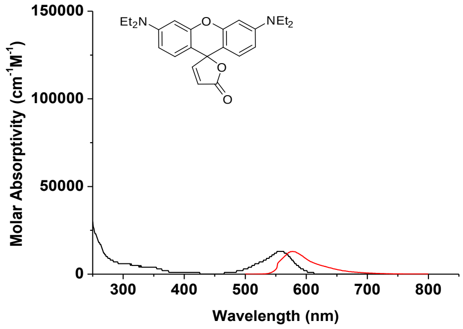

Supplementary Figure 42. The absorption (black) and fluorescence emission spectra (red) of **RD43** in pH=7.4 PBS with 1% DMSO as a co-solvent.

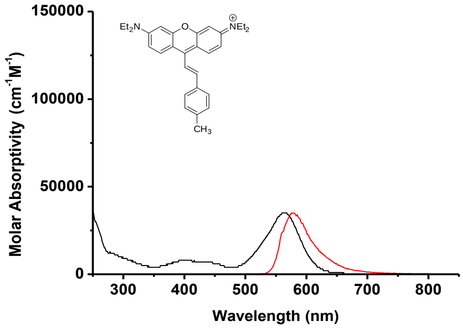

Supplementary Figure 43. The absorption (black) and fluorescence emission spectra (red) of **RD44** in pH=7.4 PBS with 1% DMSO as a co-solvent.

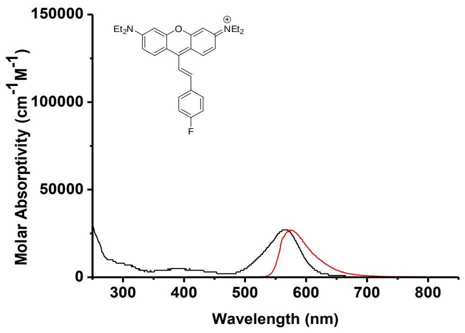

Supplementary Figure 44. The absorption (black) and fluorescence emission spectra (red) of **RD45** in pH=7.4 PBS with 1% DMSO as a co-solvent.

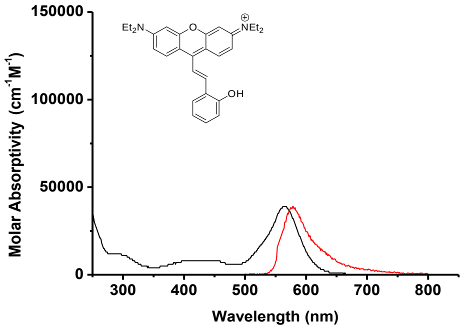

Supplementary Figure 45. The absorption (black) and fluorescence emission spectra (red) of **RD46** in pH=7.4 PBS with 1% DMSO as a co-solvent.

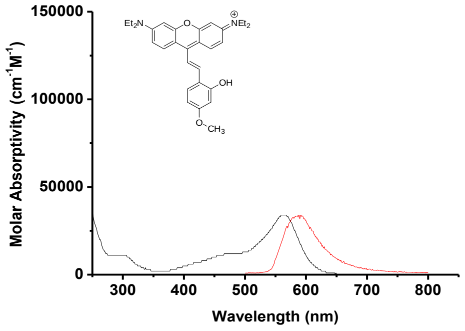

Supplementary Figure 46. The absorption (black) and fluorescence emission spectra (red) of **RD47** in pH=7.4 PBS with 1% DMSO as a co-solvent.

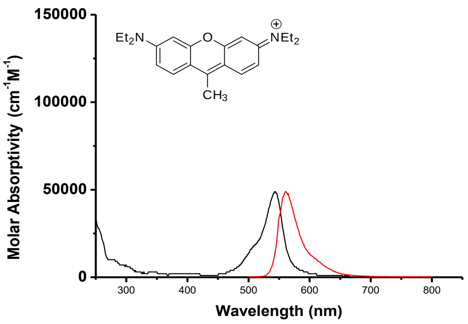

Supplementary Figure 47. The absorption (black) and fluorescence emission spectra (red) of **RD48** in pH=7.4 PBS with 1% DMSO as a co-solvent.

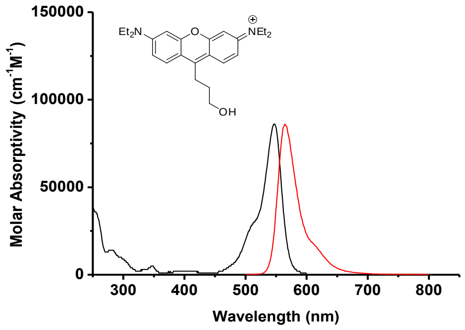

Supplementary Figure 48. The absorption (black) and fluorescence emission spectra (red) of **RD49** in pH=7.4 PBS with 1% DMSO as a co-solvent.

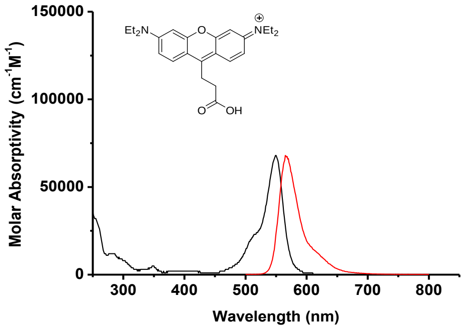

Supplementary Figure 49. The absorption (black) and fluorescence emission spectra (red) of **RD50** in pH=7.4 PBS with 1% DMSO as a co-solvent.

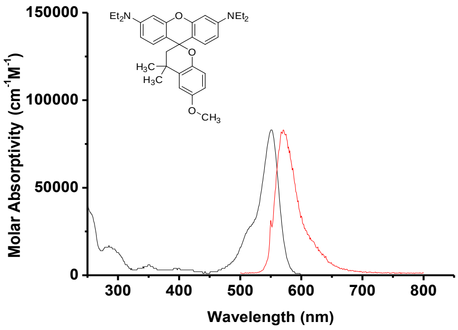

Supplementary Figure 50. The absorption (black) and fluorescence emission spectra (red) of **RD51** in pH=7.4 PBS with 1% DMSO as a co-solvent.

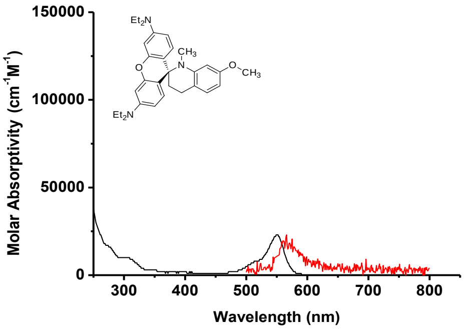

Supplementary Figure 51. The absorption (black) and fluorescence emission spectra (red) of **RD52** in pH=7.4 PBS with 1% DMSO as a co-solvent.

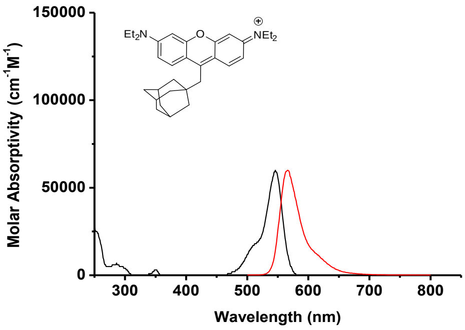

Supplementary Figure 52. The absorption (black) and fluorescence emission spectra (red) of **RD53** in pH=7.4 PBS with 1% DMSO as a co-solvent.

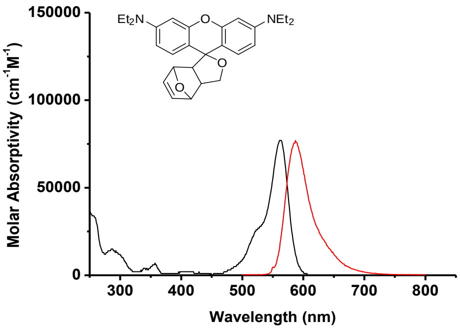

Supplementary Figure 53. The absorption (black) and fluorescence emission spectra (red) of **RD54** in pH=7.4 PBS with 1% DMSO as a co-solvent.

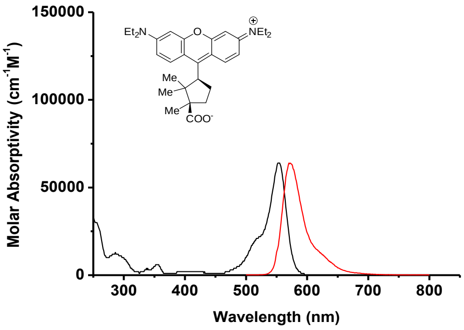

Supplementary Figure 54. The absorption (black) and fluorescence emission spectra (red) of **RD55** in pH=7.4 PBS with 1% DMSO as a co-solvent.

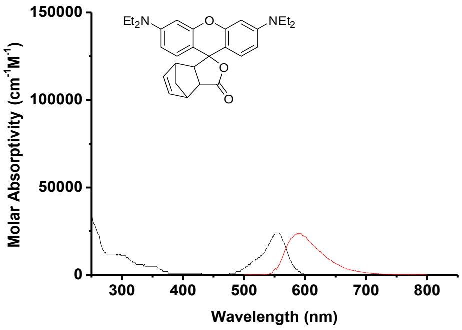

Supplementary Figure 55. The absorption (black) and fluorescence emission spectra (red) of **RD56** in pH=7.4 PBS with 1% DMSO as a co-solvent

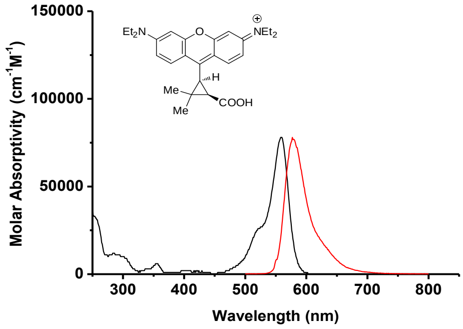

Supplementary Figure 56. The absorption (black) and fluorescence emission spectra (red) of **RD57** in pH=7.4 PBS with 1% DMSO as a co-solvent.

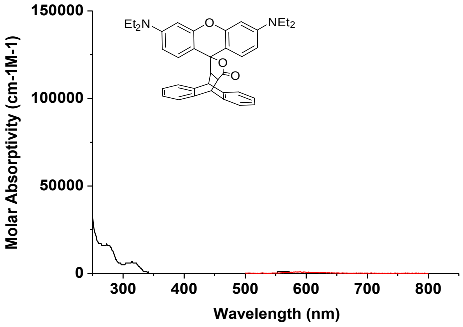

Supplementary Figure 57. The absorption (black) and fluorescence emission spectra (red) of **RD58** in pH=7.4 PBS with 1% DMSO as a co-solvent.

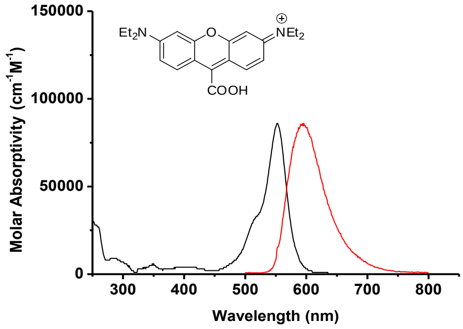

Supplementary Figure 58. The absorption (black) and fluorescence emission spectra (red) of **RD59** in pH=7.4 PBS with 1% DMSO as a co-solvent.

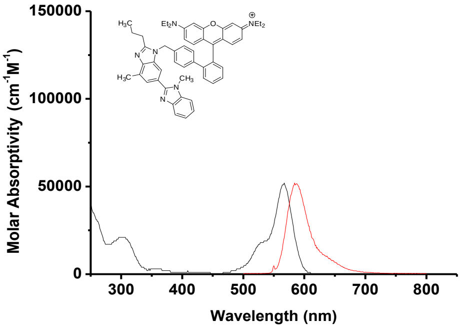

Supplementary Figure 59. The absorption (black) and fluorescence emission spectra (red) of **RD60** in pH=7.4 PBS with 1% DMSO as a co-solvent.

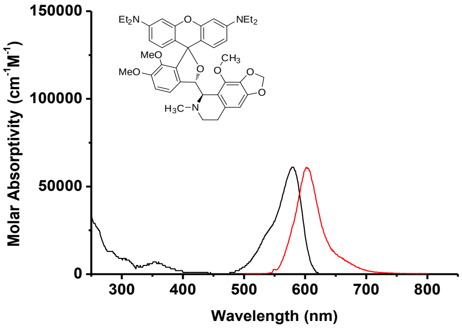

Supplementary Figure 60. The absorption (black) and fluorescence emission spectra (red) of **RD61** in pH=7.4 PBS with 1% DMSO as a co-solvent.

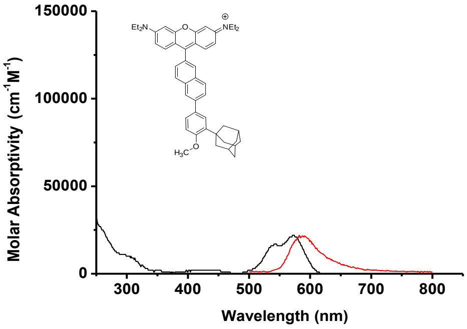

Supplementary Figure 61. The absorption (black) and fluorescence emission spectra (red) of **RD62** in pH=7.4 PBS with 1% DMSO as a co-solvent.

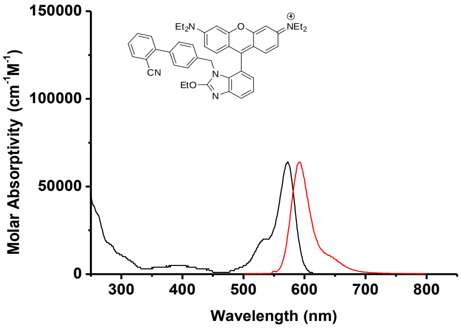

Supplementary Figure 62. The absorption (black) and fluorescence emission spectra (red) of **RD63** in pH=7.4 PBS with 1% DMSO as a co-solvent.

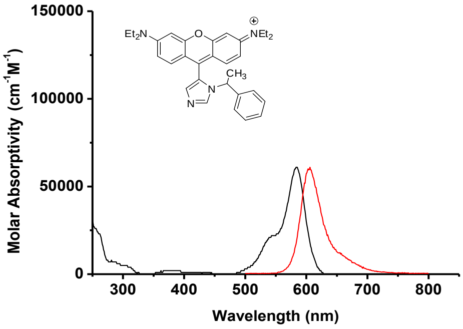

Supplementary Figure 63. The absorption (black) and fluorescence emission spectra (red) of **RD64** in pH=7.4 PBS with 1% DMSO as a co-solvent.

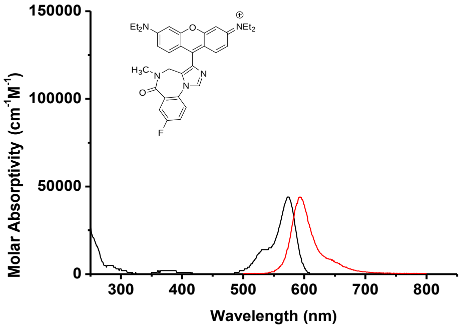

Supplementary Figure 64. The absorption (black) and fluorescence emission spectra (red) of **RD65** in pH=7.4 PBS with 1% DMSO as a co-solvent.

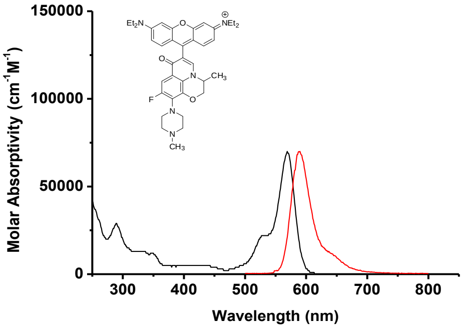

Supplementary Figure 65. The absorption (black) and fluorescence emission spectra (red) of **RD66** in pH=7.4 PBS with 1% DMSO as a co-solvent.

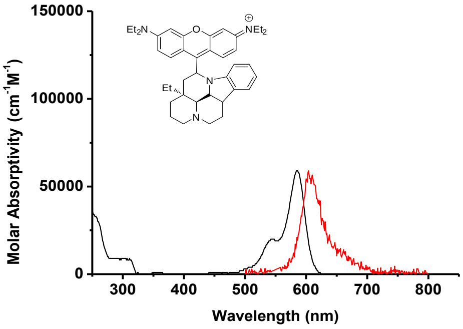

Supplementary Figure 66. The absorption (black) and fluorescence emission spectra (red) of **RD67** in pH=7.4 PBS with 1% DMSO as a co-solvent.

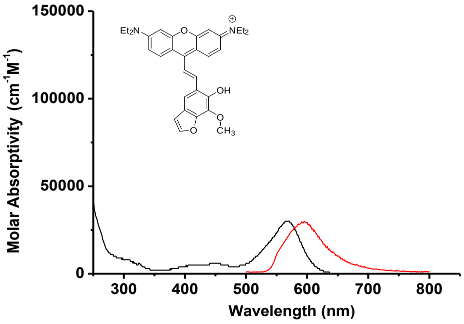

Supplementary Figure 67. The absorption (black) and fluorescence emission spectra (red) of **RD68** in pH=7.4 PBS with 1% DMSO as a co-solvent.

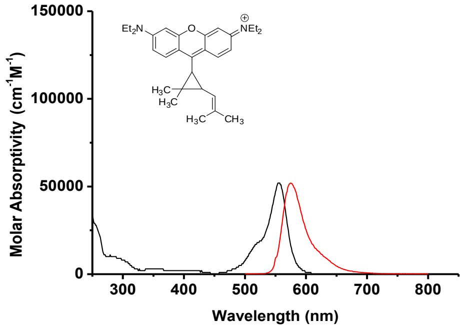

Supplementary Figure 68. The absorption (black) and fluorescence emission spectra (red) of **RD69** in pH=7.4 PBS with 1% DMSO as a co-solvent.

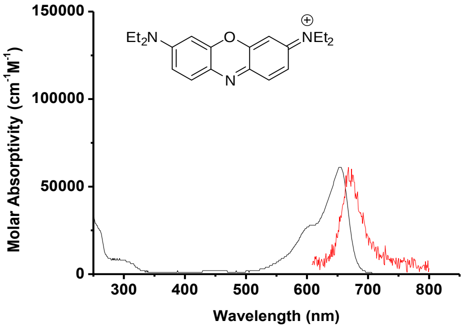

Supplementary Figure 69. The absorption (black) and fluorescence emission spectra (red) of **RD70** in pH=7.4 PBS with 1% DMSO as a co-solvent.

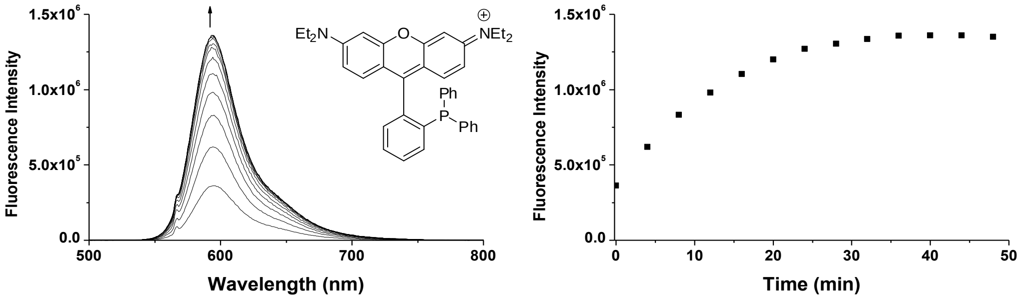

Supplementary Figure 70. Fluorescence titration of 10 μM **RD17** in PBS (50 mM, pH=7.4) in the presence of 10 μM Angeli's salt as a donor of HNO. The fluorescence intensity at 595 nm was measured every 5 min with excitation at 567 nm.

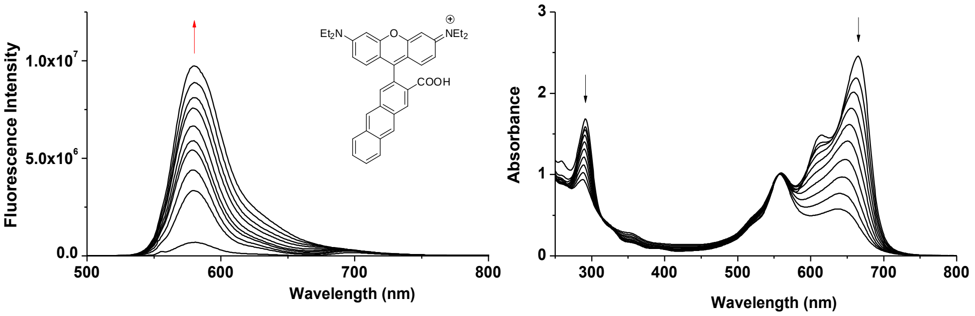

Supplementary Figure 71. Fluorescence and absorbance changes of **RD28** in PBS (50mM, pH=7.4) upon photoirradiation of codissolved methylene blue for 45 min (5 min interval). Red arrows indicates fluorescence increase due to the peroxidation of **RD28** and black arrows indicate absorbance changes of methylene blue. [**RD28**]= 10 μM, [methylene blue]= 30 μM, and 630 nm irradiation with magnetic stirring was used for $^1O_2$ formation.

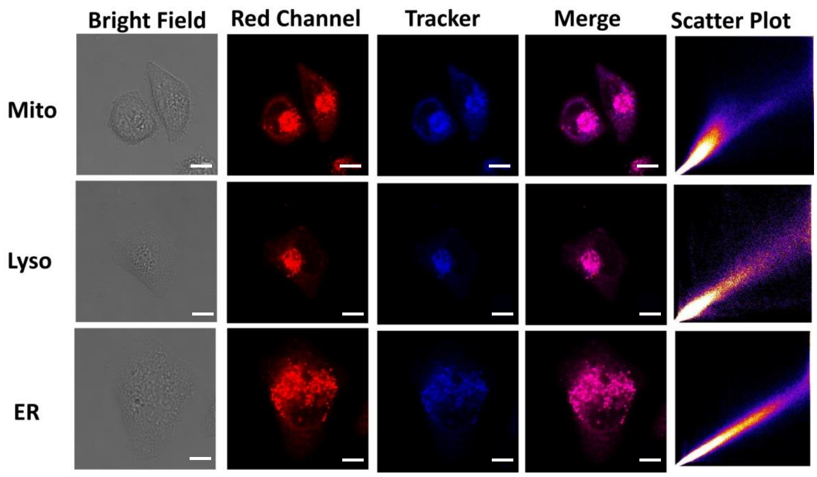

Supplementary Figure 72. Confocal fluorescence images for intracellular localization of **RD7** in HeLa cells. Cells were treated with 1 μM **RD7** for 0.5 h and stained (0.5 h) with 100 nM Mito-Tracker Green or 100 nM Lyso-Tracker DND-26 or 100 nM ER-Tracker Blue-White DPX. (Blue channel emission was collected in 410-480 nm upon excitation at 405 nm for ER-Tracker Blue-White DPX, pseudo blue, green channel emission was collected in 505-550 nm upon excitation at 488 nm for Mito-Tracker Green and Lyso-Tracker Green, pseudo blue, and red channel emission was collected in 565-680 nm upon excitation at 561 nm for **RD7**, pseudo red). Scale bar: 10 μm.

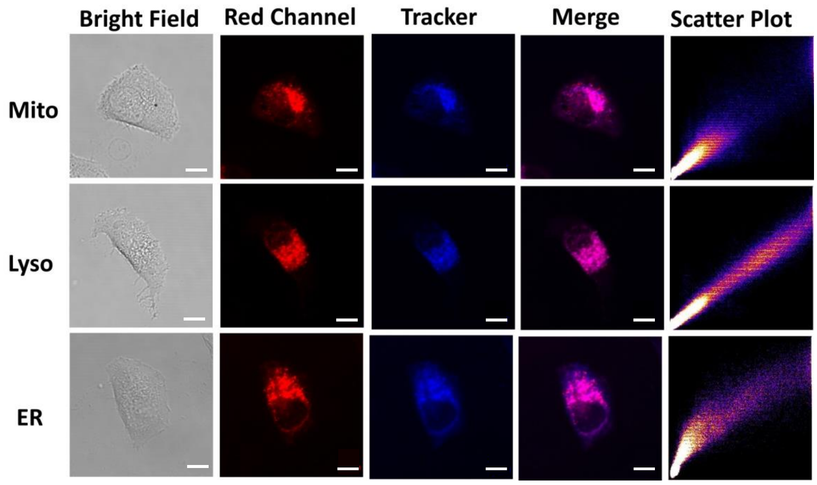

Supplementary Figure 73. Confocal fluorescence images for intracellular localization of **RD8** in HeLa cells. Cells were treated with 1 μM **RD8** for 0.5 h and stained (0.5 h) with 100 nM Mito-Tracker Green or 100 nM Lyso-Tracker DND-26 or 100 nM ER-Tracker Blue-White DPX. (Blue channel emission was collected in 410-480 nm upon excitation at 405 nm for ER-Tracker Blue-White DPX, pseudo blue, green channel emission was collected in 505-550 nm upon excitation at 488 nm for Mito-Tracker Green and Lyso-Tracker Green, pseudo blue, and red channel emission was collected in 565-680 nm upon excitation at 561 nm for **RD8**, pseudo red). Scale bar: 10 μm.

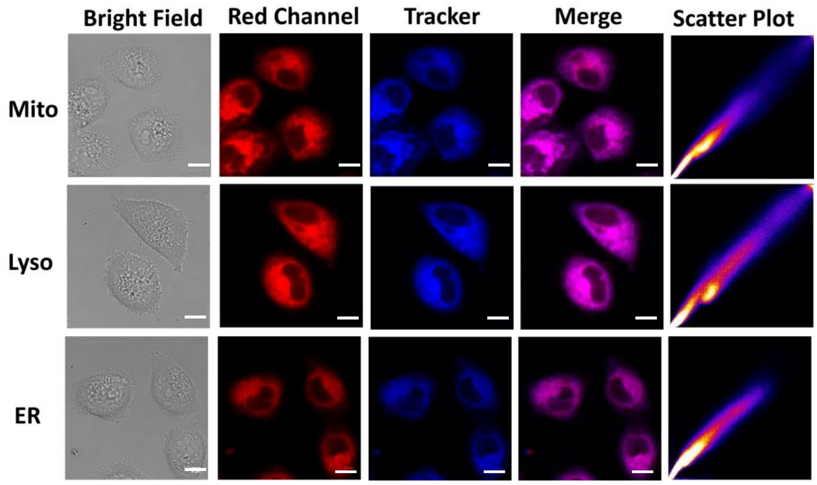

Supplementary Figure 74. Confocal fluorescence images for intracellular localization of **RD9** in HeLa cells. Cells were treated with 1 μM **RD9** for 0.5 h and stained (0.5 h) with 100 nM Mito-Tracker Green or 100 nM Lyso-Tracker DND-26 or 100 nM ER-Tracker Blue-White DPX. (Blue channel emission was collected in 410-480 nm upon excitation at 405 nm for ER-Tracker Blue-White DPX, pseudo blue, green channel emission was collected in 505-550 nm upon excitation at 488 nm for Mito-Tracker Green and Lyso-Tracker Green, pseudo blue, and red channel emission was collected in 565-680 nm upon excitation at 561 nm for **RD9**, pseudo red). Scale bar: 10 μm.

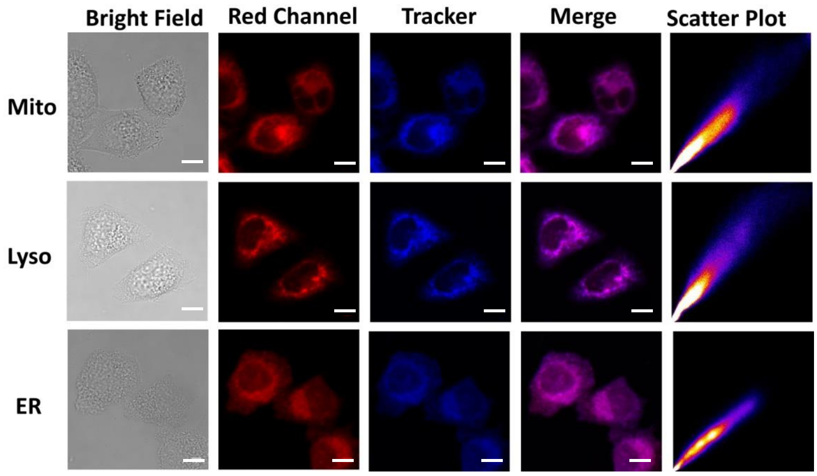

Supplementary Figure 75. Confocal fluorescence images for intracellular localization of **RD10** in HeLa cells. Cells were treated with 1 μM **RD10** for 0.5 h and stained (0.5 h) with 100 nM Mito-Tracker Green or 100 nM Lyso-Tracker DND-26 or 100 nM ER-Tracker Blue-White DPX. (Blue channel emission was collected in 410-480 nm upon excitation at 405 nm for ER-Tracker Blue-White DPX, pseudo blue, green channel emission was collected in 505-550 nm upon excitation at 488 nm for Mito-Tracker Green and Lyso-Tracker Green, pseudo blue, and red channel emission was collected in 565-680 nm upon excitation at 561 nm for **RD10**, pseudo red). Scale bar: 10 μm.

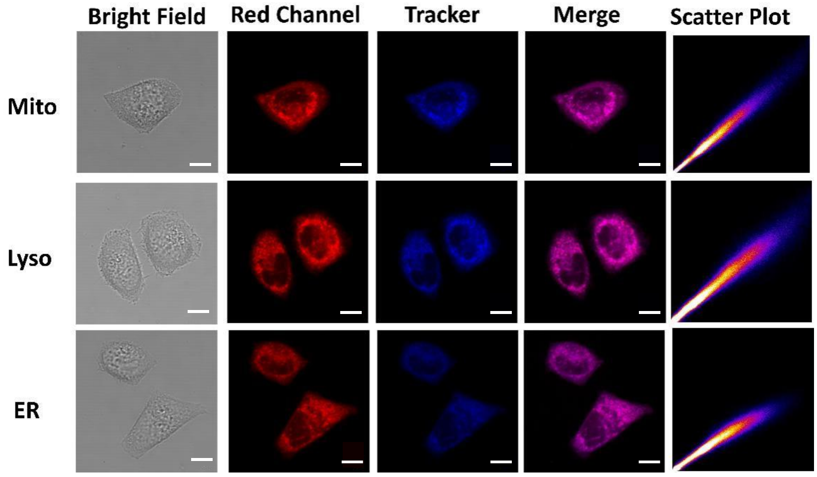

Supplementary Figure 76. Confocal fluorescence images for intracellular localization of **RD11** in HeLa cells. Cells were treated with 1 µM **RD11** for 0.5 h and stained (0.5 h) with 100 nM Mito-Tracker Green or 100 nM Lyso-Tracker DND-26 or 100 nM ER-Tracker Blue-White DPX. (Blue channel emission was collected in 410-480 nm upon excitation at 405 nm for ER-Tracker Blue-White DPX, pseudo blue, green channel emission was collected in 505-550 nm upon excitation at 488 nm for Mito-Tracker Green and Lyso-Tracker Green, pseudo blue, and red channel emission was collected in 565-680 nm upon excitation at 561 nm for **RD11**, pseudo red). Scale bar: 10 µm.

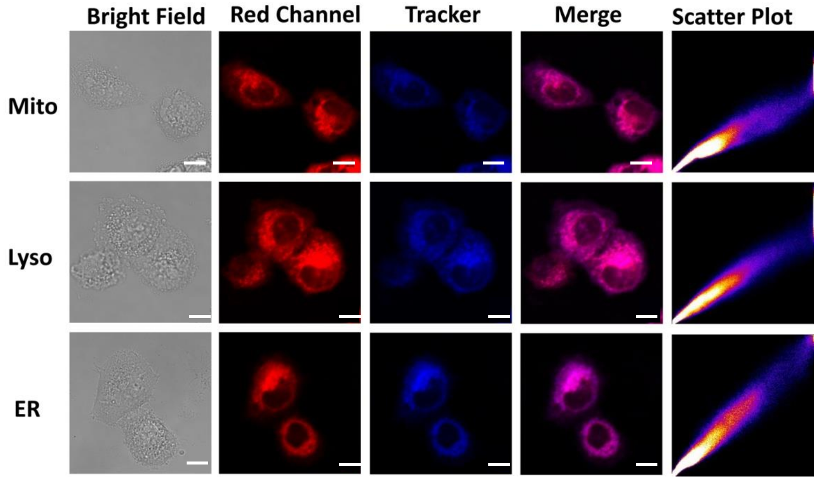

Supplementary Figure 77. Confocal fluorescence images for intracellular localization of **RD12** in HeLa cells. Cells were treated with 1 µM **RD12** for 0.5 h and stained (0.5 h) with 100 nM Mito-Tracker Green or 100 nM Lyso-Tracker DND-26 or 100 nM ER-Tracker Blue-White DPX. (Blue channel emission was collected in 410-480 nm upon excitation at 405 nm for ER-Tracker Blue-White DPX, pseudo blue, green channel emission was collected in 505-550 nm upon excitation at 488 nm for Mito-Tracker Green and Lyso-Tracker Green, pseudo blue, and red channel emission was collected in 565-680 nm upon excitation at 561 nm for **RD12**, pseudo red). Scale bar: 10 µm.

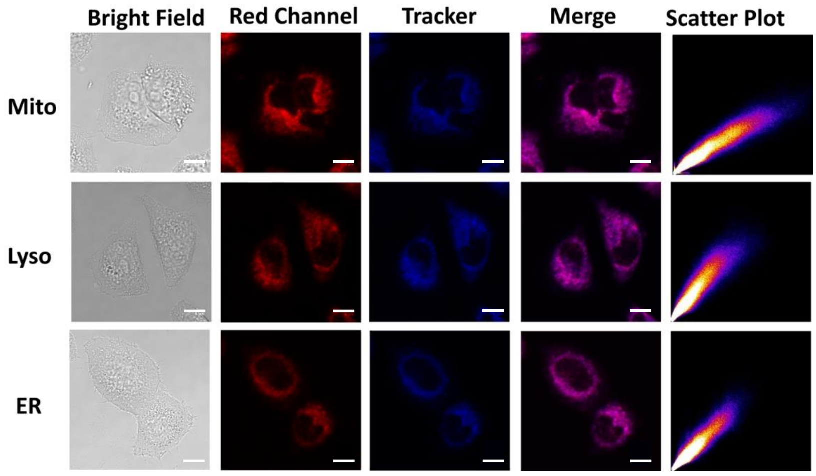

Supplementary Figure 78. Confocal fluorescence images for intracellular localization of **RD13** in HeLa cells. Cells were treated with 1 μM **RD13** for 0.5 h and stained (0.5 h) with 100 nM Mito-Tracker Green or 100 nM Lyso-Tracker DND-26 or 100 nM ER-Tracker Blue-White DPX. (Blue channel emission was collected in 410-480 nm upon excitation at 405 nm for ER-Tracker Blue-White DPX, pseudo blue, green channel emission was collected in 505-550 nm upon excitation at 488 nm for Mito-Tracker Green and Lyso-Tracker Green, pseudo blue, and red channel emission was collected in 565-680 nm upon excitation at 561 nm for **RD13**, pseudo red). Scale bar: 10 μm.

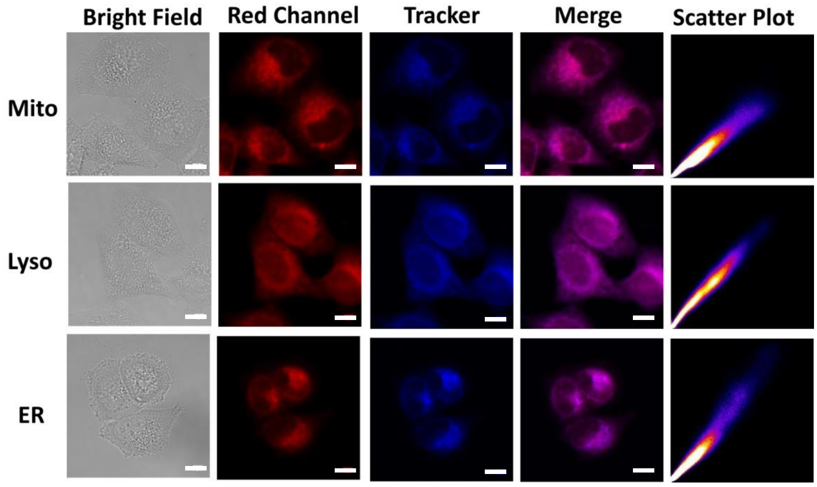

Supplementary Figure 79. Confocal fluorescence images for intracellular localization of **RD14** in HeLa cells. Cells were treated with 1 μM **RD14** for 0.5 h and stained (0.5 h) with 100 nM Mito-Tracker Green or 100 nM Lyso-Tracker DND-26 or 100 nM ER-Tracker Blue-White DPX. (Blue channel emission was collected in 410-480 nm upon excitation at 405 nm for ER-Tracker Blue-White DPX, pseudo blue, green channel emission was collected in 505-550 nm upon excitation at 488 nm for Mito-Tracker Green and Lyso-Tracker Green, pseudo blue, and red channel emission was collected in 565-680 nm upon excitation at 561 nm for **RD14**, pseudo red). Scale bar: 10 μm.

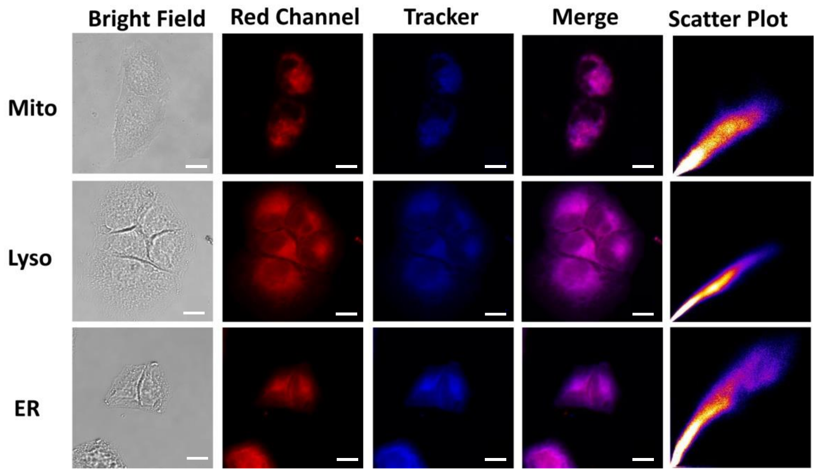

Supplementary Figure 80. Confocal fluorescence images for intracellular localization of **RD15** in A549 cells. Cells were treated with 1 μM **RD15** for 0.5 h and stained (0.5 h) with 100 nM Mito-Tracker Green or 100 nM Lyso-Tracker DND-26 or 100 nM ER-Tracker Blue-White DPX. (Blue channel emission was collected in 410-480 nm upon excitation at 405 nm for ER-Tracker Blue-White DPX, pseudo blue, green channel emission was collected in 505-550 nm upon excitation at 488 nm for Mito-Tracker Green and Lyso-Tracker Green, pseudo blue, and red channel emission was collected in 565-680 nm upon excitation at 561 nm for **RD15**, pseudo red). Scale bar: 10 μm.

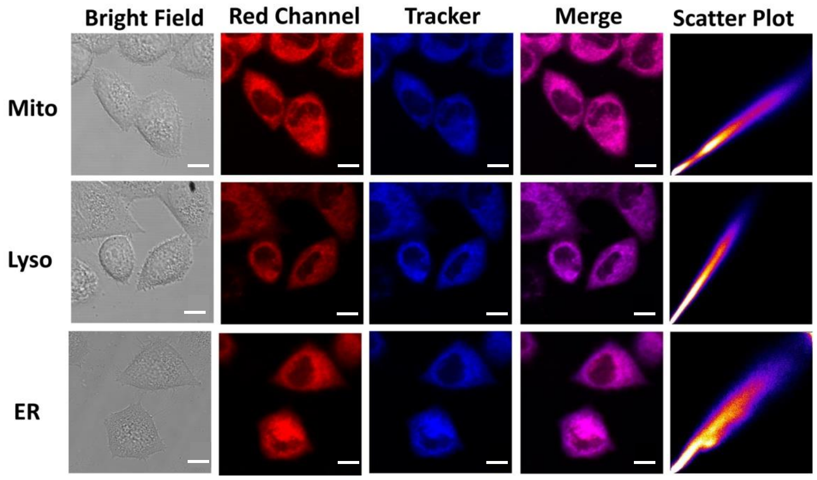

Supplementary Figure 81. Confocal fluorescence images for intracellular localization of **RD16** in HeLa cells. Cells were treated with 1 μM **RD16** for 0.5 h and stained (0.5 h) with 100 nM Mito-Tracker Green or 100 nM Lyso-Tracker DND-26 or 100 nM ER-Tracker Blue-White DPX. (Blue channel emission was collected in 410-480 nm upon excitation at 405 nm for ER-Tracker Blue-White DPX, pseudo blue, green channel emission was collected in 505-550 nm upon excitation at 488 nm for Mito-Tracker Green and Lyso-Tracker Green, pseudo blue, and red channel emission was collected in 565-680 nm upon excitation at 561 nm for **RD16**, pseudo red). Scale bar: 10 μm.

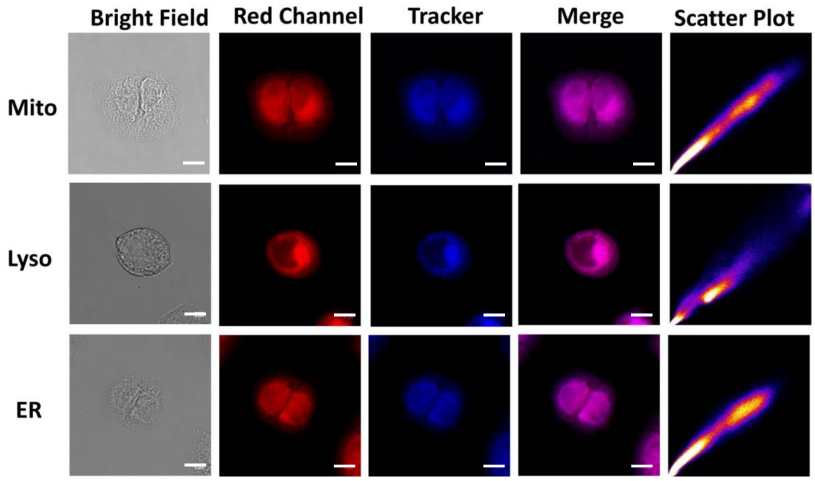

Supplementary Figure 82. Confocal fluorescence images for intracellular localization of **RD17** in A549 cells. Cells were treated with 1 μM **RD17** for 0.5 h and stained (0.5 h) with 100 nM Mito-Tracker Green or 100 nM Lyso-Tracker DND-26 or 100 nM ER-Tracker Blue-White DPX. (Blue channel emission was collected in 410-480 nm upon excitation at 405 nm for ER-Tracker Blue-White DPX, pseudo blue, green channel emission was collected in 505-550 nm upon excitation at 488 nm for Mito-Tracker Green and Lyso-Tracker Green, pseudo blue, and red channel emission was collected in 565-680 nm upon excitation at 561 nm for **RD17**, pseudo red). Scale bar: 10 μm.

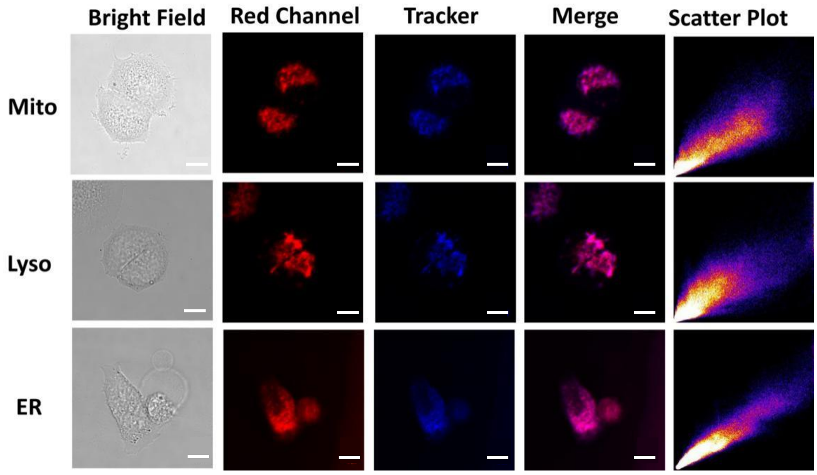

Supplementary Figure 83. Confocal fluorescence images for intracellular localization of **RD18** in A549 cells. Cells were treated with 1 μM **RD18** for 0.5 h and stained (0.5 h) with 100 nM Mito-Tracker Green or 100 nM Lyso-Tracker DND-26 or 100 nM ER-Tracker Blue-White DPX. (Blue channel emission was collected in 410-480 nm upon excitation at 405 nm for ER-Tracker Blue-White DPX, pseudo blue, green channel emission was collected in 505-550 nm upon excitation at 488 nm for Mito-Tracker Green and Lyso-Tracker Green, pseudo blue, and red channel emission was collected in 565-680 nm upon excitation at 561 nm for **RD18**, pseudo red). Scale bar: 10 μm.

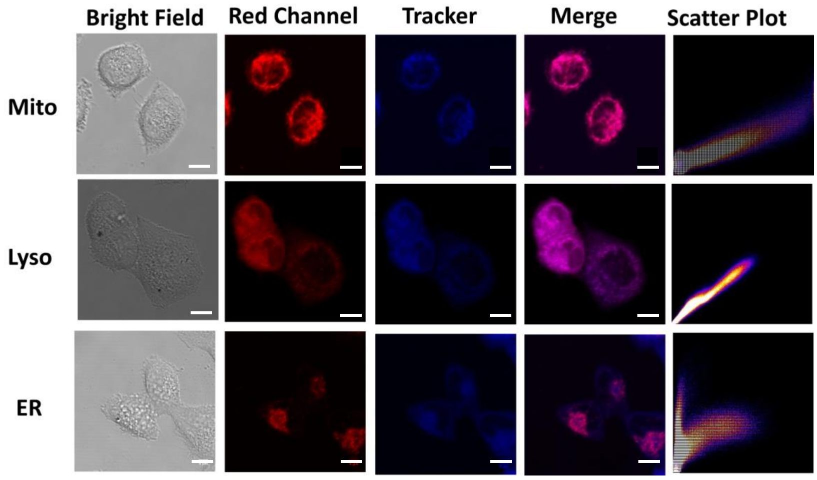

Supplementary Figure 84. Confocal fluorescence images for intracellular localization of **RD19** in HeLa cells. Cells were treated with 1 μM **RD19** for 0.5 h and stained (0.5 h) with 100 nM Mito-Tracker Green or 100 nM Lyso-Tracker DND-26 or 100 nM ER-Tracker Blue-White DPX. (Blue channel emission was collected in 410-480 nm upon excitation at 405 nm for ER-Tracker Blue-White DPX, pseudo blue, green channel emission was collected in 505-550 nm upon excitation at 488 nm for Mito-Tracker Green and Lyso-Tracker Green, pseudo blue, and red channel emission was collected in 565-680 nm upon excitation at 561 nm for **RD19**, pseudo red). Scale bar: 10 μm.

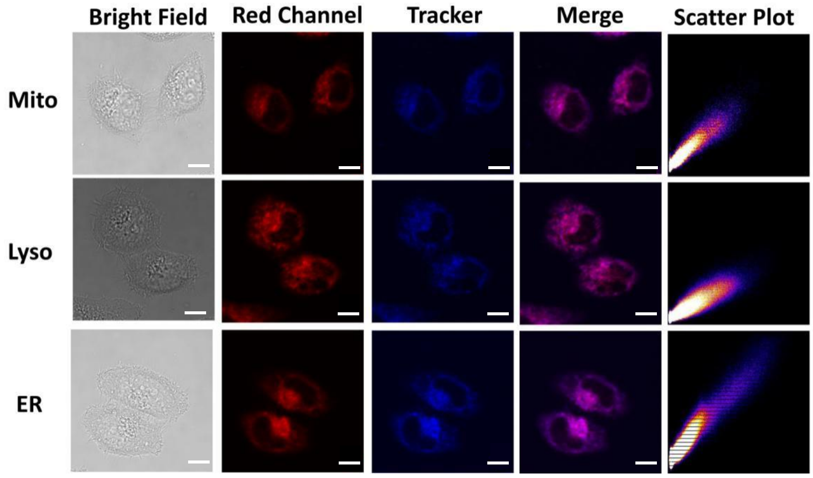

Supplementary Figure 85. Confocal fluorescence images for intracellular localization of **RD20** in HeLa cells. Cells were treated with 1 μM **RD20** for 0.5 h and stained (0.5 h) with 100 nM Mito-Tracker Green or 100 nM Lyso-Tracker DND-26 or 100 nM ER-Tracker Blue-White DPX. (Blue channel emission was collected in 410-480 nm upon excitation at 405 nm for ER-Tracker Blue-White DPX, pseudo blue, green channel emission was collected in 505-550 nm upon excitation at 488 nm for Mito-Tracker Green and Lyso-Tracker Green, pseudo blue, and red channel emission was collected in 565-680 nm upon excitation at 561 nm for **RD20**, pseudo red). Scale bar: 10 μm.

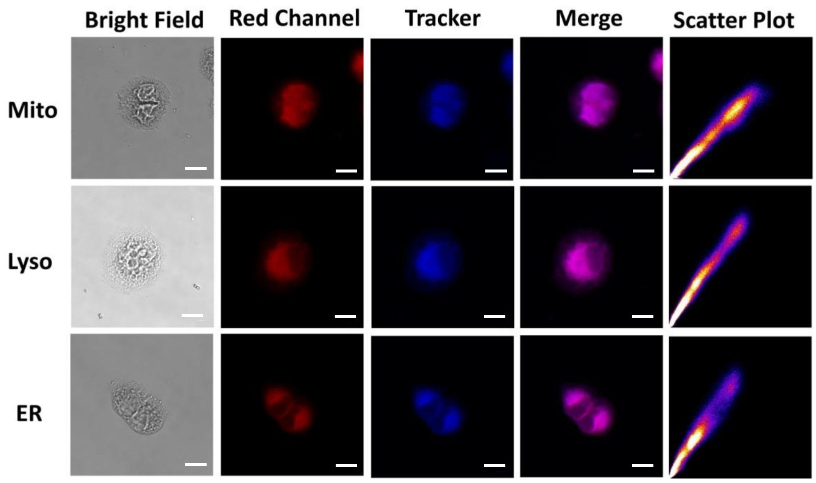

Supplementary Figure 86. Confocal fluorescence images for intracellular localization of **RD21** in A549 cells. Cells were treated with 1 μM **RD21** for 0.5 h and stained (0.5 h) with 100 nM Mito-Tracker Green or 100 nM Lyso-Tracker DND-26 or 100 nM ER-Tracker Blue-White DPX. (Blue channel emission was collected in 410-480 nm upon excitation at 405 nm for ER-Tracker Blue-White DPX, pseudo blue, green channel emission was collected in 505-550 nm upon excitation at 488 nm for Mito-Tracker Green and Lyso-Tracker Green, pseudo blue, and red channel emission was collected in 565-680 nm upon excitation at 561 nm for **RD21**, pseudo red). Scale bar: 10 μm.

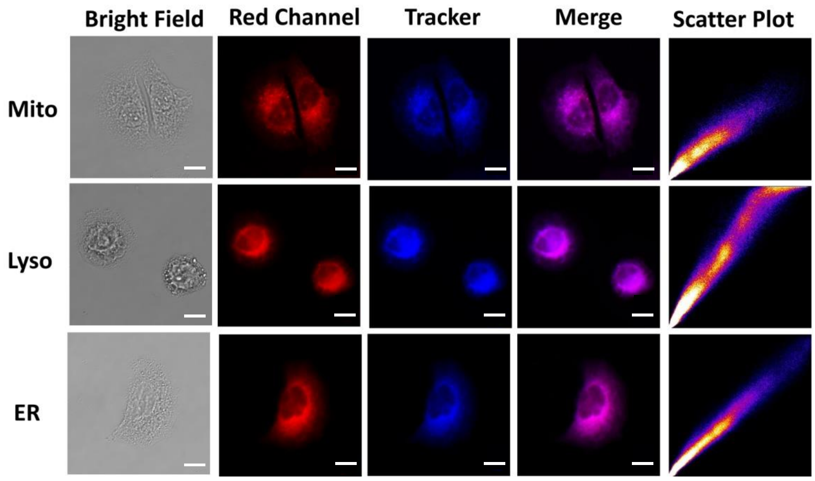

Supplementary Figure 87. Confocal fluorescence images for intracellular localization of **RD22** in A549 cells. Cells were treated with 1 μM **RD22** for 0.5 h and stained (0.5 h) with 100 nM Mito-Tracker Green or 100 nM Lyso-Tracker DND-26 or 100 nM ER-Tracker Blue-White DPX. (Blue channel emission was collected in 410-480 nm upon excitation at 405 nm for ER-Tracker Blue-White DPX, pseudo blue, green channel emission was collected in 505-550 nm upon excitation at 488 nm for Mito-Tracker Green and Lyso-Tracker Green, pseudo blue, and red channel emission was collected in 565-680 nm upon excitation at 561 nm for **RD22**, pseudo red). Scale bar: 10 μm.

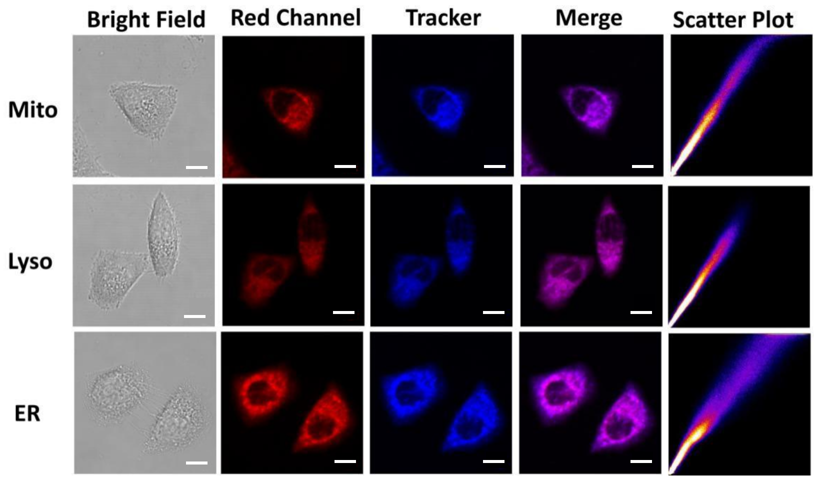

Supplementary Figure 88. Confocal fluorescence images for intracellular localization of **RD23** in HeLa cells. Cells were treated with 1 μM **RD23** for 0.5 h and stained (0.5 h) with 100 nM Mito-Tracker Green or 100 nM Lyso-Tracker DND-26 or 100 nM ER-Tracker Blue-White DPX. (Blue channel emission was collected in 410-480 nm upon excitation at 405 nm for ER-Tracker Blue-White DPX, pseudo blue, green channel emission was collected in 505-550 nm upon excitation at 488 nm for Mito-Tracker Green and Lyso-Tracker Green, pseudo blue, and red channel emission was collected in 565-680 nm upon excitation at 561 nm for **RD23**, pseudo red). Scale bar: 10 μm.

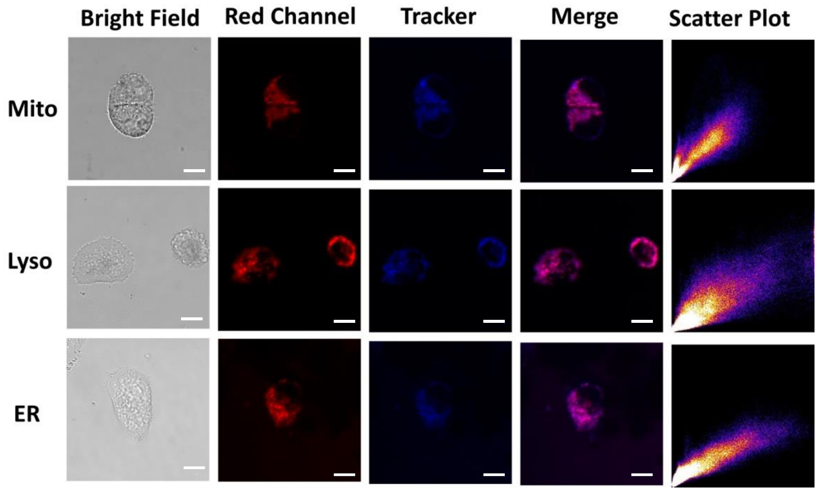

Supplementary Figure 89. Confocal fluorescence images for intracellular localization of **RD24** in A549 cells. Cells were treated with 1 μM **RD24** for 0.5 h and stained (0.5 h) with 100 nM Mito-Tracker Green or 100 nM Lyso-Tracker DND-26 or 100 nM ER-Tracker Blue-White DPX. (Blue channel emission was collected in 410-480 nm upon excitation at 405 nm for ER-Tracker Blue-White DPX, pseudo blue, green channel emission was collected in 505-550 nm upon excitation at 488 nm for Mito-Tracker Green and Lyso-Tracker Green, pseudo blue, and red channel emission was collected in 565-680 nm upon excitation at 561 nm for **RD24**, pseudo red). Scale bar: 10 μm.

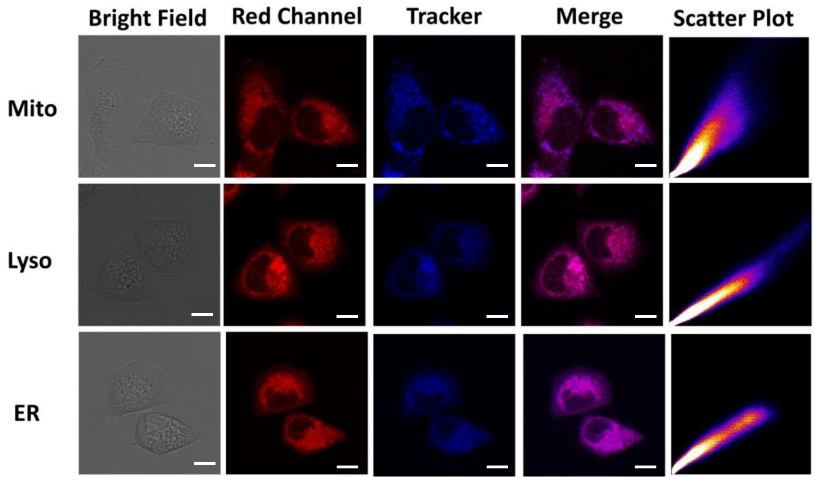

Supplementary Figure 90. Confocal fluorescence images for intracellular localization of **RD26** in HeLa cells. Cells were treated with 1 μM **RD26** for 0.5 h and stained (0.5 h) with 100 nM Mito-Tracker Green or 100 nM Lyso-Tracker DND-26 or 100 nM ER-Tracker Blue-White DPX. (Blue channel emission was collected in 410-480 nm upon excitation at 405 nm for ER-Tracker Blue-White DPX, pseudo blue, green channel emission was collected in 505-550 nm upon excitation at 488 nm for Mito-Tracker Green and Lyso-Tracker Green, pseudo blue, and red channel emission was collected in 565-680 nm upon excitation at 561 nm for **RD26**, pseudo red). Scale bar: 10 μm.

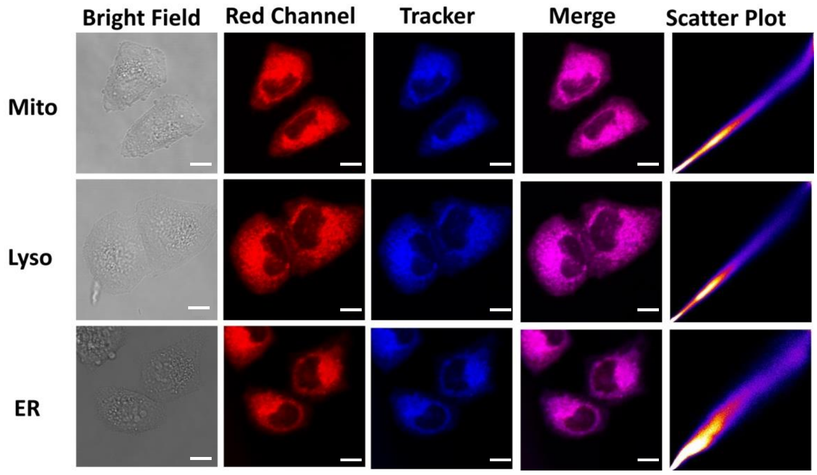

Supplementary Figure 91. Confocal fluorescence images for intracellular localization of **RD27** in HeLa cells. Cells were treated with 1 μM **RD27** for 0.5 h and stained (0.5 h) with 100 nM Mito-Tracker Green or 100 nM Lyso-Tracker DND-26 or 100 nM ER-Tracker Blue-White DPX. (Blue channel emission was collected in 410-480 nm upon excitation at 405 nm for ER-Tracker Blue-White DPX, pseudo blue, green channel emission was collected in 505-550 nm upon excitation at 488 nm for Mito-Tracker Green and Lyso-Tracker Green, pseudo blue, and red channel emission was collected in 565-680 nm upon excitation at 561 nm for **RD27**, pseudo red). Scale bar: 10 μm.

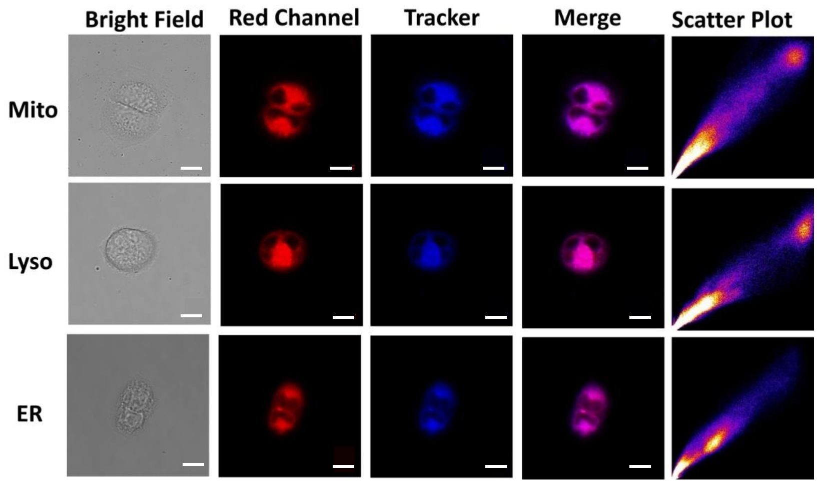

Supplementary Figure 92. Confocal fluorescence images for intracellular localization of **RD28** in A549 cells. Cells were treated with 1 μM **RD28** for 0.5 h and stained (0.5 h) with 100 nM Mito-Tracker Green or 100 nM Lyso-Tracker DND-26 or 100 nM ER-Tracker Blue-White DPX. (Blue channel emission was collected in 410-480 nm upon excitation at 405 nm for ER-Tracker Blue-White DPX, pseudo blue, green channel emission was collected in 505-550 nm upon excitation at 488 nm for Mito-Tracker Green and Lyso-Tracker Green, pseudo blue, and red channel emission was collected in 565-680 nm upon excitation at 561 nm for **RD28**, pseudo red). Scale bar: 10 μm.

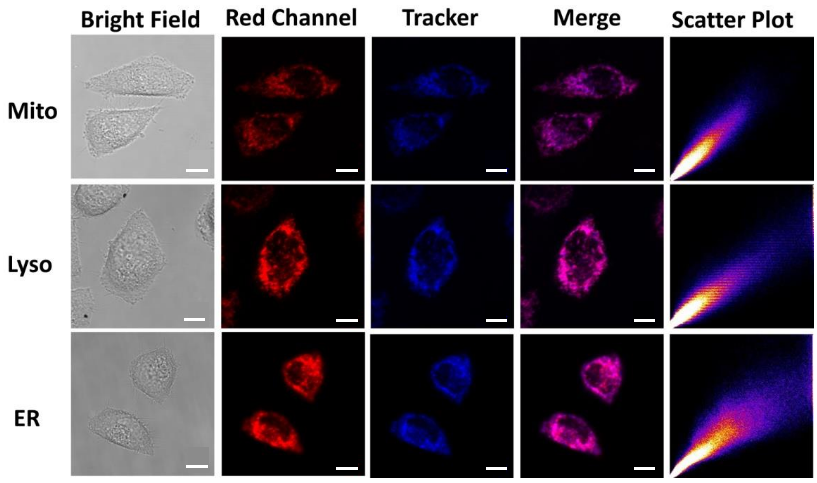

Supplementary Figure 93. Confocal fluorescence images for intracellular localization of **RD29** in HeLa cells. Cells were treated with 1 μM **RD29** for 0.5 h and stained (0.5 h) with 100 nM Mito-Tracker Green or 100 nM Lyso-Tracker DND-26 or 100 nM ER-Tracker Blue-White DPX. (Blue channel emission was collected in 410-480 nm upon excitation at 405 nm for ER-Tracker Blue-White DPX, pseudo blue, green channel emission was collected in 505-550 nm upon excitation at 488 nm for Mito-Tracker Green and Lyso-Tracker Green, pseudo blue, and red channel emission was collected in 565-680 nm upon excitation at 561 nm for **RD29**, pseudo red). Scale bar: 10 μm.

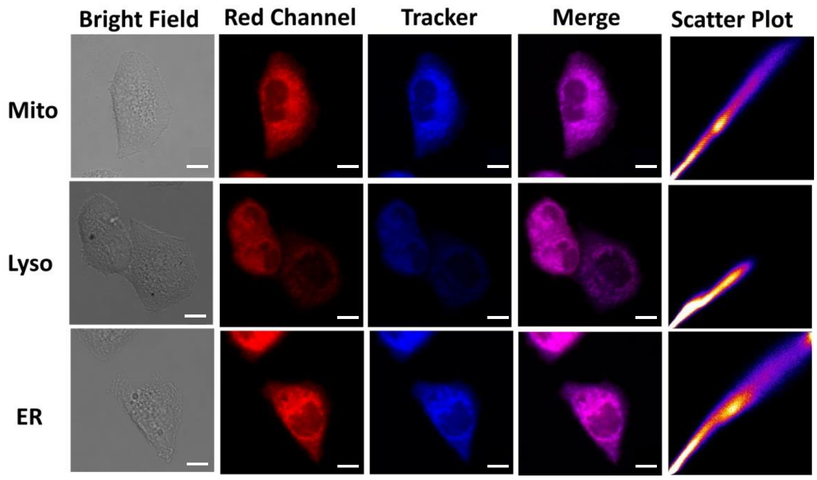

Supplementary Figure 94. Confocal fluorescence images for intracellular localization of **RD30** in HeLa cells. Cells were treated with 1 μM **RD30** for 0.5 h and stained (0.5 h) with 100 nM Mito-Tracker Green or 100 nM Lyso-Tracker DND-26 or 100 nM ER-Tracker Blue-White DPX. (Blue channel emission was collected in 410-480 nm upon excitation at 405 nm for ER-Tracker Blue-White DPX, pseudo blue, green channel emission was collected in 505-550 nm upon excitation at 488 nm for Mito-Tracker Green and Lyso-Tracker Green, pseudo blue, and red channel emission was collected in 565-680 nm upon excitation at 561 nm for **RD30**, pseudo red). Scale bar: 10 μm.

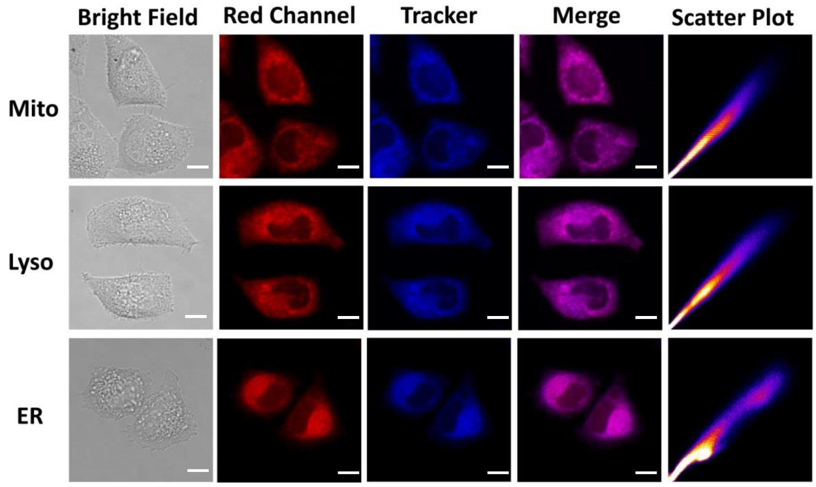

Supplementary Figure 95. Confocal fluorescence images for intracellular localization of **RD31** in HeLa cells. Cells were treated with 1 μM **RD31** for 0.5 h and stained (0.5 h) with 100 nM Mito-Tracker Green or 100 nM Lyso-Tracker DND-26 or 100 nM ER-Tracker Blue-White DPX. (Blue channel emission was collected in 410-480 nm upon excitation at 405 nm for ER-Tracker Blue-White DPX, pseudo blue, green channel emission was collected in 505-550 nm upon excitation at 488 nm for Mito-Tracker Green and Lyso-Tracker Green, pseudo blue, and red channel emission was collected in 565-680 nm upon excitation at 561 nm for **RD31**, pseudo red). Scale bar: 10 μm.

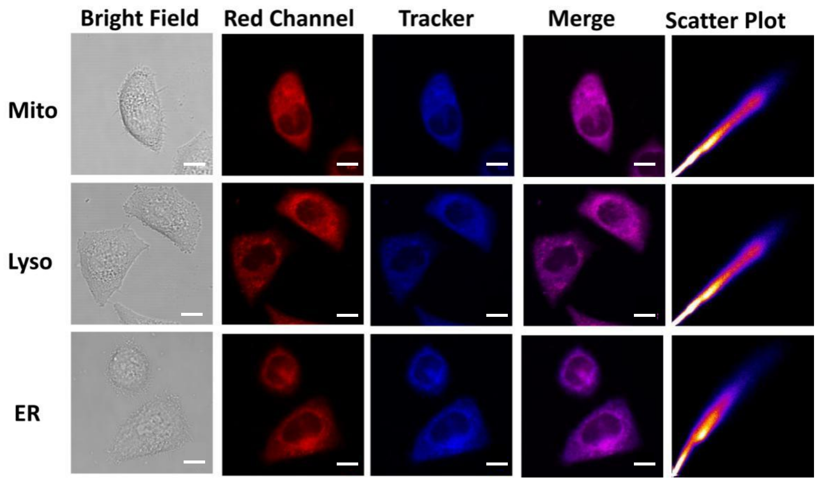

Supplementary Figure 96. Confocal fluorescence images for intracellular localization of **RD32** in HeLa cells. Cells were treated with 1 μM **RD32** for 0.5 h and stained (0.5 h) with 100 nM Mito-Tracker Green or 100 nM Lyso-Tracker DND-26 or 100 nM ER-Tracker Blue-White DPX. (Blue channel emission was collected in 410-480 nm upon excitation at 405 nm for ER-Tracker Blue-White DPX, pseudo blue, green channel emission was collected in 505-550 nm upon excitation at 488 nm for Mito-Tracker Green and Lyso-Tracker Green, pseudo blue, and red channel emission was collected in 565-680 nm upon excitation at 561 nm for **RD32**, pseudo red). Scale bar: 10 μm.

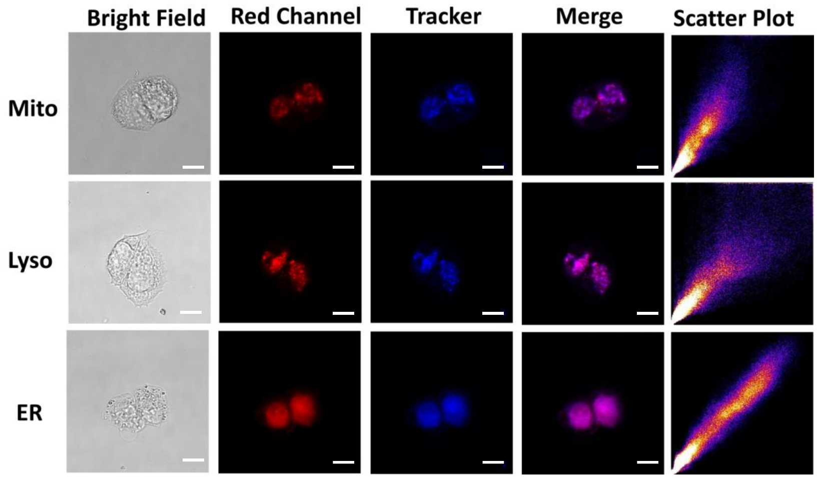

Supplementary Figure 97. Confocal fluorescence images for intracellular localization of **RD33** in A549 cells. Cells were treated with 1 μM **RD33** for 0.5 h and stained (0.5 h) with 100 nM Mito-Tracker Green or 100 nM Lyso-Tracker DND-26 or 100 nM ER-Tracker Blue-White DPX. (Blue channel emission was collected in 410-480 nm upon excitation at 405 nm for ER-Tracker Blue-White DPX, pseudo blue, green channel emission was collected in 505-550 nm upon excitation at 488 nm for Mito-Tracker Green and Lyso-Tracker Green, pseudo blue, and red channel emission was collected in 565-680 nm upon excitation at 561 nm for **RD33**, pseudo red). Scale bar: 10 μm.

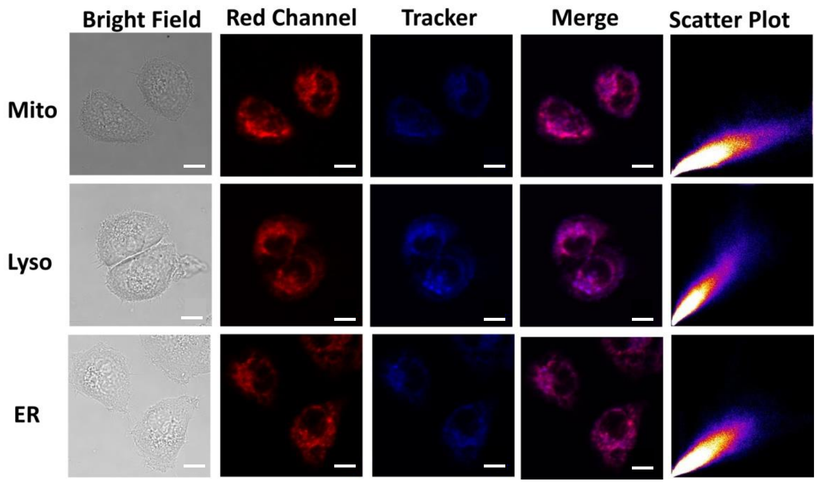

Supplementary Figure 98. Confocal fluorescence images for intracellular localization of **RD34** in HeLa cells. Cells were treated with 1 μM **RD34** for 0.5 h and stained (0.5 h) with 100 nM Mito-Tracker Green or 100 nM Lyso-Tracker DND-26 or 100 nM ER-Tracker Blue-White DPX. (Blue channel emission was collected in 410-480 nm upon excitation at 405 nm for ER-Tracker Blue-White DPX, pseudo blue, green channel emission was collected in 505-550 nm upon excitation at 488 nm for Mito-Tracker Green and Lyso-Tracker Green, pseudo blue, and red channel emission was collected in 565-680 nm upon excitation at 561 nm for **RD34**, pseudo red). Scale bar: 10 μm.

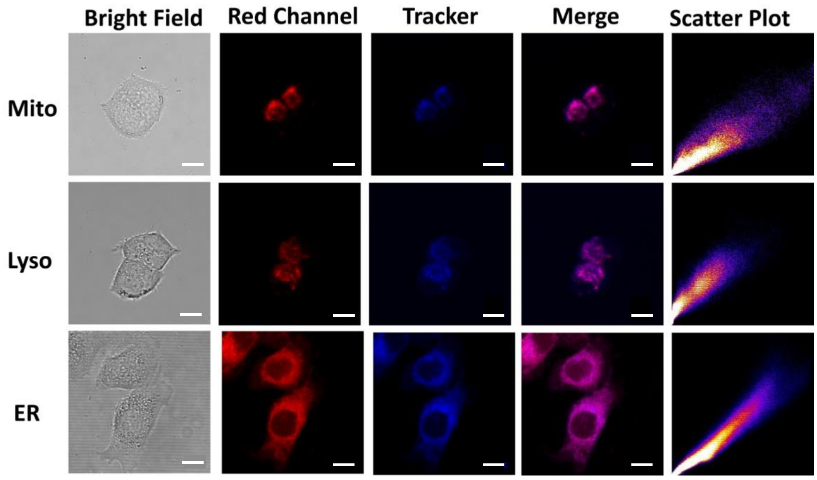

Supplementary Figure 99. Confocal fluorescence images for intracellular localization of **RD35** in A549 cells. Cells were treated with 1 μM **RD35** for 0.5 h and stained (0.5 h) with 100 nM Mito-Tracker Green or 100 nM Lyso-Tracker DND-26 or 100 nM ER-Tracker Blue-White DPX. (Blue channel emission was collected in 410-480 nm upon excitation at 405 nm for ER-Tracker Blue-White DPX, pseudo blue, green channel emission was collected in 505-550 nm upon excitation at 488 nm for Mito-Tracker Green and Lyso-Tracker Green, pseudo blue, and red channel emission was collected in 565-680 nm upon excitation at 561 nm for **RD35**, pseudo red). Scale bar: 10 μm.

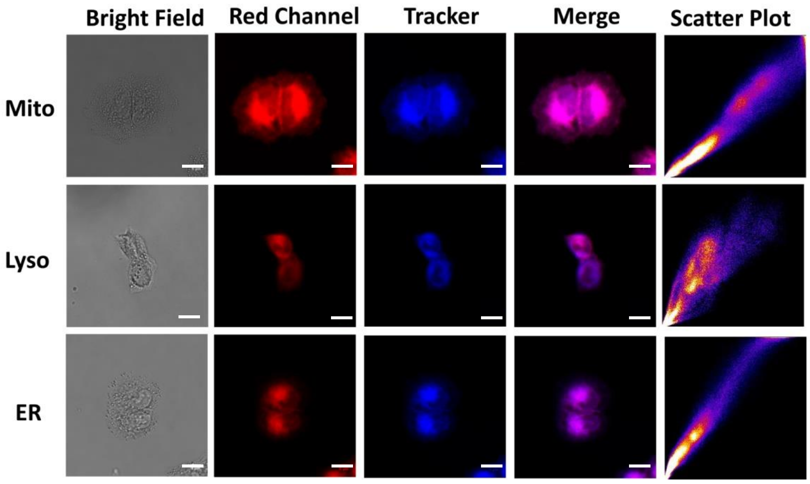

Supplementary Figure 100. Confocal fluorescence images for intracellular localization of **RD36** in A549 cells. Cells were treated with 1 μM **RD36** for 0.5 h and stained (0.5 h) with 100 nM Mito-Tracker Green or 100 nM Lyso-Tracker DND-26 or 100 nM ER-Tracker Blue-White DPX. (Blue channel emission was collected in 410-480 nm upon excitation at 405 nm for ER-Tracker Blue-White DPX, pseudo blue, green channel emission was collected in 505-550 nm upon excitation at 488 nm for Mito-Tracker Green and Lyso-Tracker Green, pseudo blue, and red channel emission was collected in 565-680 nm upon excitation at 561 nm for **RD36**, pseudo red). Scale bar: 10 μm.

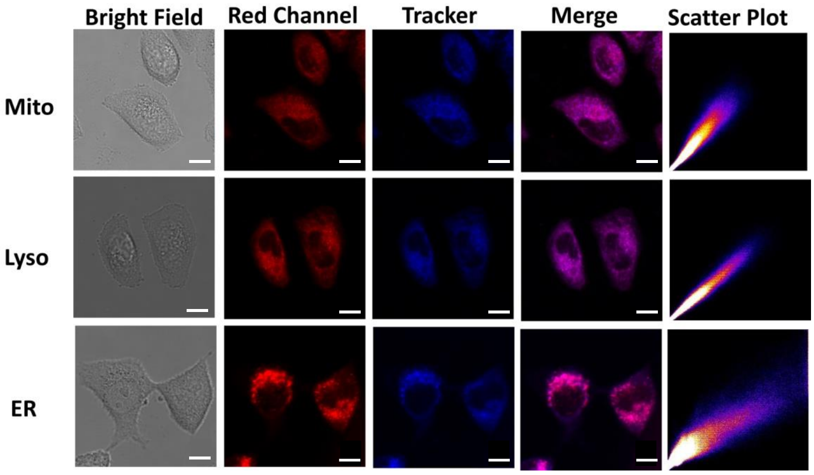

Supplementary Figure 101. Confocal fluorescence images for intracellular localization of **RD37** in HeLa cells. Cells were treated with 1 μM **RD37** for 0.5 h and stained (0.5 h) with 100 nM Mito-Tracker Green or 100 nM Lyso-Tracker DND-26 or 100 nM ER-Tracker Blue-White DPX. (Blue channel emission was collected in 410-480 nm upon excitation at 405 nm for ER-Tracker Blue-White DPX, pseudo blue, green channel emission was collected in 505-550 nm upon excitation at 488 nm for Mito-Tracker Green and Lyso-Tracker Green, pseudo blue, and red channel emission was collected in 565-680 nm upon excitation at 561 nm for **RD37**, pseudo red). Scale bar: 10 μm.

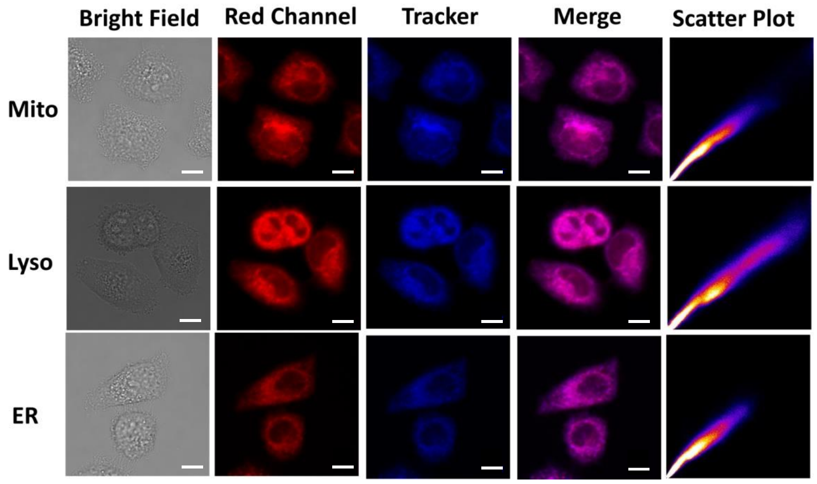

Supplementary Figure 102. Confocal fluorescence images for intracellular localization of **RD38** in A549 cells. Cells were treated with 1 μM **RD38** for 0.5 h and stained (0.5 h) with 100 nM Mito-Tracker Green or 100 nM Lyso-Tracker DND-26 or 100 nM ER-Tracker Blue-White DPX. (Blue channel emission was collected in 410-480 nm upon excitation at 405 nm for ER-Tracker Blue-White DPX, pseudo blue, green channel emission was collected in 505-550 nm upon excitation at 488 nm for Mito-Tracker Green and Lyso-Tracker Green, pseudo blue, and red channel emission was collected in 565-680 nm upon excitation at 561 nm for **RD38**, pseudo red). Scale bar: 10 μm.

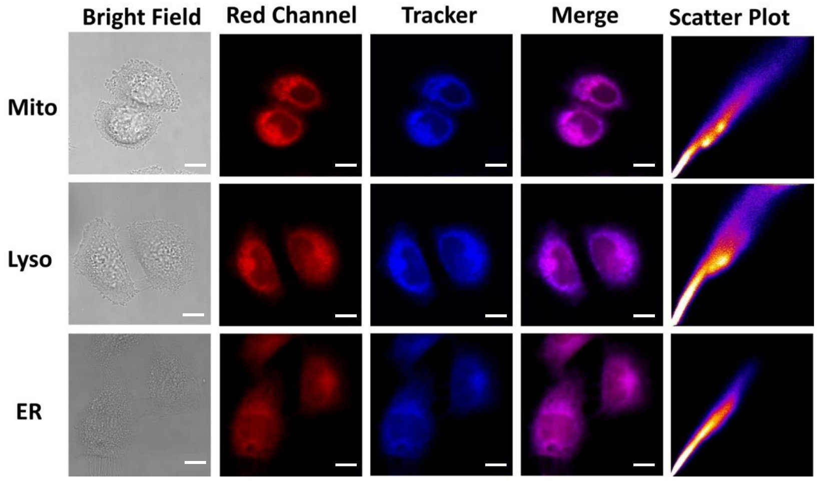

Supplementary Figure 103. Confocal fluorescence images for intracellular localization of **RD39** in HeLa cells. Cells were treated with 1 μM **RD39** for 0.5 h and stained (0.5 h) with 100 nM Mito-Tracker Green or 100 nM Lyso-Tracker DND-26 or 100 nM ER-Tracker Blue-White DPX. (Blue channel emission was collected in 410-480 nm upon excitation at 405 nm for ER-Tracker Blue-White DPX, pseudo blue, green channel emission was collected in 505-550 nm upon excitation at 488 nm for Mito-Tracker Green and Lyso-Tracker Green, pseudo blue, and red channel emission was collected in 565-680 nm upon excitation at 561 nm for **RD39**, pseudo red). Scale bar: 10 μm.

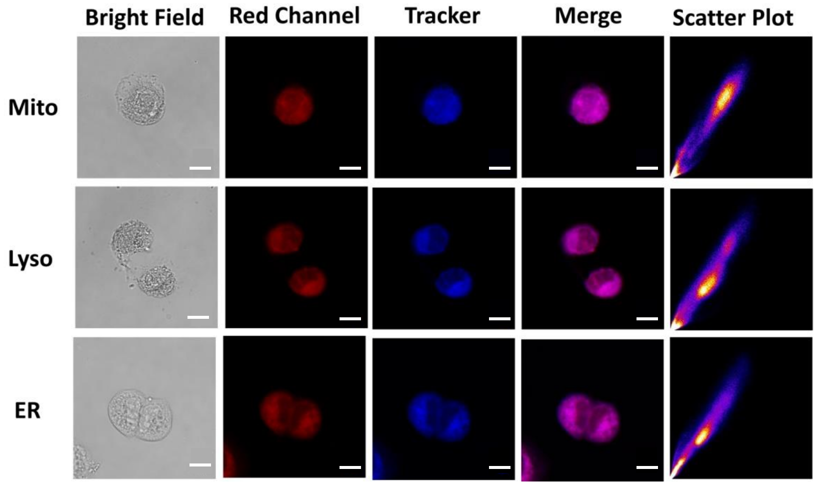

Supplementary Figure 104. Confocal fluorescence images for intracellular localization of **RD41** in A549 cells. Cells were treated with 1 µM **RD41** for 0.5 h and stained (0.5 h) with 100 nM Mito-Tracker Green or 100 nM Lyso-Tracker DND-26 or 100 nM ER-Tracker Blue-White DPX. (Blue channel emission was collected in 410-480 nm upon excitation at 405 nm for ER-Tracker Blue-White DPX, pseudo blue, green channel emission was collected in 505-550 nm upon excitation at 488 nm for Mito-Tracker Green and Lyso-Tracker Green, pseudo blue, and red channel emission was collected in 565-680 nm upon excitation at 561 nm for **RD41**, pseudo red). Scale bar: 10 µm.

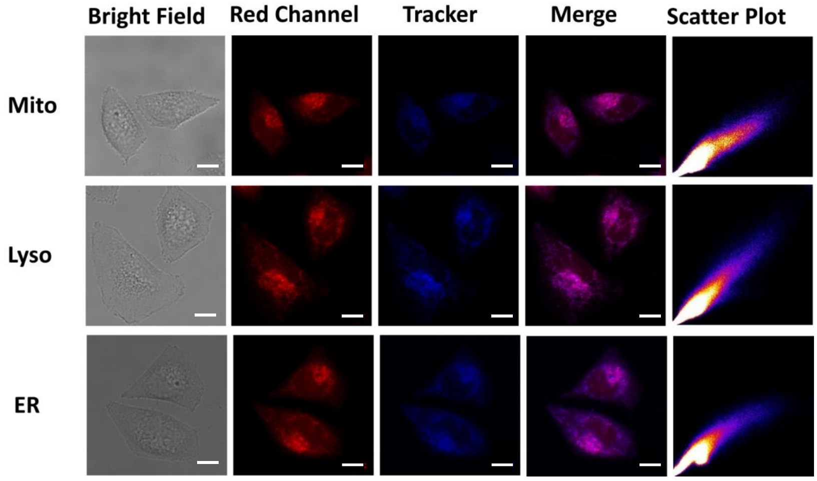

Supplementary Figure 105. Confocal fluorescence images for intracellular localization of **RD43** in HeLa cells. Cells were treated with 1 µM **RD43** for 0.5 h and stained (0.5 h) with 100 nM Mito-Tracker Green or 100 nM Lyso-Tracker DND-26 or 100 nM ER-Tracker Blue-White DPX. (Blue channel emission was collected in 410-480 nm upon excitation at 405 nm for ER-Tracker Blue-White DPX, pseudo blue, green channel emission was collected in 505-550 nm upon excitation at 488 nm for Mito-Tracker Green and Lyso-Tracker Green, pseudo blue, and red channel emission was collected in 565-680 nm upon excitation at 561 nm for **RD43**, pseudo red). Scale bar: 10 µm.

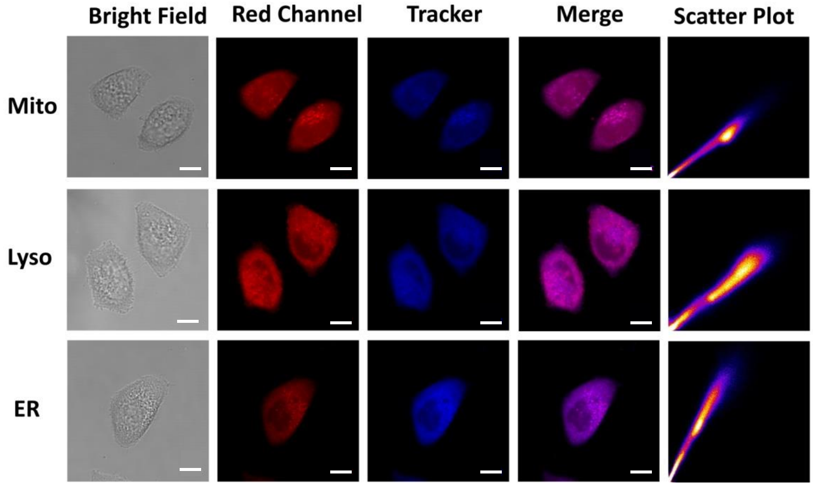

Supplementary Figure 106. Confocal fluorescence images for intracellular localization of **RD48** in HeLa cells. Cells were treated with 1 μM **RD48** for 0.5 h and stained (0.5 h) with 100 nM Mito-Tracker Green or 100 nM Lyso-Tracker DND-26 or 100 nM ER-Tracker Blue-White DPX. (Blue channel emission was collected in 410-480 nm upon excitation at 405 nm for ER-Tracker Blue-White DPX, pseudo blue, green channel emission was collected in 505-550 nm upon excitation at 488 nm for Mito-Tracker Green and Lyso-Tracker Green, pseudo blue, and red channel emission was collected in 565-680 nm upon excitation at 561 nm for **RD48**, pseudo red). Scale bar: 10 μm.

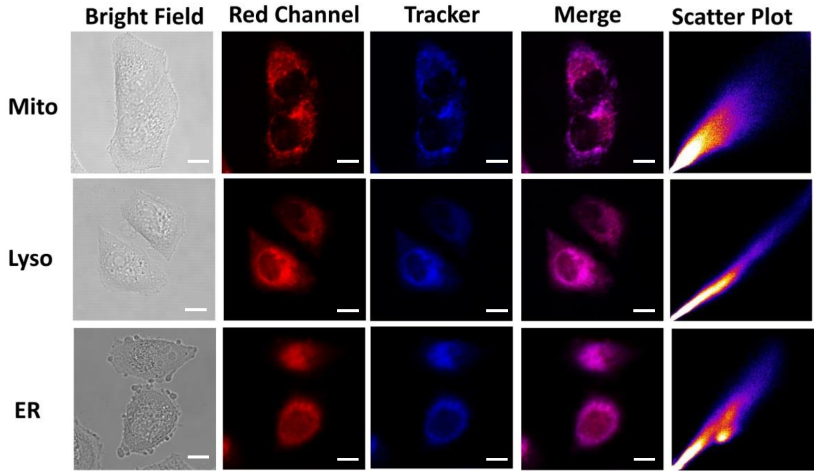

Supplementary Figure 107. Confocal fluorescence images for intracellular localization of **RD49** in HeLa cells. Cells were treated with 1 μM **RD49** for 0.5 h and stained (0.5 h) with 100 nM Mito-Tracker Green or 100 nM Lyso-Tracker DND-26 or 100 nM ER-Tracker Blue-White DPX. (Blue channel emission was collected in 410-480 nm upon excitation at 405 nm for ER-Tracker Blue-White DPX, pseudo blue, green channel emission was collected in 505-550 nm upon excitation at 488 nm for Mito-Tracker Green and Lyso-Tracker Green, pseudo blue, and red channel emission was collected in 565-680 nm upon excitation at 561 nm for **RD49**, pseudo red). Scale bar: 10 μm.

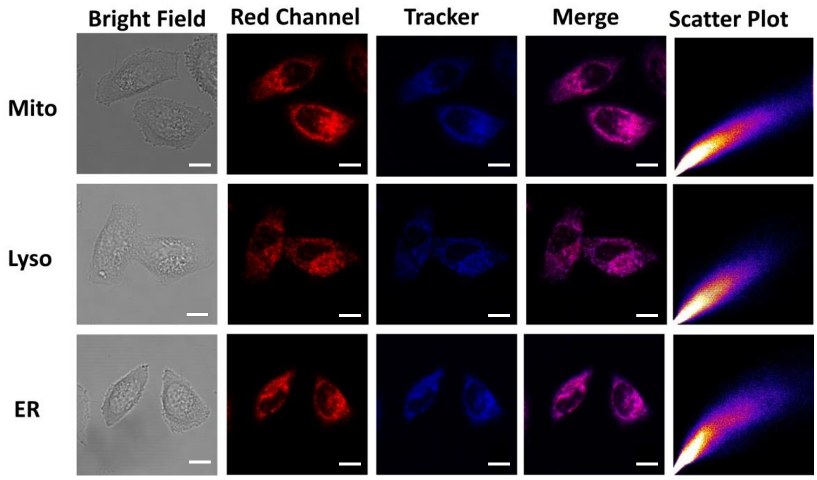

Supplementary Figure 108. Confocal fluorescence images for intracellular localization of **RD50** in HeLa cells. Cells were treated with 1 µM **RD50** for 0.5 h and stained (0.5 h) with 100 nM Mito-Tracker Green or 100 nM Lyso-Tracker DND-26 or 100 nM ER-Tracker Blue-White DPX. (Blue channel emission was collected in 410-480 nm upon excitation at 405 nm for ER-Tracker Blue-White DPX, pseudo blue, green channel emission was collected in 505-550 nm upon excitation at 488 nm for Mito-Tracker Green and Lyso-Tracker Green, pseudo blue, and red channel emission was collected in 565-680 nm upon excitation at 561 nm for **RD50**, pseudo red). Scale bar: 10 µm.

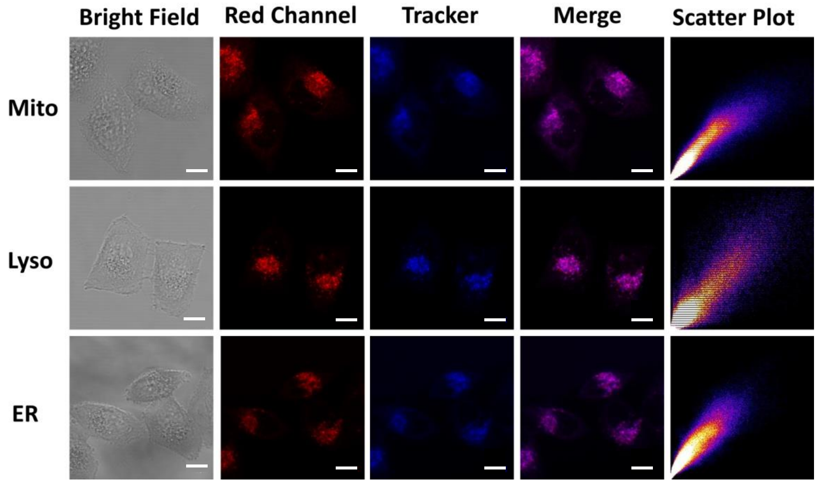

Supplementary Figure 109. Confocal fluorescence images for intracellular localization of **RD51** in HeLa cells. Cells were treated with 1 µM **RD51** for 0.5 h and stained (0.5 h) with 100 nM Mito-Tracker Green or 100 nM Lyso-Tracker DND-26 or 100 nM ER-Tracker Blue-White DPX. (Blue channel emission was collected in 410-480 nm upon excitation at 405 nm for ER-Tracker Blue-White DPX, pseudo blue, green channel emission was collected in 505-550 nm upon excitation at 488 nm for Mito-Tracker Green and Lyso-Tracker Green, pseudo blue, and red channel emission was collected in 565-680 nm upon excitation at 561 nm for **RD51**, pseudo red). Scale bar: 10 µm.

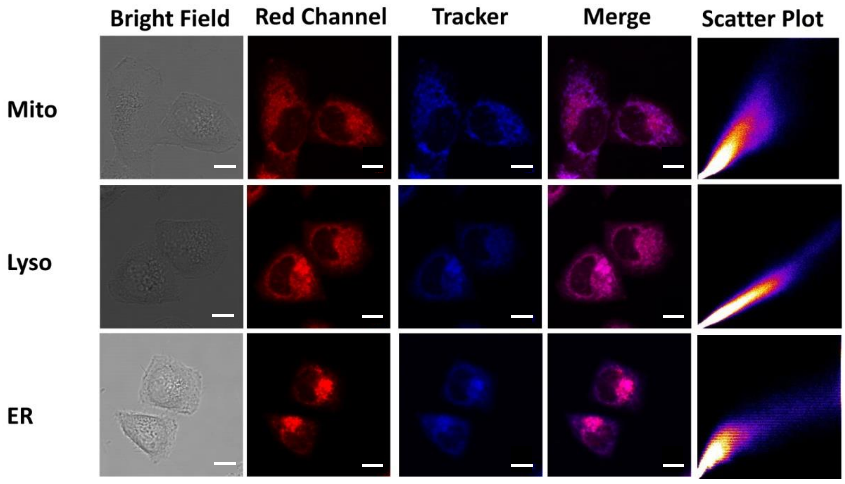

Supplementary Figure 110. Confocal fluorescence images for intracellular localization of **RD52** in HeLa cells. Cells were treated with 1 μM **RD52** for 0.5 h and stained (0.5 h) with 100 nM Mito-Tracker Green or 100 nM Lyso-Tracker DND-26 or 100 nM ER-Tracker Blue-White DPX. (Blue channel emission was collected in 410-480 nm upon excitation at 405 nm for ER-Tracker Blue-White DPX, pseudo blue, green channel emission was collected in 505-550 nm upon excitation at 488 nm for Mito-Tracker Green and Lyso-Tracker Green, pseudo blue, and red channel emission was collected in 565-680 nm upon excitation at 561 nm for **RD52**, pseudo red). Scale bar: 10 μm.

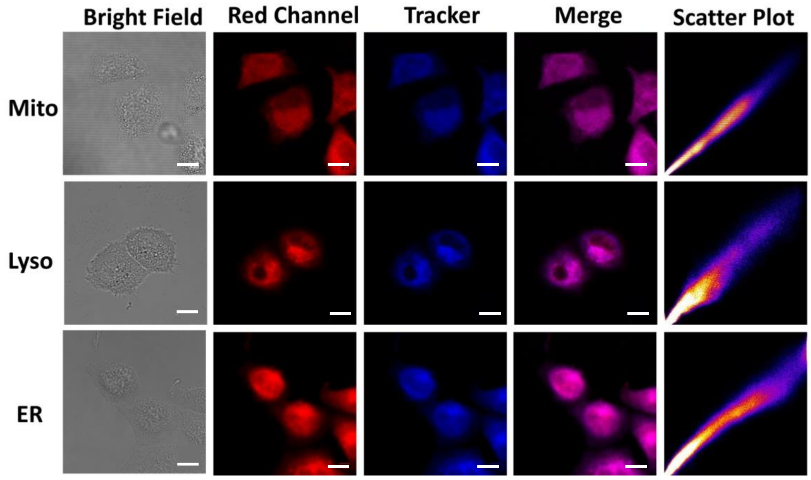

Supplementary Figure 111. Confocal fluorescence images for intracellular localization of **RD53** in HeLa cells. Cells were treated with 1 μM **RD53** for 0.5 h and stained (0.5 h) with 100 nM Mito-Tracker Green or 100 nM Lyso-Tracker DND-26 or 100 nM ER-Tracker Blue-White DPX. (Blue channel emission was collected in 410-480 nm upon excitation at 405 nm for ER-Tracker Blue-White DPX, pseudo blue, green channel emission was collected in 505-550 nm upon excitation at 488 nm for Mito-Tracker Green and Lyso-Tracker Green, pseudo blue, and red channel emission was collected in 565-680 nm upon excitation at 561 nm for **RD53**, pseudo red). Scale bar: 10 μm.

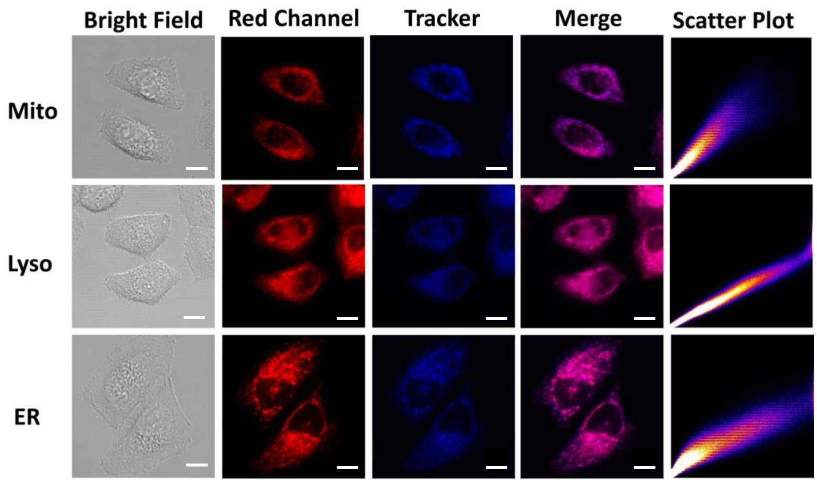

Supplementary Figure 112. Confocal fluorescence images for intracellular localization of **RD54** in HeLa cells. Cells were treated with 1 µM **RD54** for 0.5 h and stained (0.5 h) with 100 nM Mito-Tracker Green or 100 nM Lyso-Tracker DND-26 or 100 nM ER-Tracker Blue-White DPX. (Blue channel emission was collected in 410-480 nm upon excitation at 405 nm for ER-Tracker Blue-White DPX, pseudo blue, green channel emission was collected in 505-550 nm upon excitation at 488 nm for Mito-Tracker Green and Lyso-Tracker Green, pseudo blue, and red channel emission was collected in 565-680 nm upon excitation at 561 nm for **RD54**, pseudo red). Scale bar: 10 µm.

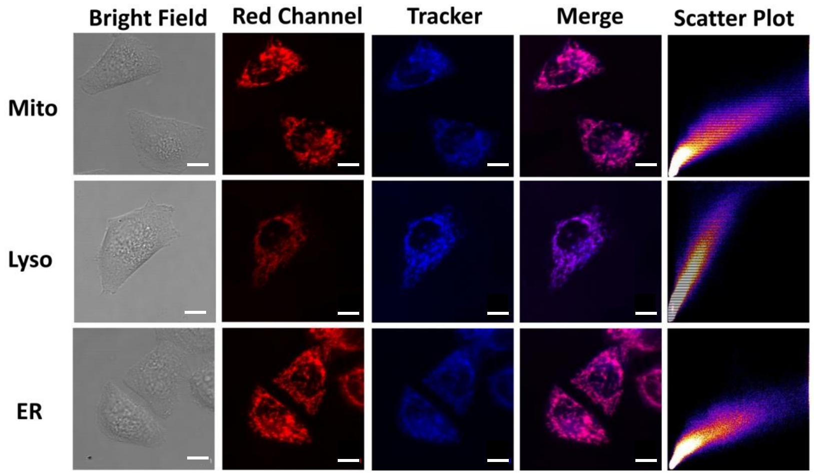

Supplementary Figure 113. Confocal fluorescence images for intracellular localization of **RD55** in HeLa cells. Cells were treated with 1 µM **RD55** for 0.5 h and stained (0.5 h) with 100 nM Mito-Tracker Green or 100 nM Lyso-Tracker DND-26 or 100 nM ER-Tracker Blue-White DPX. (Blue channel emission was collected in 410-480 nm upon excitation at 405 nm for ER-Tracker Blue-White DPX, pseudo blue, green channel emission was collected in 505-550 nm upon excitation at 488 nm for Mito-Tracker Green and Lyso-Tracker Green, pseudo blue, and red channel emission was collected in 565-680 nm upon excitation at 561 nm for **RD55**, pseudo red). Scale bar: 10 µm.

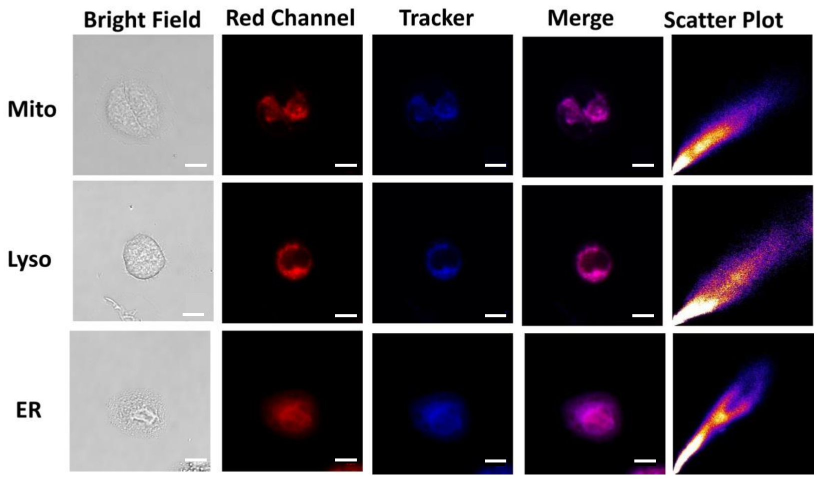

Supplementary Figure 114. Confocal fluorescence images for intracellular localization of **RD56** in A549 cells. Cells were treated with 1 μM **RD56** for 0.5 h and stained (0.5 h) with 100 nM Mito-Tracker Green or 100 nM Lyso-Tracker DND-26 or 100 nM ER-Tracker Blue-White DPX. (Blue channel emission was collected in 410-480 nm upon excitation at 405 nm for ER-Tracker Blue-White DPX, pseudo blue, green channel emission was collected in 505-550 nm upon excitation at 488 nm for Mito-Tracker Green and Lyso-Tracker Green, pseudo blue, and red channel emission was collected in 565-680 nm upon excitation at 561 nm for **RD56**, pseudo red). Scale bar: 10 μm.

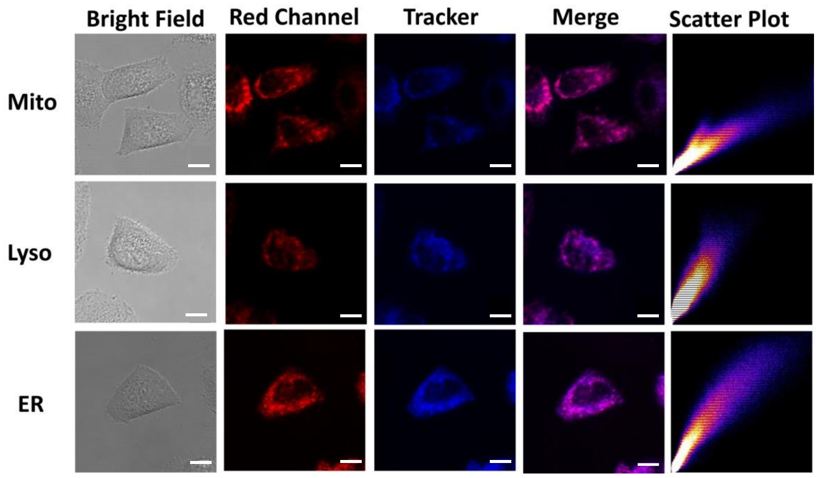

Supplementary Figure 115. Confocal fluorescence images for intracellular localization of **RD57** in HeLa cells. Cells were treated with 1 μM **RD57** for 0.5 h and stained (0.5 h) with 100 nM Mito-Tracker Green or 100 nM Lyso-Tracker DND-26 or 100 nM ER-Tracker Blue-White DPX. (Blue channel emission was collected in 410-480 nm upon excitation at 405 nm for ER-Tracker Blue-White DPX, pseudo blue, green channel emission was collected in 505-550 nm upon excitation at 488 nm for Mito-Tracker Green and Lyso-Tracker Green, pseudo blue, and red channel emission was collected in 565-680 nm upon excitation at 561 nm for **RD57**, pseudo red). Scale bar: 10 μm.

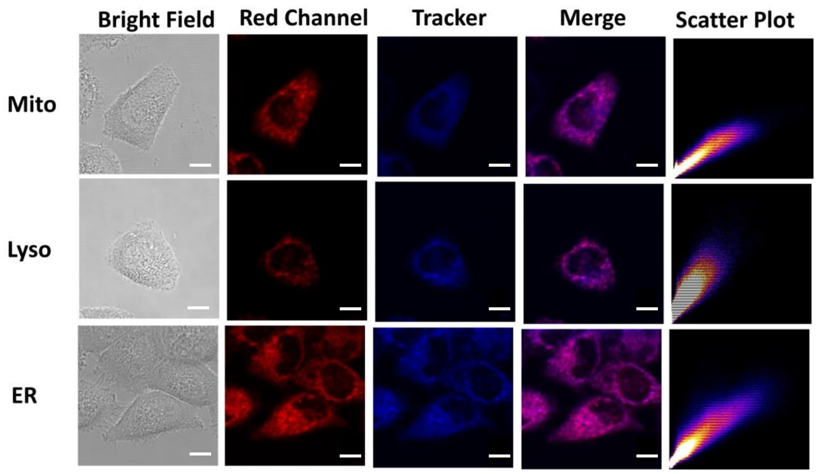

Supplementary Figure 116. Confocal fluorescence images for intracellular localization of **RD58** in HeLa cells. Cells were treated with 1 μM **RD58** for 0.5 h and stained (0.5 h) with 100 nM Mito-Tracker Green or 100 nM Lyso-Tracker DND-26 or 100 nM ER-Tracker Blue-White DPX. (Blue channel emission was collected in 410-480 nm upon excitation at 405 nm for ER-Tracker Blue-White DPX, pseudo blue, green channel emission was collected in 505-550 nm upon excitation at 488 nm for Mito-Tracker Green and Lyso-Tracker Green, pseudo blue, and red channel emission was collected in 565-680 nm upon excitation at 561 nm for **RD58**, pseudo red). Scale bar: 10 μm.

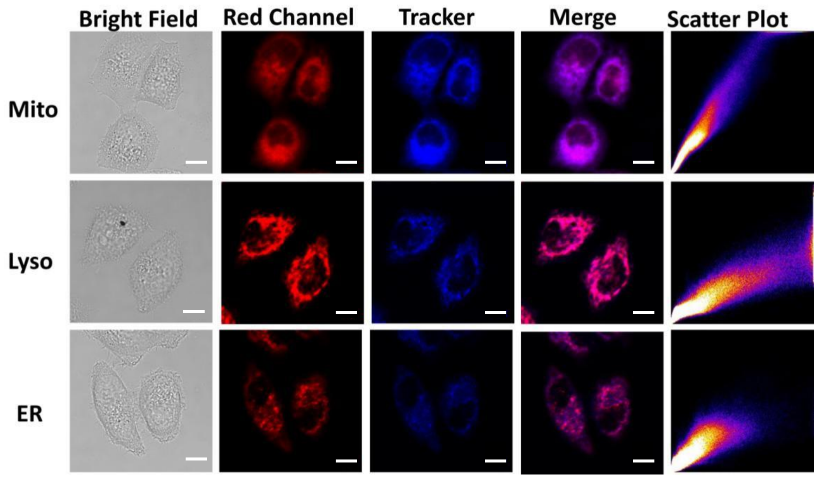

Supplementary Figure 117. Confocal fluorescence images for intracellular localization of **RD59** in HeLa cells. Cells were treated with 1 μM **RD59** for 0.5 h and stained (0.5 h) with 100 nM Mito-Tracker Green or 100 nM Lyso-Tracker DND-26 or 100 nM ER-Tracker Blue-White DPX. (Blue channel emission was collected in 410-480 nm upon excitation at 405 nm for ER-Tracker Blue-White DPX, pseudo blue, green channel emission was collected in 505-550 nm upon excitation at 488 nm for Mito-Tracker Green and Lyso-Tracker Green, pseudo blue, and red channel emission was collected in 565-680 nm upon excitation at 561 nm for **RD59**, pseudo red). Scale bar: 10 μm.

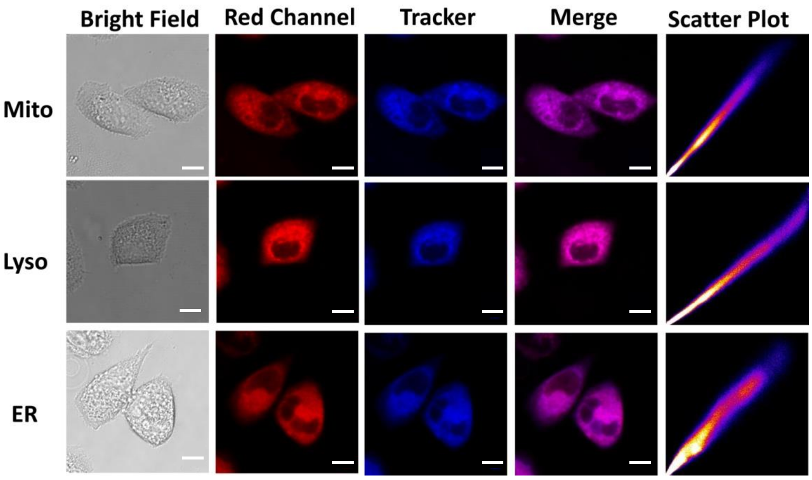

Supplementary Figure 118. Confocal fluorescence images for intracellular localization of **RD60** in HeLa cells. Cells were treated with 1 μM **RD60** for 0.5 h and stained (0.5 h) with 100 nM Mito-Tracker Green or 100 nM Lyso-Tracker DND-26 or 100 nM ER-Tracker Blue-White DPX. (Blue channel emission was collected in 410-480 nm upon excitation at 405 nm for ER-Tracker Blue-White DPX, pseudo blue, green channel emission was collected in 505-550 nm upon excitation at 488 nm for Mito-Tracker Green and Lyso-Tracker Green, pseudo blue, and red channel emission was collected in 565-680 nm upon excitation at 561 nm for **RD60**, pseudo red). Scale bar: 10 μm.

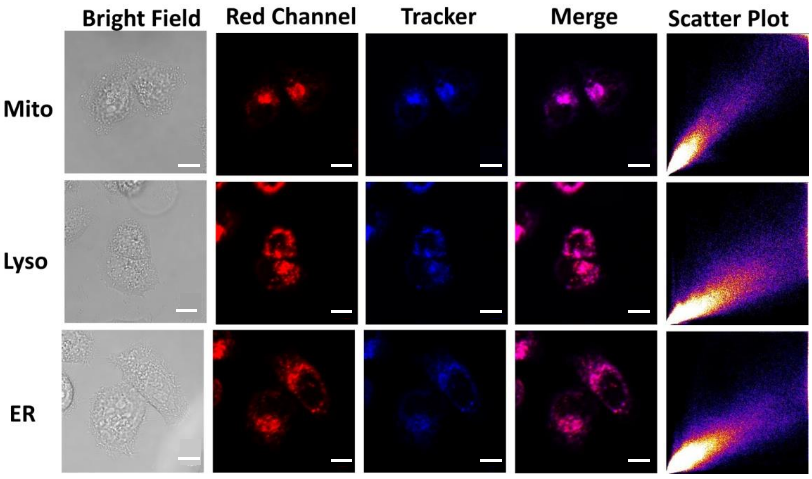

Supplementary Figure 119. Confocal fluorescence images for intracellular localization of **RD61** in HeLa cells. Cells were treated with 1 μM **RD61** for 0.5 h and stained (0.5 h) with 100 nM Mito-Tracker Green or 100 nM Lyso-Tracker DND-26 or 100 nM ER-Tracker Blue-White DPX. (Blue channel emission was collected in 410-480 nm upon excitation at 405 nm for ER-Tracker Blue-White DPX, pseudo blue, green channel emission was collected in 505-550 nm upon excitation at 488 nm for Mito-Tracker Green and Lyso-Tracker Green, pseudo blue, and red channel emission was collected in 565-680 nm upon excitation at 561 nm for **RD61**, pseudo red). Scale bar: 10 μm.

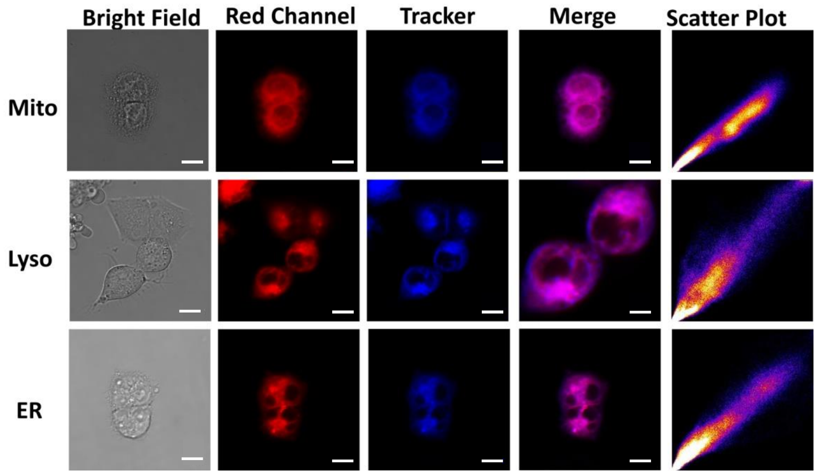

Supplementary Figure 120. Confocal fluorescence images for intracellular localization of **RD62** in A549 cells. Cells were treated with 1 μM **RD62** for 0.5 h and stained (0.5 h) with 100 nM Mito-Tracker Green or 100 nM Lyso-Tracker DND-26 or 100 nM ER-Tracker Blue-White DPX. (Blue channel emission was collected in 410-480 nm upon excitation at 405 nm for ER-Tracker Blue-White DPX, pseudo blue, green channel emission was collected in 505-550 nm upon excitation at 488 nm for Mito-Tracker Green and Lyso-Tracker Green, pseudo blue, and red channel emission was collected in 565-680 nm upon excitation at 561 nm for **RD62**, pseudo red). Scale bar: 10 μm.

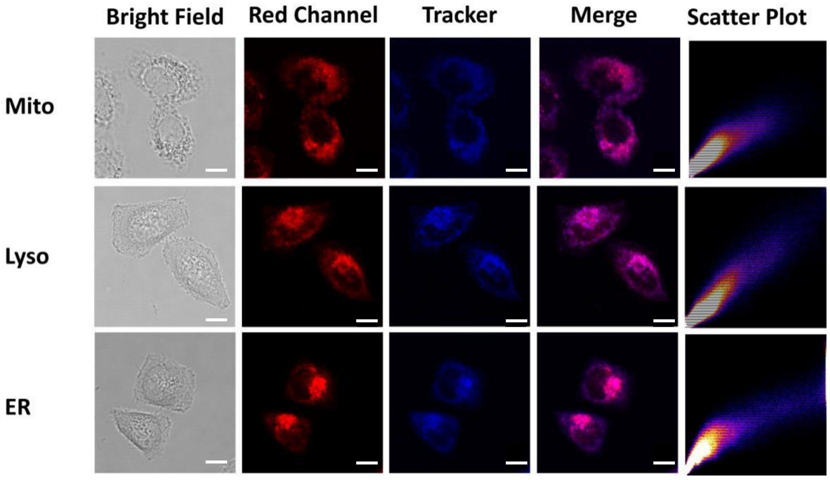

Supplementary Figure 121. Confocal fluorescence images for intracellular localization of **RD63** in A549 cells. Cells were treated with 1 μM **RD63** for 0.5 h and stained (0.5 h) with 100 nM Mito-Tracker Green or 100 nM Lyso-Tracker DND-26 or 100 nM ER-Tracker Blue-White DPX. (Blue channel emission was collected in 410-480 nm upon excitation at 405 nm for ER-Tracker Blue-White DPX, pseudo blue, green channel emission was collected in 505-550 nm upon excitation at 488 nm for Mito-Tracker Green and Lyso-Tracker Green, pseudo blue, and red channel emission was collected in 565-680 nm upon excitation at 561 nm for **RD63**, pseudo red). Scale bar: 10 μm.

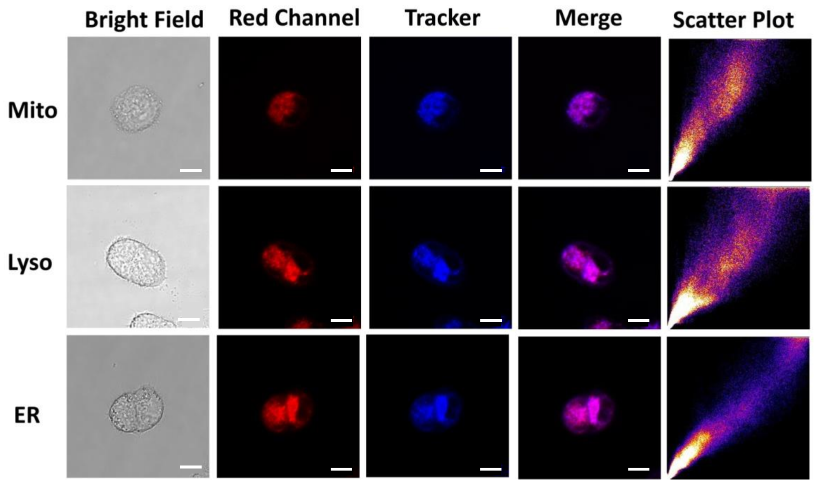

Supplementary Figure 122. Confocal fluorescence images for intracellular localization of **RD64** in A549 cells. Cells were treated with 1 μM **RD64** for 0.5 h and stained (0.5 h) with 100 nM Mito-Tracker Green or 100 nM Lyso-Tracker DND-26 or 100 nM ER-Tracker Blue-White DPX. (Blue channel emission was collected in 410-480 nm upon excitation at 405 nm for ER-Tracker Blue-White DPX, pseudo blue, green channel emission was collected in 505-550 nm upon excitation at 488 nm for Mito-Tracker Green and Lyso-Tracker Green, pseudo blue, and red channel emission was collected in 565-680 nm upon excitation at 561 nm for **RD64**, pseudo red). Scale bar: 10 μm.

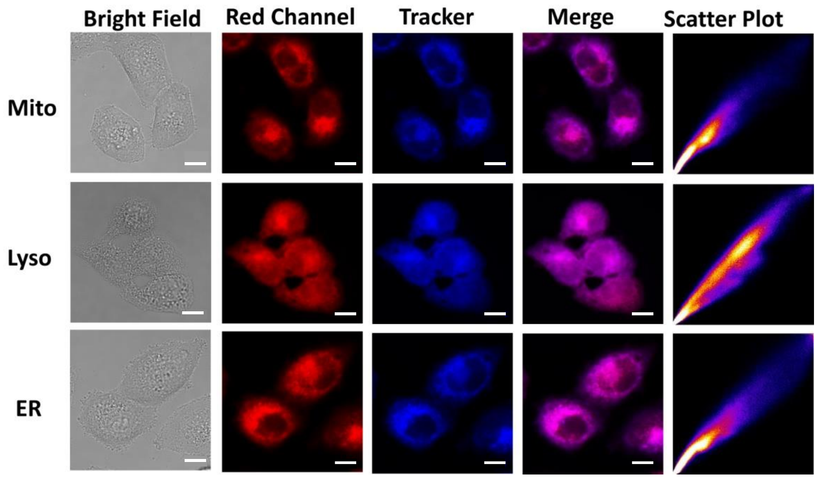

Supplementary Figure 123. Confocal fluorescence images for intracellular localization of **RD65** in HeLa cells. Cells were treated with 1 μM **RD65** for 0.5 h and stained (0.5 h) with 100 nM Mito-Tracker Green or 100 nM Lyso-Tracker DND-26 or 100 nM ER-Tracker Blue-White DPX. (Blue channel emission was collected in 410-480 nm upon excitation at 405 nm for ER-Tracker Blue-White DPX, pseudo blue, green channel emission was collected in 505-550 nm upon excitation at 488 nm for Mito-Tracker Green and Lyso-Tracker Green, pseudo blue, and red channel emission was collected in 565-680 nm upon excitation at 561 nm for **RD65**, pseudo red). Scale bar: 10 μm.

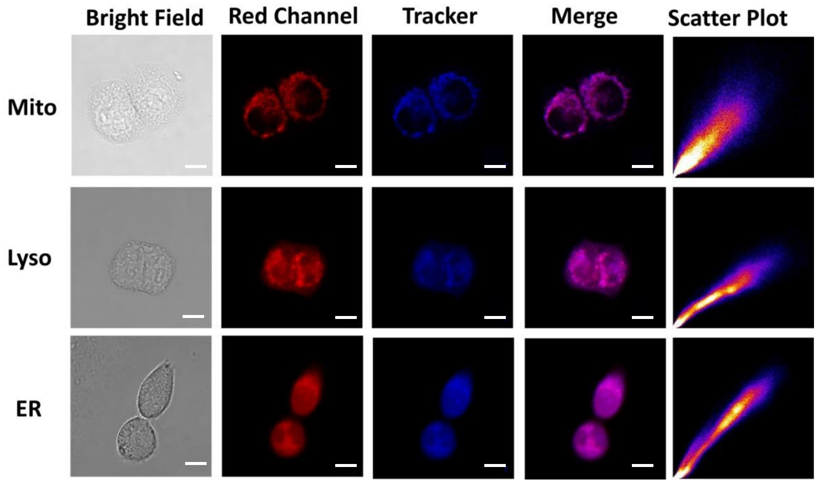

Supplementary Figure 124. Confocal fluorescence images for intracellular localization of **RD66** in A549 cells. Cells were treated with 1 μM **RD66** for 0.5 h and stained (0.5 h) with 100 nM Mito-Tracker Green or 100 nM Lyso-Tracker DND-26 or 100 nM ER-Tracker Blue-White DPX. (Blue channel emission was collected in 410-480 nm upon excitation at 405 nm for ER-Tracker Blue-White DPX, pseudo blue, green channel emission was collected in 505-550 nm upon excitation at 488 nm for Mito-Tracker Green and Lyso-Tracker Green, pseudo blue, and red channel emission was collected in 565-680 nm upon excitation at 561 nm for **RD66**, pseudo red). Scale bar: 10 μm.

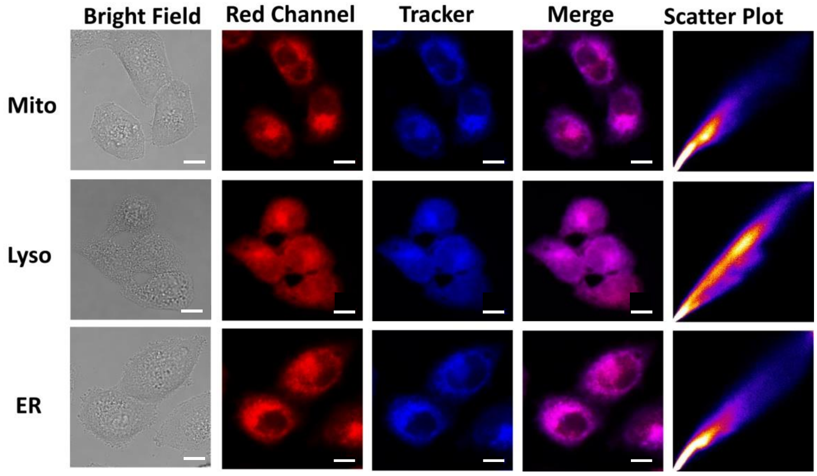

Supplementary Figure 125. Confocal fluorescence images for intracellular localization of **RD67** in HeLa cells. Cells were treated with 1 μM **RD67** for 0.5 h and stained (0.5 h) with 100 nM Mito-Tracker Green or 100 nM Lyso-Tracker DND-26 or 100 nM ER-Tracker Blue-White DPX. (Blue channel emission was collected in 410-480 nm upon excitation at 405 nm for ER-Tracker Blue-White DPX, pseudo blue, green channel emission was collected in 505-550 nm upon excitation at 488 nm for Mito-Tracker Green and Lyso-Tracker Green, pseudo blue, and red channel emission was collected in 565-680 nm upon excitation at 561 nm for **RD67**, pseudo red). Scale bar: 10 μm.

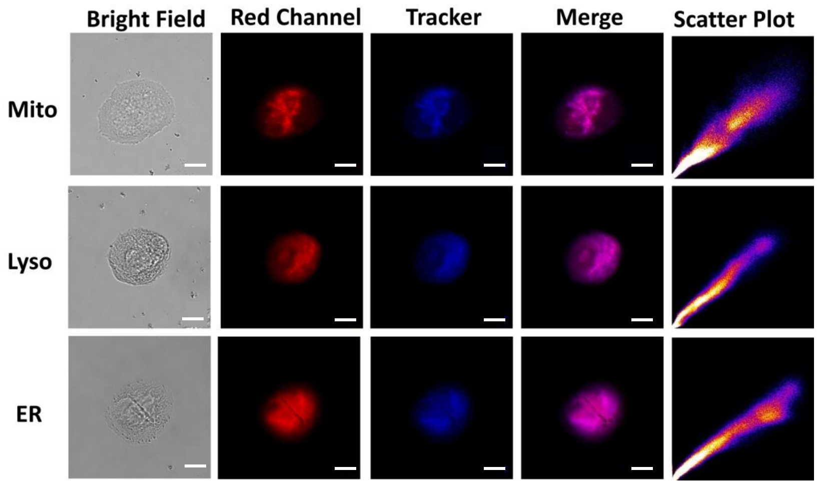

Supplementary Figure 126. Confocal fluorescence images for intracellular localization of **RD68** in A549 cells. Cells were treated with 1 μM **RD68** for 0.5 h and stained (0.5 h) with 100 nM Mito-Tracker Green or 100 nM Lyso-Tracker DND-26 or 100 nM ER-Tracker Blue-White DPX. (Blue channel emission was collected in 410-480 nm upon excitation at 405 nm for ER-Tracker Blue-White DPX, pseudo blue, green channel emission was collected in 505-550 nm upon excitation at 488 nm for Mito-Tracker Green and Lyso-Tracker Green, pseudo blue, and red channel emission was collected in 565-680 nm upon excitation at 561 nm for **RD68**, pseudo red). Scale bar: 10 μm.

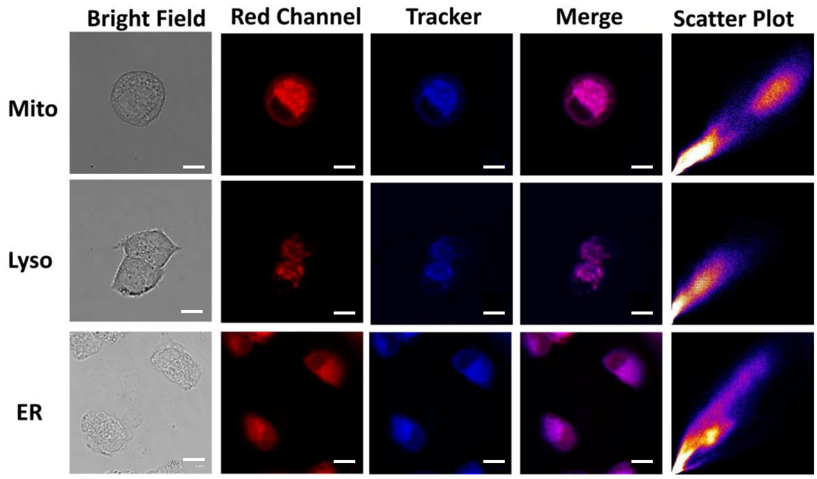

Supplementary Figure 127. Confocal fluorescence images for intracellular localization of **RD69** in A549 cells. Cells were treated with 1 μM **RD69** for 0.5 h and stained (0.5 h) with 100 nM Mito-Tracker Green or 100 nM Lyso-Tracker DND-26 or 100 nM ER-Tracker Blue-White DPX. (Blue channel emission was collected in 410-480 nm upon excitation at 405 nm for ER-Tracker Blue-White DPX, pseudo blue, green channel emission was collected in 505-550 nm upon excitation at 488 nm for Mito-Tracker Green and Lyso-Tracker Green, pseudo blue, and red channel emission was collected in 565-680 nm upon excitation at 561 nm for **RD69**, pseudo red). Scale bar: 10 μm.

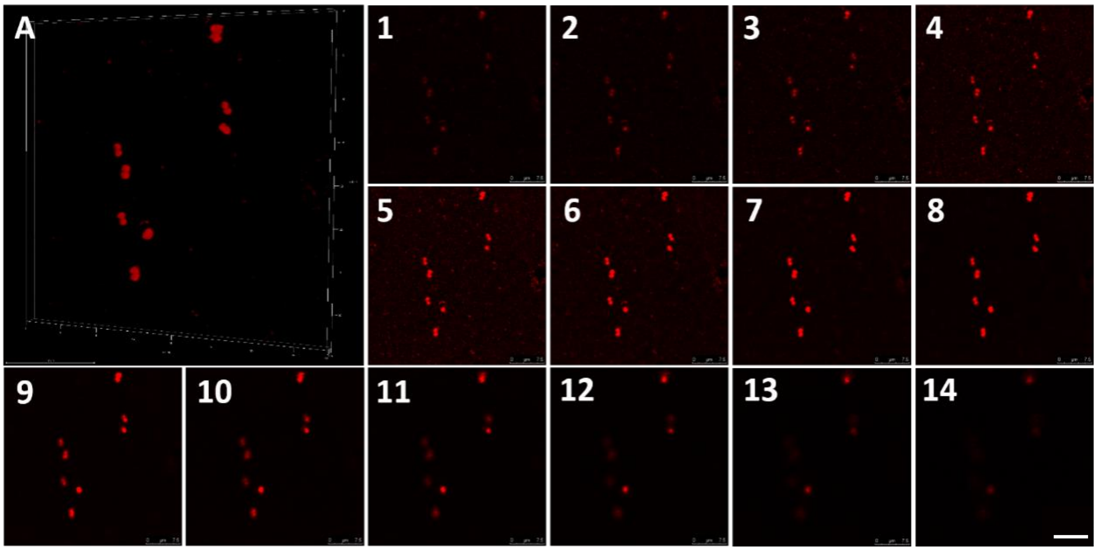

Supplementary Figure 128. Fluorescence image of MRSA stained with 8 μg mL^{-1} **RD53** for 3 min. (**A**) The 3D confocal fluorescence image. (**1-14**) Montage of a sub-set of acquired z-planes from the top to the bottom. Note: confocal images were collected with λ_{ex} = 561 nm and λ_{em} = 590-640 nm. Scale bars represent 7.5 μm.

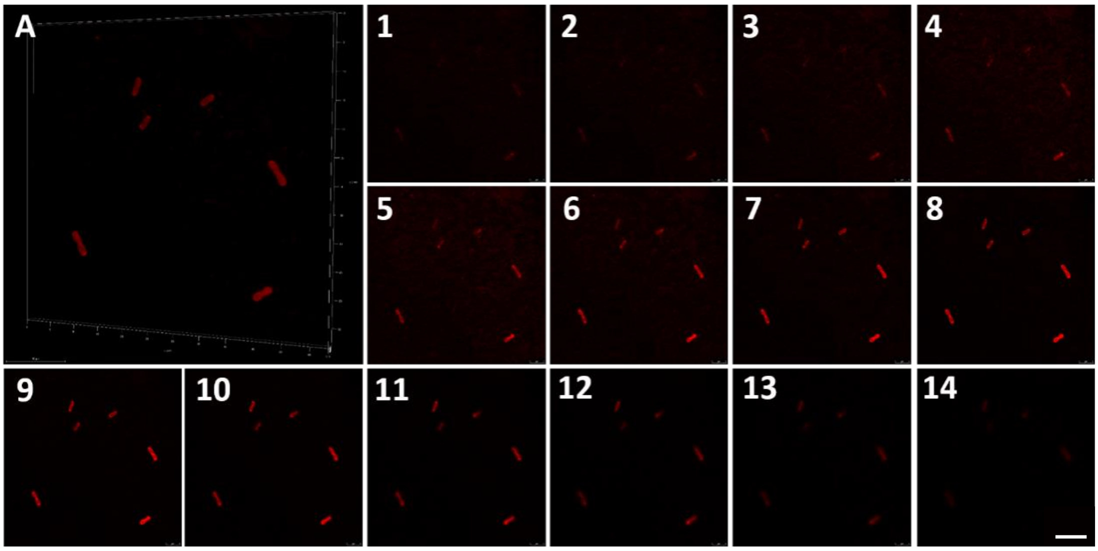

Supplementary Figure 129. Fluorescence image of *Acinetobacter baumannii* stained with 2 μg mL^{-1} **RD53** for 3 min. (**A**) The 3D confocal fluorescence image. (**1-14**) Montage of a sub-set of acquired z-planes from the top to the bottom. Note: confocal images were collected with λ_{ex} = 561 nm and λ_{em} = 590-640 nm. Scale bars represent 10 μm.

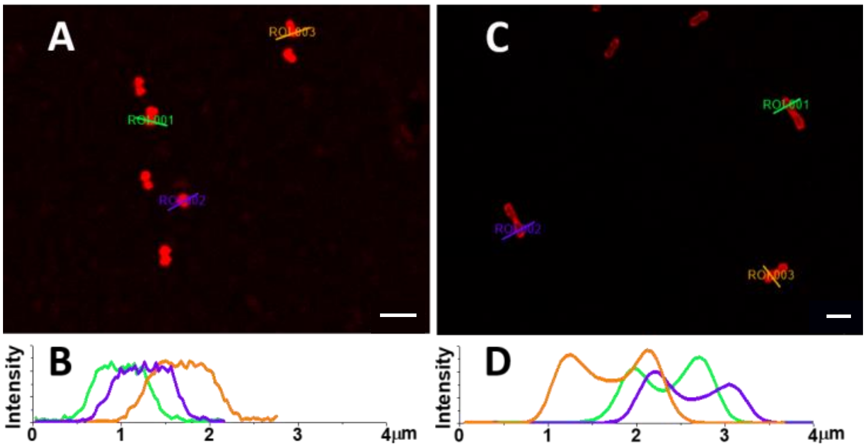

Supplementary Figure 130. Bacterial imaging with **RD53**. The confocal images of (A) MRSA (ATCC43300) and (C) *A. baumannii* (ATCC19606), stained with 2 μg mL^{-1} of **RD53** for 3 min. The line intensities of MRSA (B) and *A. baumannii* (D) were plotted. Note: confocal images were collected with λ_{ex} = 561 nm and λ_{em} = 590-640 nm. Scale bars represent 3 μm. Cells of both MRSA and *A. baumannii* were readily stained and brightly fluorescent. The fluorescence intensity of **RD53** in MRSA was essentially equally high from cell wall to cell wall, suggesting a high uptake of **RD53** into the intracellular region. In contrast, the fluorescence of **RD53** was much lower in the intracellular region of *A. baumannii* than the cell wall/membrane region.

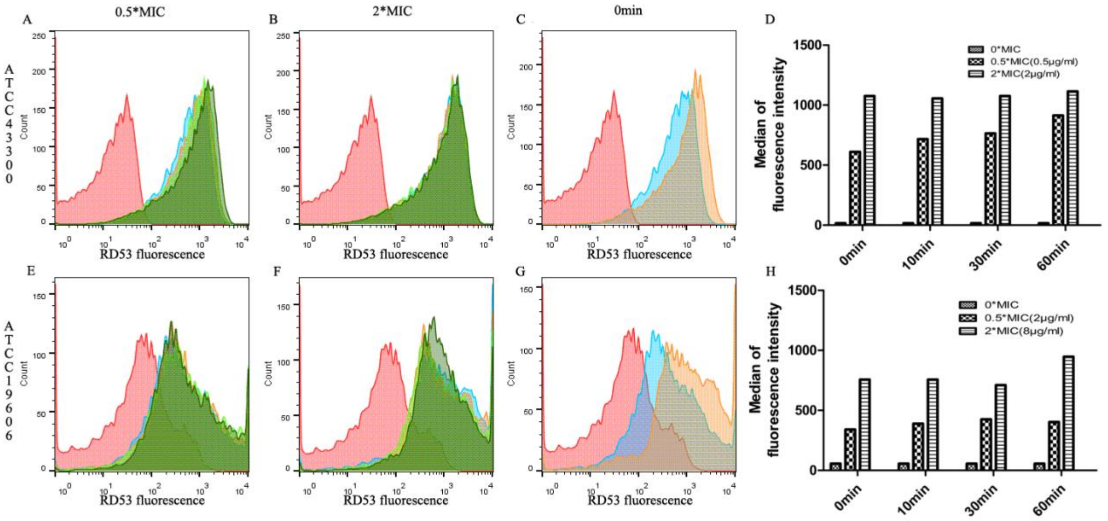

Supplementary Figure 131. Flow cytometry of ATCC43300 and ATCC19606 staining with **RD53**. After incubating a 1 mL bacterial suspension at 10^8 cells with 0.5*MIC and 2*MIC **RD53** for 0 min, 10 min, 30 min, 60 min at 37 ºC with aeration, the resultant product was washed three times by centrifugation at 5510 g for 3 min to remove all the unbound **RD53**. The bacteria were then resuspended in PBS and assayed for red fluorescence by a BD FACS Calibur FCM. (A,B,E,F) the red peaks: without **RD53**; the blue peaks: **RD53** for 0 min; the orange peaks: **RD53** for 10min; the light green peaks: **RD53** for 30 min; the blackish green peaks: **RD53** for 60 min. (C)(G) the red peaks: without **RD53**; the blue peaks represent bacterial with 0.5*MIC **RD53** , the orange peaks represent bacterial with 2*MIC **RD53**.

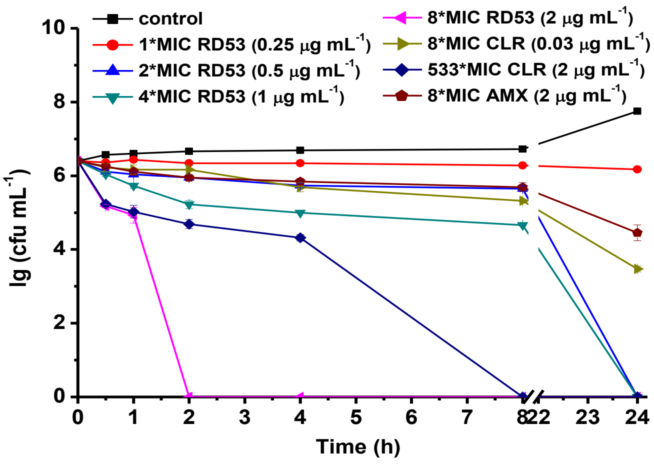

Supplementary Figure 132. Time-dependent killing of *H. pylori* by **RD53.** Data are representative of 3 independent experiments. Error bars indicate s.d.

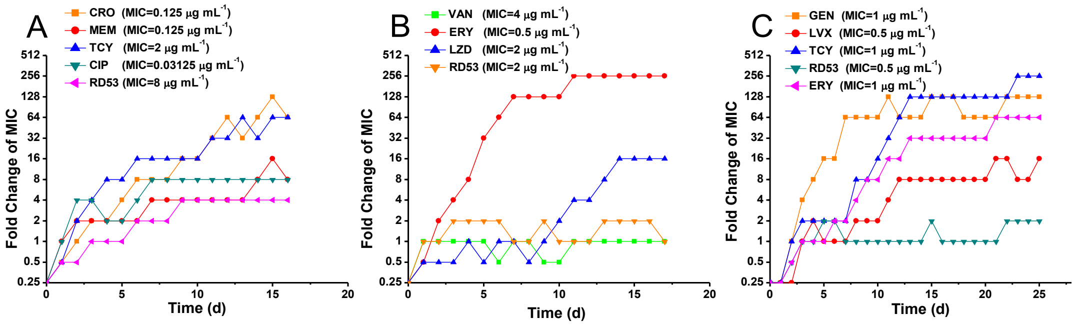

Supplementary Figure 133. Resistance acquisition of pathogens during serial passaging in the presence of sub-MIC levels of antimicrobials. The y axis is the fold change of MIC. A) *E.coli* (ATCC25922) towards **RD53** (MIC = 8 μg mL^{-1}, tested up to 4×MIC), Ceftriaxone (MIC = 0.125 μg mL^{-1}, tested up to 128×MIC), Meropenem (MIC = 0.125 μg mL^{-1}, tested up to 16×MIC), Tetracycline (MIC =2 μg mL^{-1}, tested up to 64×MIC), and Ciprofloxacin (MIC = 0.03125 μg mL^{-1}, tested up to 8×MIC). B) *E.faecalis* (ATCC29212) toward **RD53** (MIC = 2 μg mL^{-1}, tested up to 2×MIC), Linezolid (MIC = 2 μg mL^{-1}, tested up to 16 MIC), Erythromycin (MIC = 0.5 μg mL^{-1}, tested up to 256×MIC), and Vancomycin (MIC = 4 μg mL^{-1}, tested up to 1×MIC). C) *S.aureus* (ATCC25923) toward **RD53** (MIC = 0.5 μg mL^{-1}, tested up to 2×MIC), Gentamicin (MIC = 1 μg mL^{-1}, tested up to 128×MIC), Levofloxacin (MIC = 0.5 μg mL^{-1}, tested up to 16×MIC), Tetracycline (MIC = 1 μg mL^{-1}, tested up to 256×MIC), and Erythromycin (MIC = 1 μg mL^{-1}, tested up to 64×MIC)

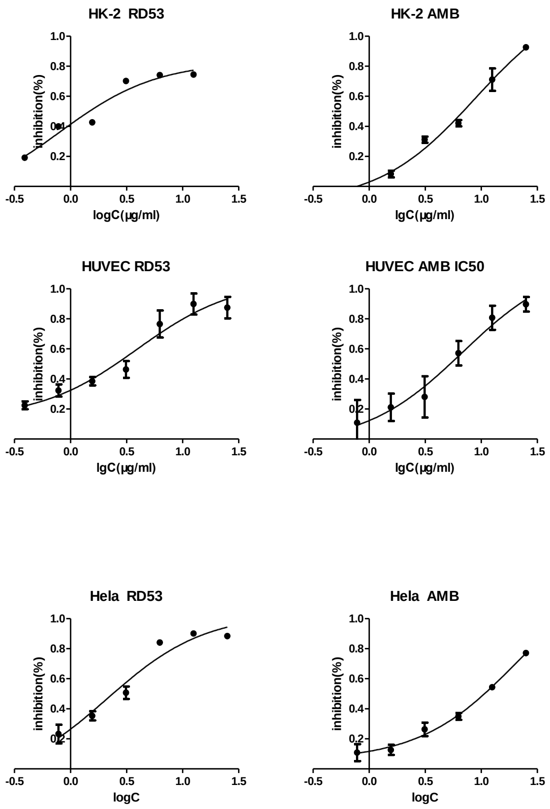

Supplementary Figure 134. Cytotoxicity study of **RD53** on mammal cells in comparison with amphotericin B. Tested cells are human kidney 2 cells (HK-2), human umbilical vein endothelial cells (HUVEC) and Hela cells. **RD53**: CC_{50} = 0.804 μg mL^{-1} for HK-2, CC_{50} = 3.912 μg mL^{-1} for HUVEC, CC_{50} = 2.122 μg mL^{-1} for Hela Cells; amphotericin B: CC_{50} = 8.870 μg mL^{-1} for HK-2, CC_{50} = 6.846 μg mL^{-1} for HUVEC, CC_{50} = 20.74 μg mL^{-1}. Data are representative of 3 independent experiments. Error bars indicate s.d.

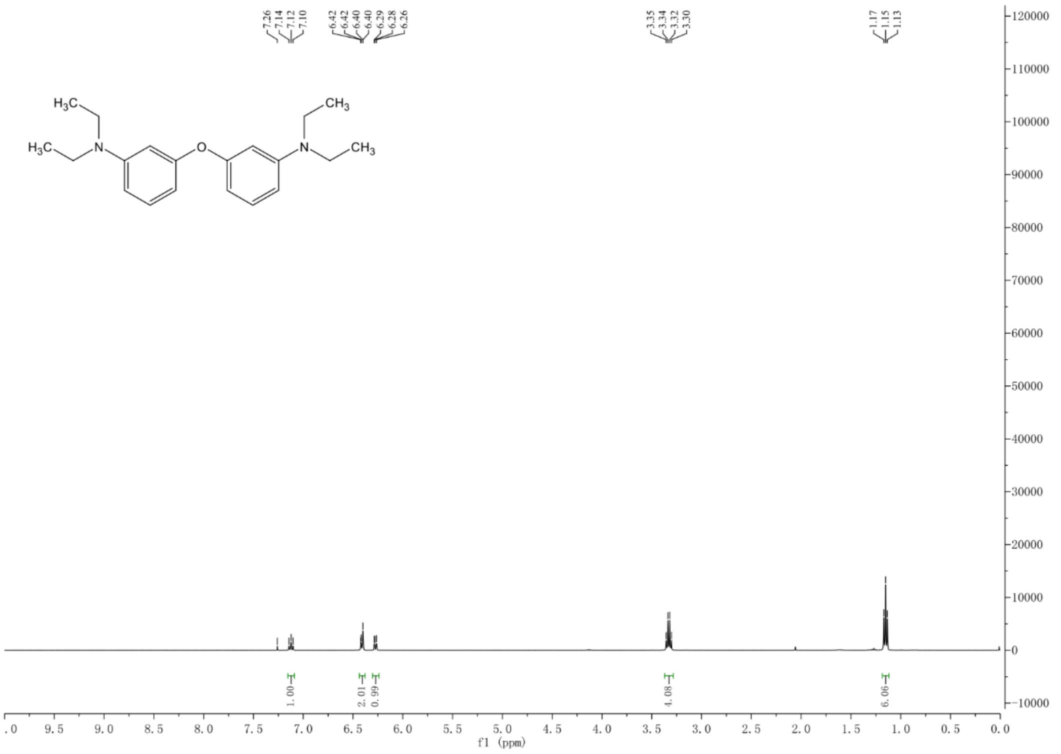

Supplementary Figure 135. The ^{1}H-NMR of compound **1a** in $CDCl_3$.

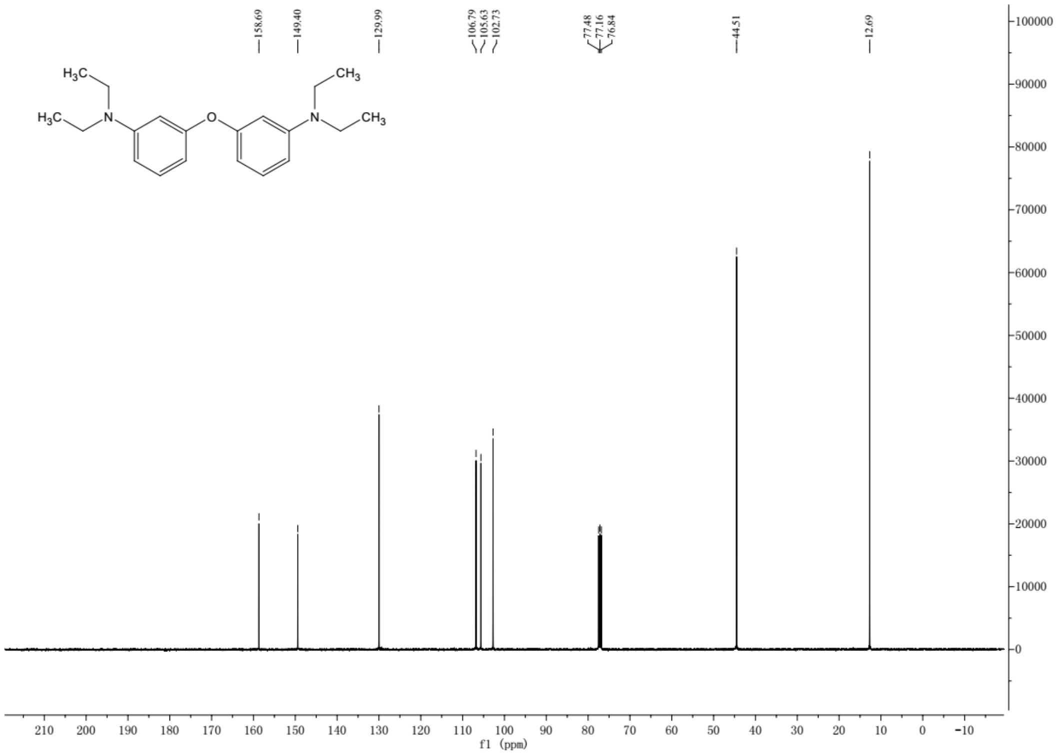

Supplementary Figure 136. The ^{13}C-NMR of compound **1a** in $CDCl_3$

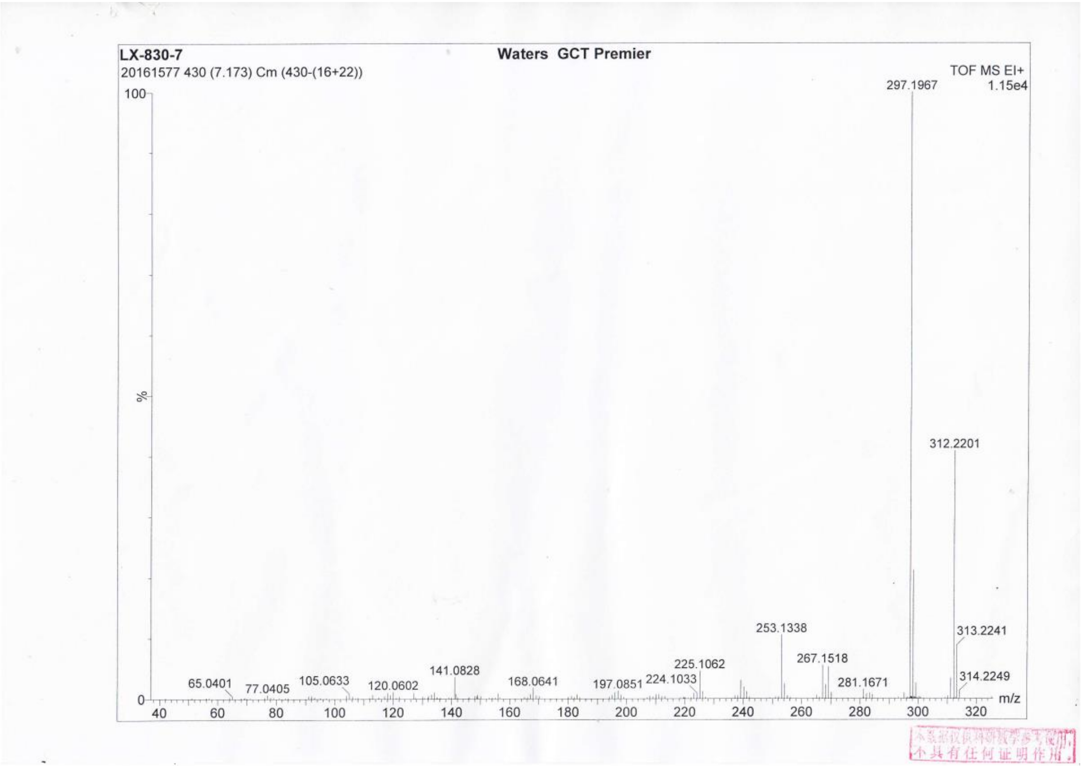

Supplementary Figure 137. The HR-MS of compound **1a**

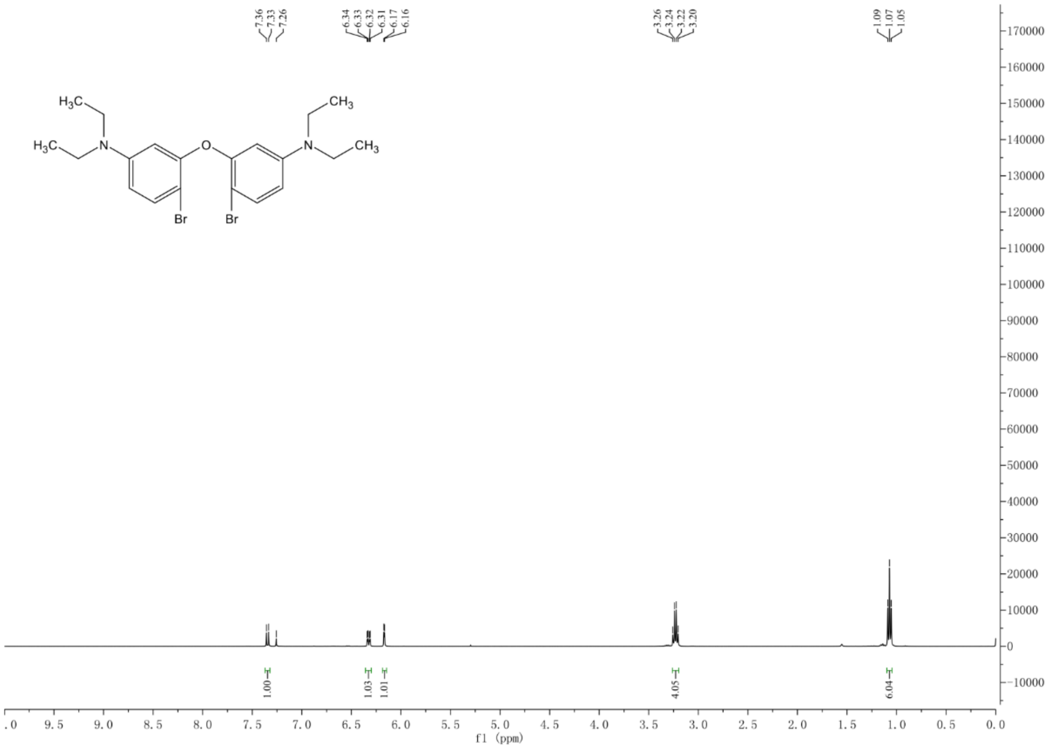

Supplementary Figure 138. The ^{1}H-NMR of compound **1b** in $CDCl_3$.

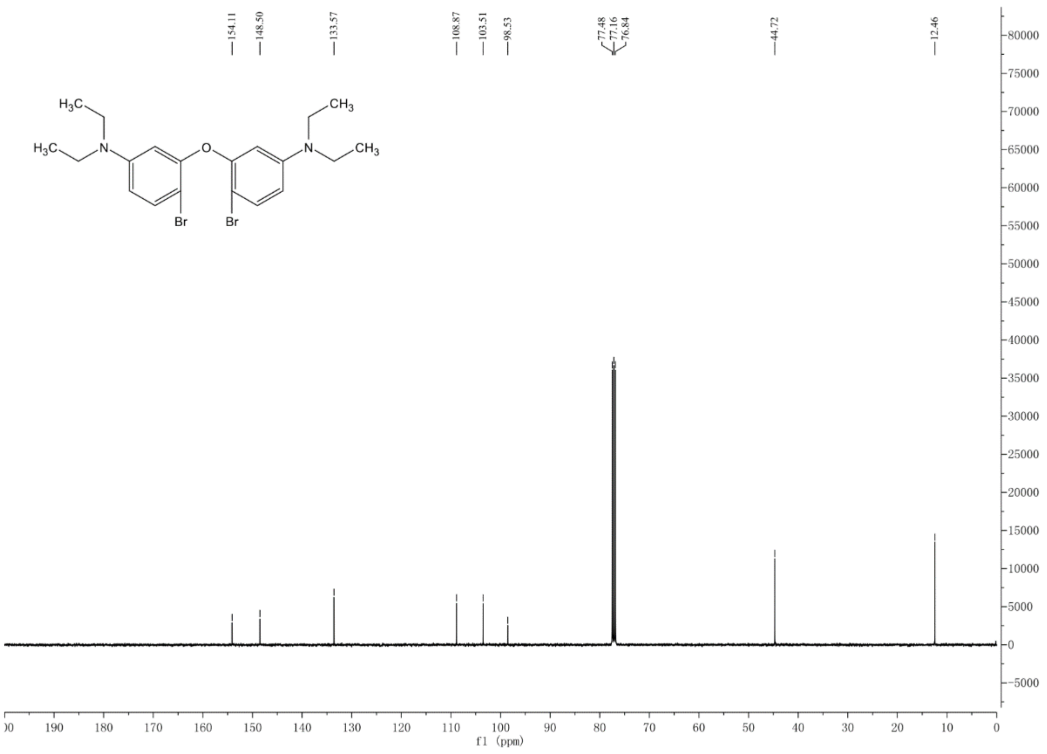

Supplementary Figure 139. The ^{13}C-NMR of compound **1b** in $CDCl_3$

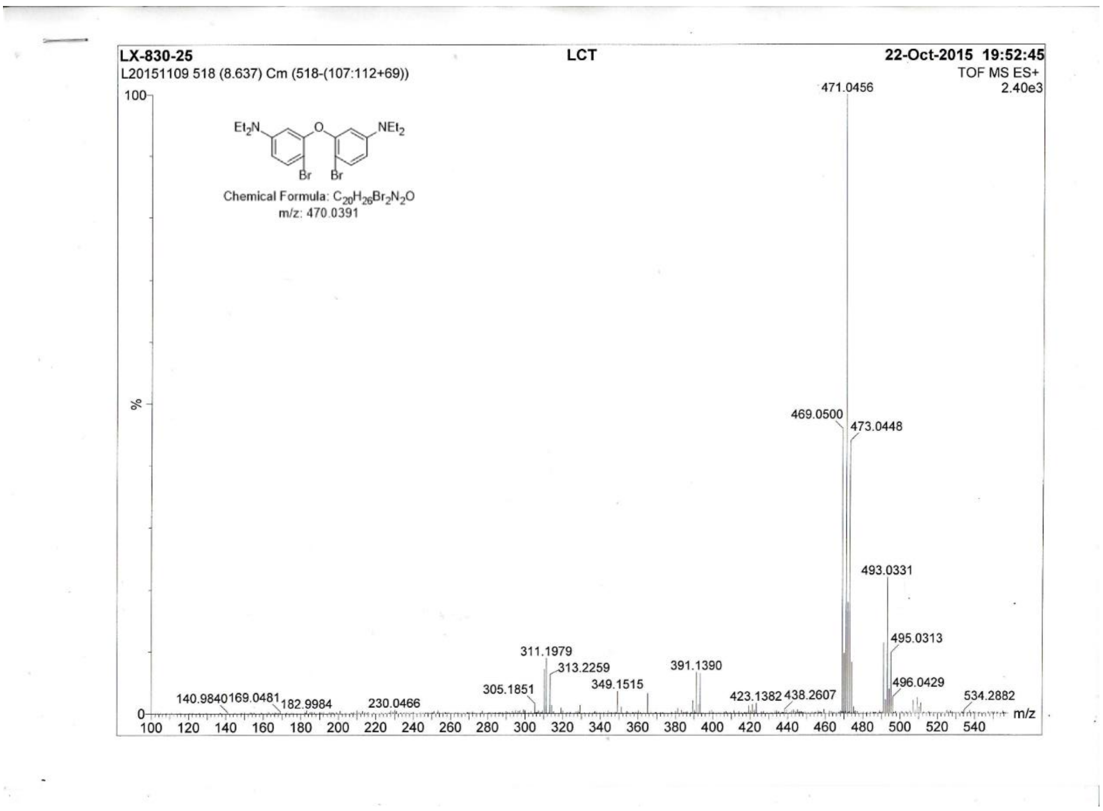

Supplementary Figure 140. The HR-MS of compound **1b**

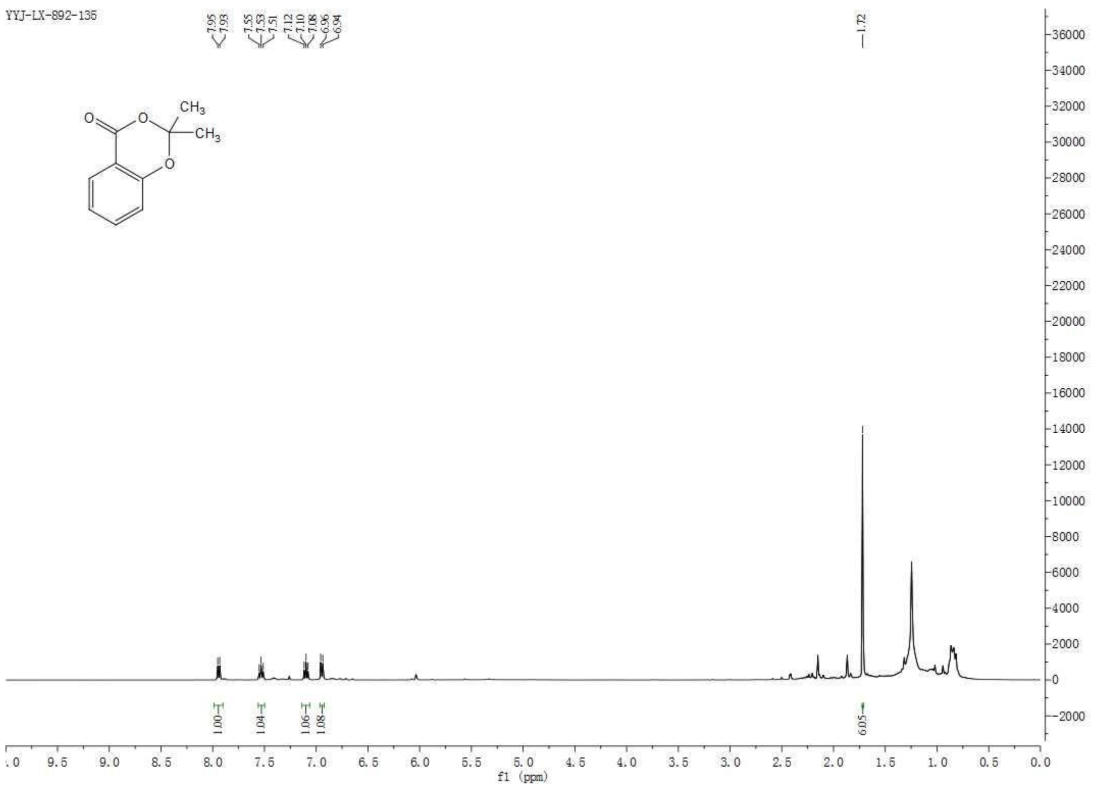

Supplementary Figure 141. The ^{1}H-NMR of compound **S10** in $CDCl_3$

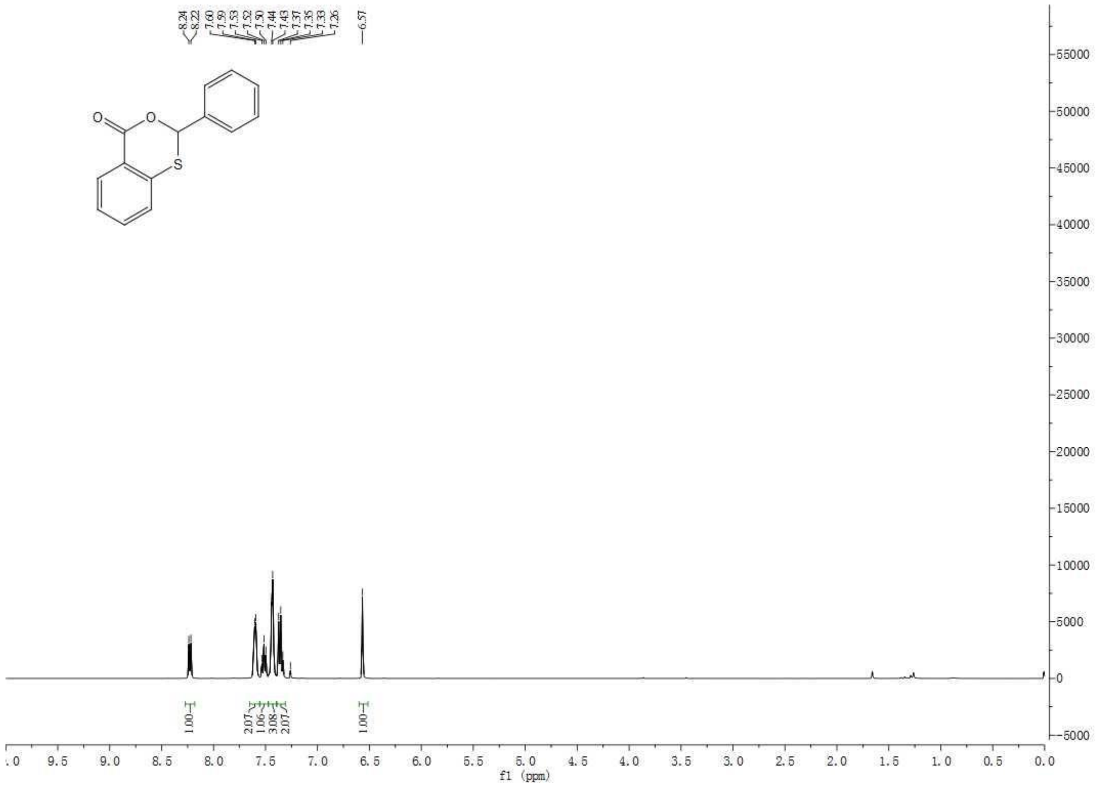

Supplementary Figure 142. The ^{1}H-NMR of compound **S11** in $CDCl_3$

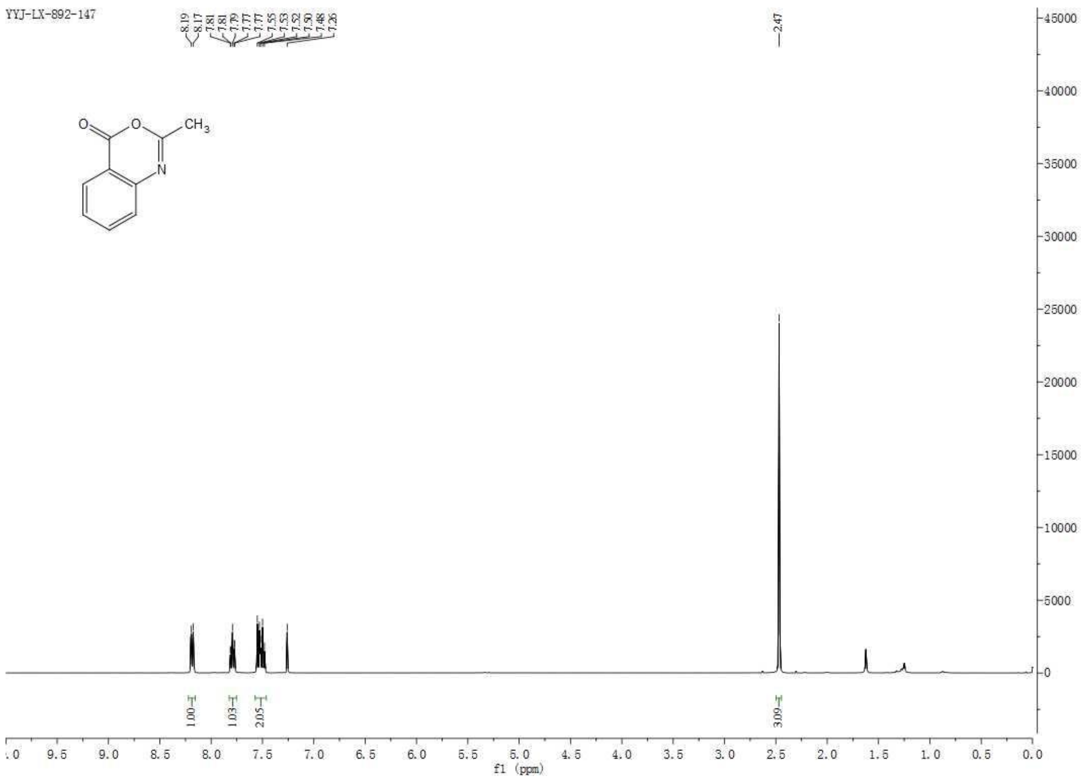

Supplementary Figure 143. The ^{1}H-NMR of compound **S13** in $CDCl_3$

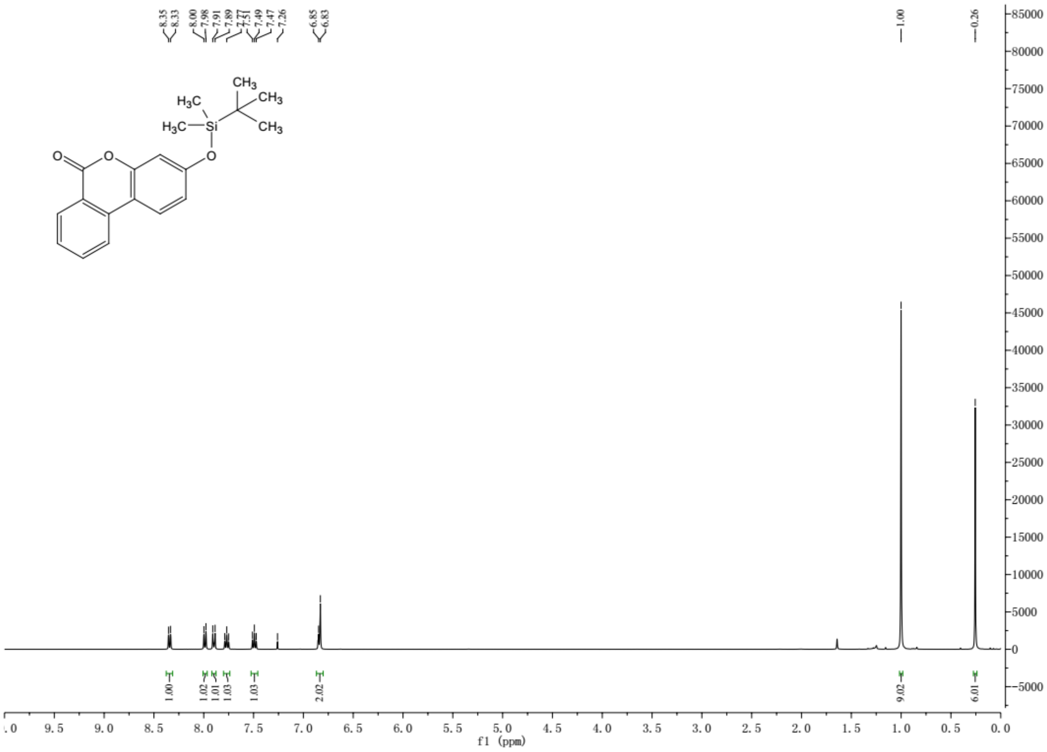

Supplementary Figure 144. The ^{1}H-NMR of compound **S19** in $CDCl_3$

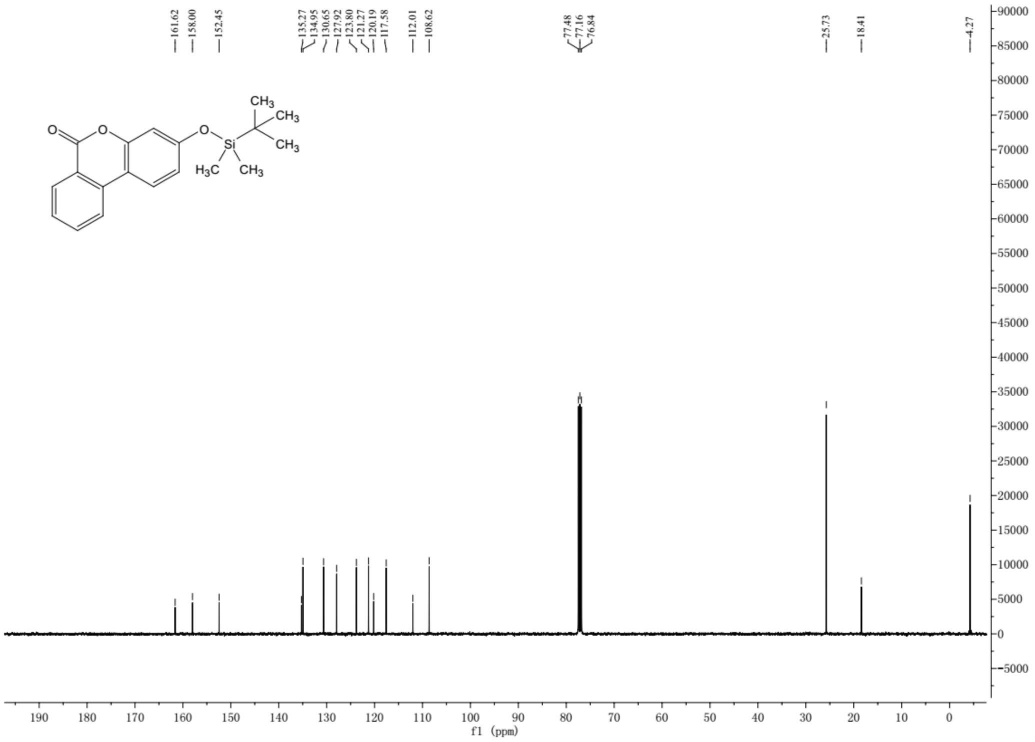

Supplementary Figure 145. The ^{13}C-NMR of compound **S19** in $CDCl_3$

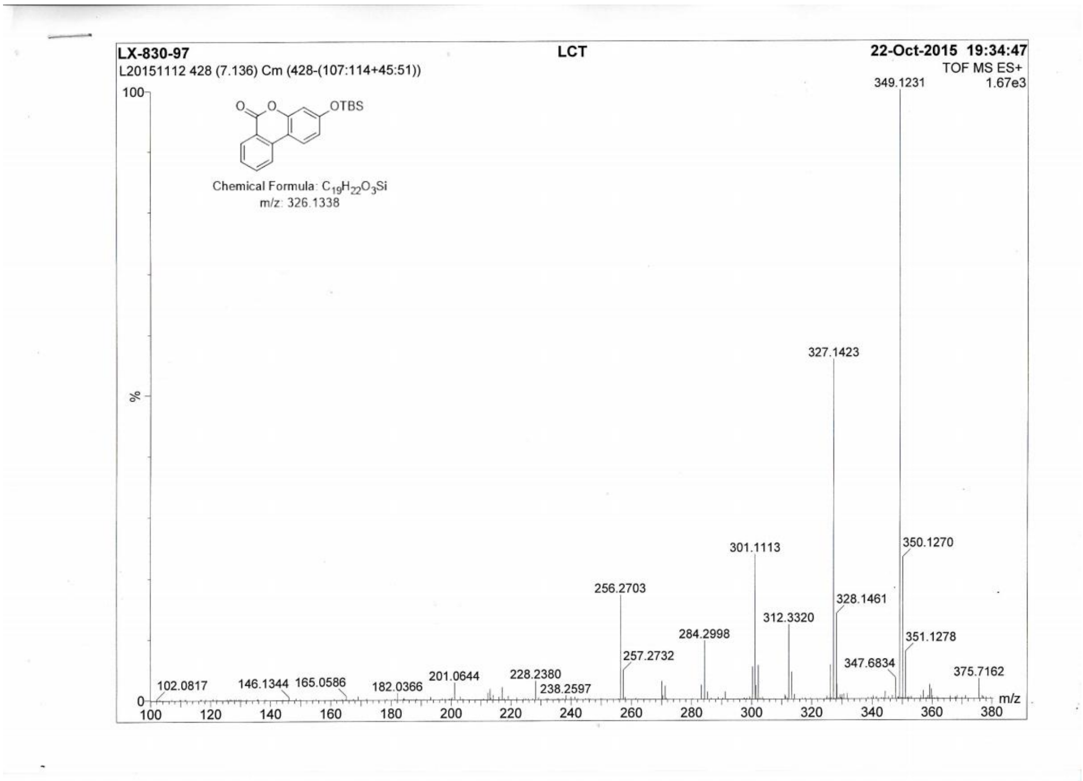

Supplementary Figure 146. The HR-MS of compound **S19**

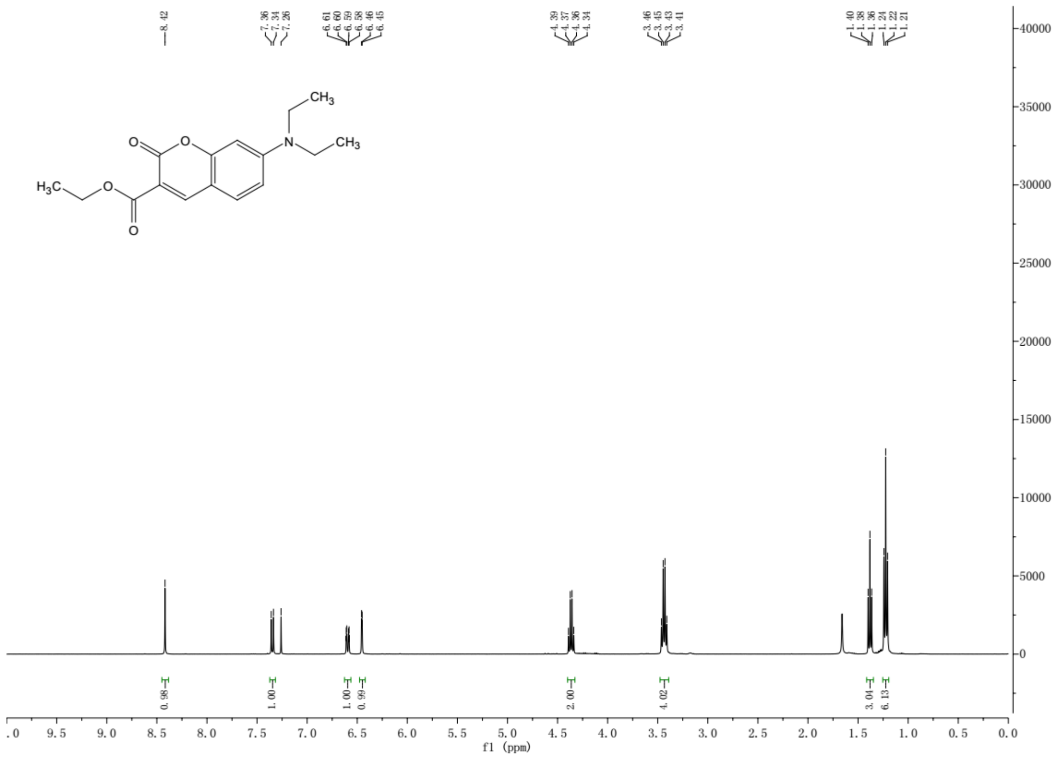

Supplementary Figure 147. The ^{1}H-NMR of compound **S42** in $CDCl_3$

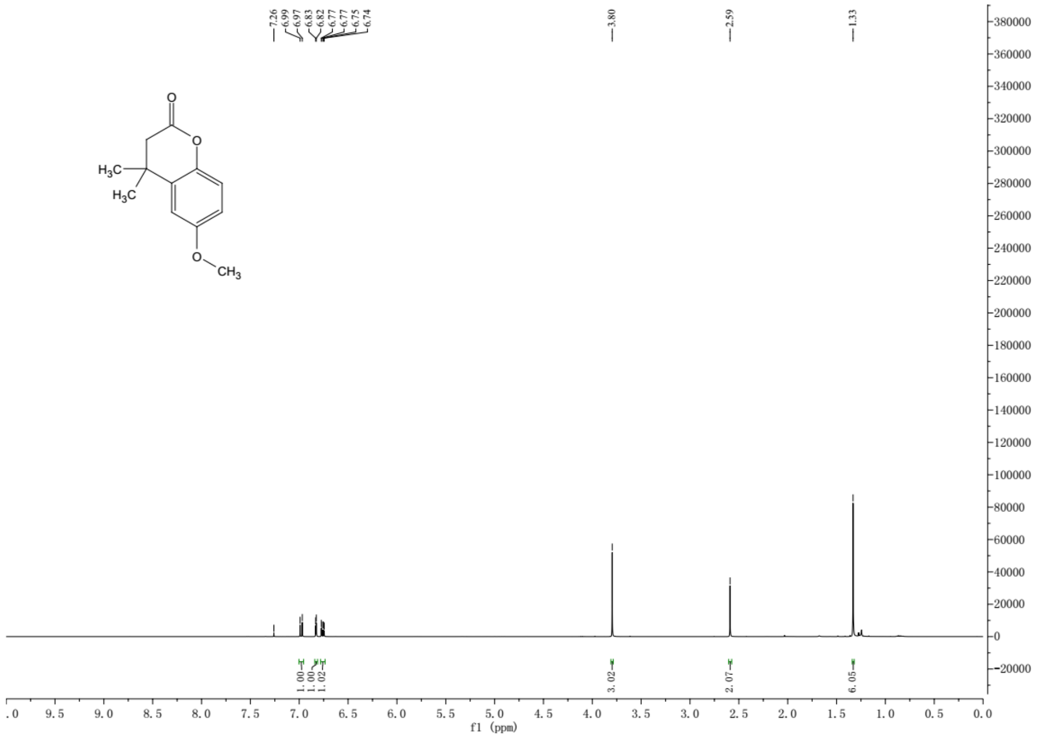

Supplementary Figure 148. The ^{1}H-NMR of compound **S51** in $CDCl_3$

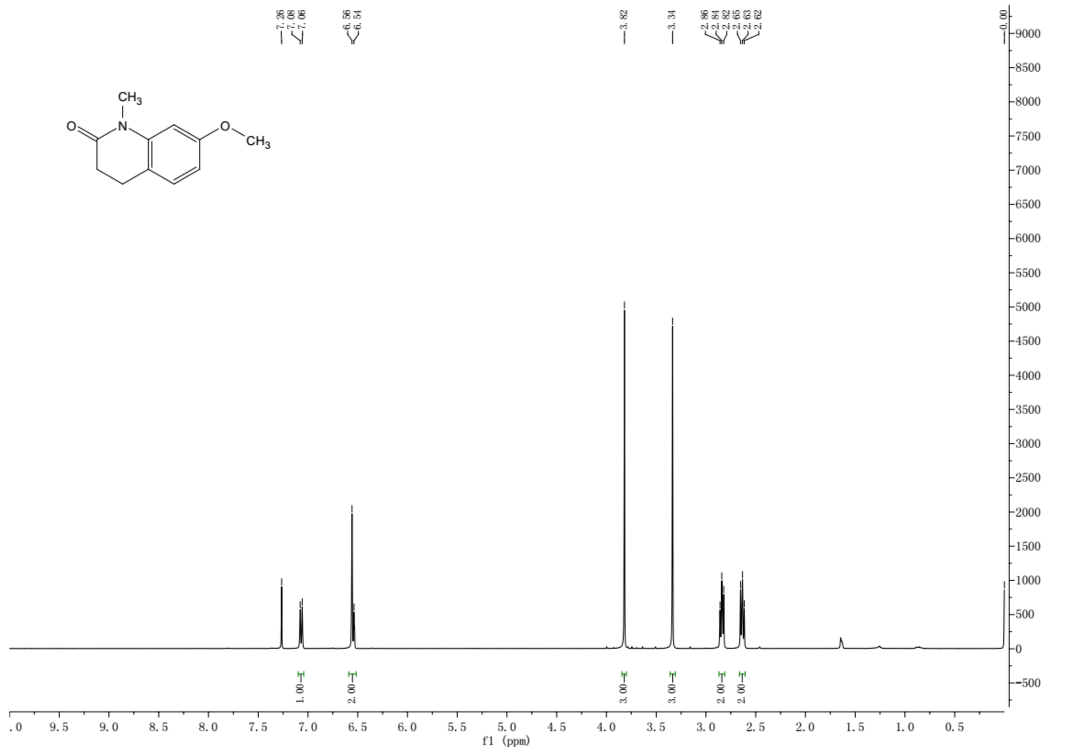

Supplementary Figure 149. The ^{1}H-NMR of compound **S52** in $CDCl_3$

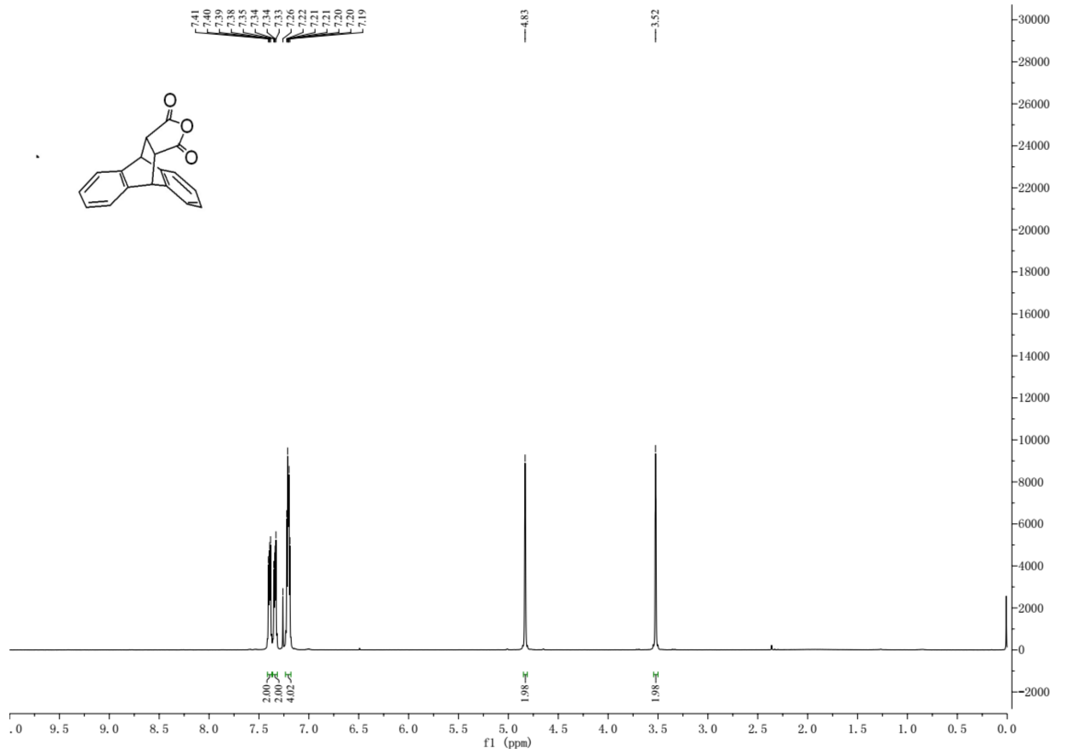

Supplementary Figure 150. The ^{1}H-NMR of compound **S58** in $CDCl_3$

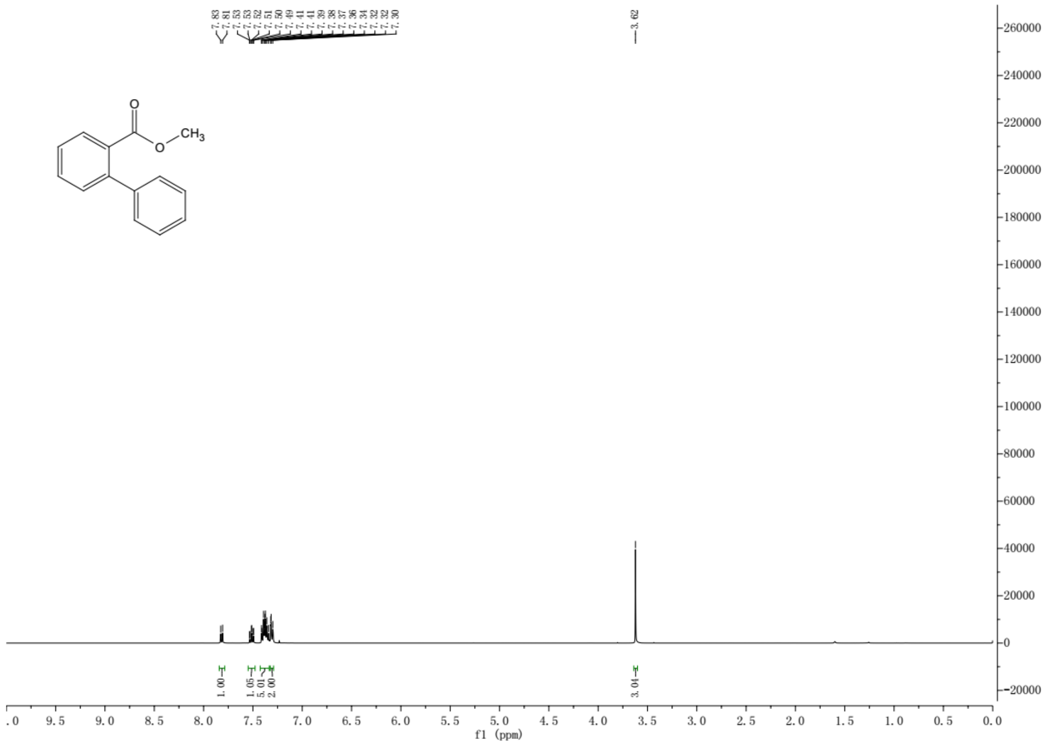

Supplementary Figure 151. The ^{1}H-NMR of compound **S16** in $CDCl_3$

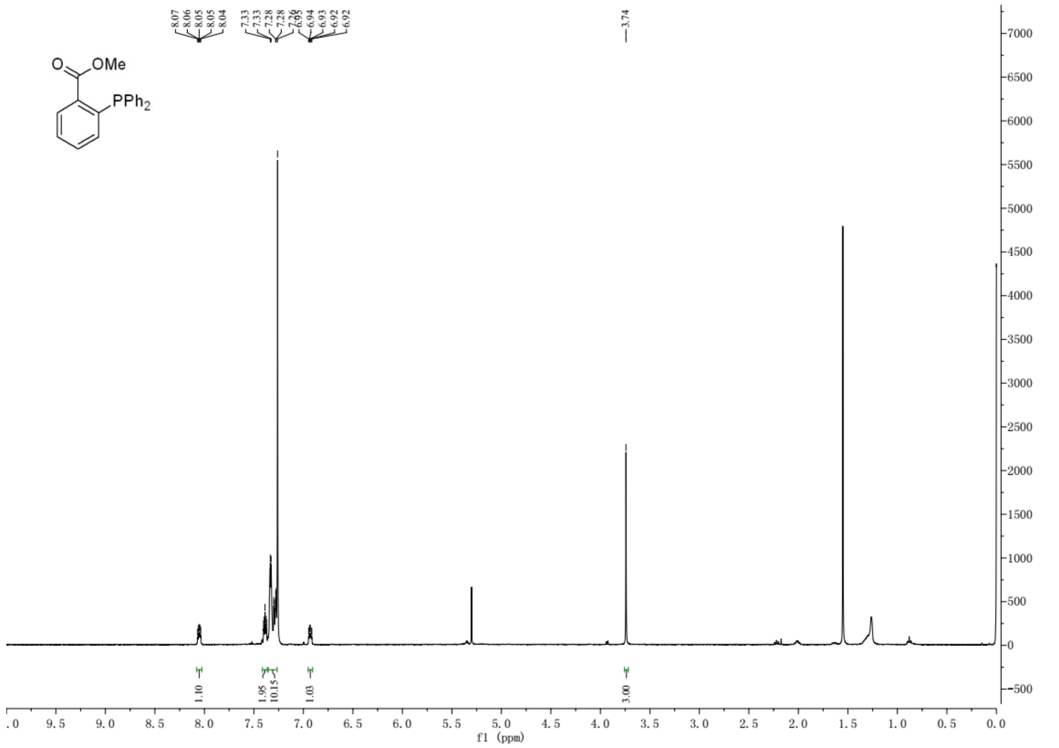

Supplementary Figure 152. The ^{1}H-NMR of compound **S17** in $CDCl_3$

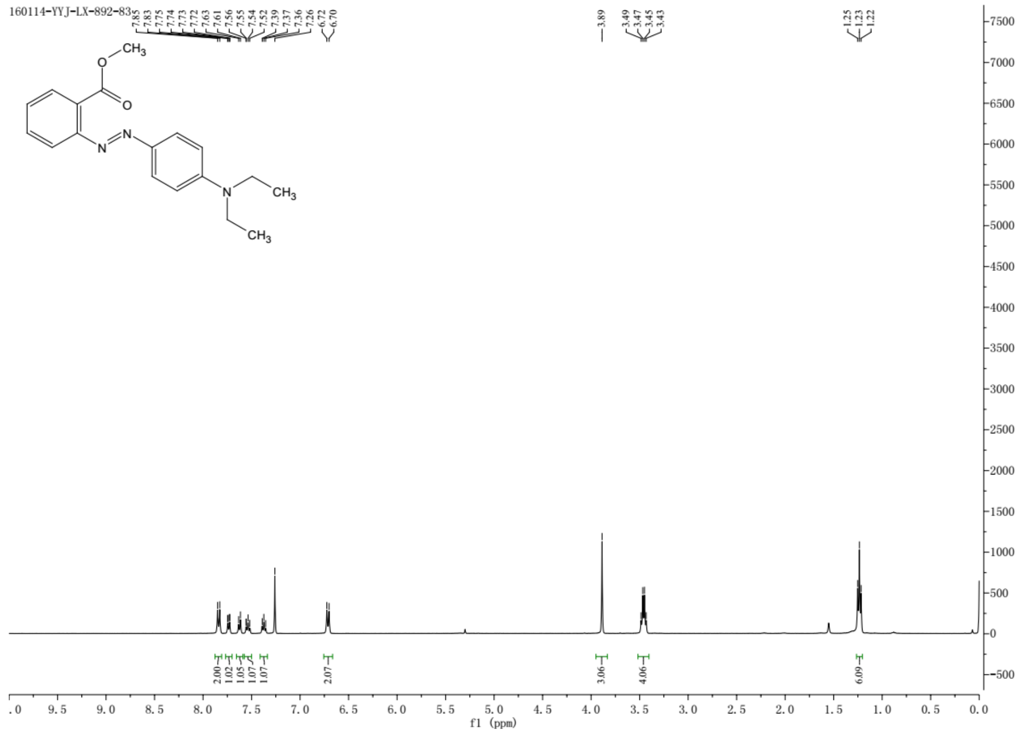

Supplementary Figure 153. The ^{1}H-NMR of compound **S21** in $CDCl_3$

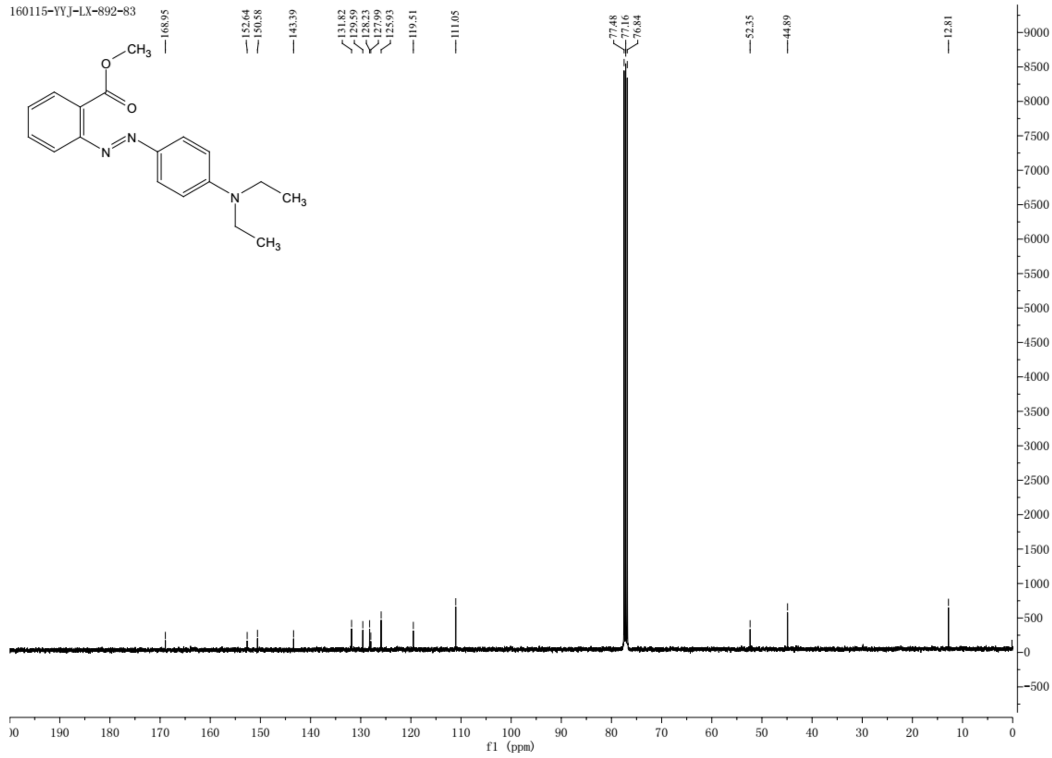

Supplementary Figure 154. The ^{13}C-NMR of compound **S21** in $CDCl_3$

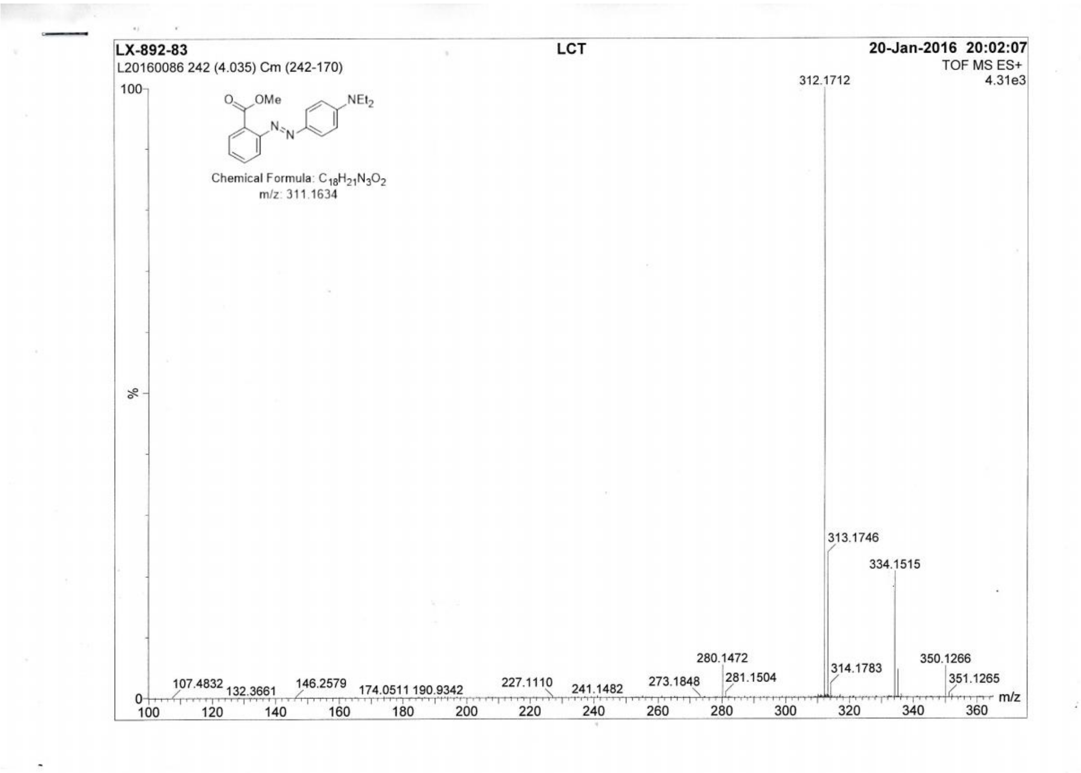

Supplementary Figure 155. The HR-MS of compound **S21**

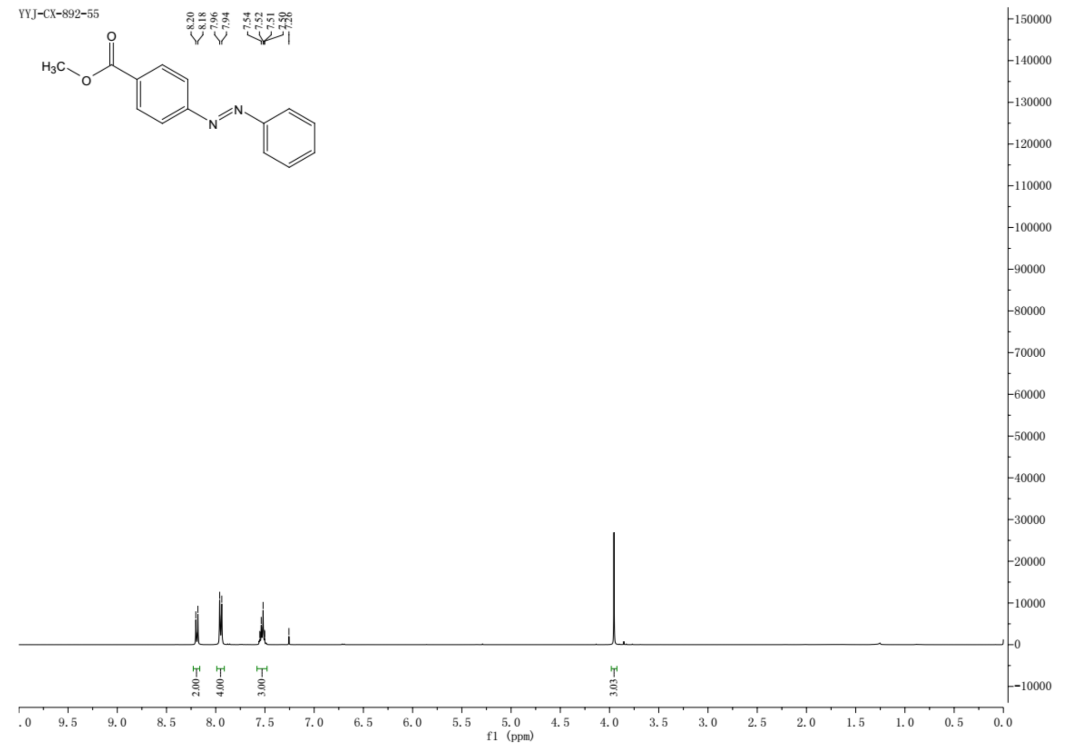

Supplementary Figure 156. The ^{1}H-NMR of compound **S22** in $CDCl_3$

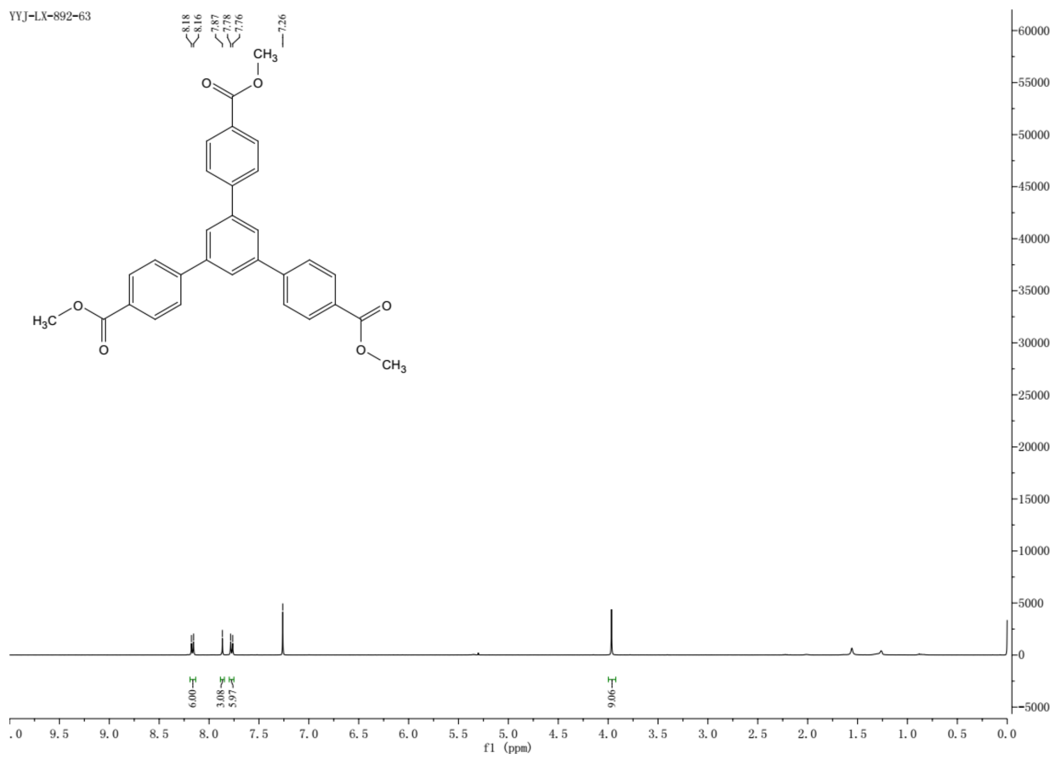

Supplementary Figure 157. The ^{1}H-NMR of compound **S24** in $CDCl_3$

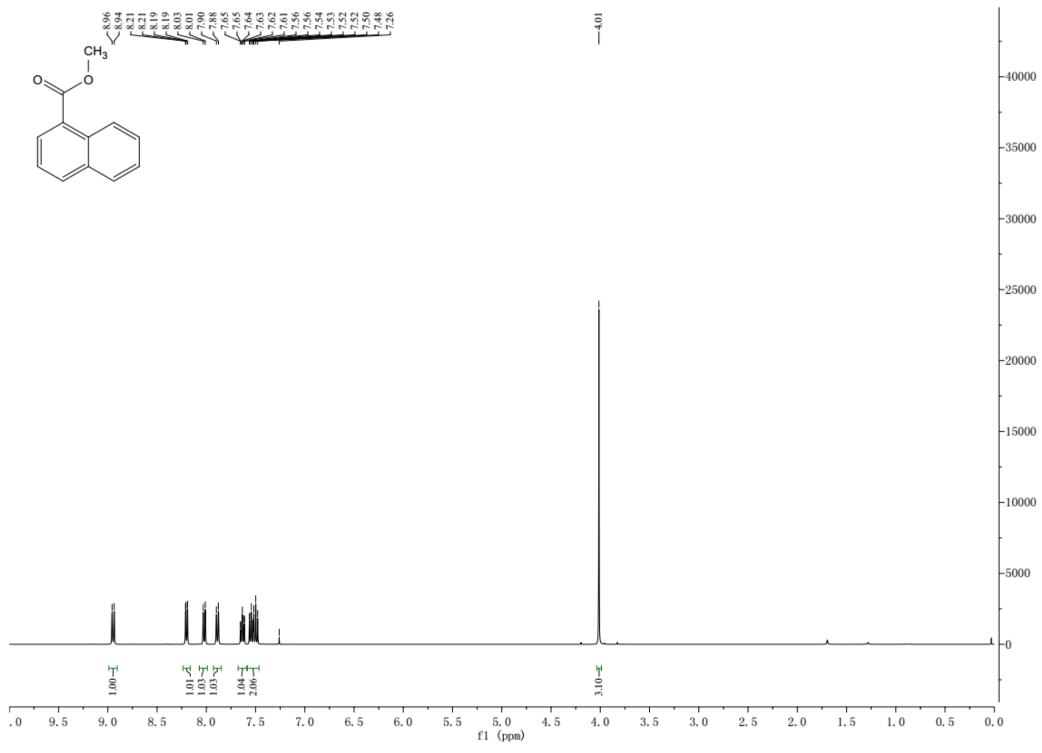

Supplementary Figure 158. The ^{1}H-NMR of compound **S25** in $CDCl_3$

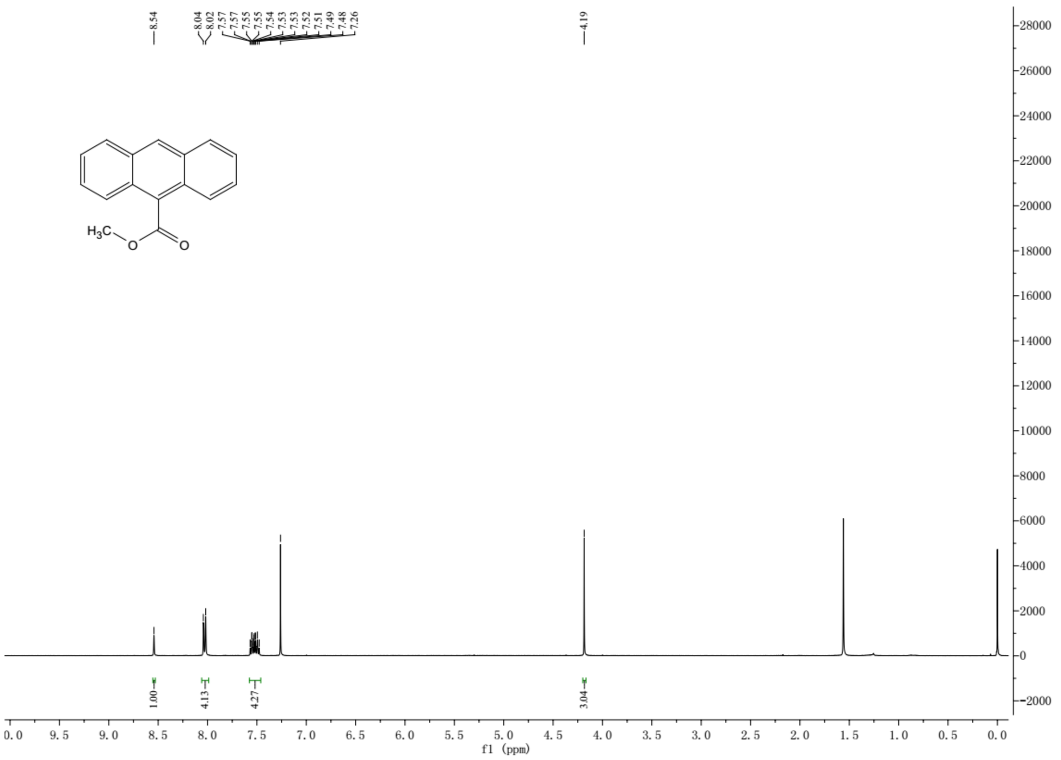

Supplementary Figure 159. The ^{1}H-NMR of compound **S27** in $CDCl_3$

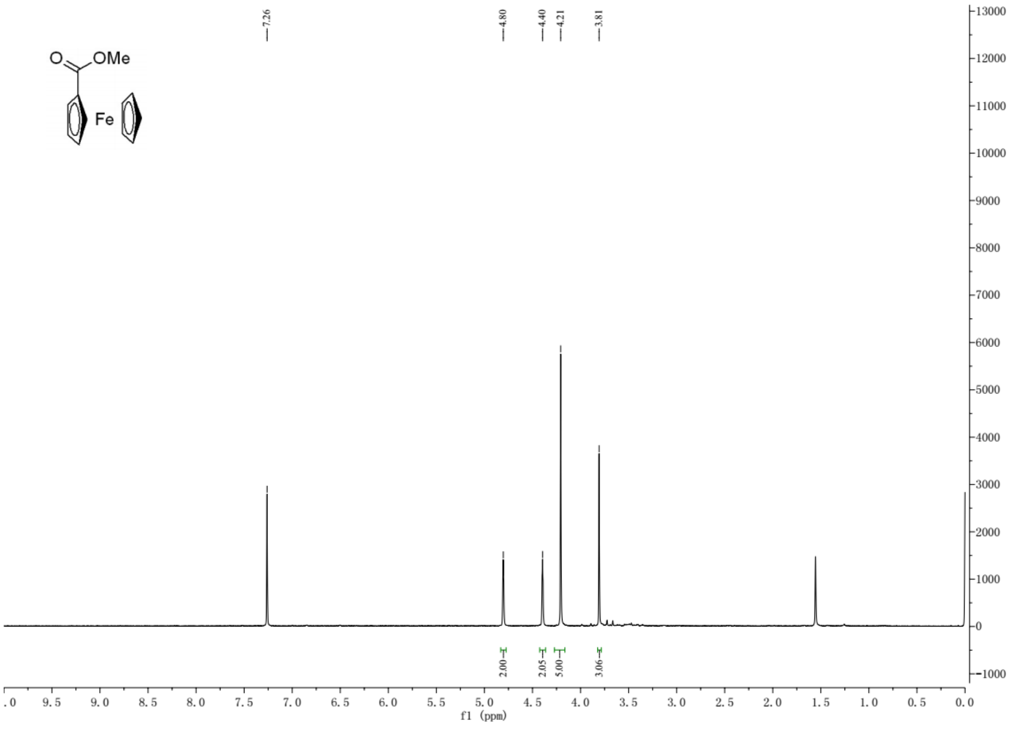

Supplementary Figure 160. The ^{1}H-NMR of compound **S39** in $CDCl_3$

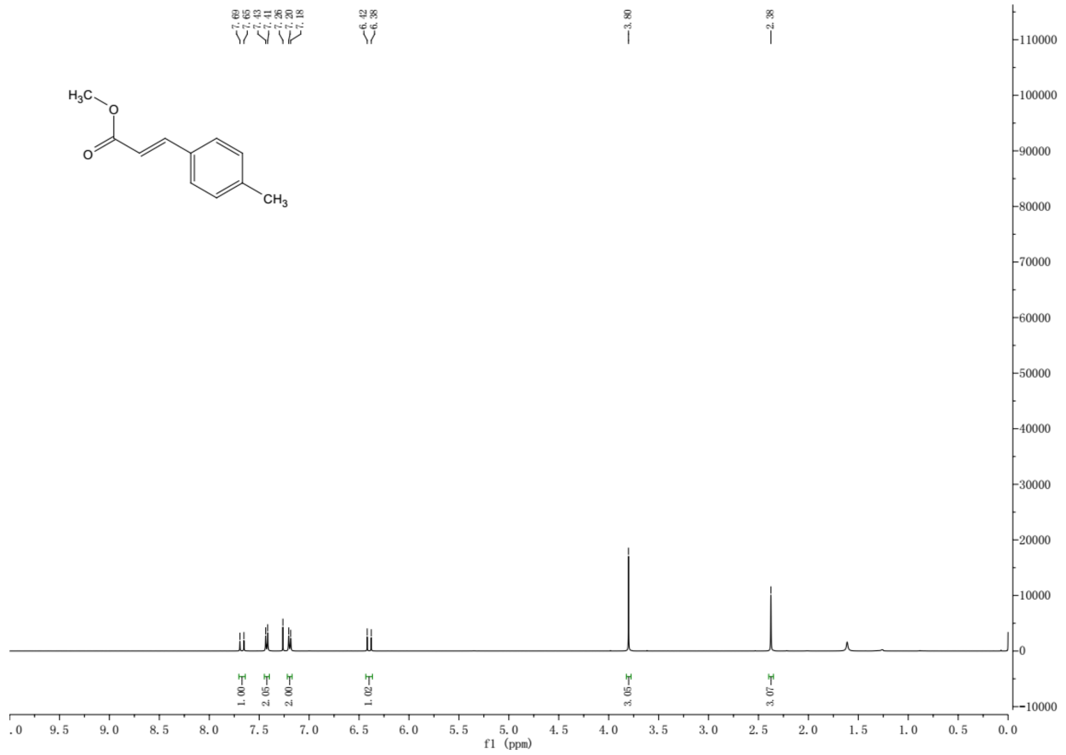

Supplementary Figure 161. The ^{1}H-NMR of compound **S44** in $CDCl_3$

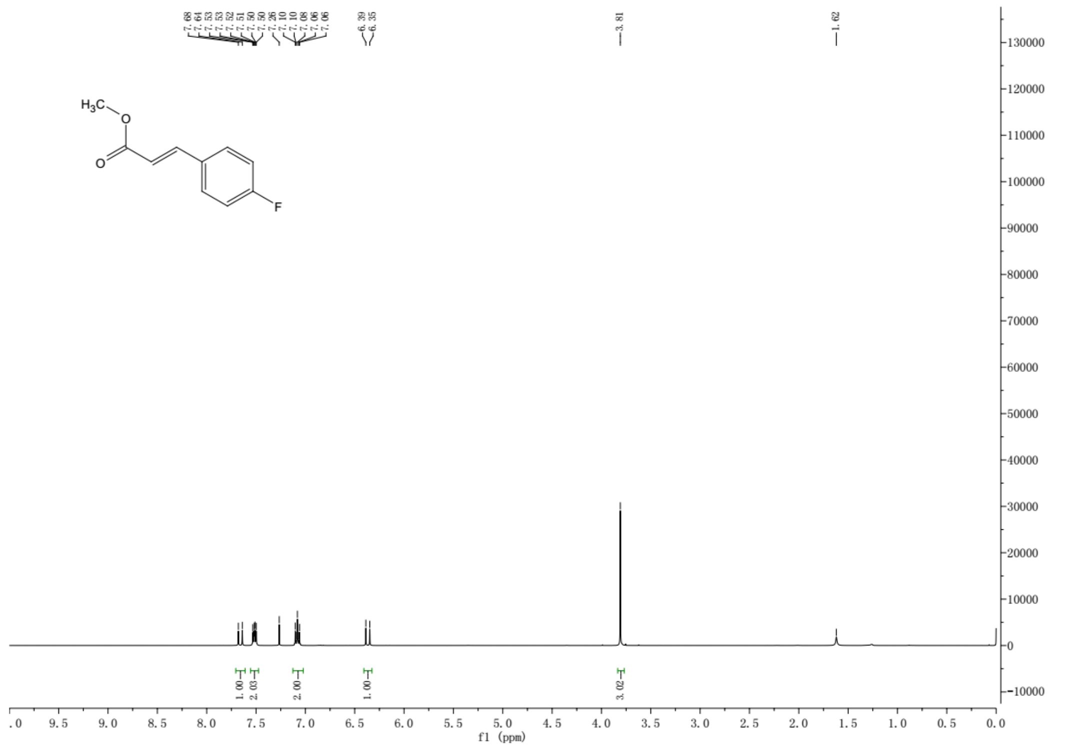

Supplementary Figure 162. The ^{1}H-NMR of compound **S45** in $CDCl_3$

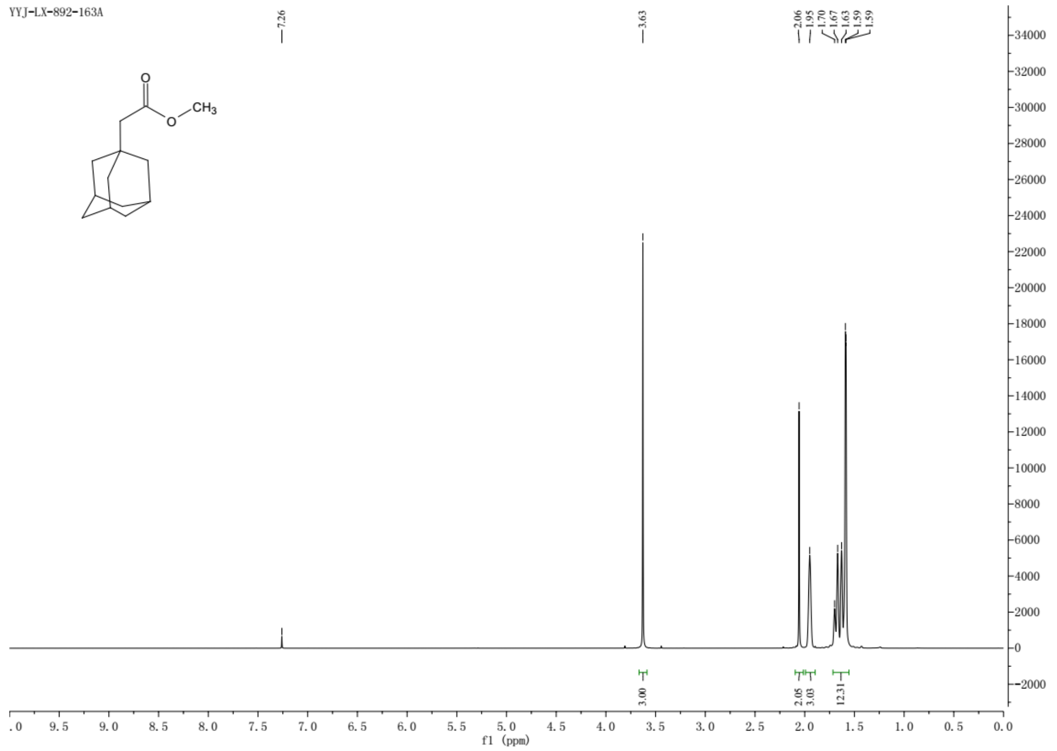

Supplementary Figure 163. The ^{1}H-NMR of compound S**53** in $CDCl_3$

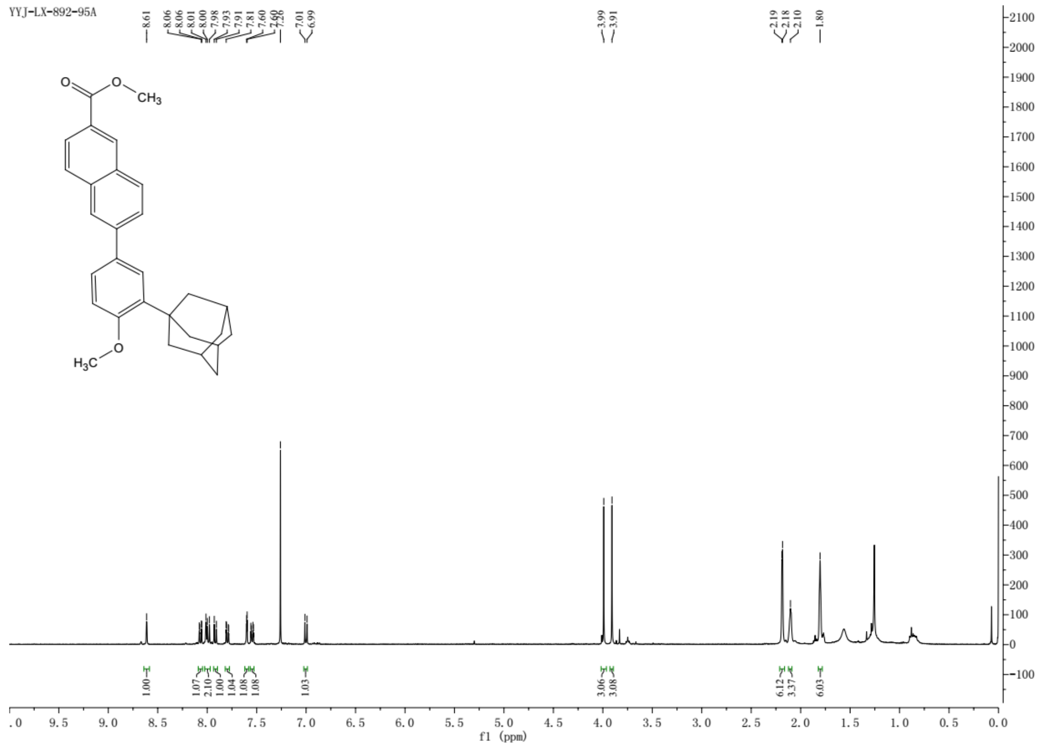

Supplementary Figure 164. The ^{1}H-NMR of compound **S62** in $CDCl_3$

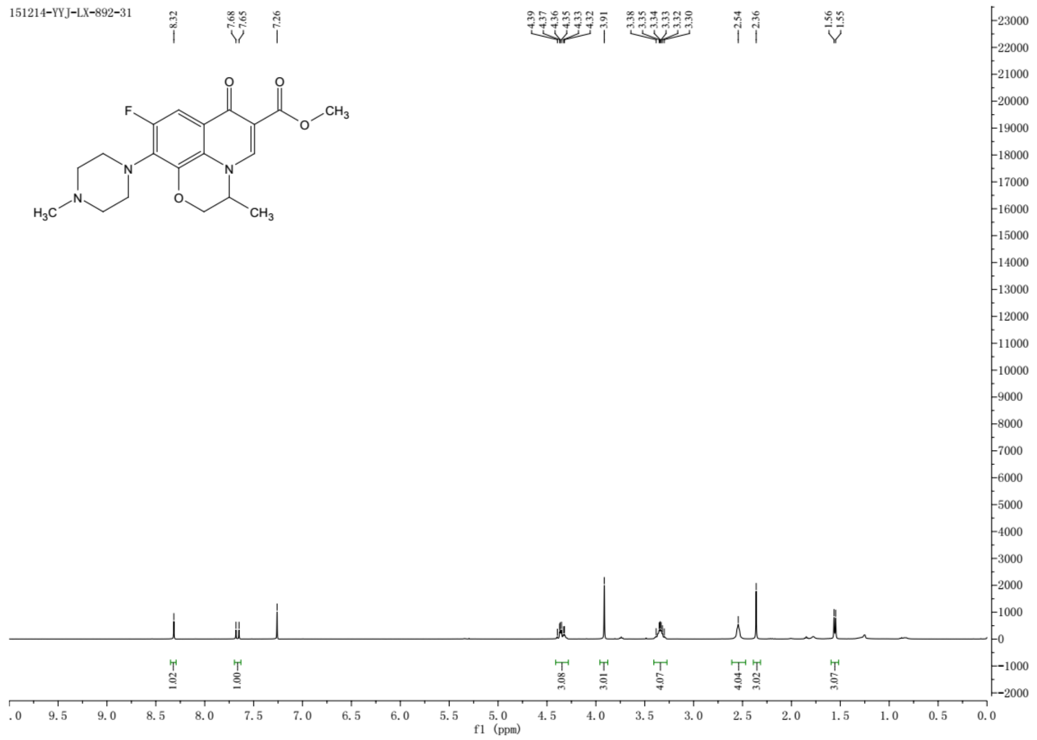

Supplementary Figure 165. The ^{1}H-NMR of compound **S66** in $CDCl_3$

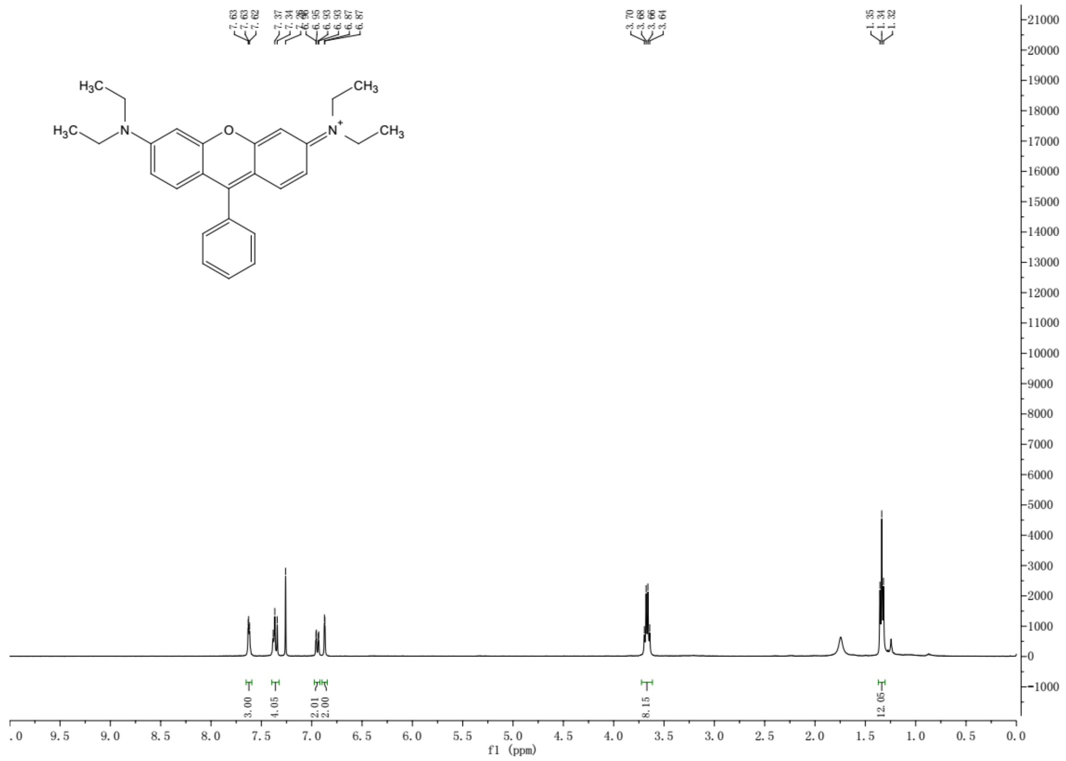

Supplementary Figure 166. The ^{1}H-NMR of compound **RD1** in $CDCl_3$

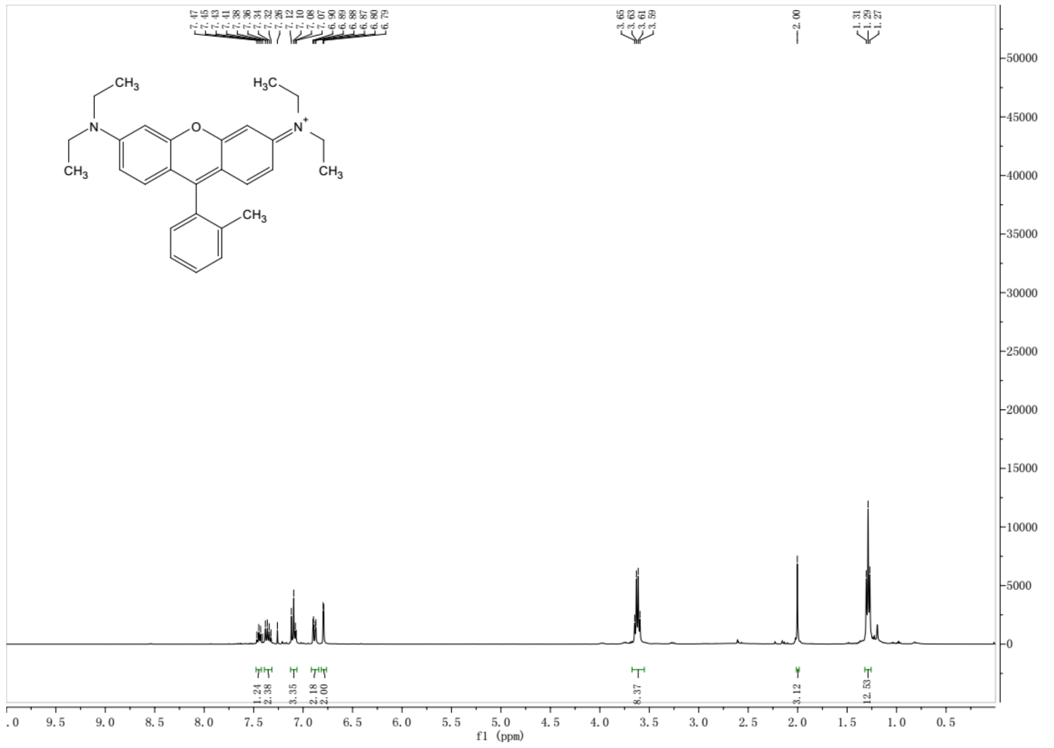

Supplementary Figure 167. The ^{1}H-NMR of compound **RD2** in $CDCl_3$

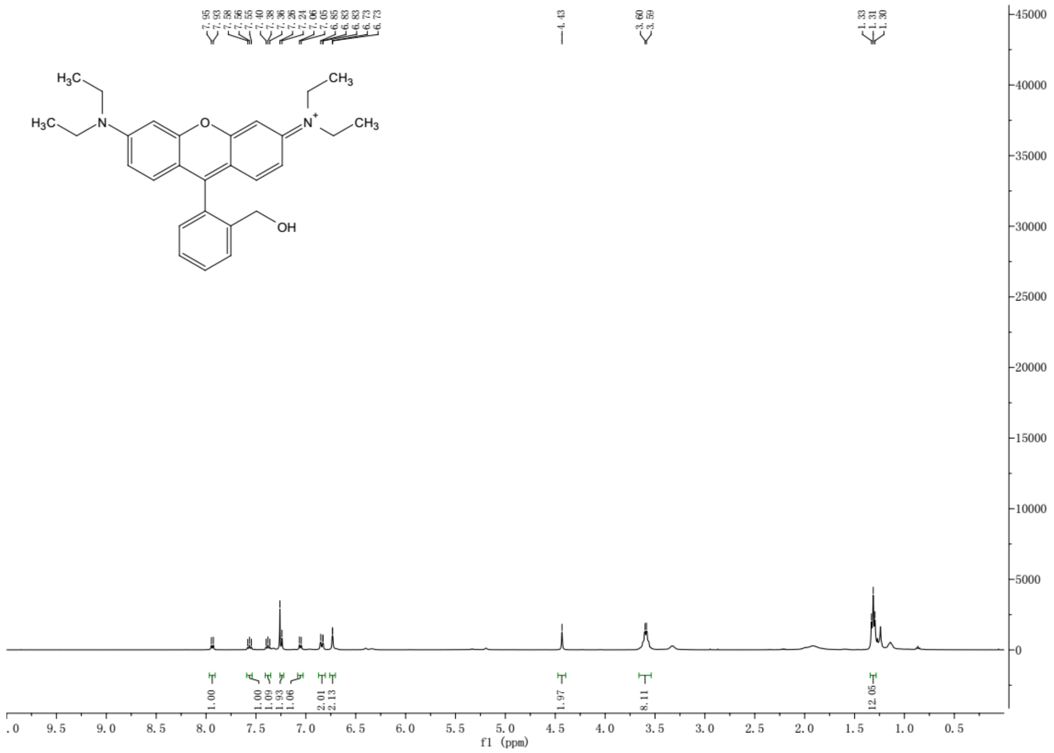

Supplementary Figure 168. The ^{1}H-NMR of compound **RD3** in $CDCl_3$

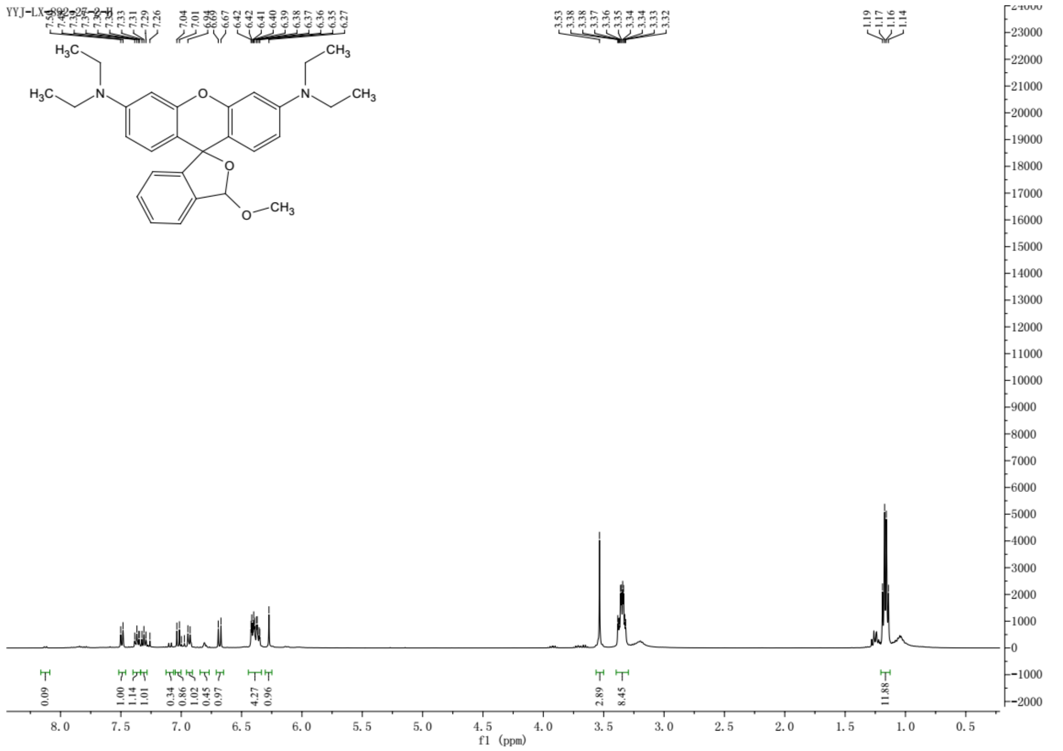

Supplementary Figure 169. The ^{1}H-NMR of compound **RD4** in $CDCl_3$

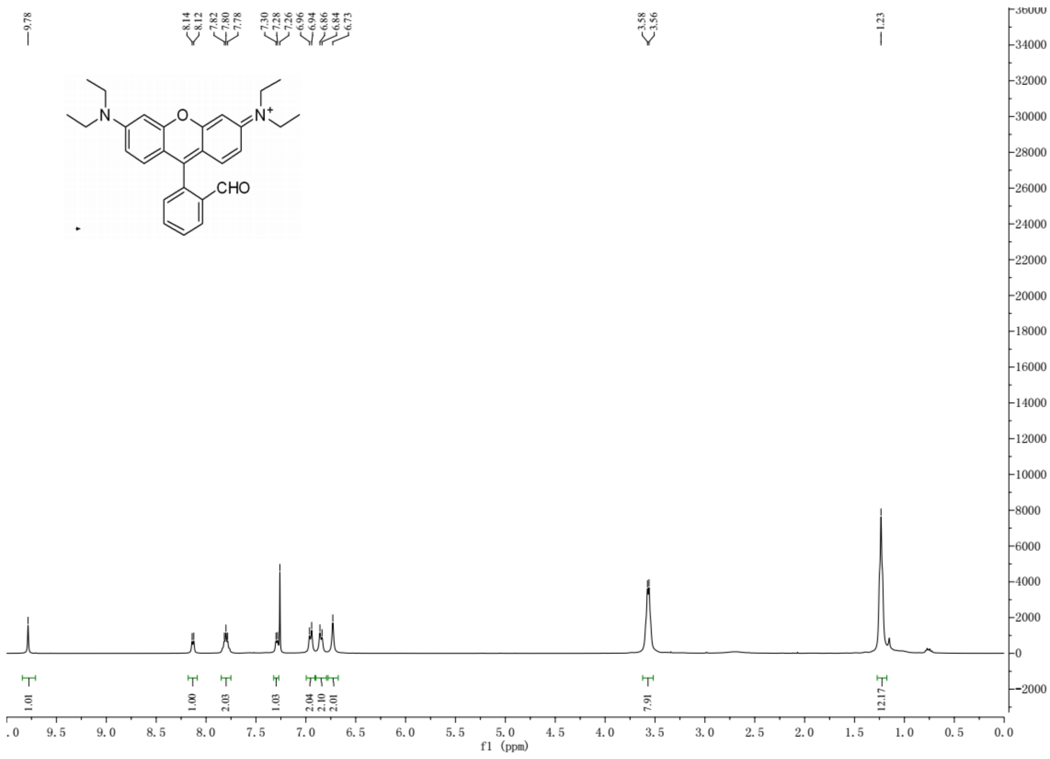

Supplementary Figure 170. The ^{1}H-NMR of compound **RD5** in $CDCl_3$

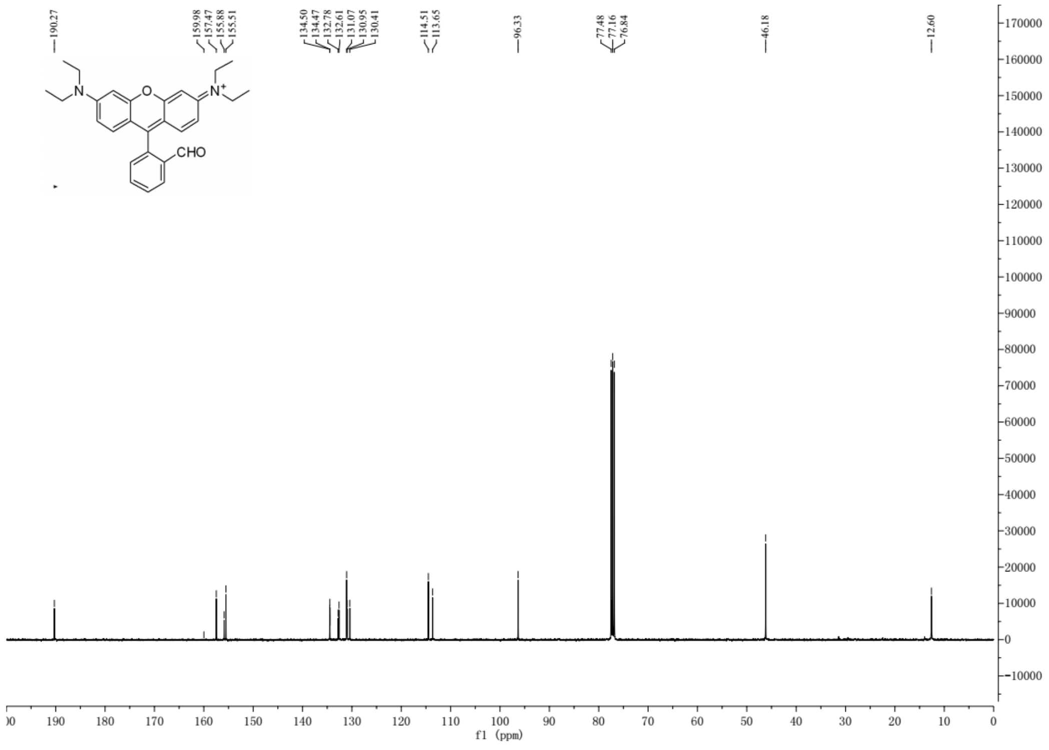

Supplementary Figure 171. The ^{13}C-NMR of compound **RD5** in $CDCl_3$

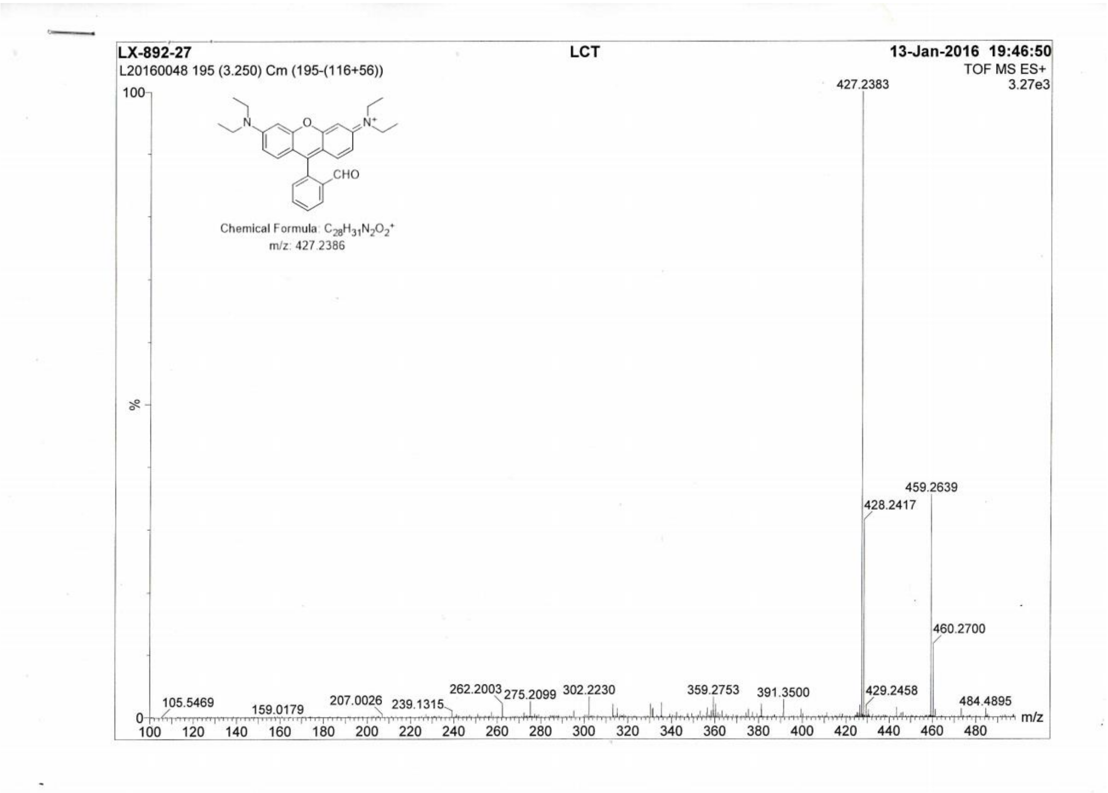

Supplementary Figure 172. The HR-MS of compound **RD5**

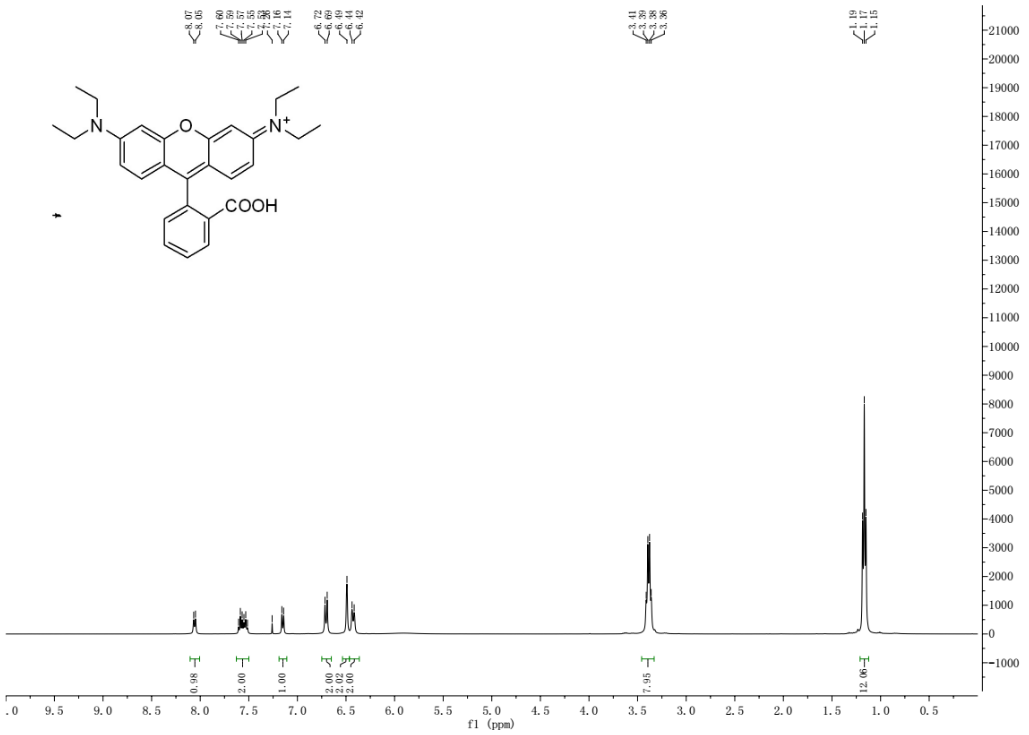

Supplementary Figure 173. The ^{1}H-NMR of compound **RD6** in $CDCl_3$

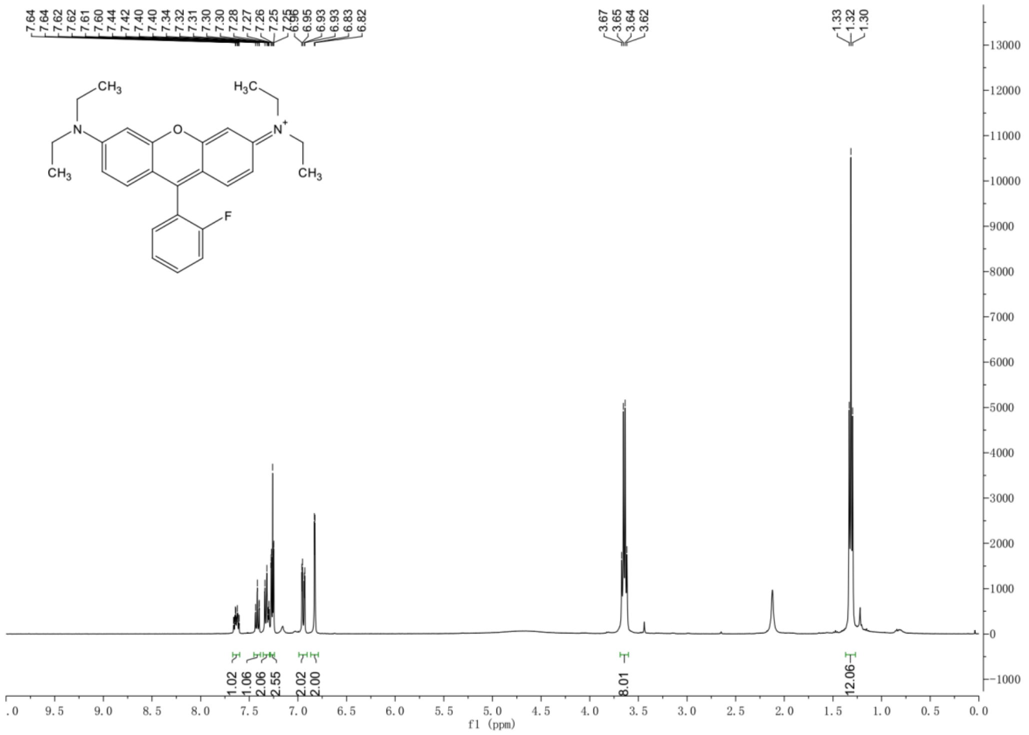

Supplementary Figure 174. The ^{1}H-NMR of compound **RD7** in $CDCl_3$

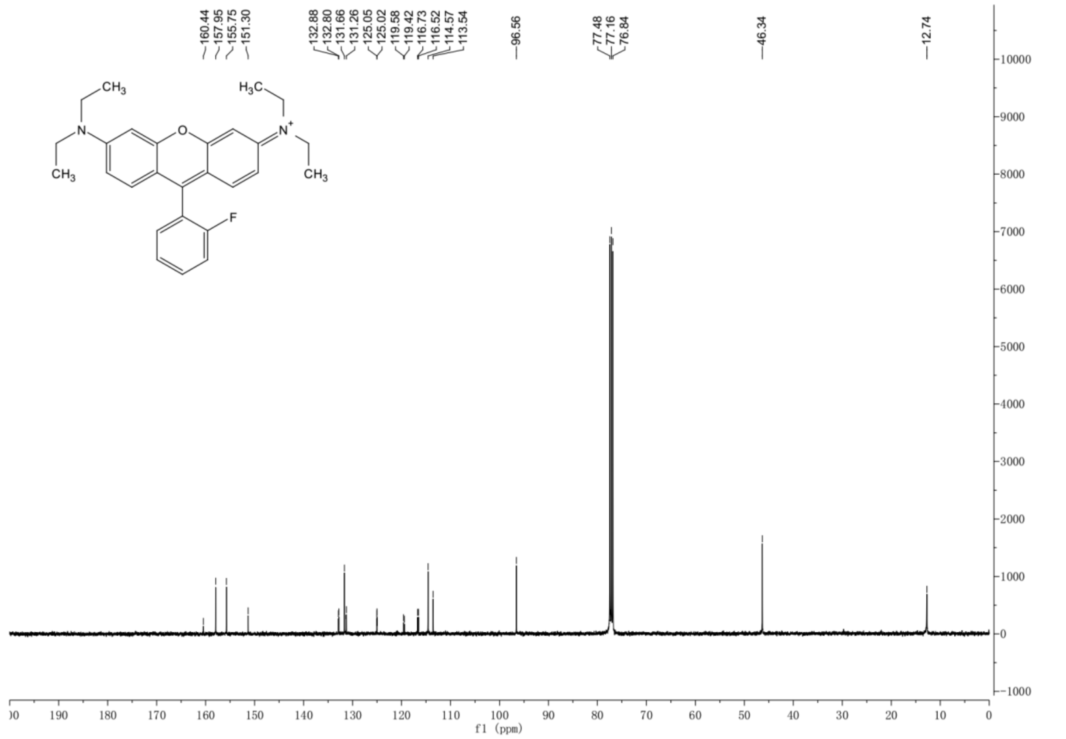

Supplementary Figure 175. The ^{13}C-NMR of compound **RD7** in $CDCl_3$

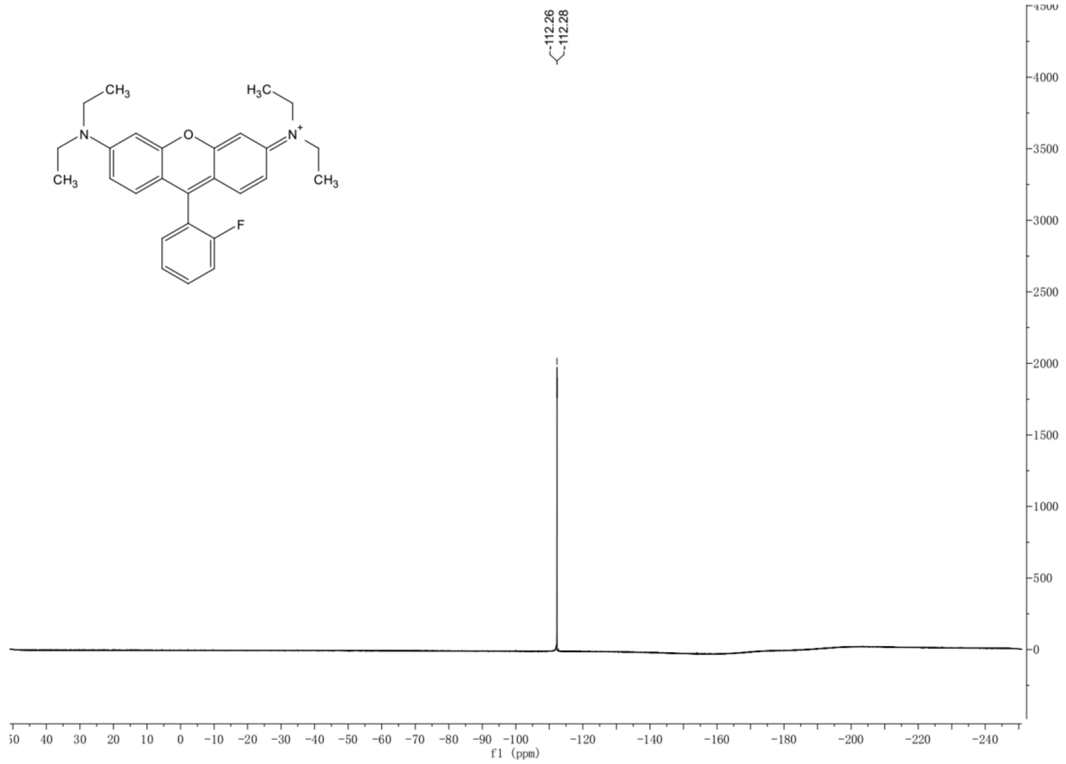

Supplementary Figure 176. The ^{19}F-NMR of compound **RD7** in $CDCl_3$

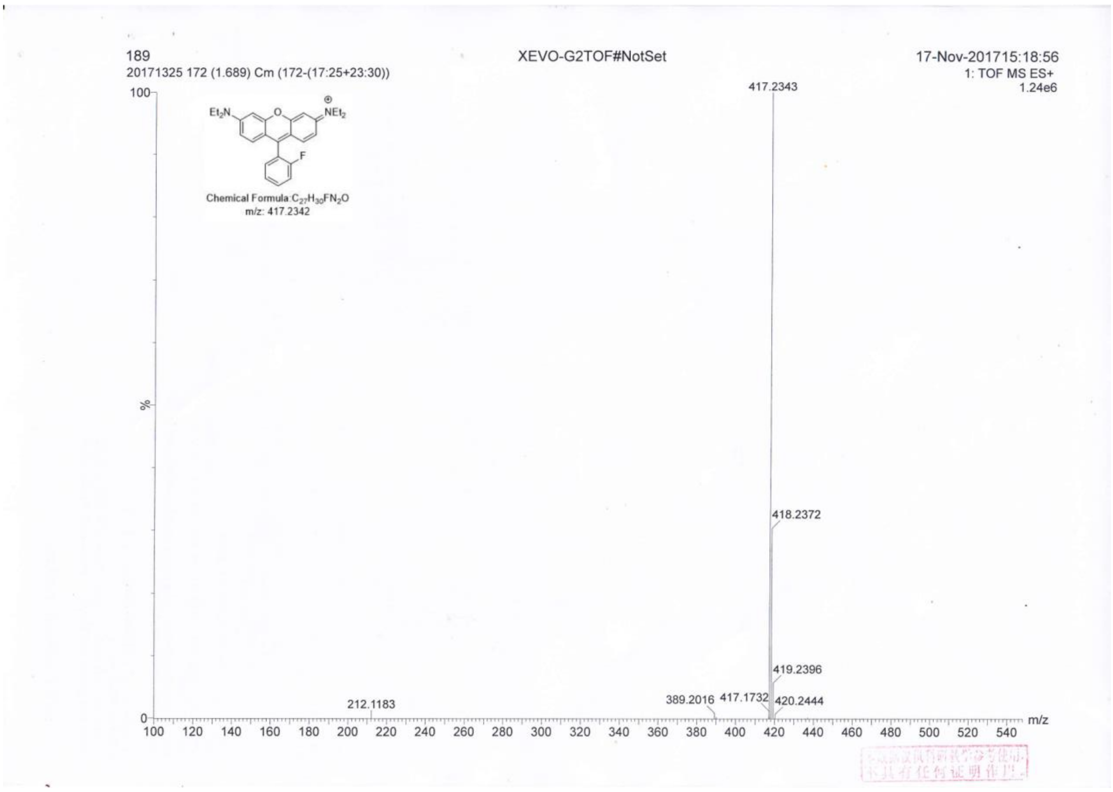

Supplementary Figure 177. The HR-MS of compound **RD7**

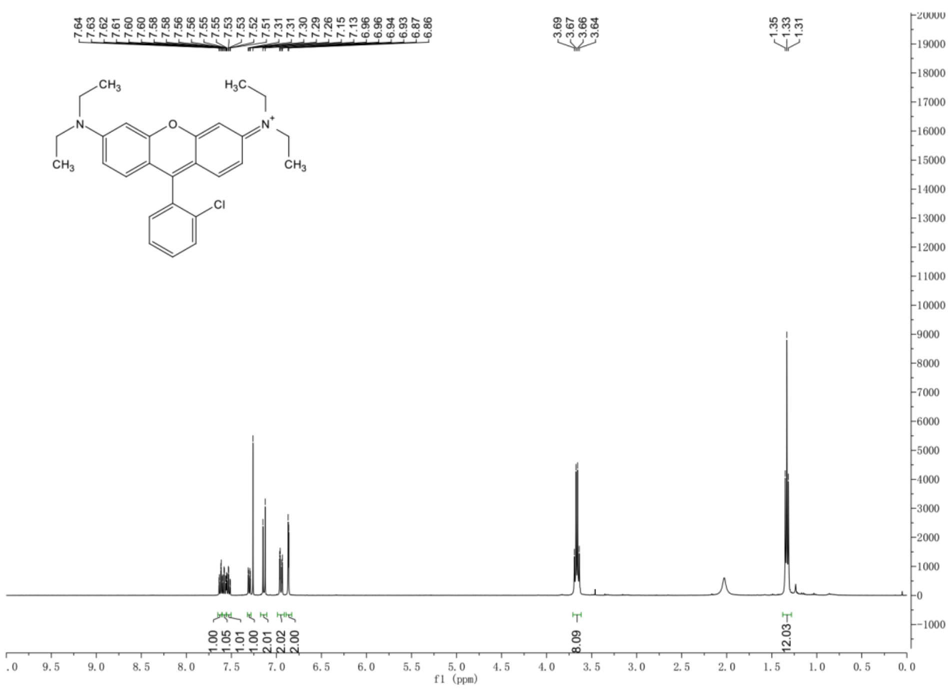

Supplementary Figure 178. The ^{1}H-NMR of compound **RD8** in $CDCl_3$

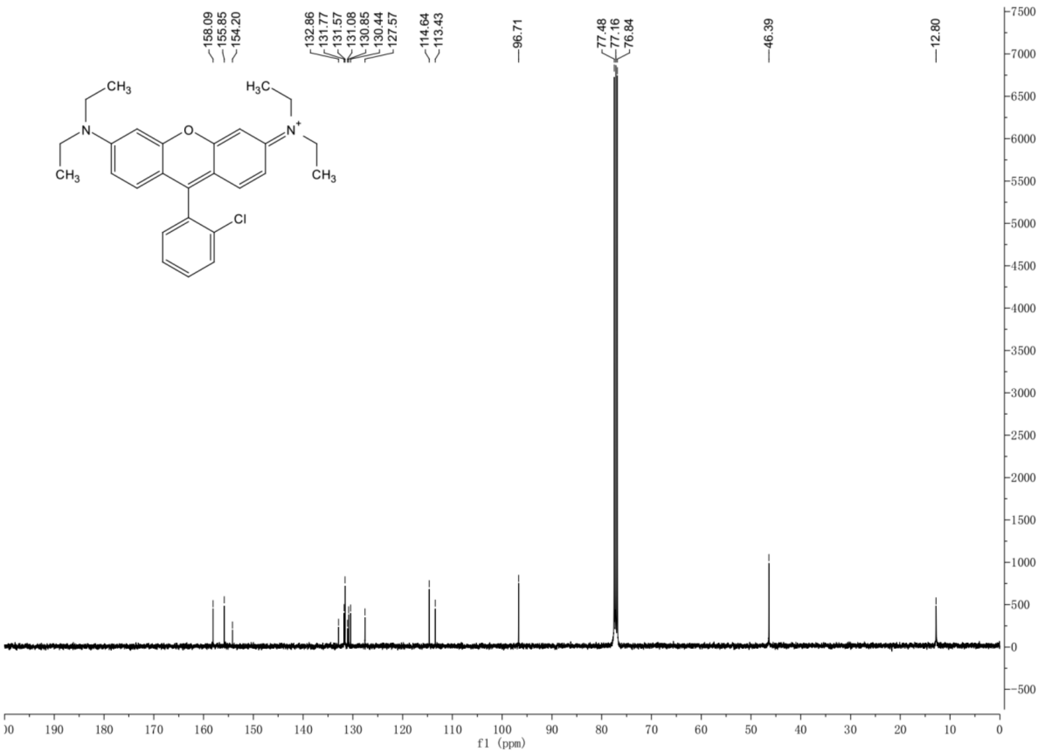

Supplementary Figure 179. The ^{13}C-NMR of compound **RD8** in $CDCl_3$

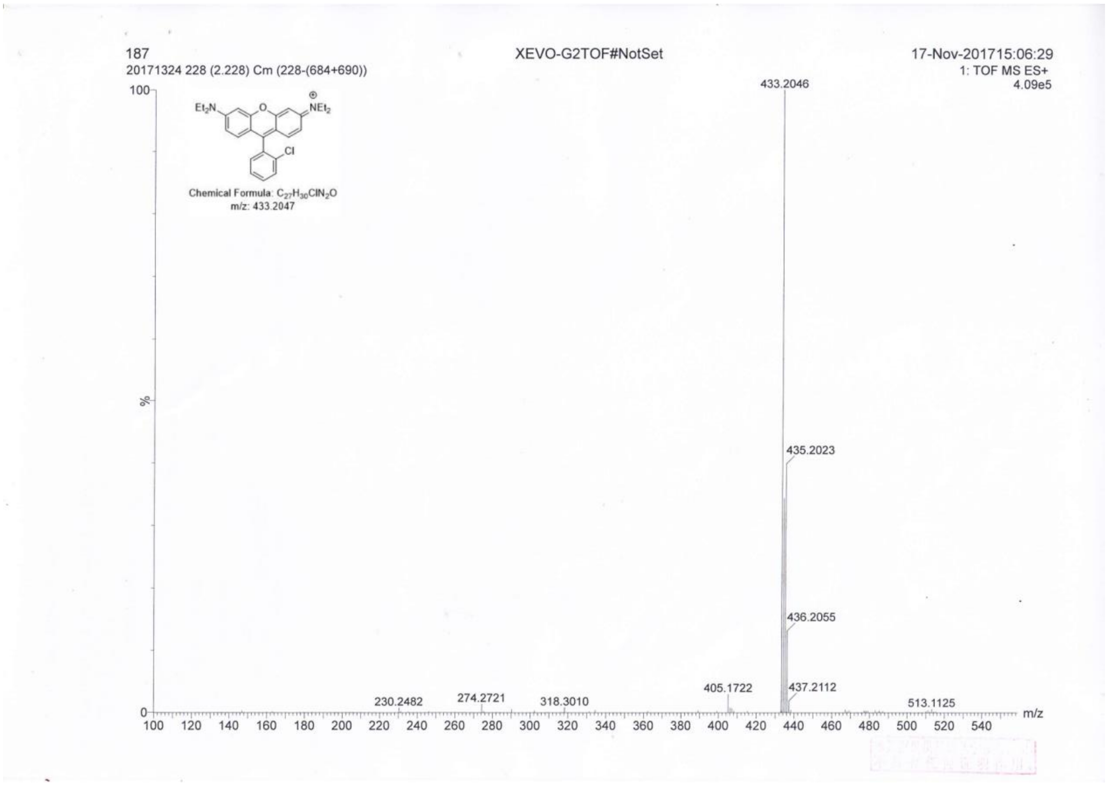

Supplementary Figure 180. The HR-MS of compound **RD8**

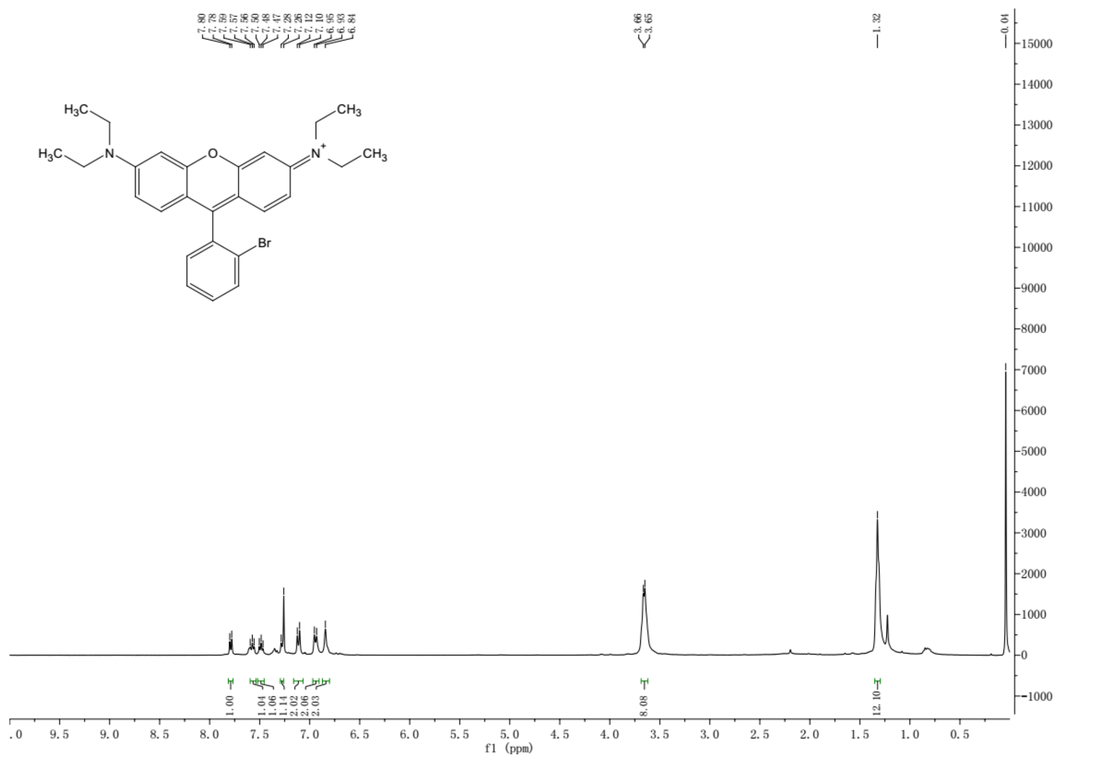

Supplementary Figure 181. The ^{1}H-NMR of compound **RD9** in $CDCl_3$

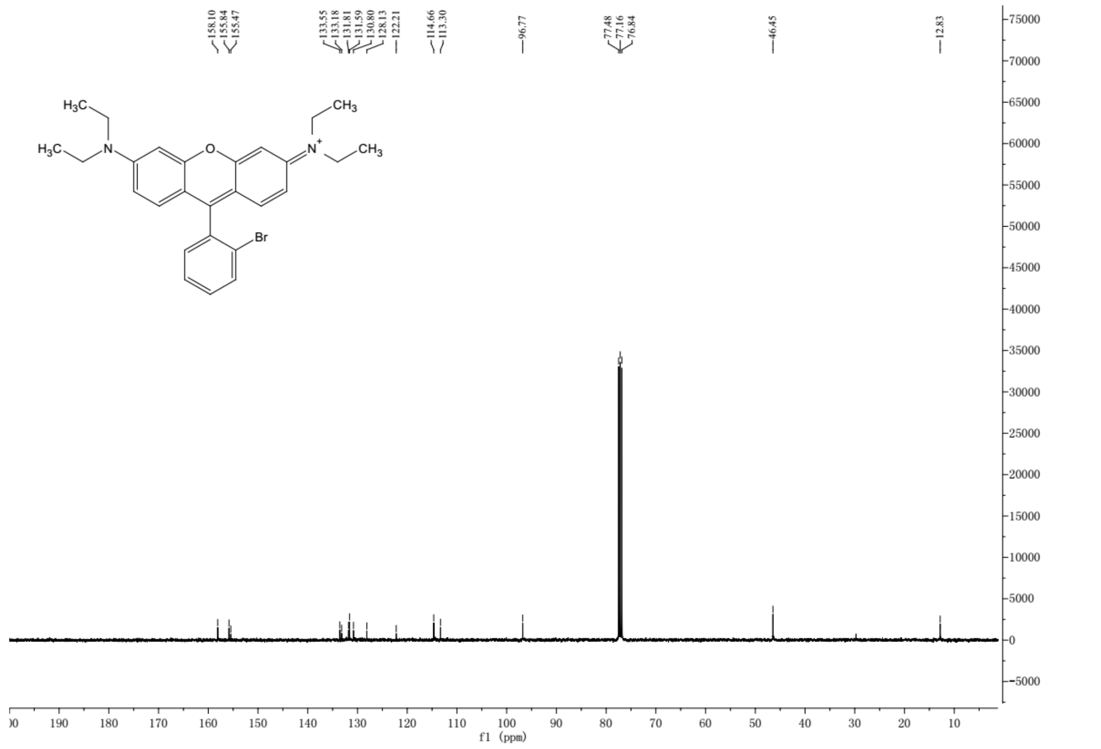

Supplementary Figure 182. The ^{13}C-NMR of compound **RD9** in $CDCl_3$

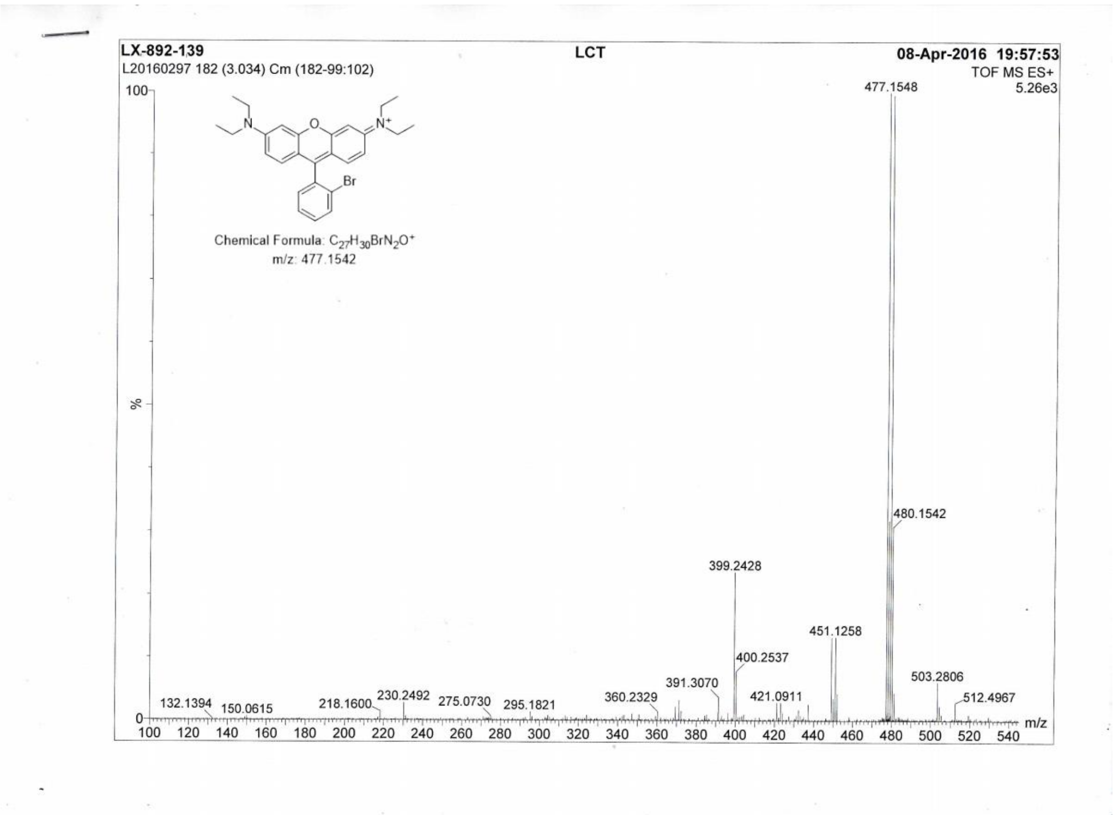

Supplementary Figure 183. The HR-MS of compound **RD9**

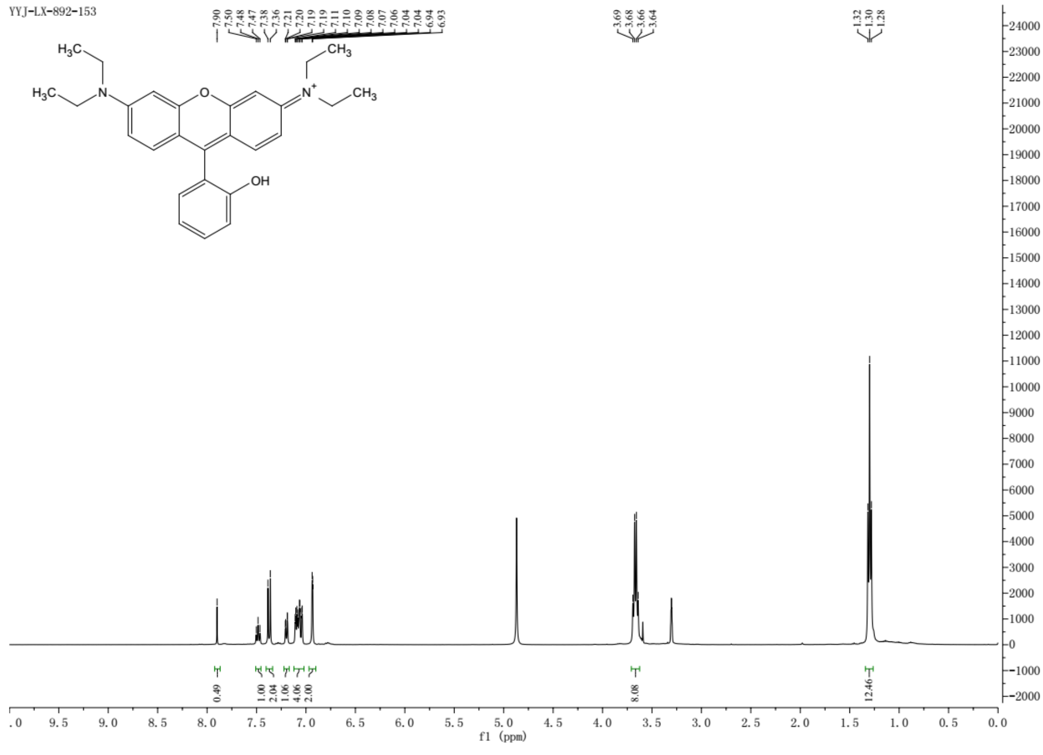

Supplementary Figure 184. The ^{1}H-NMR of compound **RD10** in CD_3OD

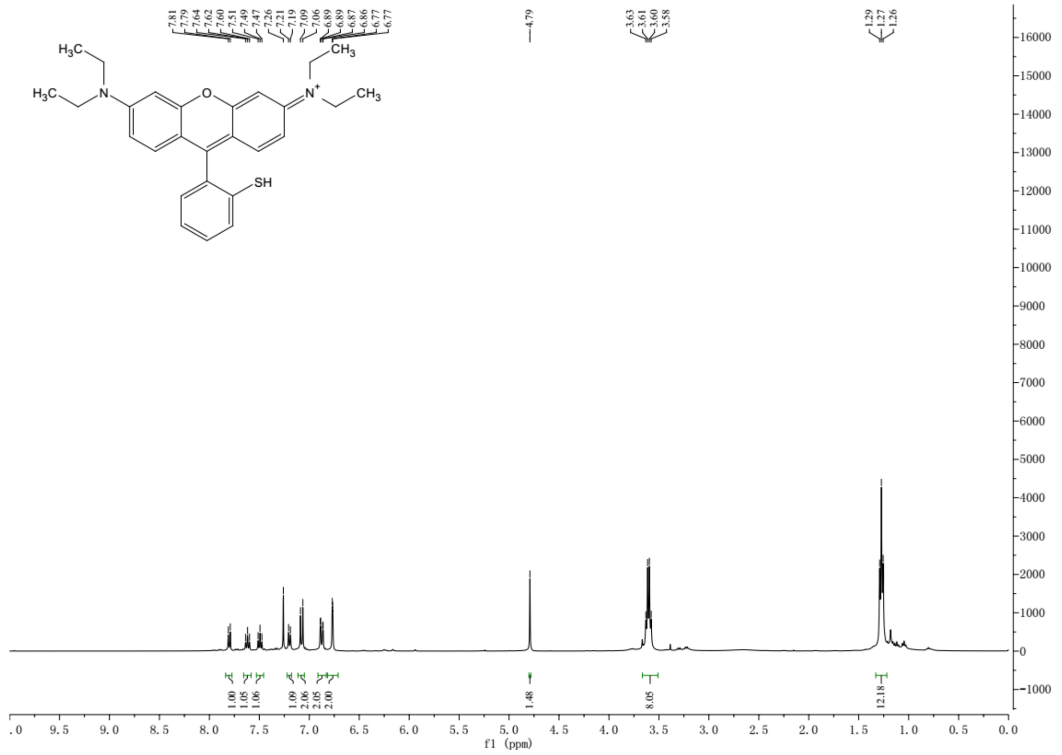

Supplementary Figure 185. The ^{1}H-NMR of compound **RD11** in $CDCl_3$

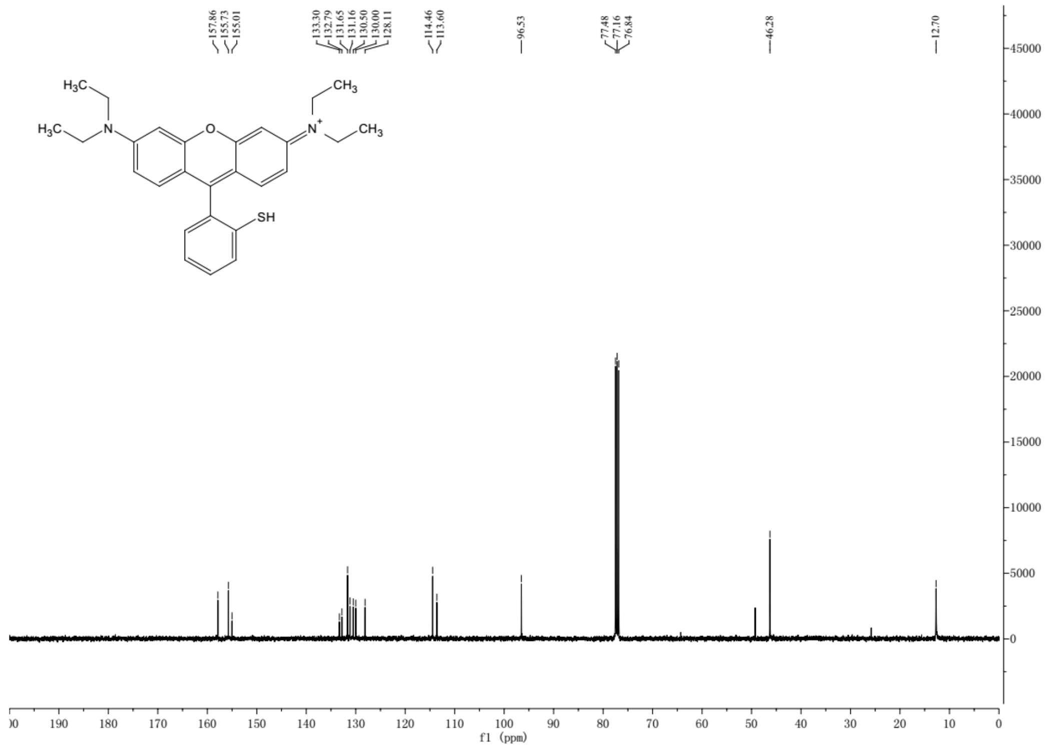

Supplementary Figure 186. The ^{13}C-NMR of compound **RD11** in $CDCl_3$

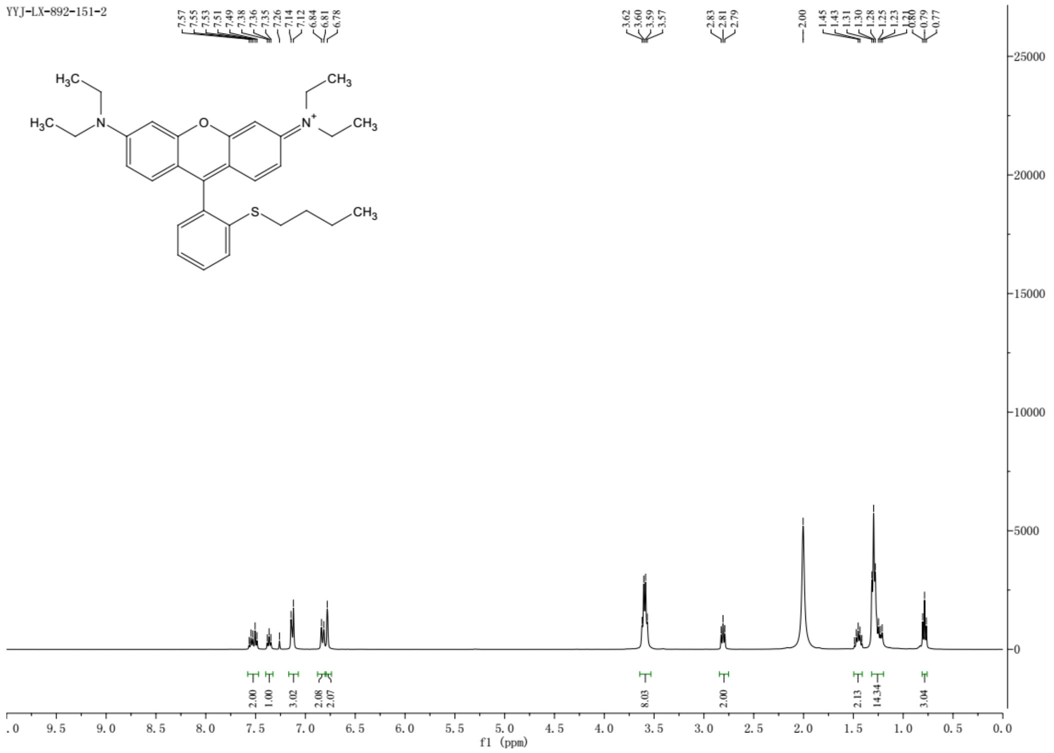

Supplementary Figure 187. The ^{1}H-NMR of compound **RD12** in $CDCl_3$

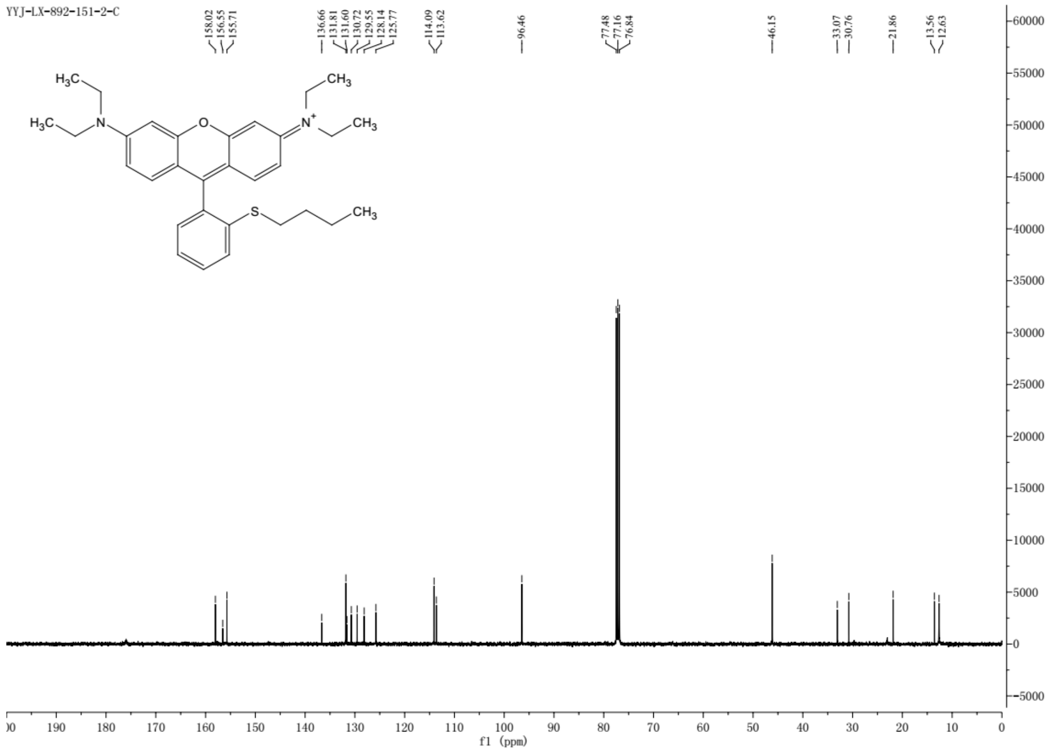

Supplementary Figure 188. The ^{13}C-NMR of compound **RD12** in $CDCl_3$

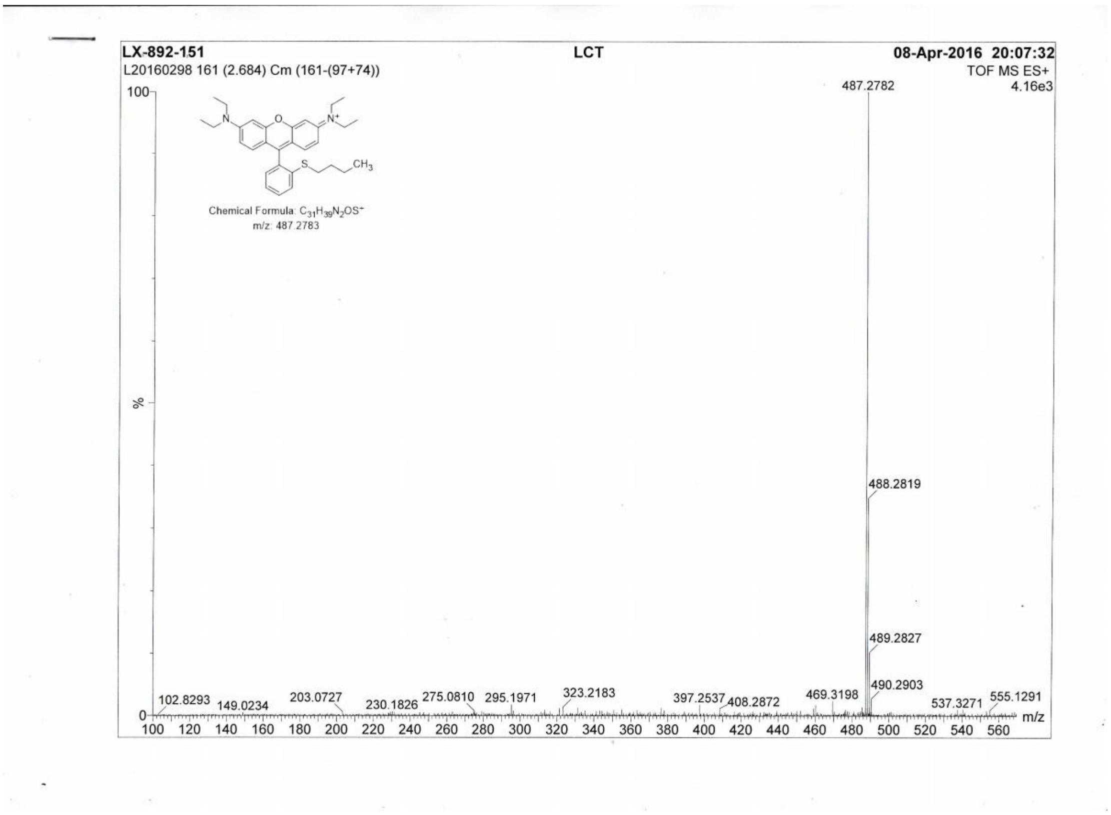

Supplementary Figure 189. The HR-MS of compound **RD12**

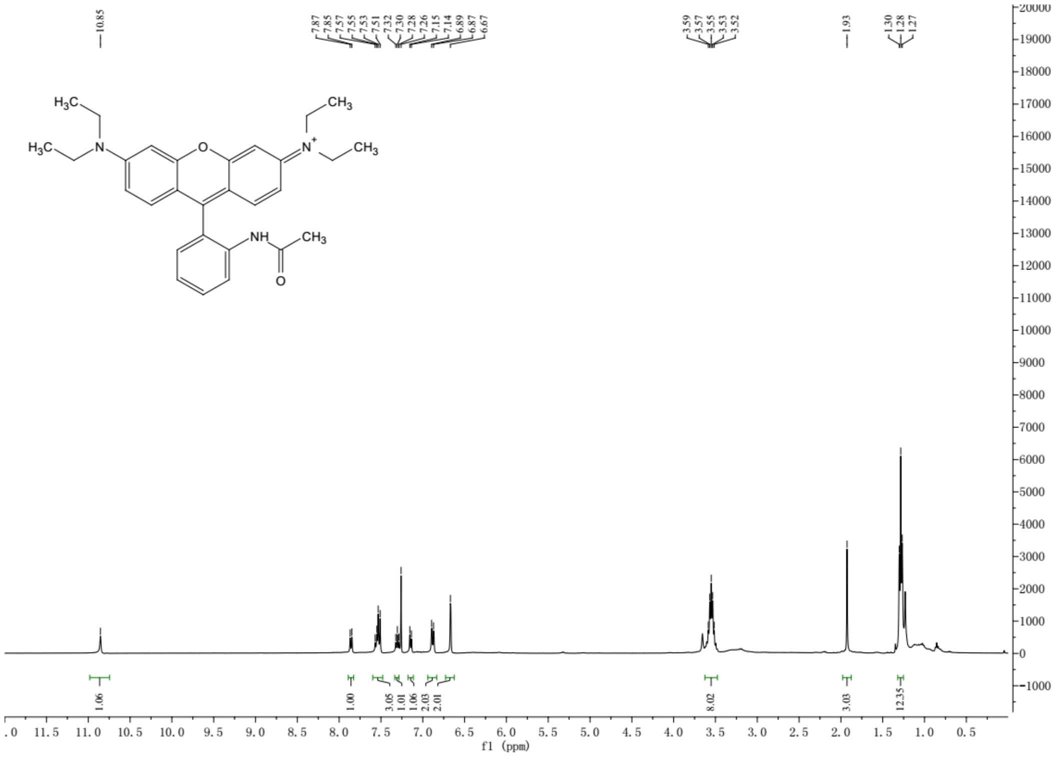

Supplementary Figure 190. The ^{1}H-NMR of compound **RD13** in $CDCl_3$

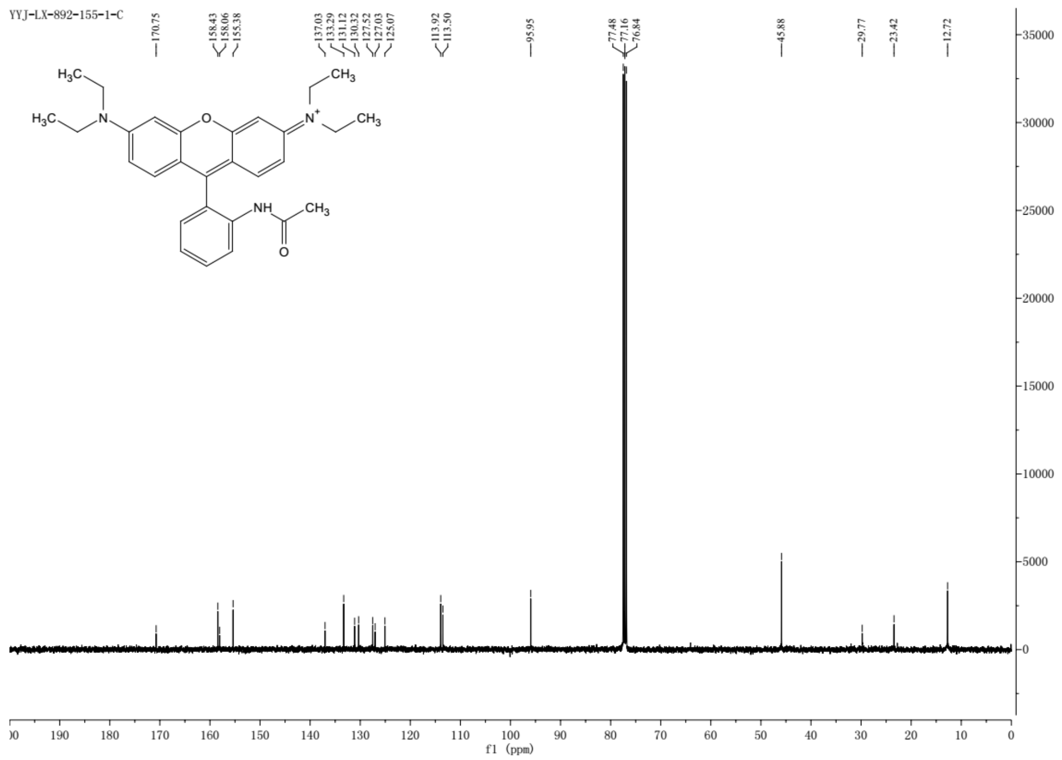

Supplementary Figure 191. The ^{13}C-NMR of compound **RD13** in $CDCl_3$

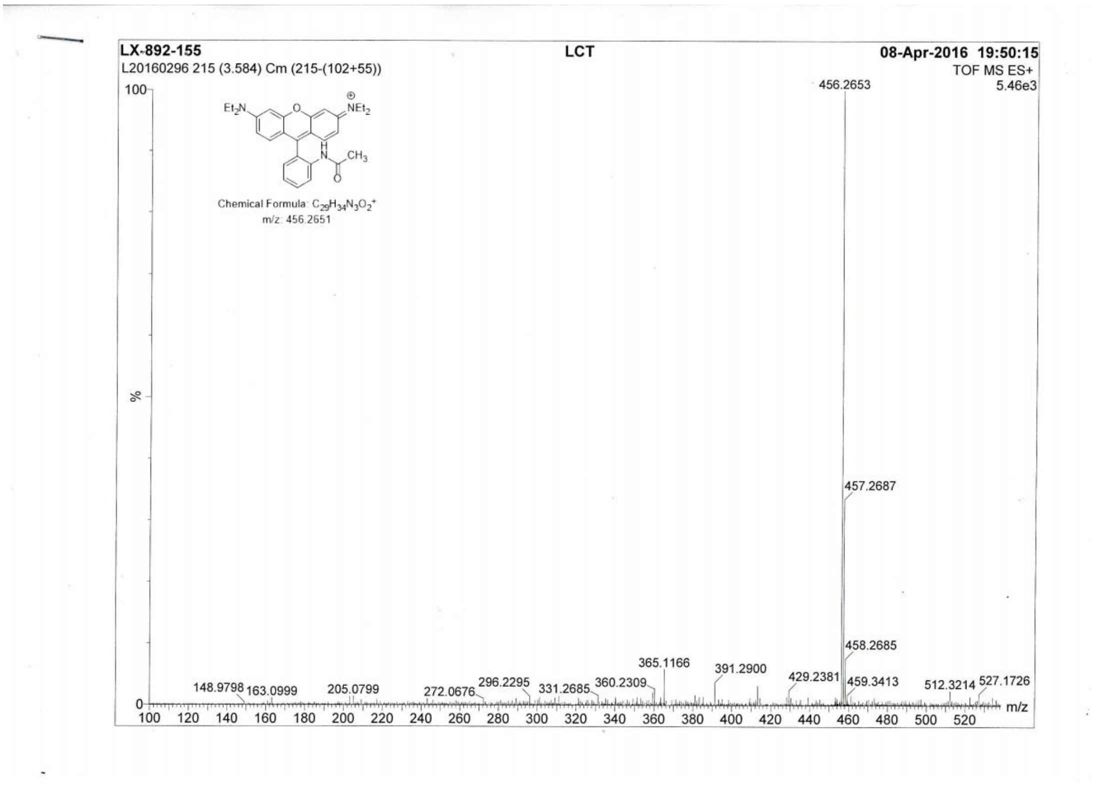

Supplementary Figure 192. The HR-MS of compound **RD13**

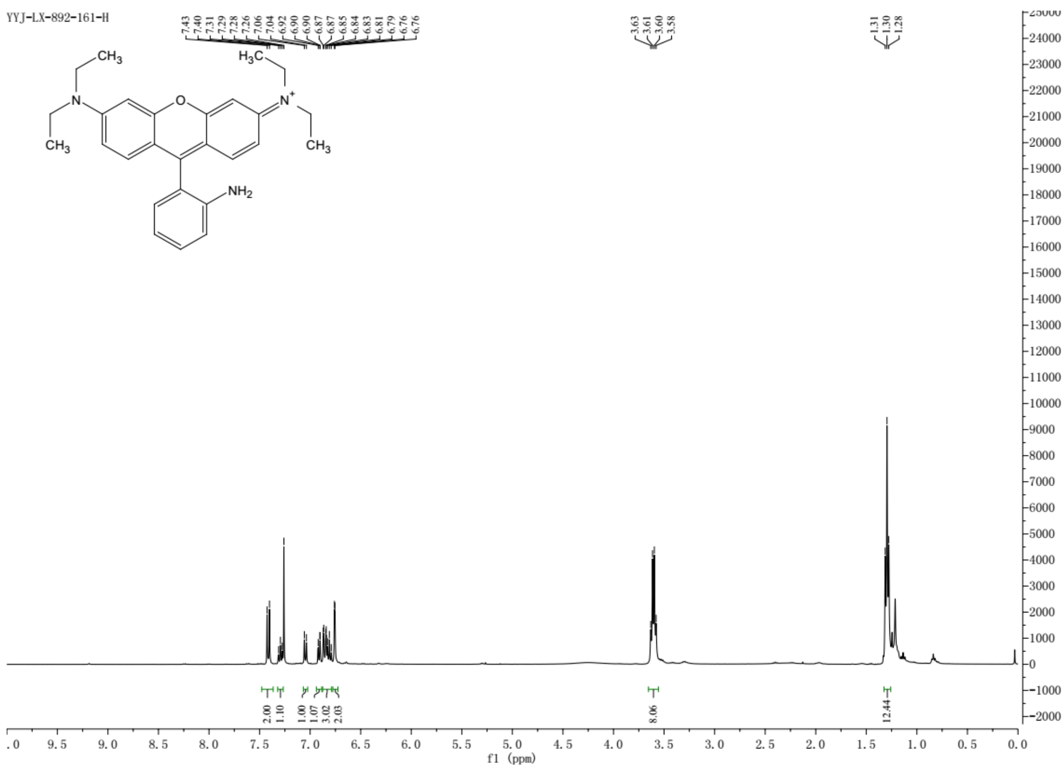

Supplementary Figure 193. The ^{1}H-NMR of compound **RD14** in $CDCl_3$

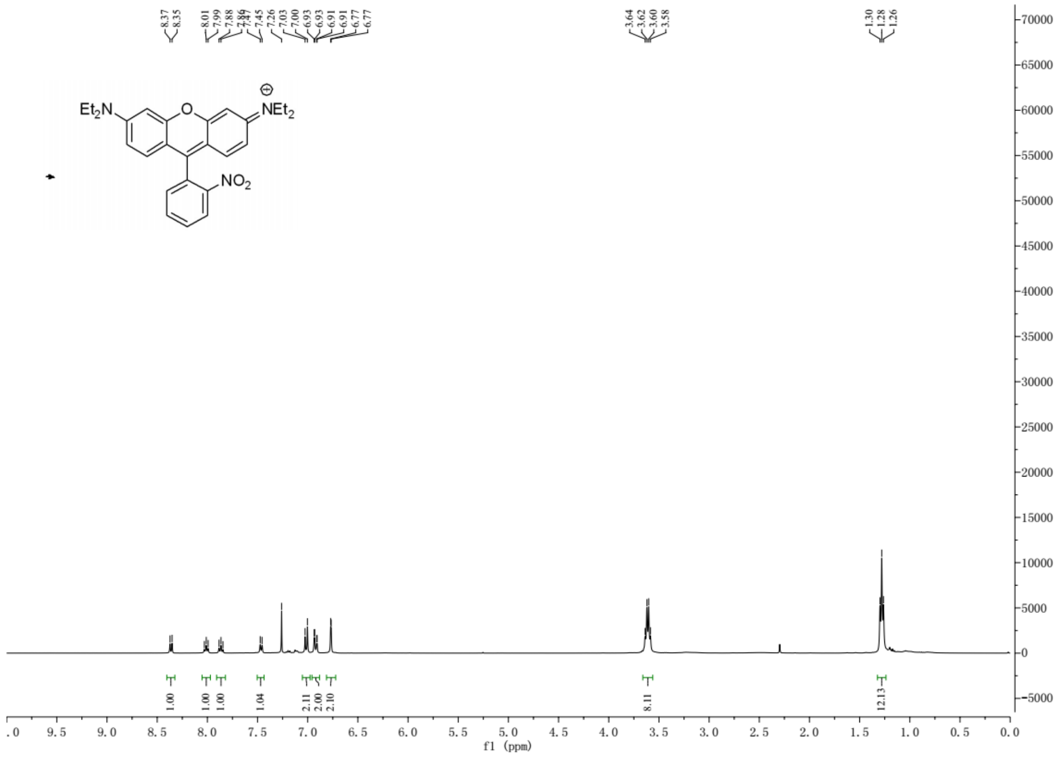

Supplementary Figure 194. The ^{1}H-NMR of compound **RD15** in $CDCl_3$

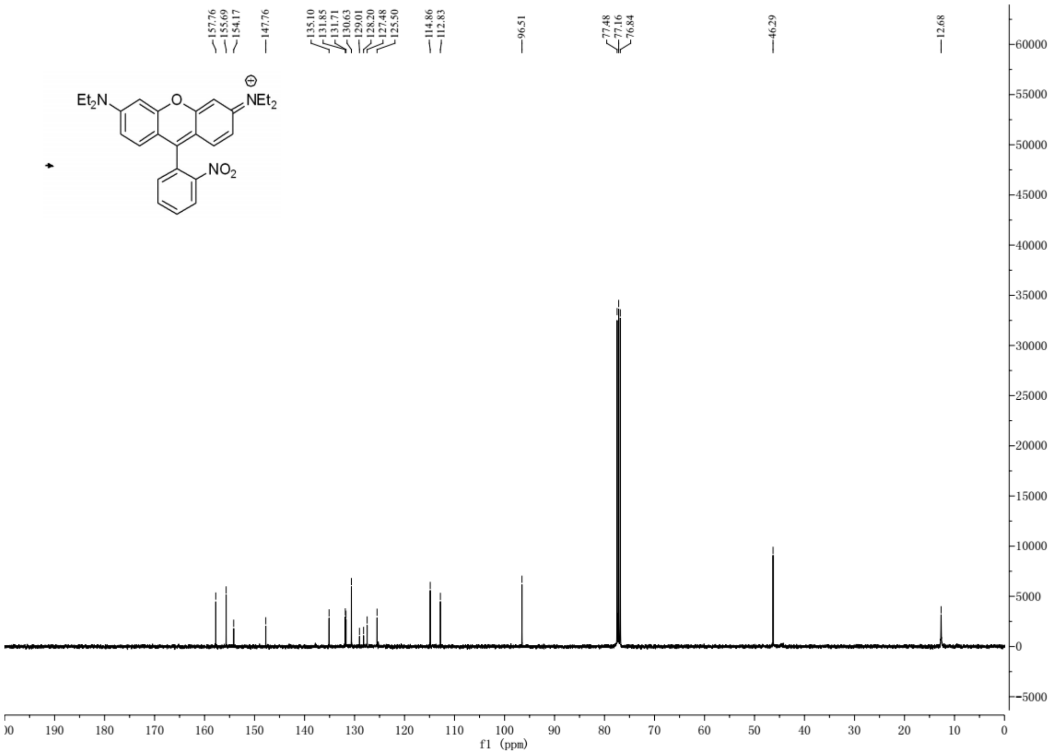

Supplementary Figure 195. The ^{13}C-NMR of compound **RD15** in $CDCl_3$

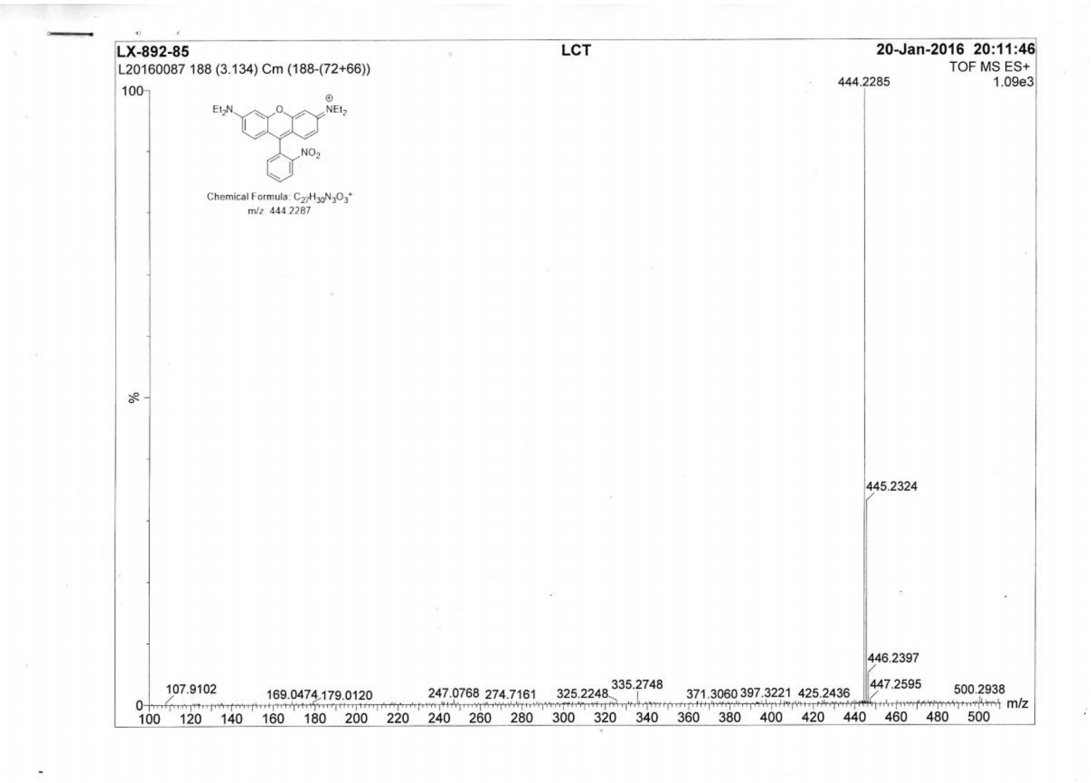

Supplementary Figure 196. The HR-MS of compound **RD15**

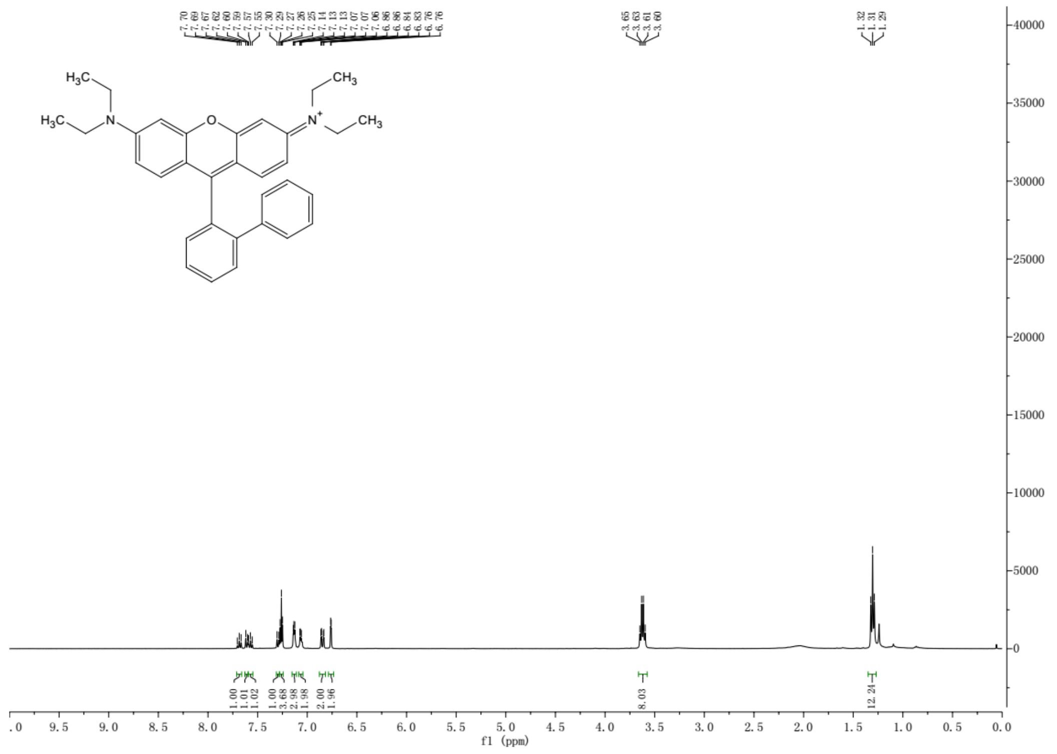

Supplementary Figure 197. The ^{1}H-NMR of compound **RD16** in $CDCl_3$

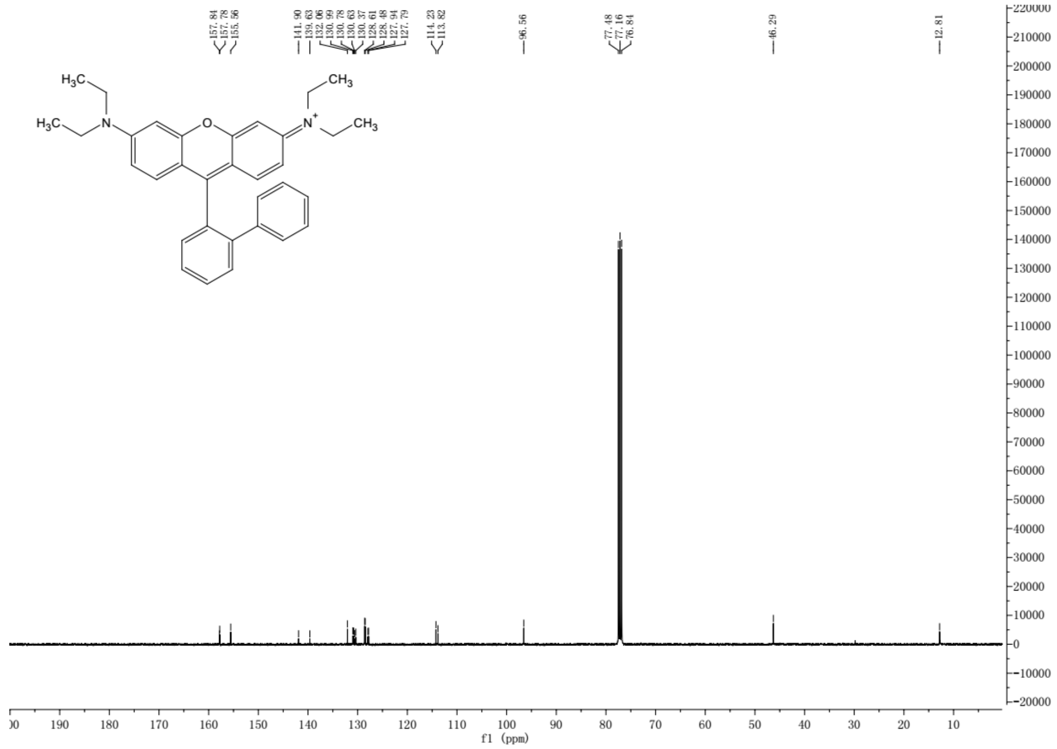

Supplementary Figure 198. The ^{13}C-NMR of compound **RD16** in $CDCl_3$

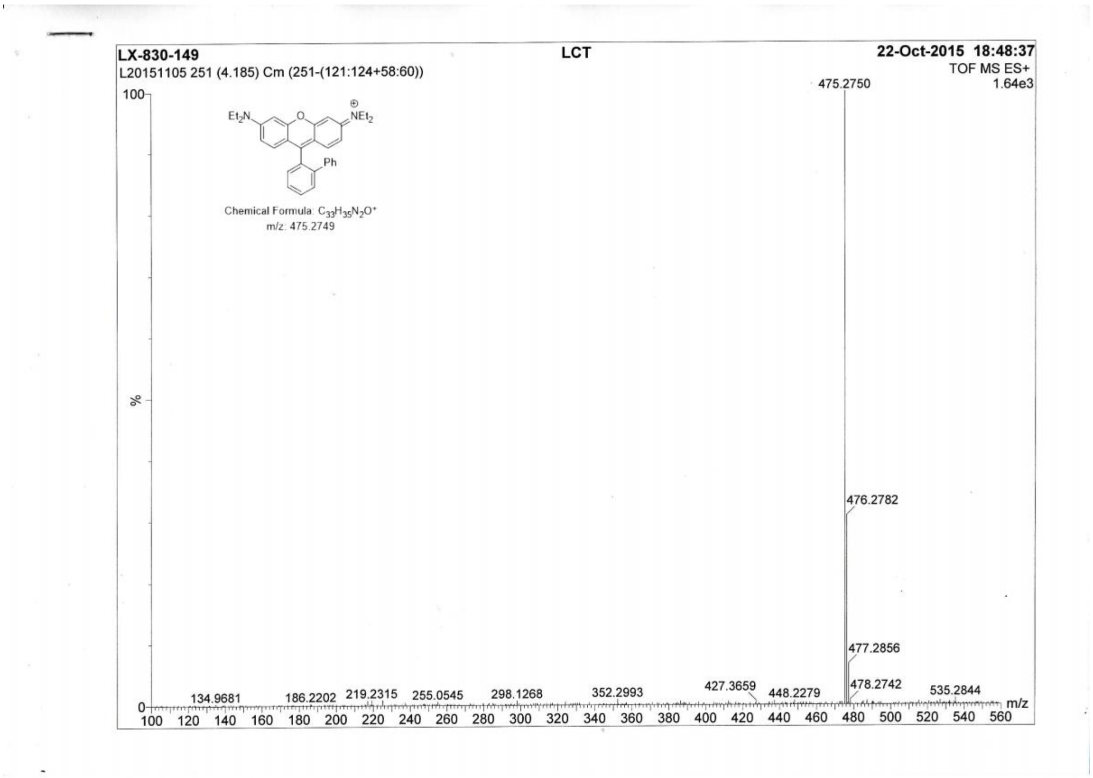

Supplementary Figure 199. The HR-MS of compound **RD16**

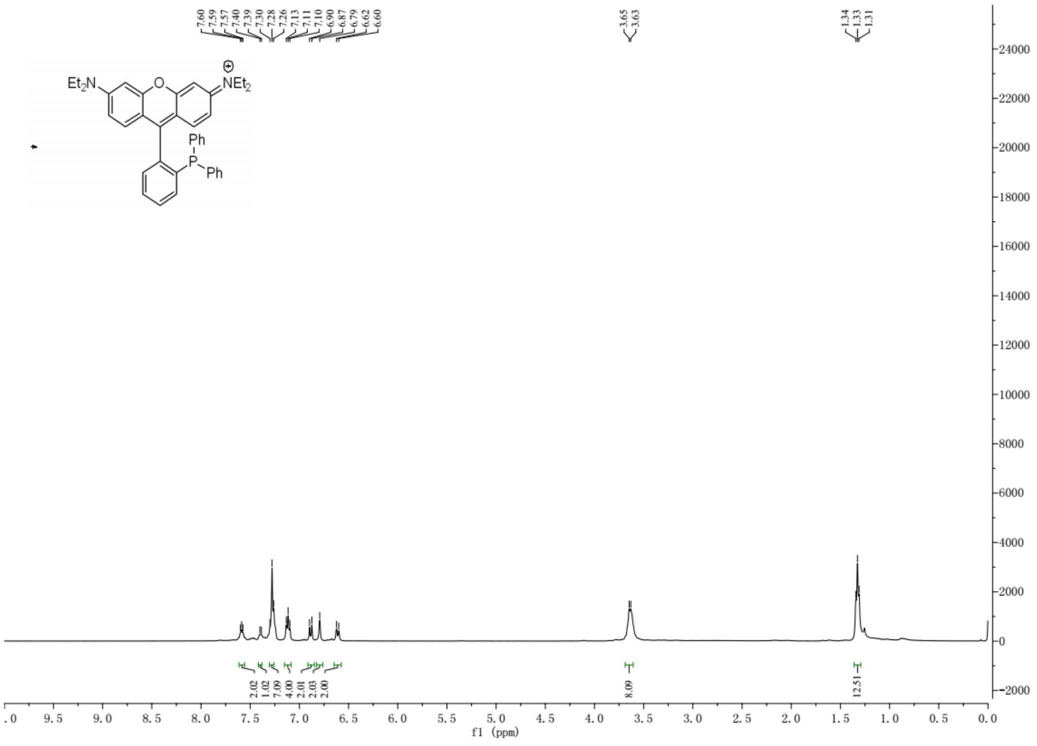

Supplementary Figure 200. The ^{1}H-NMR of compound **RD17** in $CDCl_3$

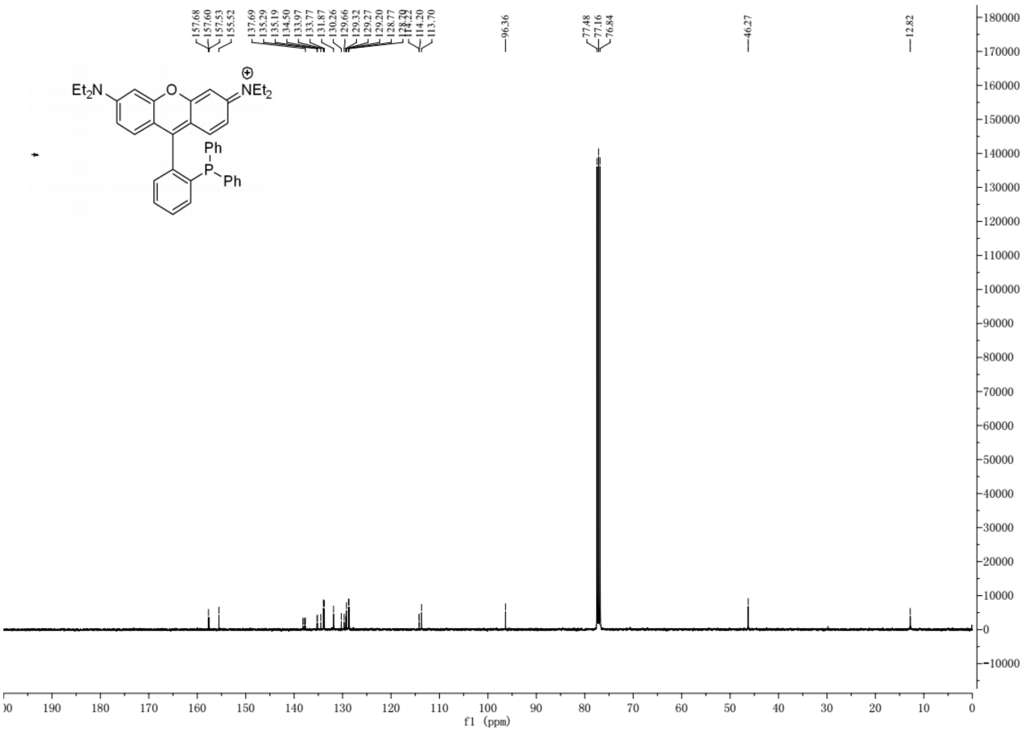

Supplementary Figure 201. The ^{13}C-NMR of compound **RD17** in $CDCl_3$

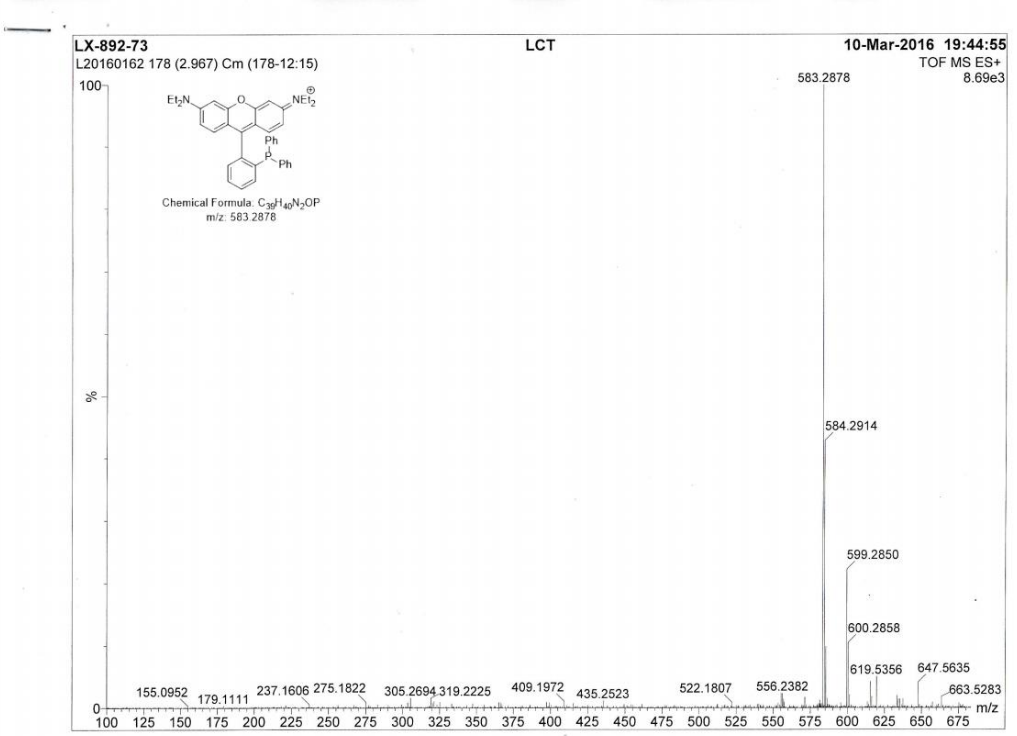

Supplementary Figure 202. The HR-MS of compound **RD17**

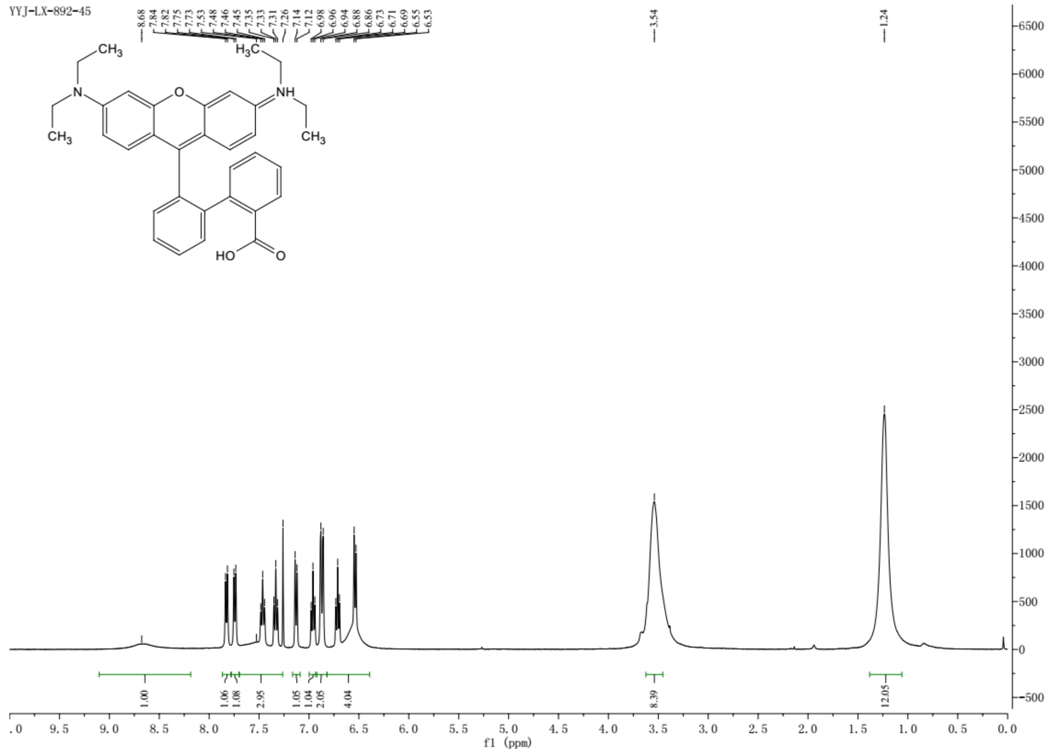

Supplementary Figure 203. The ^{1}H-NMR of compound **RD18** in $CDCl_3$

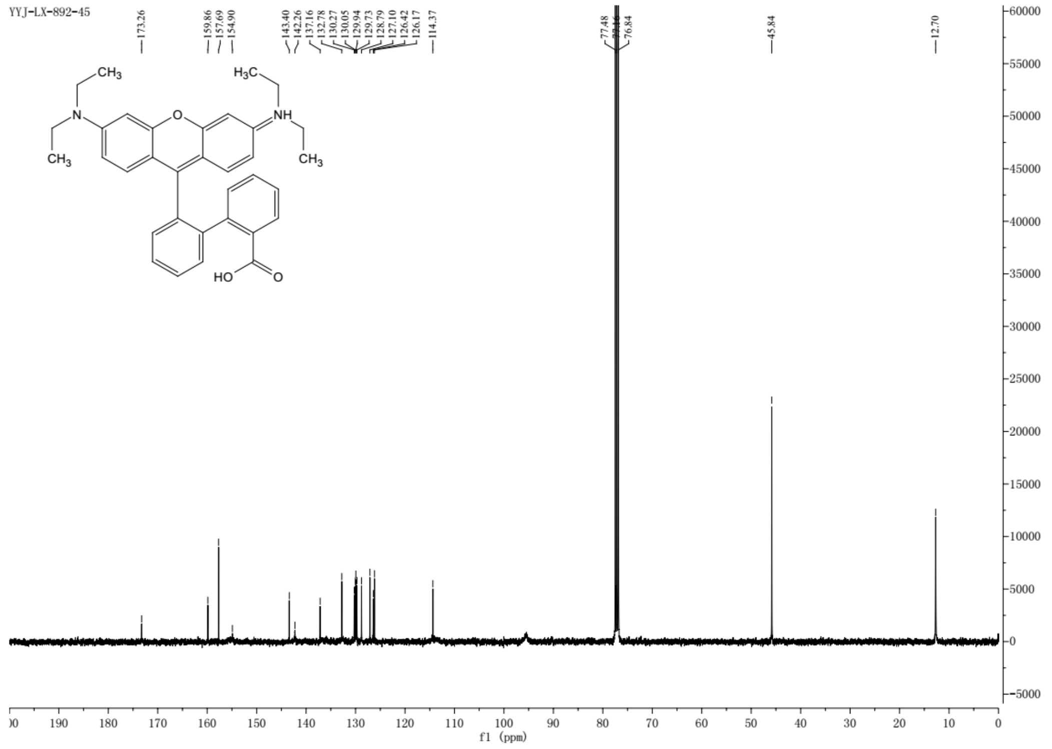

Supplementary Figure 204. The ^{13}C-NMR of compound **RD18** in $CDCl_3$

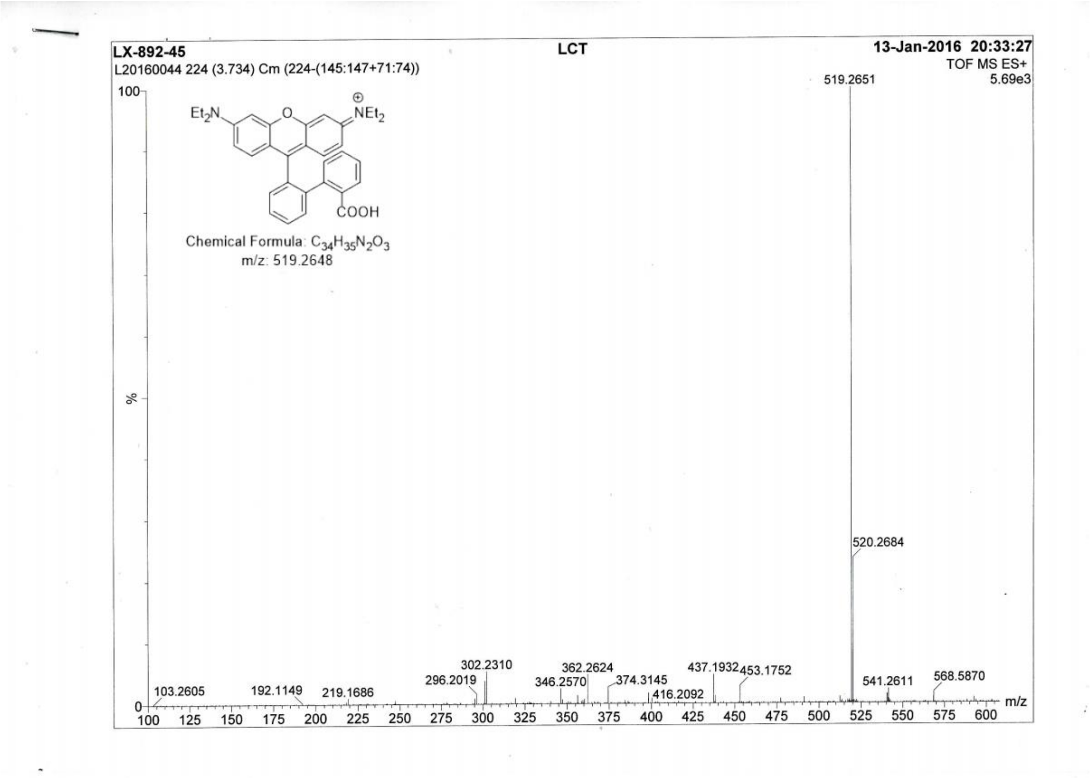

Supplementary Figure 205. The HR-MS of compound **RD18**

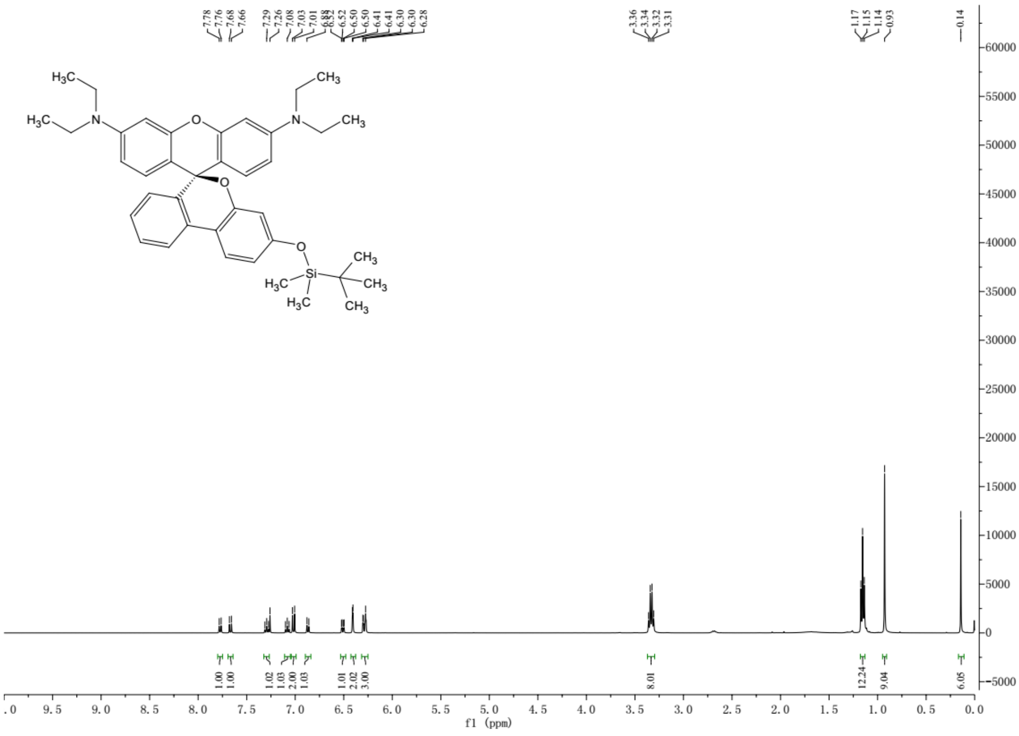

Supplementary Figure 206. The ^{1}H-NMR of compound **RD19** in $CDCl_3$

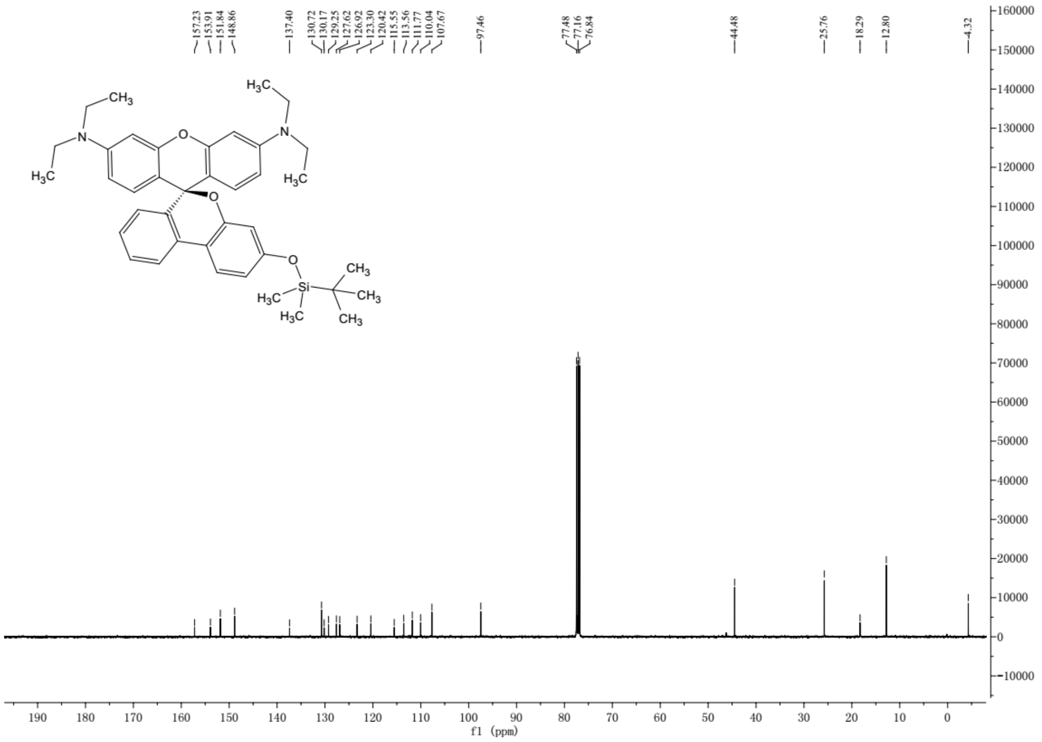

Supplementary Figure 207. The ^{13}C-NMR of compound **RD19** in $CDCl_3$

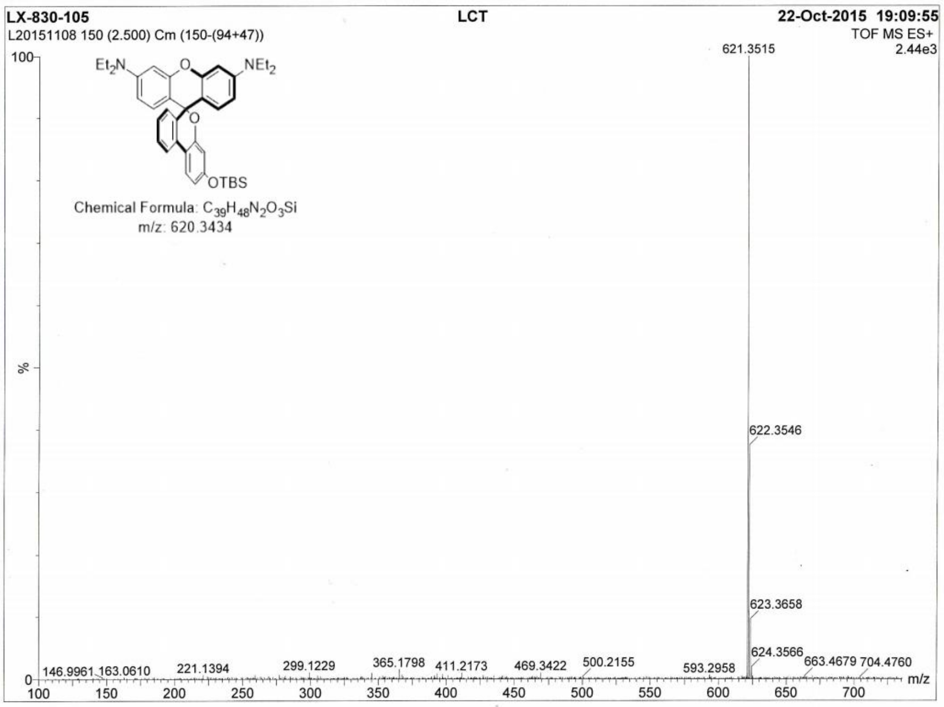

Supplementary Figure 208. The HR-MS of compound **RD19**

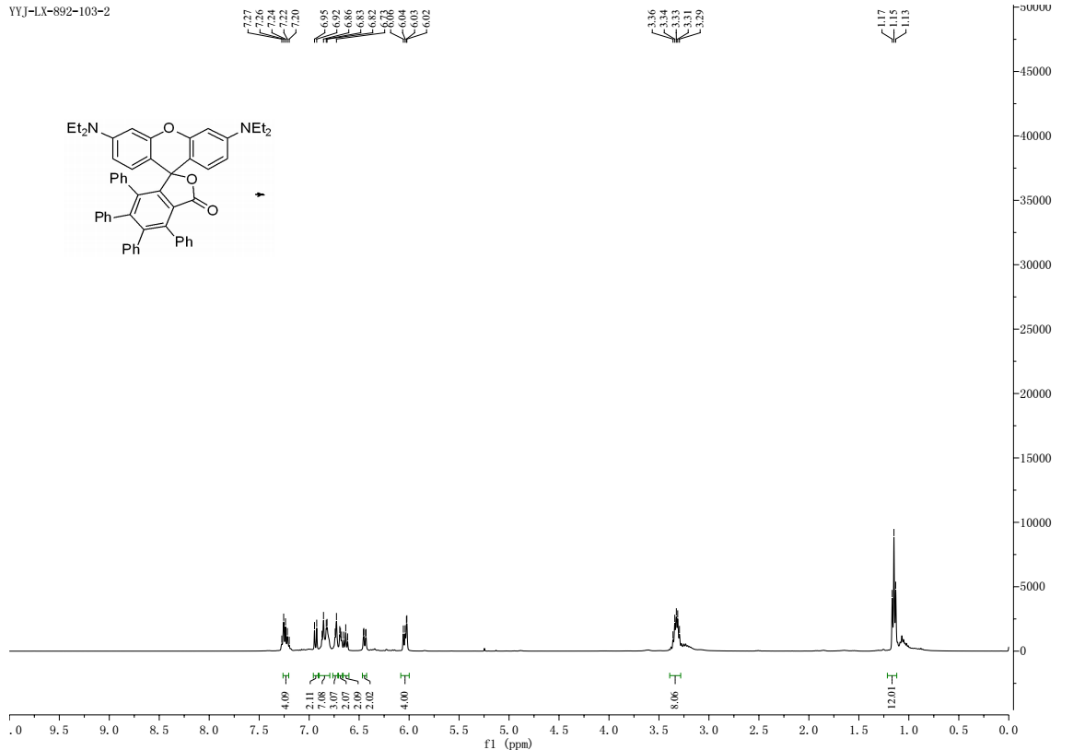

Supplementary Figure 209. The ^{1}H-NMR of compound **RD20** in $CDCl_3$

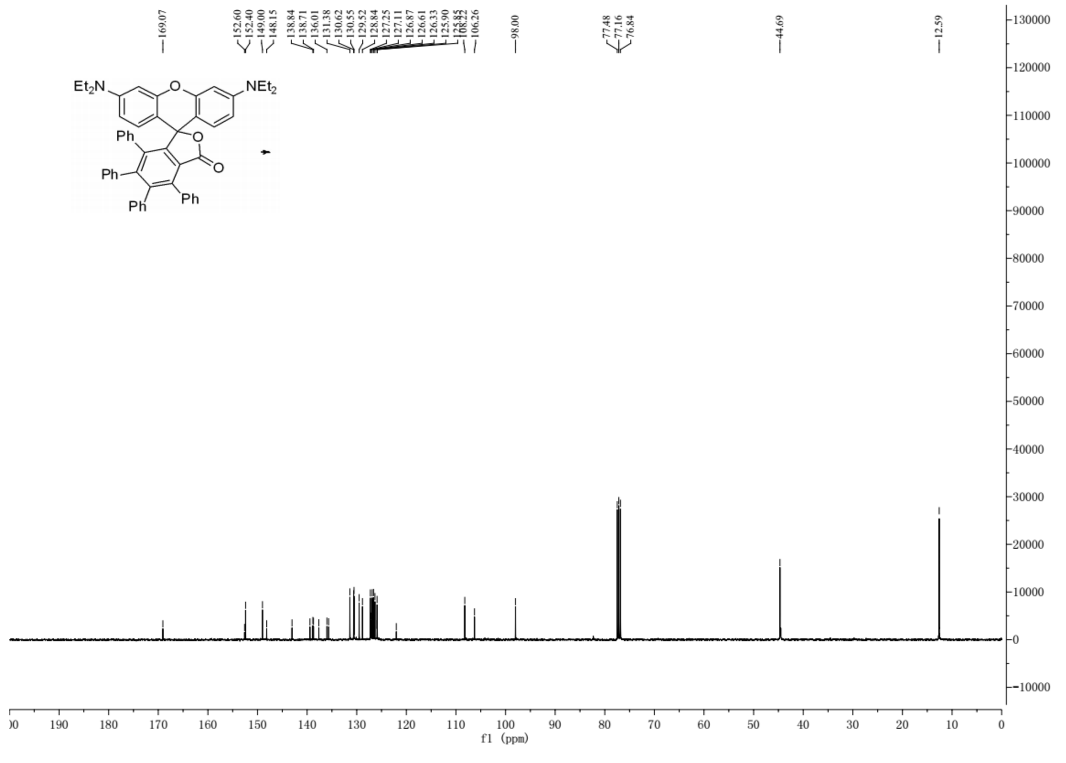

Supplementary Figure 210. The ^{13}C-NMR of compound **RD20** in $CDCl_3$

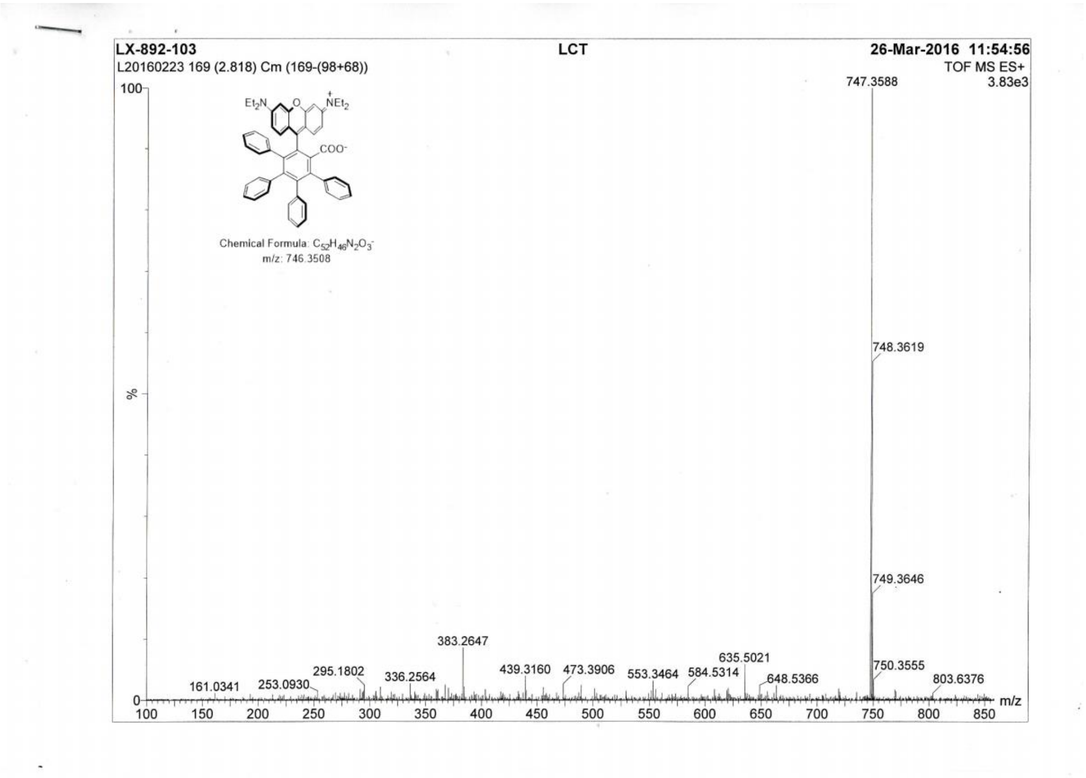

Supplementary Figure 211. The HR-MS of compound **RD20**

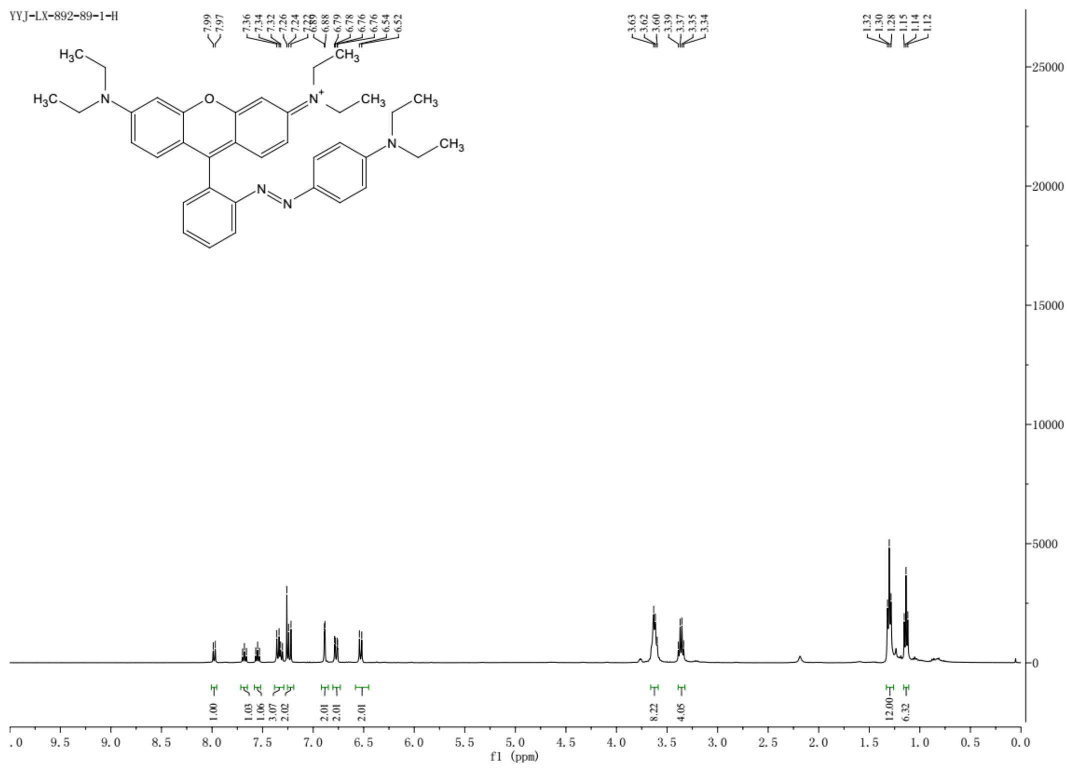

Supplementary Figure 212. The ^{1}H-NMR of compound **RD21** in $CDCl_3$

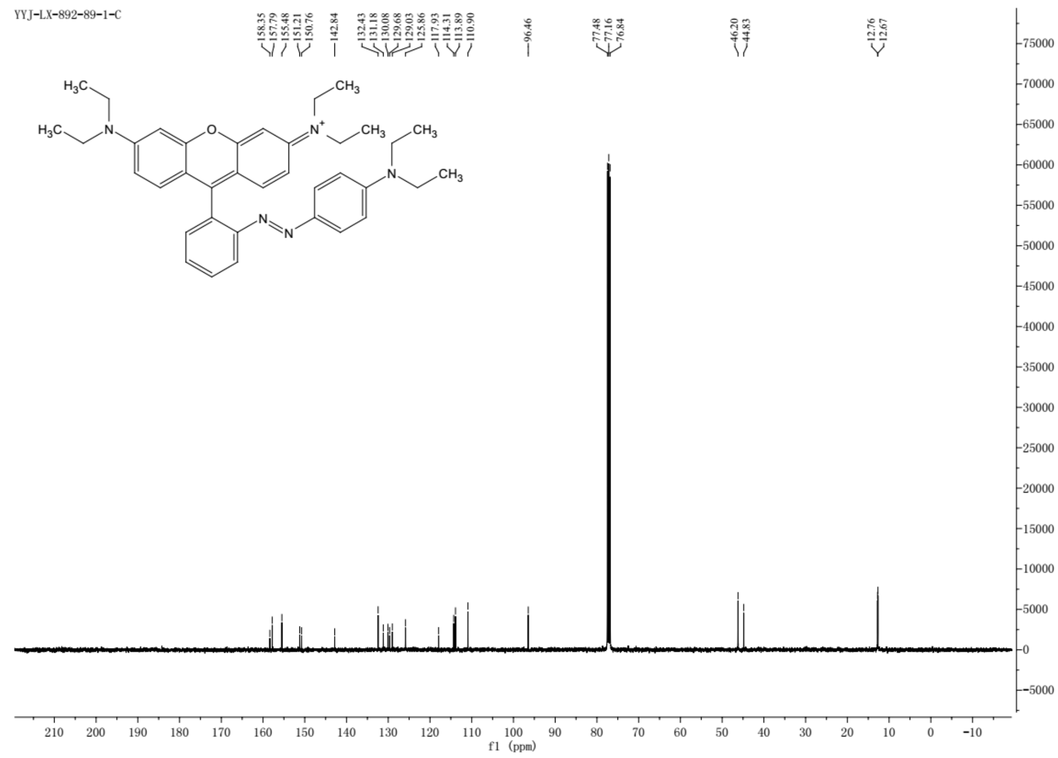

Supplementary Figure 213. The ^{13}C-NMR of compound **RD21** in $CDCl_3$

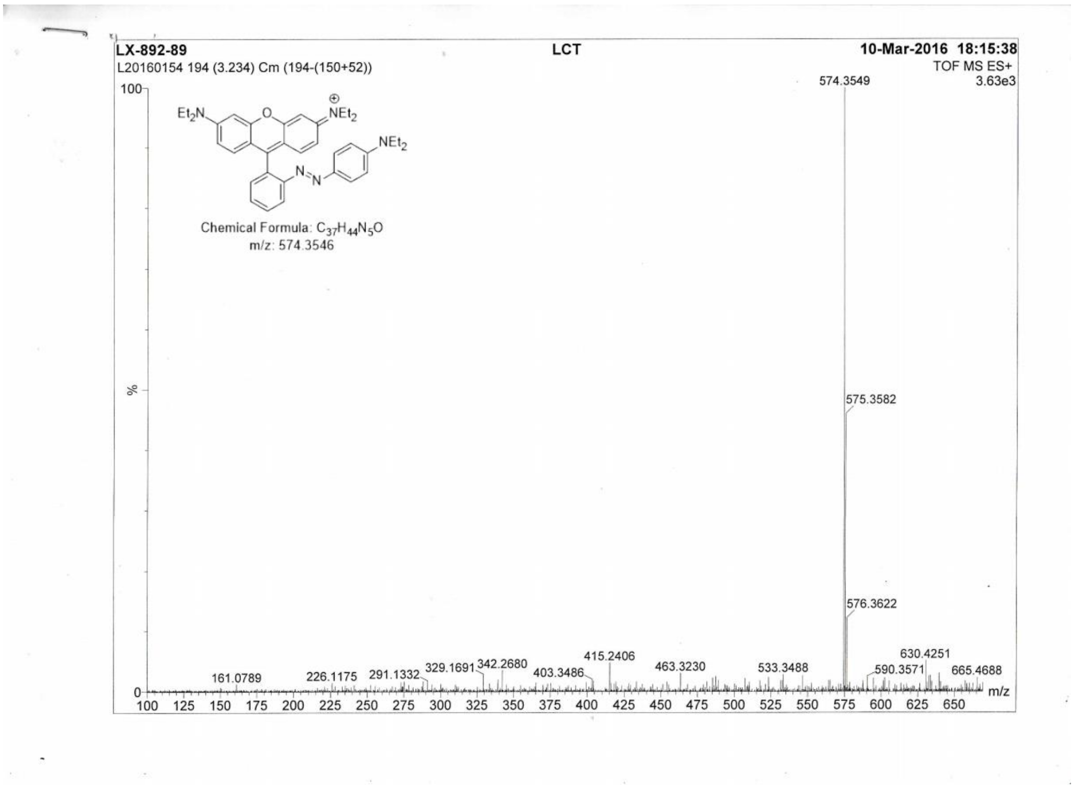

Supplementary Figure 214. The HR-MS of compound **RD21**

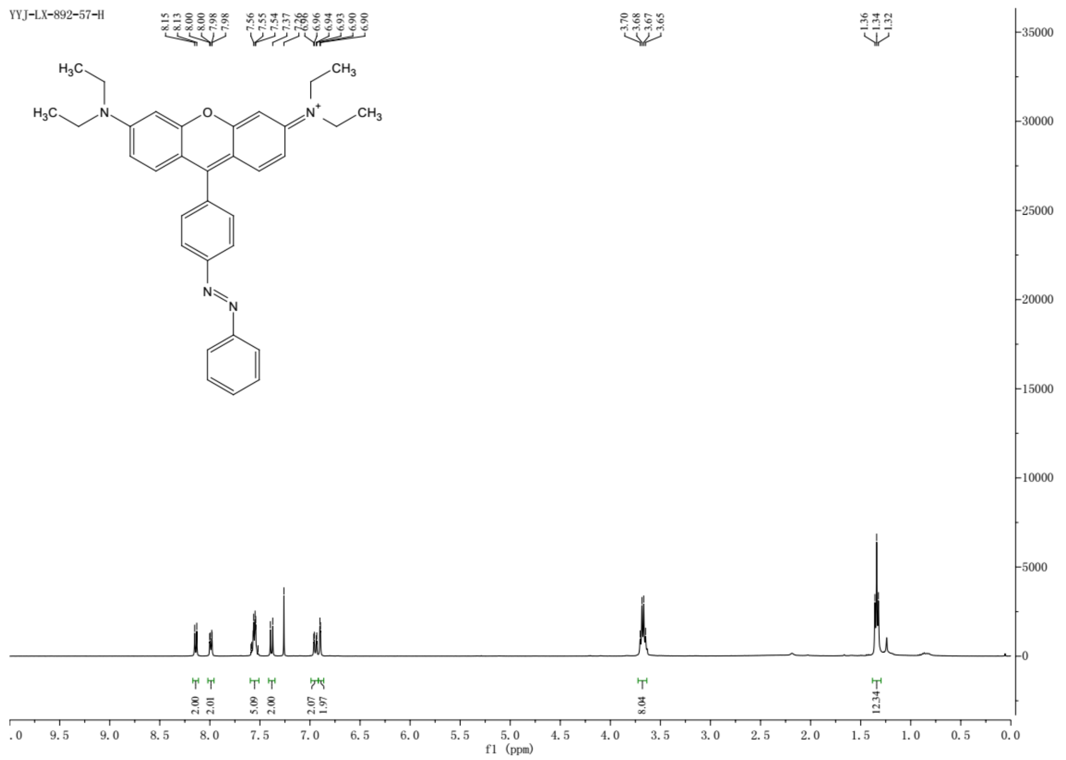

Supplementary Figure 215. The ^{1}H-NMR of compound **RD22** in $CDCl_3$

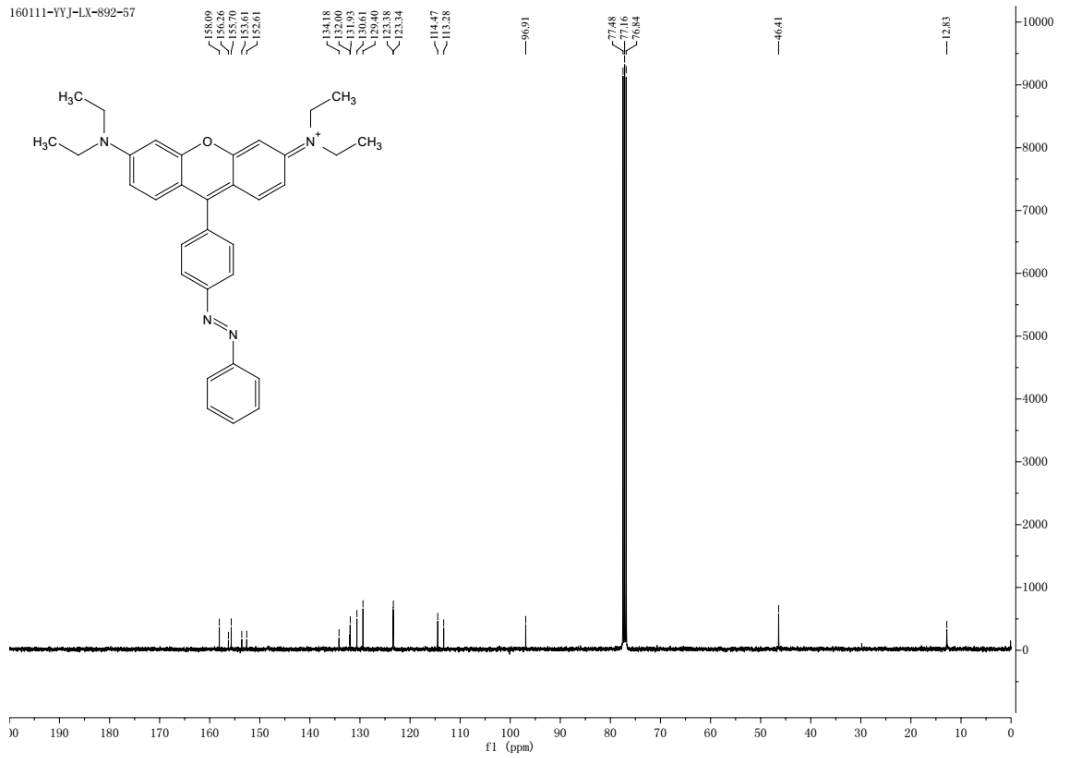

Supplementary Figure 216. The ^{13}C-NMR of compound **RD22** in $CDCl_3$

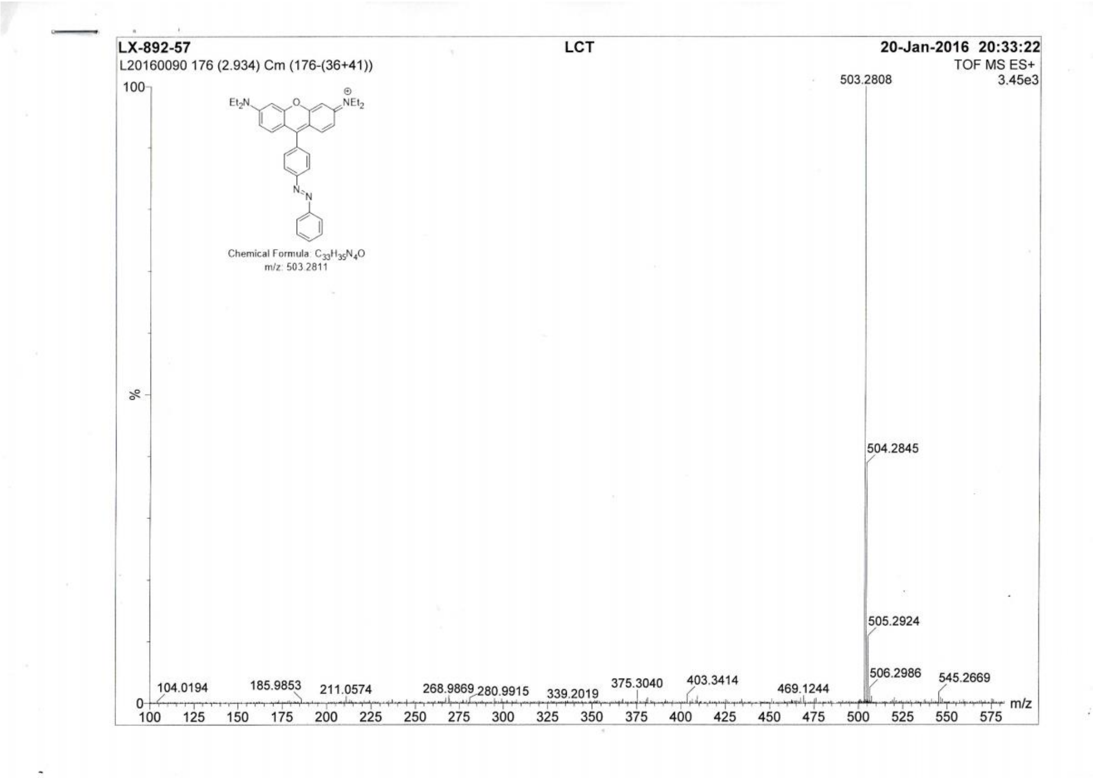

Supplementary Figure 217. The HR-MS of compound **RD22**

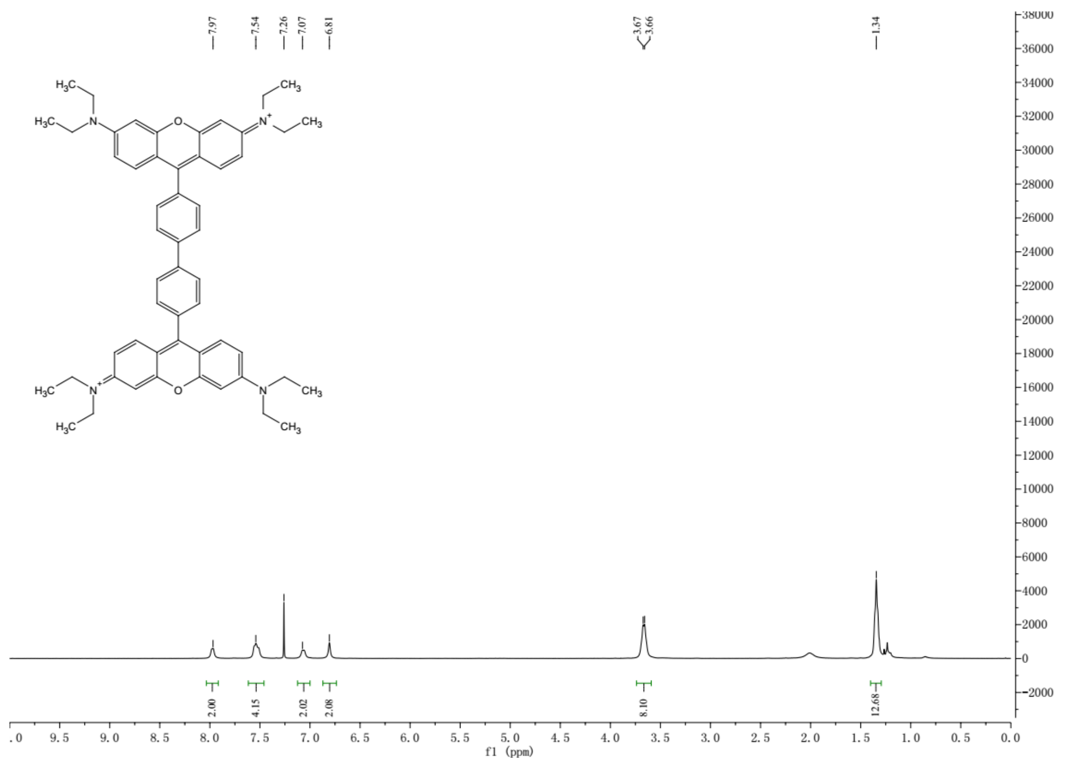

Supplementary Figure 218. The ^{1}H-NMR of compound **RD23** in $CDCl_3$

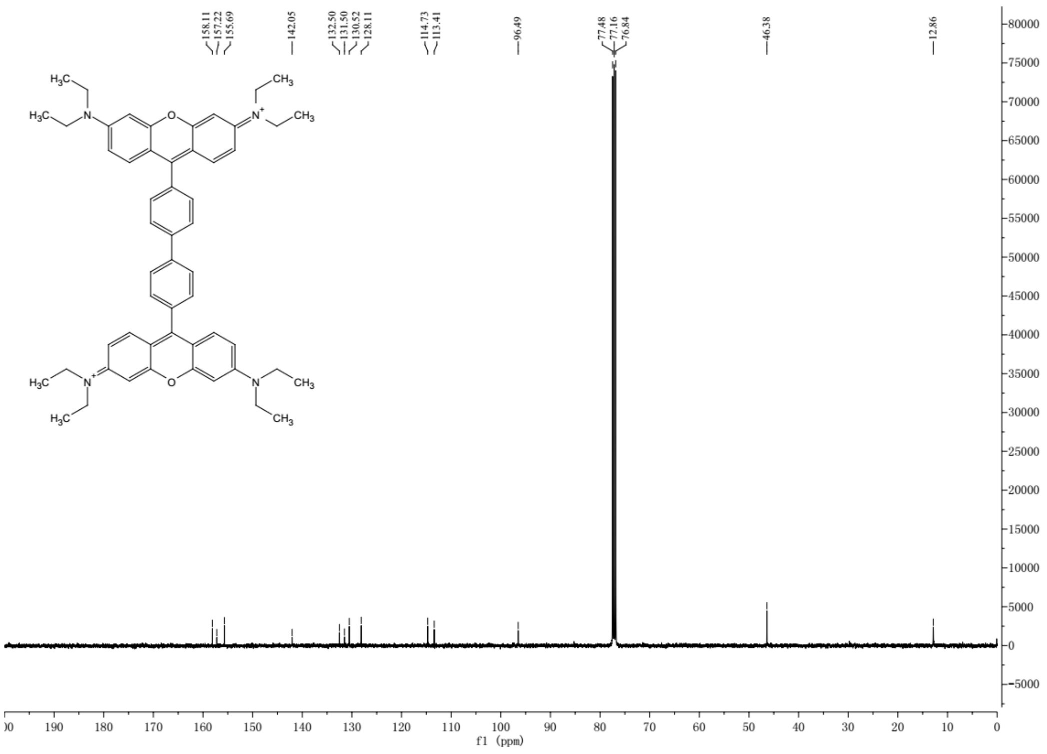

Supplementary Figure 219. The ^{13}C-NMR of compound **RD23** in $CDCl_3$

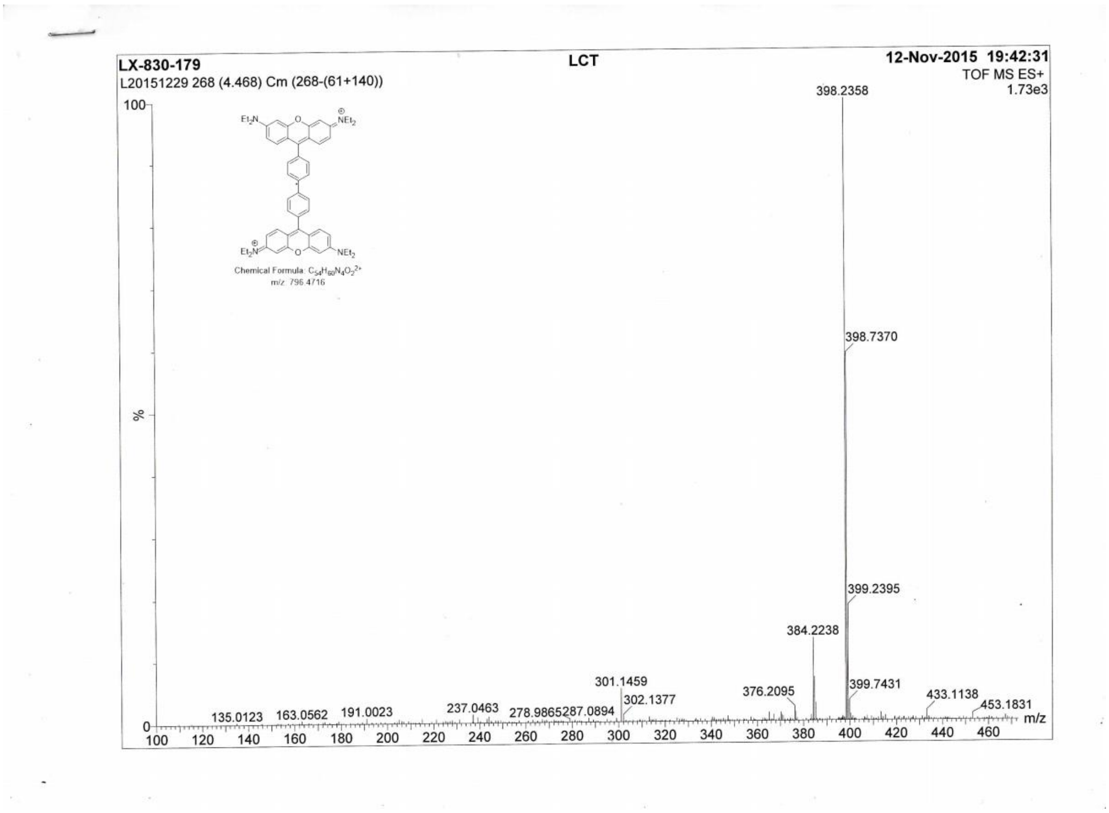

Supplementary Figure 220. The HR-MS of compound **RD23**

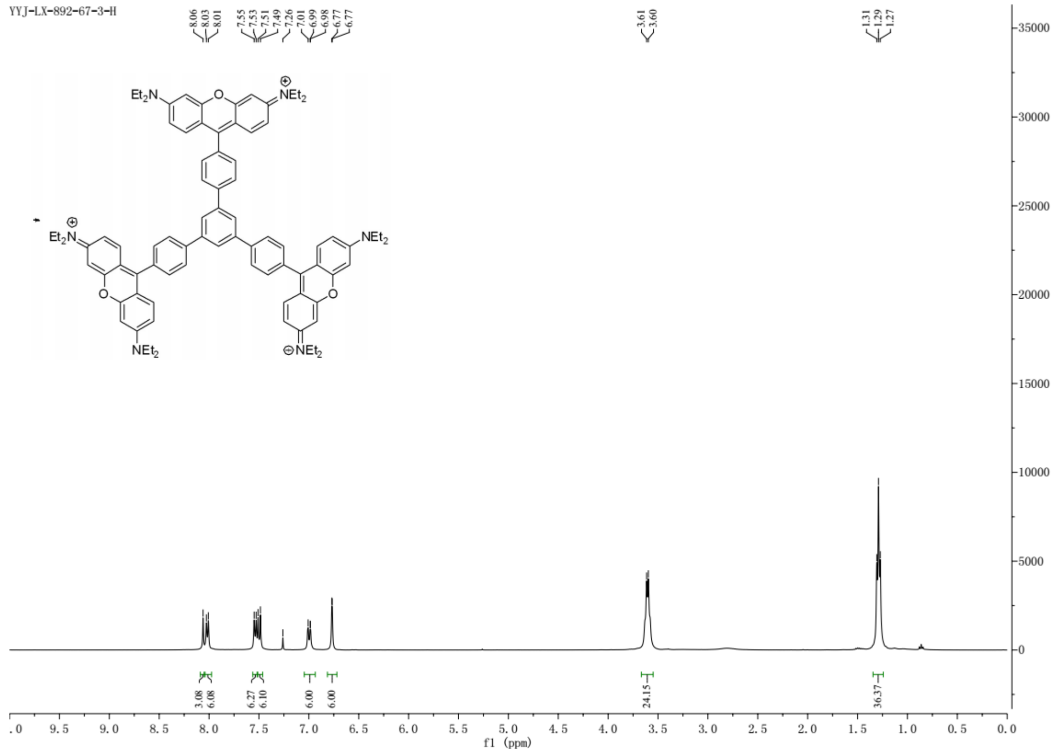

Supplementary Figure 221. The ^{1}H-NMR of compound **RD24** in $CDCl_3$

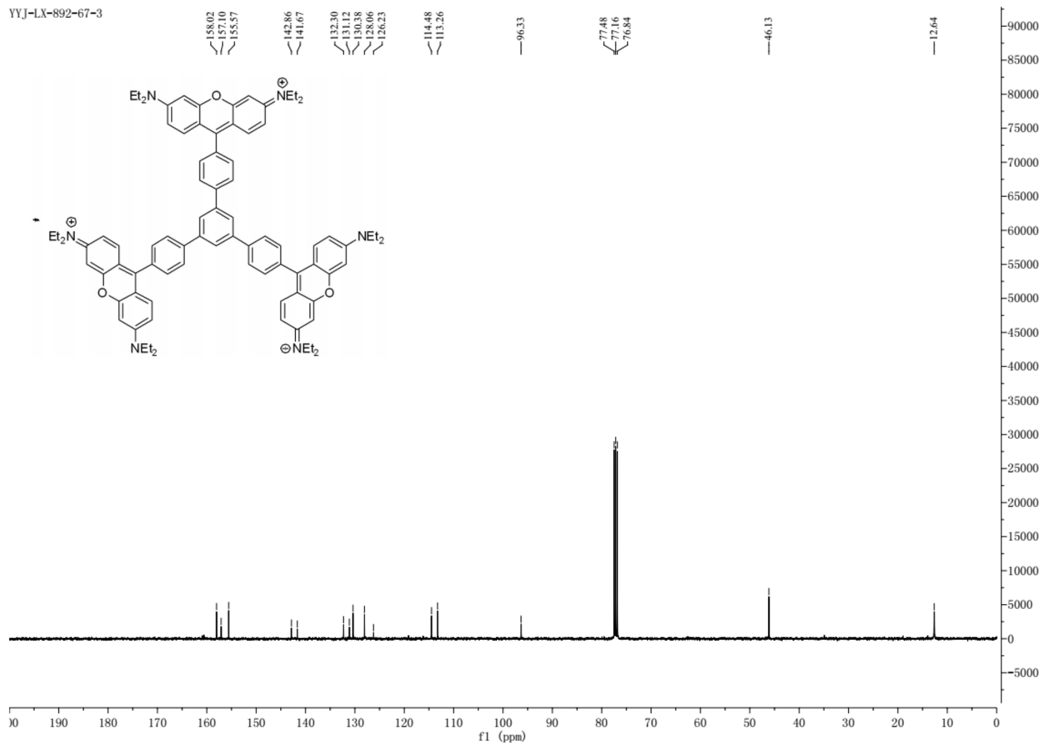

Supplementary Figure 222. The ^{13}C-NMR of compound **RD24** in $CDCl_3$

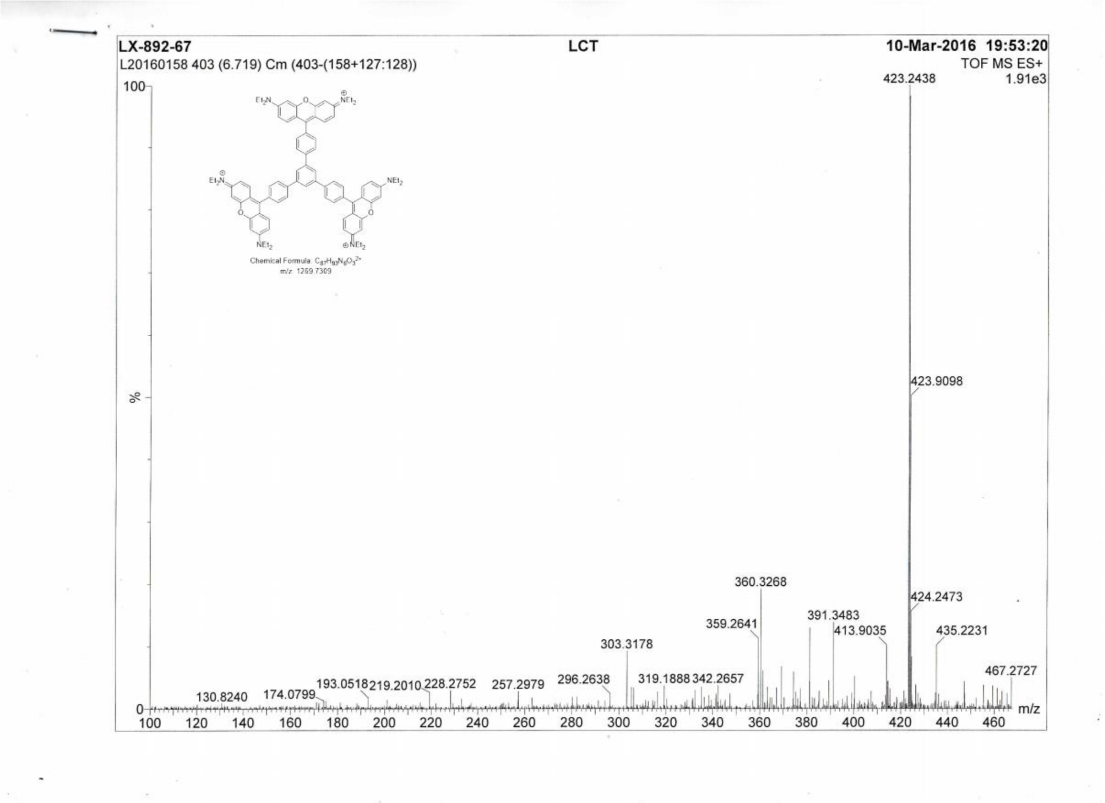

Supplementary Figure 223. The HR-MS of compound **RD24**

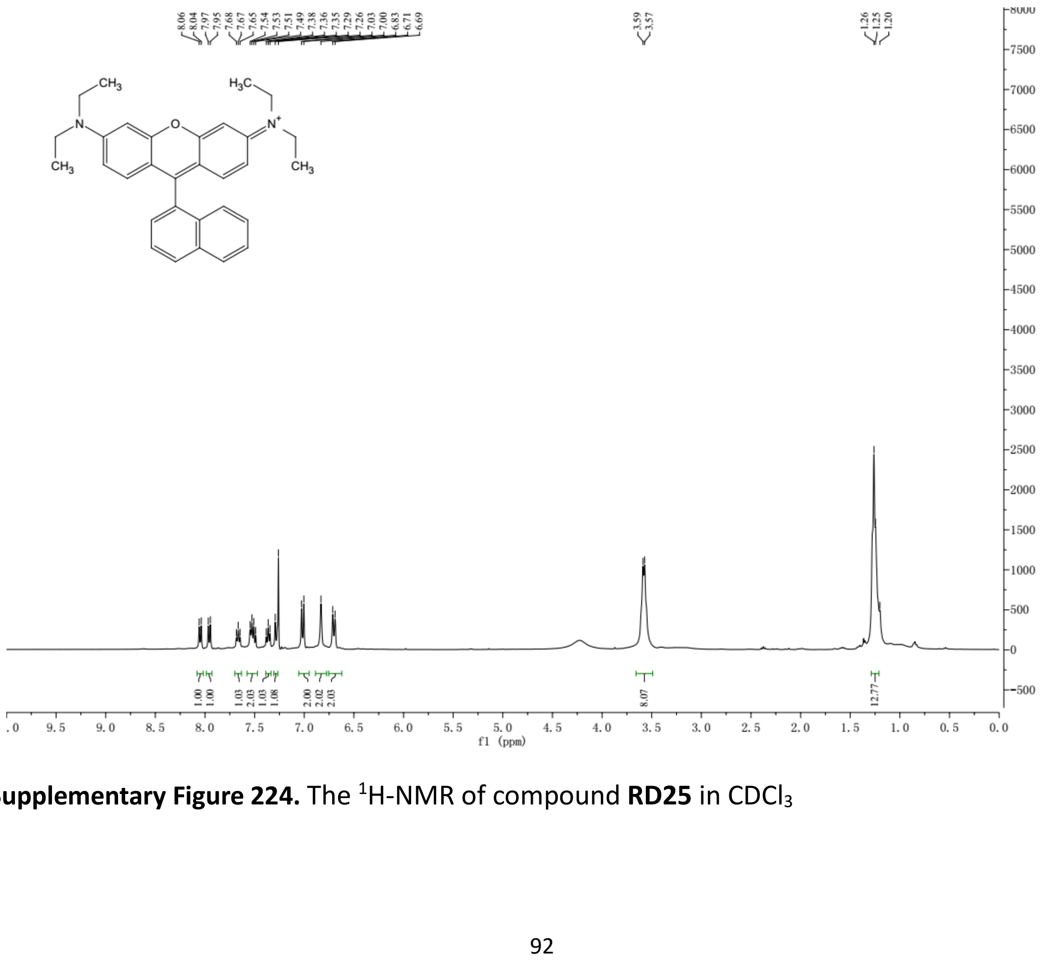

Supplementary Figure 224. The ^{1}H-NMR of compound **RD25** in $CDCl_3$

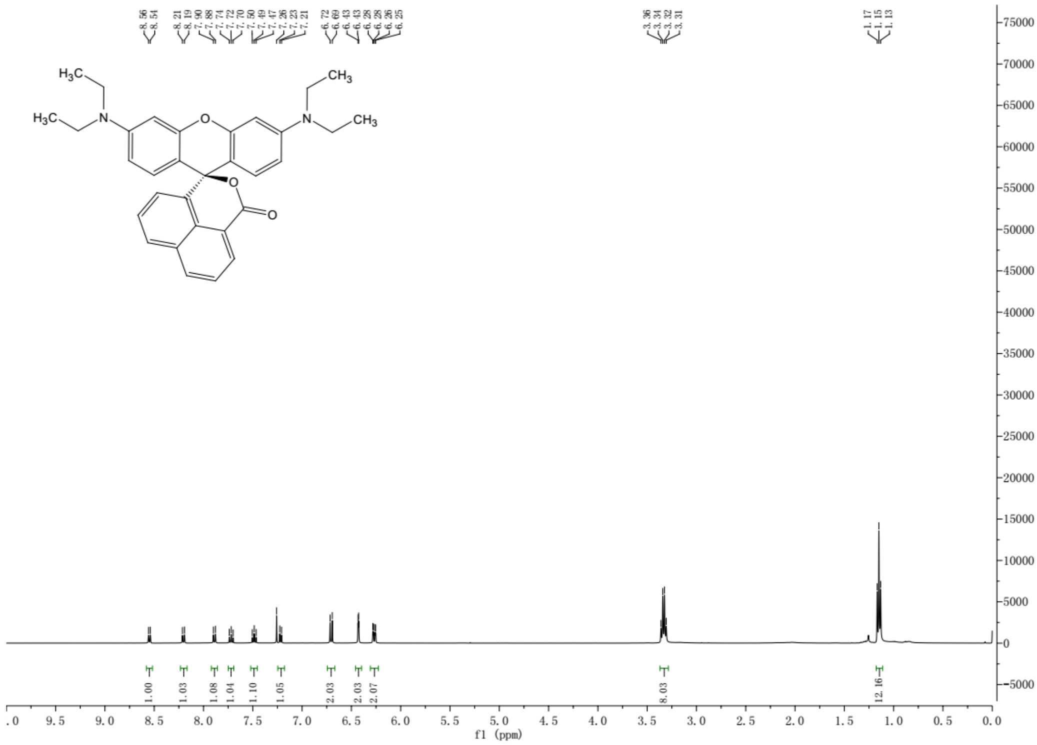

Supplementary Figure 225. The ^{1}H-NMR of compound **RD26** in $CDCl_3$

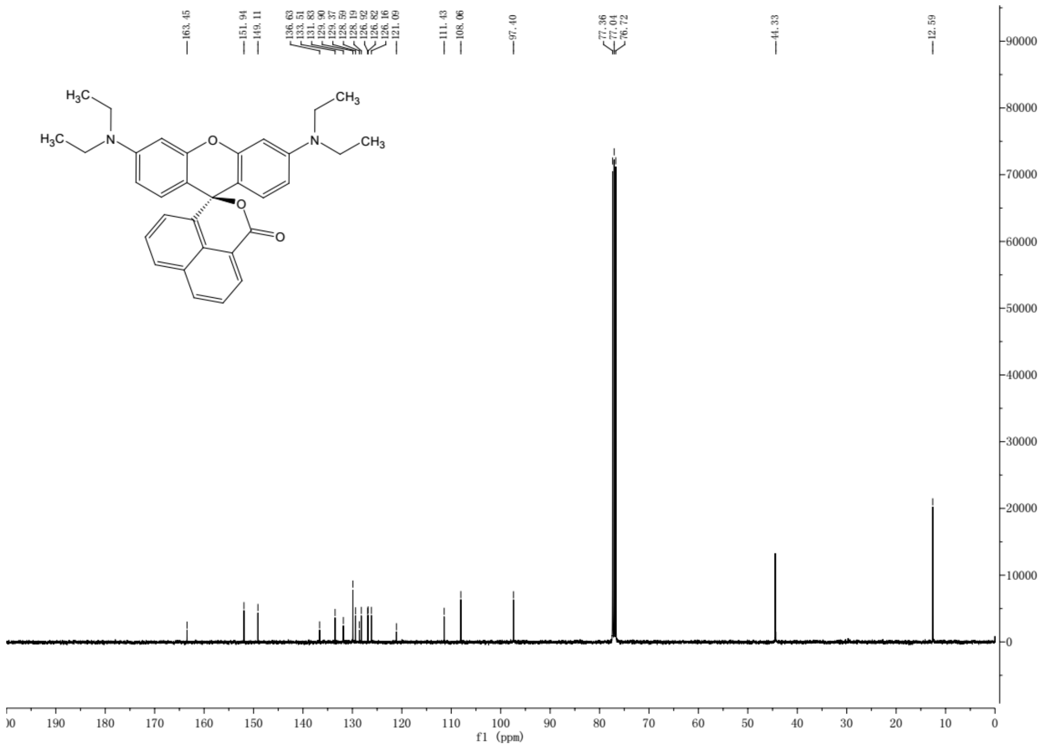

Supplementary Figure 226. The ^{13}C-NMR of compound **RD26** in $CDCl_3$

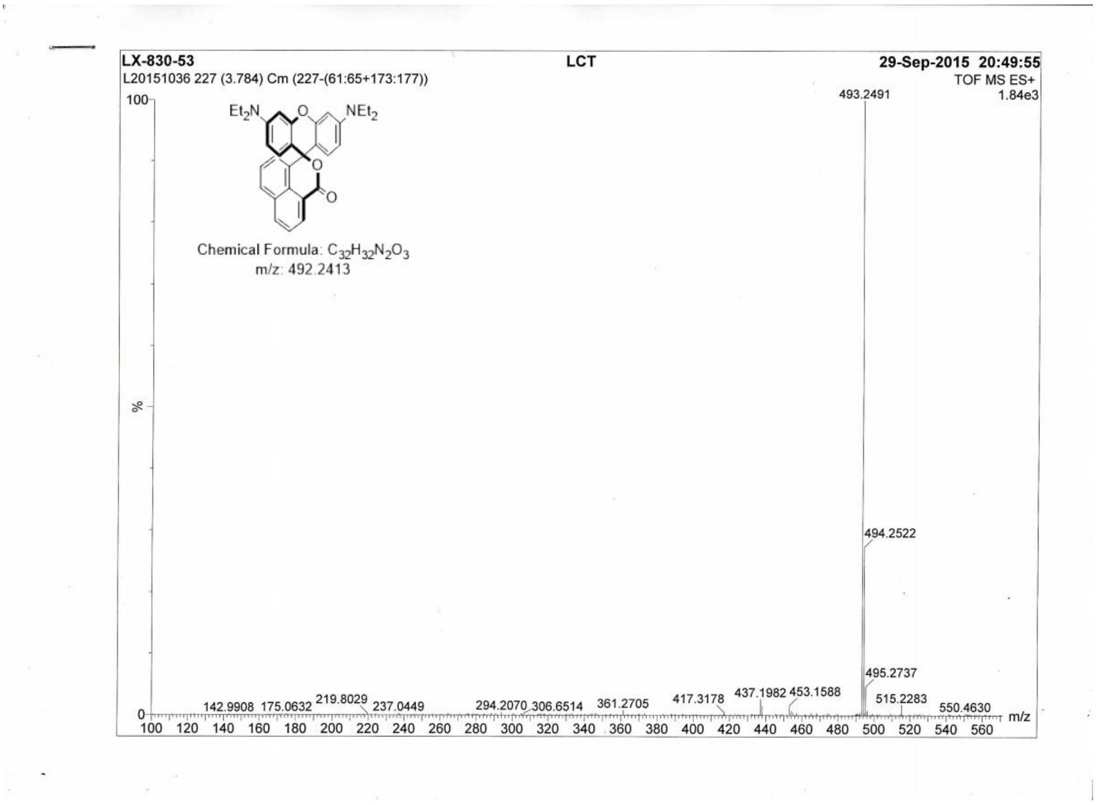

Supplementary Figure 227. The HR-MS of compound **RD26**

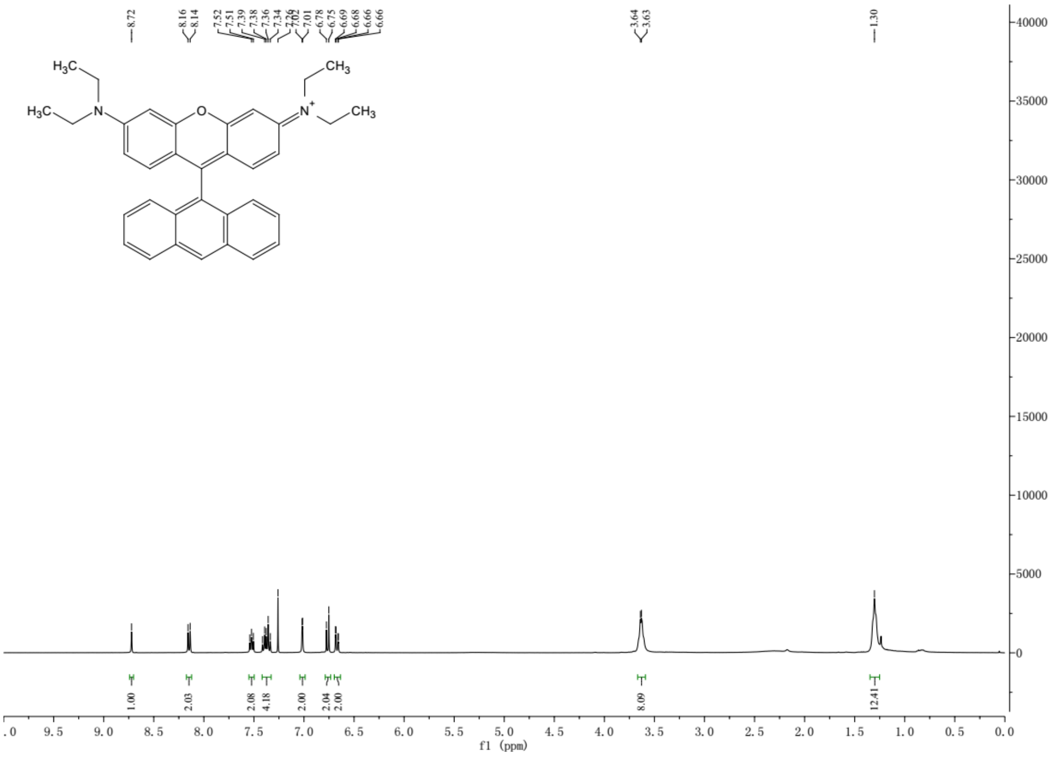

Supplementary Figure 228. The ^{1}H-NMR of compound **RD27** in $CDCl_3$

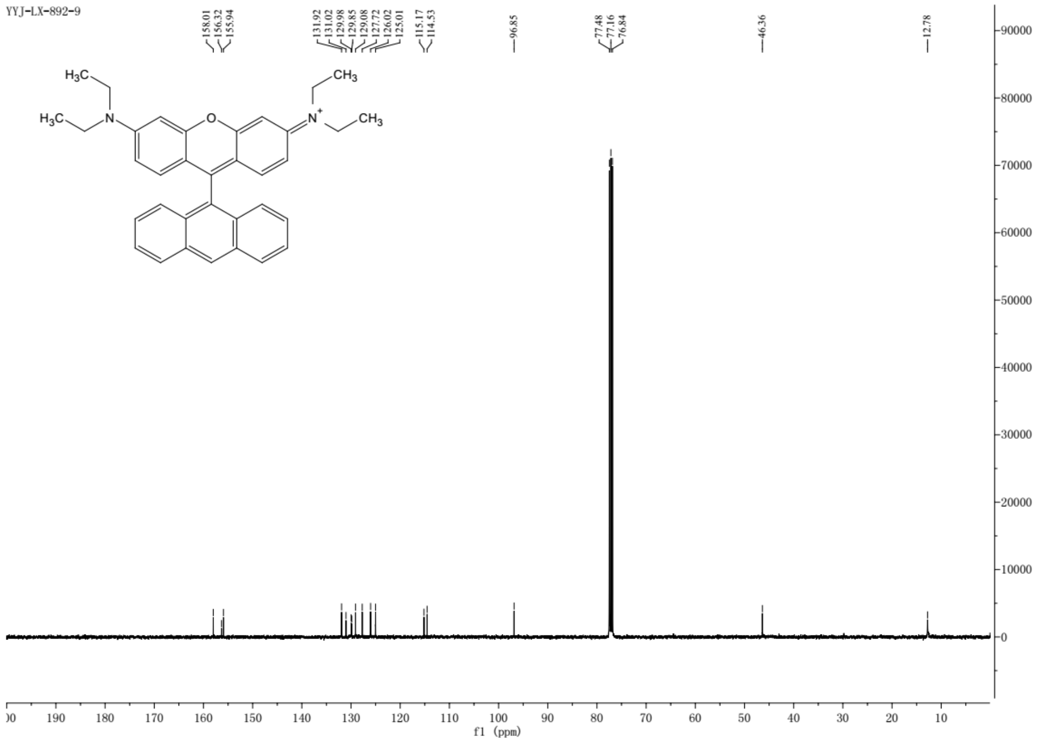

Supplementary Figure 229. The ^{13}C-NMR of compound **RD27** in $CDCl_3$

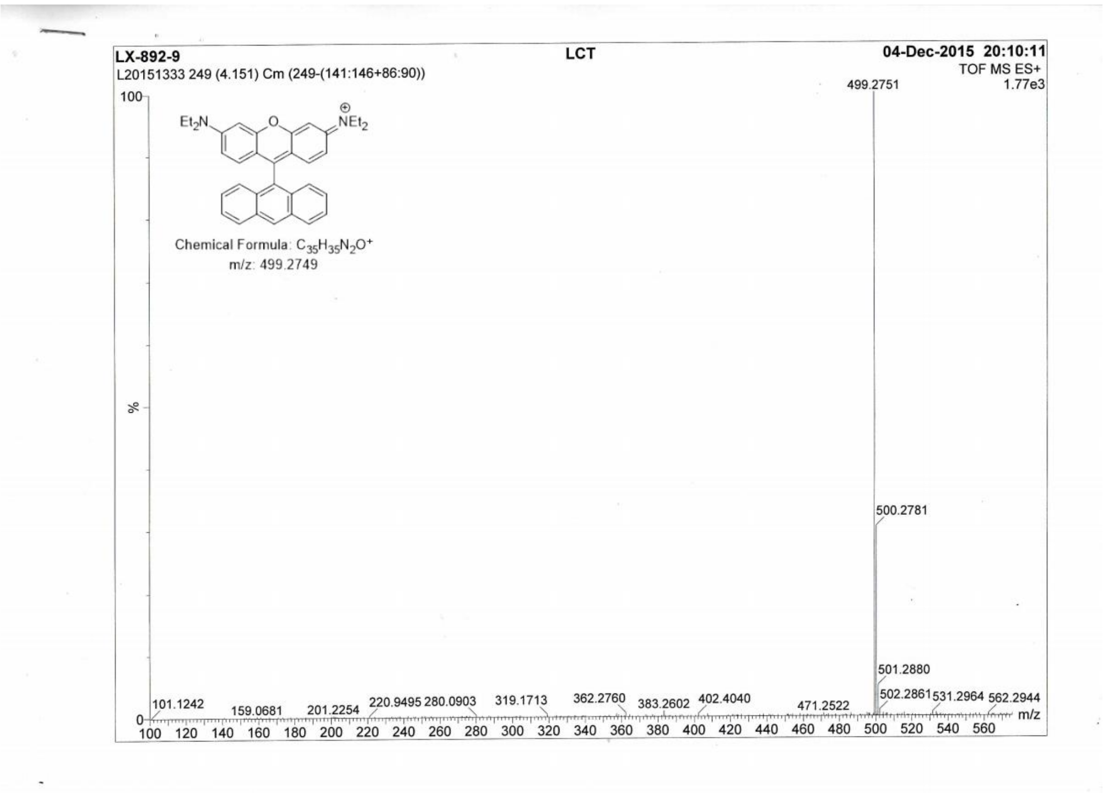

Supplementary Figure 230. The HR-MS of compound **RD27**

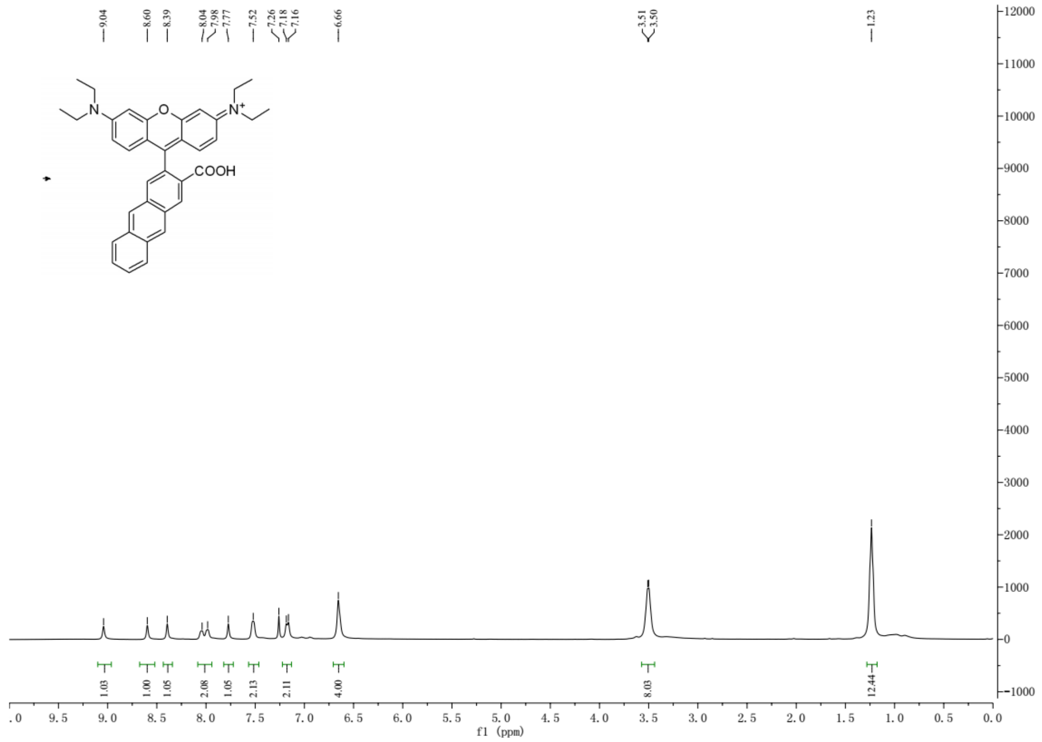

Supplementary Figure 231. The ^{1}H-NMR of compound **RD28** in $CDCl_3$

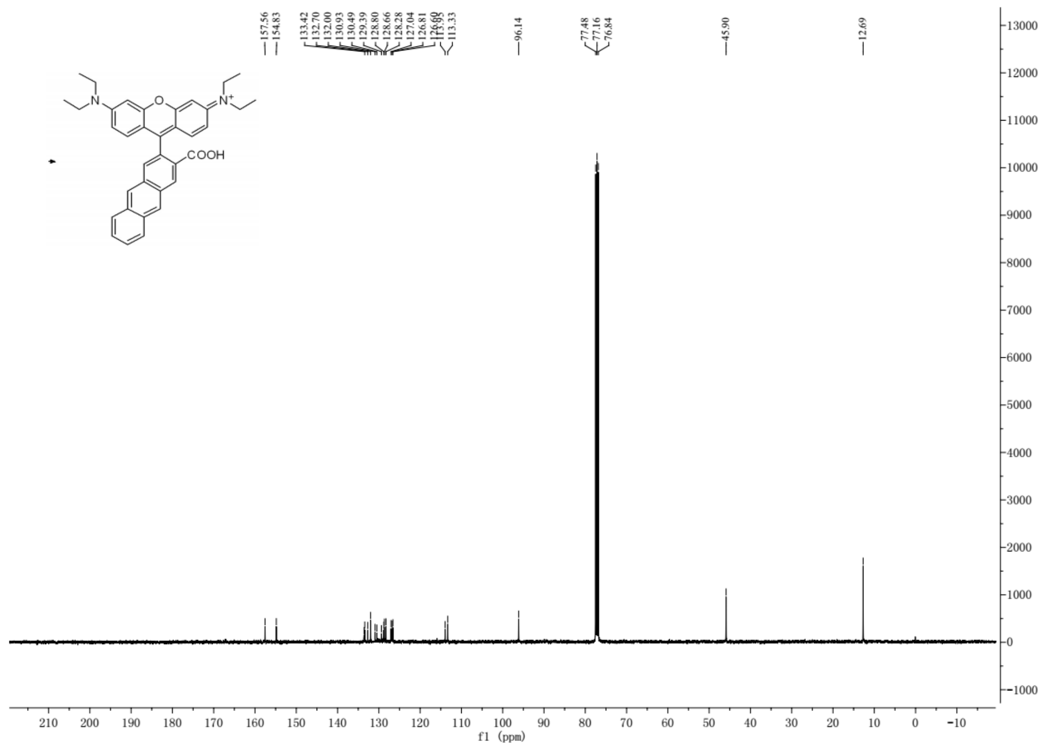

Supplementary Figure 232. The ^{13}C-NMR of compound **RD28** in $CDCl_3$

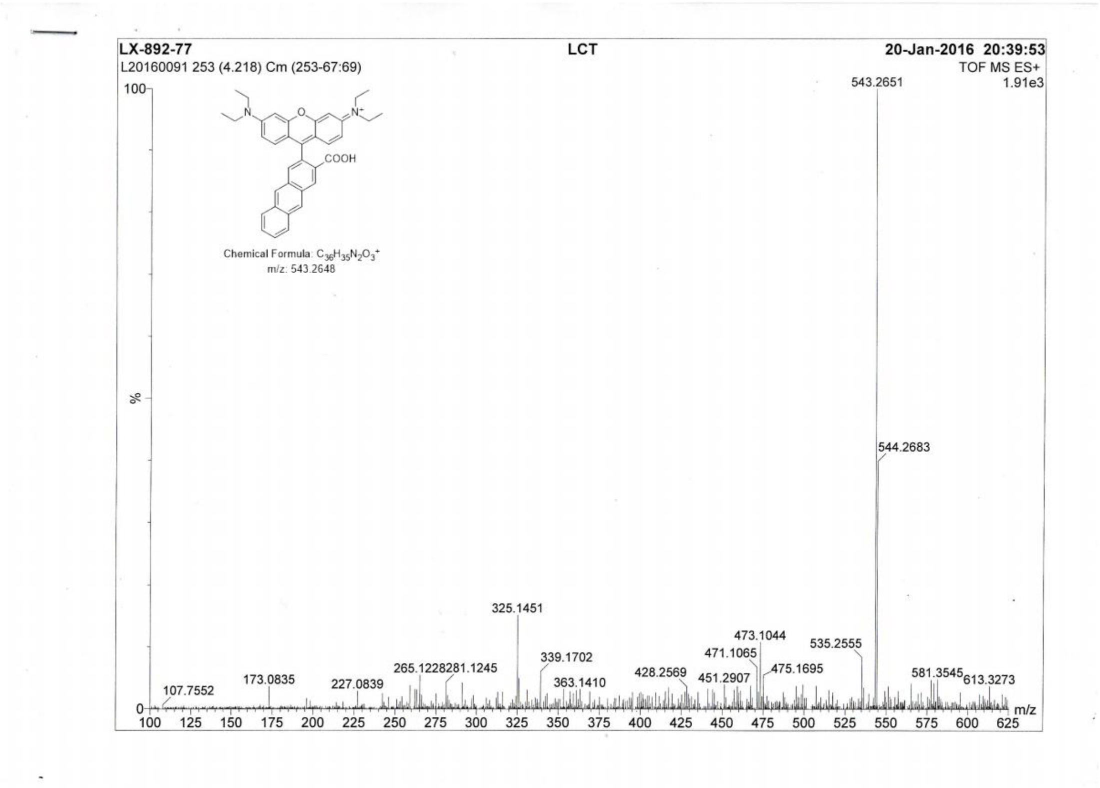

Supplementary Figure 233. The HR-MS of compound **RD28**

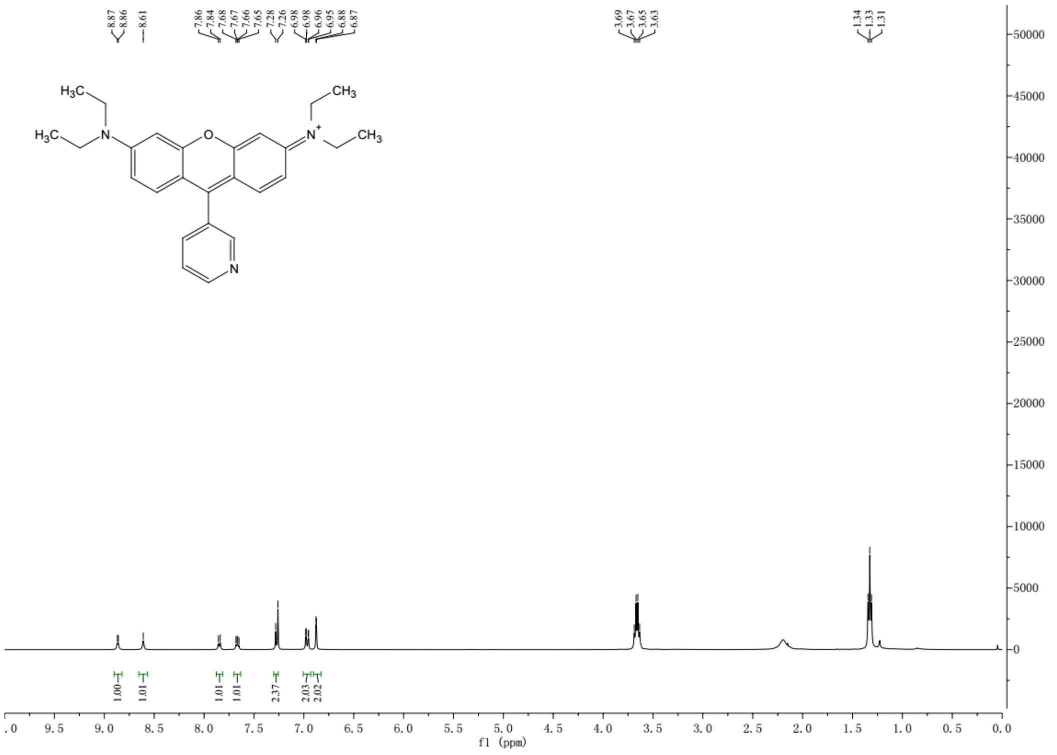

Supplementary Figure 234. The ^{1}H-NMR of compound **RD29** in $CDCl_3$

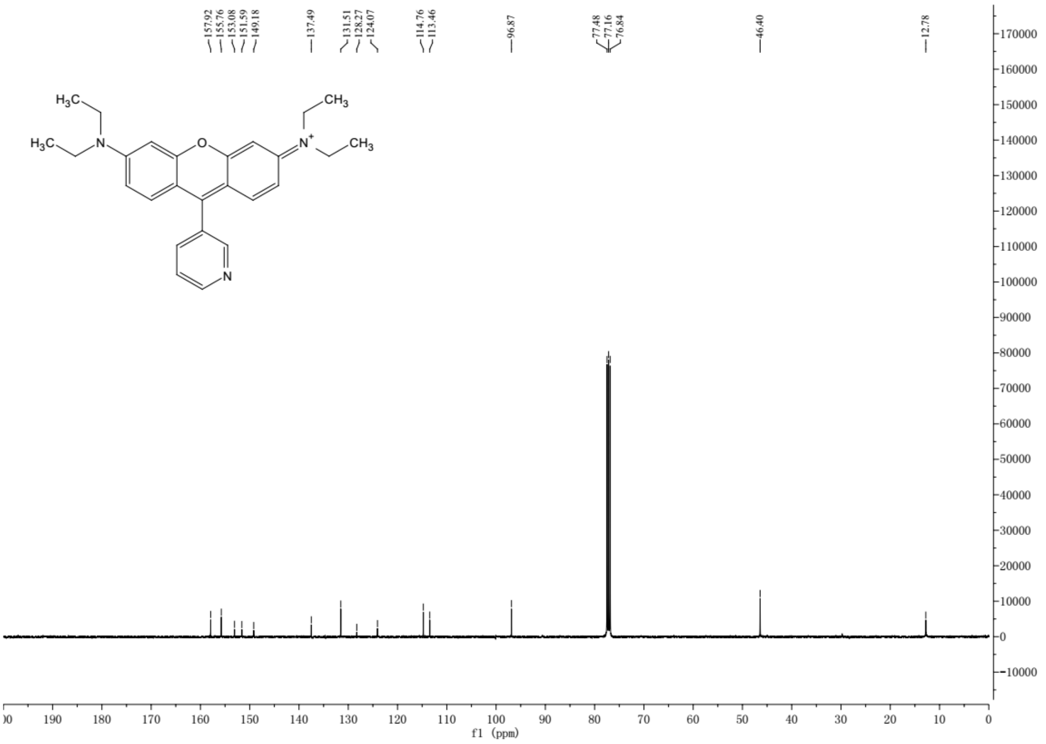

Supplementary Figure 235. The ^{13}C-NMR of compound **RD29** in $CDCl_3$

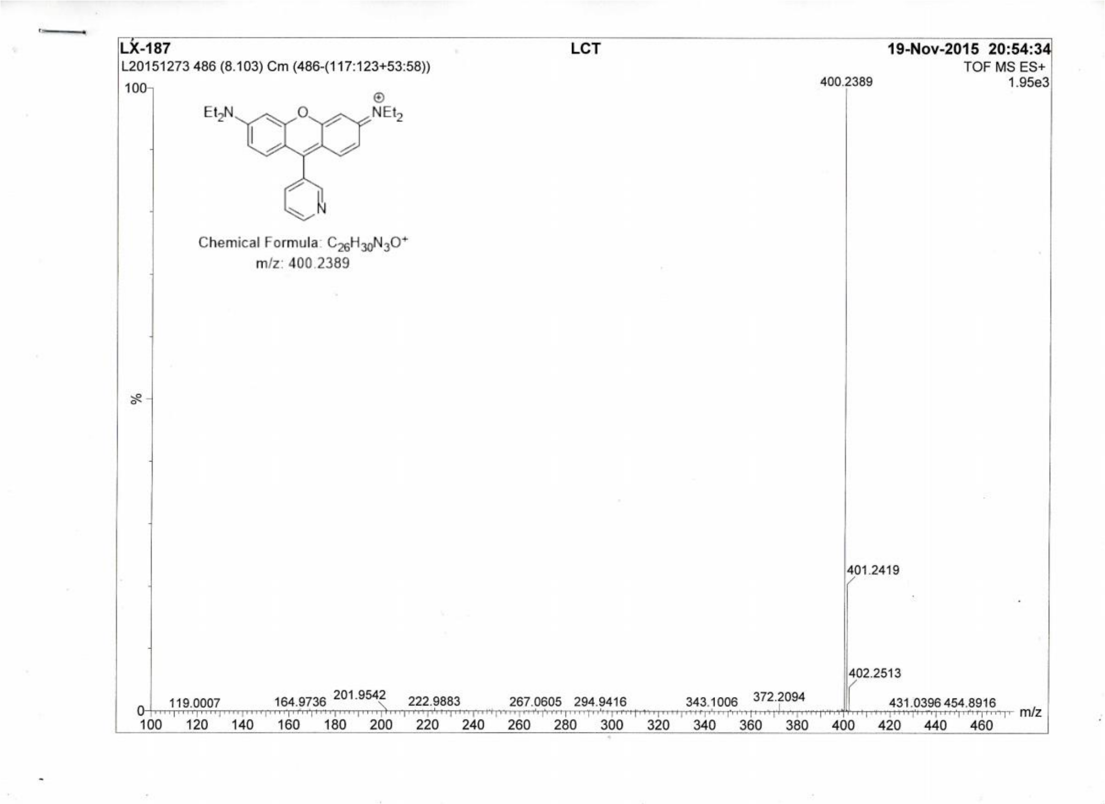

Supplementary Figure 236. The HR-MS of compound **RD29**

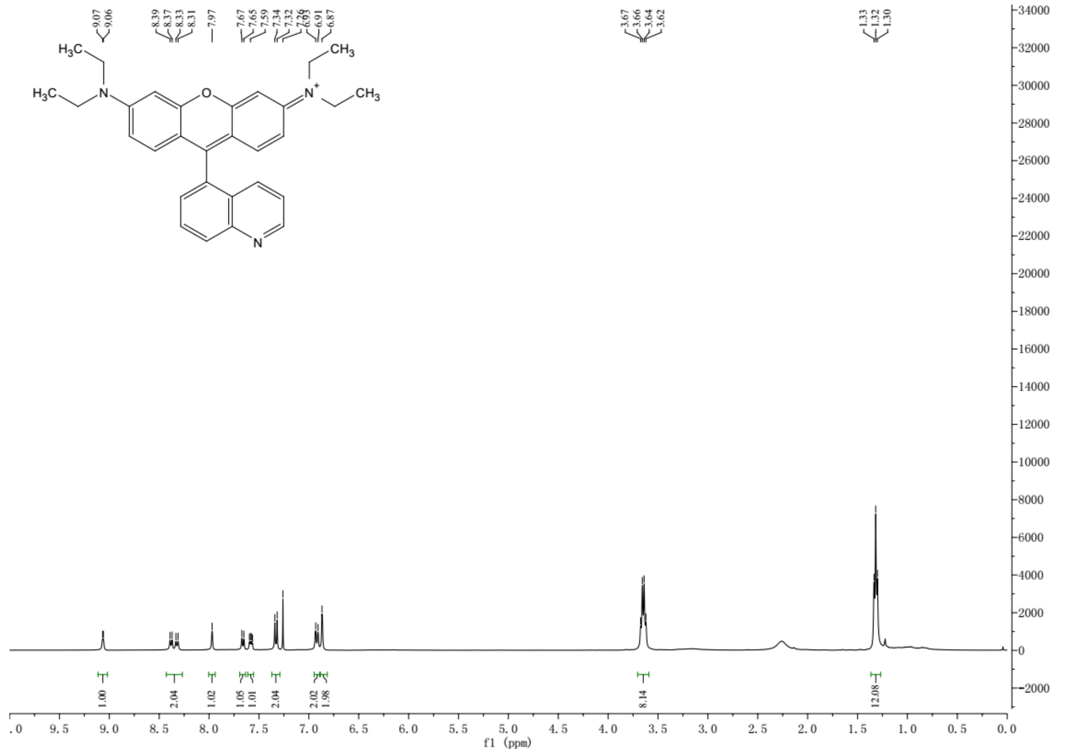

Supplementary Figure 237. The ^{1}H-NMR of compound **RD30** in $CDCl_3$

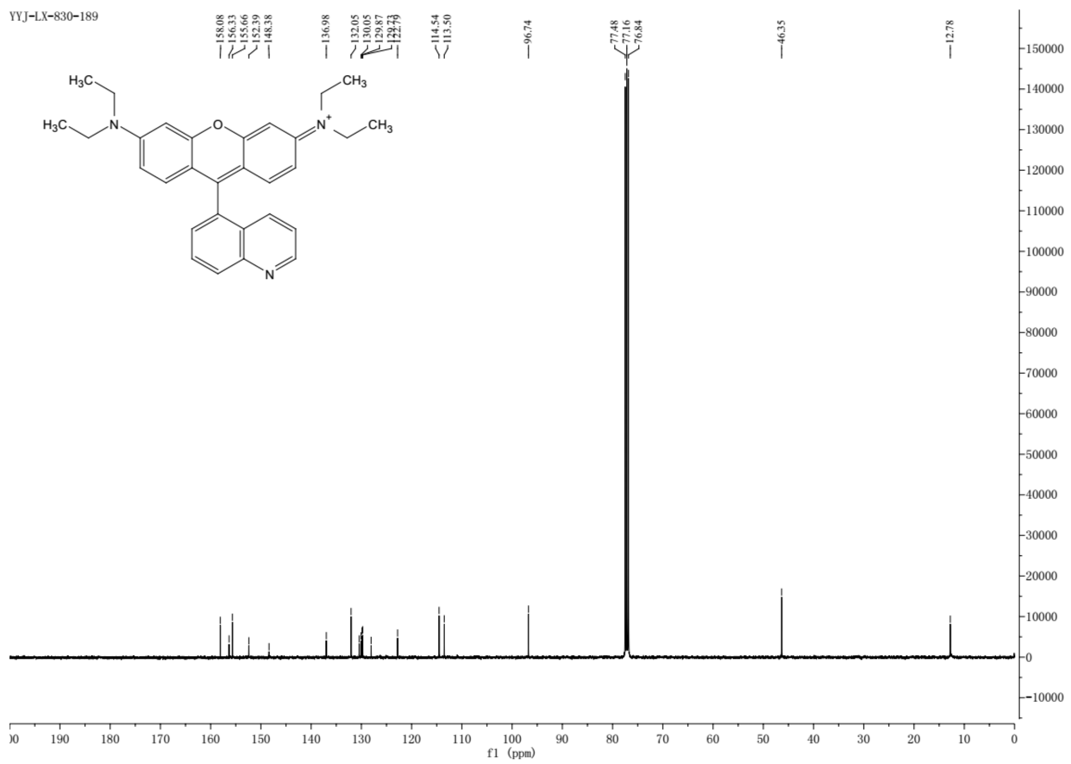

Supplementary Figure 238. The ^{13}C-NMR of compound **RD30** in $CDCl_3$

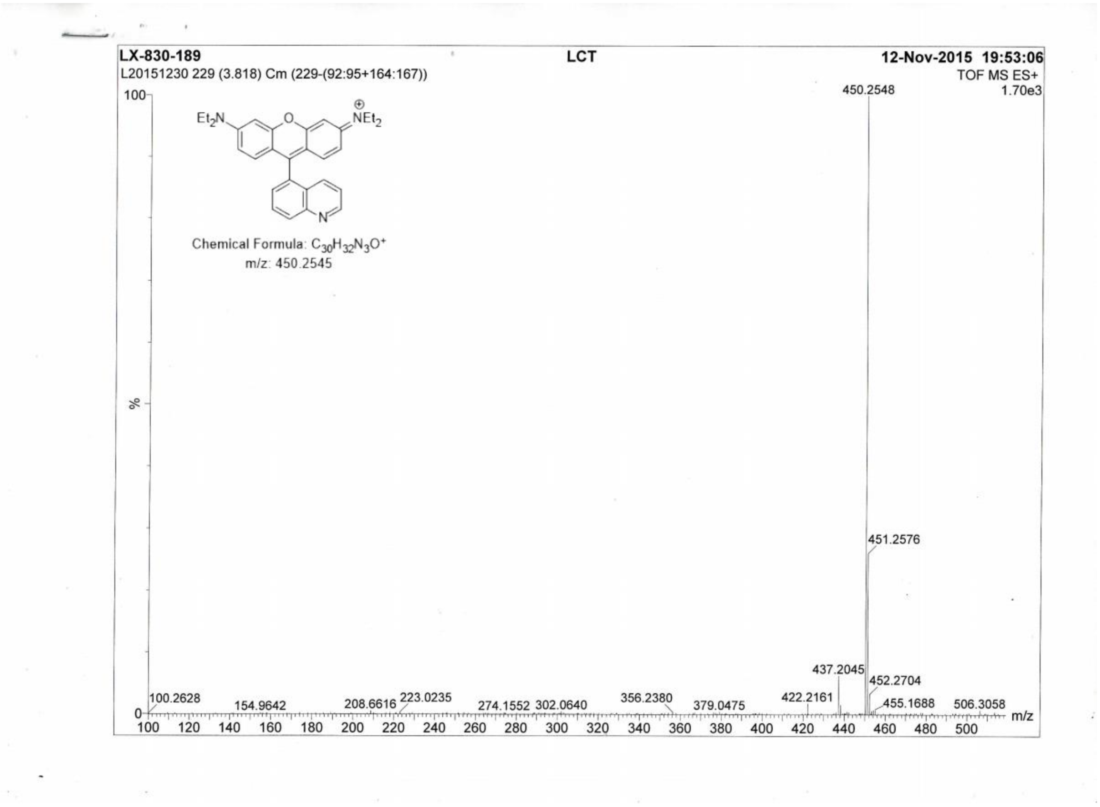

Supplementary Figure 239. The HR-MS of compound **RD30**

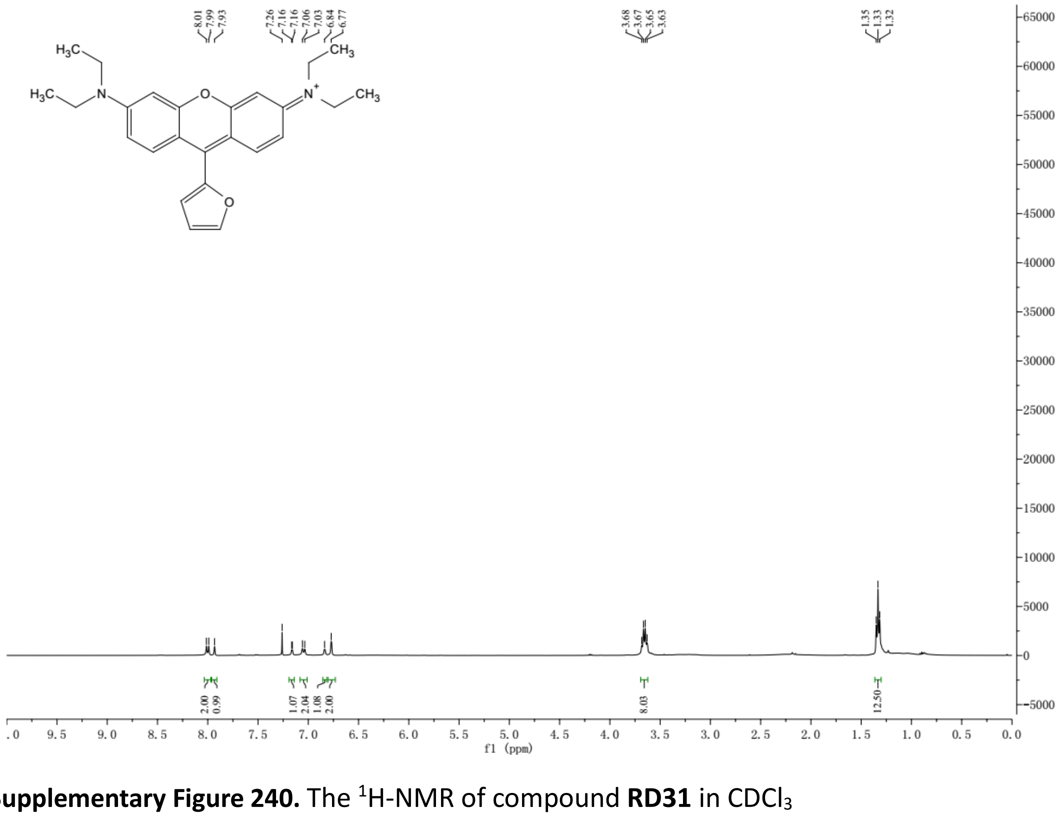

Supplementary Figure 240. The ^{1}H-NMR of compound **RD31** in $CDCl_3$

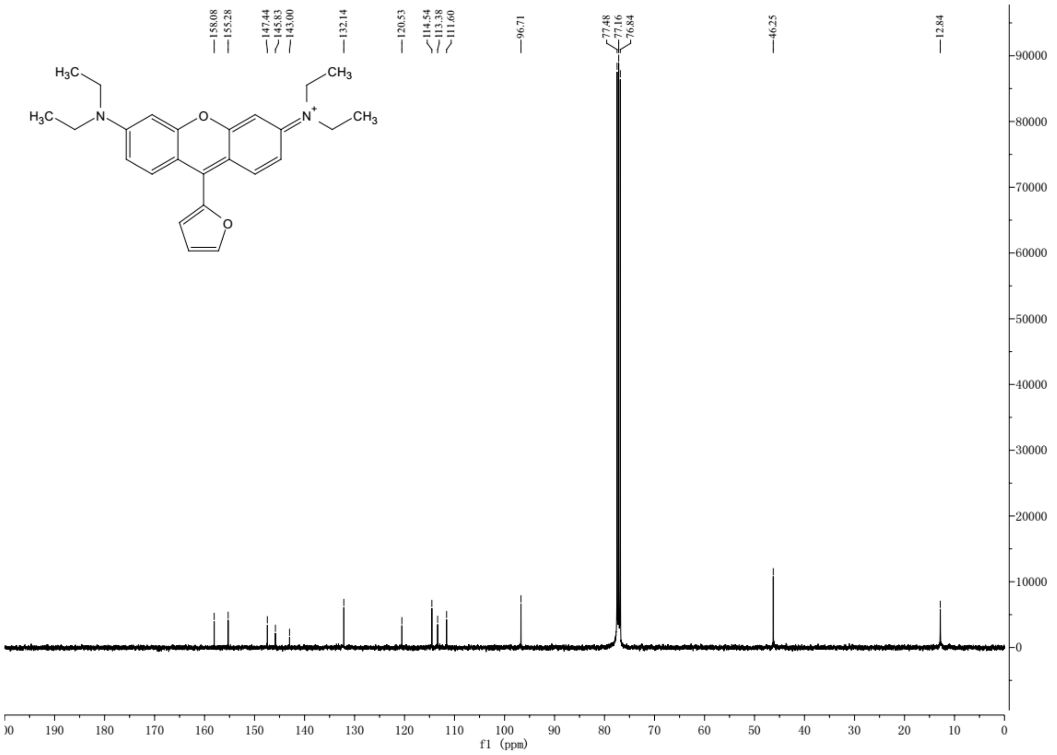

Supplementary Figure 241. The ^{13}C-NMR of compound **RD31** in $CDCl_3$

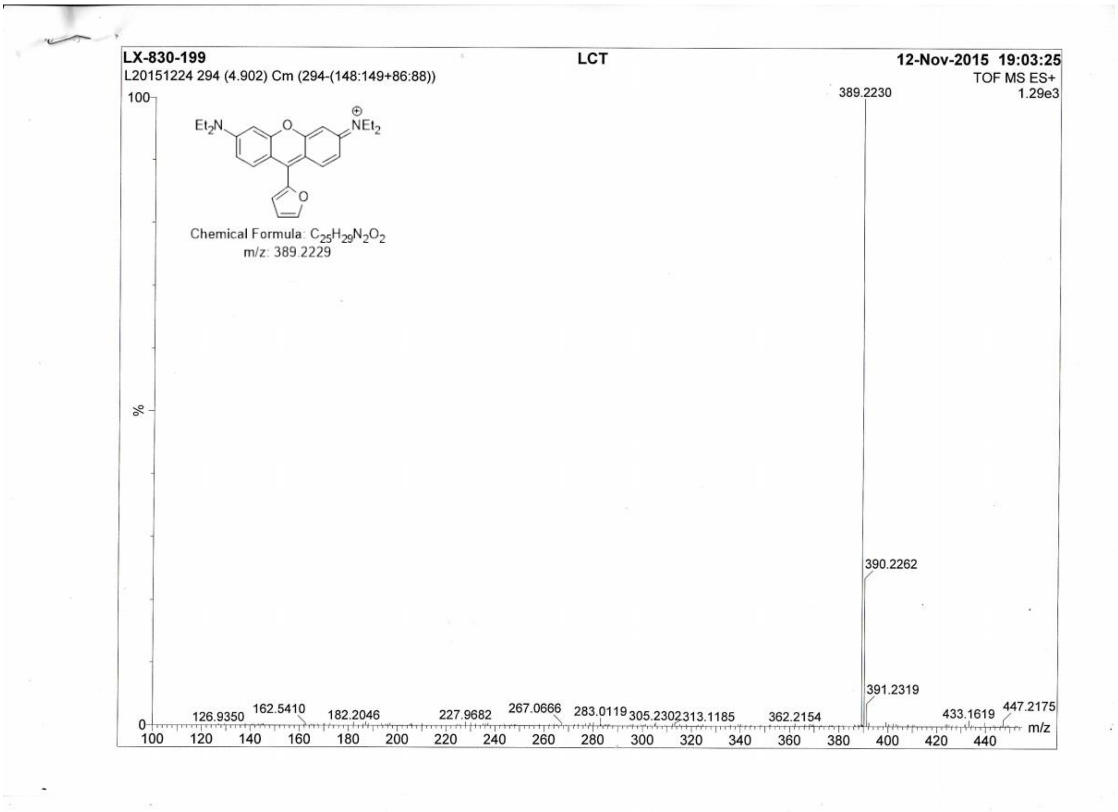

Supplementary Figure 242. The HR-MS of compound **RD31**

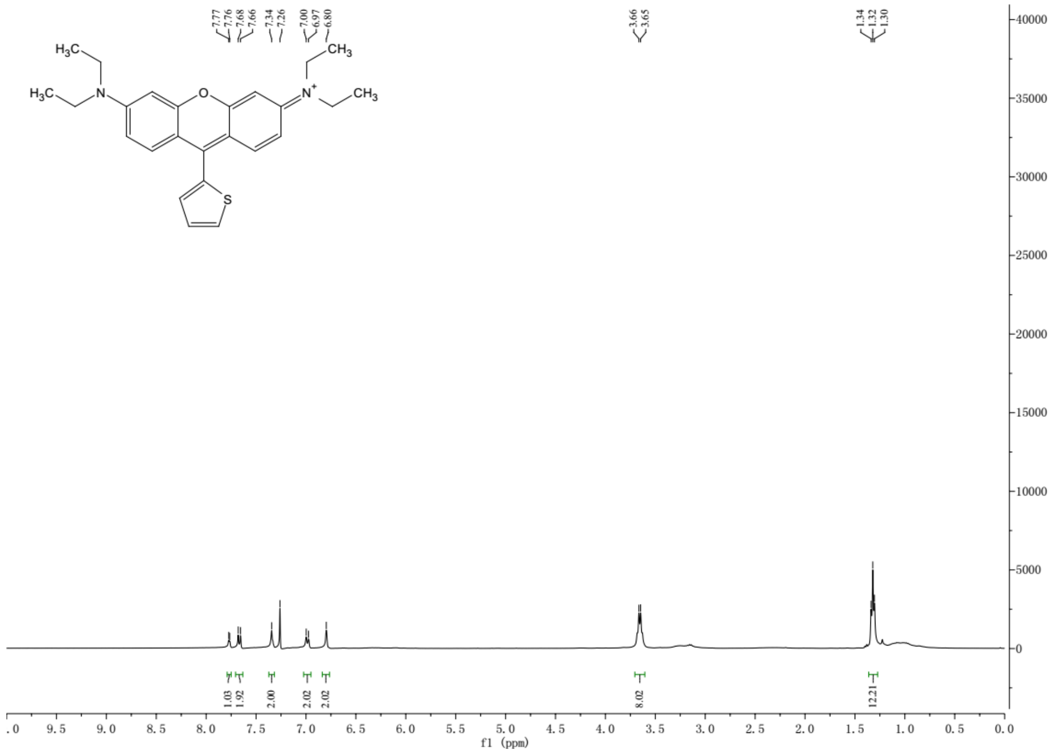

Supplementary Figure 243. The ^{1}H-NMR of compound **RD32** in $CDCl_3$

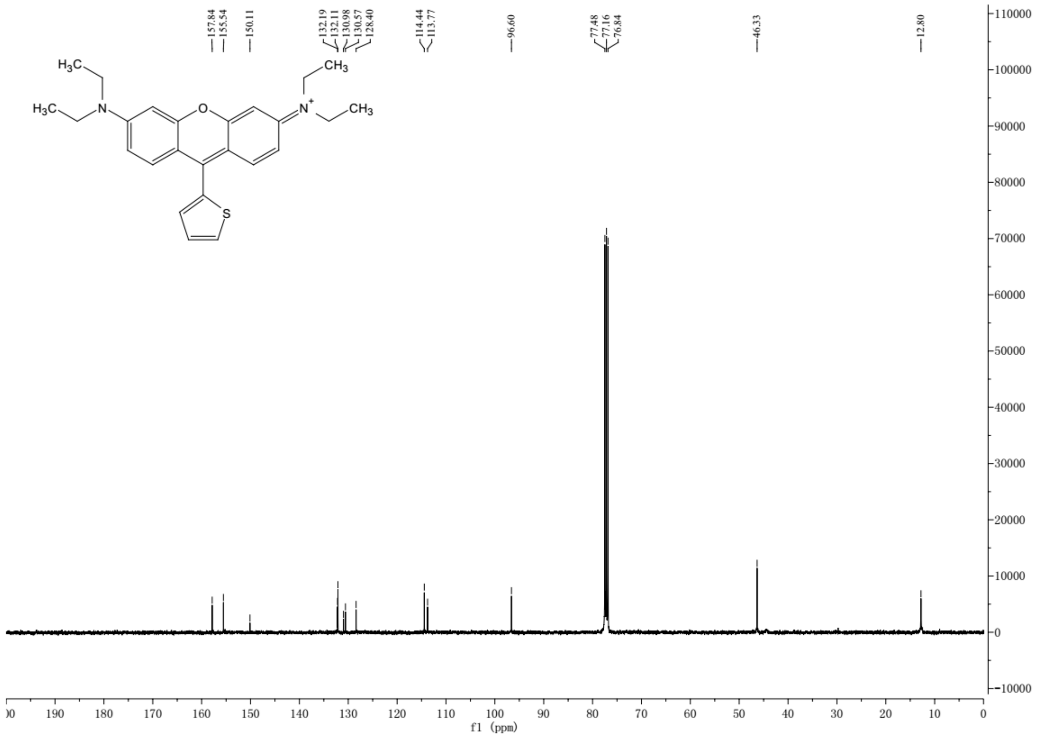

Supplementary Figure 244. The ^{13}C-NMR of compound **RD32** in $CDCl_3$

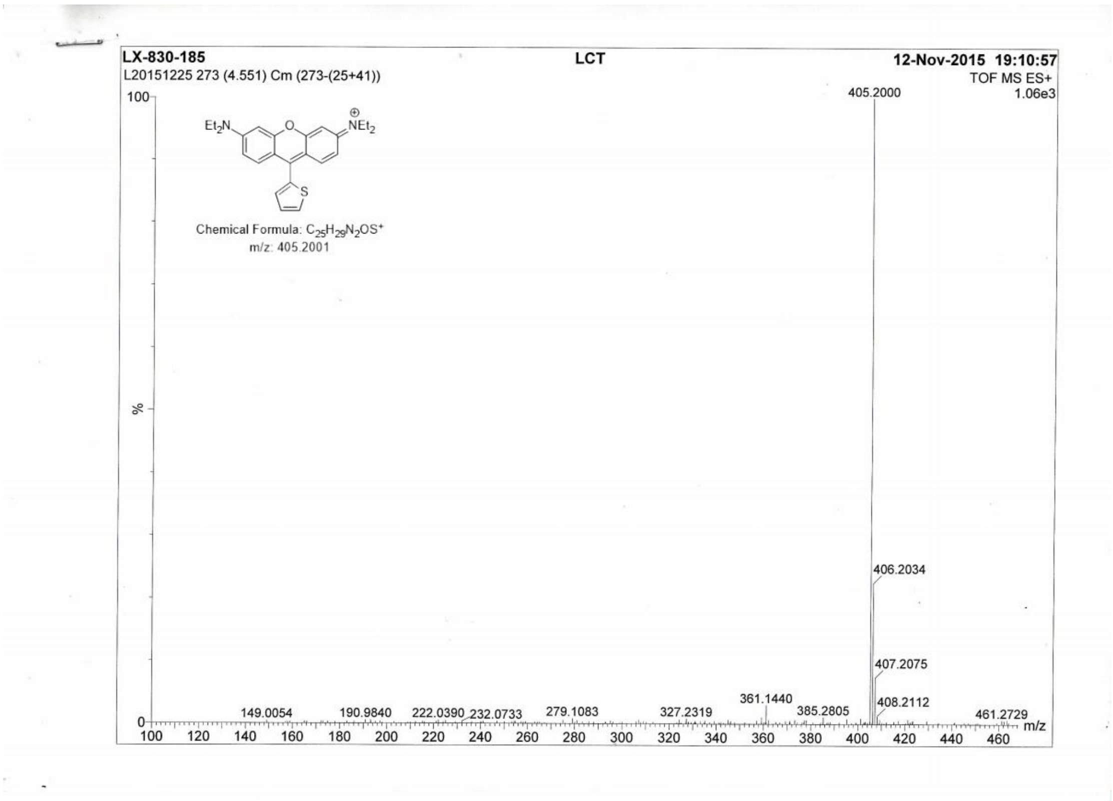

Supplementary Figure 245. The HR-MS of compound **RD32**

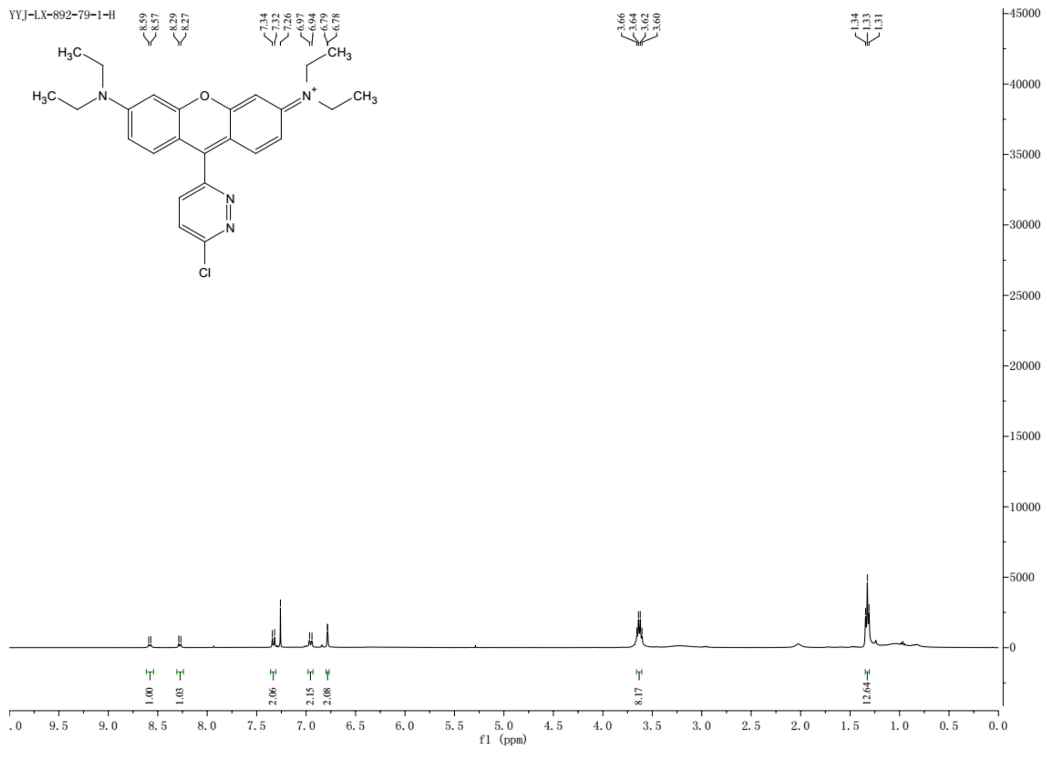

Supplementary Figure 246. The ^{1}H-NMR of compound **RD33** in $CDCl_3$

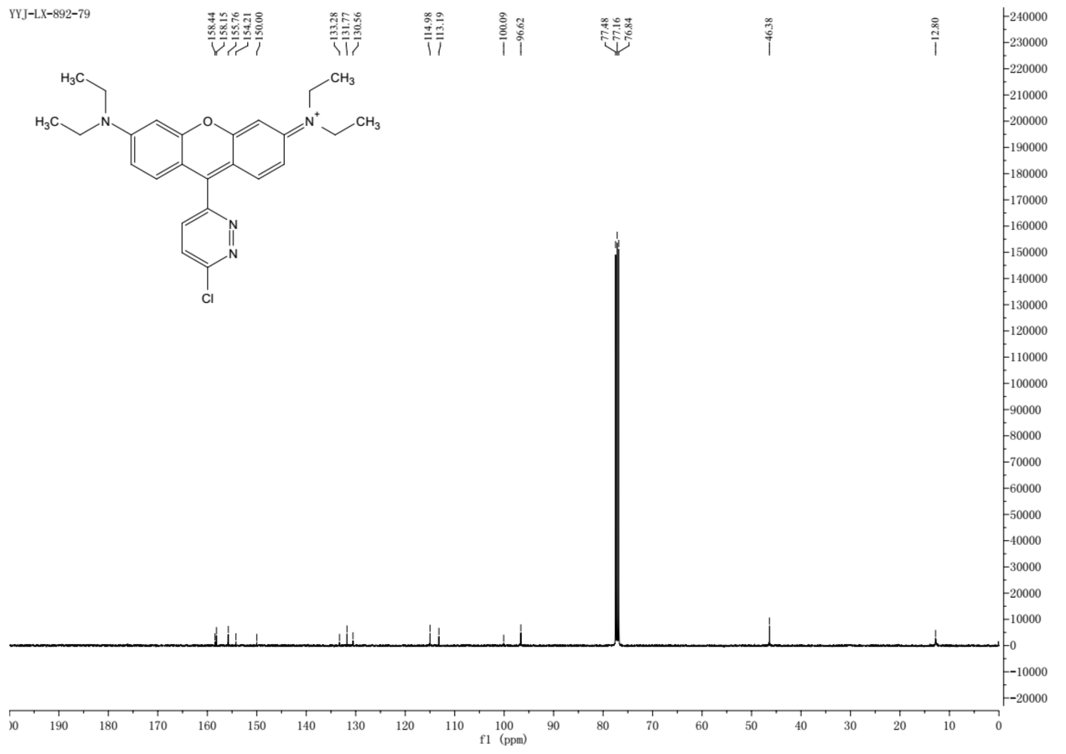

Supplementary Figure 247. The ^{13}C-NMR of compound **RD33** in $CDCl_3$

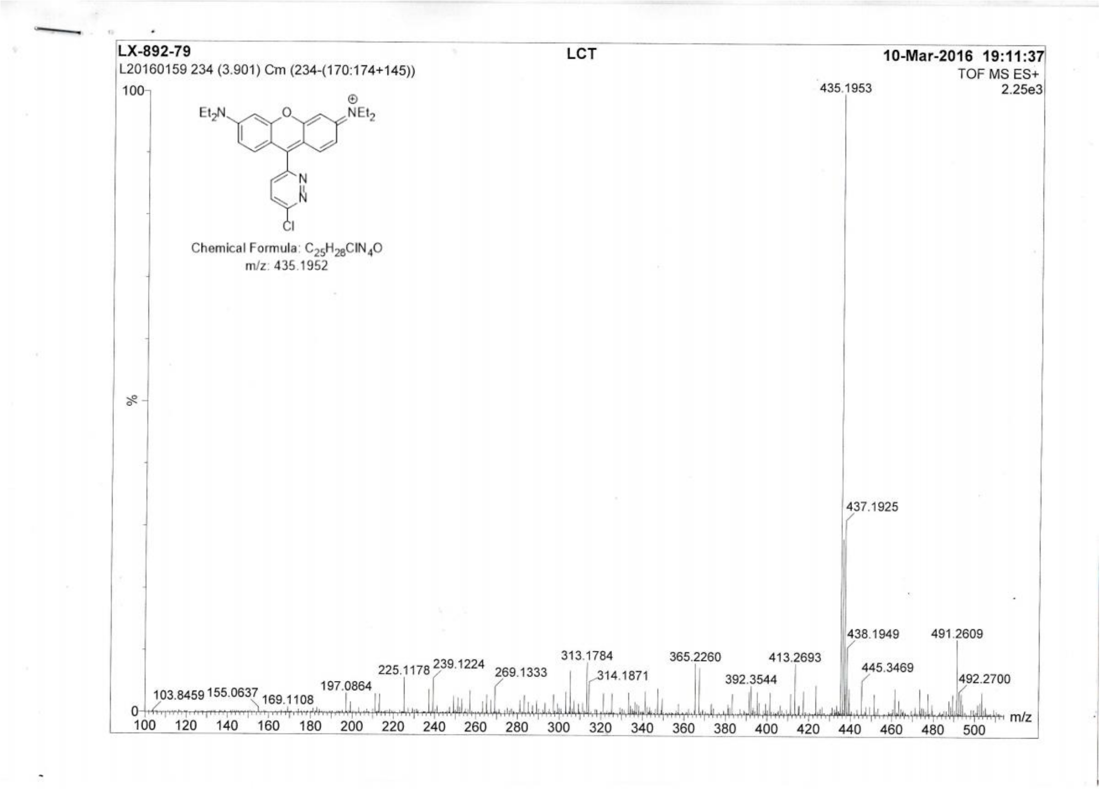

Supplementary Figure 248. The HR-MS of compound **RD33**

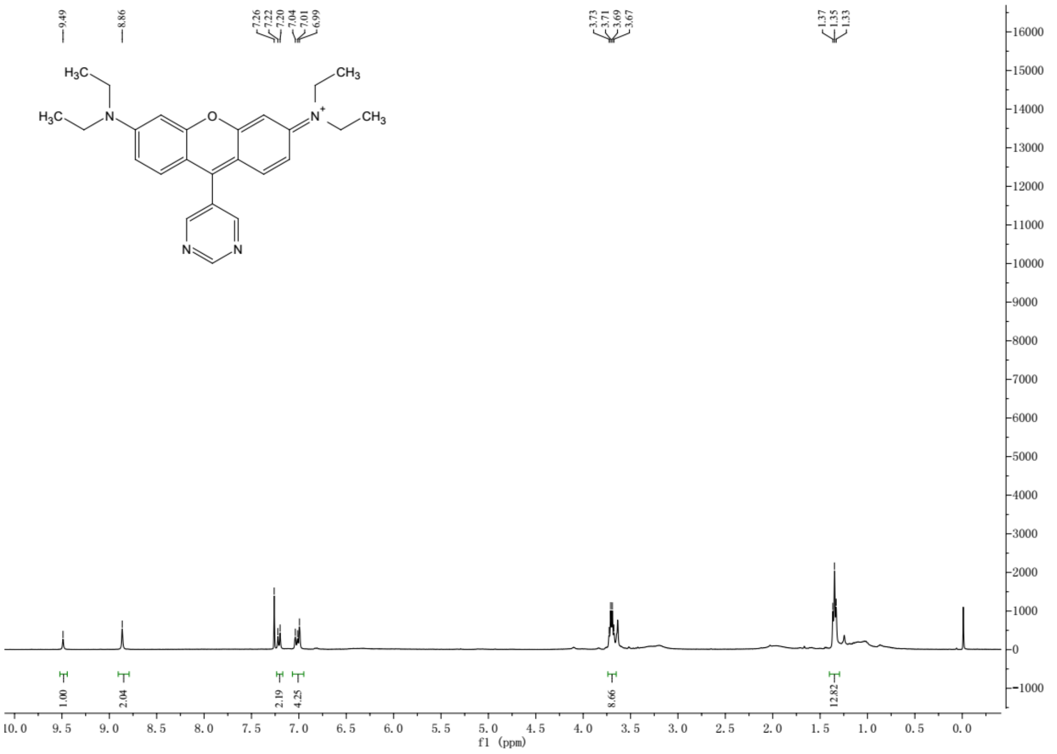

Supplementary Figure 249. The ^{1}H-NMR of compound **RD34** in $CDCl_3$

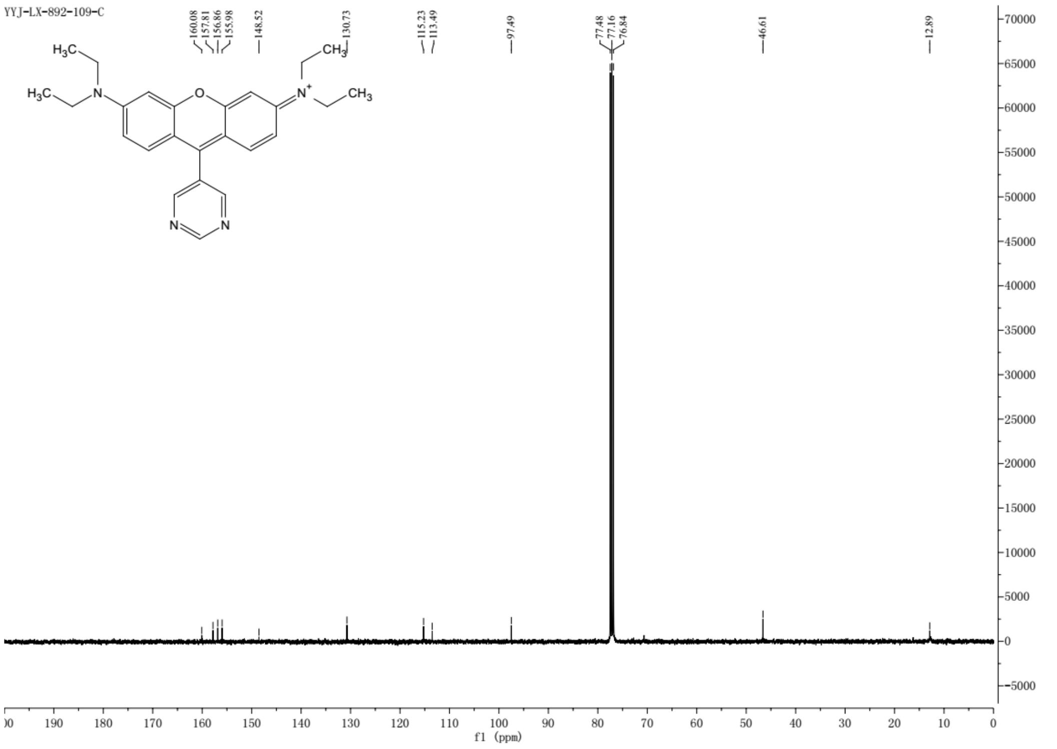

Supplementary Figure 250. The ^{13}C-NMR of compound **RD34** in $CDCl_3$

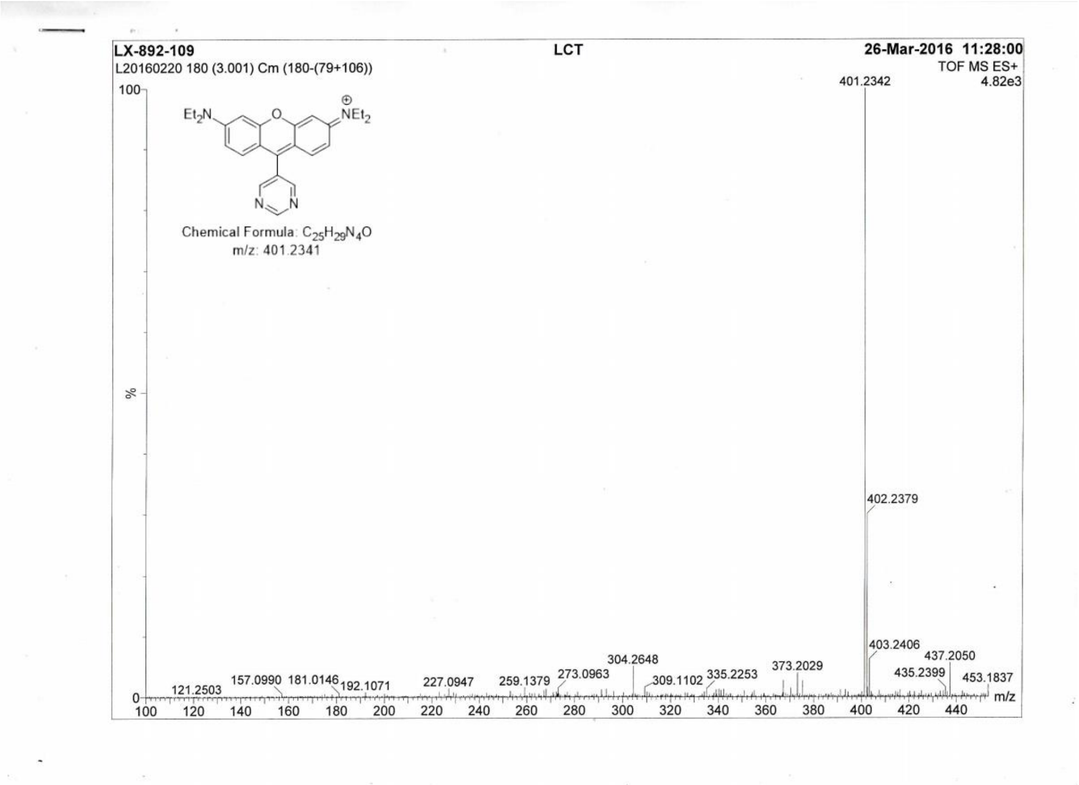

Supplementary Figure 251. The HR-MS of compound **RD34**

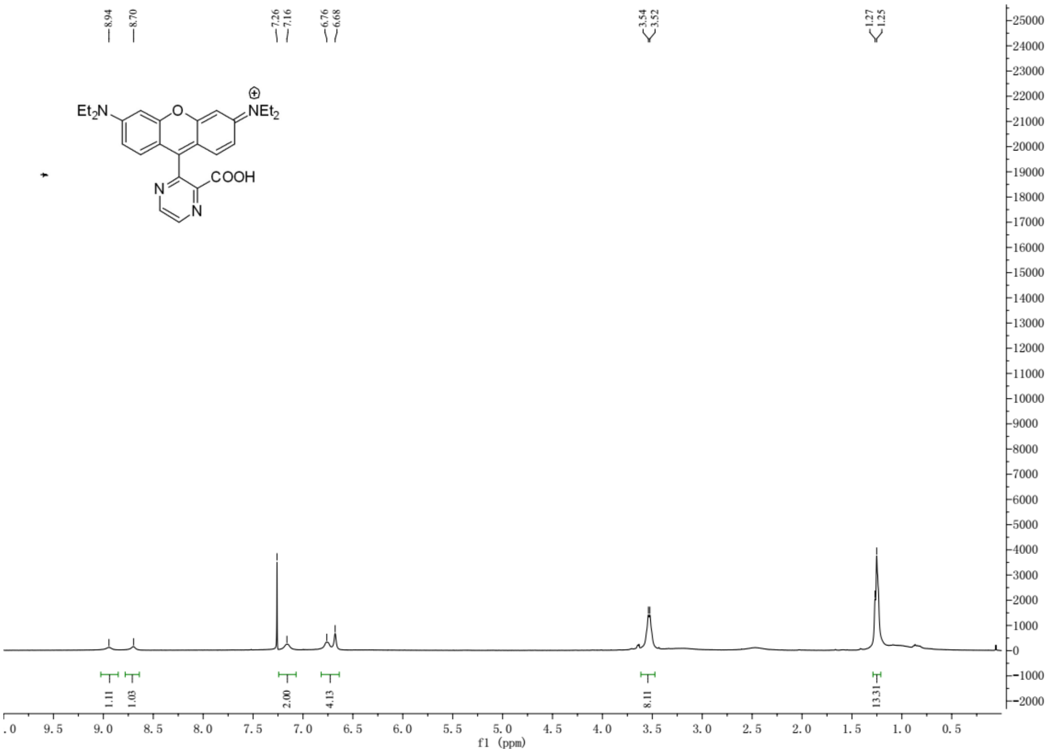

Supplementary Figure 252. The ^{1}H-NMR of compound **RD35** in $CDCl_3$

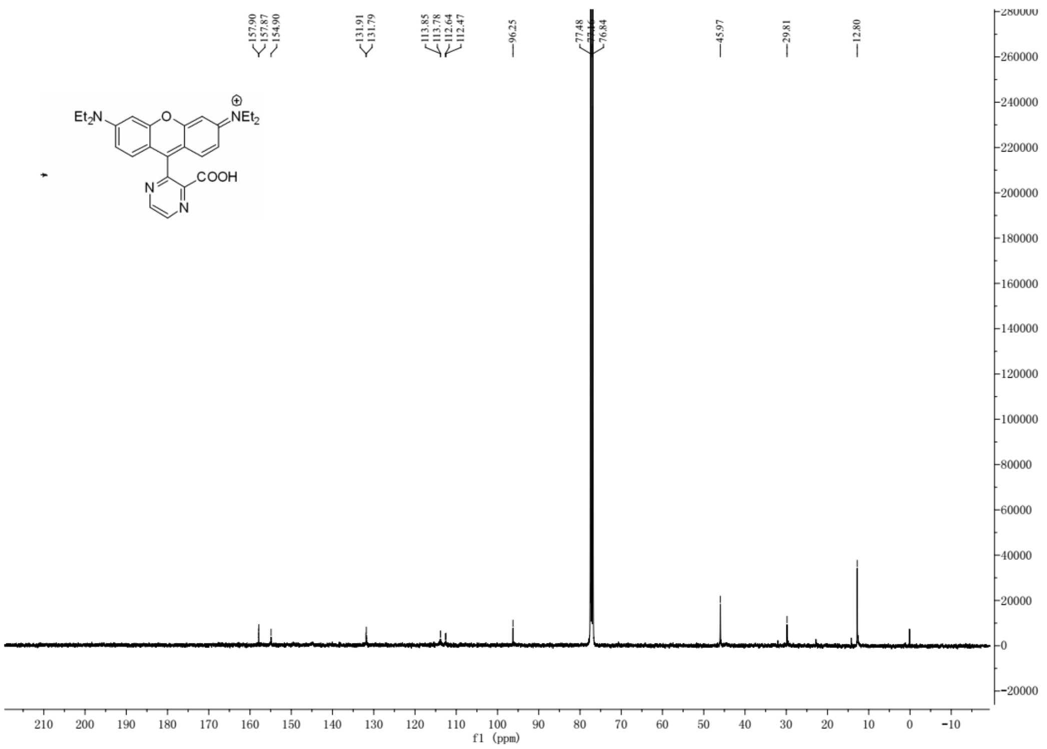

Supplementary Figure 253. The ^{13}C-NMR of compound **RD35** in $CDCl_3$

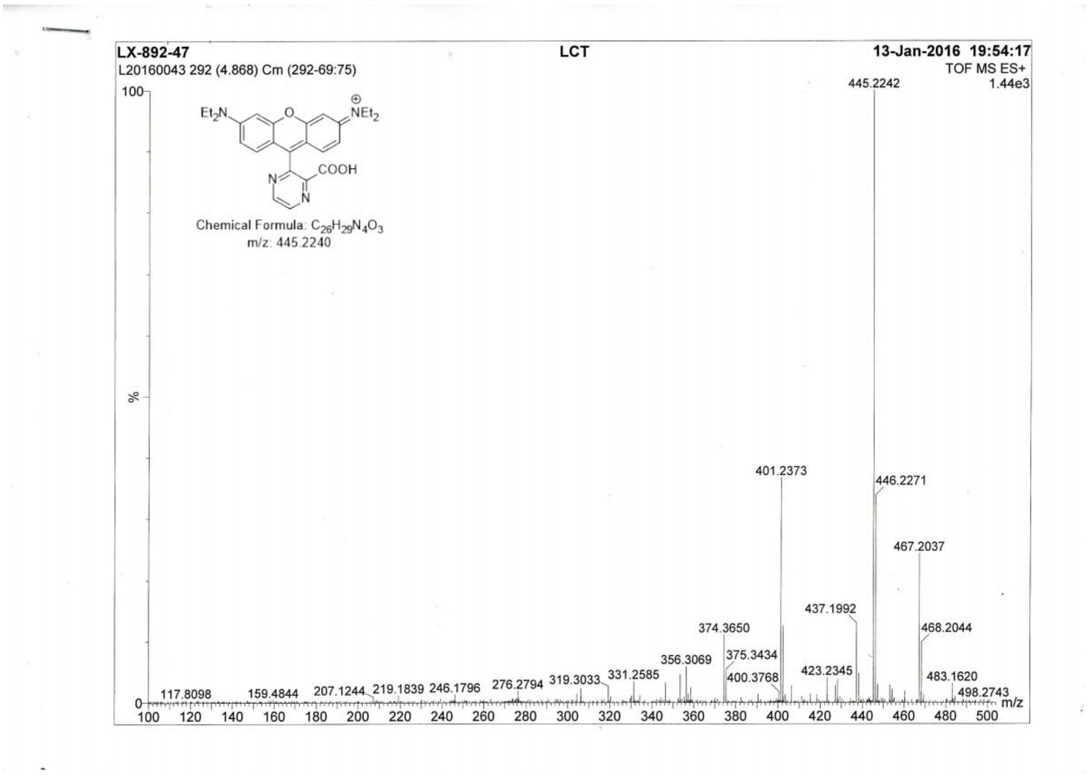

Supplementary Figure 254. The HR-MS of compound **RD35**

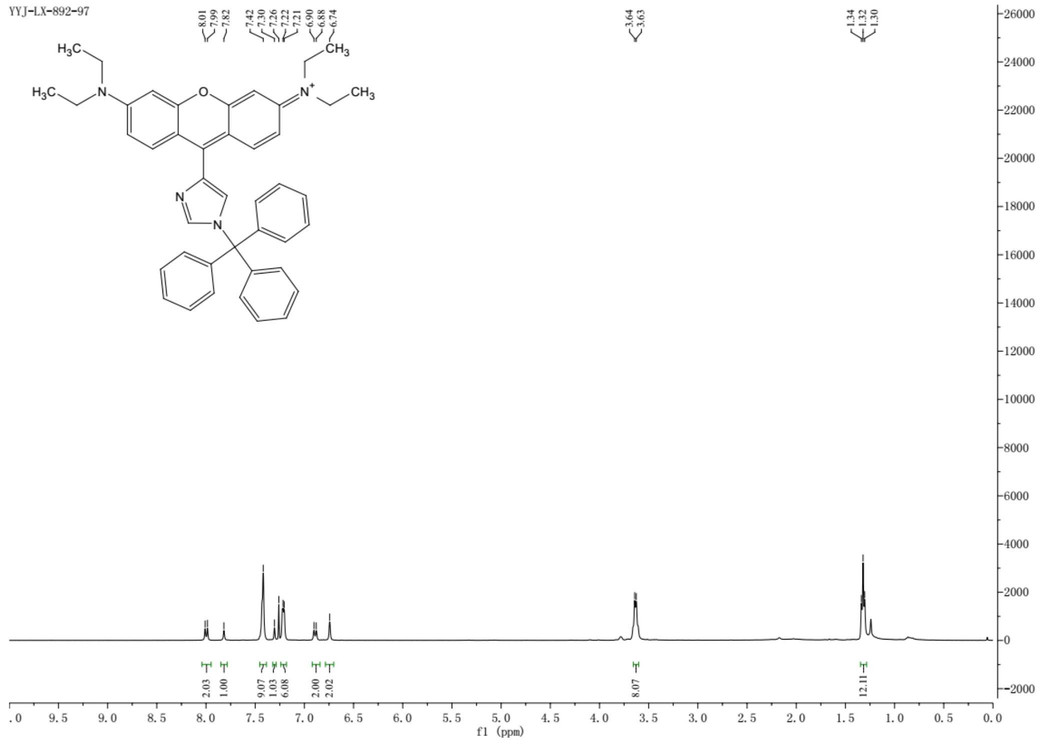

Supplementary Figure 255. The ^{1}H-NMR of compound **RD36** in $CDCl_3$

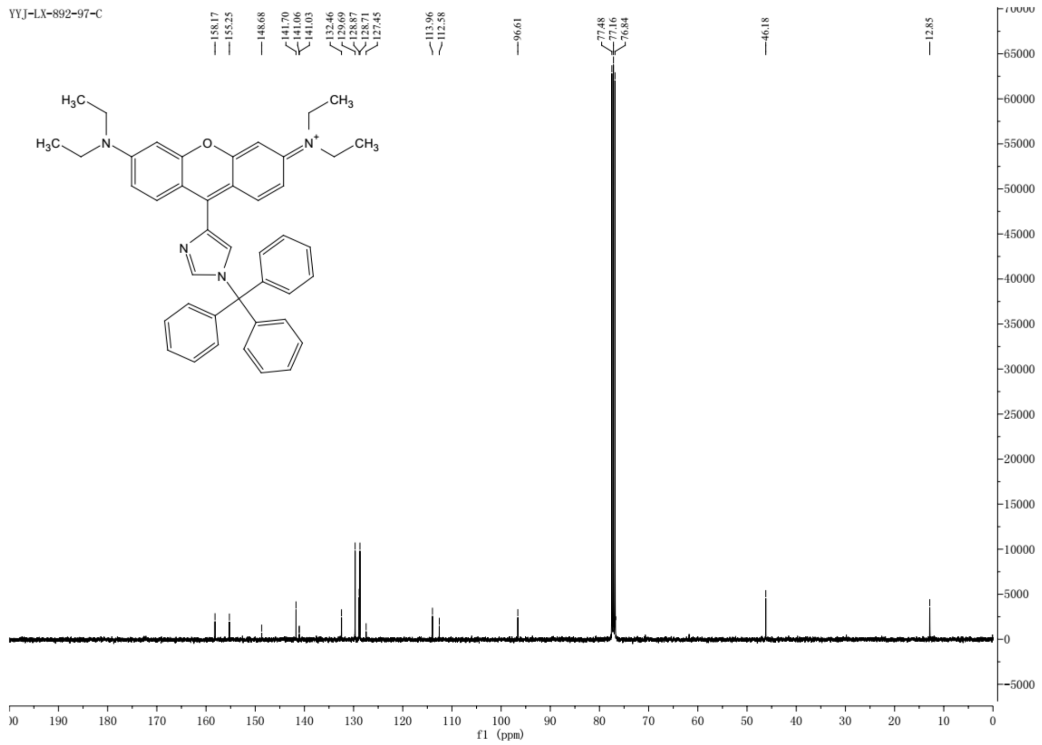

Supplementary Figure 256. The ^{13}C-NMR of compound **RD36** in $CDCl_3$

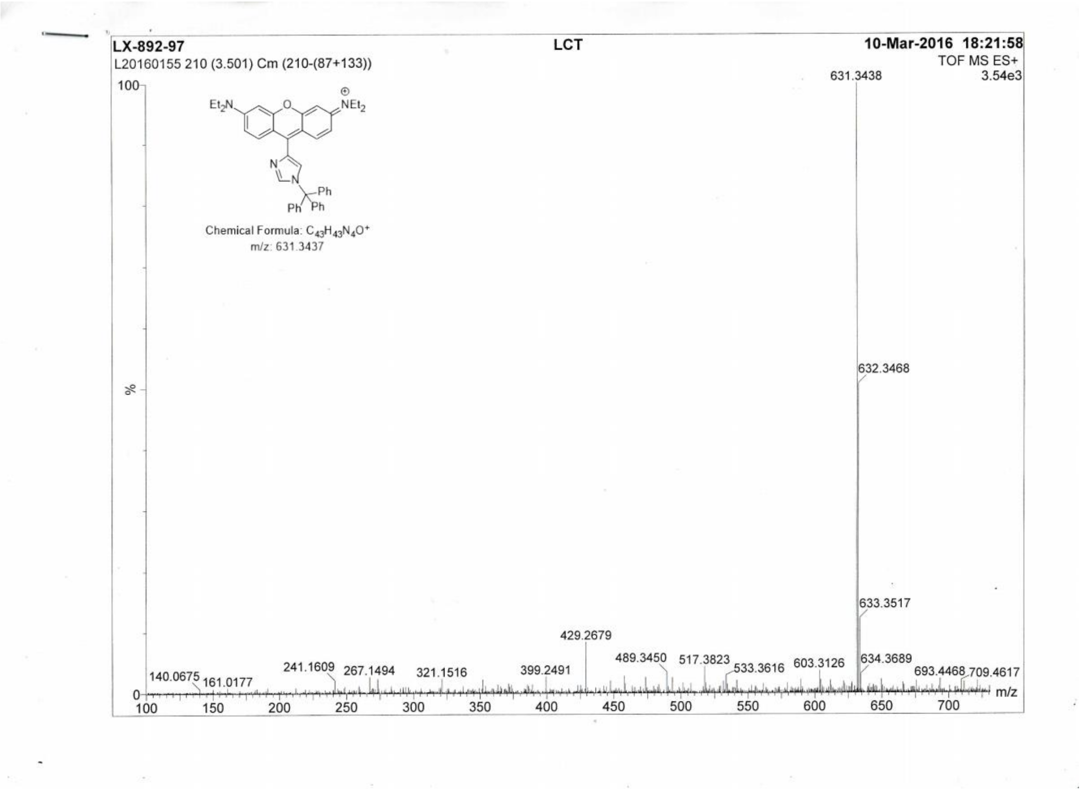

Supplementary Figure 257. The HR-MS of compound **RD36**

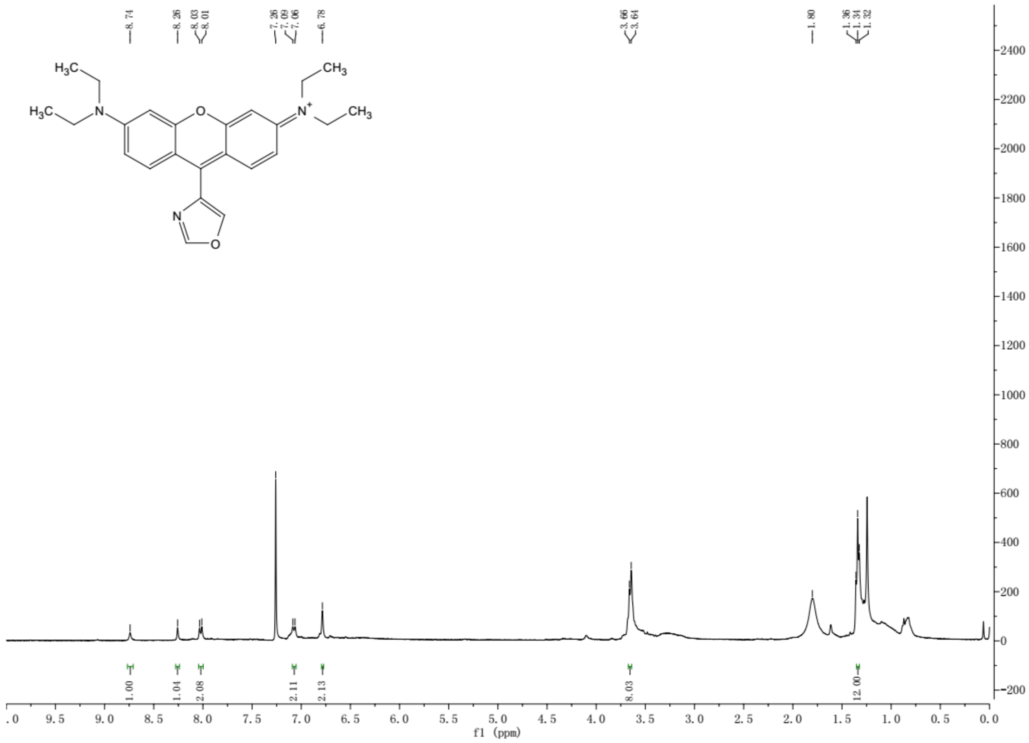

Supplementary Figure 258. The ^{1}H-NMR of compound **RD37** in $CDCl_3$

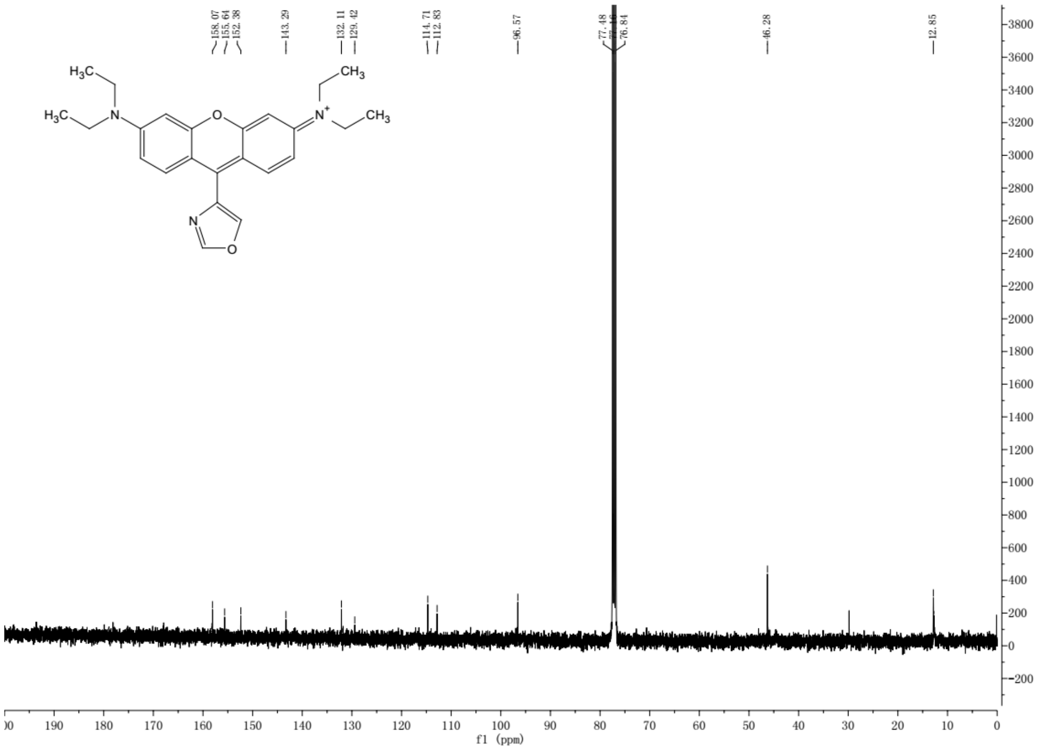

Supplementary Figure 259. The ^{13}C-NMR of compound **RD37** in $CDCl_3$

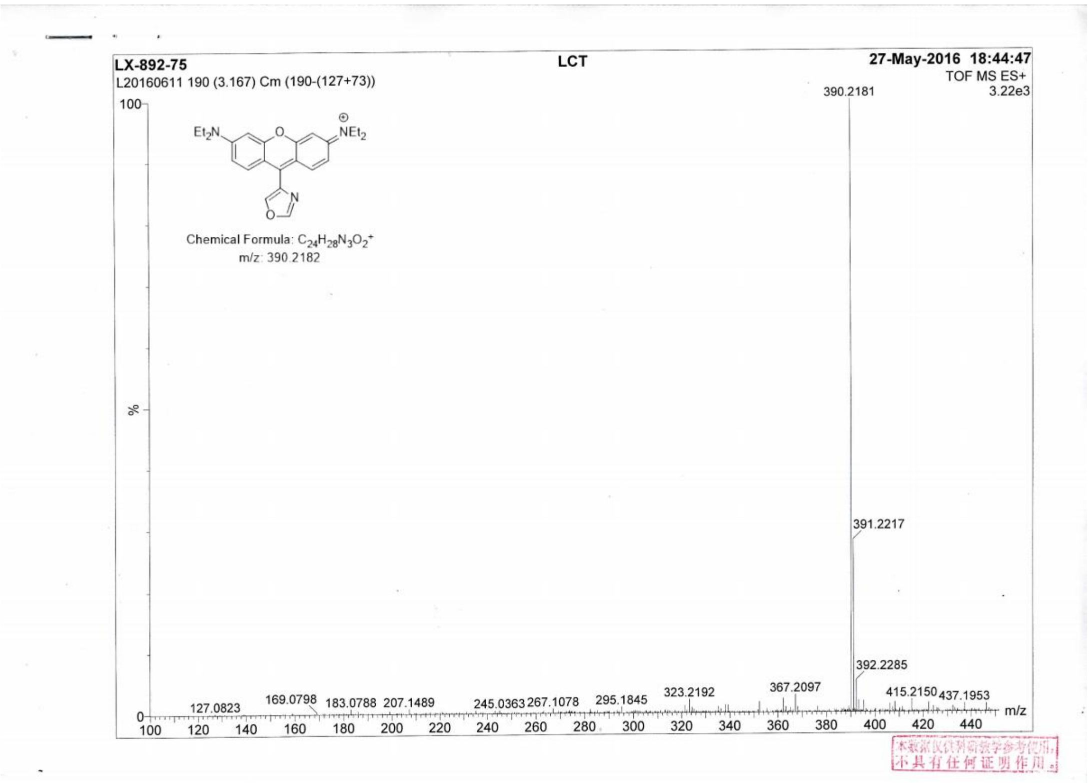

Supplementary Figure 260. The HR-MS of compound **RD37**

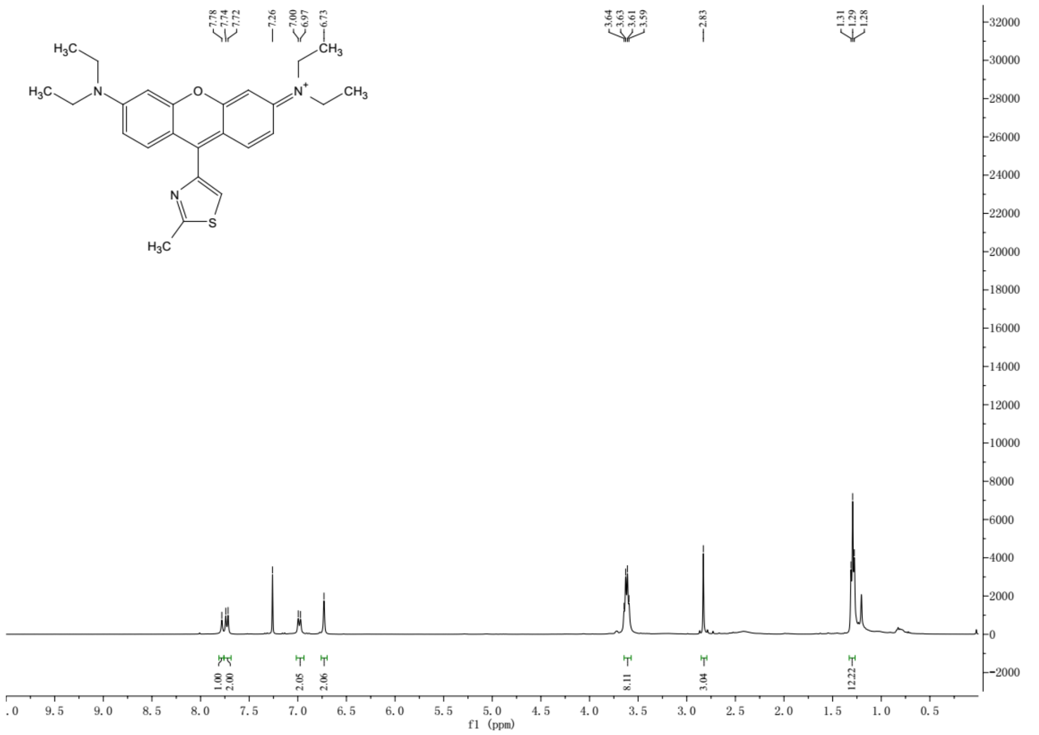

Supplementary Figure 261. The ^{1}H-NMR of compound **RD38** in $CDCl_3$

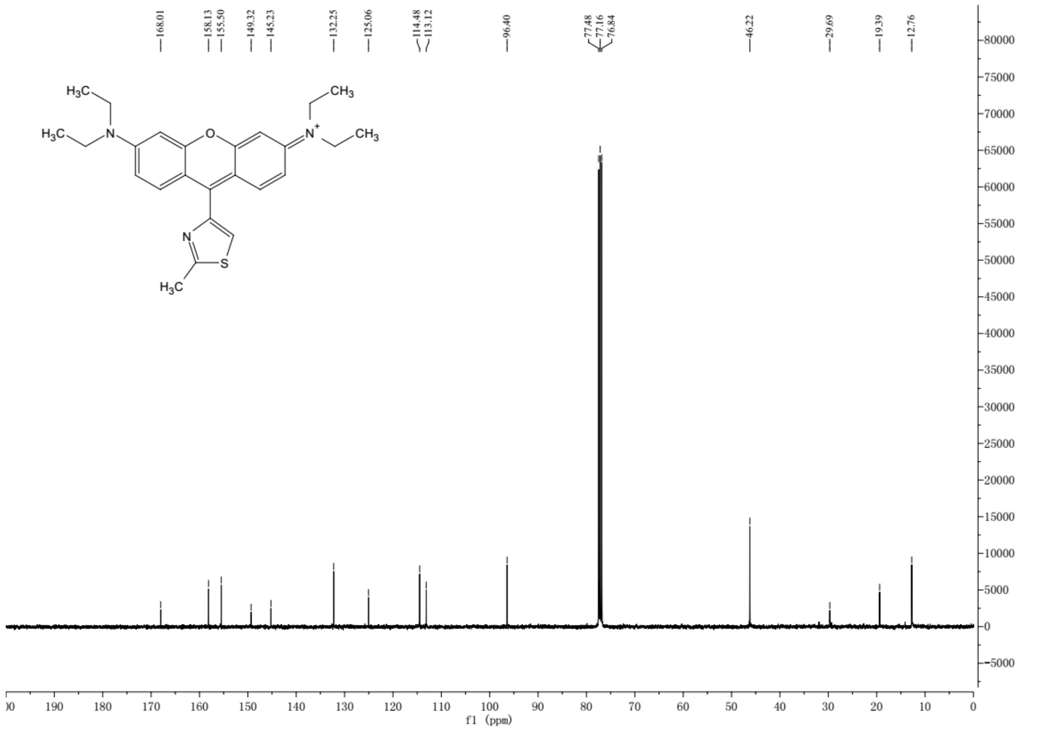

Supplementary Figure 262. The ^{13}C-NMR of compound **RD38** in $CDCl_3$

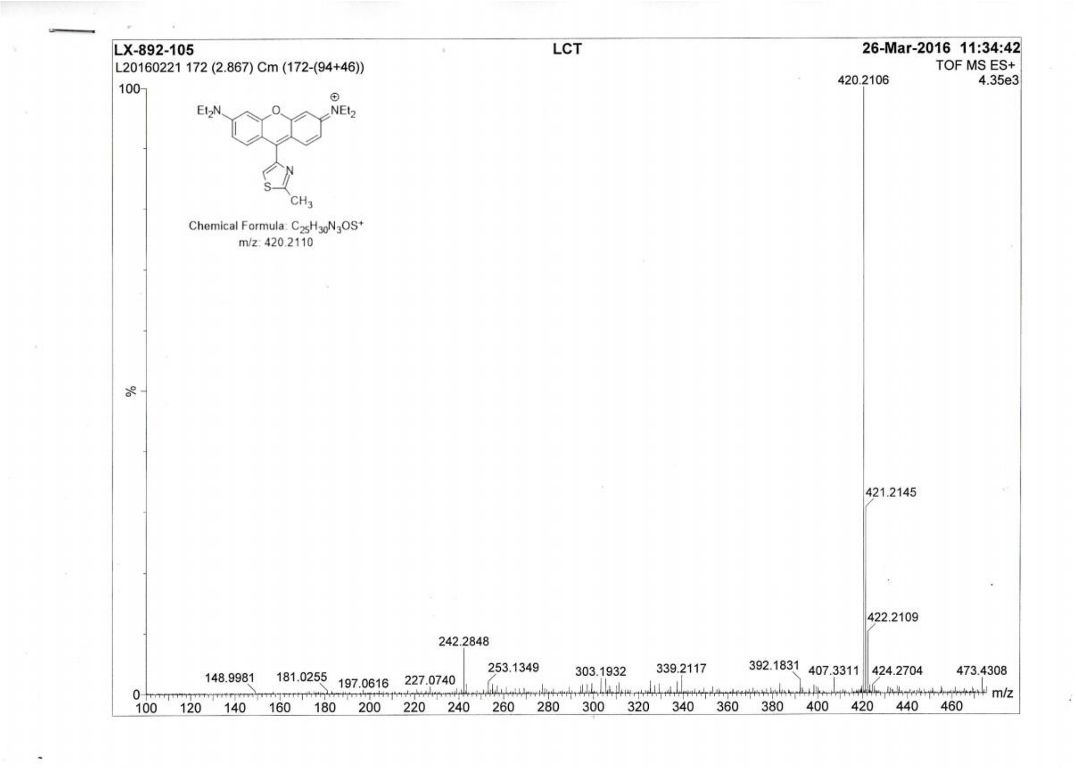

Supplementary Figure 263. The HR-MS of compound **RD38**

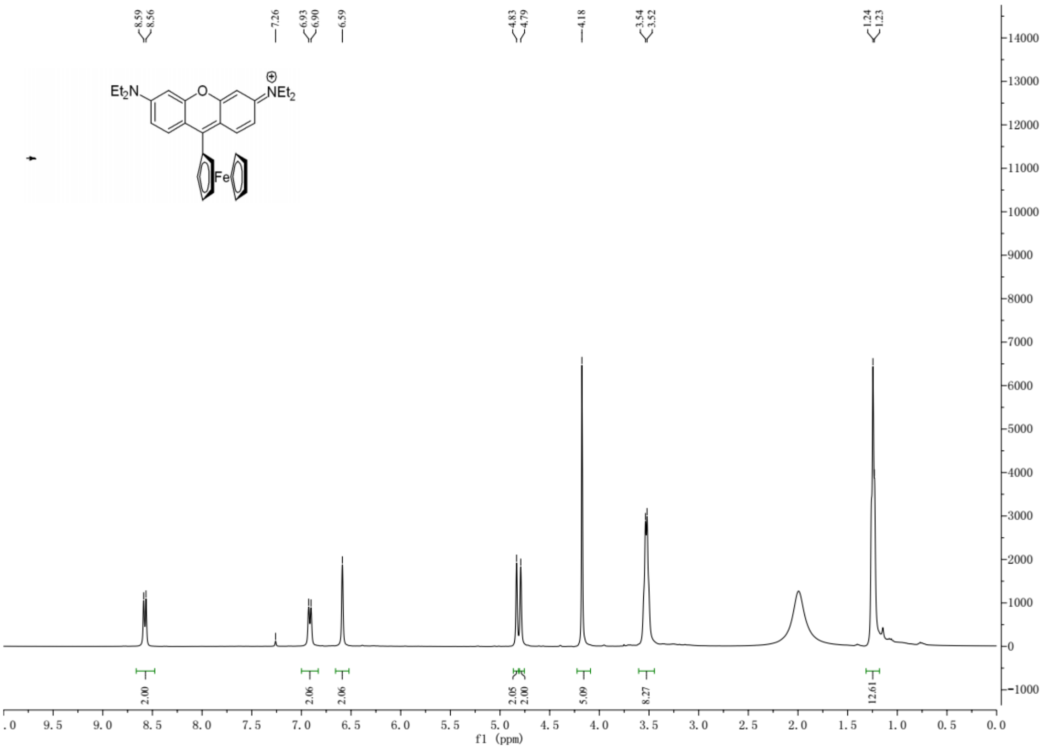

Supplementary Figure 264. The ^{1}H-NMR of compound **RD39** in $CDCl_3$

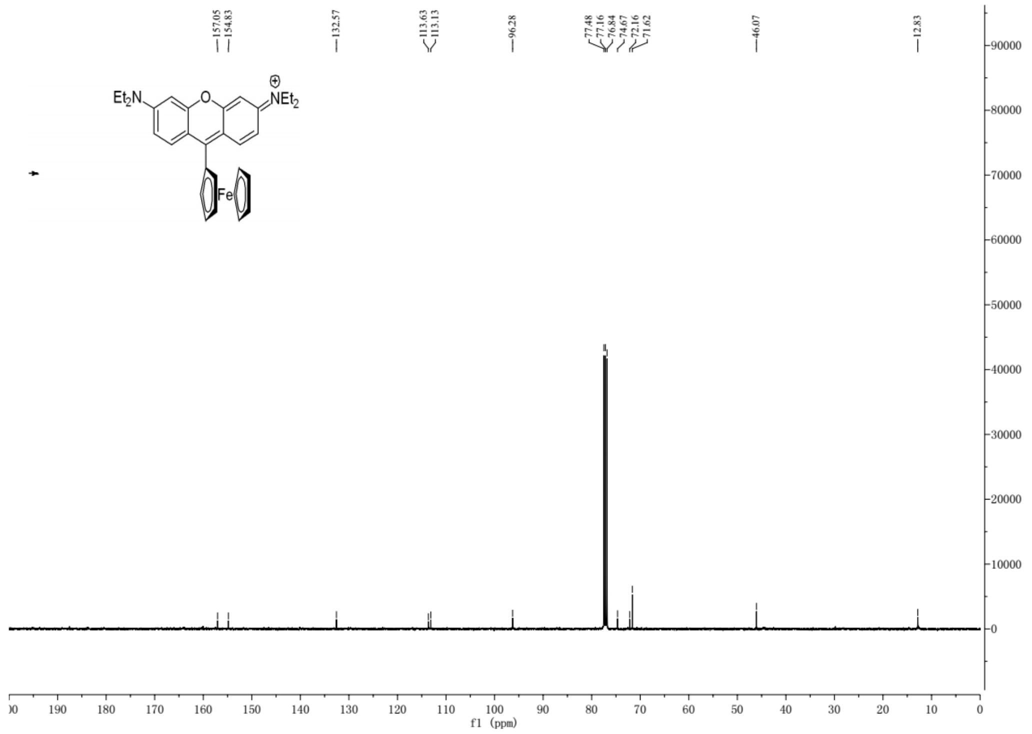

Supplementary Figure 265. The ^{13}C-NMR of compound **RD39** in $CDCl_3$

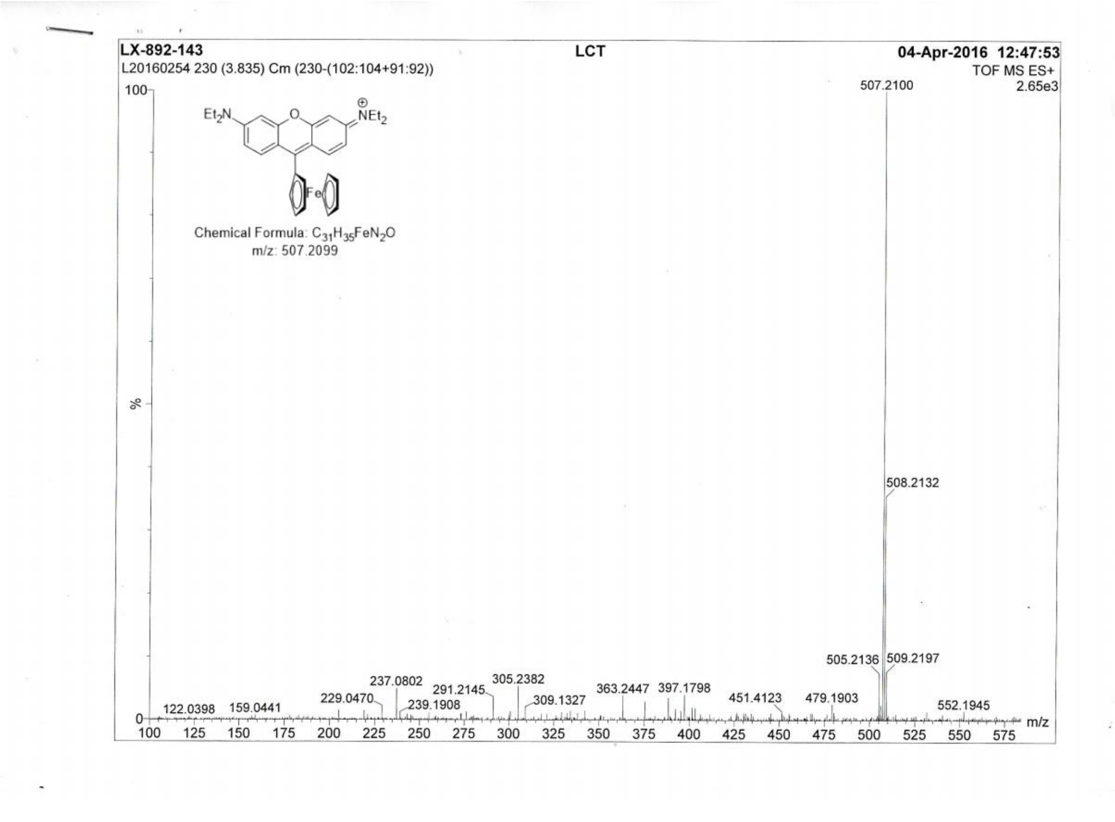

Supplementary Figure 266. The HR-MS of compound **RD39**

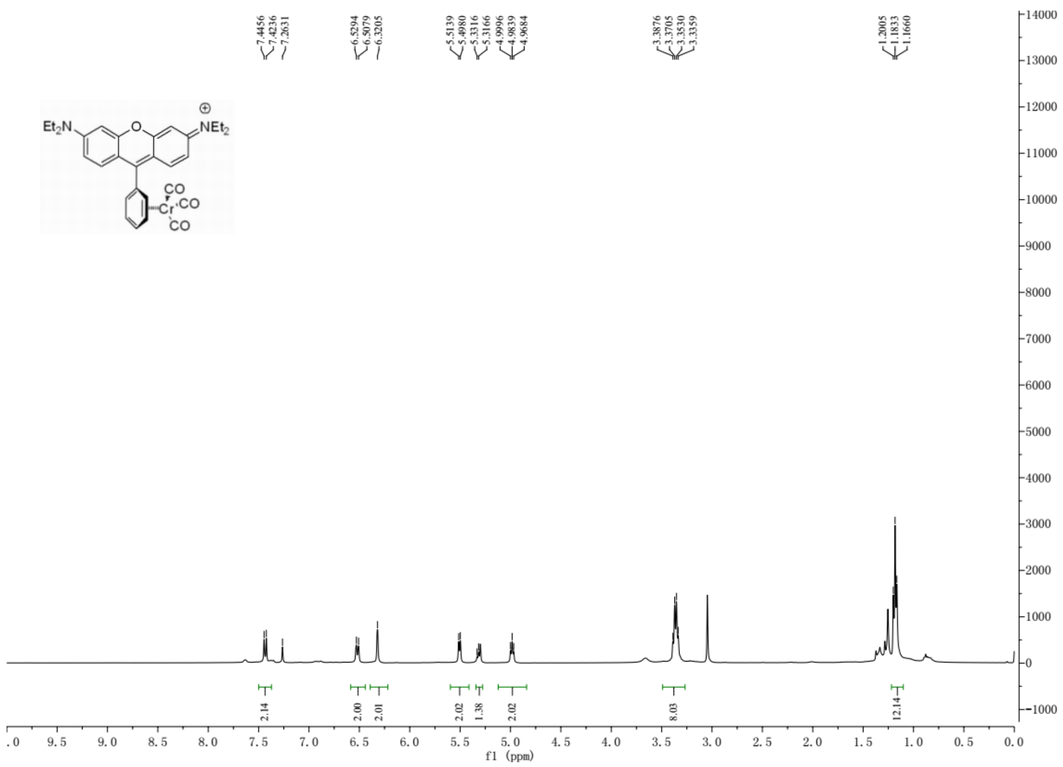

Supplementary Figure 267. The ^{1}H-NMR of compound RD40 in $CDCl_3$

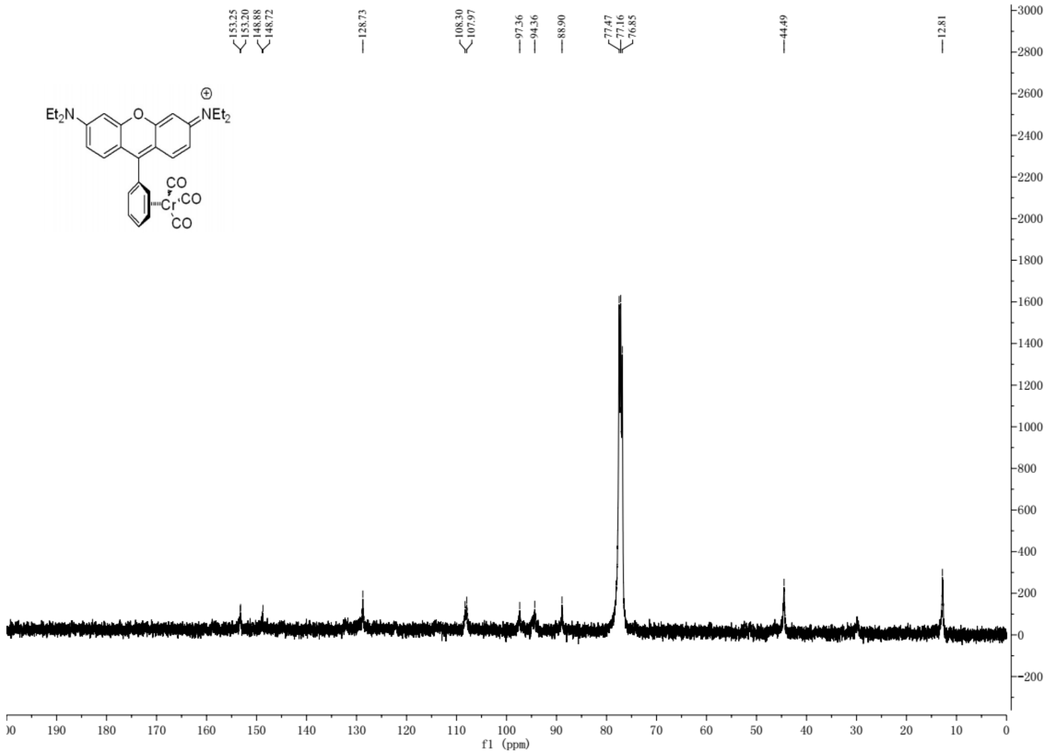

Supplementary Figure 268. The ^{13}C-NMR of compound RD40 in $CDCl_3$

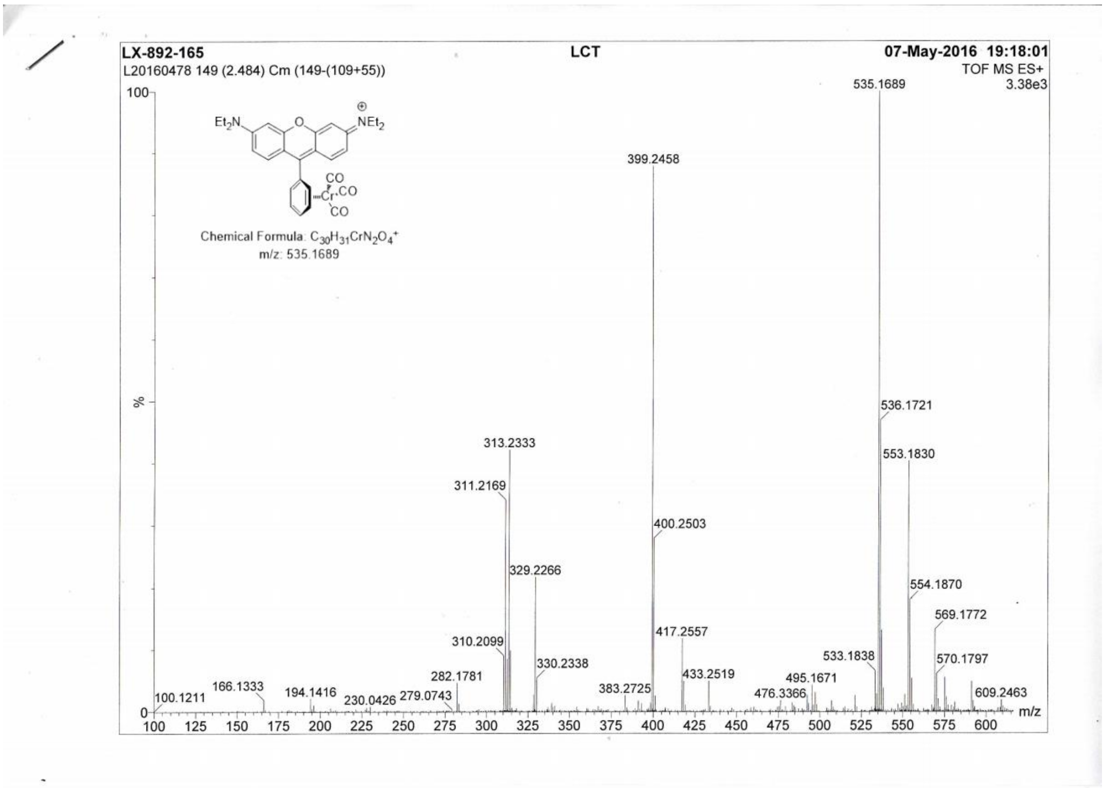

Supplementary Figure 269. The HR-MS of compound **RD40**

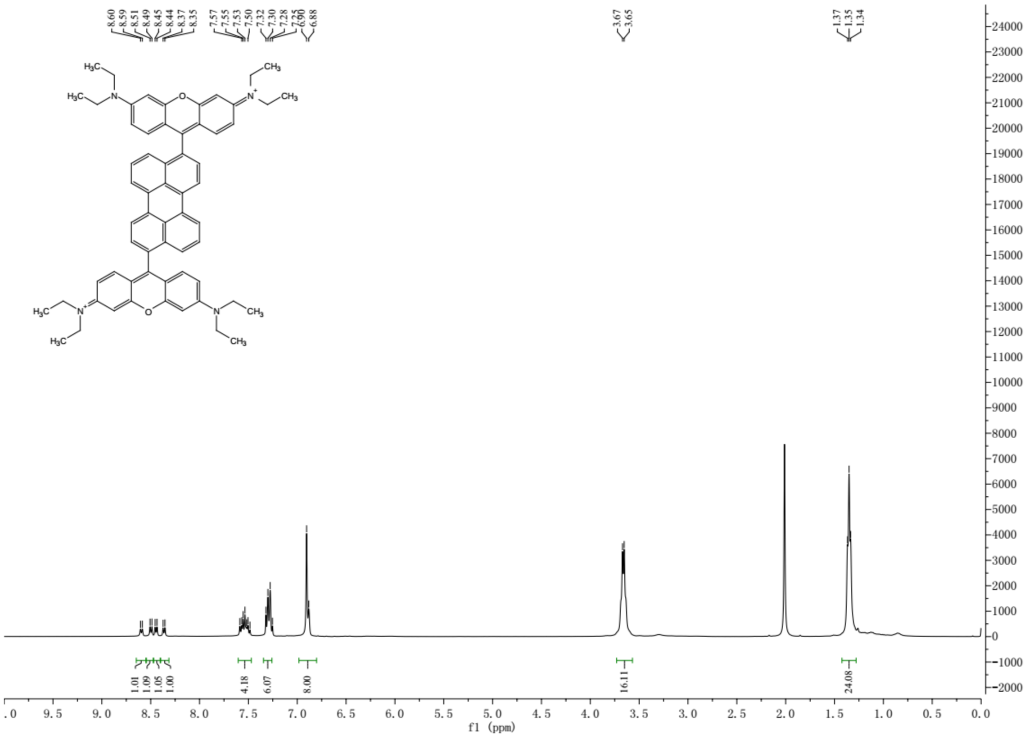

Supplementary Figure 270. The ^{1}H-NMR of compound **RD41** in $CDCl_3$

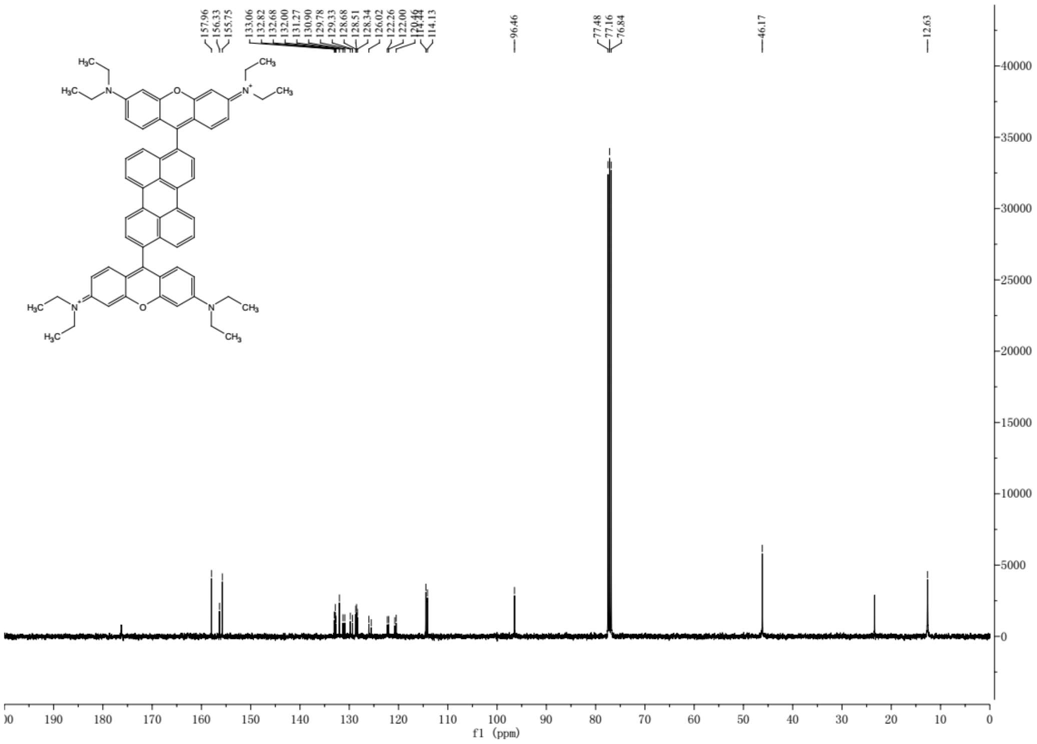

Supplementary Figure 271. The ^{13}C-NMR of compound **RD41** in $CDCl_3$

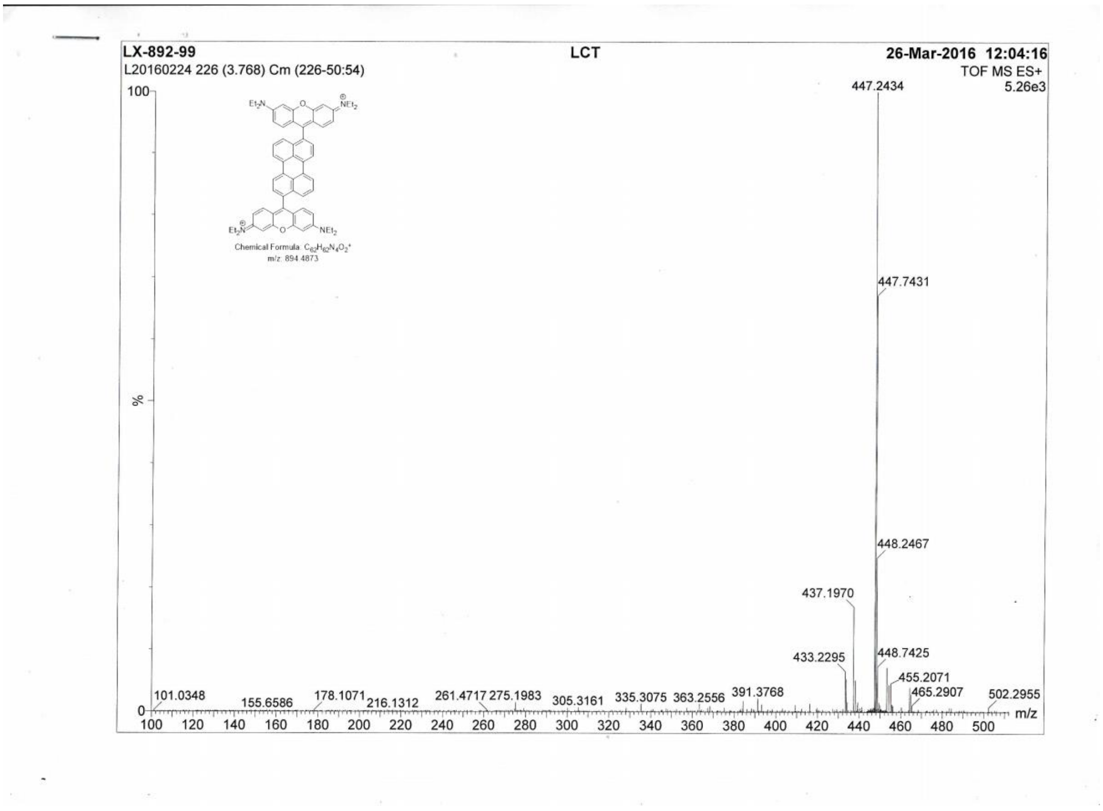

Supplementary Figure 272. The HR-MS of compound **RD41**

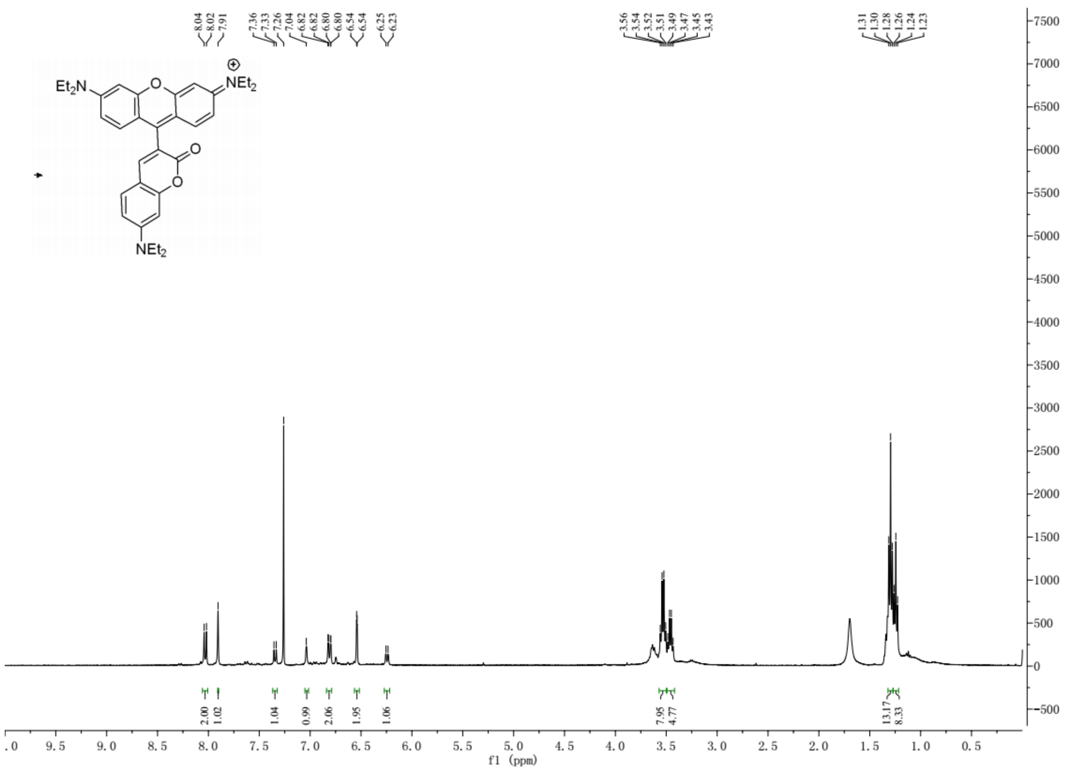

Supplementary Figure 273. The ^{1}H-NMR of compound **RD42** in $CDCl_3$

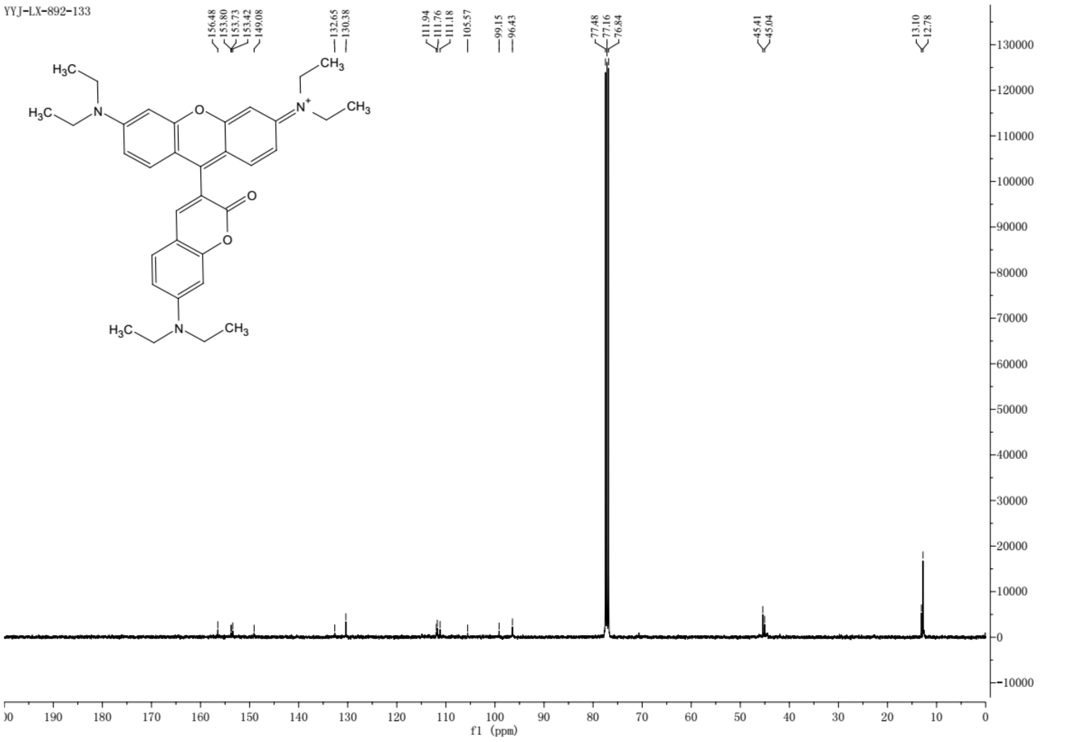

Supplementary Figure 274. The ^{13}C-NMR of compound **RD42** in $CDCl_3$

Elemental Composition Report | **Page 1**

Multiple Mass Analysis: 2 mass(es) processed
Tolerance = 5.0 PPM / DBE: min = -1.5, max = 100.0
Isotope cluster parameters: Separation = 1.0 Abundance = 1.0%

Monoisotopic Mass, Odd and Even Electron Ions
5557 formula(e) evaluated with 8 results within limits (up to 50 closest results for each mass)

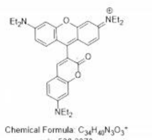

LX-892-133 | LCT | 04-Apr-2016 12:12:23
L20160251 212 (3.534) Cm (212-(127+92)) | TOF MS ES+ 458

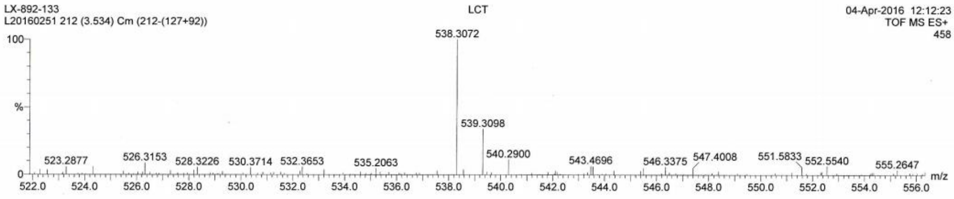

Mass	RA	Calc. Mass	mDa	PPM	DBE	Score	Formula
Minimum:	20.00				-1.5		
Maximum:	100.00		5.0	5.0	100.0		
538.3072	100.00	538.3070	0.2	0.4	16.5	n/a	12C34 H40 N3 O3
		538.3065	0.7	1.3	21.0	n/a	12C38 13C H39 N O
		538.3083	-1.1	-2.1	16.0	n/a	12C36 H42 O4
		538.3052	2.0	3.8	21.5	n/a	12C36 13C H37 N4
		538.3096	-2.4	-4.5	21.0	n/a	12C37 H38 N4
539.3098	33.76	539.3103	-0.5	-1.0	16.5	n/a	12C33 13C H40 N3 O3
		539.3108	-1.0	-1.8	12.0	n/a	12C29 H41 N5 O5
		539.3117	-1.9	-3.5	16.0	n/a	12C35 13C H42 O4

Supplementary Figure 275. The HR-MS of compound **RD42**

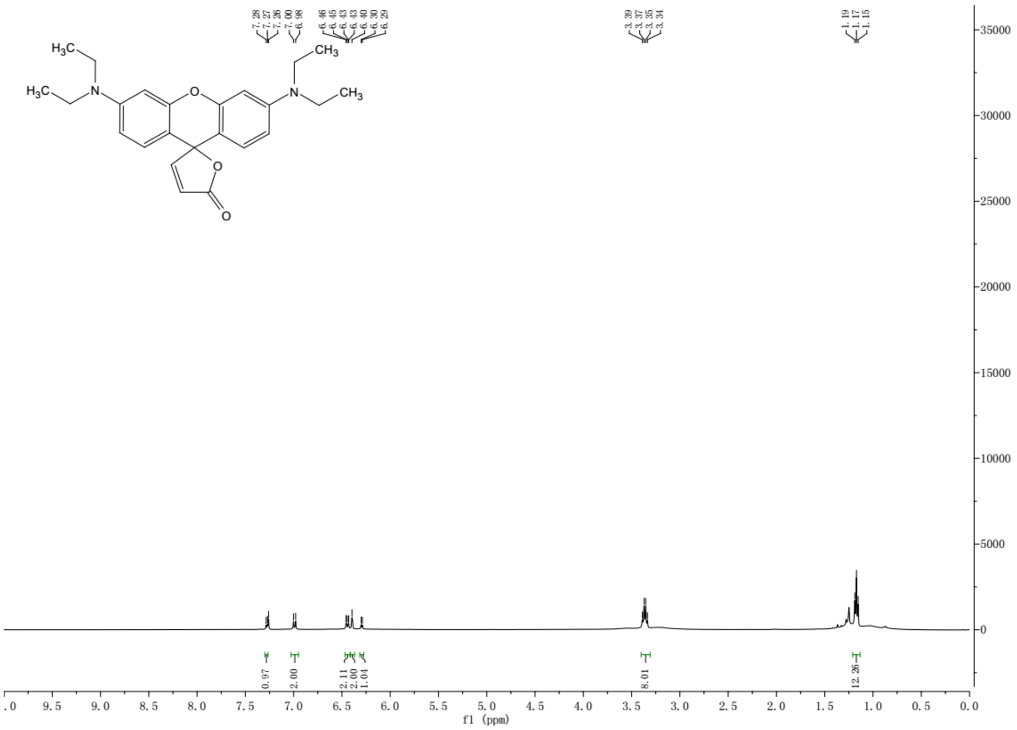

Supplementary Figure 276. The ^{1}H-NMR of compound **RD43** in $CDCl_3$

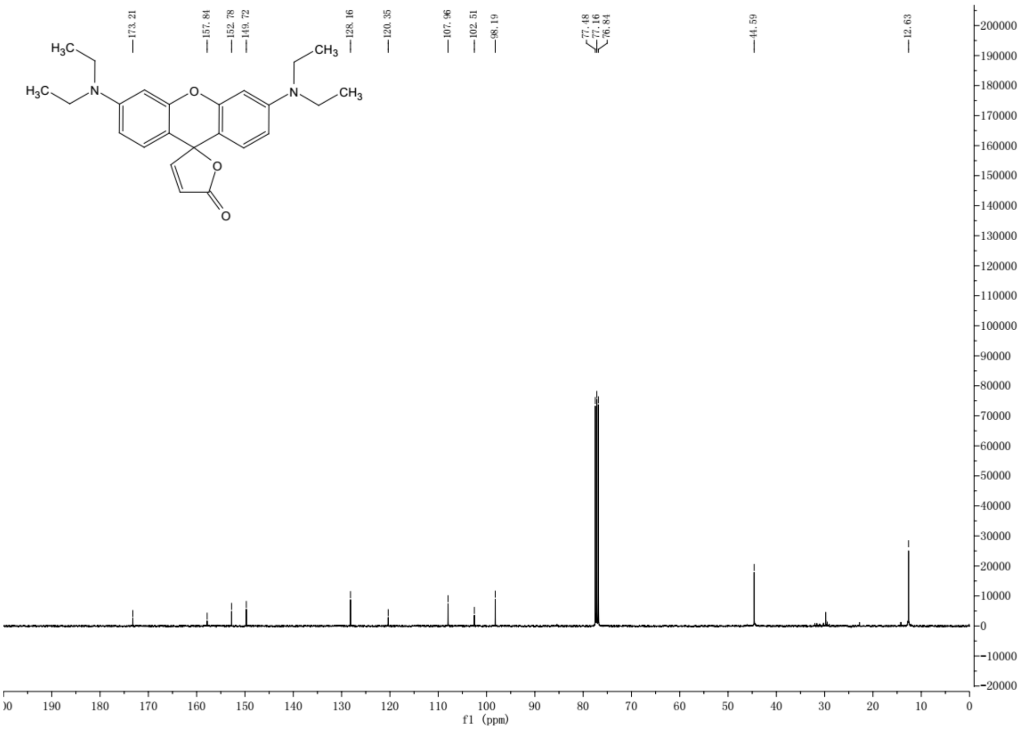

Supplementary Figure 277. The ^{13}C-NMR of compound **RD43** in $CDCl_3$

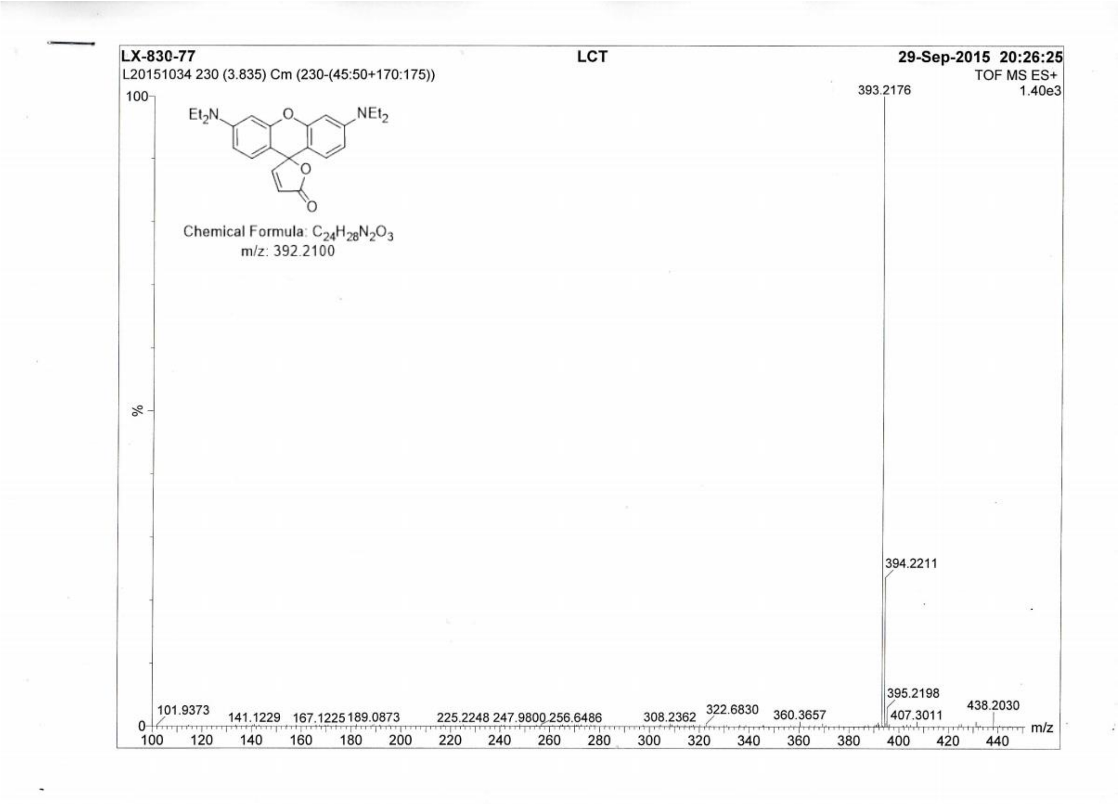

Supplementary Figure 278. The HR-MS of compound **RD43**

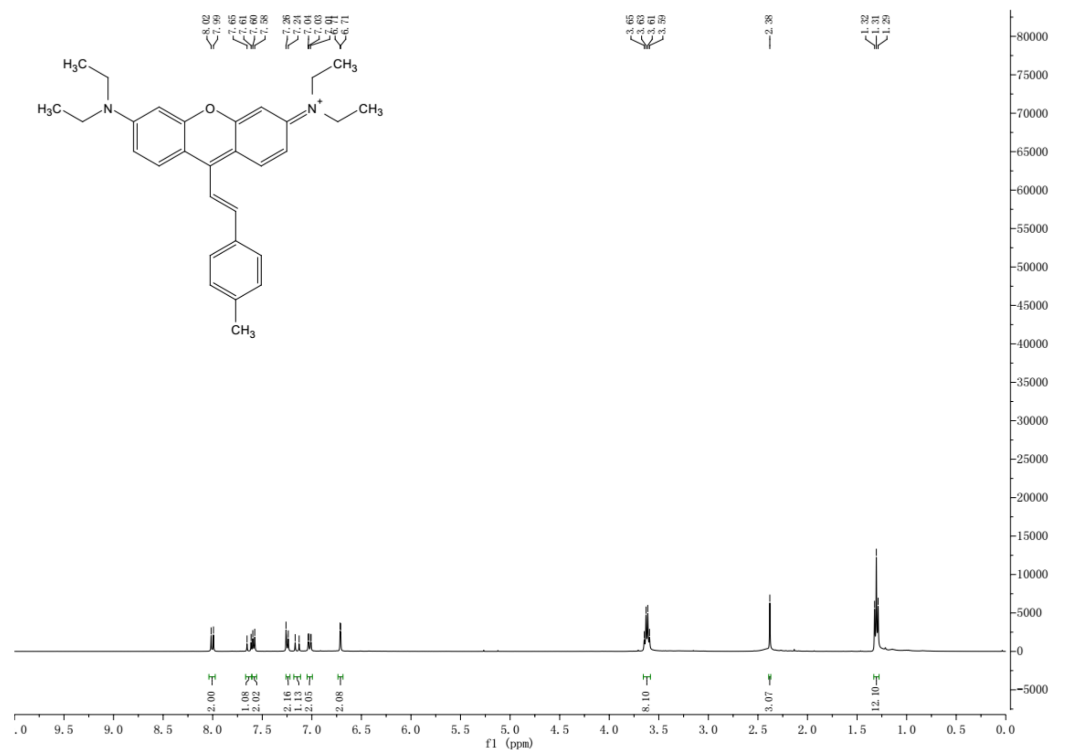

Supplementary Figure 279. The ^{1}H-NMR of compound **RD44** in $CDCl_3$

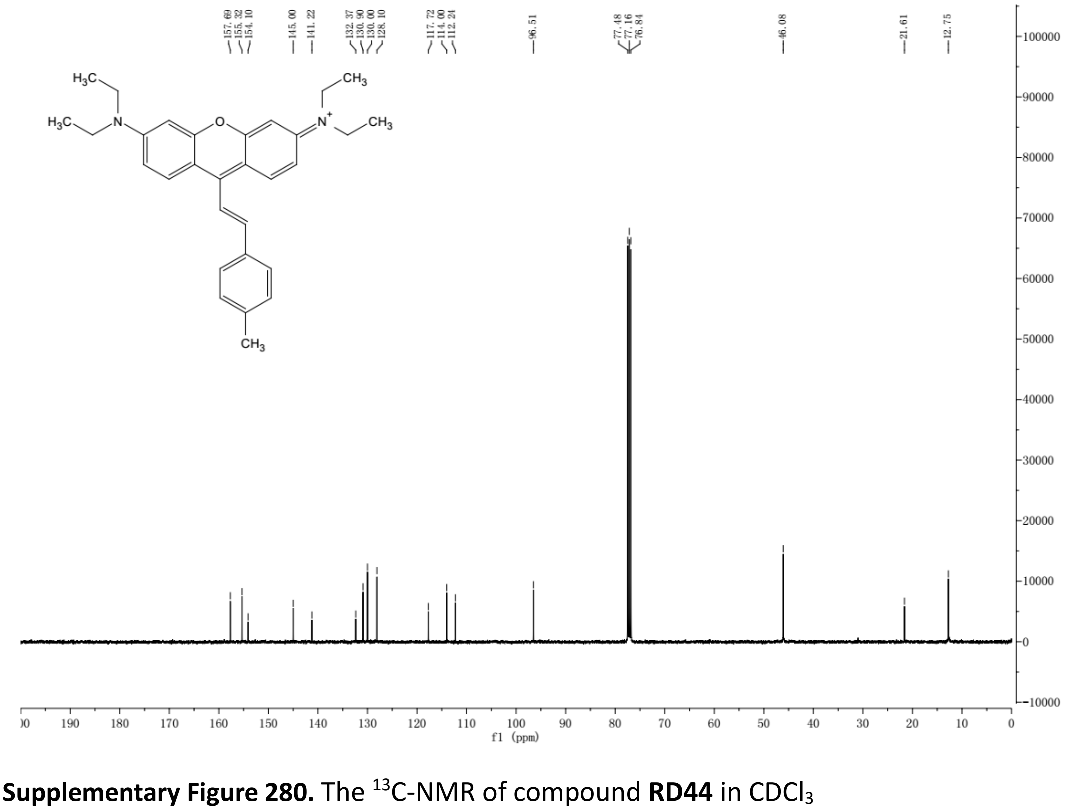

Supplementary Figure 280. The ^{13}C-NMR of compound **RD44** in $CDCl_3$

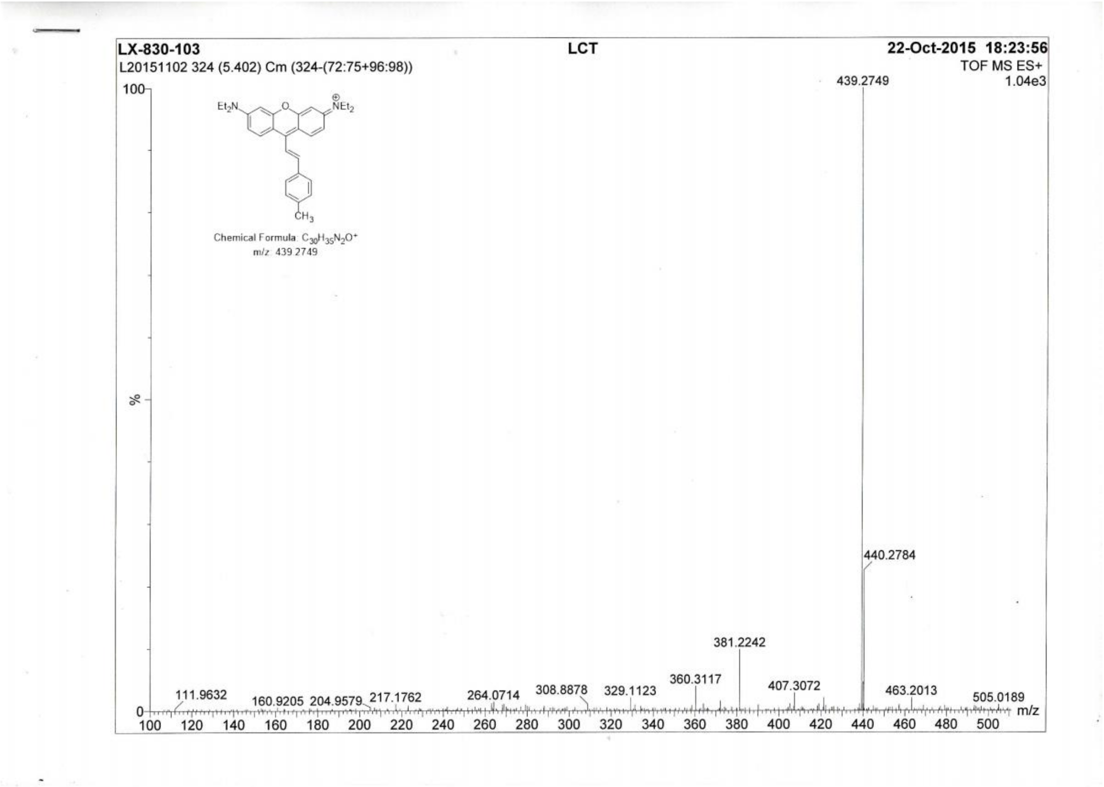

Supplementary Figure 281. The HR-MS of compound **RD44**

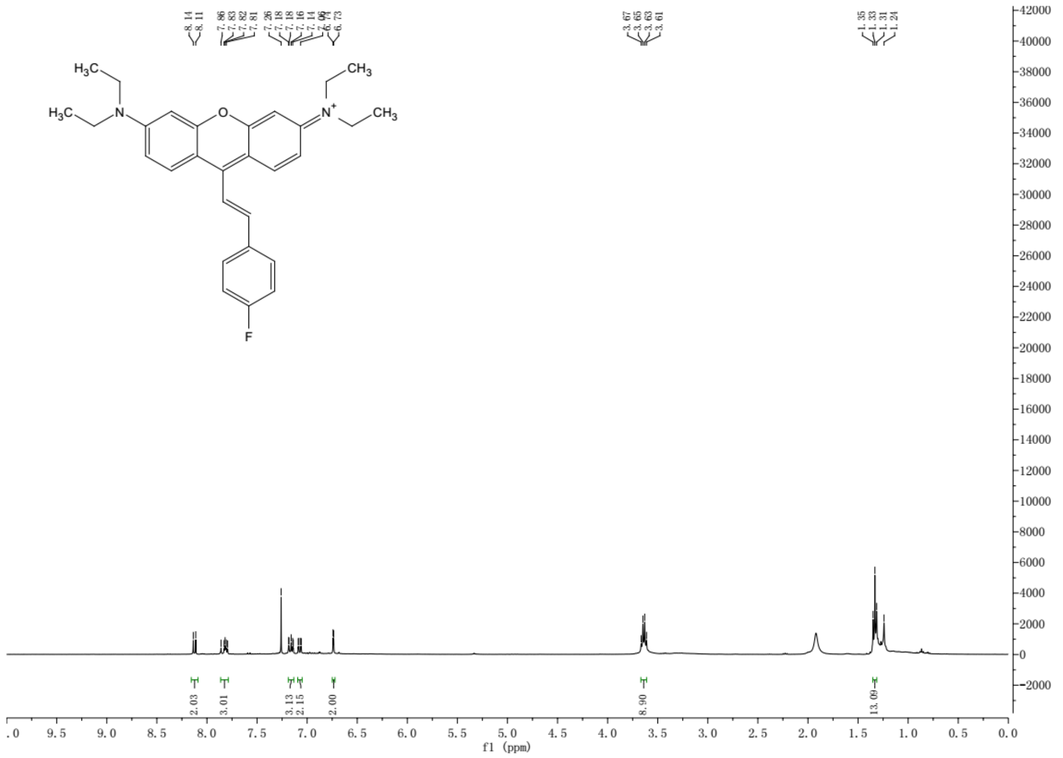

Supplementary Figure 282. The ^{1}H-NMR of compound **RD45** in $CDCl_3$

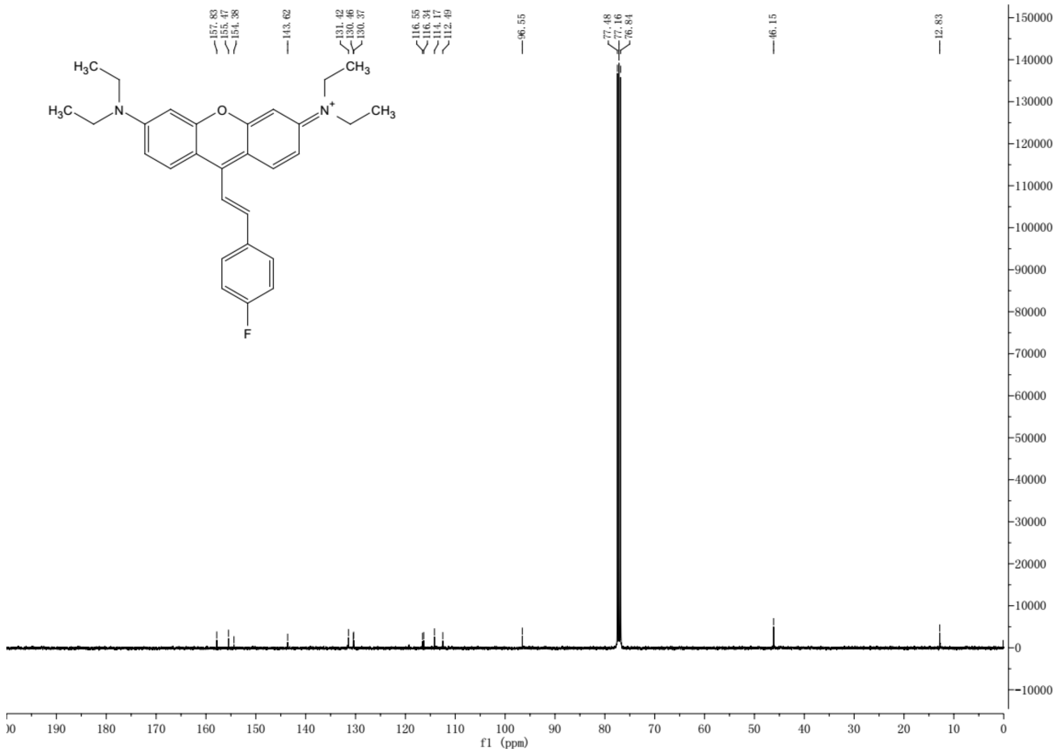

Supplementary Figure 283. The ^{13}C-NMR of compound **RD45** in $CDCl_3$

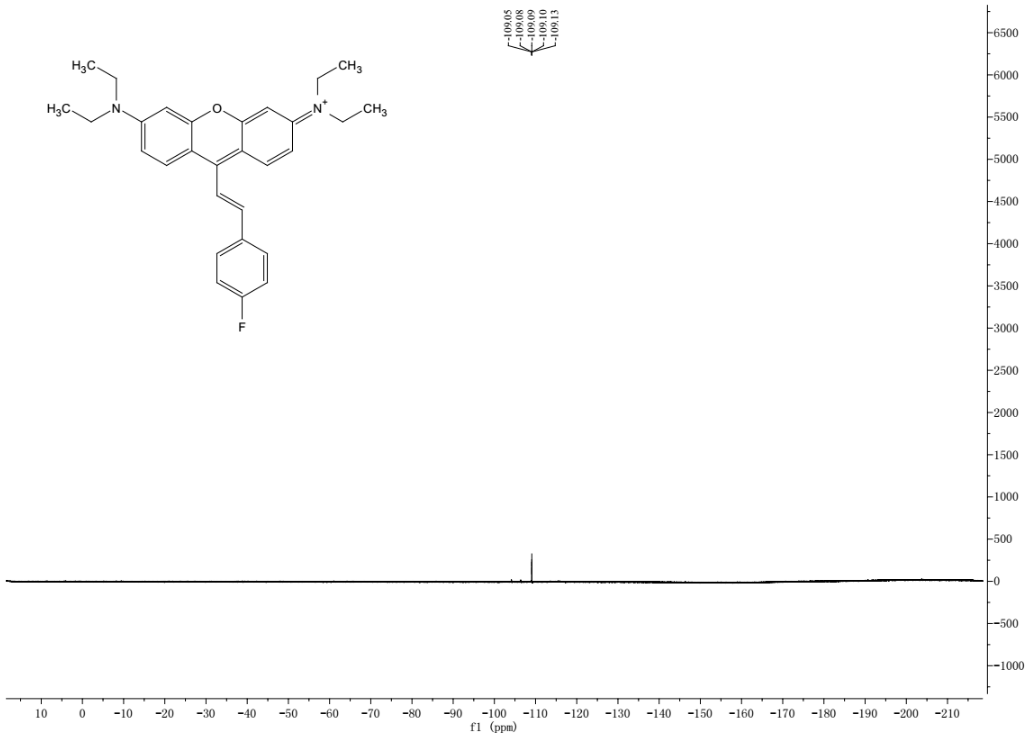

Supplementary Figure 284. The ^{19}F-NMR of compound **RD45** in $CDCl_3$

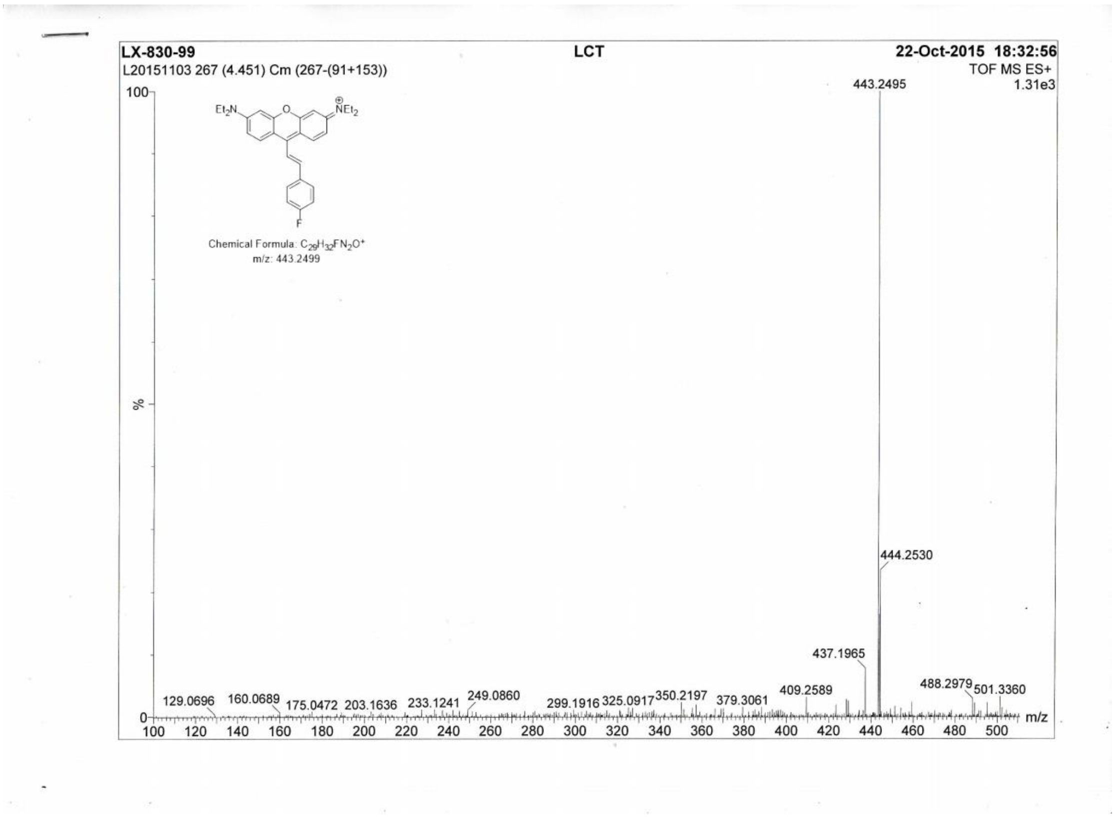

Supplementary Figure 285. The HR-MS of compound **RD45**

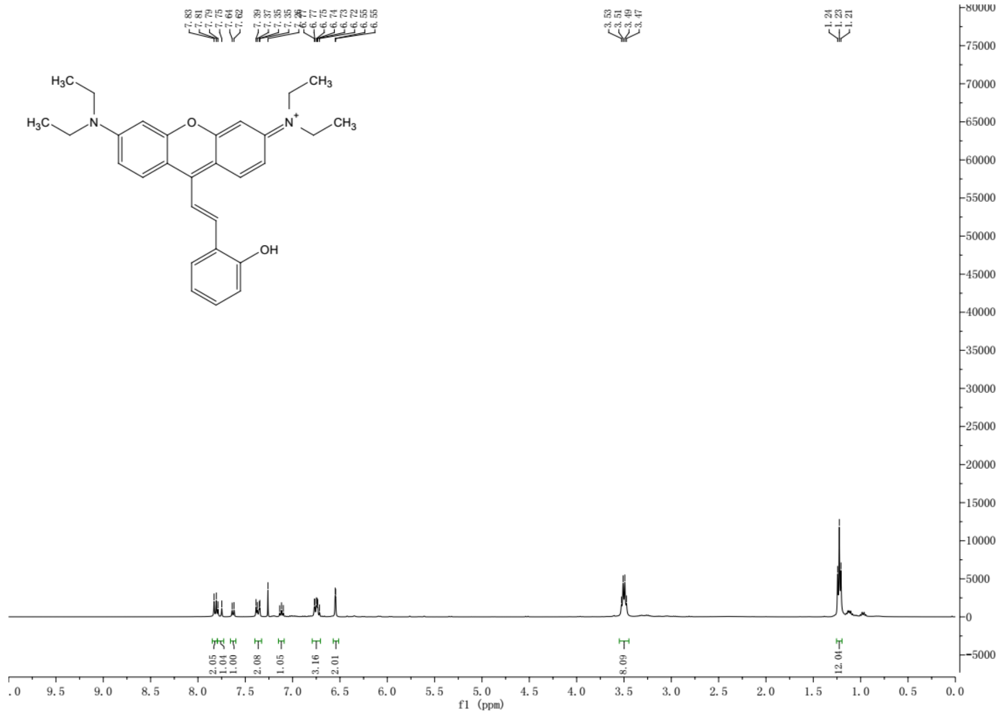

Supplementary Figure 286. The ^{1}H-NMR of compound **RD46** in $CDCl_3$

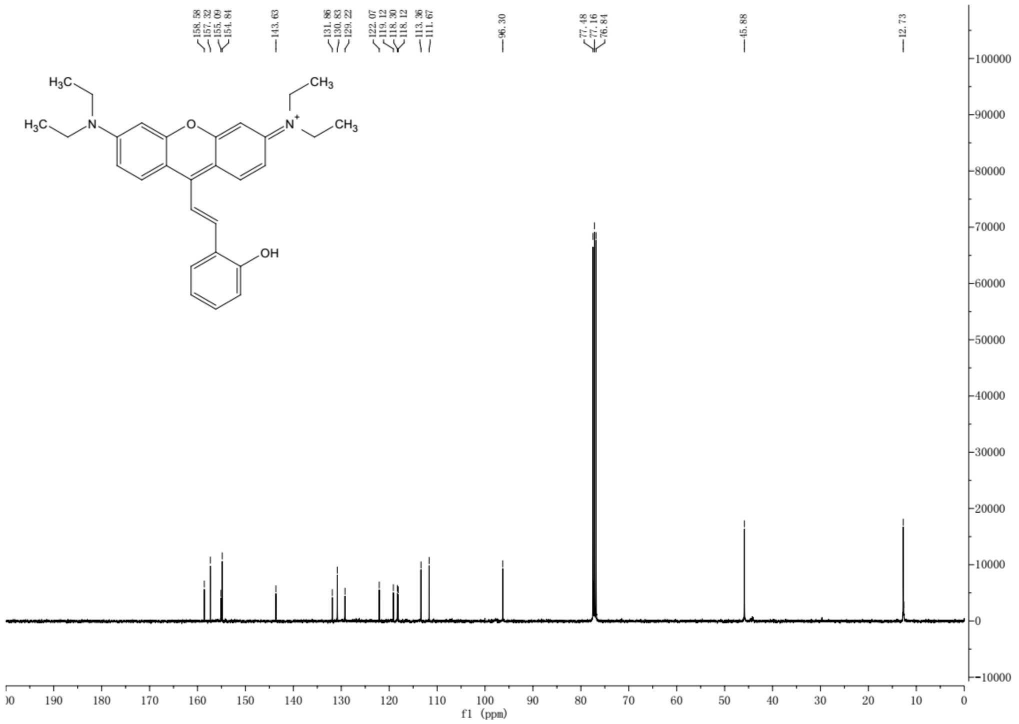

Supplementary Figure 287. The ^{13}C-NMR of compound **RD46** in $CDCl_3$

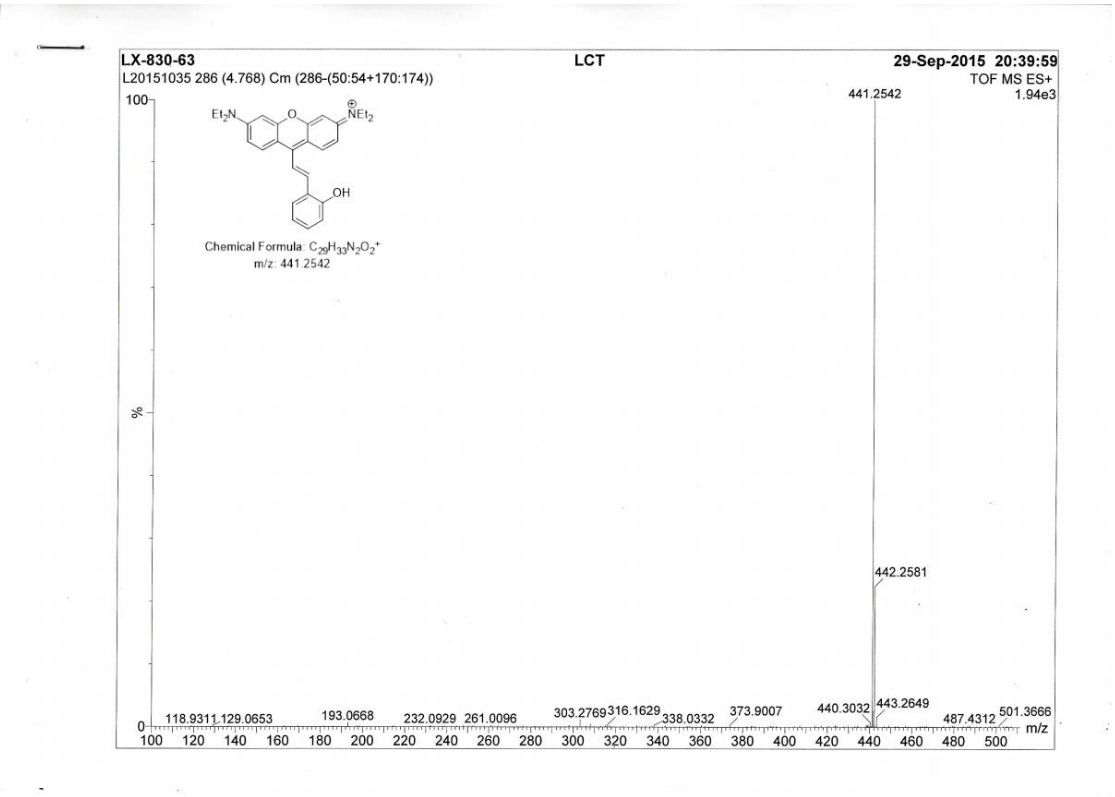

Supplementary Figure 288. The HR-MS of compound **RD46**

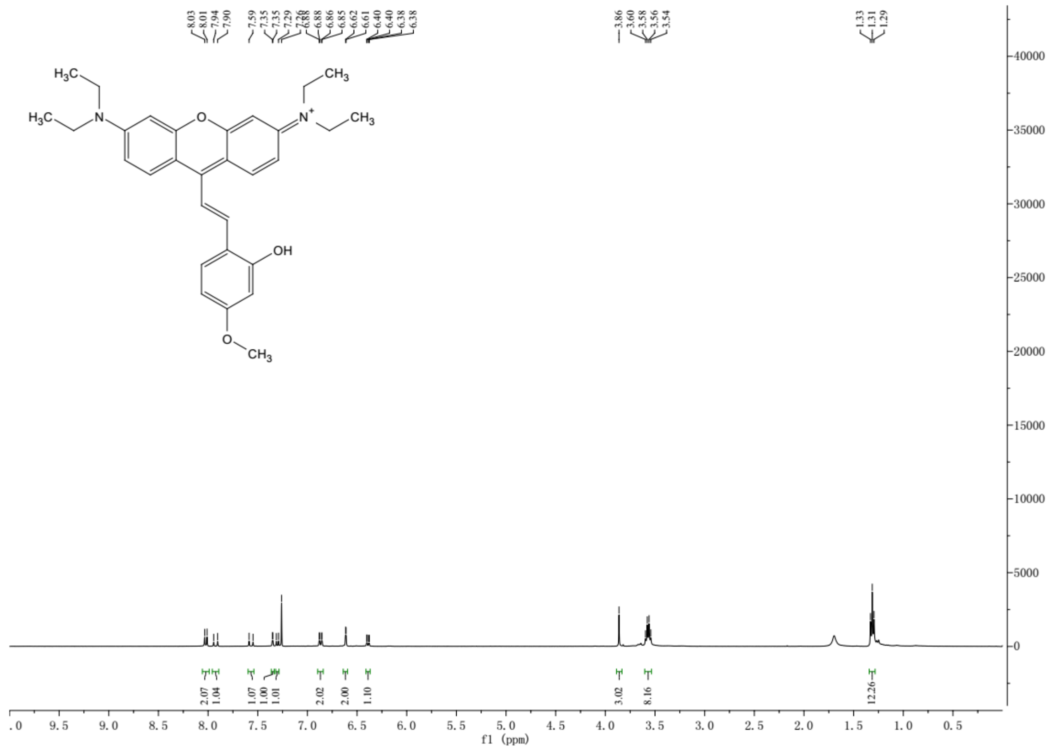

Supplementary Figure 289. The ^{1}H-NMR of compound **RD47** in $CDCl_3$

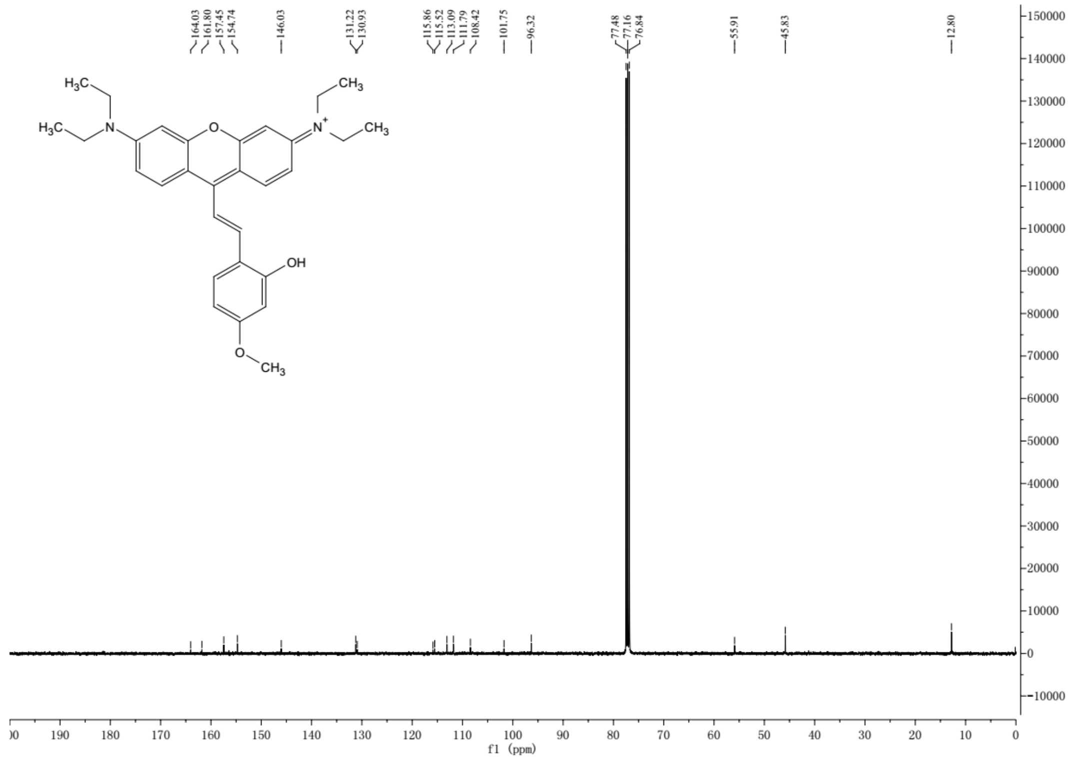

Supplementary Figure 290. The ^{13}C-NMR of compound **RD47** in $CDCl_3$

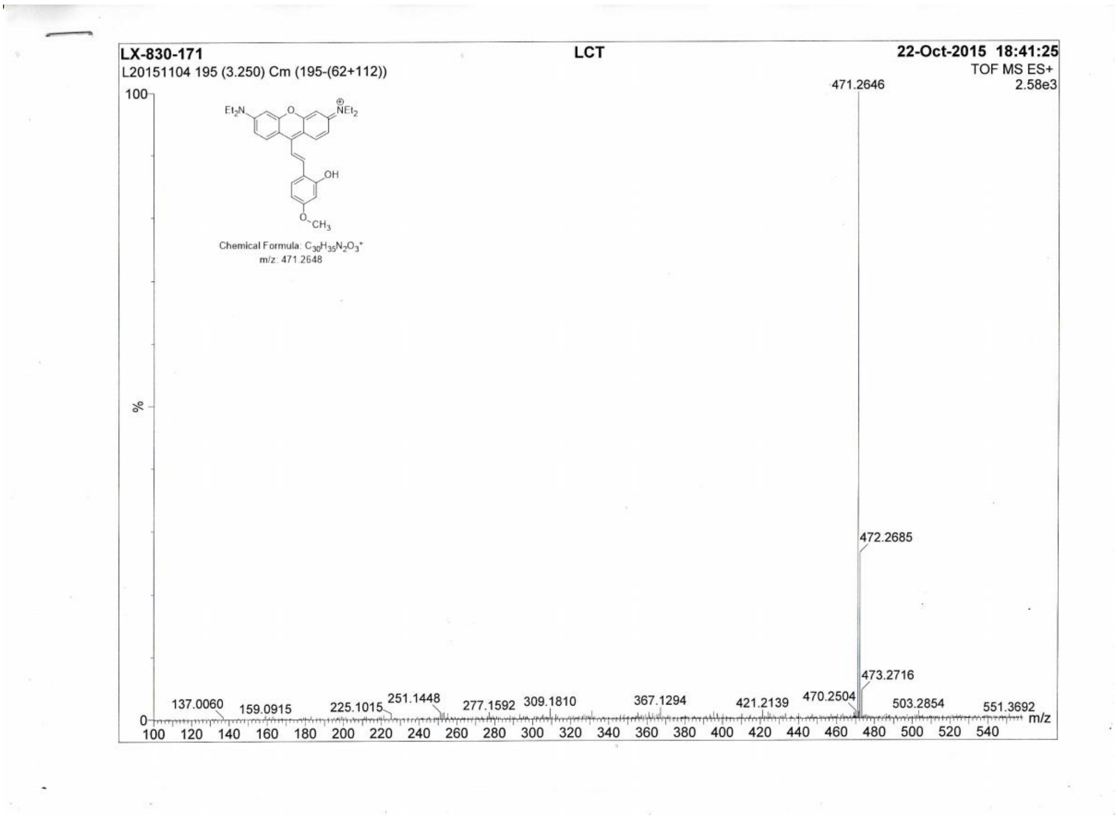

Supplementary Figure 291. The HR-MS of compound **RD47**

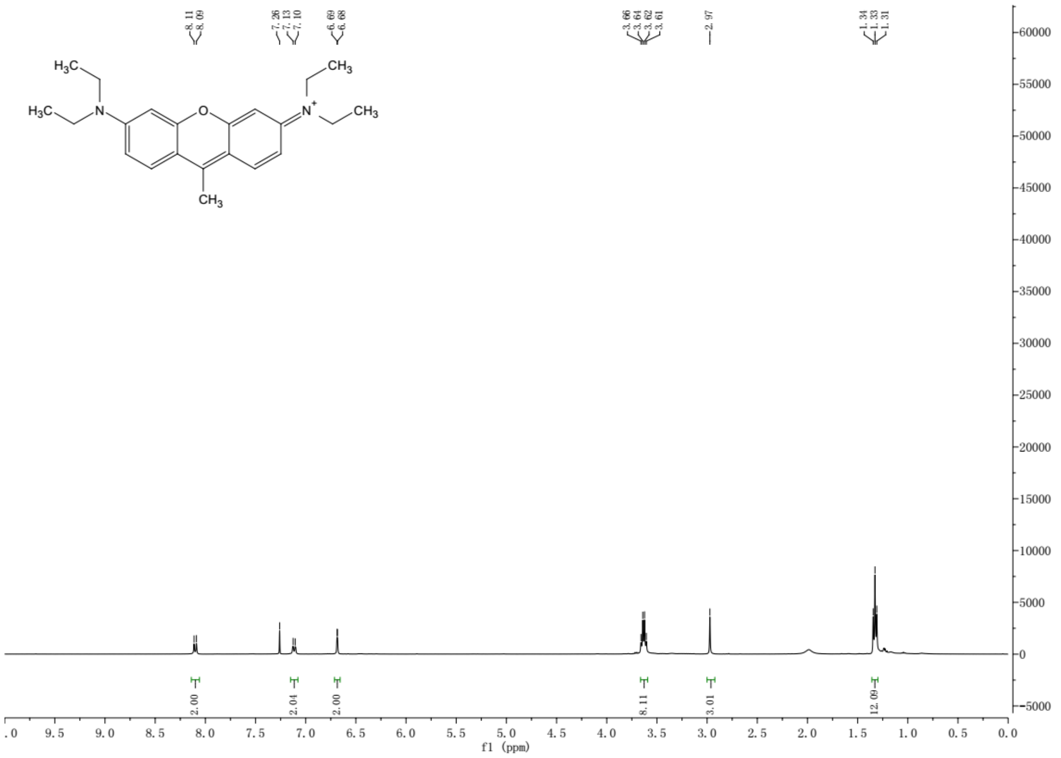

Supplementary Figure 292. The ^{1}H-NMR of compound **RD48** in $CDCl_3$

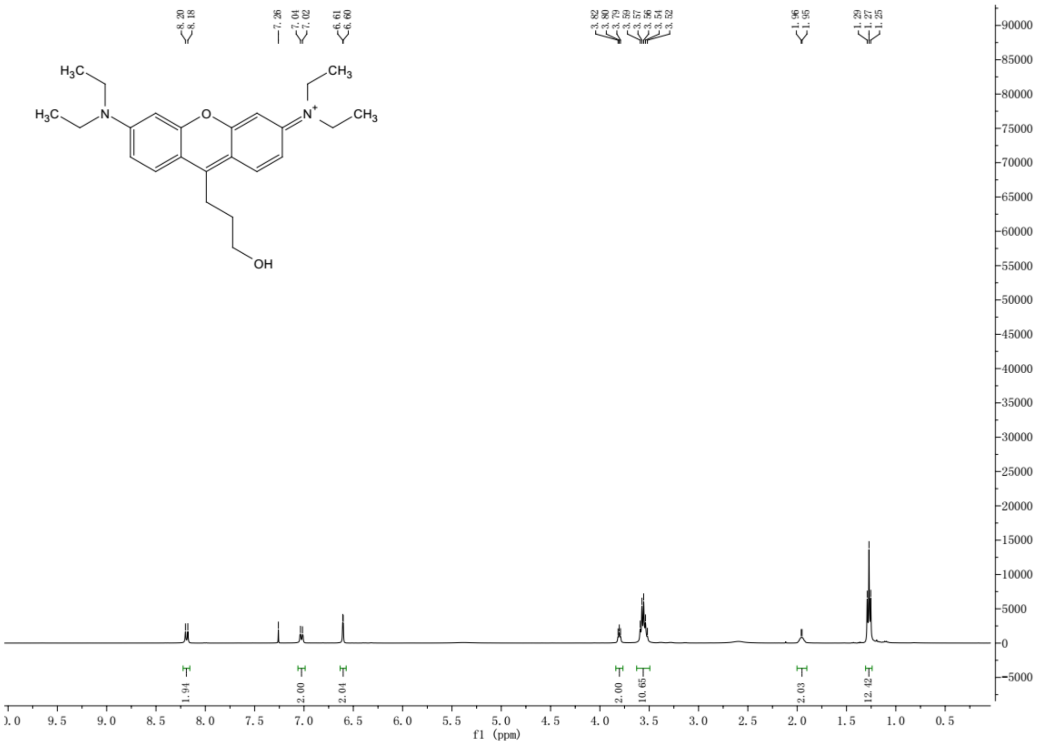

Supplementary Figure 293. The ^{1}H-NMR of compound **RD49** in $CDCl_3$

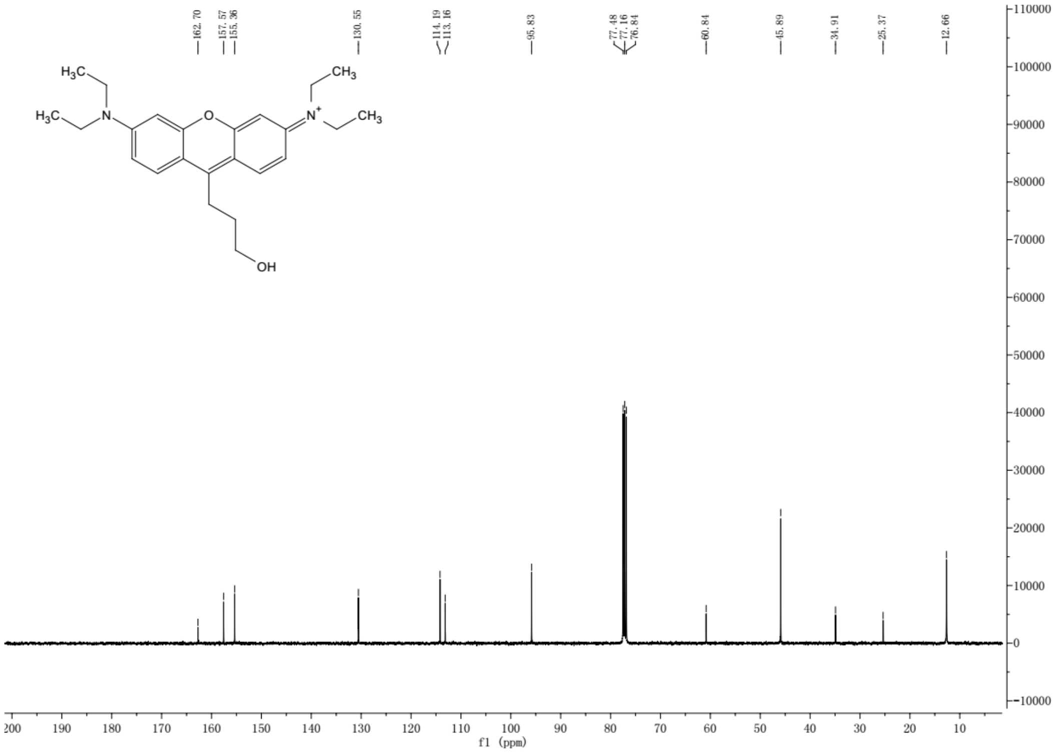

Supplementary Figure 294. The ^{13}C-NMR of compound **RD49** in $CDCl_3$

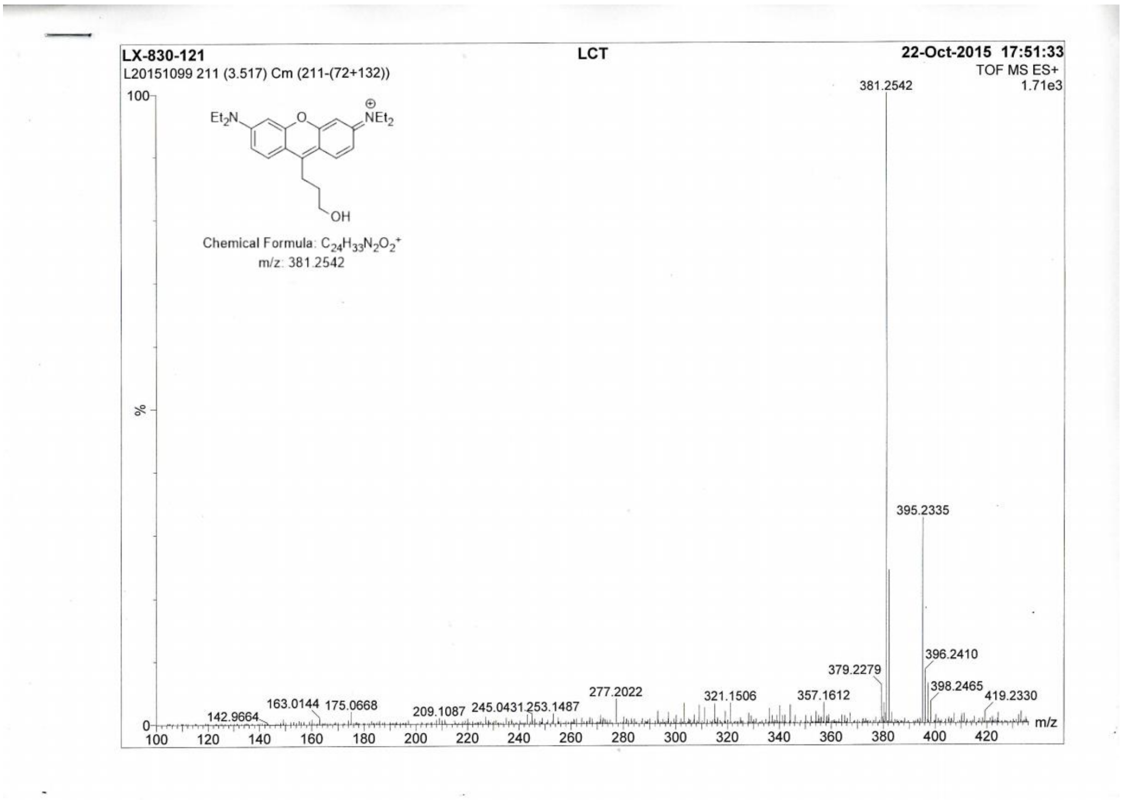

Supplementary Figure 295. The HR-MS of compound **RD49**

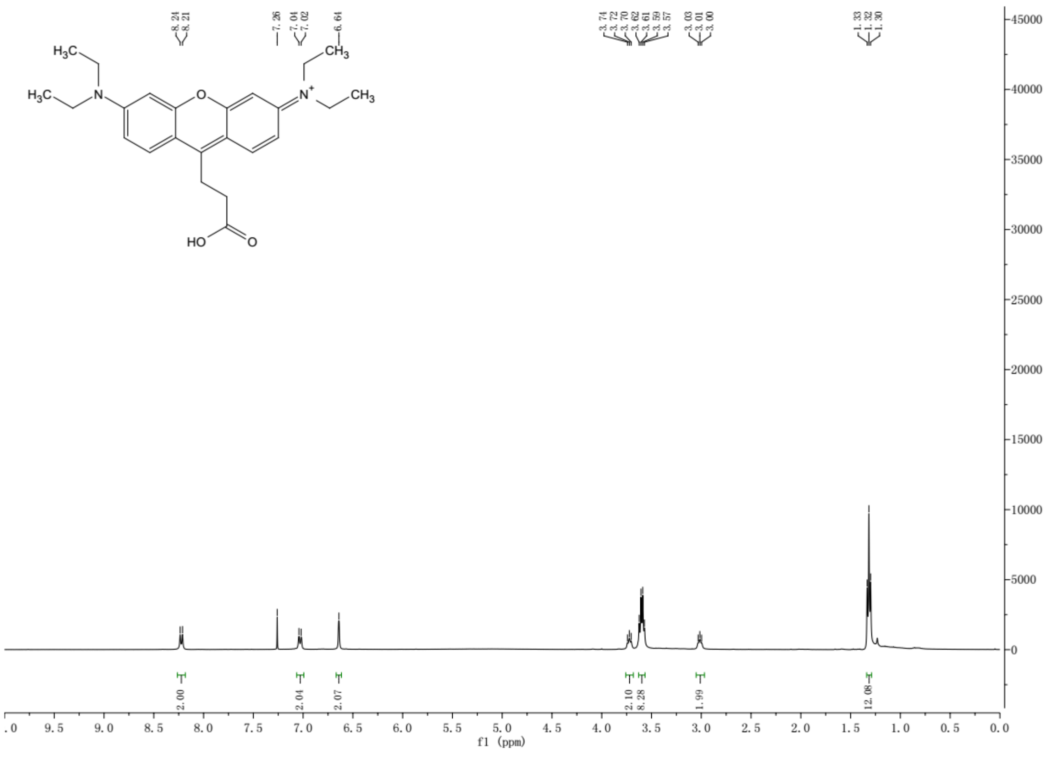

Supplementary Figure 296. The ^{1}H-NMR of compound **RD50** in $CDCl_3$

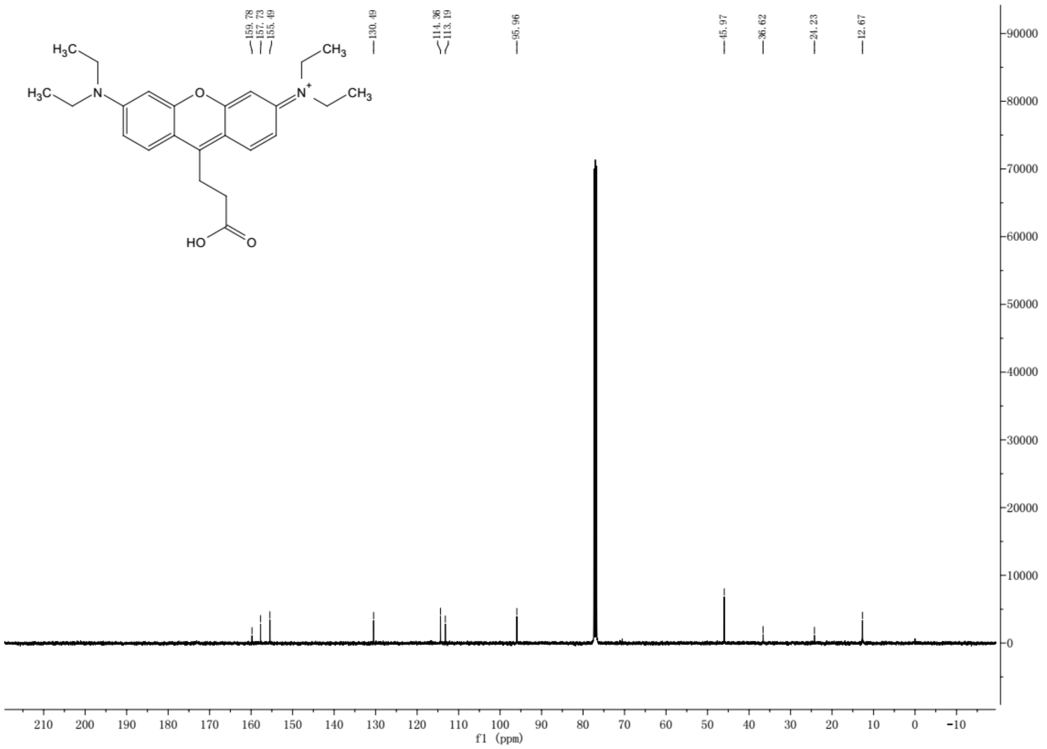

Supplementary Figure 297. The ^{13}C-NMR of compound **RD50** in $CDCl_3$

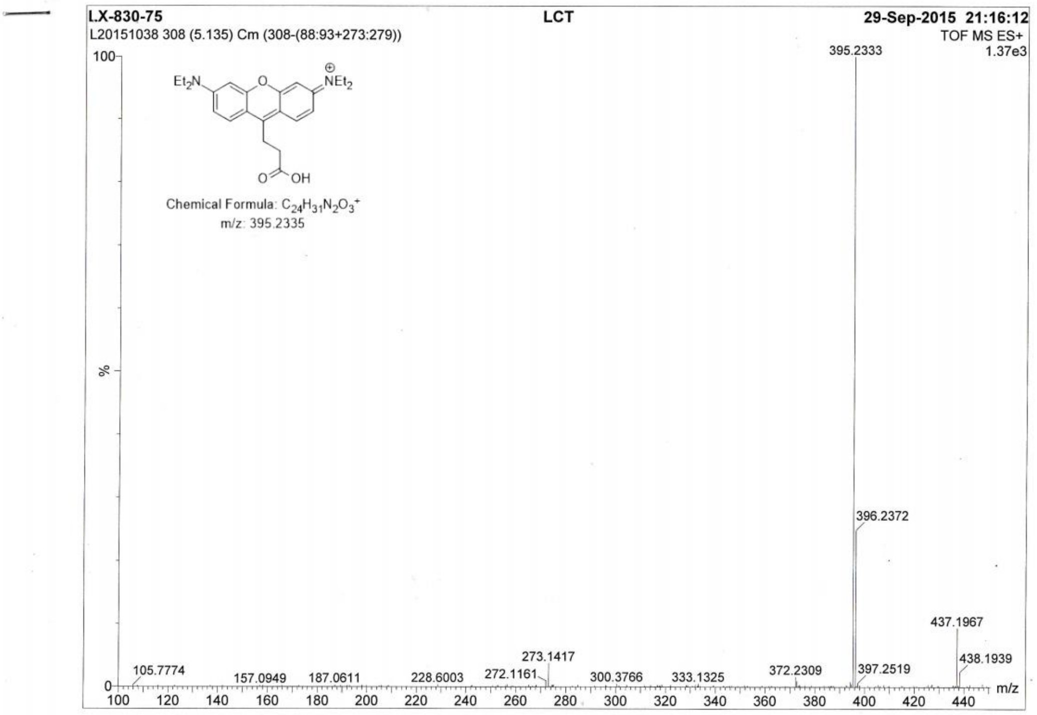

Supplementary Figure 298. The HR-MS of compound **RD50**

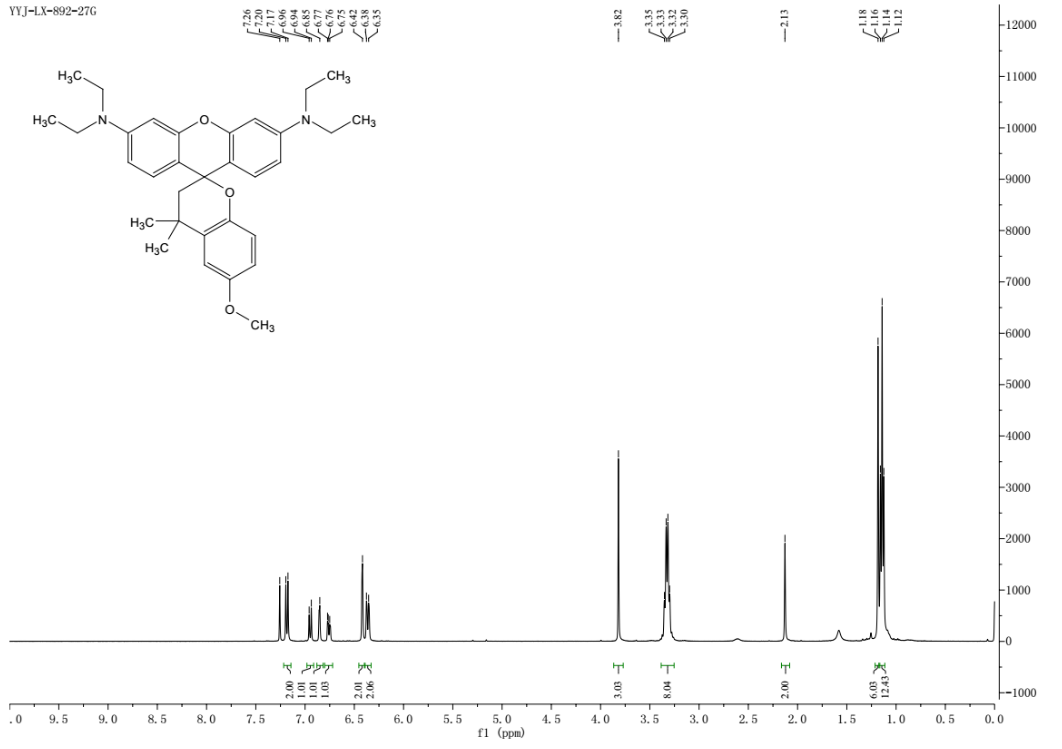

Supplementary Figure 299. The ^{1}H-NMR of compound **RD51** in $CDCl_3$

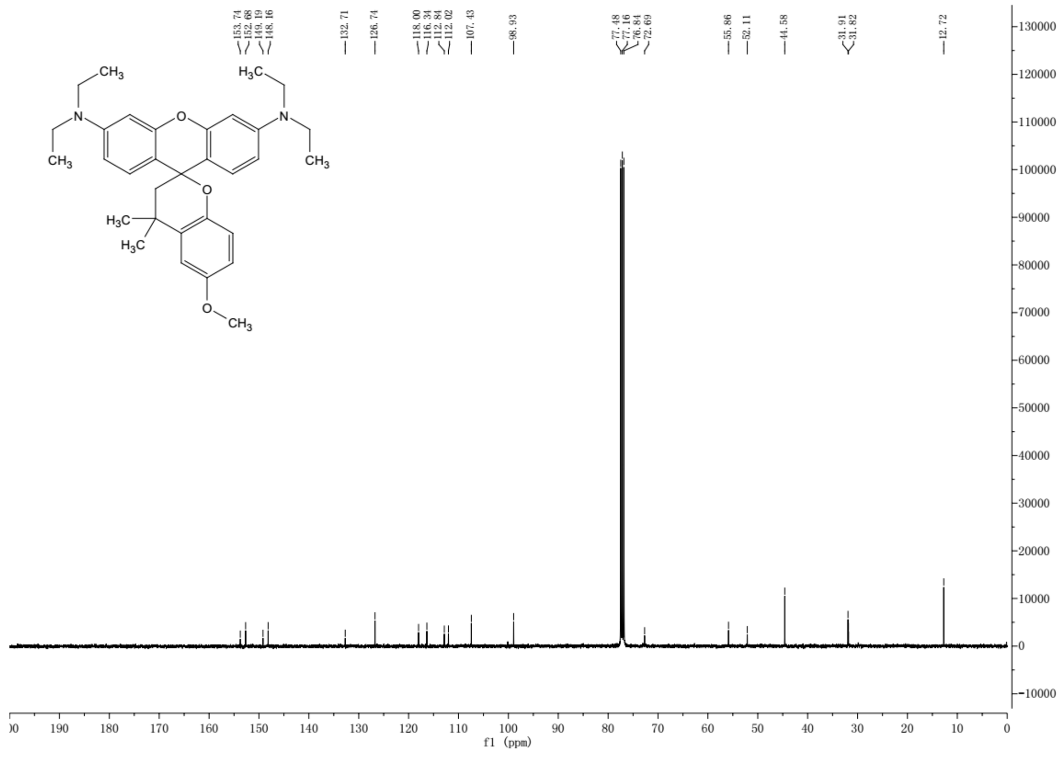

Supplementary Figure 300. The ^{13}C-NMR of compound **RD51** in $CDCl_3$

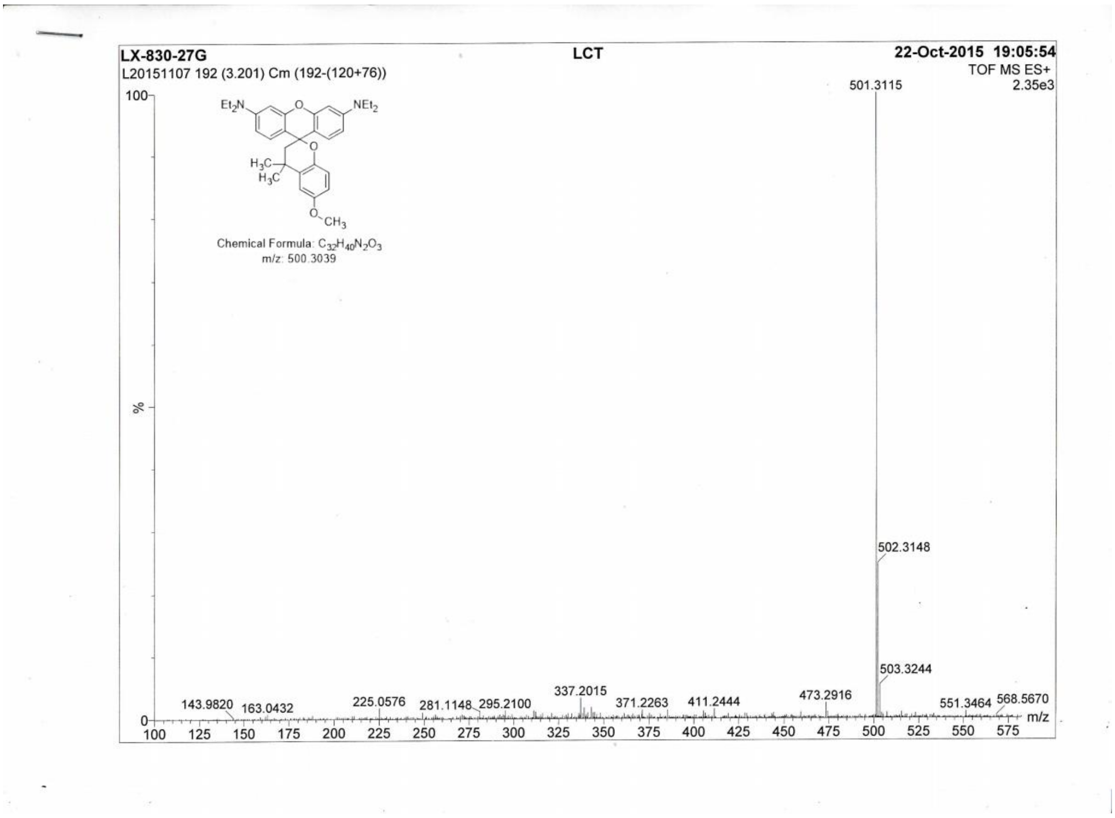

Supplementary Figure 301. The HR-MS of compound **RD51**

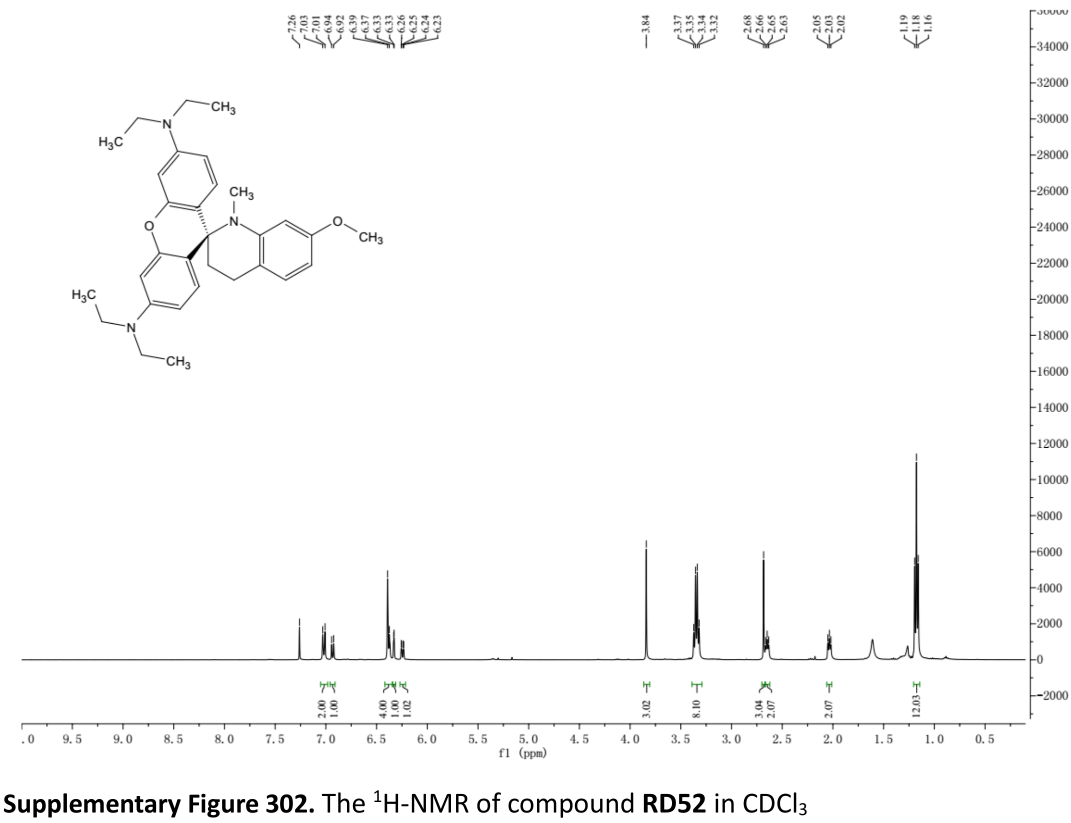

Supplementary Figure 302. The ^{1}H-NMR of compound **RD52** in $CDCl_3$

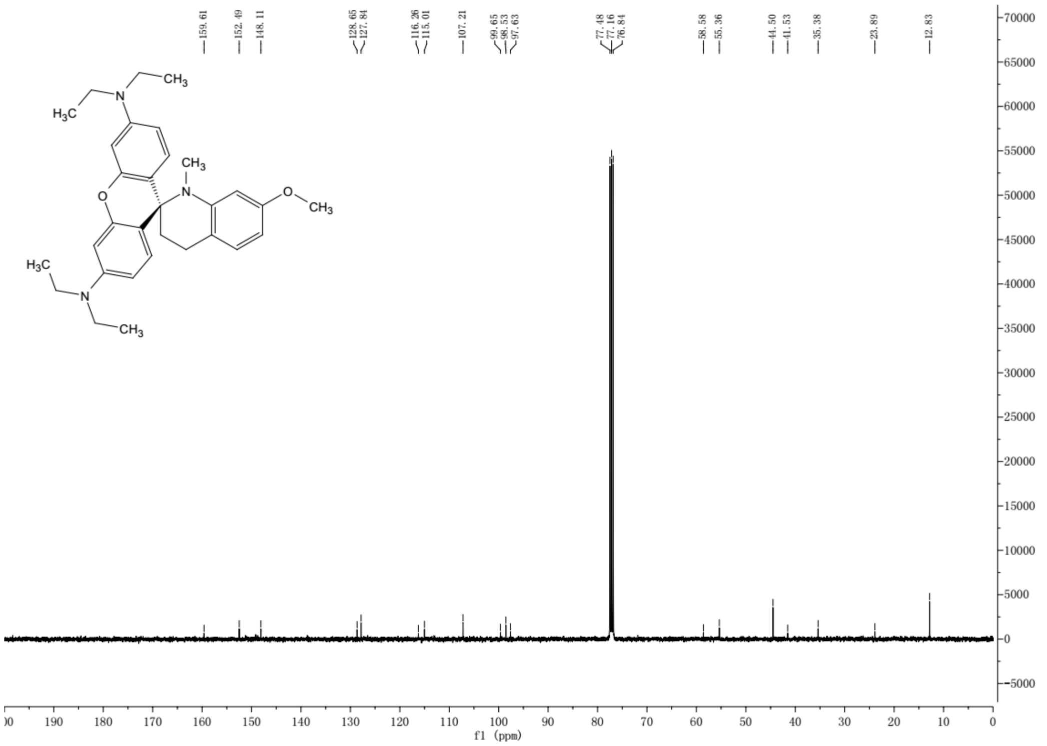

Supplementary Figure 303. The ^{13}C-NMR of compound **RD52** in $CDCl_3$

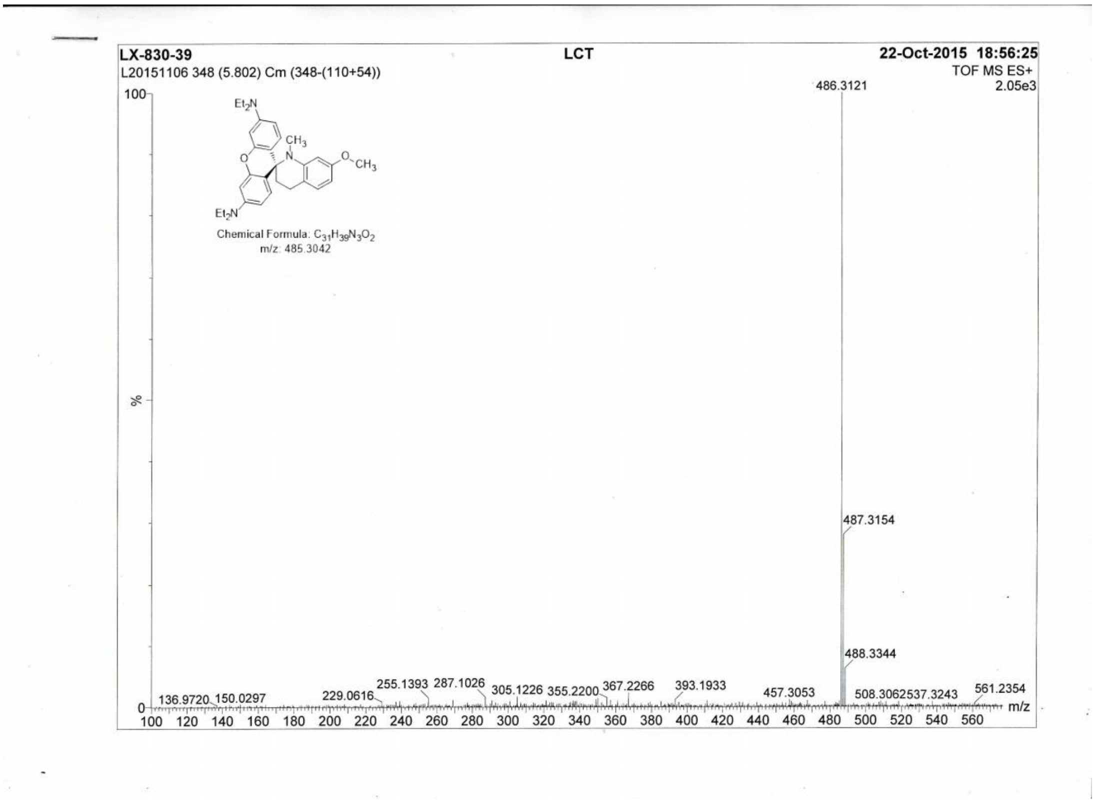

Supplementary Figure 304. The HR-MS of compound **RD52**

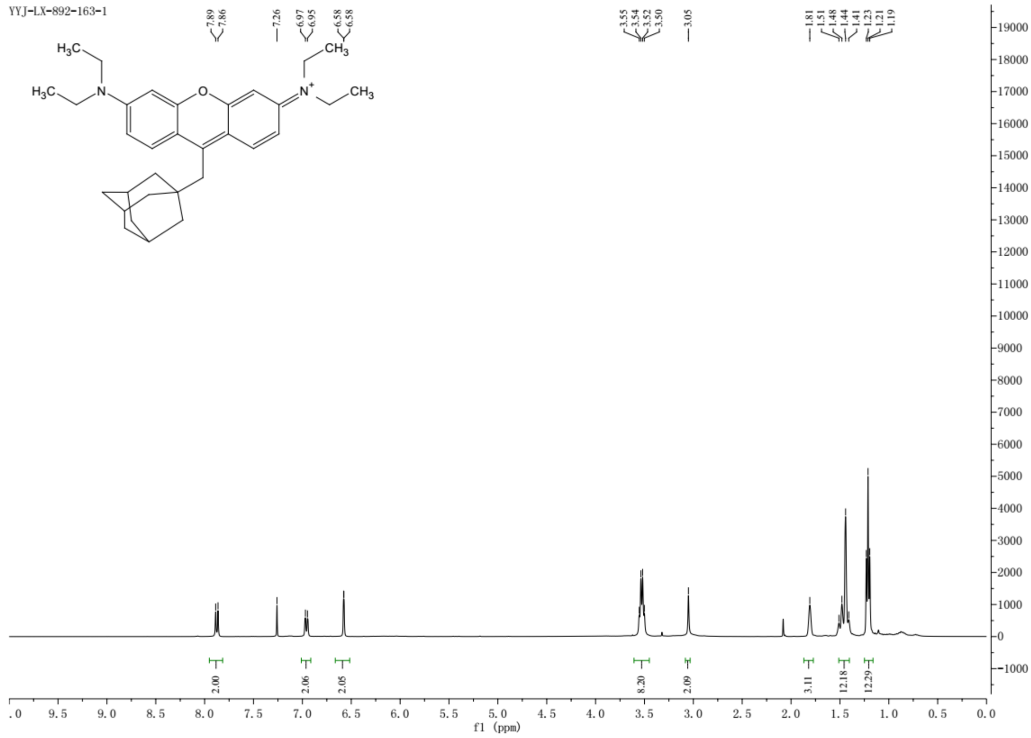

Supplementary Figure 305. The ^{1}H-NMR of compound **RD53** in $CDCl_3$

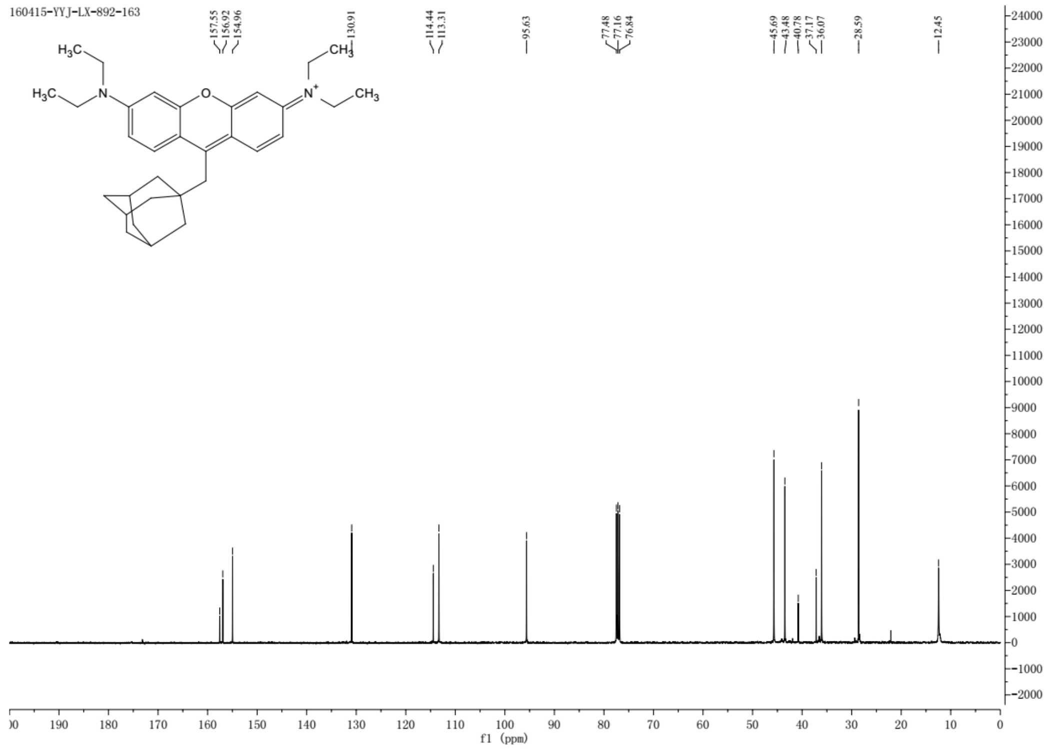

Supplementary Figure 306. The ^{13}C-NMR of compound **RD53** in $CDCl_3$

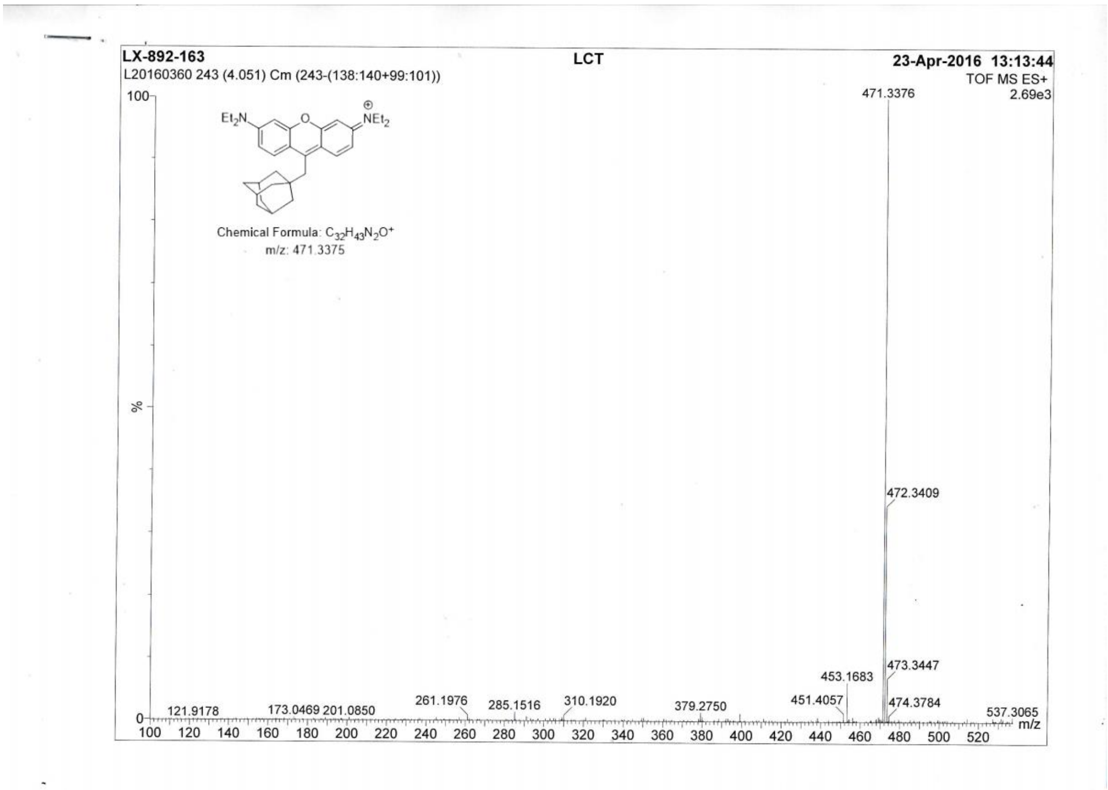

Supplementary Figure 307. The HR-MS of compound **RD53**

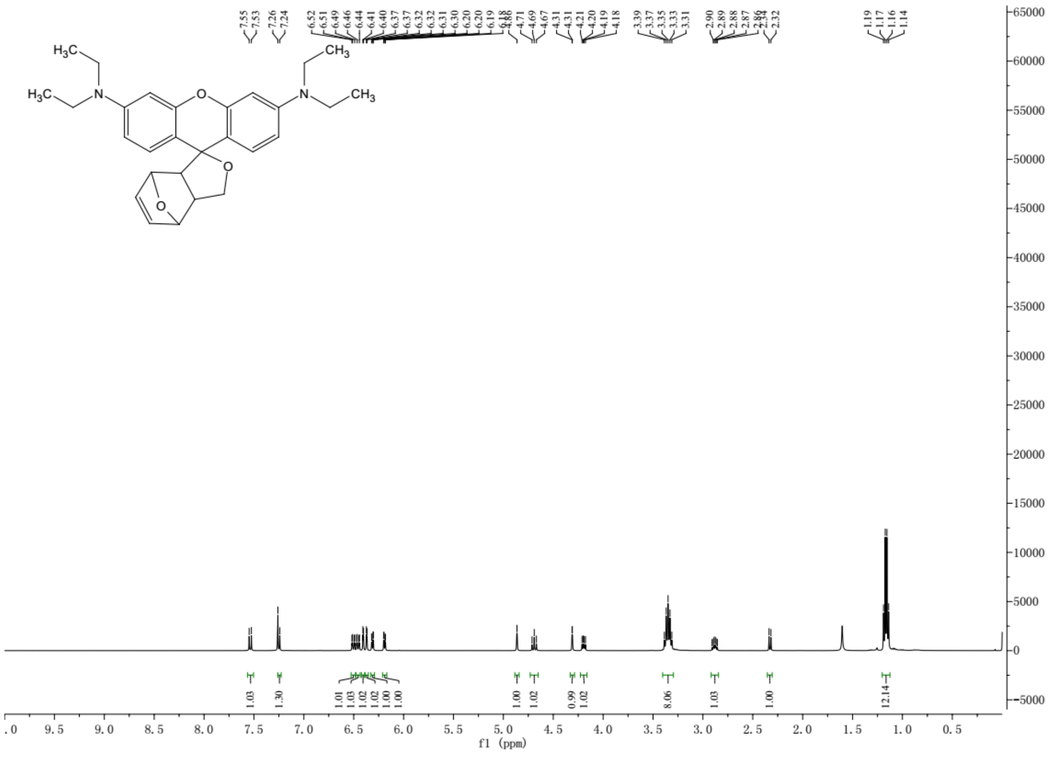

Supplementary Figure 308. The ^{1}H-NMR of compound **RD54** in $CDCl_3$

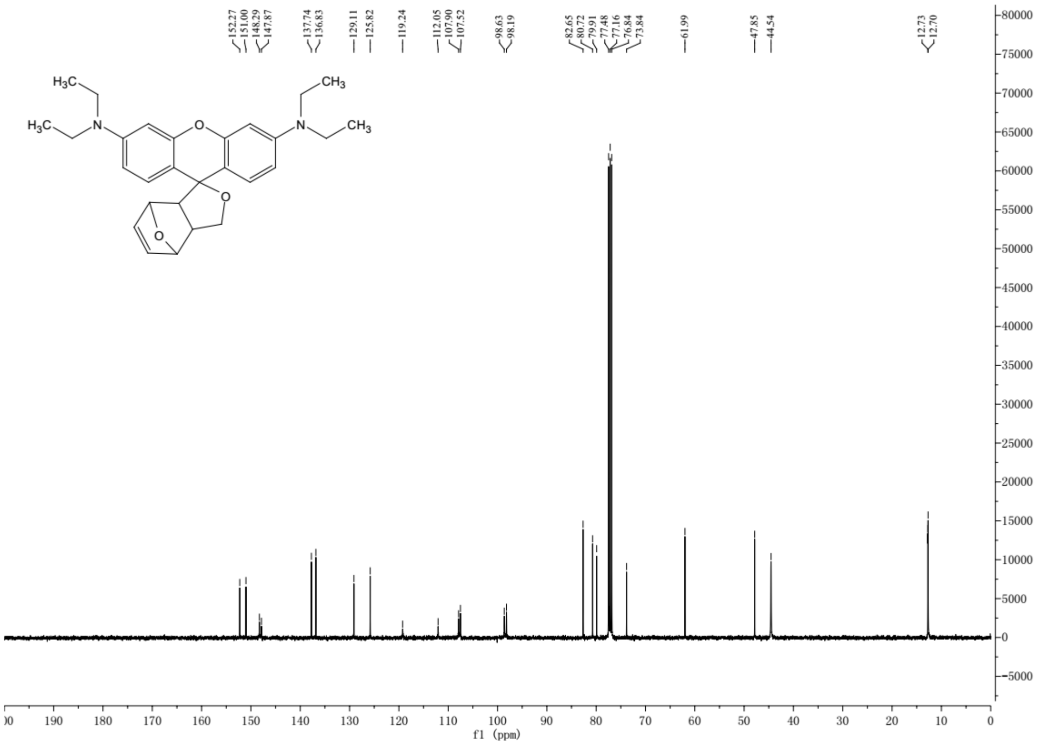

Supplementary Figure 309. The ^{13}C-NMR of compound **RD54** in $CDCl_3$

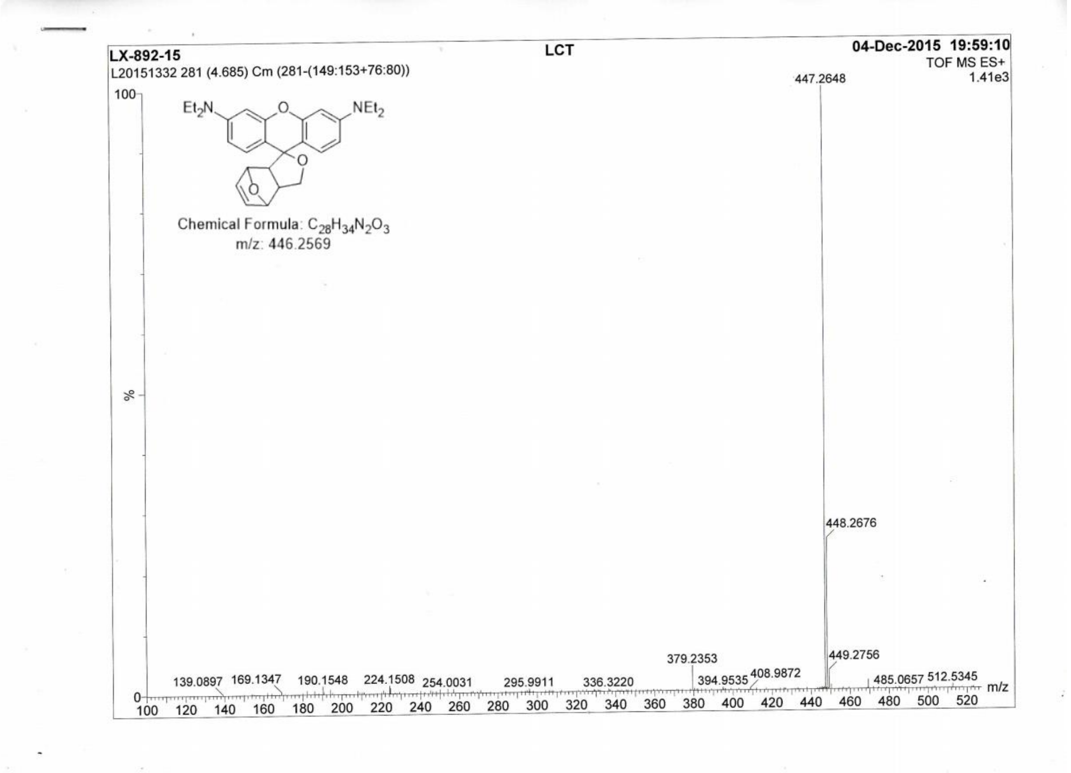

Supplementary Figure 310. The HR-MS of compound **RD54**

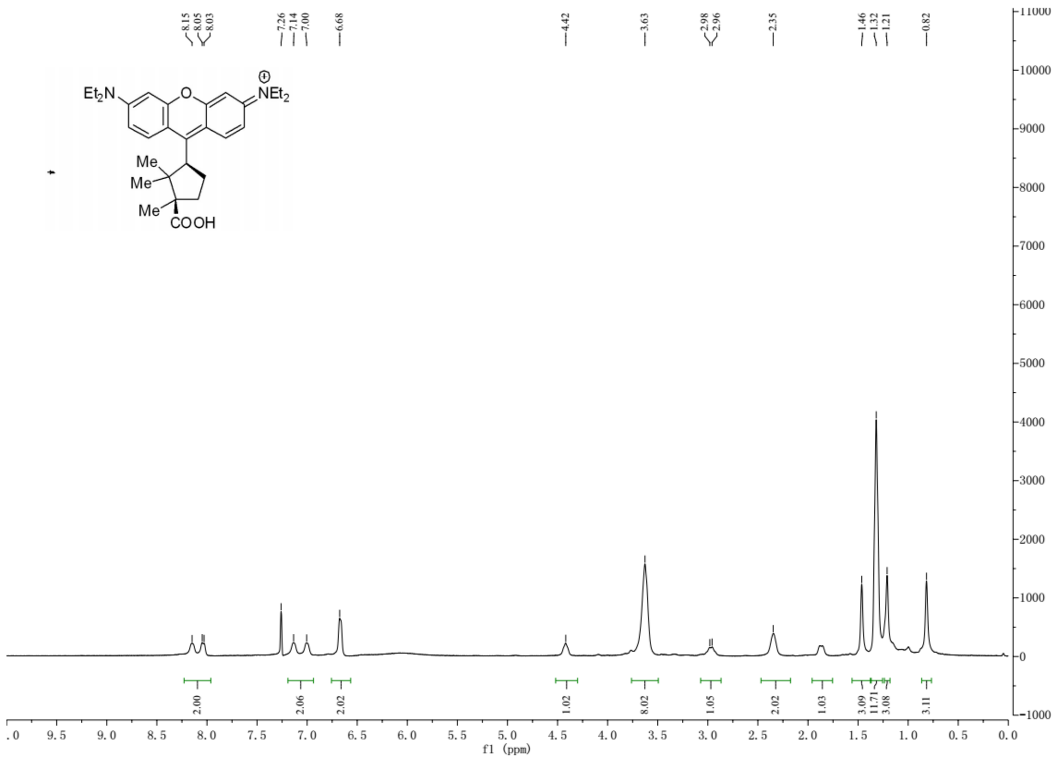

Supplementary Figure 311. The ^{1}H-NMR of compound **RD55** in $CDCl_3$

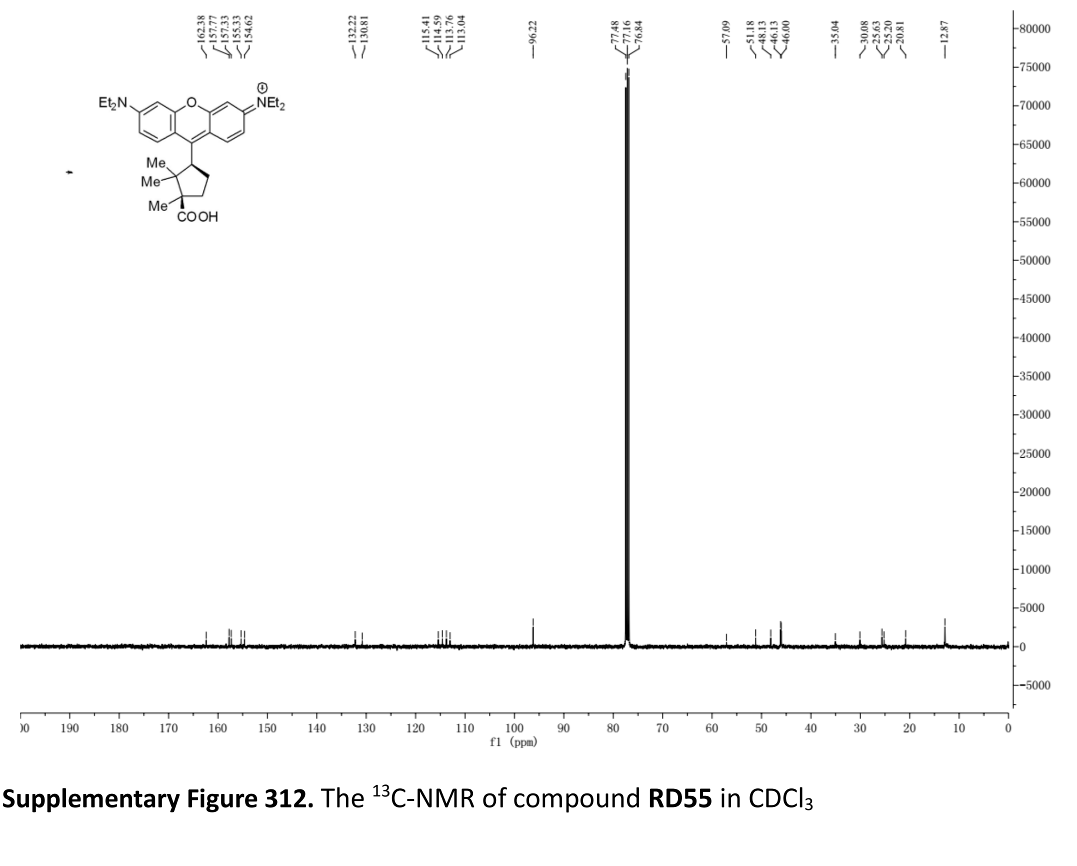

Supplementary Figure 312. The ^{13}C-NMR of compound **RD55** in $CDCl_3$

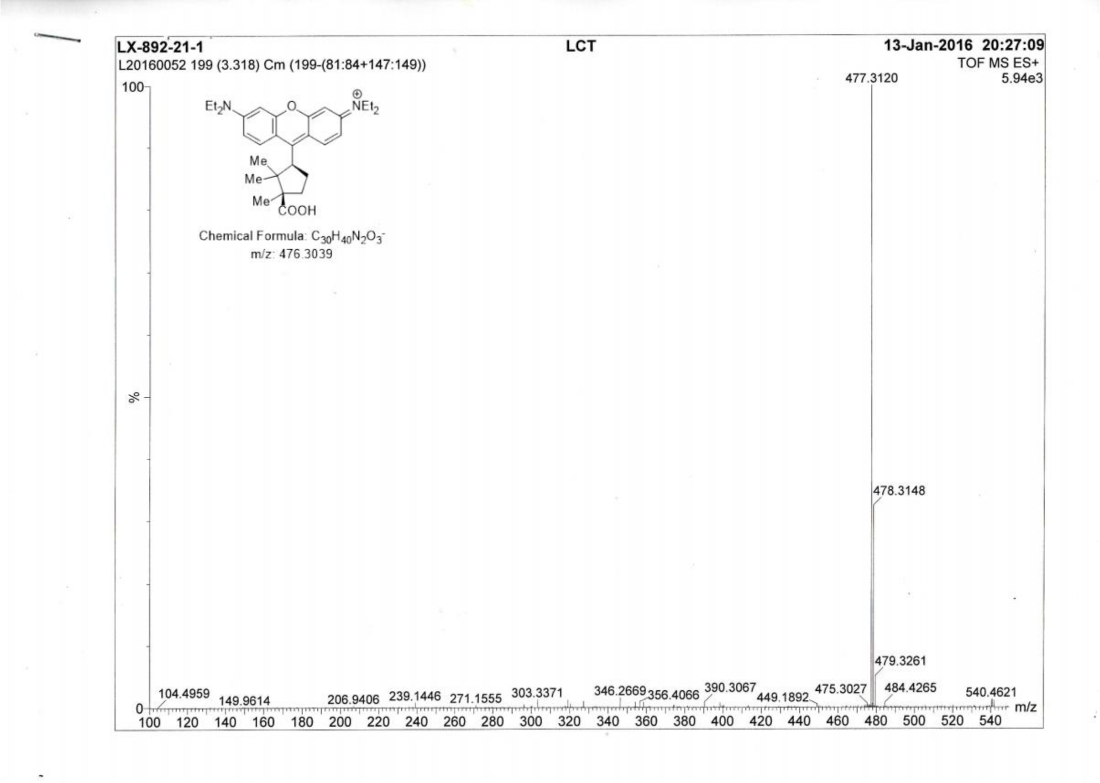

Supplementary Figure 313. The HR-MS of compound **RD55**

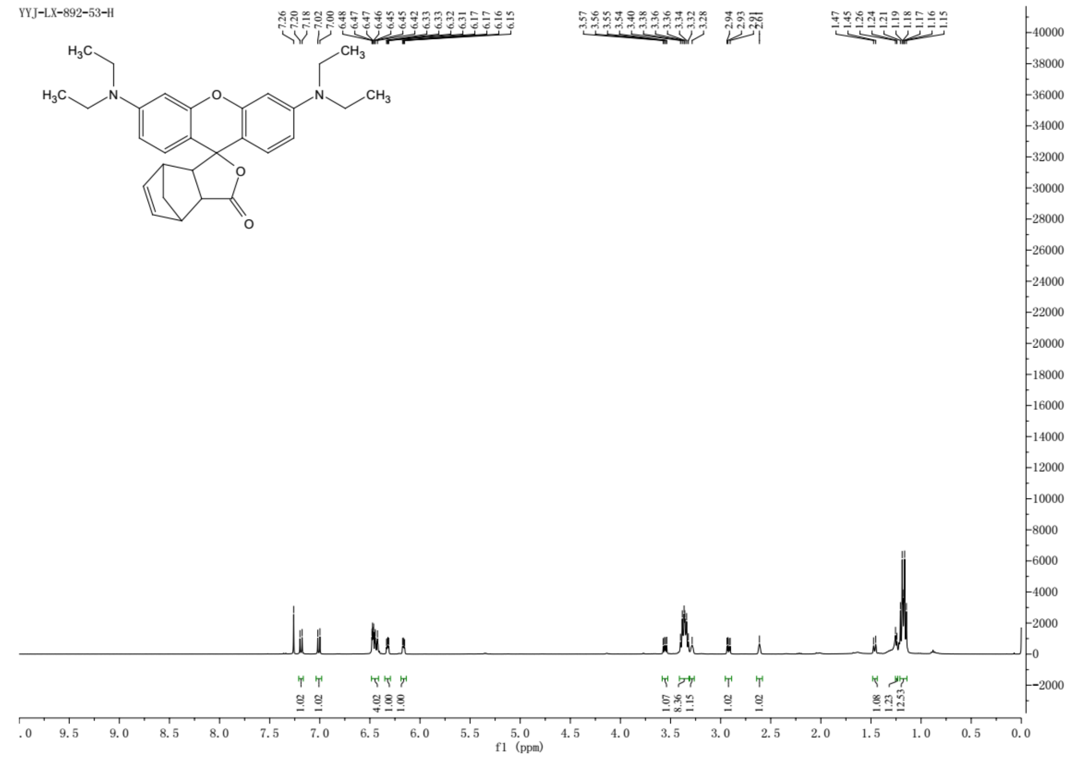

Supplementary Figure 314. The ^{1}H-NMR of compound **RD56** in $CDCl_3$

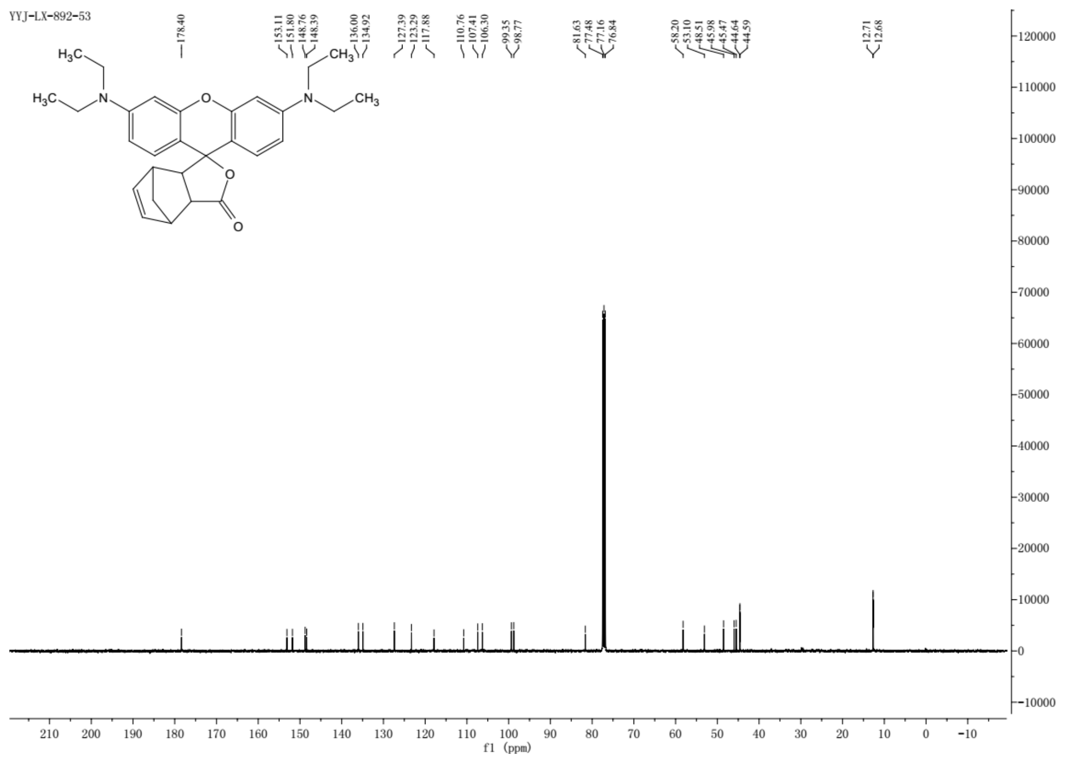

Supplementary Figure 315. The ^{13}C-NMR of compound **RD56** in $CDCl_3$

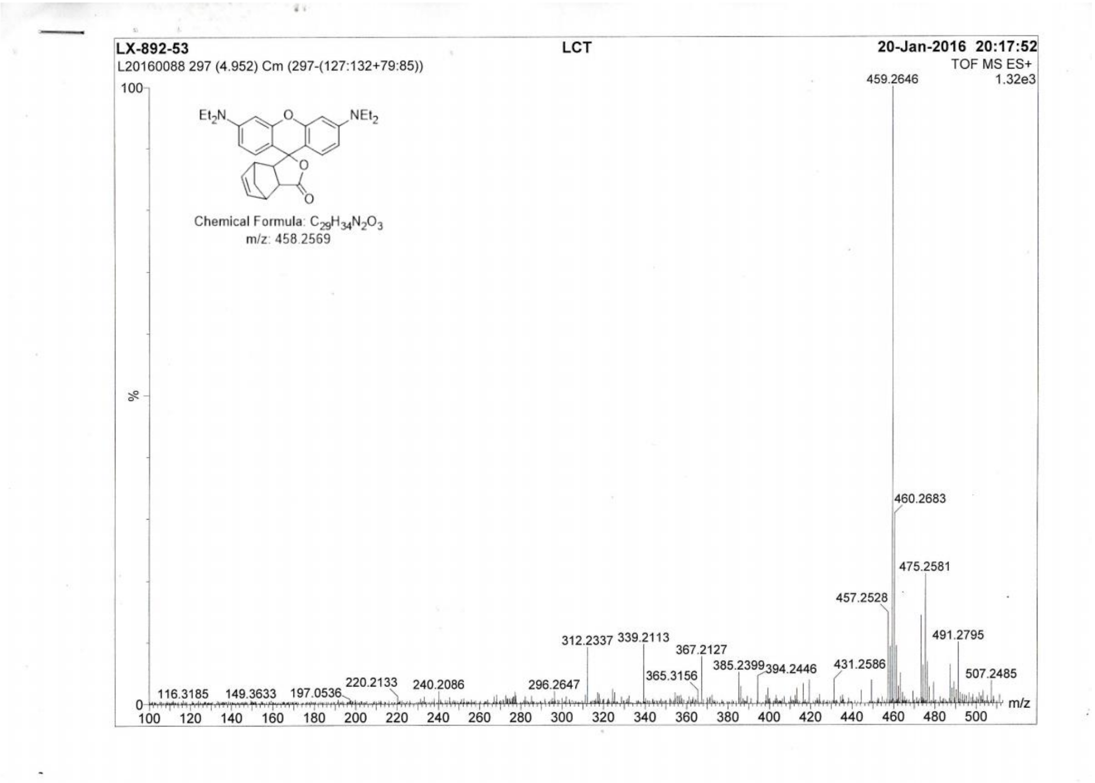

Supplementary Figure 316. The HR-MS of compound **RD56**

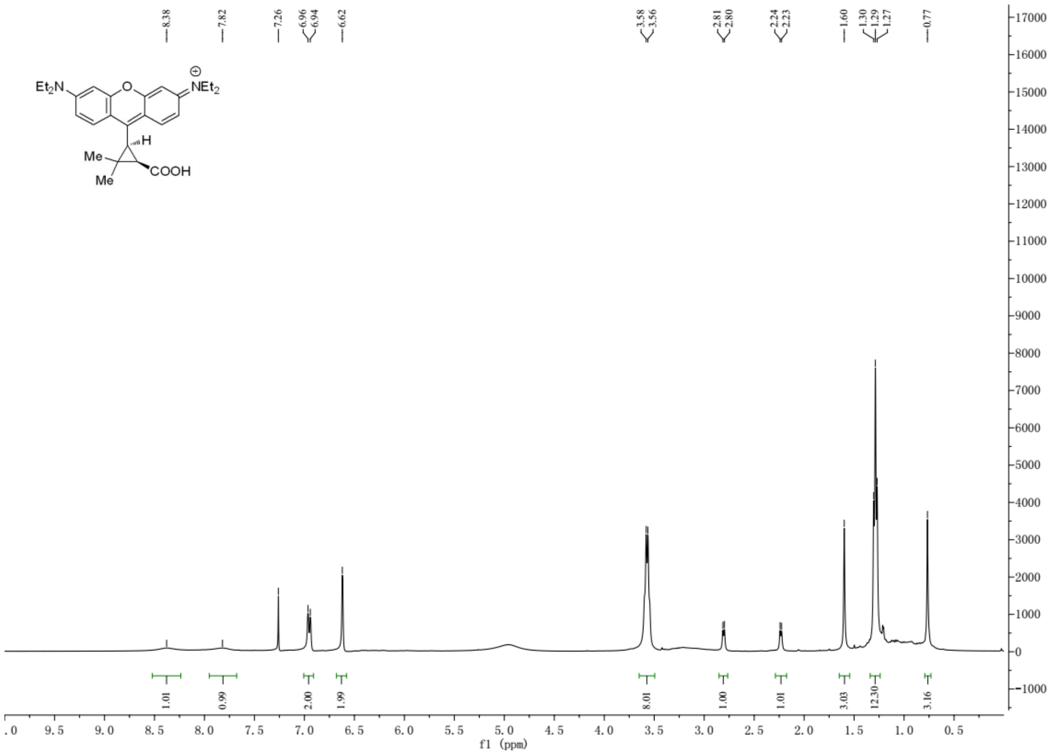

Supplementary Figure 317. The ^{1}H-NMR of compound **RD57** in $CDCl_3$

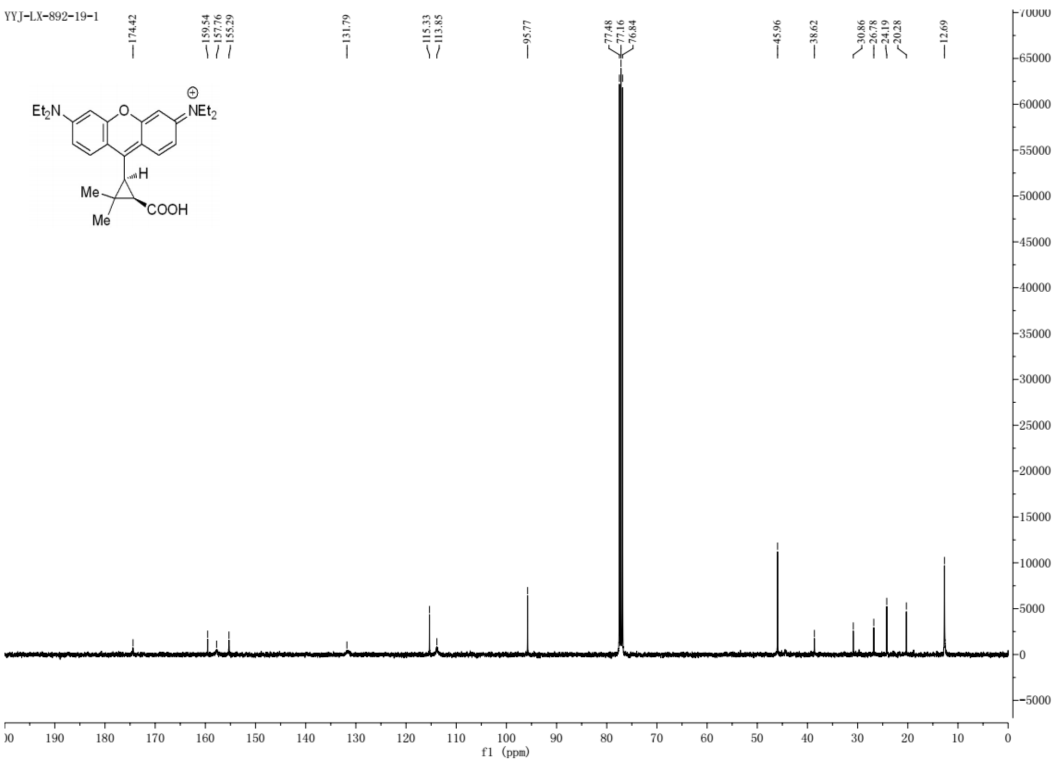

Supplementary Figure 318. The ^{13}C-NMR of compound **RD57** in $CDCl_3$

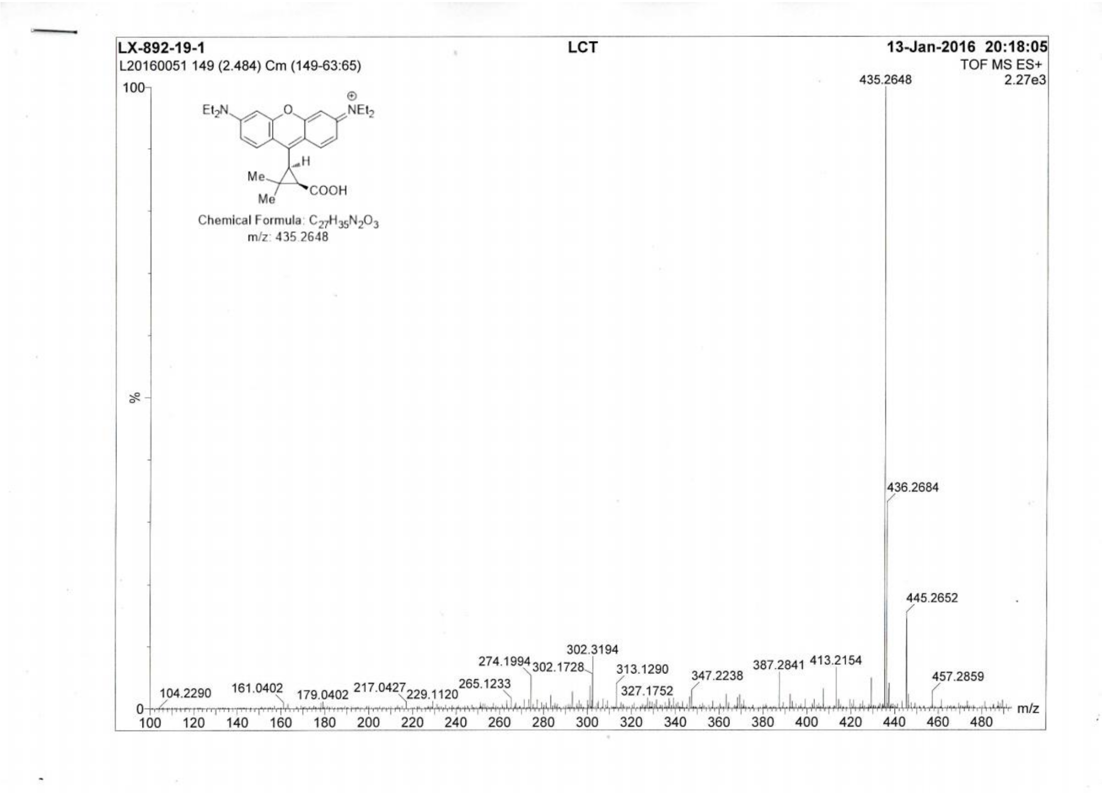

Supplementary Figure 319. The HR-MS of compound **RD57**

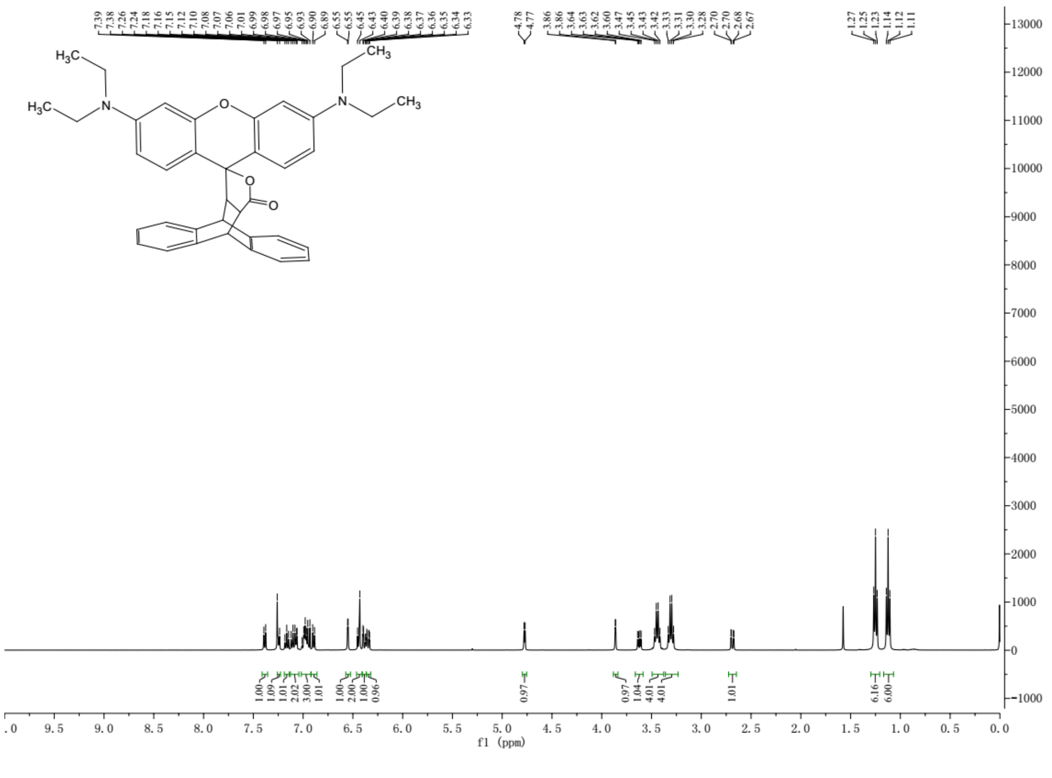

Supplementary Figure 320. The ^{1}H-NMR of compound **RD58** in $CDCl_3$

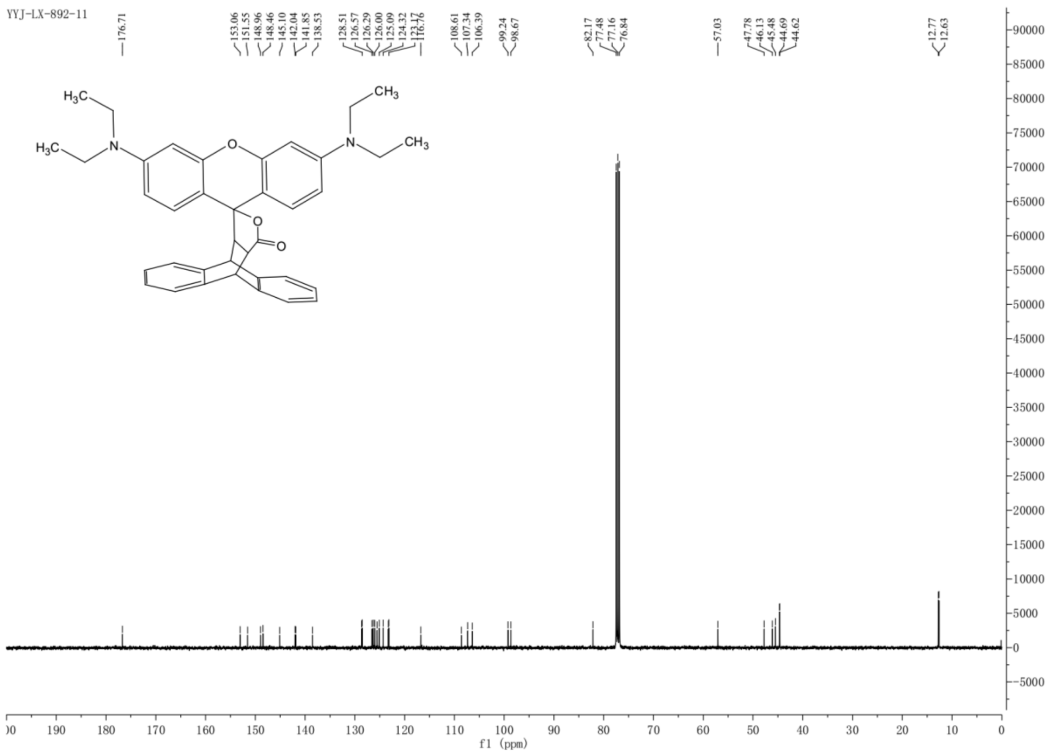

Supplementary Figure 321. The ^{13}C-NMR of compound **RD58** in $CDCl_3$

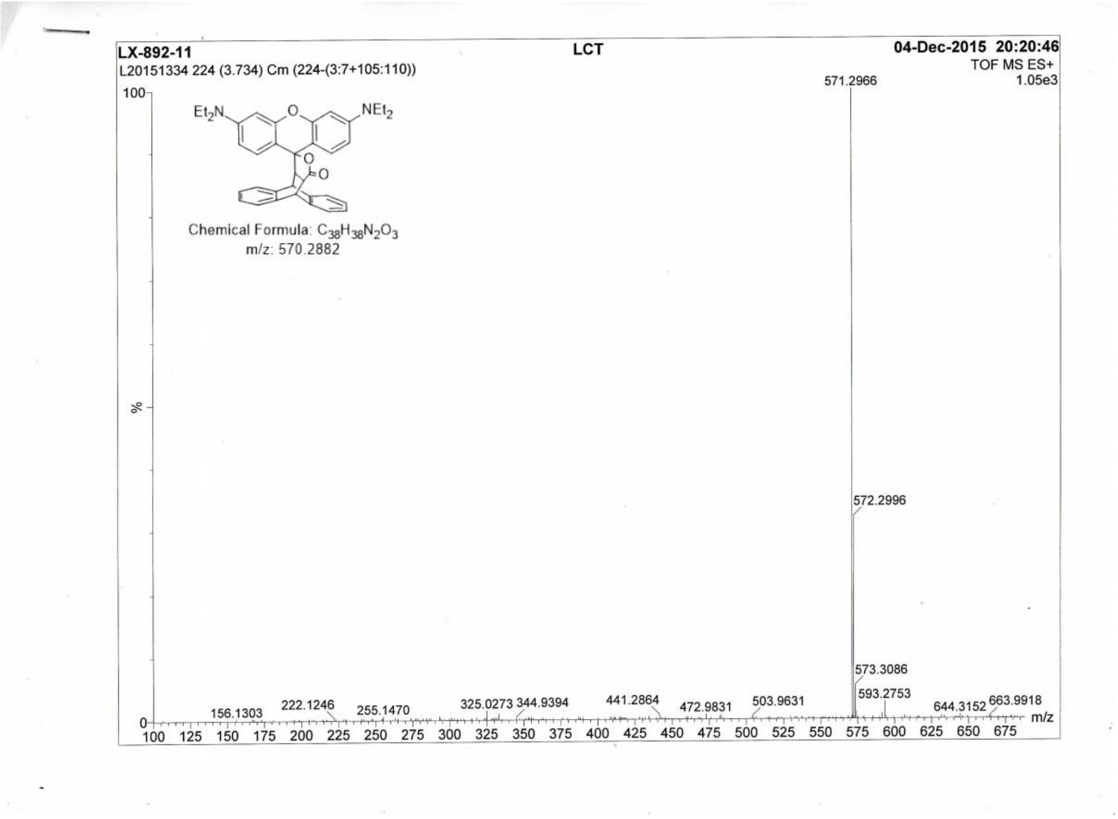

Supplementary Figure 322. The HR-MS of compound **RD58**

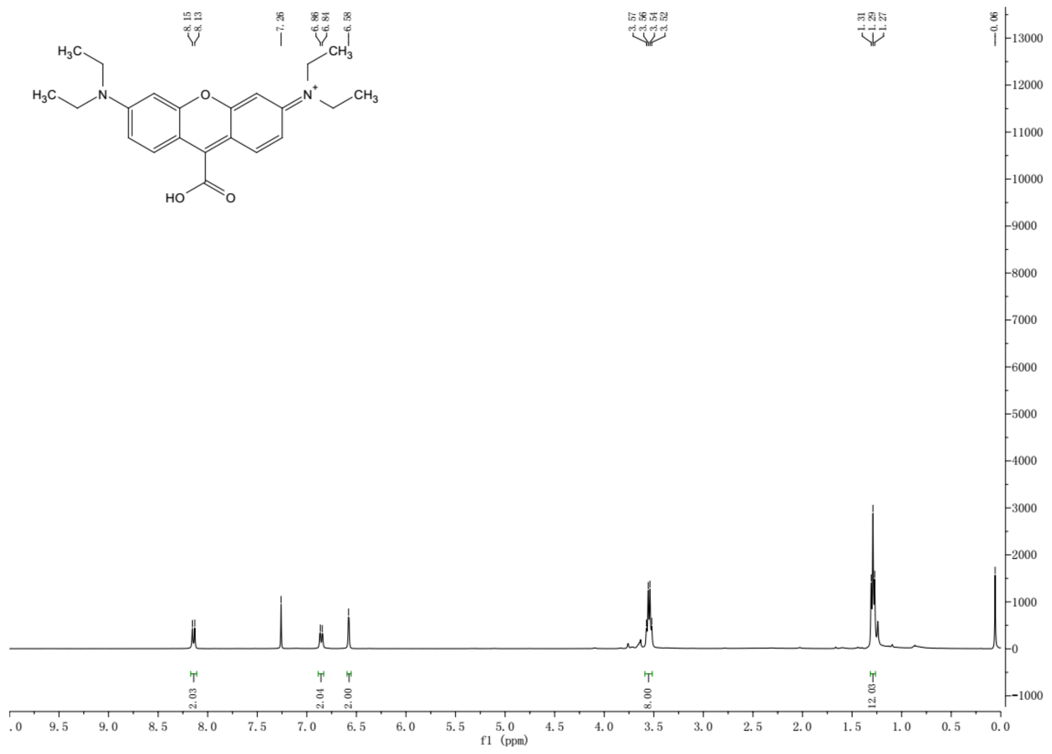

Supplementary Figure 323. The ^{1}H-NMR of compound **RD59** in $CDCl_3$

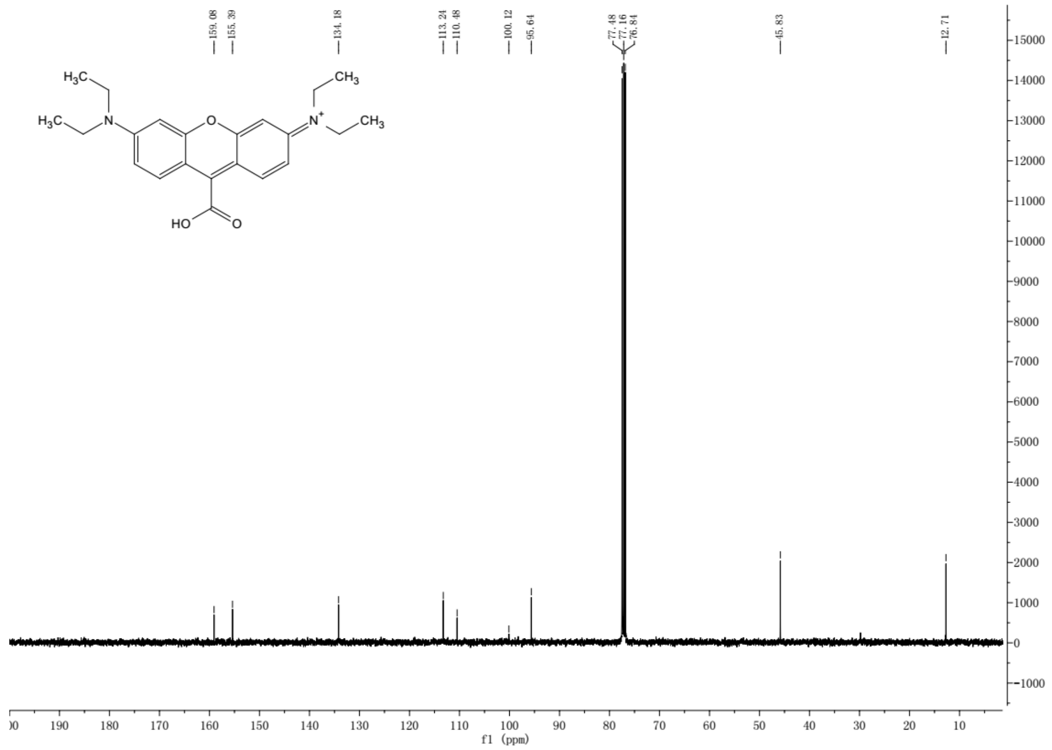

Supplementary Figure 324. The ^{13}C-NMR of compound **RD59** in $CDCl_3$

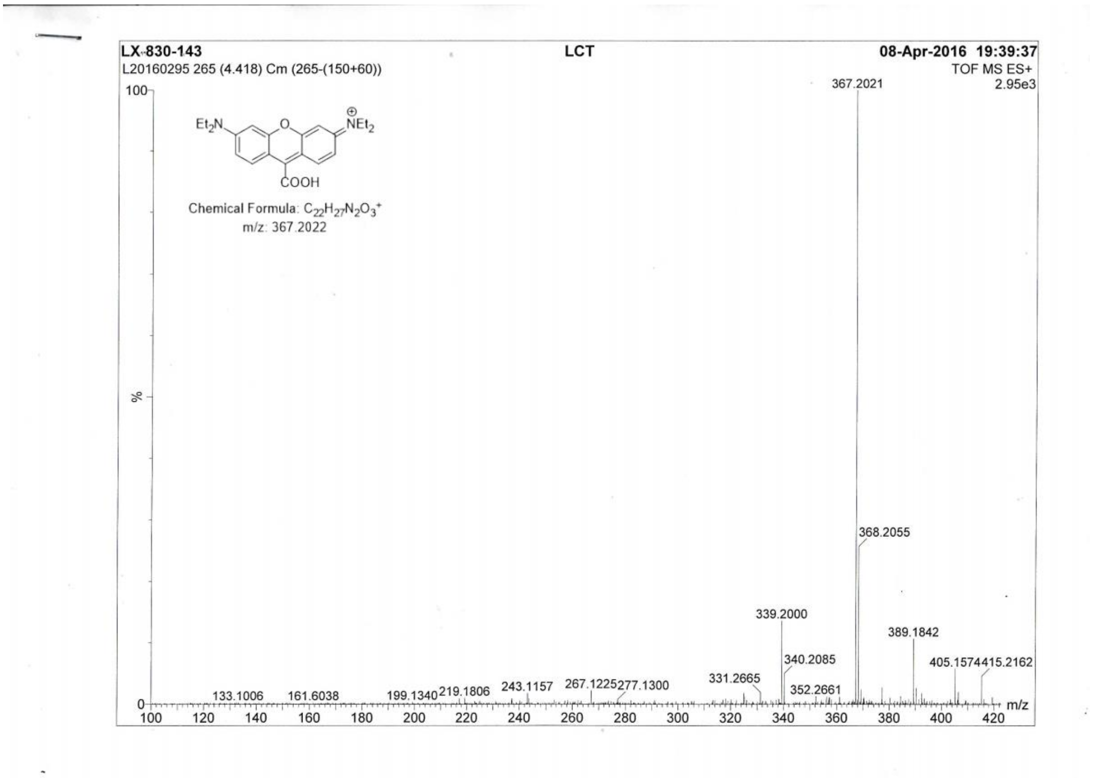

Supplementary Figure 325. The HR-MS of compound **RD59**

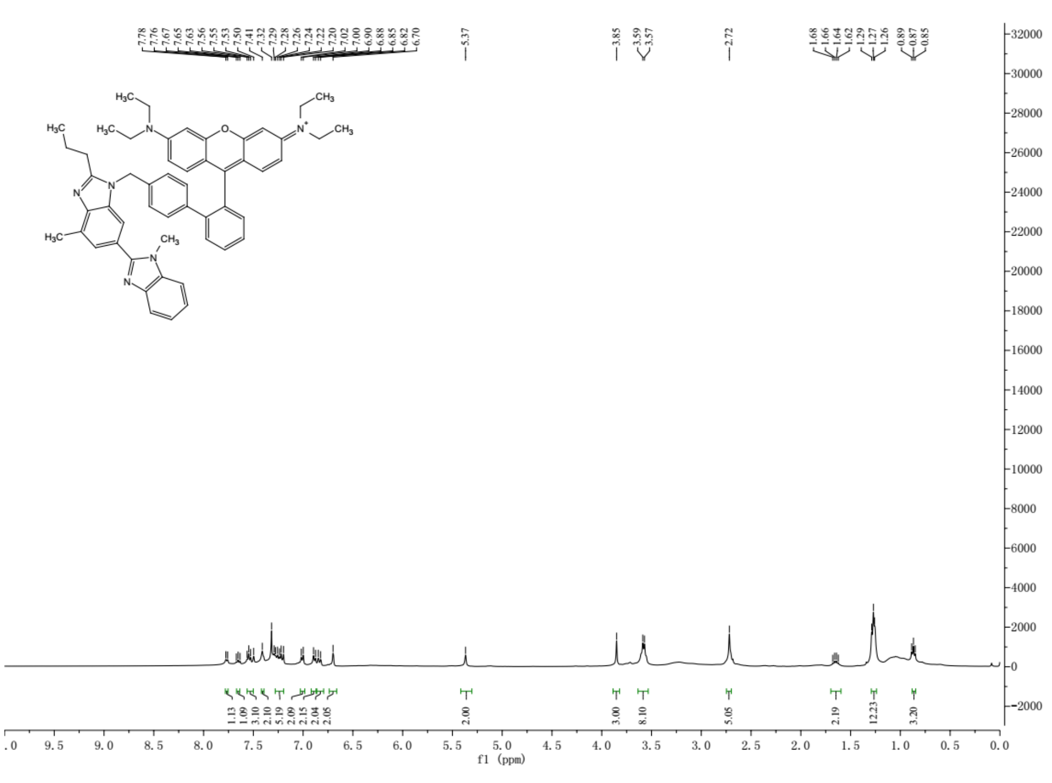

Supplementary Figure 326. The ^{1}H-NMR of compound **RD60** in $CDCl_3$

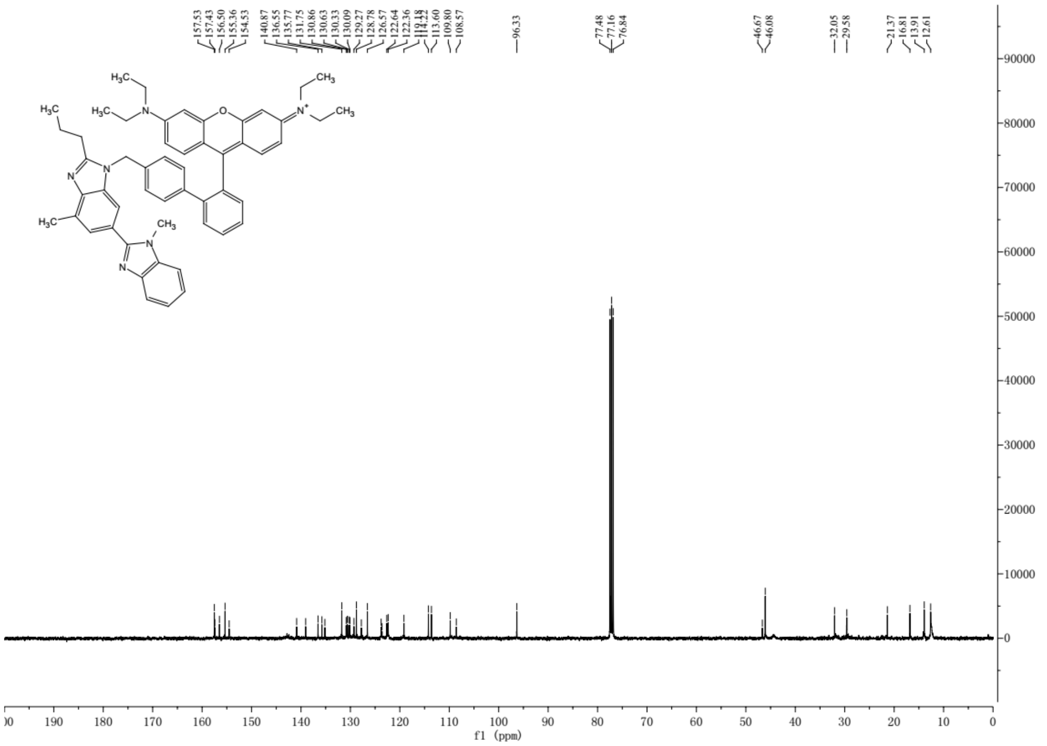

Supplementary Figure 327. The ^{13}C-NMR of compound **RD60** in $CDCl_3$

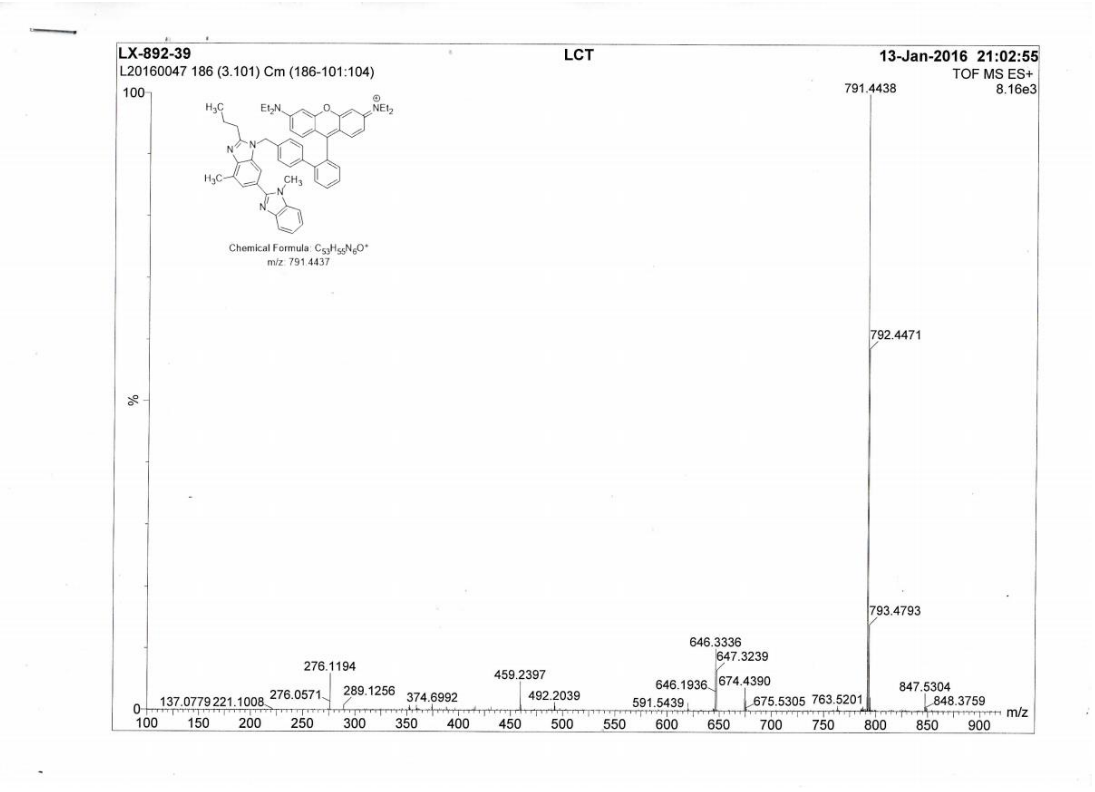

Supplementary Figure 328. The HR-MS of compound **RD60**

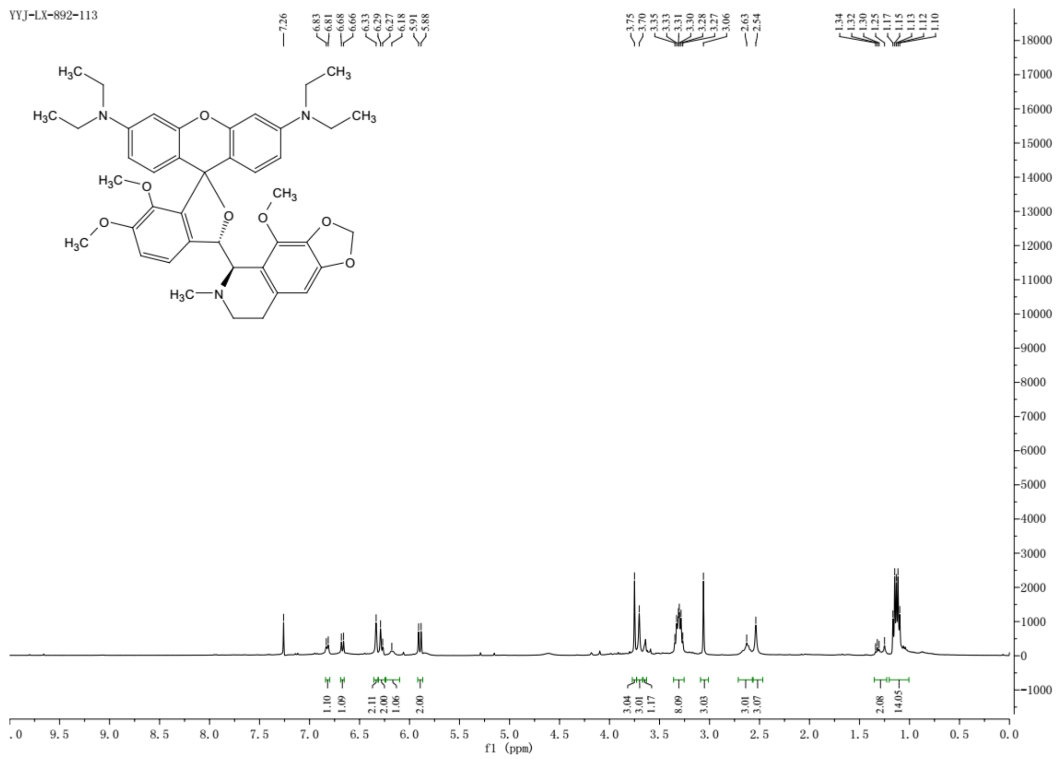

Supplementary Figure 329. The ^{1}H-NMR of compound **RD61** in $CDCl_3$

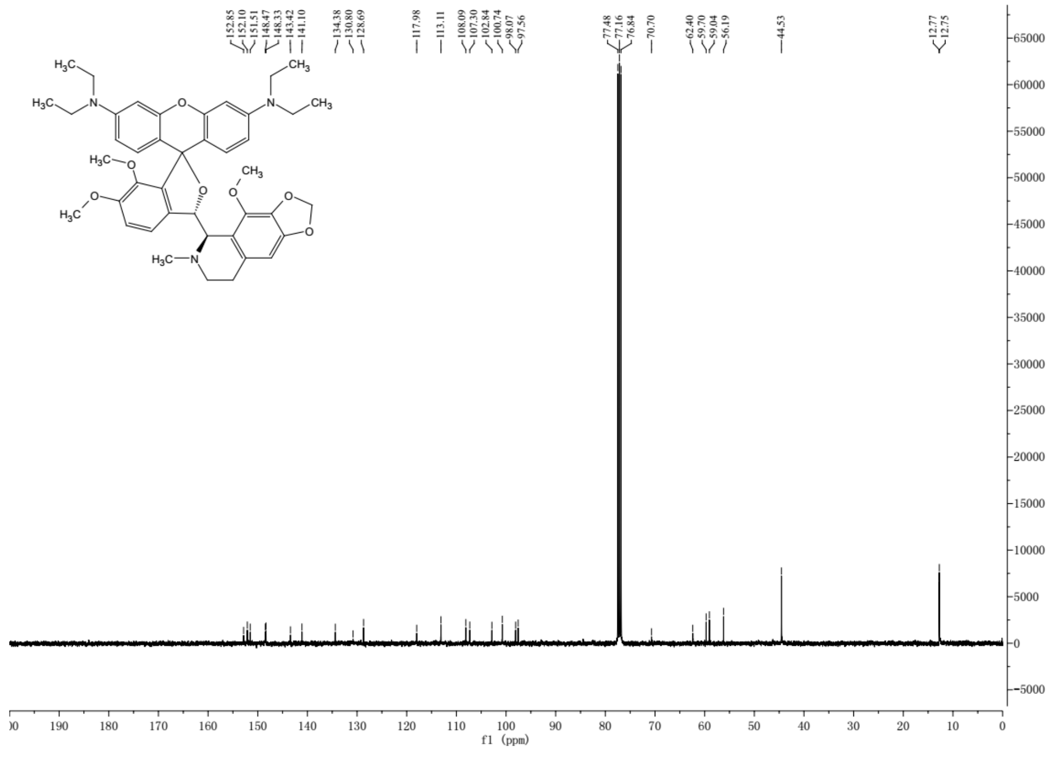

Supplementary Figure 330. The ^{13}C-NMR of compound **RD61** in $CDCl_3$

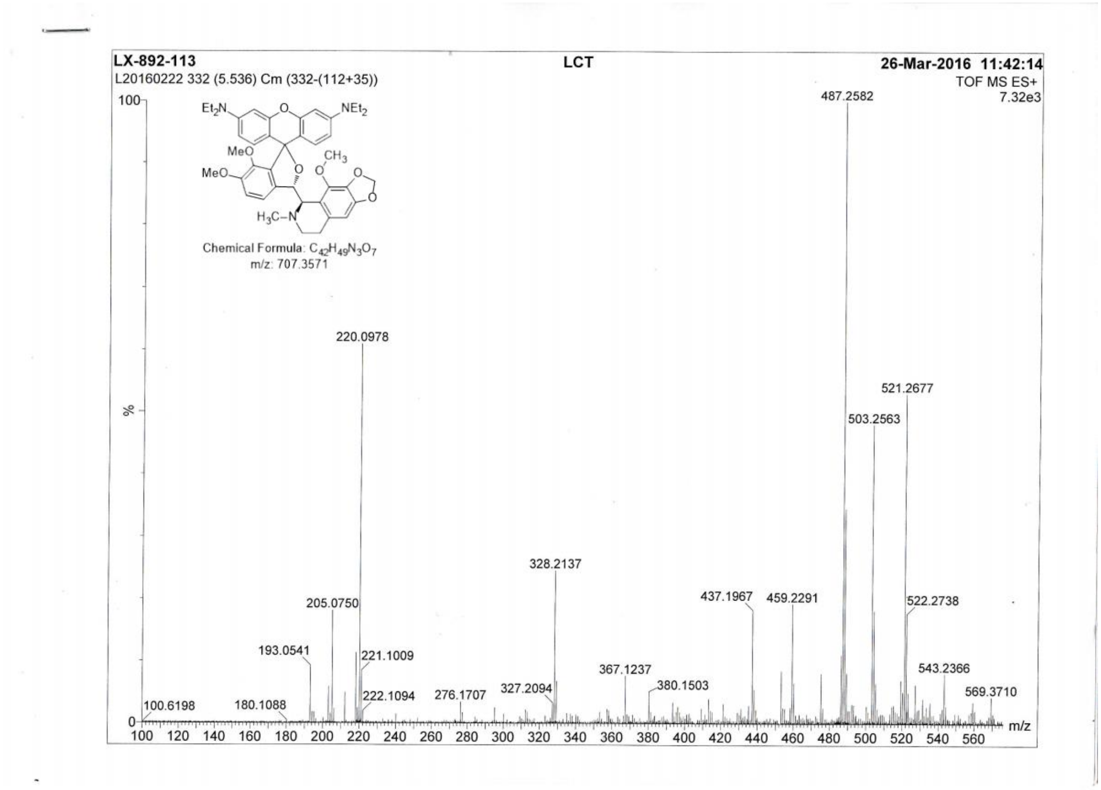

Supplementary Figure 331. The HR-MS of compound **RD61**

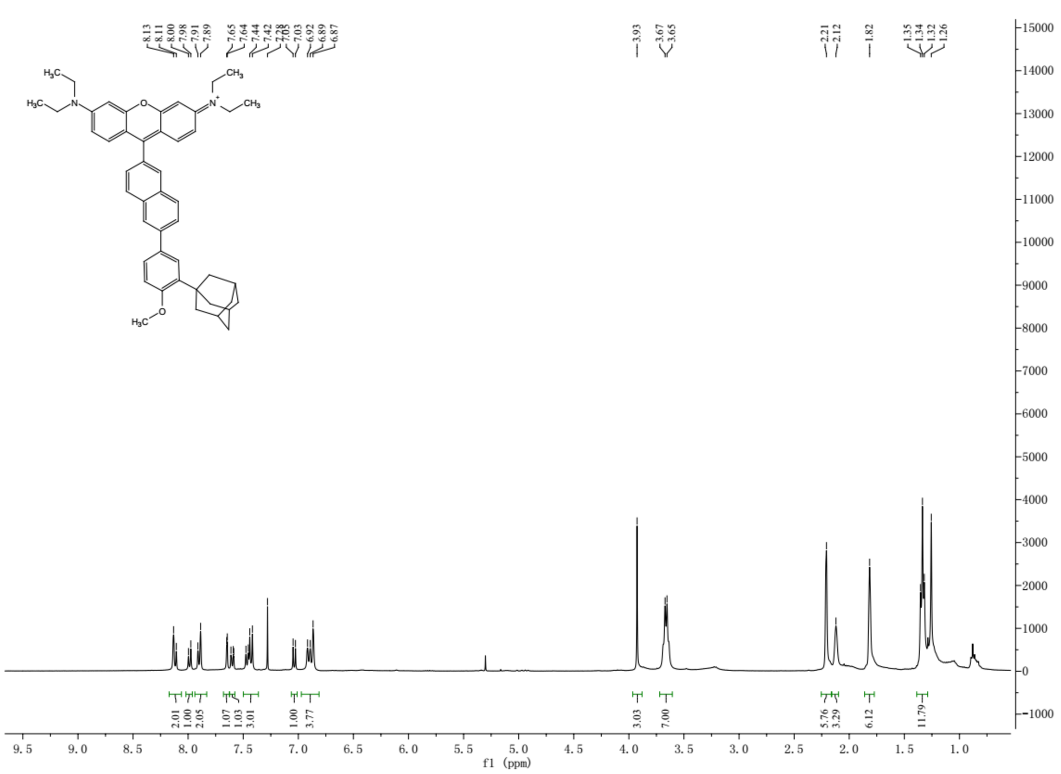

Supplementary Figure 332. The ^{1}H-NMR of compound **RD62** in $CDCl_3$

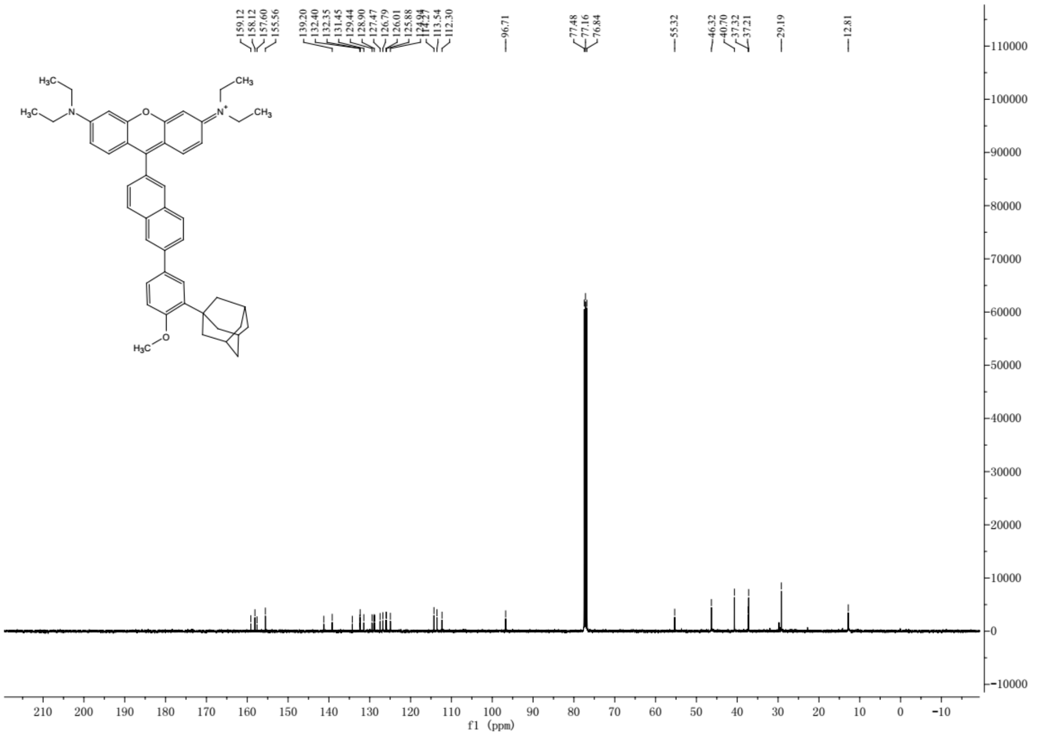

Supplementary Figure 333. The ^{13}C-NMR of compound **RD62** in $CDCl_3$

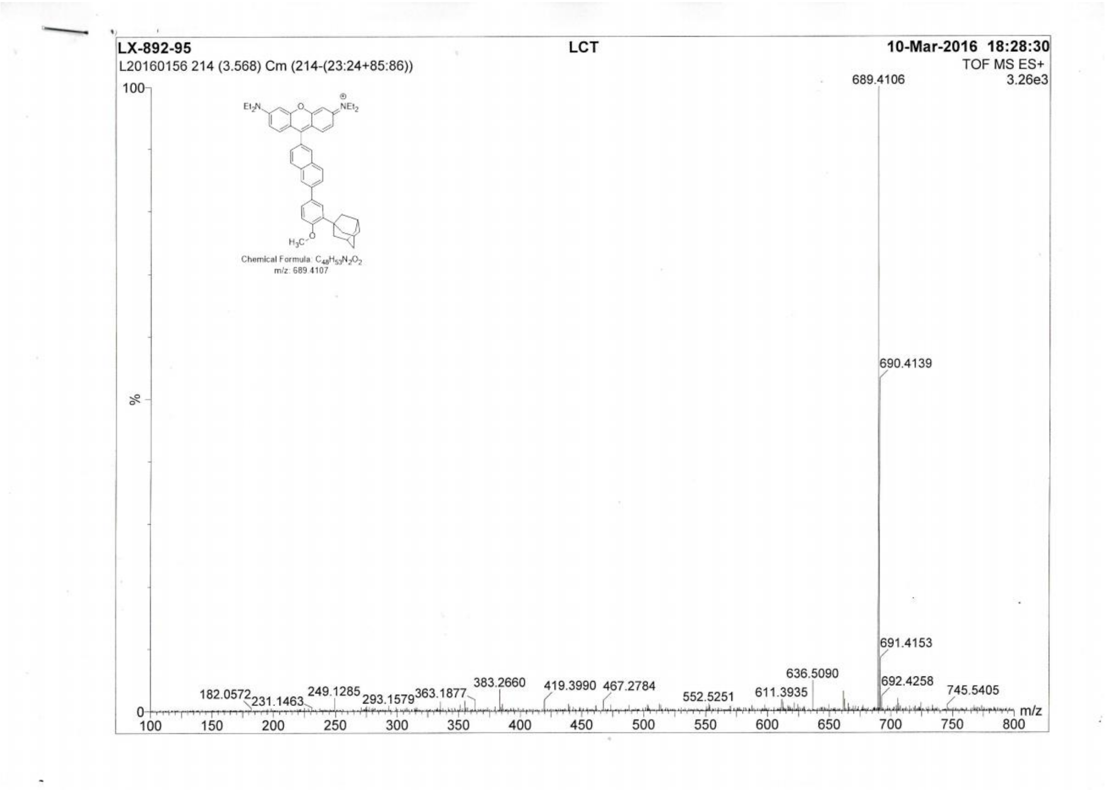

Supplementary Figure 334. The HR-MS of compound **RD62**

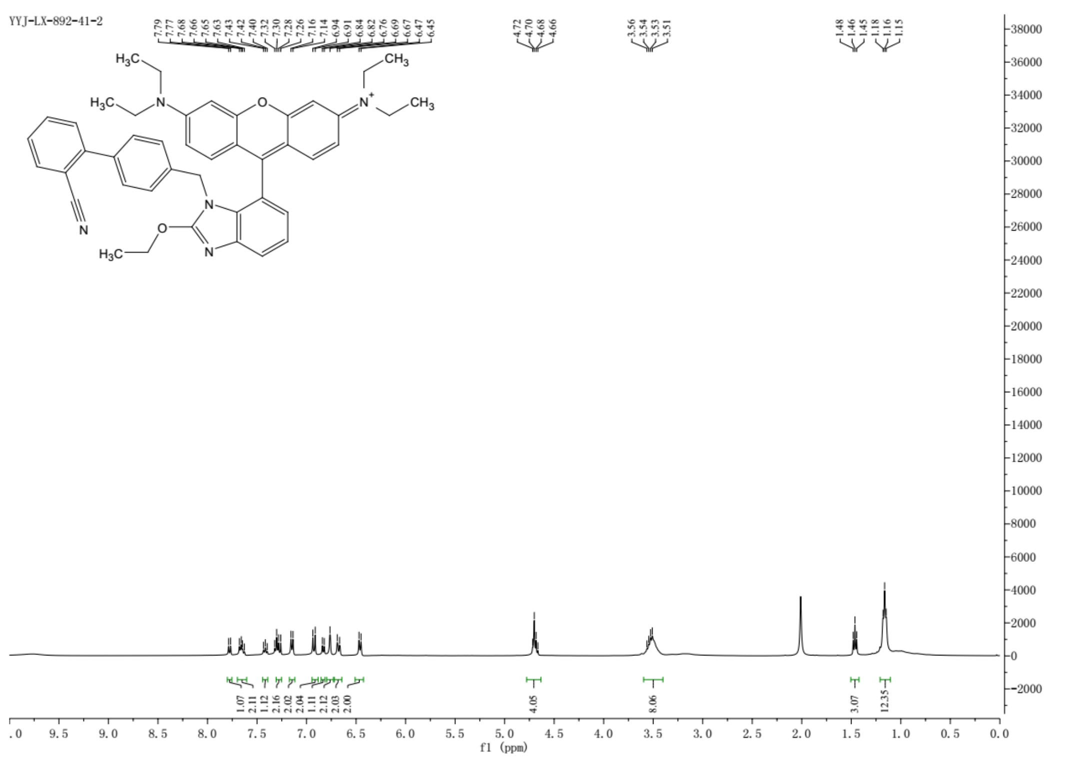

Supplementary Figure 335. The ^{1}H-NMR of compound **RD63** in $CDCl_3$

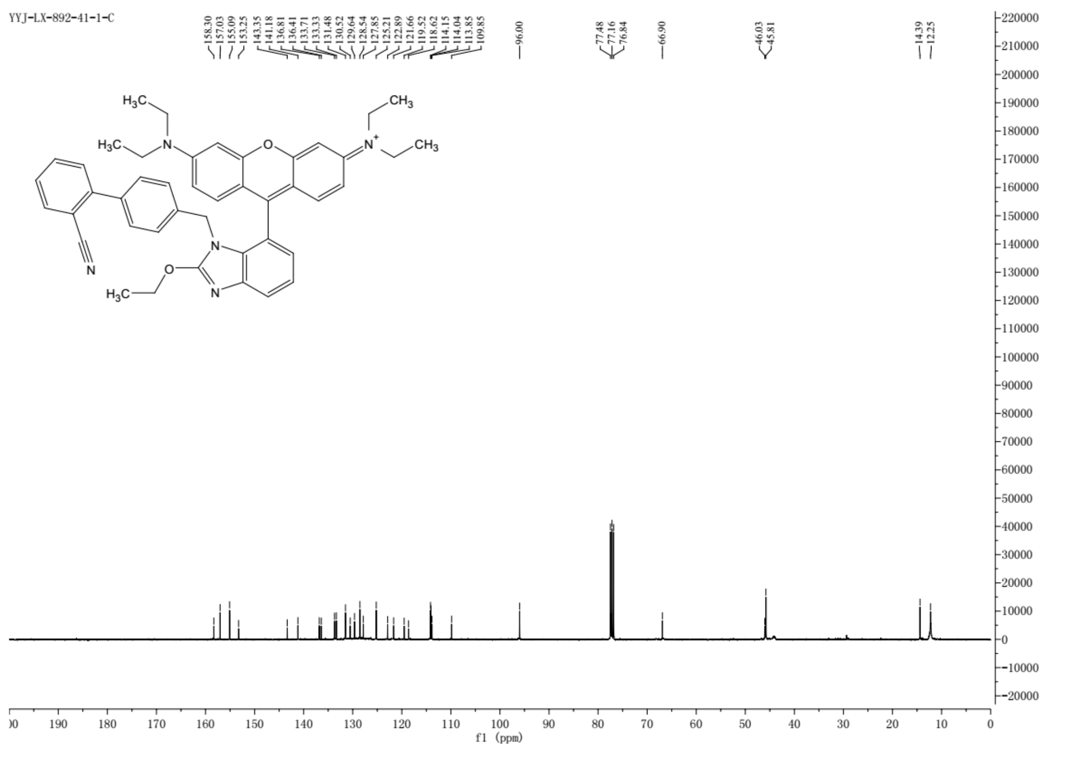

Supplementary Figure 336. The ^{13}C-NMR of compound **RD63** in $CDCl_3$

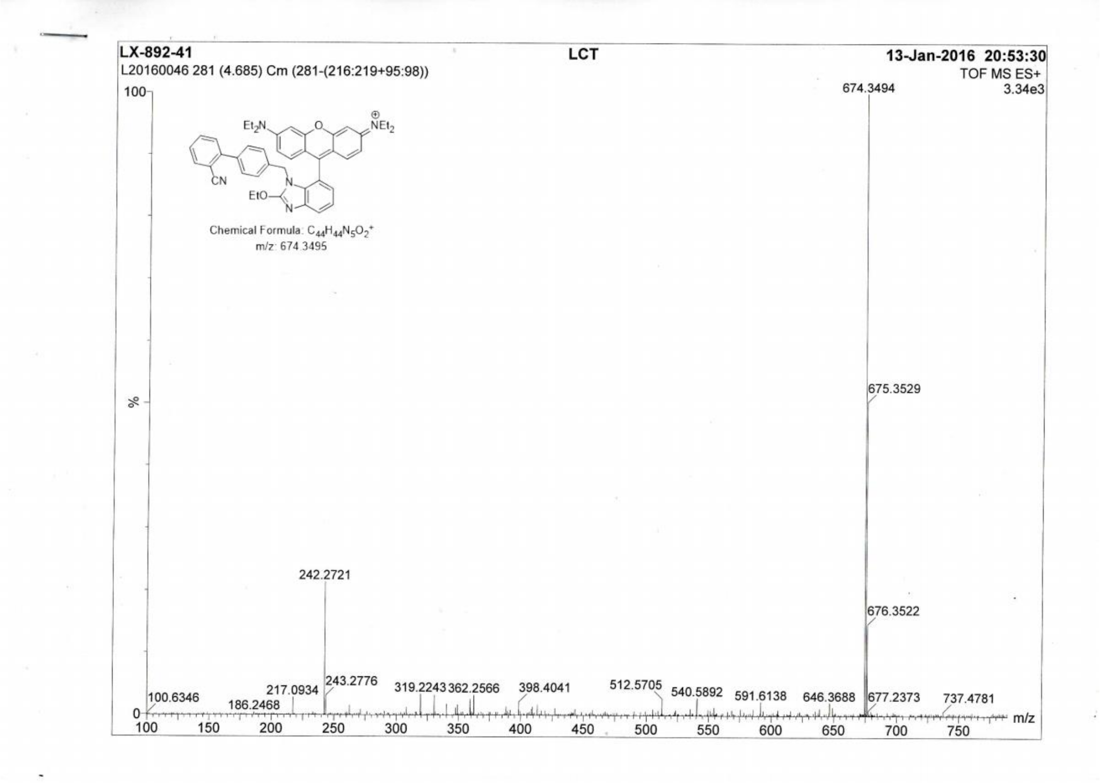

Supplementary Figure 337. The HR-MS of compound **RD63**

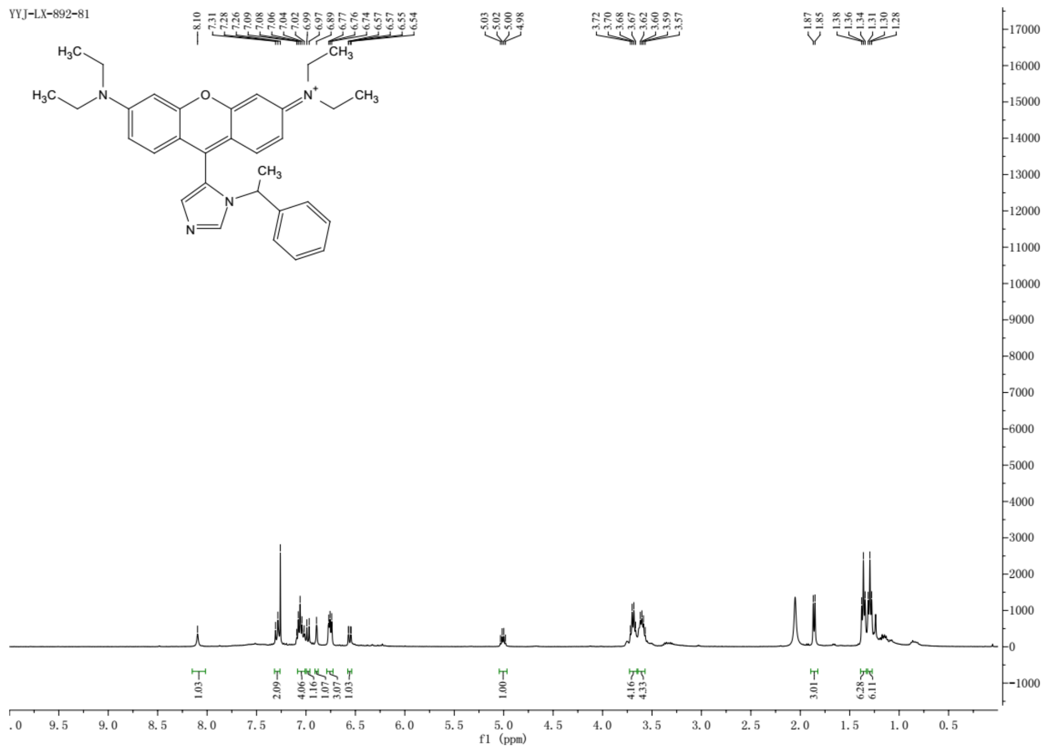

Supplementary Figure 338. The ^{1}H-NMR of compound **RD64** in $CDCl_3$

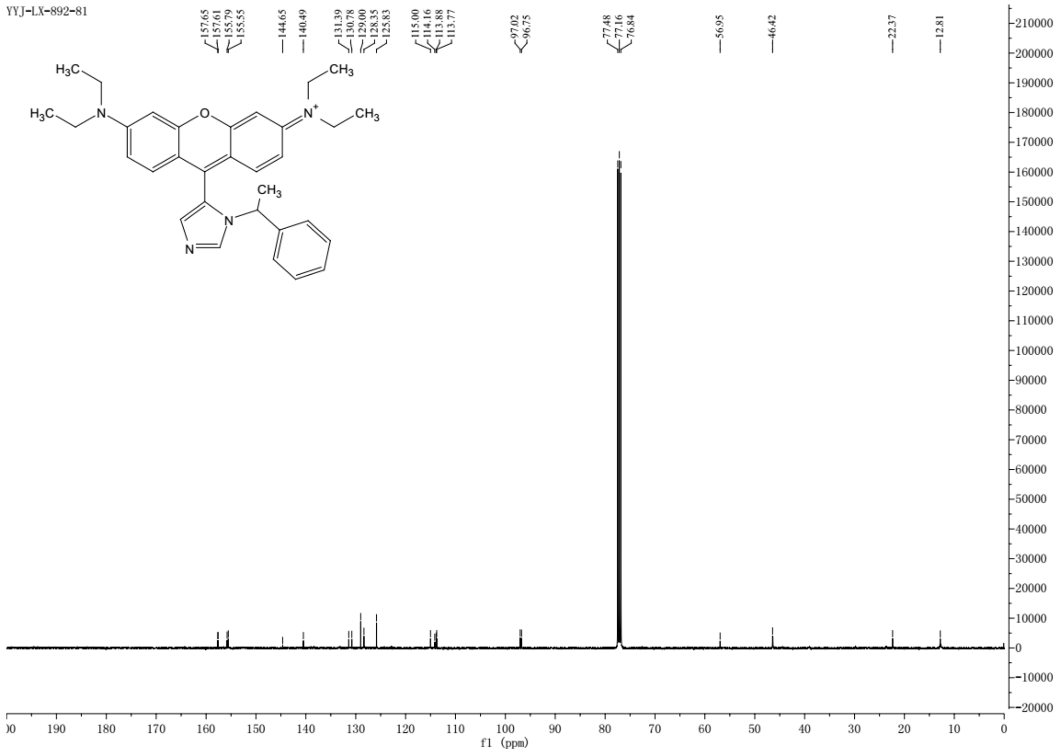

Supplementary Figure 339. The ^{13}C-NMR of compound **RD64** in $CDCl_3$

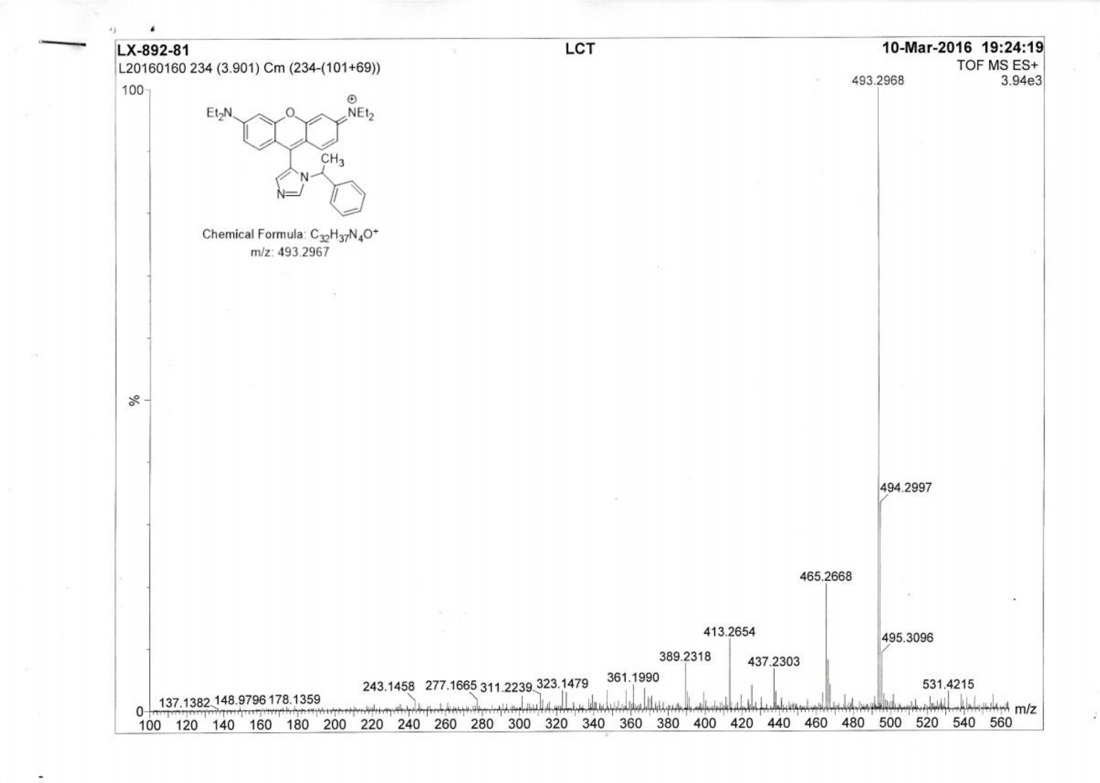

Supplementary Figure 340. The HR-MS of compound **RD64**

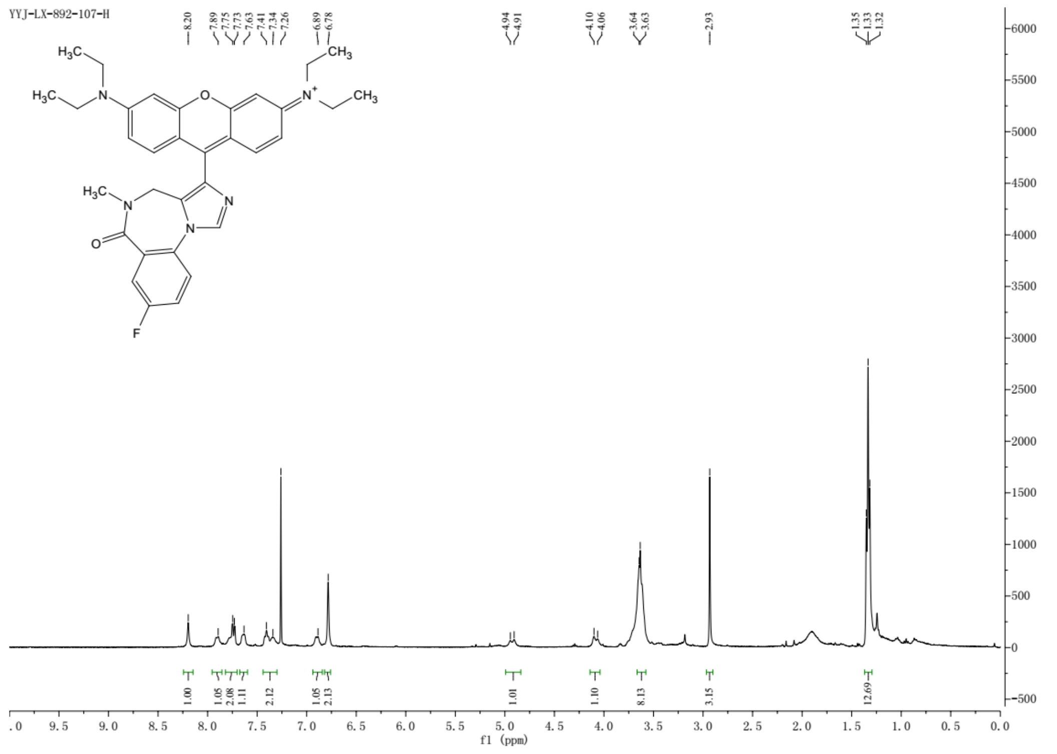

Supplementary Figure 341. The ^{1}H-NMR of compound **RD65** in $CDCl_3$

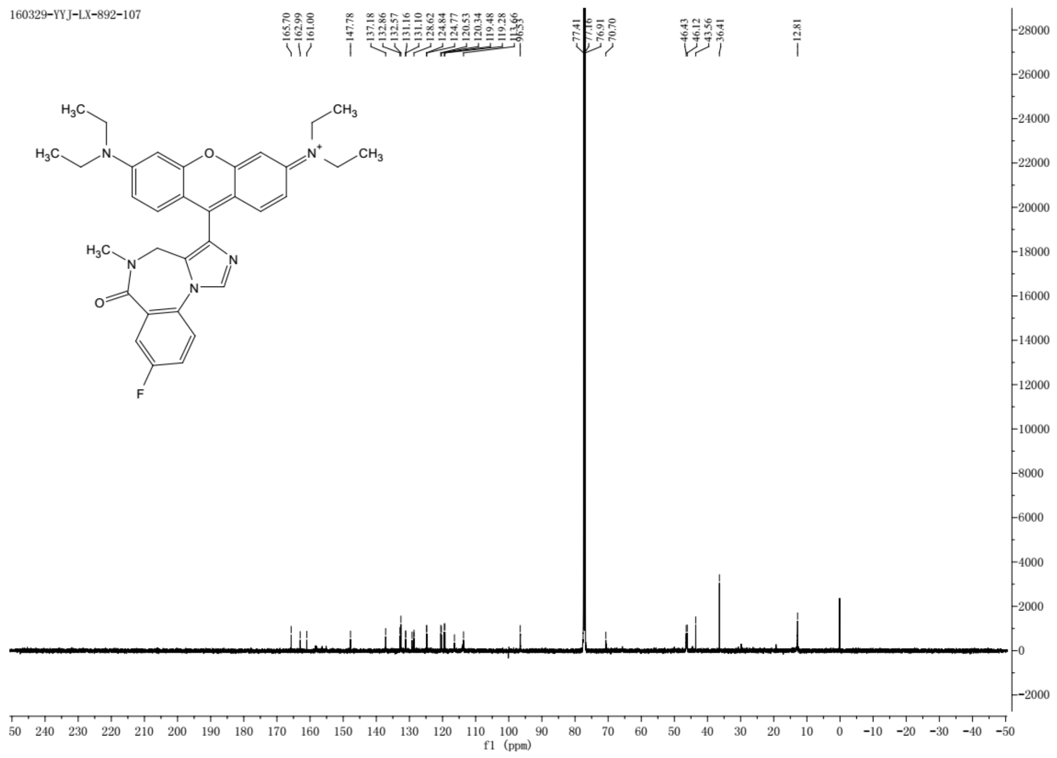

Supplementary Figure 342. The ^{13}C-NMR of compound **RD65** in $CDCl_3$

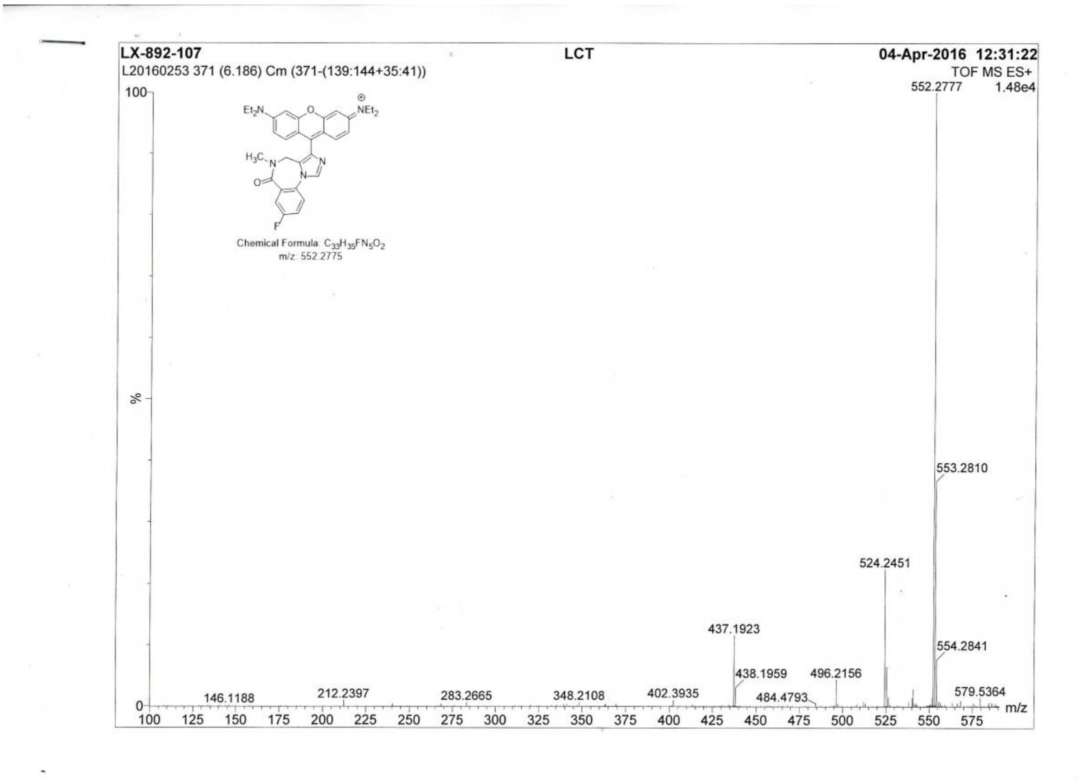

Supplementary Figure 343. The HR-MS of compound **RD65**

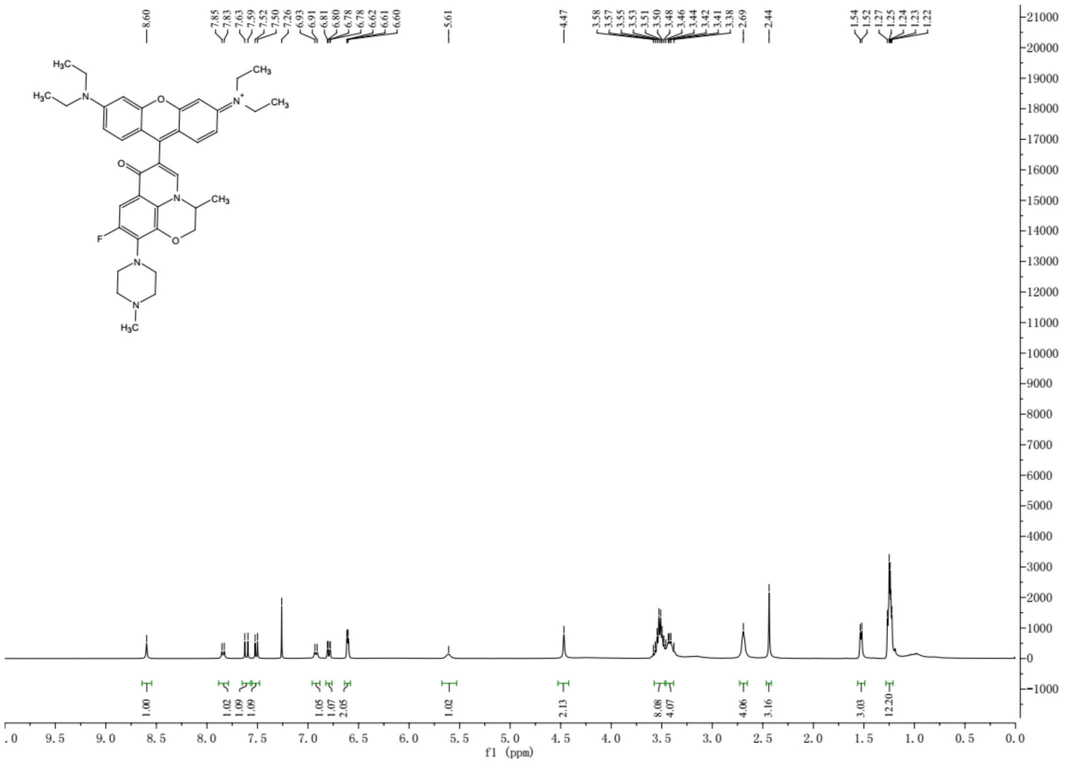

Supplementary Figure 344. The ^{1}H-NMR of compound **RD66** in $CDCl_3$

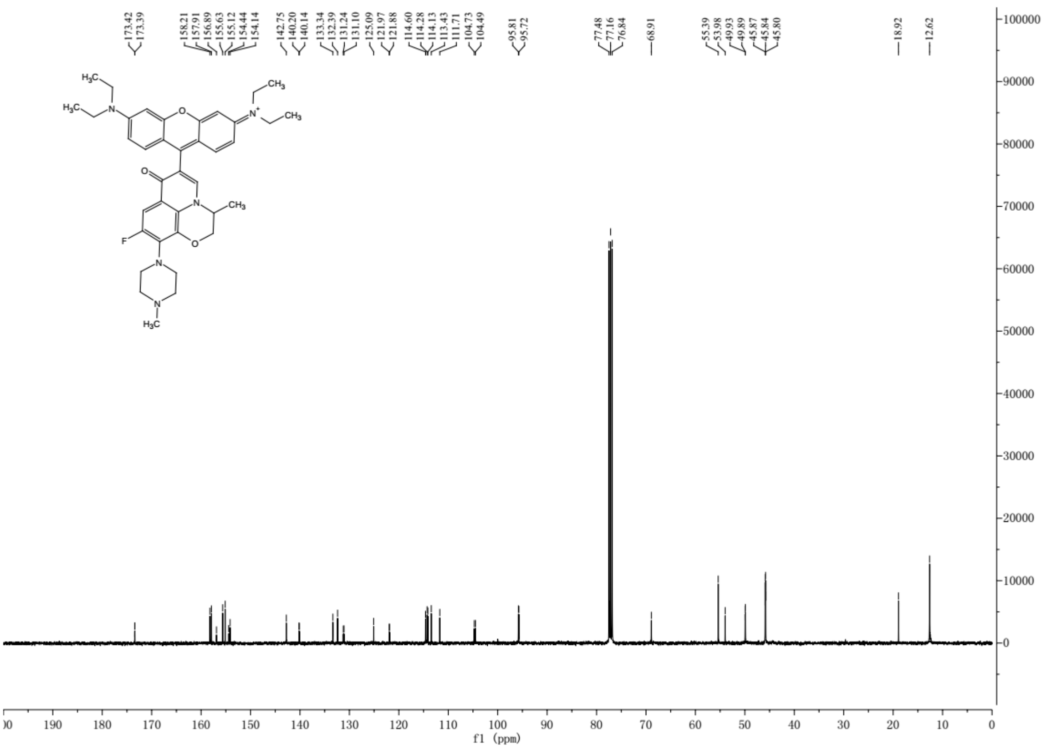

Supplementary Figure 345. The ^{13}C-NMR of compound **RD66** in $CDCl_3$

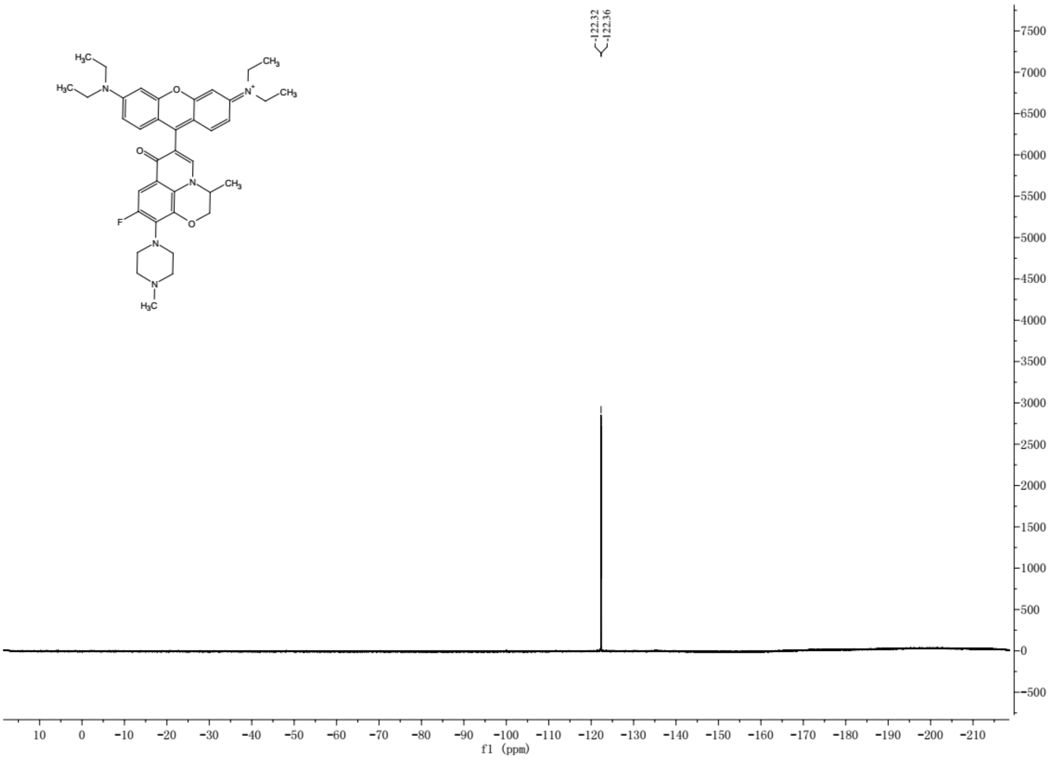

Supplementary Figure 346. The ^{19}F-NMR of compound **RD66** in $CDCl_3$

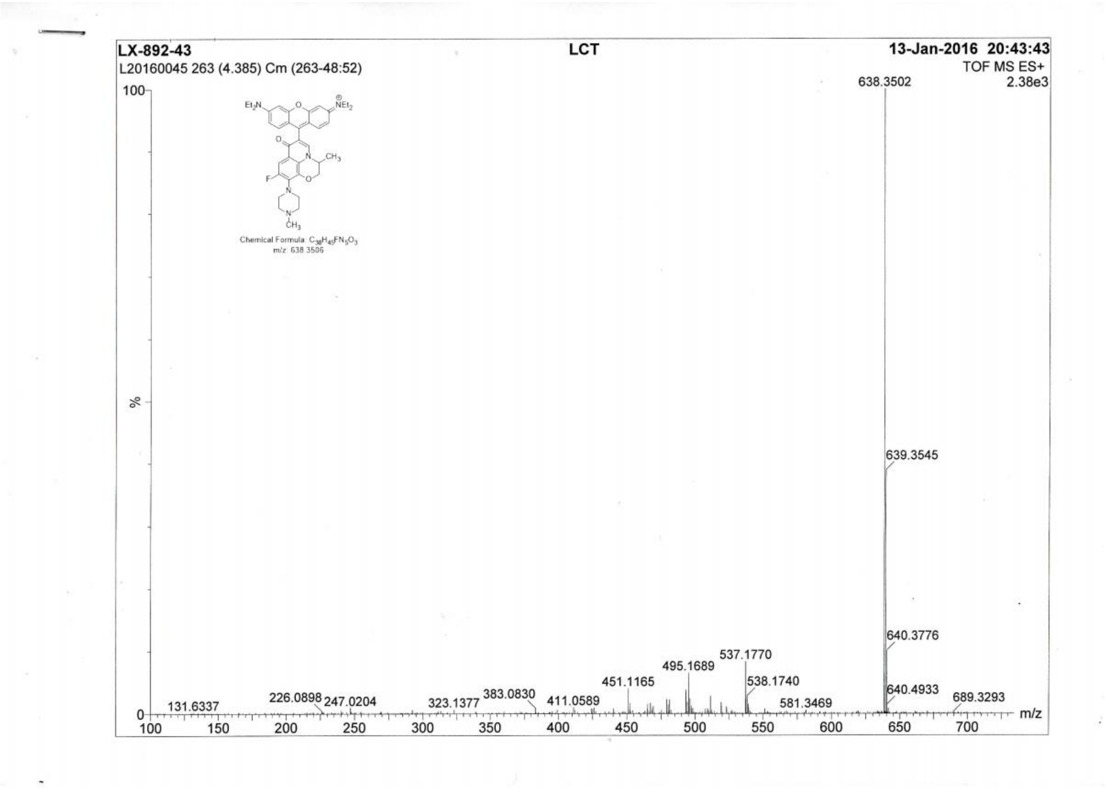

Supplementary Figure 347. The HR-MS of compound **RD66**

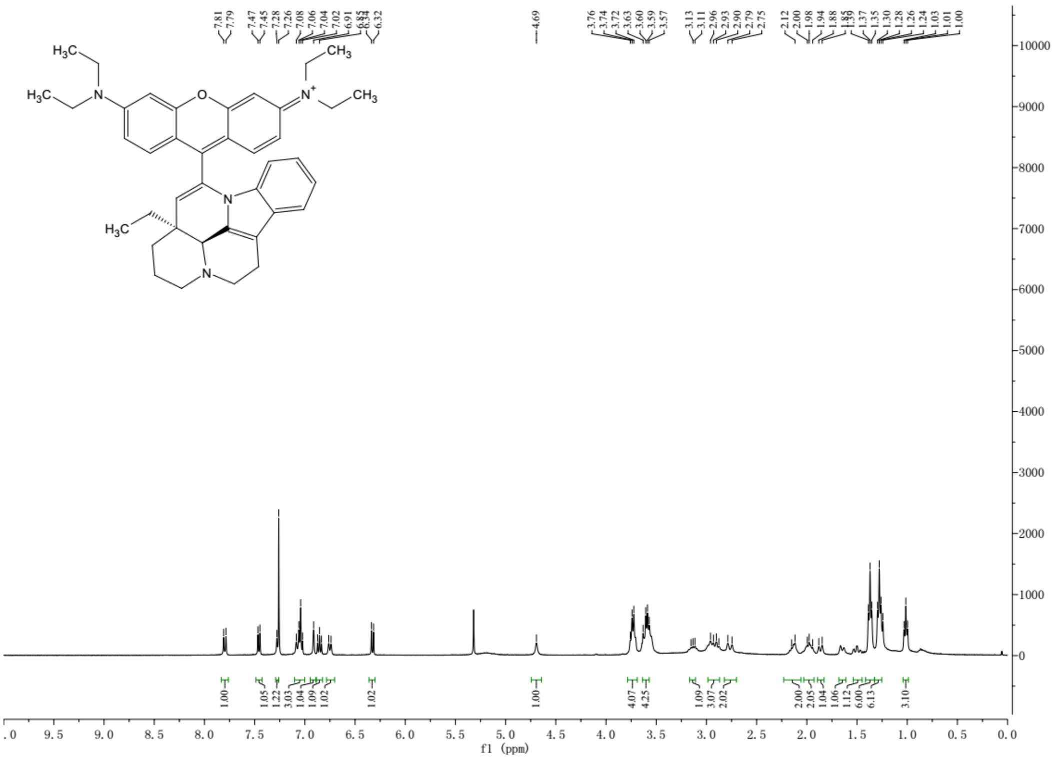

Supplementary Figure 348. The ^{1}H-NMR of compound **RD67** in $CDCl_3$

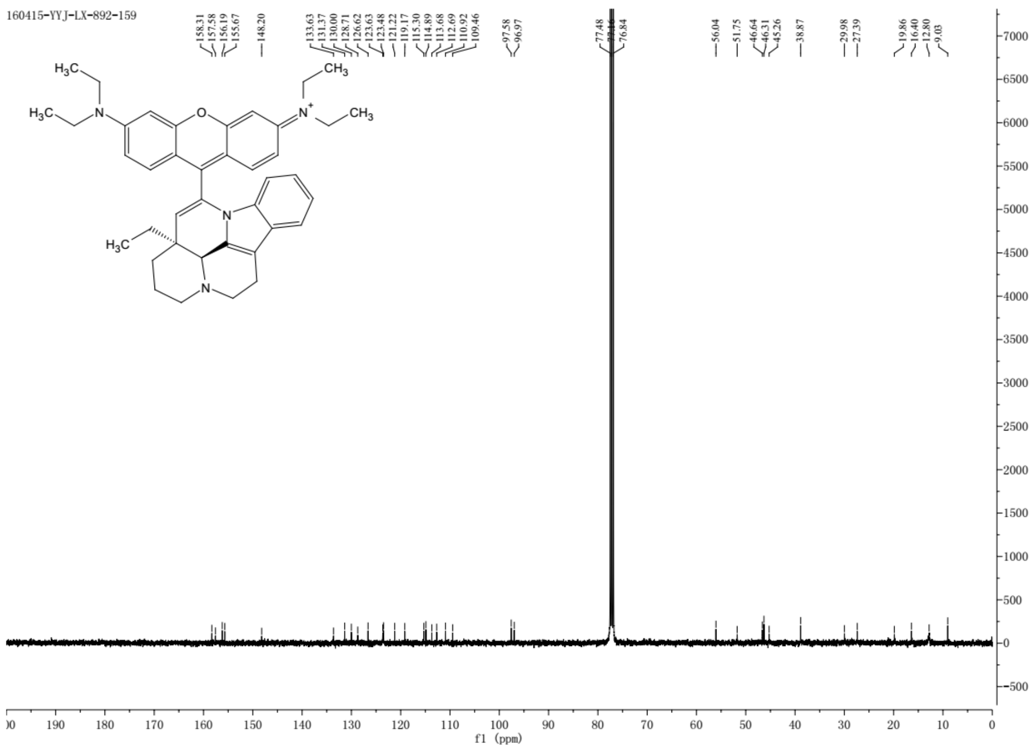

Supplementary Figure 349. The ^{13}C-NMR of compound **RD67** in $CDCl_3$

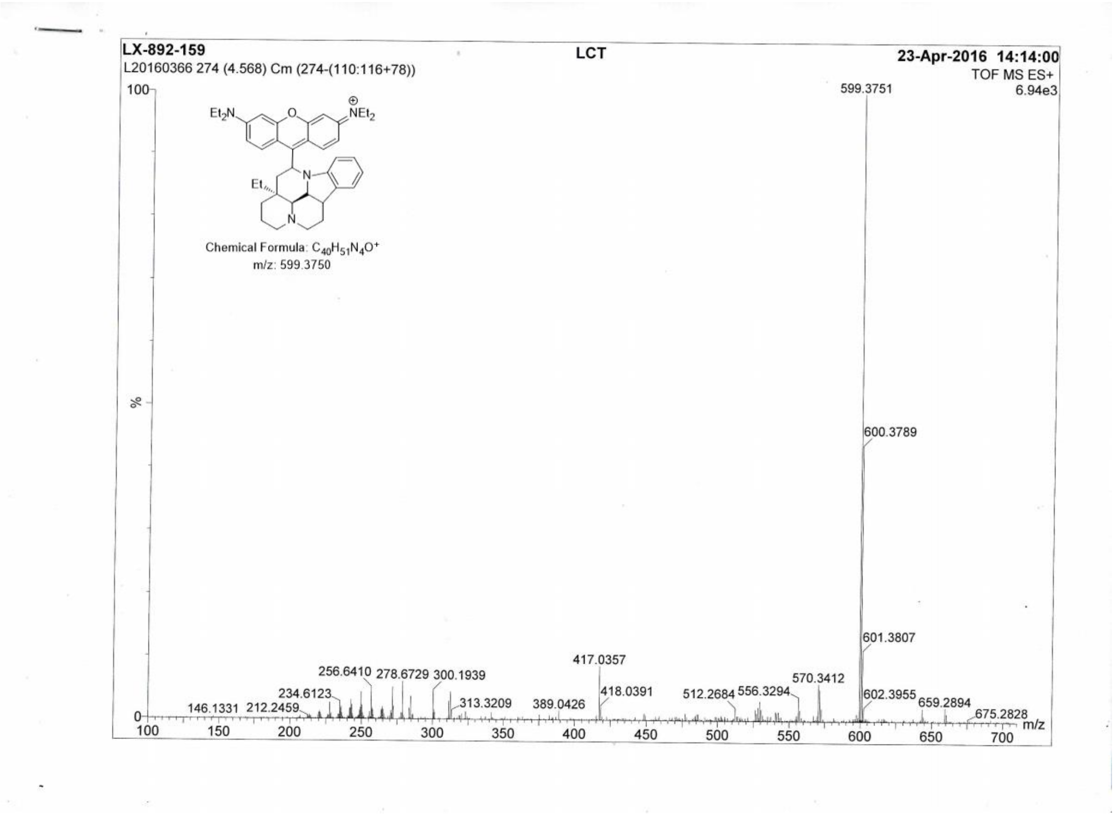

Supplementary Figure 350. The HR-MS of compound **RD67**

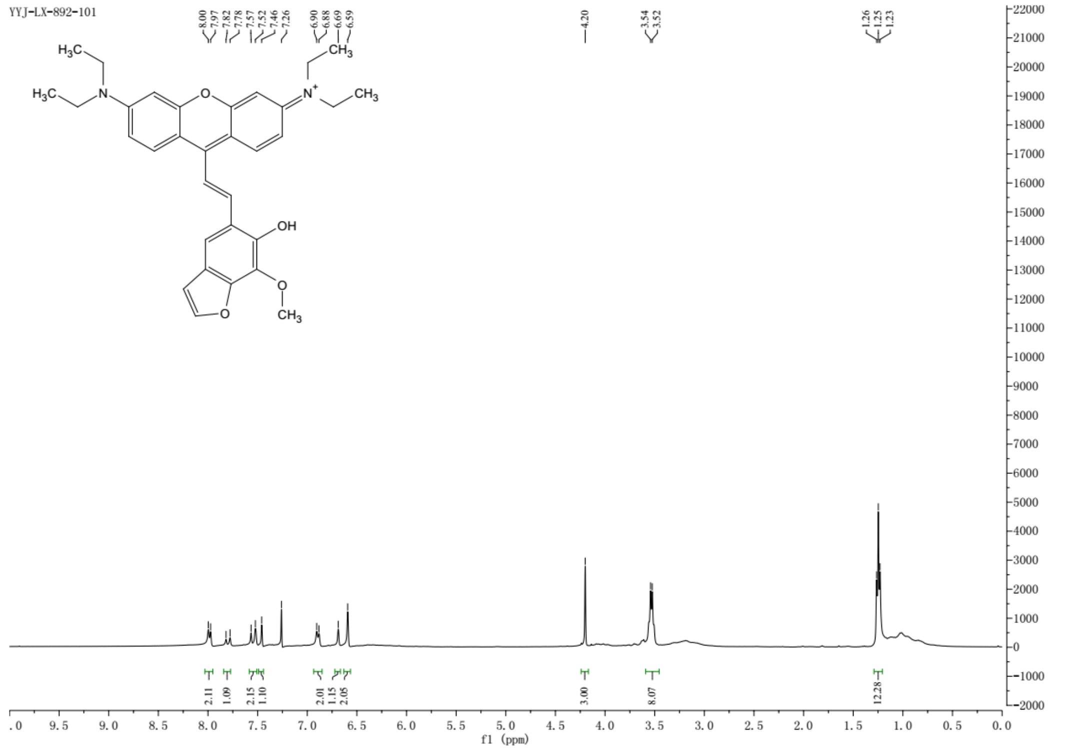

Supplementary Figure 351. The ^{1}H-NMR of compound **RD68** in $CDCl_3$

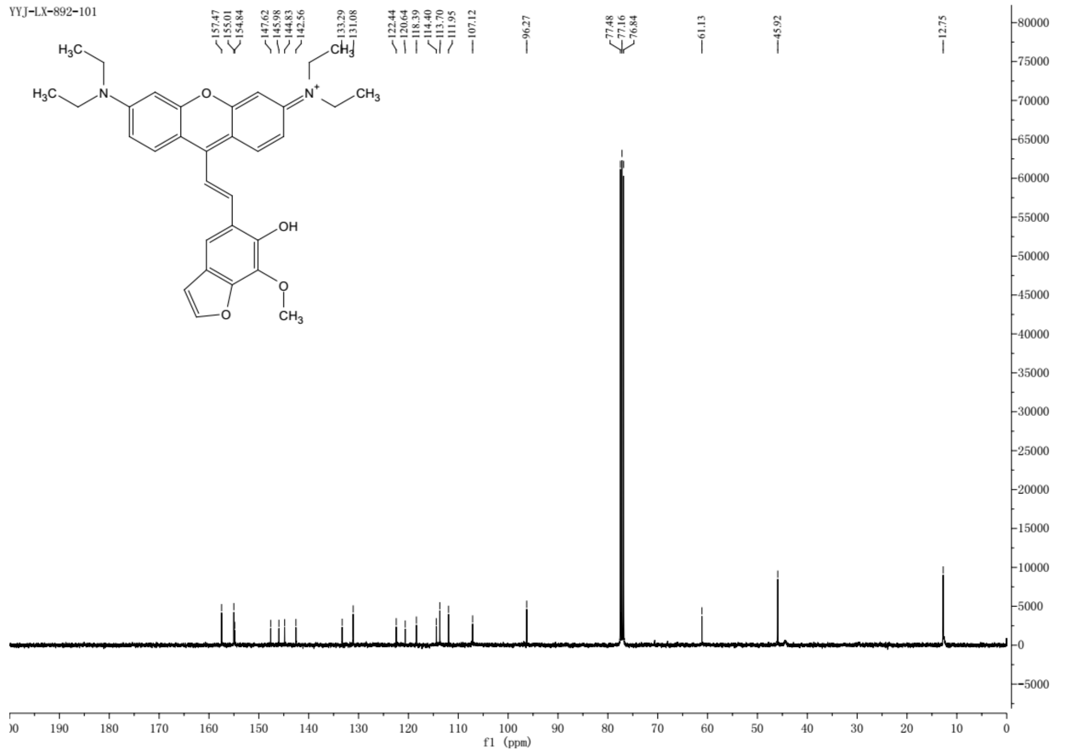

Supplementary Figure 352. The ^{13}C-NMR of compound **RD68** in $CDCl_3$

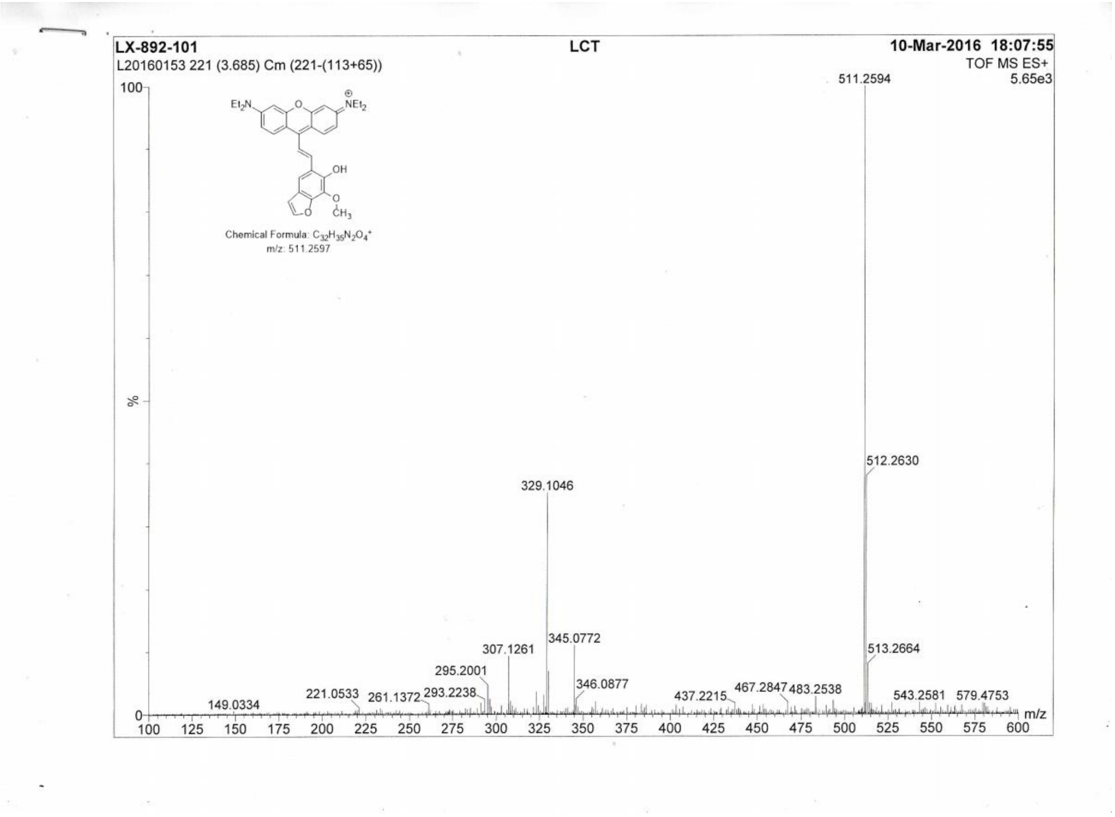

Supplementary Figure 353. The HR-MS of compound **RD68**

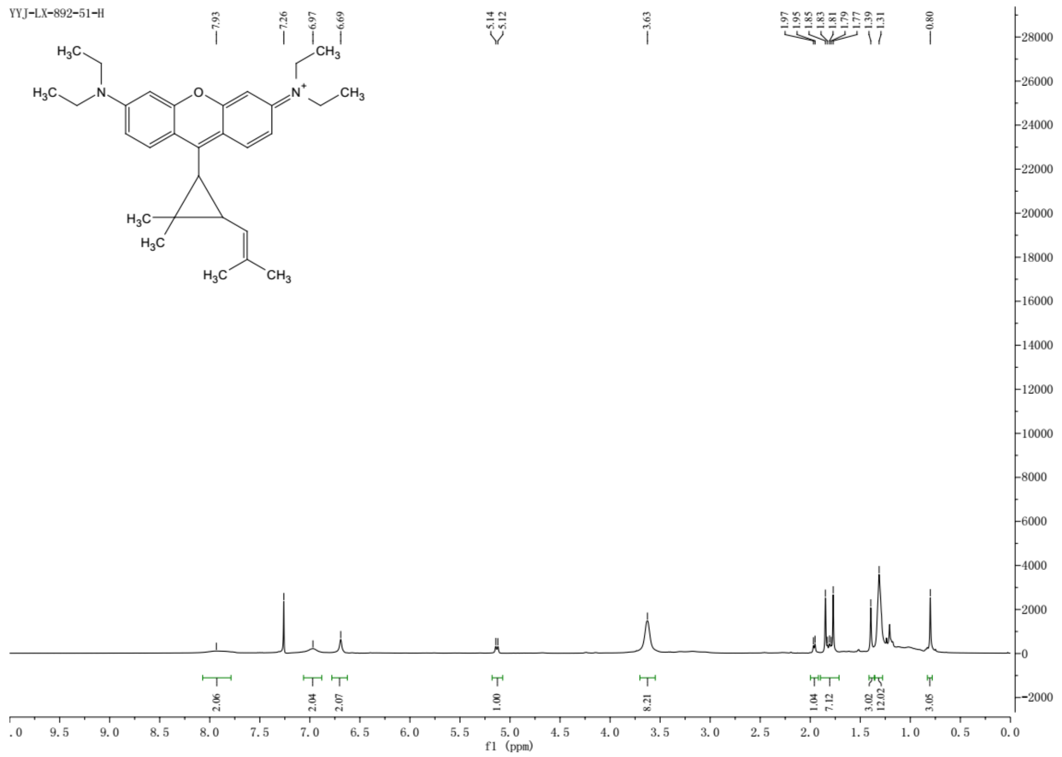

Supplementary Figure 354. The ^{1}H-NMR of compound **RD69** in $CDCl_3$

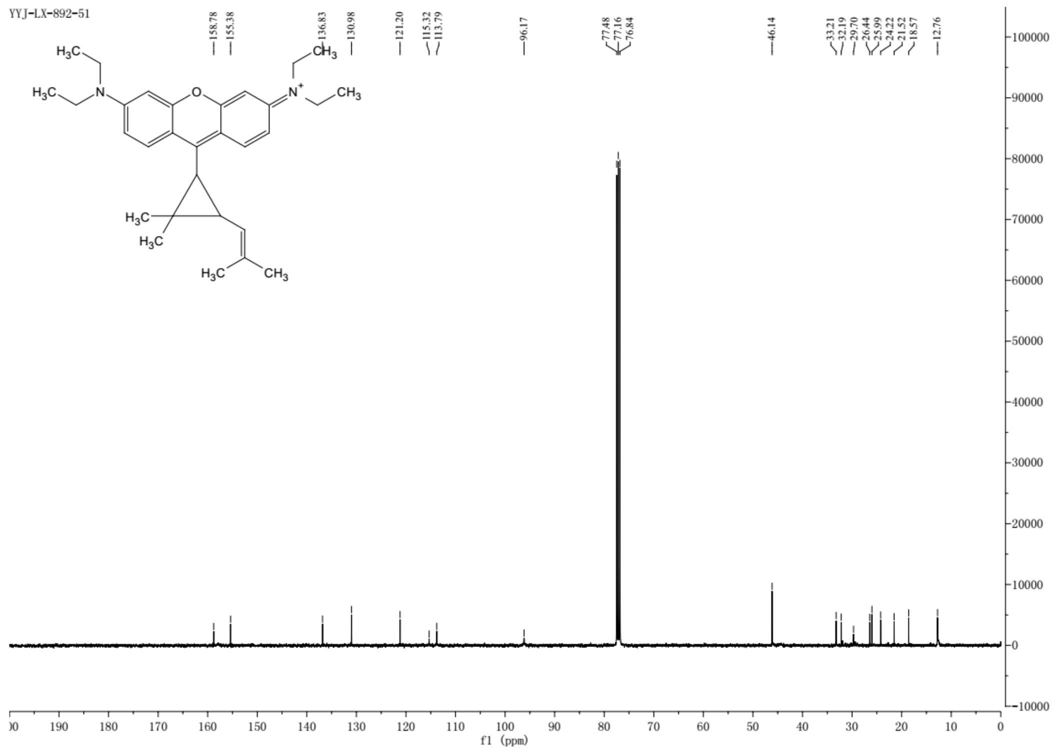

Supplementary Figure 355. The ^{13}C-NMR of compound **RD69** in $CDCl_3$

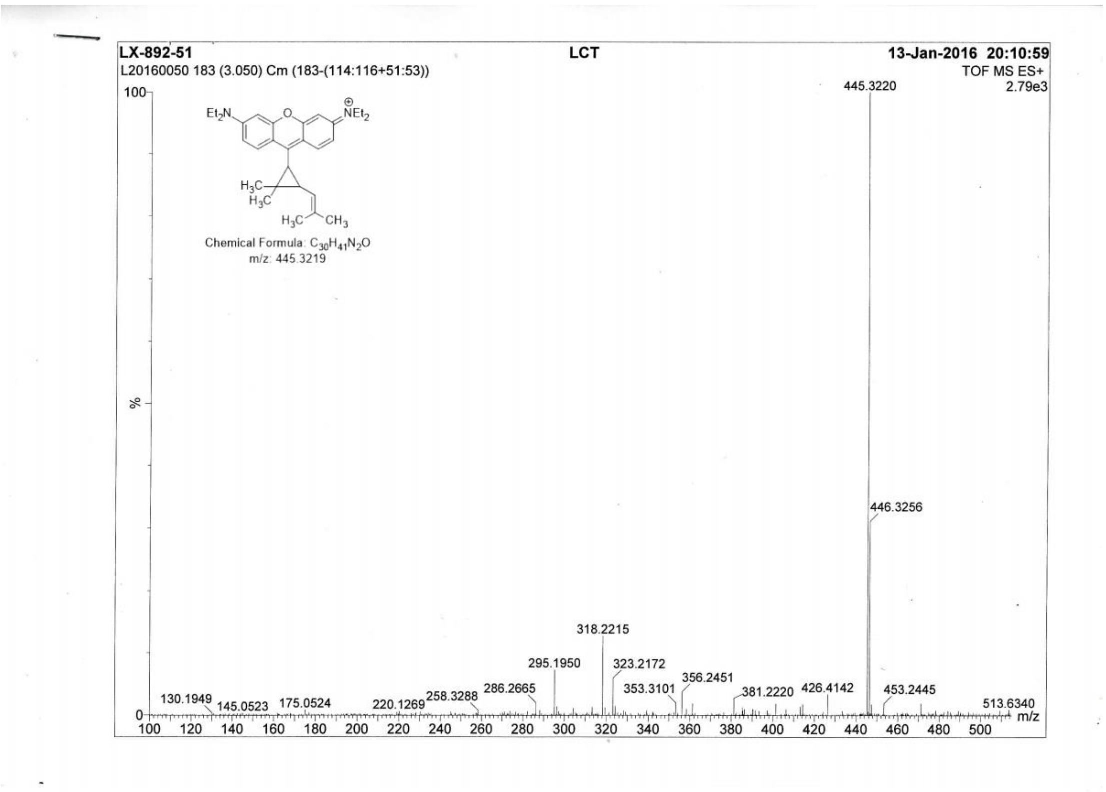

Supplementary Figure 356. The HR-MS of compound **RD69**

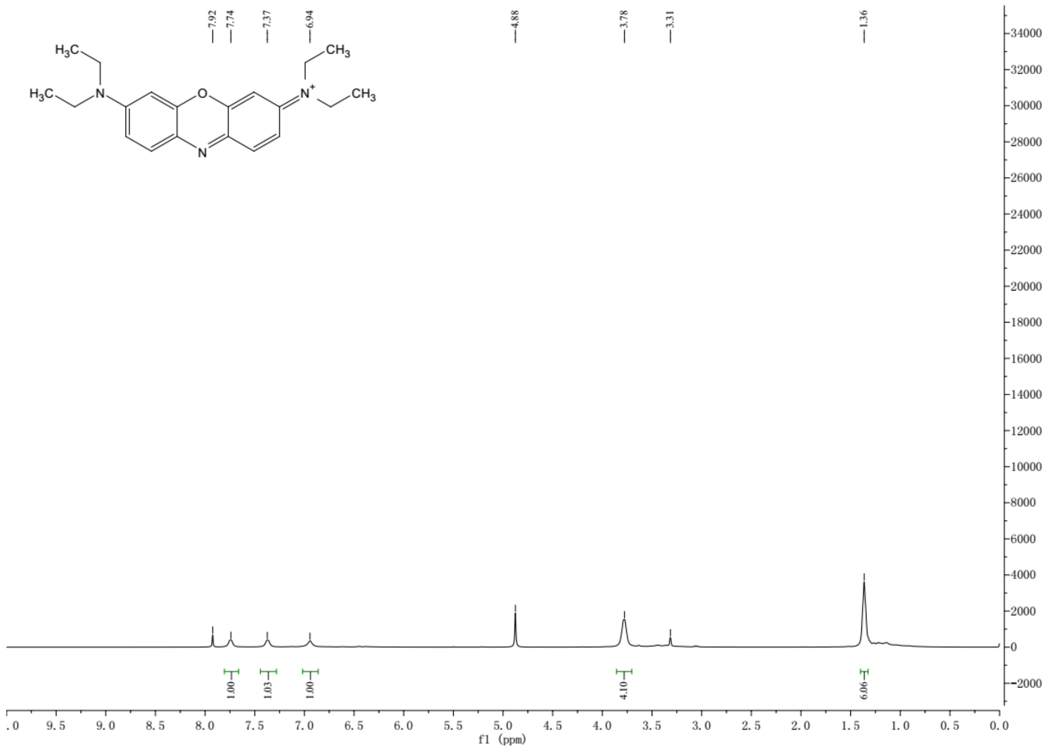

Supplementary Figure 357. The ^{1}H-NMR of compound **RD70** in CD_3OD.

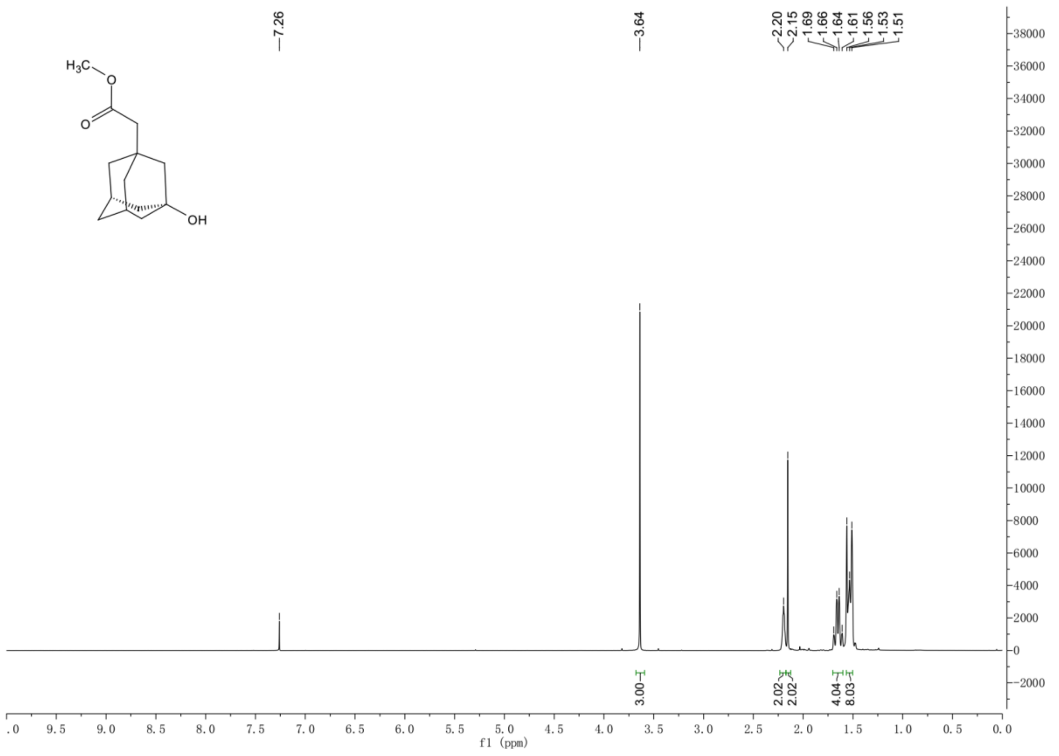

Supplementary Figure 358. The ^{1}H-NMR of compound **S71** in $CDCl_3$

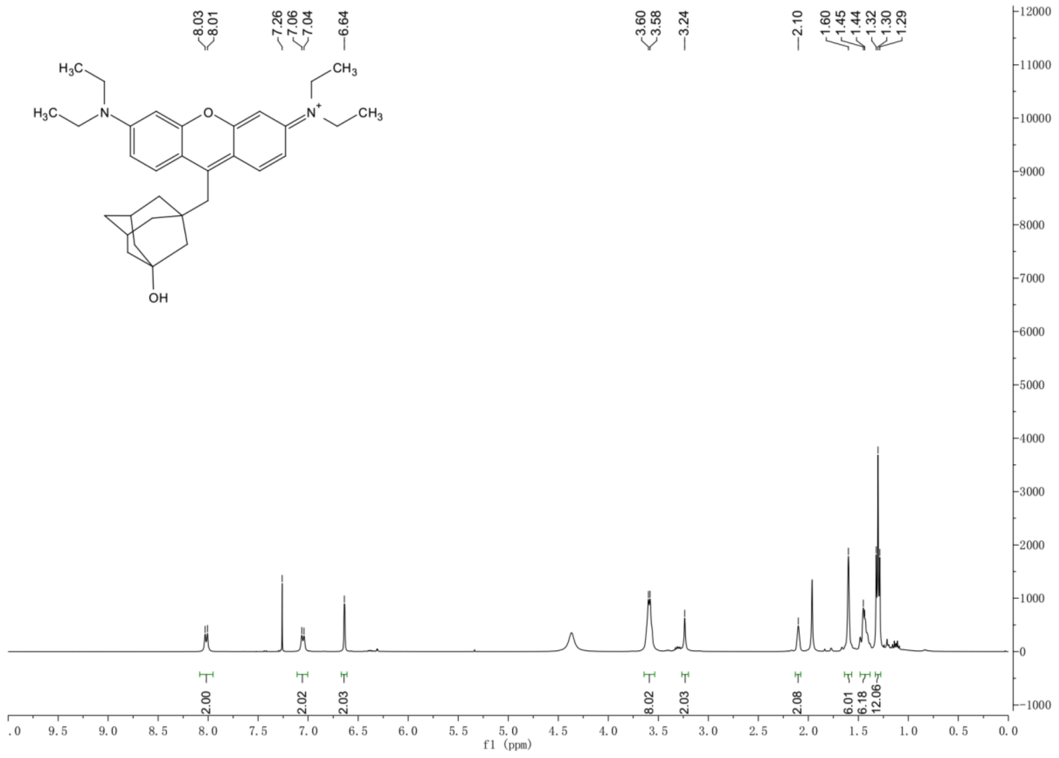

Supplementary Figure 359. The ^{1}H-NMR of compound **RD71** in $CDCl_3$

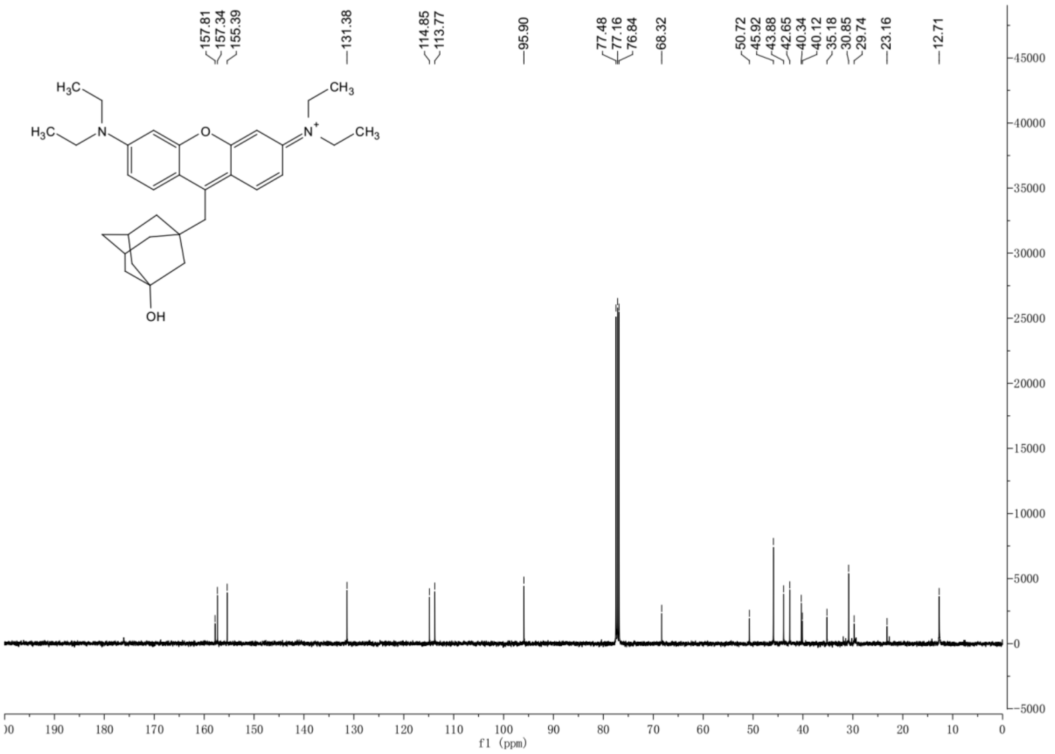

Supplementary Figure 360. The ^{13}C-NMR of compound **RD71** in $CDCl_3$

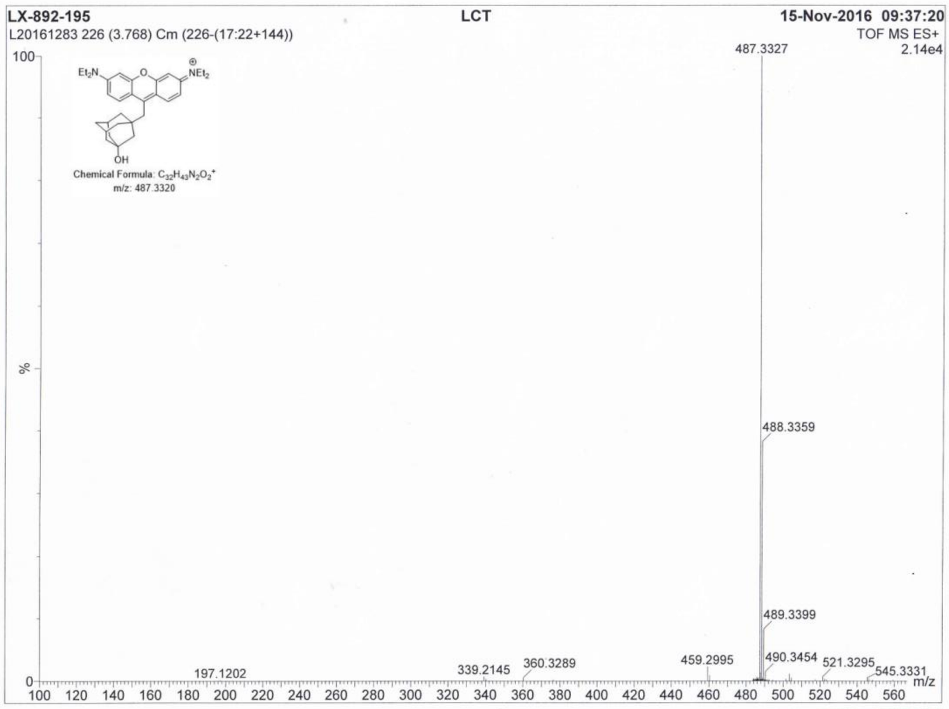

Supplementary Figure 361. The HR-MS of compound **RD71**

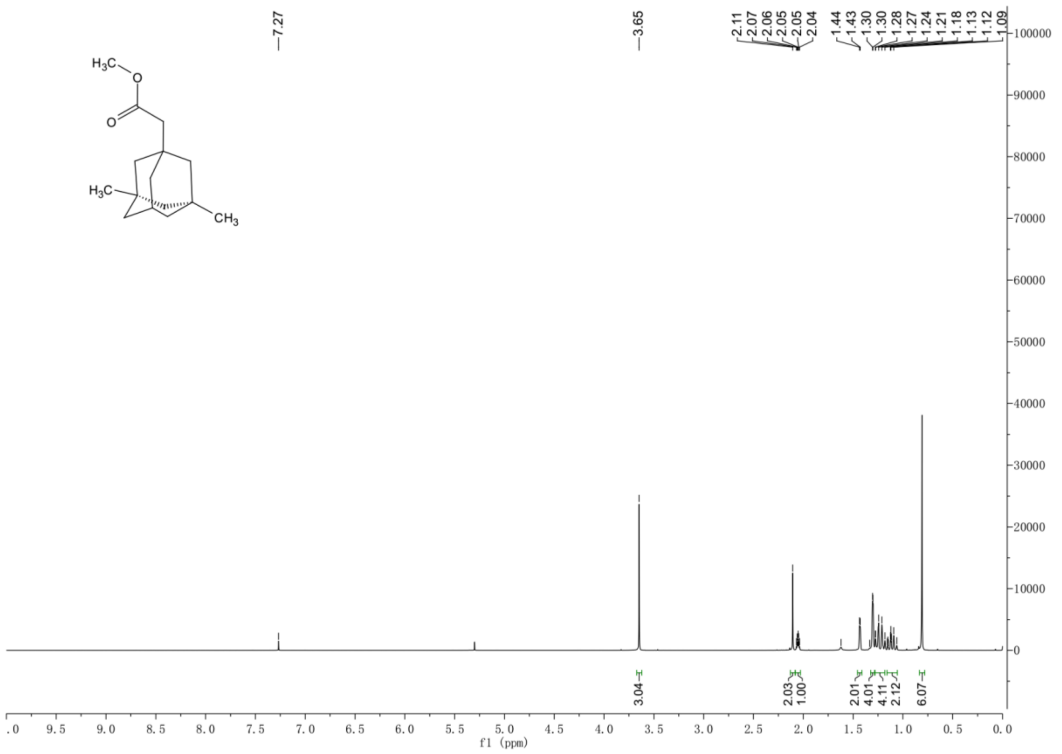

Supplementary Figure 362. The ^{1}H-NMR of compound **S72** in $CDCl_3$

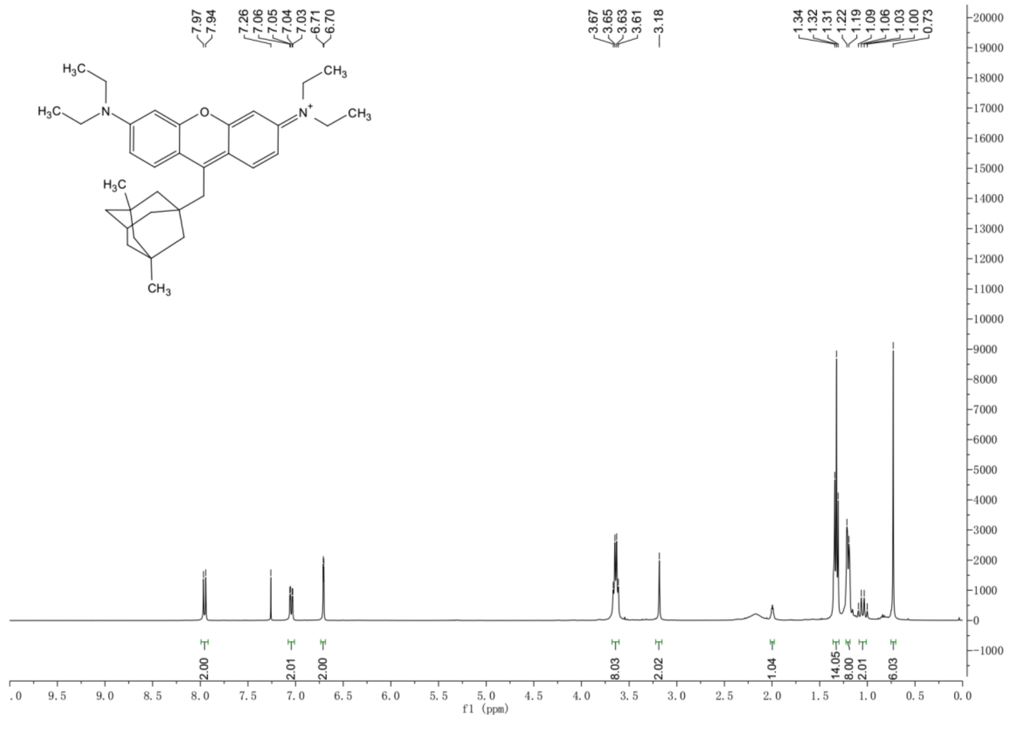

Supplementary Figure 363. The ^{1}H-NMR of compound **RD72** in $CDCl_3$

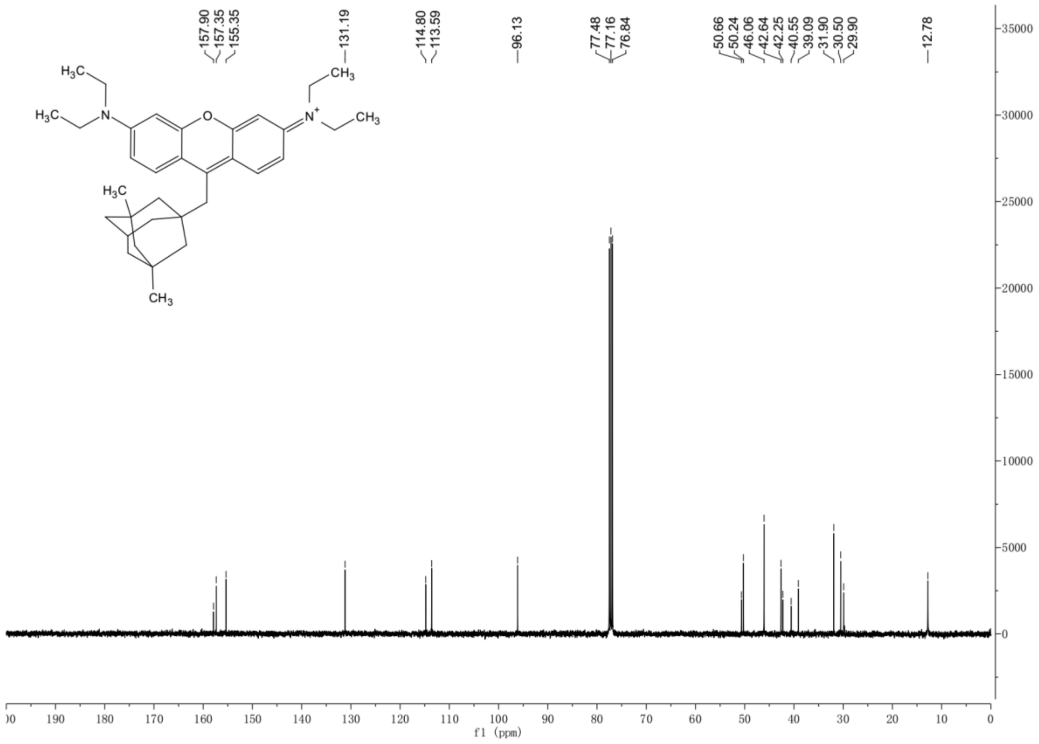

Supplementary Figure 364. The ^{13}C-NMR of compound **RD72** in $CDCl_3$

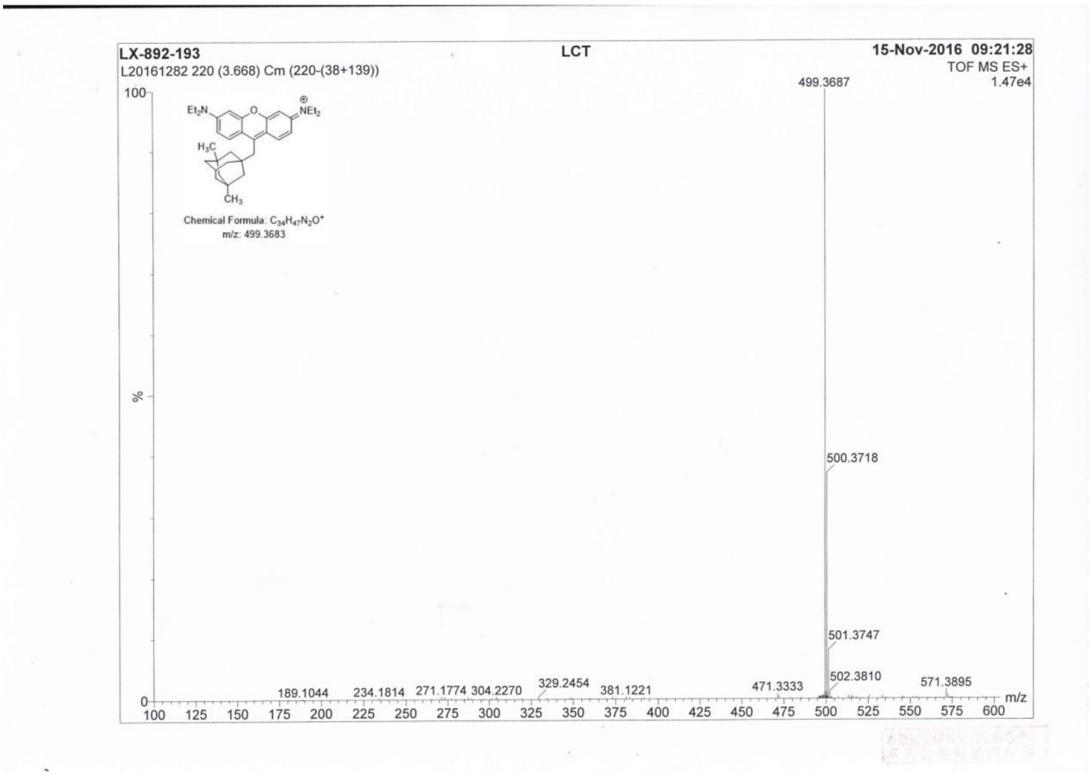

Supplementary Figure 365. The HR-MS of compound **RD72**

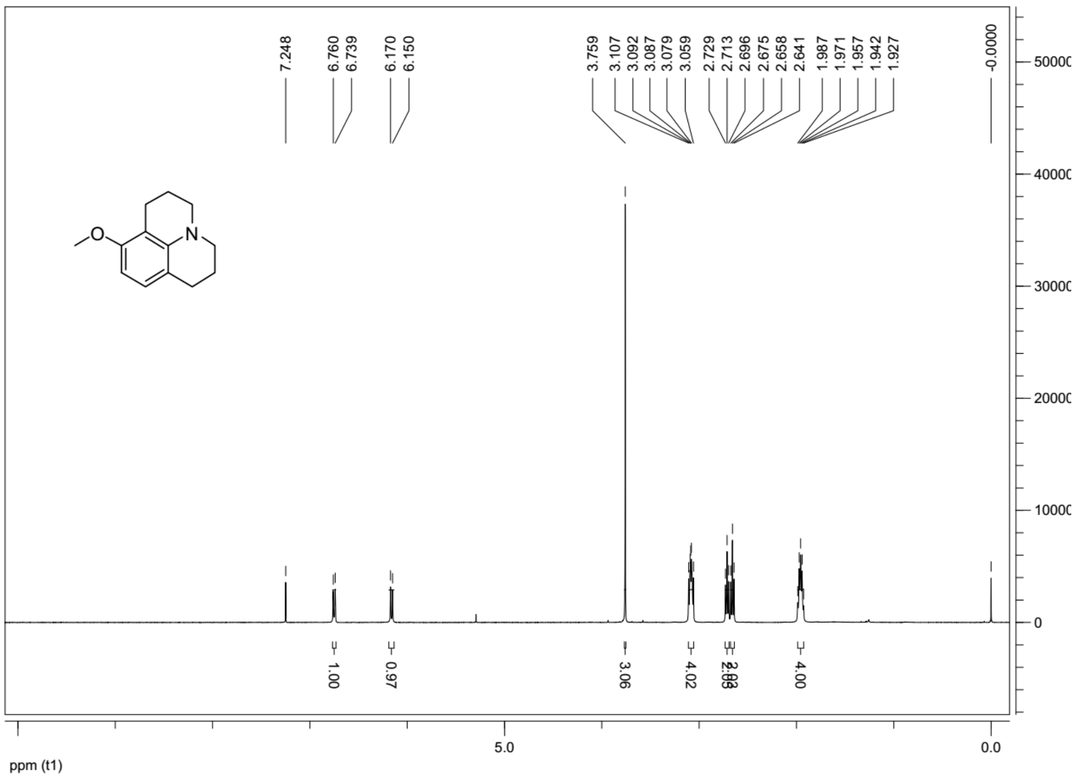

Supplementary Figure 366 The ^{1}H-NMR of compound **73a** in $CDCl_3$

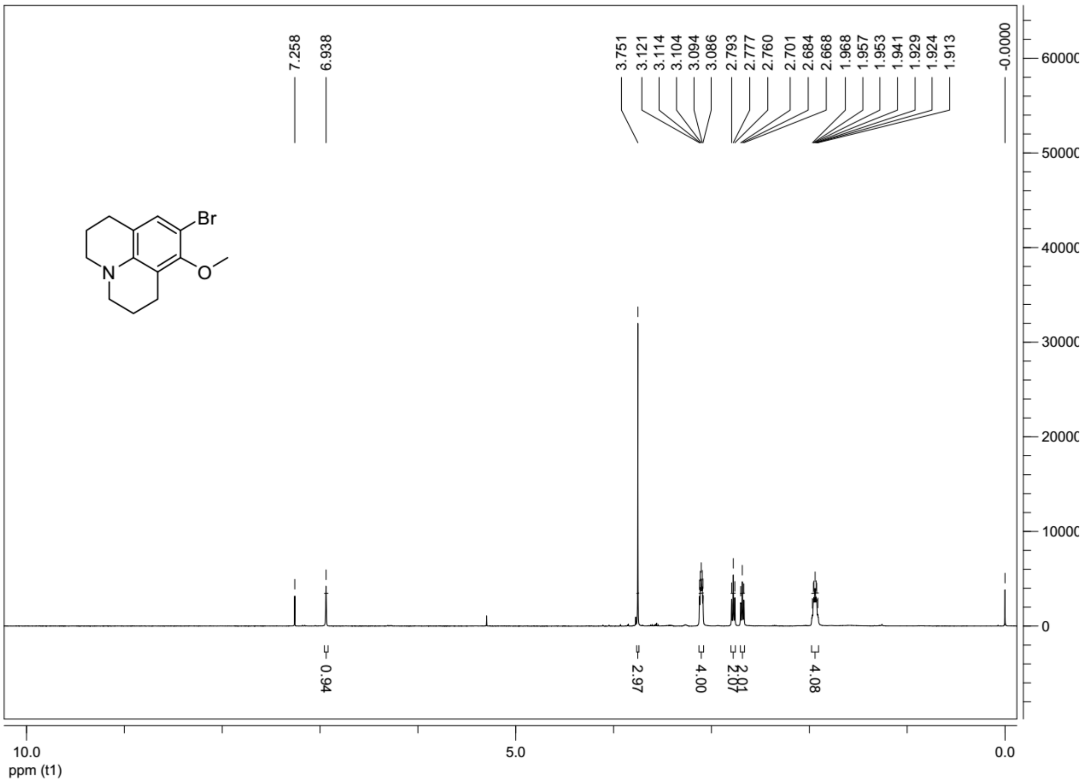

Supplementary Figure 367. The ^{1}H-NMR of compound **73b** in $CDCl_3$

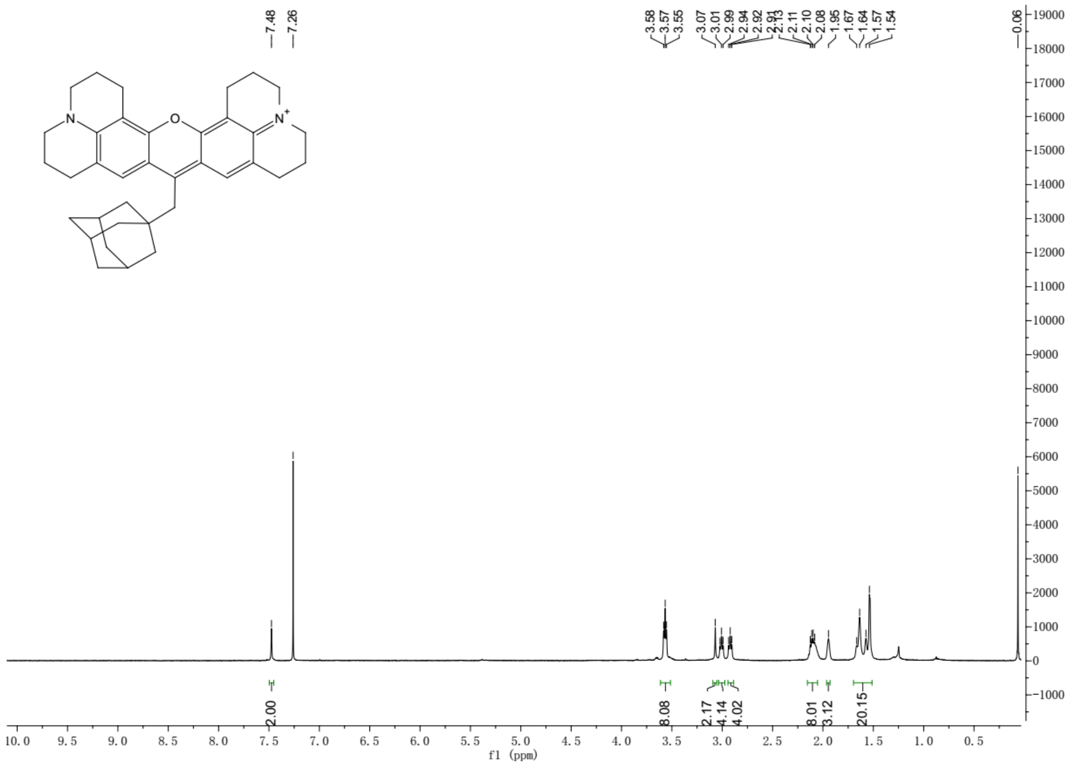

Supplementary Figure 368. The ^{1}H-NMR of compound **RD73** in $CDCl_3$

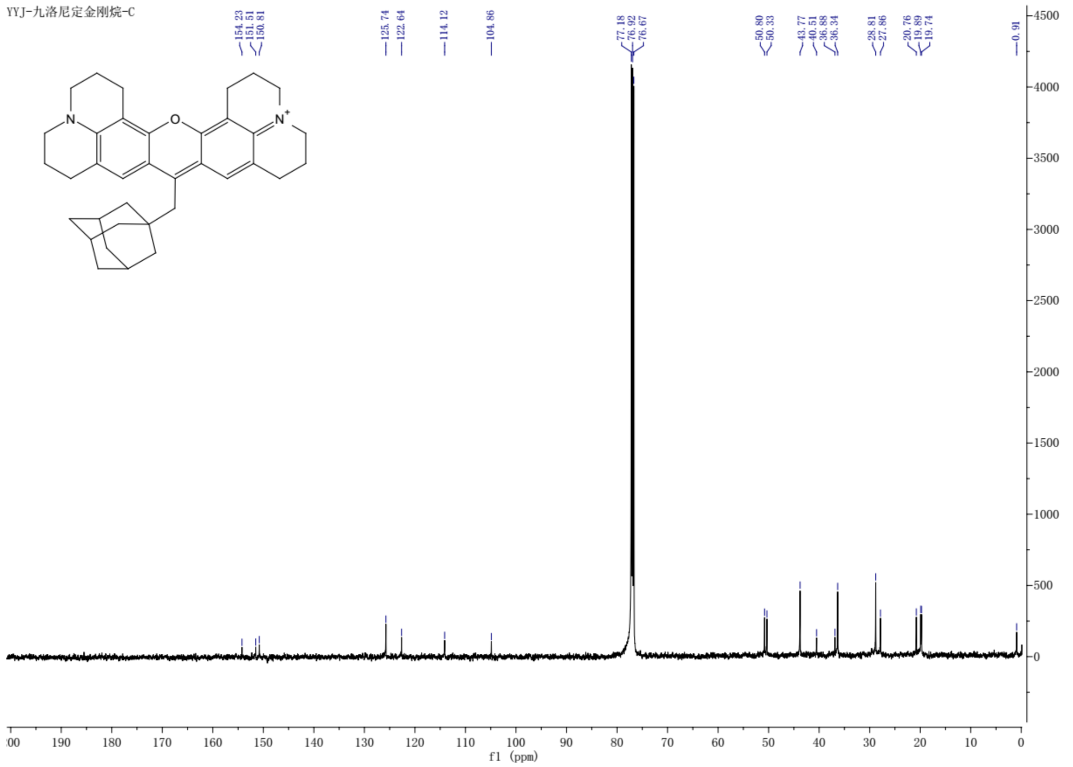

Supplementary Figure 369. The ^{13}C-NMR of compound **RD73** in $CDCl_3$

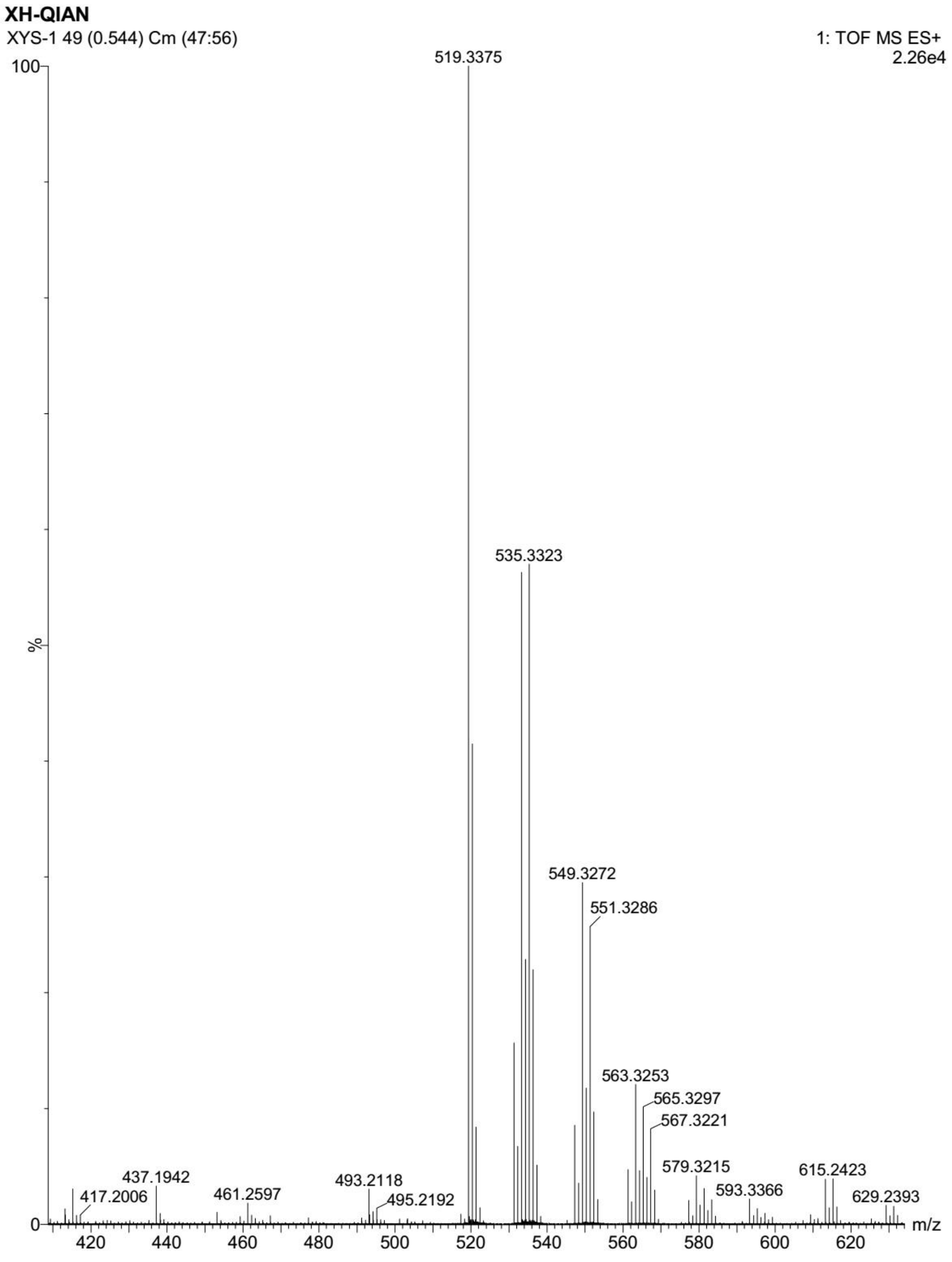

Supplementary Figure 370. The HR-MS of compound **RD73**

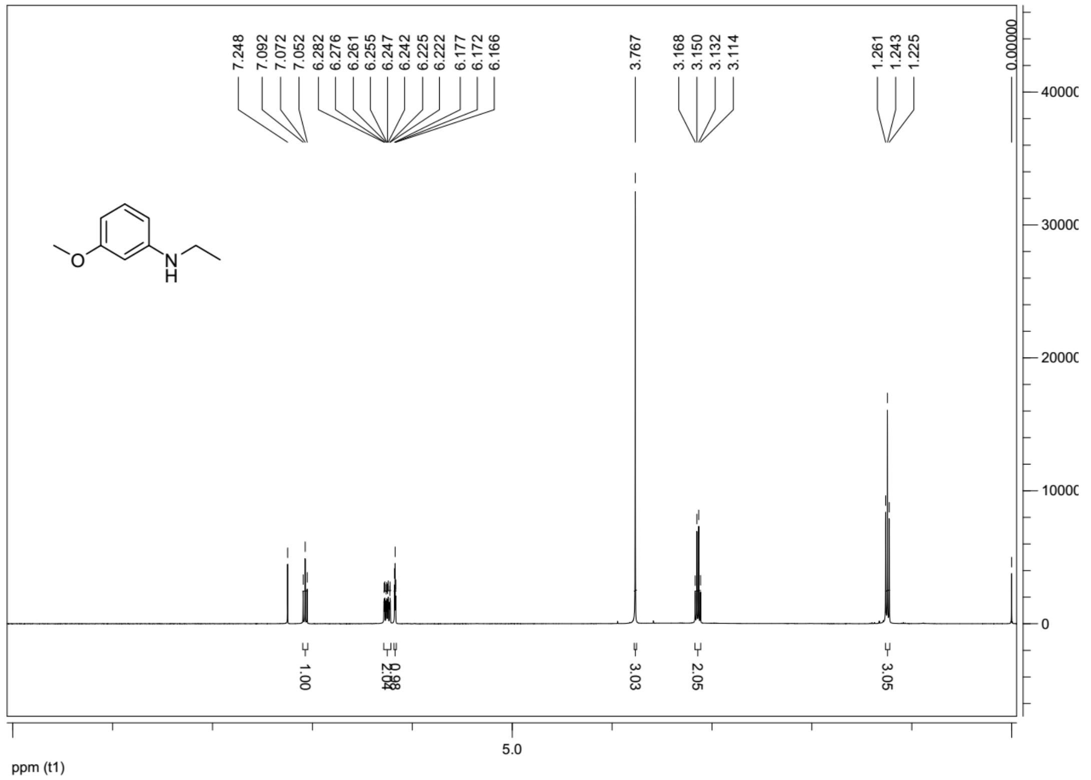

Supplementary Figure 371. The ^{1}H-NMR of compound **74a** in $CDCl_3$

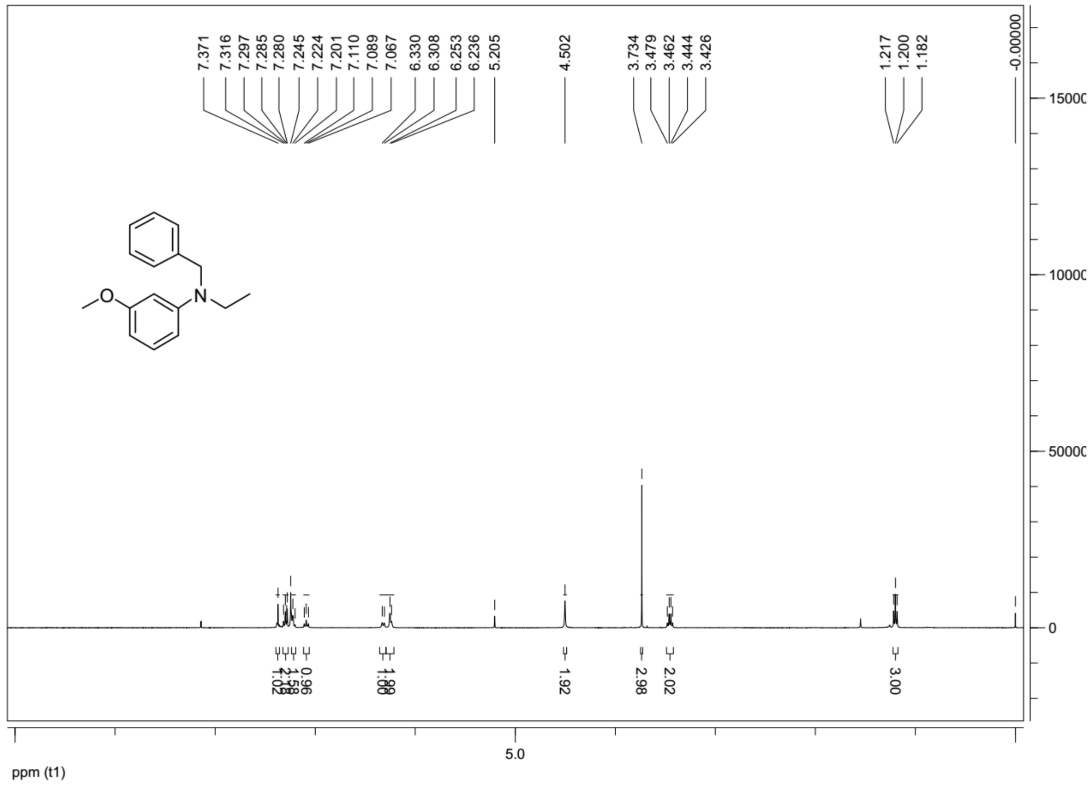

Supplementary Figure 372. The ^{1}H-NMR of compound **74b** in $CDCl_3$

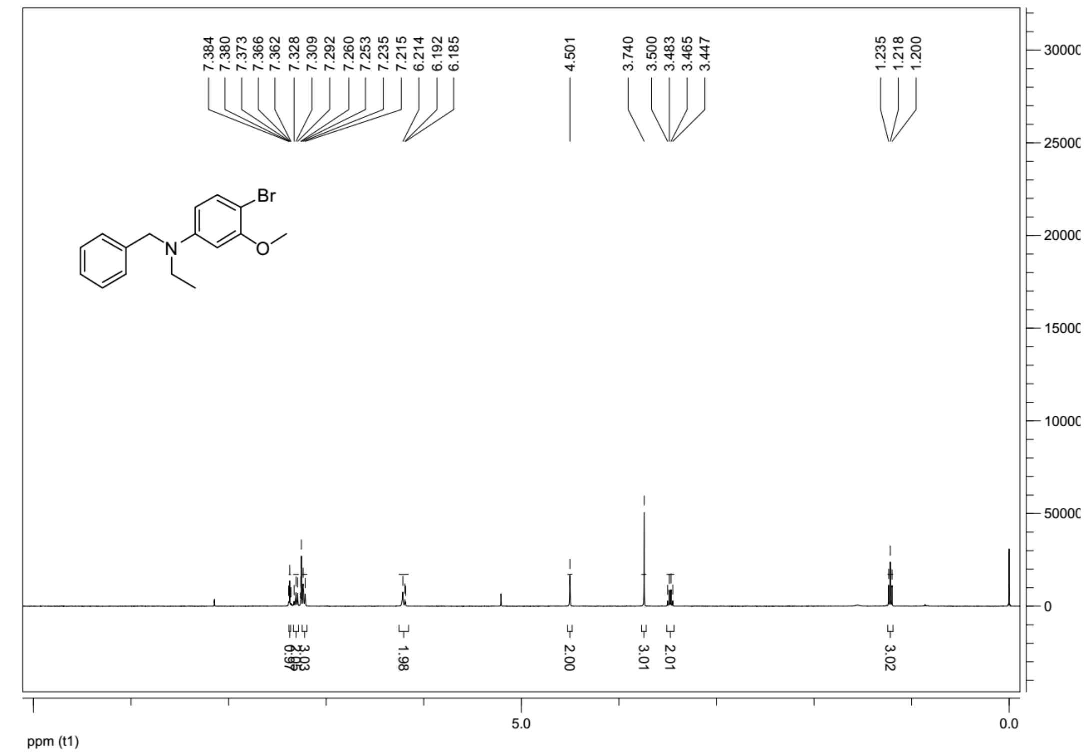

Supplementary Figure 373. The ^{1}H-NMR of compound **74c** in $CDCl_3$

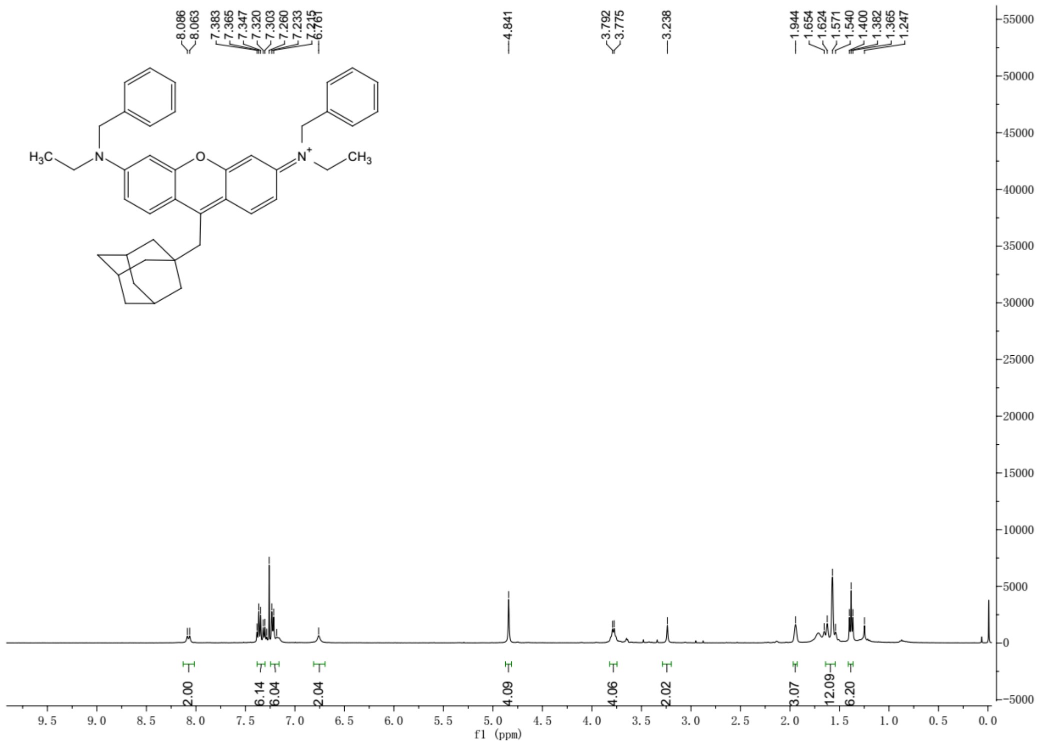

Supplementary Figure 374. The ^{1}H-NMR of compound **RD74** in $CDCl_3$

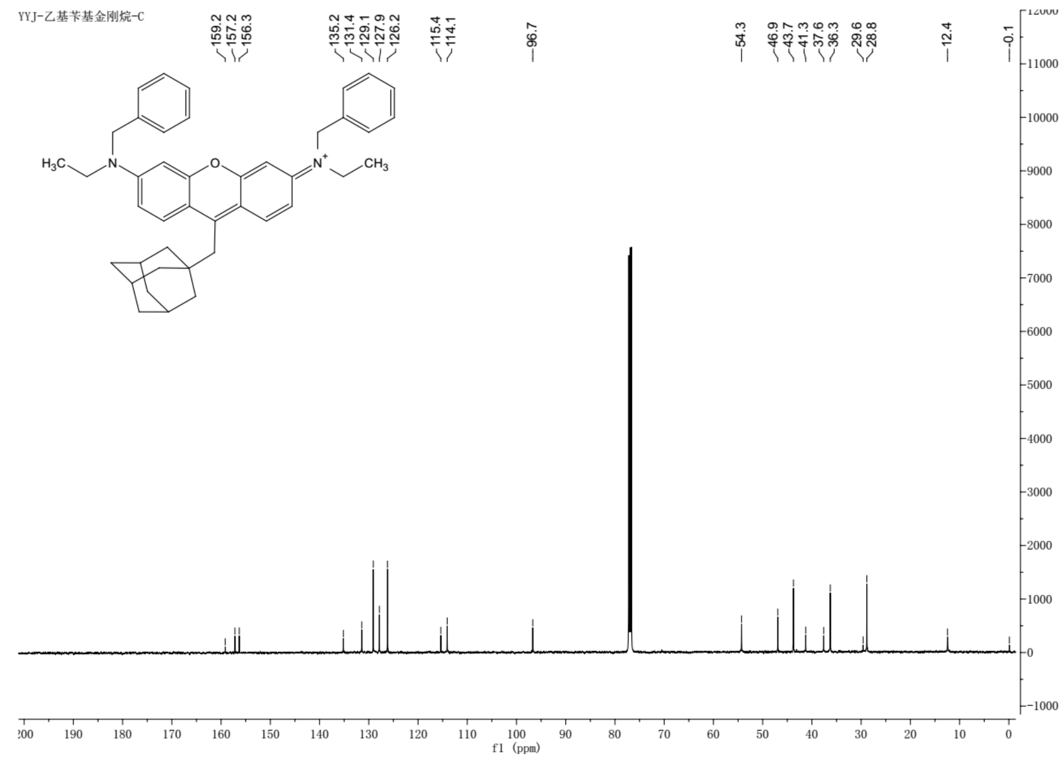

Supplementary Figure 375. The ^{13}C-NMR of compound **RD74** in $CDCl_3$

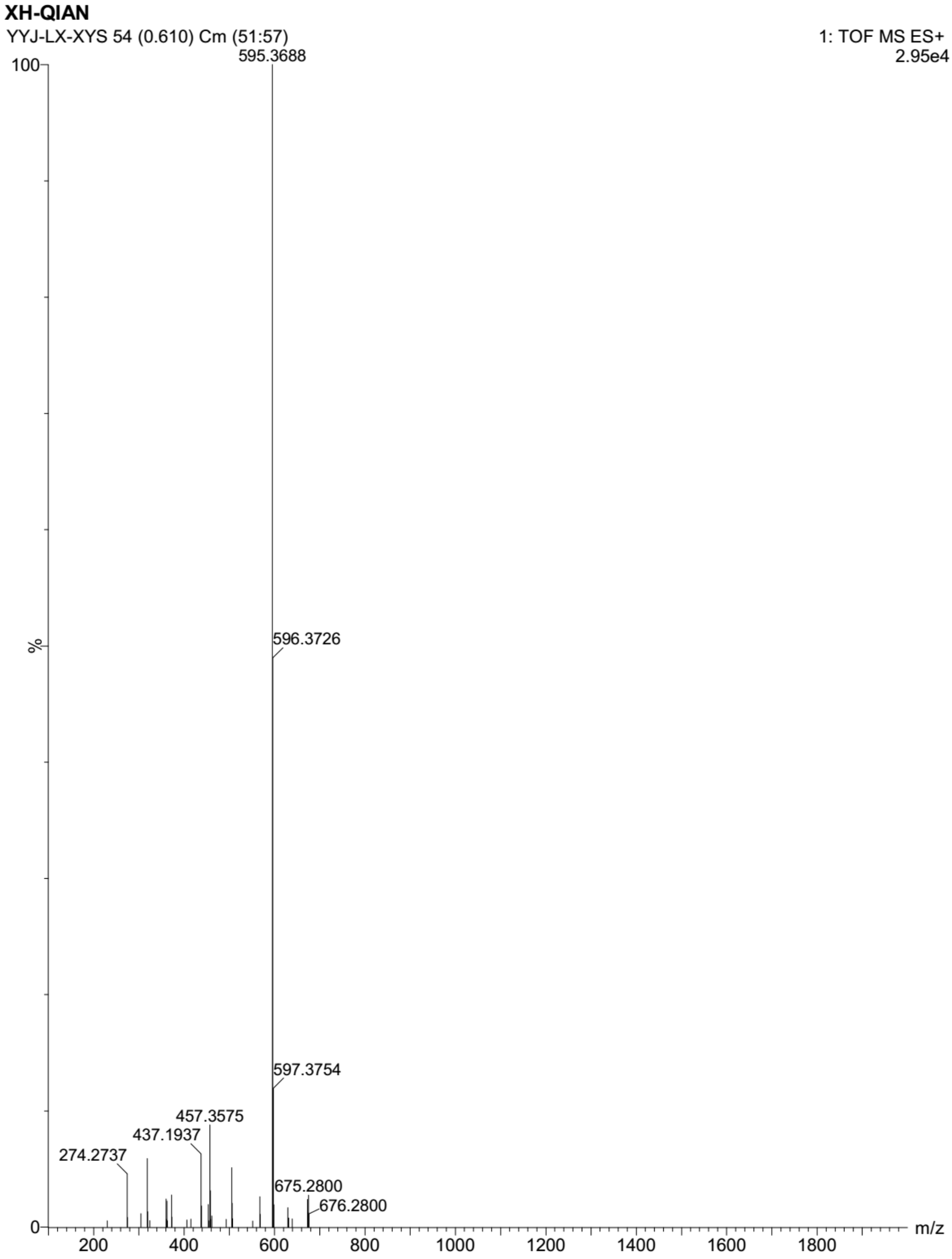

Supplementary Figure 376. The HR-MS of compound **RD74**

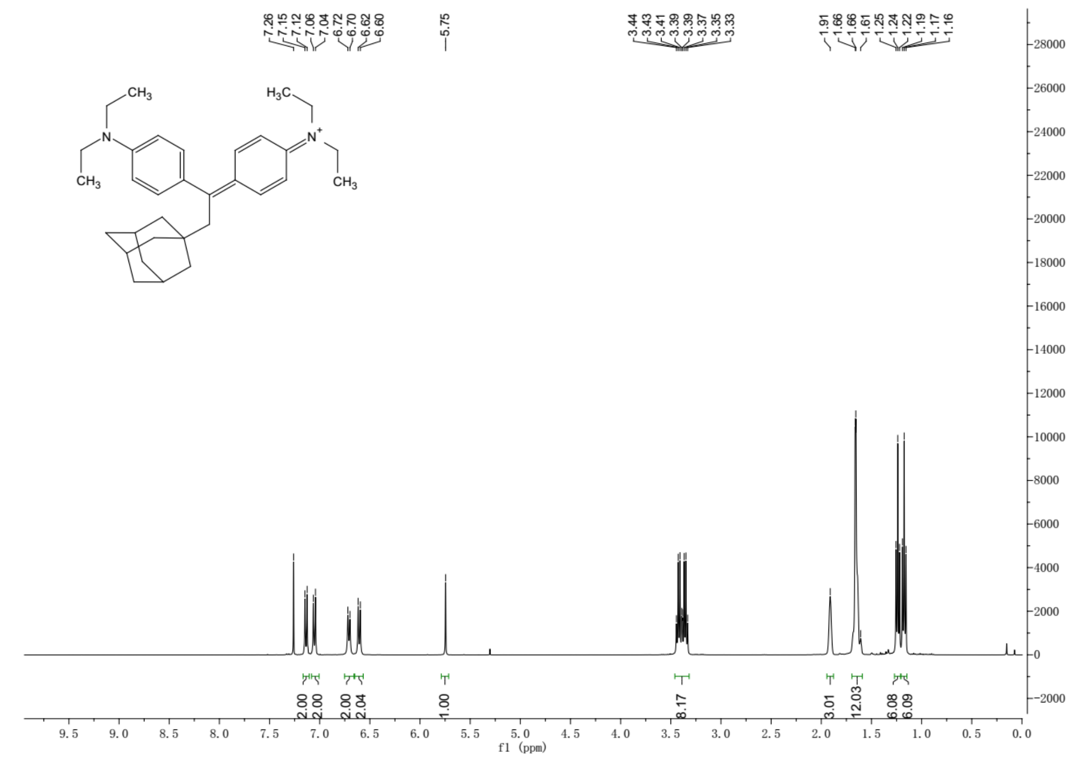

Supplementary Figure 377. The ^{1}H-NMR of compound **C1** in $CDCl_3$

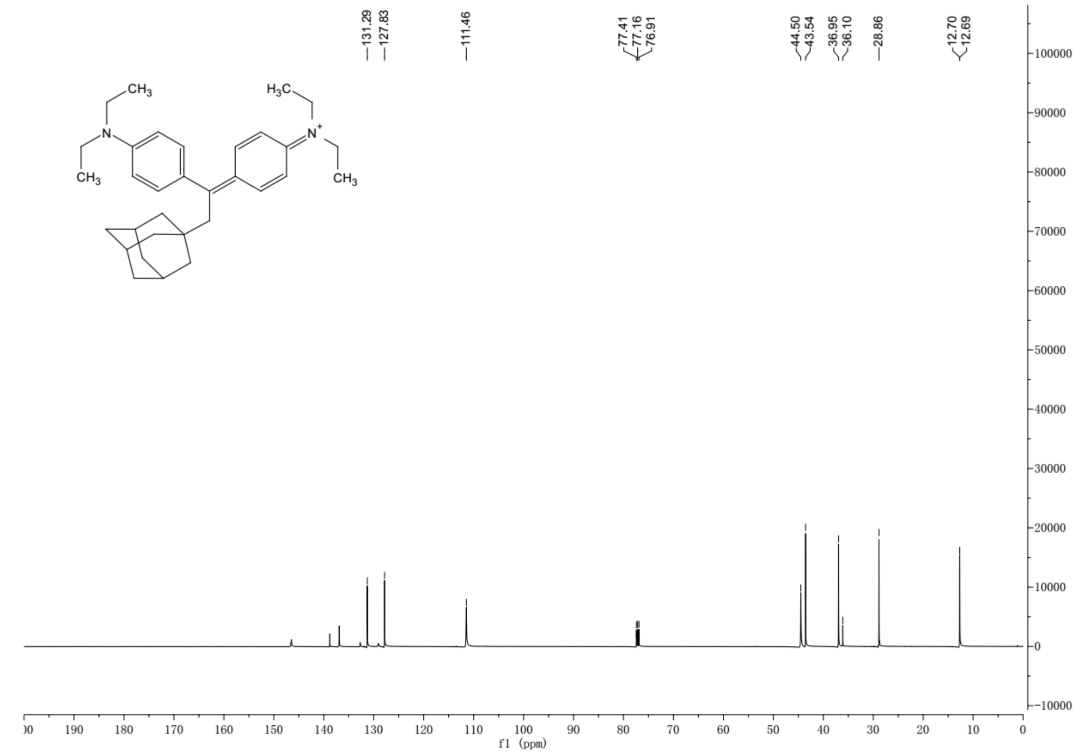

Supplementary Figure 378. The ^{13}C-NMR of compound **C1** in $CDCl_3$

Single Mass Analysis
Tolerance = 5.0 mDa / DBE: min = -1.5, max = 50.0
Element prediction: Off
Number of isotope peaks used for i-FIT = 2

Monoisotopic Mass, Even Electron Ions
1 formula(e) evaluated with 1 results within limits (up to 50 best isotopic matches for each mass)
Elements Used:
C: 0-32 H: 0-45 N: 0-2
XH-QIAN
YYJ-LX-3 197 (2.251) Cm (196:197)

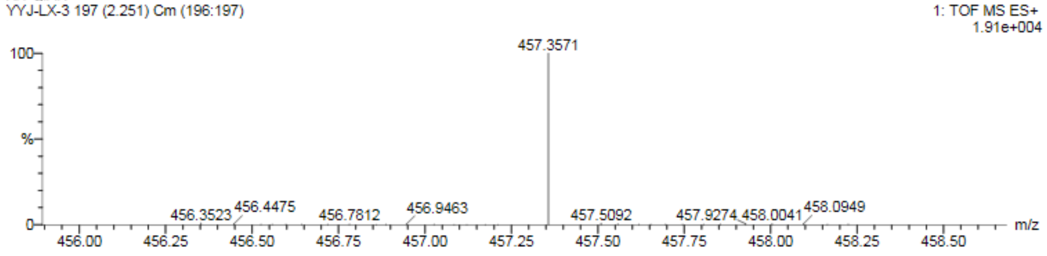

Minimum:				-1.5			
Maximum:		5.0	30.0	50.0			
Mass	Calc. Mass	mDa	PPM	DBE	i-FIT	i-FIT (Norm)	Formula
457.3571	457.3583	-1.2	-2.6	11.5	128.5	0.0	C32 H45 N2

Supplementary Figure 379. The HR-MS of compound **C1**

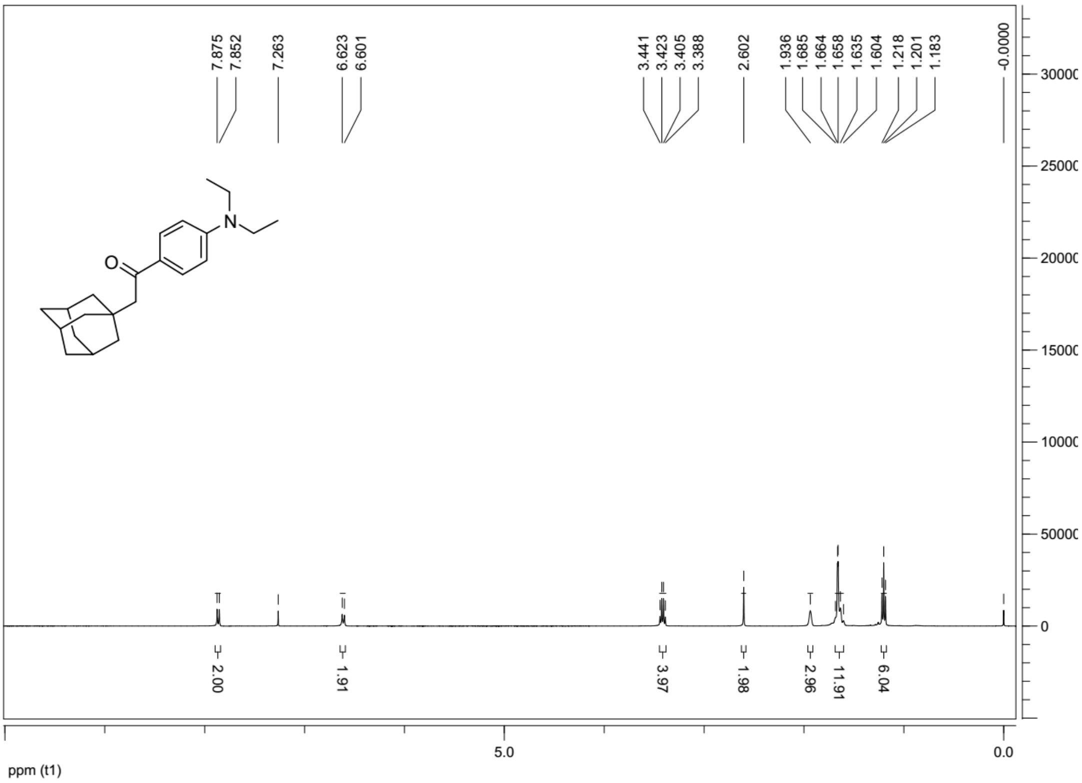

Supplementary Figure 380. The ^1H-NMR of compound **C2** in $CDCl_3$

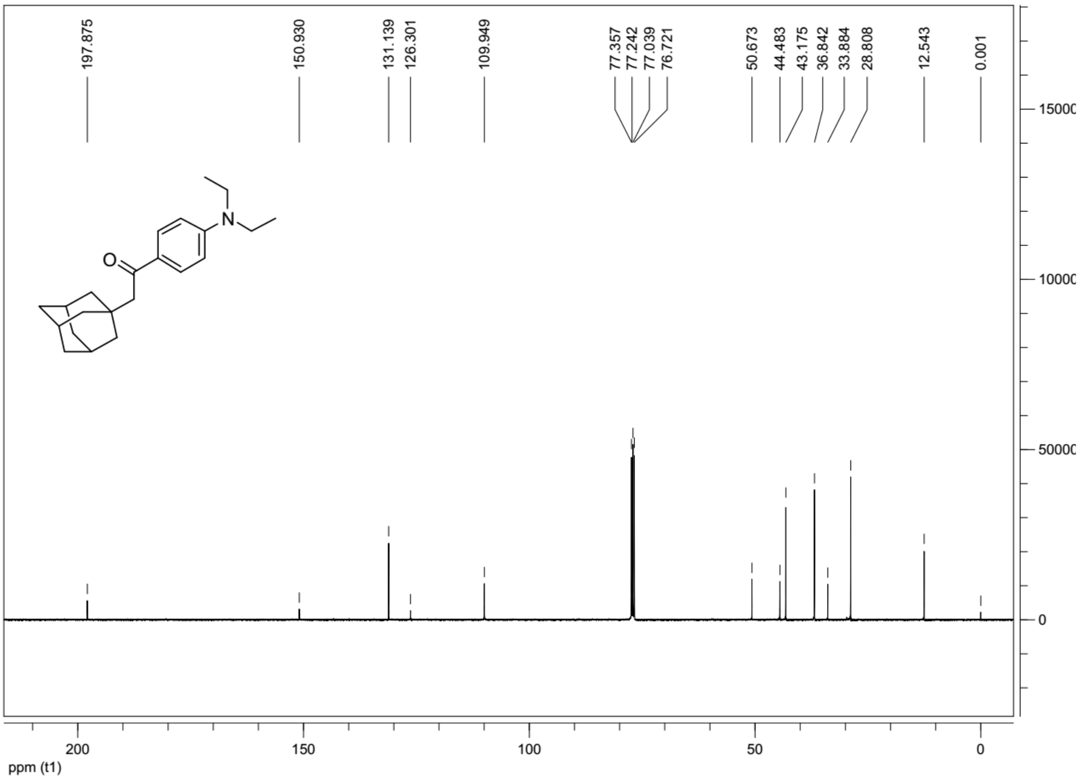

Supplementary Figure 381. The ^{13}C-NMR of compound **C2** in $CDCl_3$

Single Mass Analysis
Tolerance = 5.0 mDa / DBE: min = -1.5, max = 50.0
Element prediction: Off
Number of isotope peaks used for i-FIT = 2

Monoisotopic Mass, Even Electron Ions
2 formula(e) evaluated with 1 results within limits (up to 50 best isotopic matches for each mass)
Elements Used:
C: 0-22 H: 0-32 N: 0-1 O: 0-1
XH-QIAN
XYS-5 93 (1.052) Cm (92:95)

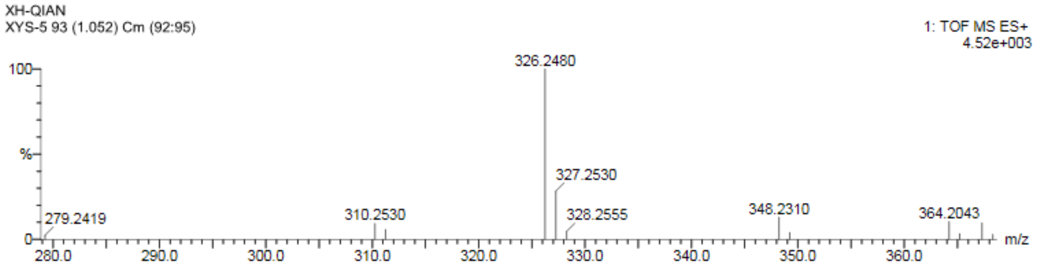

Minimum:				-1.5			
Maximum:		5.0	30.0	50.0			
Mass	Calc. Mass	mDa	PPM	DBE	i-FIT	i-FIT (Norm)	Formula
326.2480	326.2484	-0.4	-1.2	7.5	10.9	0.0	C22 H32 N O

Supplementary Figure 382. The HR-MS of compound **C2**

Supplementary Tables

Supplementary Table 1. Spectral properties of **RD** dyes in pH=7.4 PBS (50 mM) with 1% DMSO. (**RD23/24** were tested in ethanol). Φ_f was calculated with Rhodamine B as a reference (0.31 in H_2O and 0.49 in ethanol).

Dyes	λ_{abs} (nm)	λ_{abs} (nm)	Stokes shift (nm)	ε ($M^{-1}cm^{-1}$)	ϕ_f	Dyes	λ_{abs} (nm)	λ_{abs} (nm)	Stokes shift (nm)	ε ($M^{-1}cm^{-1}$)	ϕ_f
**RD1**	556	578	22	6.90E+04	0.219	**RD37**	577	599	22	3.60E+04	0.077
**RD2**	556	574	18	7.50E+04	0.190	**RD38**	573	599	26	6.10E+04	0.078
**RD3**	559	577	18	7.90E+04	0.195	**RD39**	560	593	23	2.70E+04	0.026
**RD5**	561	591	30	5.30E+04	0.154	**RD40**	585	N	N	2.27E+04	N
**RD6**	554	577	23	4.40E+04	0.310	**RD41**	569	603	34	5.90E+04	0.005
**RD7**	565	588	23	5.88E+04	0.185	**RD42**	563	591	28	6.40E+04	0.008
**RD8**	565	587	22	6.40E+04	0.234	**RD43**	559	577	18	1.30E+04	0.045
**RD9**	564	585	21	6.20E+04	0.155	**RD44**	559	578	19	3.50E+04	0.025
**RD10**	559	581	22	8.00E+04	0.212	**RD45**	561	576	15	2.70E+04	0.042
**RD11**	560	587	27	4.40E+04	0.090	**RD46**	557	579	22	3.90E+04	0.012
**RD12**	563	586	23	6.10E+04	0.173	**RD47**	556	588	32	5.40E+04	0.009
**RD13**	566	590	24	5.60E+04	0.157	**RD48**	545	561	16	4.90E+04	0.228
**RD14**	561	581	20	7.90E+04	0.011	**RD49**	547	564	17	8.70E+04	0.263
**RD15**	567	590	23	6.40E+04	0.004	**RD50**	549	565	16	6.80E+04	0.281
**RD16**	562	583	21	7.70E+04	0.181	**RD51**	551	571	20	8.30E+04	0.039
**RD17**	566	591	25	6.10E+04	0.045	**RD52**	550	566	16	2.30E+04	0.017
**RD18**	563	588	25	8.70E+04	0.223	**RD53**	547	586	39	6.00E+04	0.369
**RD19**	566	583	17	3.00E+03	0.121	**RD54**	562	587	25	7.70E+04	0.207
**RD20**	569	583	14	1.40E+04	0.088	**RD55**	554	570	16	6.40E+04	0.327
**RD21**	564	593	29	4.80E+04	0.005	**RD56**	555	590	35	2.50E+04	0.350
**RD22**	562	593	31	5.70E+04	0.006	**RD57**	559	577	18	7.80E+04	0.253
**RD23**	558	586	28	1.37E+05	0.087	**RD58**	567	591	24	1.00E+03	0.377
**RD24**	558	585	27	2.20E+05	0.140	**RD59**	553	595	42	8.60E+04	0.164
**RD25**	558	580	22	7.60E+04	0.121	**RD60**	567	584	17	5.20E+04	0.117
**RD26**	563	587	24	7.00E+04	0.261	**RD61**	580	602	22	6.10E+04	0.149
**RD27**	556	580	24	8.00E+04	0.006	**RD62**	572	582	10	2.20E+04	0.016
**RD28**	556	579	23	6.60E+04	0.026	**RD63**	572	592	20	6.40E+04	0.150
**RD29**	565	591	25	7.90E+04	0.125	**RD64**	584	606	22	6.10E+04	0.079
**RD30**	561	587	26	7.50E+04	0.144	**RD65**	574	592	18	4.40E+04	0.092
**RD31**	583	617	34	4.60E+04	0.018	**RD66**	569	590	21	7.00E+04	0.100
**RD32**	572	594	22	5.30E+04	0.014	**RD67**	586	604	18	5.90E+04	0.009
**RD33**	582	623	41	4.80E+04	0.032	**RD68**	568	596	28	3.00E+04	0.011
**RD34**	574	600	26	4.50E+04	0.074	**RD69**	556	575	19	5.20E+04	0.267
**RD35**	575	612	37	6.10E+04	0.084	**RD70**	654	668	14	6.20E+04	0.008
**RD36**	568	591	23	5.50E+04	0.117						

Supplementary Table 2. Antimicrobial activity of **RD1-70** against Gram-positive methicillin-resistant *Staphylococcus aureus* (MRSA, ATCC43300) and Gram-negative *Acinetobacter baumannii* (ATCC19606)

	ATCC43300	ATCC19606		ATCC43300	ATCC19606		ATCC43300	ATCC19606
**RD1**	1	>64	**RD25**	>64	>64	**RD49**	8	64
**RD2**	>64	>64	**RD26**	32	>64	**RD50**	>64	>64
**RD3**	>64	>64	**RD27**	4	16	**RD51**	32	>64
**RD4**	>64	>64	**RD28**	8	>64	**RD52**	>64	>64
**RD5**	8	>64	**RD29**	4	>64	**RD53**	1	4
**RD6**	>64	>64	**RD30**	2	32	**RD54**	>64	>64
**RD7**	>64	>64	**RD31**	1	32	**RD55**	8	>64
**RD8**	>64	>64	**RD32**	16	64	**RD56**	16	64
**RD9**	2	16	**RD33**	4	>64	**RD57**	32	>64
**RD10**	16	32	**RD34**	32	>64	**RD58**	>64	>64
**RD11**	4	64	**RD35**	>64	>64	**RD59**	>64	>64
**RD12**	4	8	**RD36**	2	>64	**RD60**	2	>64
**RD13**	32	>64	**RD37**	4	>64	**RD61**	4	>64
**RD14**	2	64	**RD38**	2	>64	**RD62**	32	>64
**RD15**	1	>64	**RD39**	32	>64	**RD63**	2	>64
**RD16**	16	16	**RD40**	>64	>64	**RD64**	8	>64
**RD17**	2	>64	**RD41**	8	>64	**RD65**	>64	64
**RD18**	8	>64	**RD42**	16	>64	**RD66**	>64	32
**RD19**	>64	>64	**RD43**	>64	>64	**RD67**	4	32
**RD20**	>64	>64	**RD44**	4	4	**RD68**	4	>64
**RD21**	2	64	**RD45**	1	4	**RD69**	2	16
**RD22**	1	4	**RD46**	1	>64	**RD70**	32	64
**RD23**	8	>64	**RD47**	2	>64	**Vancomycin**	2	
**RD24**	>64	>64	**RD48**	2	64	**Linezolid**	1	

Supplementary Table 3. Antimicrobial activity of **S1-S69** against Gram-positive methicillin-resistant *Staphylococcus aureus* (MRSA, ATCC43300) and Gram-negative *Acinetobacter baumannii* (ATCC19606)

	ATCC43300	ATCC19606		ATCC43300	ATCC19606		ATCC43300	ATCC19606
**S1**	>64	>64	**S25**	>64	>64	**S49**	>64	>64
**S2**	>64	>64	**S26**	>64	>64	**S50**	>64	>64
**S3**	>64	>64	**S27**	>64	>64	**S51**	>64	>64
**S4**	>64	>64	**S28**	>64	>64	**S52**	>64	>64
**S5**	>64	>64	**S29**	>64	>64	**S53**	>64	>64
**S6**	>64	>64	**S30**	>64	>64	**S54**	>64	>64
**S7**	>64	>64	**S31**	>64	>64	**S55**	>64	>64
**S8**	>64	>64	**S32**	>64	>64	**S56**	>64	>64
**S9**	>64	>64	**S33**	>64	>64	**S57**	>64	>64
**S10**	>64	>64	**S34**	>64	>64	**S58**	>64	>64
**S11**	>64	>64	**S35**	>64	>64	**S59**	>64	>64
**S12**	>64	>64	**S36**	>64	>64	**S60**	>64	>64
**S13**	>64	>64	**S37**	>64	>64	**S61**	>64	>64
**S14**	>64	>64	**S38**	>64	>64	**S62**	>64	>64
**S15**	>64	>64	**S39**	>64	>64	**S63**	>64	>64
**S16**	>64	>64	**S40**	>64	>64	**S64**	>64	>64
**S17**	>64	>64	**S41**	>64	>64	**S65**	>64	>64
**S18**	>64	>64	**S42**	>64	>64	**S66**	>64	>64
**S19**	>64	>64	**S43**	>64	>64	**S67**	>64	>64
**S20**	>64	>64	**S44**	>64	>64	**S68**	>64	>64
**S21**	>64	>64	**S45**	>64	>64	**S69**	>64	>64
**S22**	>64	>64	**S46**	>64	>64	**S70**	>64	>64
**S23**	>64	>64	**S47**	>64	>64			
**S24**	>64	>64	**S48**	>64	>64			

Supplementary Table 4. The abbreviations and strains of all the organisms used in this article.

Organism	Abbreviation	Strain
Staphylococcus aureus	*S.aureus*,MSSA	ATCC25923, CMCC26003
	S.aureus,MRSA	ATCC43300
Enterococcus faecalis	*E.faecalis*,VSE	ATCC29212
	E.faecalis,VRE	ATCC51299
Enterococcus faecium	*E.faecium*	ATCC35667
Staphylococcus epidermidis	*S.epidermidis*	CMCC26069
Streptococcus pyogenes	*S.pyogenes*	CMCC32006
Helicobacter pylori	*H.pylori*	Sydney Strain 1(SS1)
Acinetobacter baumannii	*A.baumannii*	ATCC19606
Pseudomonas aeruginosa	*P.aeruginosa*	ATCC27853
Escherichia coli	*E.coli*	ATCC25922
Shigella flexneri	*S.flexneri*	ATCC51081

Supplementary Table 5. MIC of reference antibiotics against different organisms.

Antibiotic	Organism	MIC (μg mL^{-1})
Vancomycin	ATCC43300	2
	ATCC29212	4
Linezolid	ATCC43300	1
	ATCC51299	2
	ATCC29212	2
Tigecycline	ATCC43300	2
	ATCC51299	1
	ATCC19606	2
Levofloxacin	ATCC43300	0.5
	ATCC25923	0.5
Ciprofloxacin	ATCC51299	1
	ATCC19606	1
	ATCC25922	0.03125
Daptomycin	ATCC51299	4
Polymyxin E	ATCC19606	1
Meropenem	ATCC19606	1
	ATCC25922	0.125
Ceftriaxone	ATCC25922	0.125
Tetracycline	ATCC25922	2
	ATCC25923	1
Erythromycin	ATCC29212	0.5
	ATCC25923	1
Gentamicin	ATCC25923	1
Clarithromycin	Sydney Strain 1(SS1)	0.0039
Amoxicillin	Sydney Strain 1(SS1)	0.25

Supplementary Table 6. Diversity Index of the library of our group and Chang's group.

Index	Yang's	Chang's
Diversity_NumAssemblies	0.66667	0.22222
Diversity_NumFPFeatures	18.875	3.2278
FP MinDistance	0	0.054795
FP MaxDistance	0.85443	0.86400
FP AvgDistance	0.62450	0.64432
Property MinDistance	0	0
Property MaxDistance	1.6679	1.1751
Property AverDistance	0.43709	0.46771
Diversity FractionCells	0.012000	0.016000

Supplementary Table 7. MIC of 30 individual colonies purified from 5th and 10th passage.

strain	antibiotic	passage	Colonies growable in Cx /tested colonies
A. baumannii	**RD22**	5th	29/30
		10th	4/30
	**RD53**	5th	23/30
		10th	3/30
	PmE	5th	30/30
		10th	30/30
	CIP	5th	30/30
		10th	30/30
	TGC	5th	30/30
		10th	30/30
E.coli	**RD53**	5th	30/30
		10th	5/30
	CRO	5th	30/30
		10th	30/30
	TCY	5th	25/30
		10th	29/30
	CIP	5th	30/30
		10th	30/30

Thirty individual colonies are randomly selected from heritable resistant bacteria (*Acinetobacter baumannii* and *Escherichia coli*) on MHA plates and the absolute fold change of MIC with respect to **RD22**, **RD53** and control drugs are tested. Before measuring MIC, each individual colony was inoculated to antibiotic-free broth for three times. The MIC's of control drugs to almost all individual colonies of heritable resistant bacteria were higher than C_x in Figure 3. In comparison, more colonies of **RD22** and **RD53** resistant bacteria could not grow in C_x. These results indicate that the two bacteria had lower tendency of resistance acquisition toward **RD22** and **RD53** than what Figure 3 in the manuscript has shown.

Supplementary Table 8. Spontaneous frequency of resistance experiments.

Strain	antibiotic	MIC($\mu g\ ml^{-1}$)	spontaneous frequency of resistance		
			2*MIC	4*MIC	8*MIC
ATCC43300	**RD53**	0.5	10^{-7}	10^{-8}	$<10^{-9}$
	**RD22**	0.25	10^{-6}	10^{-7}	10^{-8}
	VAN	2	$5*10^{-8}$	10^{-8}	10^{-9}
	LEV	0.25	10^{-5}	10^{-6}	10^{-7}
ATCC19606	**RD53**	8	$2*10^{-6}$	$<10^{-9}$	$<10^{-9}$
	**RD22**	8	$3*10^{-5}$	$1*10^{-5}$	$1*10^{-6}$
	PmE	1	10^{-4}	$2*10^{-5}$	10^{-6}
	TGC	2	10^{-8}	$<10^{-9}$	$<10^{-9}$
	CIP	1	10^{-5}	$2*10^{-6}$	$<10^{-9}$
ATCC51299	**RD53**	2	$<10^{-9}$	$<10^{-9}$	$<10^{-9}$
	**RD22**	2	$<10^{-9}$	$<10^{-9}$	$<10^{-9}$
	TGC	0.125	$<10^{-9}$	$<10^{-9}$	$<10^{-9}$
	CIP	0.5	$<10^{-9}$	$<10^{-9}$	$<10^{-9}$
	LNZ	2	$<10^{-9}$	$<10^{-9}$	$<10^{-9}$
ATCC29212	**RD53**	1	$<10^{-9}$	$<10^{-9}$	$<10^{-9}$
	**RD22**	1	$<10^{-9}$	$<10^{-9}$	$<10^{-9}$
	TGC	0.125	$<10^{-9}$	$<10^{-9}$	$<10^{-9}$
	CIP	1	$<10^{-9}$	$<10^{-9}$	$<10^{-9}$
	LNZ	2	$<10^{-9}$	$<10^{-9}$	$<10^{-9}$
ATCC25922	**RD53**	8	10^{-8}	10^{-9}	$<10^{-9}$
	**RD22**	32	$<10^{-9}$	$<10^{-9}$	$<10^{-9}$
	CIP	0.015625	10^{-7}	$<10^{-9}$	$<10^{-9}$
	CRO	0.0625	10^{-5}	10^{-6}	10^{-7}
	TCY	2	10^{-6}	10^{-7}	10^{-8}

10^9 bacteria cells per plate were plated onto MHA plates containing 2xMIC, 4xMIC, and 8xMIC **RD53**, **RD22** or control drugs. After 48 hours of incubation, the plates were examined for colonies. Spontaneous frequency = the number of colonies grown on the agar plates containing the compound/10^9. **RD22** and **RD53** were less prone to induce resistance than some control drugs.

Supplementary Table 9. Susceptibility of **RD53** resistant *A. baumannii* (**RD53**-Ab-20th) to five clinical antibacterial drugs.

MIC ($\mu g\ mL^{-1}$)	ATCC19606	**RD53**-Ab-20th
**RD53**	4	128
Tigecycline	1	0.5
Ciprofloxacin	1	0.5
Polymyxin E	1	1
Levofloxacin	1	0.25
Ampicillin-sulbactam	8	16

Supplementary Methods

General methods. Chemicals were purchased from major venders based in China and used without further purification. Analytical grade solvents were from Titan Scientific, China. THF was dried over sodium/benzoketyl still. The ^{1}H-NMR and ^{13}C-NMR spectra were collected on a Bruker AV-400 spectrometer. Chemicals shifts are referenced to the residue solvent peaks and given in parts per million (ppm). HRMS spectra were acquired on a Micromass GCT spectrometer. Absorption spectra were collected by a SHIMADZU UV-2600 UV-vis spectrophotometer. Fluorescence excitation and emission spectra were collected in a PTI-QM4 steady-stead fluorimeter with a 75 W Xenon arc-lamp and a model 810 PMT. The voltage of PMT was 950 V. The excitation and emission slits were set to 2 nm and all emission spectra were corrected.

Molar Absorptivity and fluorescence quantum yields determinations. The DMSO solution of dyes at 100 μM were prepared and used as a stock for further preparation of dilute dye solutions. The first dye solution for measurement was prepared by diluting 10.0 μL (with a 10 μL Hamilton® air-tight micro-syringe) of this dye stock into 0.99 mL pH=7.4 PBS. The absorption spectrum and the emission spectrum of the resulting solution (in a 4 mL Starna® micro-fluorescence cuvette) were acquired. Then, the dye solution was diluted by half using pH=7.4 PBS and the abs/em of the resulting solution were acquired. Then, diluted again by half and collected abs/em again. And so on until five sets of abs/em spectra with an absorbance below 0.1 were available. The abs/em spectra from these five sets were used to calculate the fluorescence quantum yields. The absorbance values were plotted against the dye concentration. The slope was calculated, which is the extinction coefficient of **RD** dyes. The fluorescence quantum yield of **RD** dyes in H_2O are calculated following a protocol provided by Jobin Yvin Ltd. at http://www.horiba.com/fileadmin/uploads/Scientific/Documents/Fluorescence/quantumyieldstrad.pdf. A fluorescence quantum yield of 0.31 for Rhodamine B in H_2O, reported in *J. Photoch. Photobio. 1999, 70, 737-744* was used to calculate the fluorescence quantum yields of all rhodamine dyes.

Colocalization cell imaging. HeLa human cervical carcinoma cells (Cell Bank of Chinese Academy of Science, cat. no. TCHu 187)/A549 (Cell Bank of Chinese Academy of Science, cat. no. TCHu150) cells were purchased from Cell Bank of Type Culture Collection of Chinese Academy of Science. The cells were cultured in Dlubecco's modified Eagle's medium (DMEM, Gibco) containing 10% (v/v) fetal bovine serum (FBS, Hyclone), 1% (v/v) penicillin (100 units/mL) and streptomycin (100 μg/mL). Cultures were maintained at 37 °C in humidified air containing 5 % CO_2. Before imaging, the cells were passed and plated on glass-bottomed dished (NEST).

HeLa/A549 cells were incubated with 1 μM RD dye and 0.1 μM MitoTracker Green FM/0.1 μM LysoTracker-Green DND-26 (Invitrogen)/0.1 μM ER-Tracker Blue-White DPX (Invitrogen) sequentially and washed with PBS for three times to remove excess dyes. Confocal images were recorded on a Leica TCS SP8 confocal microscope. Briefly, three lasers (405 nm for ER-Tracker Blue-White DPX, and 488 nm for MitoTracker Green FM and LysoTracker-Green DND-26, 561 nm for RD,) were sequentially applied for imaging to avoid emissive overlap between two dyes. Lasers were focused at the back focal plane of a 60x oil objective. The fluorescence emission was filtered with a DM 405/488/561 and further separate the two channels with SDM 560. The wavelength selection performed by a galvanometer diffraction grating is used as following: 410-480 nm for ER-Tracker

Blue-White DPX, 505-550 nm for MitoTracker Green FM and LysoTracker-Green DND-26, 565-680 nm for RD. The co-localization data was analyzed with the manufacturer's software.

Bacterial Cell Imaging. After incubating a 1-mL bacterial suspension at 108 cells with 0.5*MIC and 8*MIC **RD53** for 3min at 37 °C with aeration at 220 r.p.m., the resultant product was washed three times by centrifugation (5510 g, 3 min) to remove all the unbound **RD53**. The bacteria were then resuspended in PBS. Confocal images were recorded on a Leica TCS SP8 STED 3X confocal microscope with a 100x oil objective. Confocal images were collected with λ_{ex} = 561 nm and λ_{em} = 590-640 nm.

Mammalian cytotoxicity of RD53. Cytotoxicity was determined on normal human kidney 2 cells HK-2 cells human renal proximal tubular cells (Cell Bank of Chinese Academy of Science, cat. no. SCSP-511), HUVEC Primary Umbilical Vein Endothelial Cells (American type culture collection, cat. no. PCS-100-013) and HeLa human cervical carcinoma cells (Cell Bank of Chinese Academy of Science, cat. no. TCHu 187). Briefly, HK-2 and HeLa cells were cultured in RPMI-1640 medium supplemented with FBS, and HUVEC were cultured in DMEM, high glucose medium supplemented with FBS. When cells reached ~80% confluence, they were harvested and resuspended in the growth medium to ~2.0x10^5 cells mL^{-1}. 100 µL cells were inoculated into 96 well plate and incubated at 37 °C with 5% CO_2 for 4.5 h. Then, 0.25 µL of twofold serial compound dilutions in DMSO to 99.75 µL of media were added to the plate and incubated at 37 °C with 5% CO_2 for 43 hrs. For cell viability test, 20 µL 0.5% MTT solution were added to the plate and the supernatant was discarded after 4 h incubation. Then 150 µL DMSO were added to dissolve formazan and the A_{490nm} (OD_{490}) was measured. 50% cell cytotoxicity (CC_{50}) was analyzed in GraphPad Prism.

Synthesis and characterization.

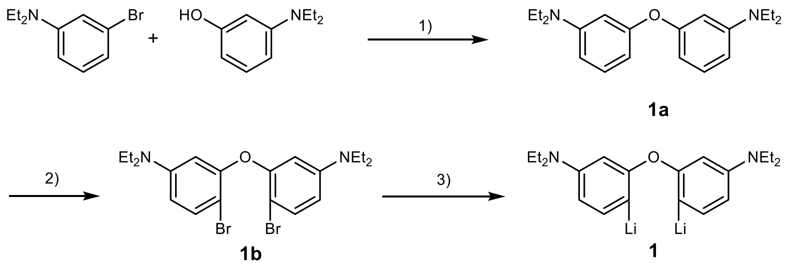

Supplementary Figure 383. Synthesis route to compound **1**. 1) Cs_2CO_3, CuBr, DMF, 140 °C; 2) Br_2, DCM, r. t.; 3) nBuLi/tBuLi, THF, -78 °C - r. t..

Synthesis of 3,3'-oxybis(N,N-diethylaniline) (1a) . To a solution of 3-bromo-N,N-diethylaniline (2 g, 1 equiv., 8.77 mmol) in dry DMF (40 ml) was added 3-(diethylamino)phenol (2.17 g, 1.5 equiv., 13.15 mmol), Cs_2CO_3 (4.28 g, 1.5 equiv., 13.15 mmol), and CuBr (126 mg, 0.1 equiv., 0.88 mmol). The reaction mixture was thoroughly deoxygenated by bubbling Ar for 15 minutes, heated to 140 °C with rigorous stirring for 20 h before cooled to room temperature. A saturated solution of NH_4Cl (50 mL) was added and the resulting mixture was extracted repeatedly with CH_2Cl_2. The organic layer was combined, dried with $MgSO_4$ and filtered. Both CH_2Cl_2 and DMF was removed under reduced pressure to yield a viscous residue, which was purified by a flash column using a mixture of petroleum ether and EtOAc [100:1, v/v] as an eluent to afford **1a** (2.25 g) as a transparent liquid in an 82% yield. ^{1}H NMR (400 MHz, $CDCl_3$): δ 7.12 (t, *J* = 8.3 Hz, 2H), 6.42-6.40 (m, 4H), 6.27 (dd, *J* = 8.3 Hz, 2.0 Hz, 2H), 3.33 (q, *J* = 7.0 Hz, 8H), 1.15 (t, *J* = 7.0 Hz, 12H). ^{13}C NMR (101 MHz, $CDCl_3$) δ 158.7, 149.4, 130.0, 106.8, 105.6, 102.7, 44.5, 12.7. EI-HRMS (m/z): $[M+H]^+$ calcd. for $C_{20}H_{28}N_2O$,

312.2202; found 312.2201

Synthesis of 3,3'-oxybis(4-bromo-N,N-diethylaniline) (1b). A solution of dry bromine (0.66ml, 2 equiv., 12.80 mmol) in CH_2Cl_2 (20 ml) was added with vigorous stirring to a solution of compound **1a** (2 g, 1 equiv., 6.40 mmol) in the same solvent (20 ml). A saturated solution of $NaHCO_3$ (50 mL) was added after an hour and the resulting mixture was extracted repeatedly with CH_2Cl_2. The organic layer was combined, dried with $MgSO_4$ and filtered. CH_2Cl_2 was removed under reduced pressure to yield a white solid (**1b**, 3.01 g) in a quantitative yield. ^{1}H NMR (400 MHz, $CDCl_3$): δ 7.35 (d, *J* = 8.9 Hz, 2H), 6.33 (dd, *J* = 8.9 Hz, 2.8 Hz, 2H), 6.16 (d, *J* = 2.8 Hz, 2H), 3.23 (q, *J* = 7.0 Hz, 8H), 1.07 (t, *J* = 7.0 Hz, 12H). ^{13}C NMR (101 MHz, $CDCl_3$) δ 154.1, 148.5, 133.6, 108.9, 103.5, 98.5, 44.7, 12.5. EI-HRMS (m/z): $[M+H]^+$ calcd. for $C_{20}H_{27}Br_2N_2O$, 471.0470; found 471.0456

Synthesis of dilithium reagent 1. A solution of n-BuLi (2 equiv, 2.5 M in hexane) was syringed into a solution of compound **1b** (1 equiv) in anhydrous THF at −78 °C dropwise, and the resulting mixture **1** was stirred at −78 °C for another 30 minutes prior to use in the next step (general procedure B). For the preparation of **RD11**, the dilithium reagent **1** was prepared by adding t-BuLi (4 equiv, 1.6 M in hexane) into a solution of compound **1b** (1 equiv) in anhydrous THF.

S1, S2, S3, S4, S6, S7, S8, S9, S12, S14, S15, S18, S20, S23, S26, S28, S29, S30, S31, S32, S33, S34, S35, S36, S37, S38, S40, S41, S43, S46, S47, S48, S49, S50, S54, S55, S56, S57, S59, S60, S61, S63, S64, S65, S67, S68, S69, S70 were obtained from commercial sources and used without further purification.

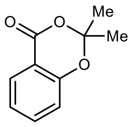

2,2-dimethyl-4H-benzo[d][1,3]dioxin-4-one (S10[1]). S10 was prepared using the reported procedure, and the spectral data are in good agreement with the literature. Colorless liquid. ^{1}H NMR (400 MHz, $CDCl_3$): δ 7.94 (d, *J* = 7.8 Hz, 1H), 7.53 (t, *J* = 7.4 Hz, 1H), 7.10 (t, *J* = 7.5 Hz, 1H), 6.95 (d, *J* = 8.2 Hz, 1H), 1.72 (s, 6H).

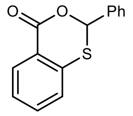

2-phenyl-4H-benzo[d][1,3]oxathiin-4-one (S11[2]). **S11** was prepared using the reported procedure, and the spectral data are in good agreement with the literature. White solid. ^{1}H NMR (400 MHz, $CDCl_3$): δ 8.23 (d, *J* = 8.0 Hz, 1H), 7.60 (d, *J* = 4.2 Hz, 2H), 7.52 (t, *J* = 7.4 Hz, 1H), 7.44-7.42 (m, 3H), 7.37-7.33 (m, 2H), 6.57 (s, 1H).

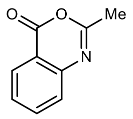

2-methyl-4H-benzo[d][1,3]oxazin-4-one (S13[3]). **S13** was prepared using the reported procedure, and the spectral data are in good agreement with the literature. Colorless liquid. ^{1}H NMR (400 MHz, $CDCl_3$): δ 8.18 (d, *J* = 7.4 Hz, 1H), 7.79 (td, *J* = 7.2 Hz, 1.1 Hz, 1H), 7.54 (d, *J* = 8.0 Hz, 1H), 7.50 (d, *J* = 7.4 Hz, 1H), 2.47 (s, 3H).

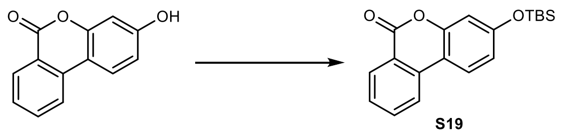

Supplementary Figure 384. Synthesis route to compound **S19**. Reaction condition: TBSCl, DMAP, imidazole, DMF, r. t..

Synthesis of 3-((tert-butyldimethylsilyl)oxy)-6H-benzo[c]chromen-6-one (S19). To a solution of *tert*-butylchlorodimethysilane (710.28 mg, 1 equiv, 4.71 mmol) in DMF (20 mL) was added 3-hydroxy-6H-benzo[c]chromen-6-one (1g, 1 equiv, 4.71 mmol), imidazole (481.22 mg, 1.5 equiv, 7,07 mmol), and 4-dimethylaminopyridine (28.79 mg, 0.05 equiv, 0.24 mmol) in separate portions with stirring. After being stirred for 2h at ambient temperature, the reaction mixture was poured into water and the suspension was extracted repeatedly with CH_2Cl_2. The organic layer was combined, dried with $MgSO_4$ and filtered. CH_2Cl_2 was removed under reduced pressure to yield a white solid (1.49 g) in a 97% yield. ^{1}H NMR (400 MHz, $CDCl_3$): δ 8.34 (d, *J* = 7.9Hz, 1H), 7.99 (d, *J* = 7.3 Hz, 1H), 7.90(d, *J* = 9.4 Hz, 1H), 7.77(t, *J* = 7.7 Hz, 1H), 7.50 (t, *J* = 7.6 Hz, 1H), 6.85-6.83 (m, *2*H), 1.00(s, 9H), 0.26(s, 6H) ; ^{13}C NMR (101 MHz, $CDCl_3$) δ 161.6, 158.0, 152.5, 135.3, 135.0, 130.7, 127.9, 123.8, 121.3, 120.2, 117.6, 112.0, 108.6, 25.7, 18.4, 4.3. ESI-MS (m/z): $[M+Na]^+$ calcd. for $C_{19}H_{22}NaO_3Si$, 349.1236; found 349.1231.

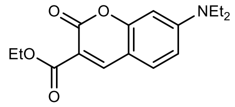

ethyl 7-(diethylamino)-2-oxo-2H-chromene-3-carboxylate (S42[4]). **S42** was prepared using the reported procedure, and the spectral data are in good agreement with the literature. Yellow liquid. ^{1}H NMR (400 MHz, $CDCl_3$): δ 8.42 (s, 1H), 7.35 (d, *J* = 8.9 Hz, 1H), 6.59 (dd, *J* = 8.9 Hz, 2.4 Hz, 1H), 6.46 (d, *J* =2.4 Hz, 1H), 4.36 (q, *J* = 7.0 Hz, 2H), 3.44 (q, *J* = 7.1 Hz, 4H), 1.38 (t, *J* = 7.1 Hz, 3H), 1.22 (t, *J* = 7.1 Hz, 6H).

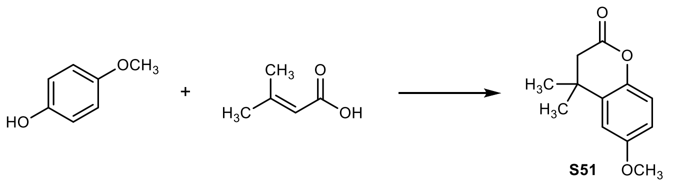

Supplementary Figure 385. Synthesis route to compound **S51**. Reaction condition: $MeSO_3H$, 68 °C.

6-methoxy-4,4-dimethylchroman-2-one (S51[5]). 4-methoxyphenol (1 g, 1 equiv., 8.06 mmol), 3-methylbut-2-enoic acid (887.14 mg, 1.1 equiv., 8.86 mmol) were dissolved in 10 ml of methylsulphonic acid, and the mixture was heated to 68°C for 6 hours before poured into 100 ml ice water. A saturated solution of $NaHCO_3$ (50 mL) was added and the resulting mixture was extracted repeatedly with CH_2Cl_2. The organic layer was combined, dried with $MgSO_4$ and filtered. CH_2Cl_2 was removed under reduced pressure to yield a viscous residue, which was purified by a flash column using a mixture of petroleum ether and EtOAc [15:1, v/v] as an eluent to afford **S51** (1.42 g) as a transparent liquid in an 85% yield. ^{1}H NMR (400 MHz, $CDCl_3$): δ 6.98 (d, *J* = 8.8 Hz, 1H), 6.82 (d, *J* = 2.9 Hz, 1H), 6.76 (dd, *J* = 8.8 Hz, 2.9 Hz, 1H), 3.80 (s, 3H), 2.59 (s, *2*H), 1.33 (s, 6H).

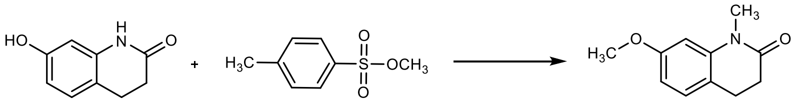

S52

Supplementary Figure 386. Synthesis route to compound **S52**. Reaction condition: NaH, MeCN, reflux.

7-methoxy-1-methyl-3,4-dihydroquinolin-2(1H)-one (S52[6]). 7-hydroxy-3,4-dihydroquinolin-2(1H)-one (1 g, 1 equiv., 6.13 mmol), methyl 4-methylbenzenesulfonate (2.51 g, 2.2 equiv., 13.48 mmol), NaH (323.50 mg, 2.2 equiv, 13.48 mmol) were dissolved in 50 ml of MeCN, and the mixture was heated to reflux for 18 hours. The resulting mixture was extracted repeatedly with CH_2Cl_2. The organic layer was combined, dried with $MgSO_4$ and filtered. CH_2Cl_2 was removed under reduced pressure to yield a white residue, which was recrystallized using a mixture of petroleum ether and EtOAc to afford **S52** (1.02 g) as a white crystal in an 86% yield. ^{1}H NMR (400 MHz, $CDCl_3$): δ 7.07 (d, *J* = 7.9 Hz, 1H), 6.56 (s, 1H), 3.82 (s, 3H), 3.34 (s, 3H), 2.84 (t, *J* = 7.8 Hz, 2H), 2.63 (t, *J* = 7.8 Hz, 2H), 3.80 (s, 3H), 2.59 (s, *2*H), 1.33 (s, 6H).

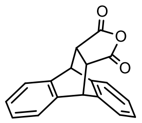

(9R,10S,11S,15R)-9,10,11,15-tetrahydro-9,10-[3,4]furanoanthracene-12,14-dione (S58[7]). S58 was prepared using the reported procedure, and the spectral data are in good agreement with the literature. White solid. ^{1}H NMR (400 MHz, $CDCl_3$): δ 7.41-7.38 (m, 2H), 7.35-7.33 (m, 2H), 7.22-7.19 (m, 4H), 4.83 (s, 2H), 3.52 (s, 2H).

General procedure A for the preparation of S16, S17, S21, S22, S24, S25, S27, S39, S44, S45, S53, S62, S66.

A solution of the corresponding acid in MeOH was added 0.1 mL concentrate H_2SO_4. The mixture was heated under reflux for 8 h before cooled down to room temperature. MeOH was removed under reduced pressure and the residue was extracted repeatedly with CH_2Cl_2. The organic layer was combined, dried with $MgSO_4$ and filtered. CH_2Cl_2 was removed under reduced pressure to yield a product in a virtually quantitative yield.

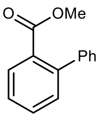

Methyl [1,1'-biphenyl]-2-carboxylate (S16[8]). Prepared according to general procedure A using [1,1'-biphenyl]-2-carboxylic acid to give a transparent liquid. ^{1}H NMR (400 MHz, $CDCl_3$): δ 7.84 (d, *J* = 7.7 Hz, 1H), 7.54 (t, *J* = 7.5 Hz, 1H), 7.44-7.33 (m, 7H), 3.65 (s, 3H).

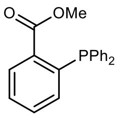

Methyl 2-(diphenylphosphino)benzoate (S17[9]). Prepared according to general procedure A using 2-(diphenylphosphanyl)benzoic acid to give a white solid. ^{1}H NMR (400 MHz, $CDCl_3$): δ 8.07-8.04 (m, 1H), 7.40-7.37 (m, 2H), 7.33-7.27 (m, 10H), 6.95-6.91 (m, 1H), 3.74 (s, 3H).

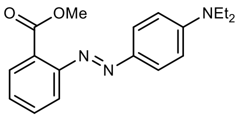

(E)-methyl 2-((4-(diethylamino)phenyl)diazenyl)benzoate (S21). Prepared according to general procedure A using (*E*)-2-((4-(diethylamino)phenyl)diazenyl)benzoic acid to give a red liquid. ^{1}H NMR (400 MHz, $CDCl_3$): δ 7.84 (d, *J* = 9.1 Hz, 2H), 7.23 (dd, *J* = 7.6 Hz, 1.0 Hz, 1H), 7.62 (d, *J* = 7.4 Hz, 1H), 7.53 (t, *J* = 6.8 Hz, 1H), 7.37 (t, *J* = 6.8 Hz, 1H), 6.71 (d, *J* = 9.1 Hz, 2H), 3.89 (s, 3H), 3.46 (q, *J* = 7.1 Hz, 4H), 1.23 (t, J = 7.1 Hz, 6H); 13C NMR (101 MHz, CDCl3) δ169.0, 152.6, 150.6, 143.4, 131.8, 129.6, 128.2, 128.0, 125.9, 119.5, 111.1, 52.4, 44.9, 12.8. ESI-HRMS (m/z): $[M+1]^+$ calcd. for $C_{18}H_{22}N_3O_2^+$, 312.1712; found 312.1712

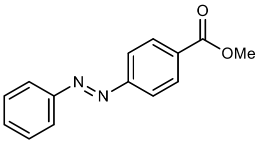

(E)-methyl 4-(phenyldiazenyl)benzoate (S22[10]). Prepared according to general procedure A using (E)-4-(phenyldiazenyl)benzoic acid to give a yellow solid. ^{1}H NMR (400 MHz, $CDCl_3$): δ 8.19 (d, *J* = 8.6 Hz, 2H), 7.95 (d, *J* = 8.6 Hz, 4H), 7.56-7.49 (m, 3H), 3.95 (s, 3H).

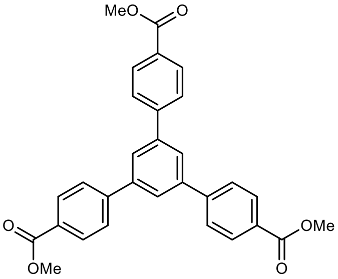

Dimethyl 5'-(4-(methoxycarbonyl)phenyl)-[1,1':3',1''-terphenyl]-4,4''-dicarboxylate (S24[11]). Prepared according to general procedure A using 5'-(4-carboxyphenyl)- [1,1':3',1''-terphenyl]-4,4''-dicarboxylic acid to give a white solid.^{1}H NMR (400 MHz, $CDCl_3$): δ 8.17 (d, *J* = 8.4 Hz, 6H), 7.87 (s, 3H), 7.77 (d, *J* = 8.4 Hz, 6H), 3.97 (s, 9H).

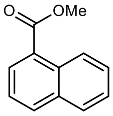

Methyl 1-naphthoate (S25[12]). Prepared according to general procedure A using 1-naphthoic acid to give a colorless liquid. ^{1}H NMR (400 MHz, $CDCl_3$): δ 8.95 (d, *J* = 8.7 Hz, 1H), 8.20 (dd, *J* = 7.3 Hz, 1.0 Hz, 1H), 8.02 (d, *J* = 8.2 Hz, 1H), 7.89 (d, *J* = 8.2 Hz, 1H), 7.63 (t, *J* = 8.2 Hz, 1H), 7.54 (t, *J* = 8.3 Hz, 1H), 7.50 (t, *J* = 8.2 Hz, 1H), 4.01 (s, 3H).

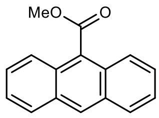

Methyl anthracene-9-carboxylate (S27[13]). Prepared according to general procedure A using anthracene-9-carboxylic acid to give a light yellow solid. ^{1}H NMR (400 MHz, $CDCl_3$): δ 8.54 (s, 1H), 8.03 (d, *J* = 9.6 Hz, 4H), 7.57-7.48 (m, 4H), 4.19 (s, 3H).

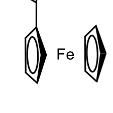

Methyl ferrocenecarboxylate (S39[14]). Prepared according to general procedure A using ferrocenecarboxylic acid to give a yellow solid. ^{1}H NMR (400 MHz, $CDCl_3$): δ 4.80 (s, 2H), 4.40 (s, 2H), 4.21 (s, 5H), 3.81 (s, 3H).

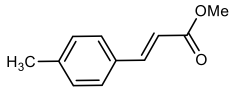

Methyl (*E*)-3-(p-tolyl)acrylate (S44[15]). Prepared according to general procedure A using (*E*)-3-(p-tolyl)acrylic acid to give a white solid. ^{1}H NMR (400 MHz, $CDCl_3$): δ 7.67 (d, *J* = 12 Hz, 1H), 7.42 (d, *J* = 8.1 Hz, 2H), 7.19 (d, *J* = 8.0 Hz, 2H), 6.40 (d, *J* = 12 Hz, 1H), 3.80 (s, 3H), 2.38 (s, 3H).

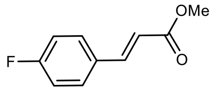

Methyl (*E*)-3-(4-fluorophenyl)acrylate (S45[16]). Prepared according to general procedure A using (*E*)-3-(4-fluorophenyl)acrylic acid to give a white solid. ^{1}H NMR (400 MHz, $CDCl_3$): δ 7.66 (d, *J* = 16 Hz, 1H), 7.53-7.50 (m, 2H), 7.10-7.06 (m, 2H), 6.37 (d, *J* = 16 Hz, 1H), 3.81 (s, 3H).

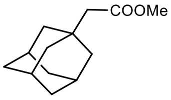

Methyl 2-((3r,5r,7r)-adamantan-1-yl)acetate (S53[17]). Prepared according to general procedure A using 2-(adamantan-1-yl)acetic acid to give a colorless liquid. ^{1}H NMR (400 MHz, $CDCl_3$): δ 3.63 (s, 3H), 2.06 (s, 2H), 1.95 (s, 3H), 1.70-1.59 (m, 12H).

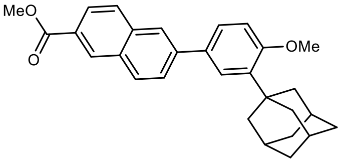

Methyl 6-(3-((3r,5r,7r)-adamantan-1-yl)-4-methoxyphenyl)-2-naphthoate (S62[19]). Prepared according to general procedure A using 6-(3-((3r,5r,7r)-adamantan-1-yl)-4-methoxyphenyl) -2-naphthoic acid to give a white solid. ^{1}H NMR (400 MHz, $CDCl_3$): δ 8.61 (s, 1H), 8.07 (dd, *J* = 8.7 Hz, 1.5 Hz, 1H), 8.01 (s, 1H), 8.00 (d, *J* = 8.7 Hz, 1H), 7.92 (d, *J* = 8.6 Hz, 1H), 7.80 (dd, *J* = 8.5 Hz, 1.6 Hz, 1H), 7.60 (d, *J* = 2.2 Hz, 1H), 7.55 (dd, *J* = 8.4 Hz, 2.3 Hz, 1H), 7.01 (d, *J* = 8.5 Hz, 1H), 3.99 (s, 3H), 3.91 (s, 3H), 2.18 (s, 6H), 2.10 (s, 3H), 1.80 (s, 6H).

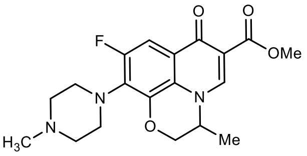

Methyl 9-fluoro-3-methyl-7-oxo-10-(piperidin-1-yl)-3,7-dihydro-2H-[1,4]oxazino[2,3,4-ij] quinoline -6-carboxylate (S66[18]). Prepared according to general procedure A using 9-fluoro-3-methyl-10-(4-methylpiperazin-1-yl)-7-oxo-2,3-dihydro-7H-[1,4]oxazino[2,3,4-ij]quinoline-6-carboxylic acid to give a yellow solid. ^{1}H NMR (400 MHz, $CDCl_3$): δ 8.62 (s, 1H), 7.69 (d, *J* = 12.2 Hz, 1H), 4.56-4.51 (m, 1H), 4.45 (dd, *J* = 11.6 Hz, 2.0 Hz, 1H), 4.36 (dd, *J* = 11.4 Hz, 2.0 Hz, 1H), 3.45-3.34 (m, 4H), 2.54 (s, 4H), 2.36 (s, 3H), 1.61 (d, *J* = 6.8 Hz, 3H).

General procedures B for the preparation of rhodamine RD1-70 from S1-70.

A solution of dilithium reagent **1** (1.3 equiv) (2.6 equiv for **S23, S41** and 3.9 equiv for **S24**) in anhydrous THF was slowly added to a solution of compound **S1-70** (100 mg, 1 equiv) in anhydrous THF (20 mL) at −78 °C via syringe. The reaction mixture was allowed to warm to room temperature and stirred for another 2h. Saturated NH_4Cl solution (20 mL) was poured into the reaction flask, and the reaction mixture was stirred for another 2h before extracted with CH_2Cl_2 repeatedly (3X). The combined organic layer was dried with anhydrous $CaCl_2$ powder, filtered, and concentrated under reduced pressure to give a viscous residue, exchanged with a suitable anion exchange resin from which compounds **RD1-70** were obtained from a flash column over silica with a mixture of CH_2Cl_2 and MeOH.

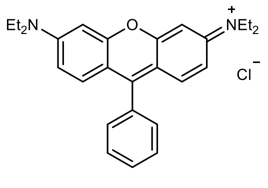

N-(6-(diethylamino)-9-phenyl-3H-xanthen-3-ylidene)-N-ethylethanaminium chloride salt (RD1[20]). Compound **RD1** was prepared from **S1** (100 mg, 1 equiv) and dilithium reagent **1** (1.3 equiv) according to general procedure B. Violet solid (268 mg) was obtained in an 85% yield by flash chromatography (CH_2Cl_2/MeOH = 12:1, v/v). ^1H NMR (400 MHz, $CDCl_3$): δ 7.63-7.62 (m, 3H), 7.38-7.34 (m, 4H), 6.94 (dd, J = 8.0 Hz, 1.6Hz, 2H), 6.87 (d, J = 1.6 Hz, 1H), 3.67 (q, J = 7.0 Hz, 8H), 1.34 (t, J = 7.0 Hz, 12H).

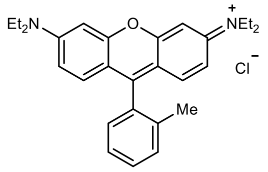

N-(6-(diethylamino)-9-(o-tolyl)-3H-xanthen-3-ylidene)-N-ethylethanaminium chloride salt (RD2[20]). Compound **RD2** was prepared from **S2** (100 mg, 1 equiv) and dilithium reagent **1** (1.3 equiv) according to general procedure B. Violet solid (240 mg) was obtained in an 80% yield by flash chromatography (CH_2Cl_2/MeOH = 12:1, v/v). ^1H NMR (400 MHz, $CDCl_3$) δ 7.45 (t, J = 7.7 Hz, 1H), 7.38−7.32 (m, 2H), 7.12−7.07 (m, 3H), 6.90 (dd, J = 9.5, 2.2 Hz, 2H), 6.80 (d, J = 2.2 Hz, 2H), 3.62 (q, J = 7.1 Hz, 8H), 2.0 (s, 3H), 1.29 (t, J = 7.1 Hz, 12H).

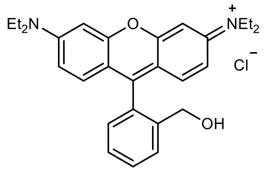

N-(6-(diethylamino)-9-(2-(hydroxymethyl)phenyl)-3H-xanthen-3-ylidene)-N-ethylethanaminium chloride salt (**RD3[21]**). Compound **RD3** was prepared from **S3** (100 mg, 1 equiv) and dilithium reagent **1** (1.3 equiv) according to general procedure B. Violet solid (279 mg) in an 81% yield by flash chromatography (CH_2Cl_2/MeOH = 10:1, v/v). ^1H NMR (400 MHz, $CDCl_3$): δ 7.94 (d, *J* = 7.3 Hz, 1H), 7.56 (t, *J* = 7.3 Hz, 1H), 7.38 (t, *J* = 7.3 Hz, 1H), 7.25 (d, *J* = 9.4 Hz, 2H), 7.05 (d, *J* = 7.3 Hz, 1H), 6.84 (dd, *J* = 9.4Hz, 1.0 Hz, 2H), 6.73 (d, *J* = 1.0 Hz, 2H), 4.43 (s, 2H), 3.59 (q, *J* = 7.0 Hz, 8H), 1.31(t, *J* = 7.0 Hz, 12H).

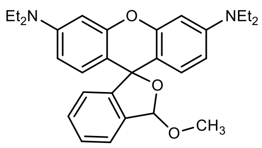

N3',N3',N6',N6'-tetraethyl-3-methoxy-3H-spiro[isobenzofuran-1,9'-xanthene]-3',6'-diamine (**RD4**). Compound **RD4** was prepared from **S4** (100 mg, 1 equiv) and dilithium reagent **1** (1.3 equiv) according to general procedure B. Pink solid (235 mg) was obtained in an 84% yield by flash chromatography (CH_2Cl_2/MeOH = 30:1, v/v). ^1H NMR (400 MHz, $CDCl_3$): δ 7.49 (d, *J* = 7.4 Hz, 1H), 7.37 (t, *J* = 7.3 Hz, 1H), 7.33 (t, *J* = 7.3 Hz, 1H), 7.01 (d, *J* = 6.4 Hz, 1H), 6.93 (d, *J* = 7.5 Hz, 1H), 6.68 (d, *J* = 8.8 Hz, 1H), 6.42-6.35 (m, 4H), 6.27 (s, 1H), 3.53 (s, 3H), 3.38-3.32 (m, 8H), 1.19-1.14 (m, 12H).

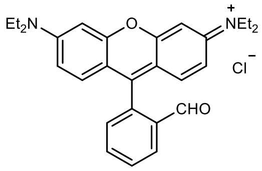

N-(6-(diethylamino)-9-(2-formylphenyl)-3H-xanthen-3-ylidene)-N-ethylethanaminium chloride salt (RD5) Compound **RD5** was prepared from **S5** (100 mg, 1 equiv) and dilithium reagent **1** (1.3 equiv) according to general procedure B. Violet solid (210 mg) was obtained in a 75% yield by flash chromatography (CH_2Cl_2/MeOH = 10:1, v/v). ^1H NMR (400 MHz, $CDCl_3$): δ 9.78 (s, 1H), 8.13 (d, *J* = 6.6 Hz, 1H), 7.81-7.78 (m, 2H), 7.29 (d, *J* = 6.2 Hz, 1H), 6.95 (d, *J* = 8.6 Hz, 2H), 6.85 (d, *J* = 8.4 Hz, 2H), 6.73 (s, 2H), 3.57 (q, *J* = 8.0 Hz, 8H), 1.23 (t, *J* = 7.8 Hz, 12H);^{13}C NMR (101 MHz, $CDCl_3$) δ190.3, 157.5, 155.9, 155.5, 134.5, 134.5, 132.8, 132.6, 131.1, 131.0, 130.4, 114.5, 113.7, 96.3 , 46.2, 12.6. ESI-HRMS (m/z): $[M]^+$ calcd. for $C_{28}H_{31}N_2O_2$, 427.2386; found 427.2383.

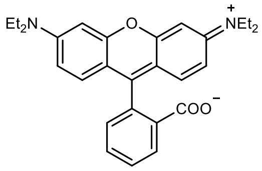

2-(6-(diethylamino)-3-(diethyliminio)-3H-xanthen-9-yl)benzoate (**RD6**). Compound **RD6** was prepared from **S6** (100 mg, 1 equiv) and dilithium reagent **1** (1.3 equiv) according to general procedure B. Violet solid (268 mg) was obtained in an 89% yield by flash chromatography (CH_2Cl_2/MeOH = 10:1, v/v). ^1H NMR (400 MHz, $CDCl_3$): δ 8.05 (d, *J* = 7.4 Hz, 1H), 7.59 (t, *J* = 7.4 Hz, 1H), 7.53 (d, *J* = 7.4 Hz, 1H), 7.15 (d, *J* = 7.4 Hz, 1H), 6.70 (d, *J* = 8.7 Hz, 2H), 6.49 (s, 2H), 6.42 (d, *J* = 8.7 Hz, 2H), 3.38 (q, *J* = 6.9 Hz, 8H), 1.17 (t, *J* = 6.9 Hz, 12H).

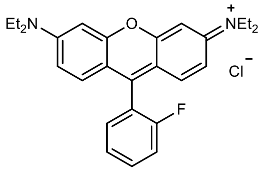

3-(diethyl-l4-azanylidene)-N,N-diethyl-9-(2-fluorophenyl)-3H-xanthen-6-amine chloride salt (**RD7**). Compound **RD7** was prepared from **S7** (100 mg, 1 equiv) and dilithium reagent **1** (1.3 equiv) according to general procedure B. Violet solid (165 mg) was obtained in a 57% yield by flash chromatography (CH_2Cl_2/MeOH = 12:1, v/v). ^1H NMR (400 MHz, $CDCl_3$): δ 7.66-7.61 (m, 1H), 7.42

(t, *J* = 7.6 Hz, 1H), 7.34-7.30 (m, 2H), 7.26 (dd, *J* = 9.5 Hz, 1.2 Hz, 2H), 6.94 (dd, *J* = 9.6 Hz, 1.2 Hz, 2H),, 6.82 (d, *J* = 1.2 Hz, 2H), 3.65 (q, *J* = 7.2 Hz, 8H), 1.32 (t, *J* = 7.1 Hz, 12H); ^{13}C NMR (101 MHz, $CDCl_3$) δ160.4, 158.0, 155.8, 151.3, 132.9, 132.8, 131.7, 131.3, 125.1, 125.0, 119.6, 119.4, 116.7, 116.5, 114.6, 113.5, 96. 6, 46.3, 12.7. ESI-MS (m/z): $[M]^+$ calcd. for $C_{27}H_{30}FN_2O$, 417.2342; found 417.2343.

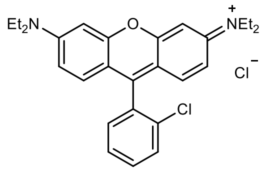

9-(2-chlorophenyl)-3-(diethyl-l4-azanylidene)-N,N-diethyl-3H-xanthen-6-amine chloride salt (RD8). Compound **RD8** was prepared from **S8** (100 mg, 1 equiv) and dilithium reagent **1** (1.3 equiv) according to general procedure B. Violet solid (155 mg) was obtained in a 61% yield by flash chromatography (CH_2Cl_2/MeOH = 12:1, v/v). ^{1}H NMR (400 MHz, $CDCl_3$): δ 7.62 (dd, *J* = 8.0 Hz, 1.5 Hz, 1H), 7.58 (td, *J* = 7.2 Hz, 1.8 Hz, 1H), 7.53 (td, *J* = 7.4 Hz, 1.6 Hz, 1H), 7.30 (dd, *J* = 6.9 Hz, 1.6 Hz, 1H), 7.14 (d, *J* = 9.5 Hz, 2H), 6.95 (dd, *J* = 9.5 Hz, 2.5 Hz, 2H), 6.86 (d, *J* = 2.4 Hz, 2H), 3.66 (q, *J* = 7.2 Hz, 8H), 1.33 (t, *J* = 7.1 Hz, 12H); ^{13}C NMR (101 MHz, $CDCl_3$) δ158.1, 155.9, 154.2, 132.9, 131.8, 131.6, 131.1, 130.9, 130.4, 127.6, 114.6, 113.4, 96.7, 46.4, 12.8; ESI-MS (m/z): $[M]^+$ calcd. for $C_{27}H_{30}ClN_2O$, 433.2047; found 433.2046.

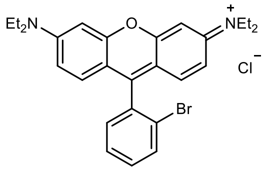

N-(9-(2-bromophenyl)-6-(diethylamino)-3H-xanthen-3-ylidene)-N-ethylethanaminium chloride salt (RD9) Compound **RD9** was prepared from **S9** (100 mg, 1 equiv) and dilithium reagent **1** (1.3 equiv) according to general procedure B. Violet solid (148 mg) was obtained in a 68% yield by flash chromatography (CH_2Cl_2/MeOH = 12:1, v/v). ^{1}H NMR (400 MHz, $CDCl_3$): δ 7.79 (d, *J* = 7.9 Hz, 1H), 7.57 (t, *J* = 7.0 Hz, 1H), 7.49 (t, *J* = 7.2 Hz, 1H), 7.27 (d, *J* = 7.9 Hz, 1H), 7.11 (d, *J* = 9.1 Hz, 2H), 6.94 (d, *J* = 8.9 Hz, 2H), 6.84 (s, 2H), 3.65 (q, *J* = 7.2 Hz, 8H), 1.32 (t, *J* = 7.0 Hz, 12H); 13C NMR (101 MHz, $CDCl_3$) δ158.1, 155.8, 155.5, 133.6, 133.2, 131.8, 131.6, 130.8, 128.1, 122.2, 114.7, 113.3, 96.8, 46.5, 12.8; ESI-MS (m/z): $[M]^+$ calcd. for $C_{27}H_{30}BrN_2O$, 477.1542; found 477.1548

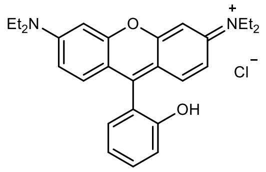

N-(6-(diethylamino)-9-(2-hydroxyphenyl)-3H-xanthen-3-ylidene)-N-ethylethanaminium chloride salt (RD10[22]). Compound **RD10** was prepared from **S10** (100 mg, 1 equiv) and dilithium reagent **1** (1.3 equiv) according to general procedure B. Violet solid (181 mg) was obtained in a 72% yield by flash chromatography (CH_2Cl_2/MeOH = 10:1, v/v). ^{1}H NMR (400 MHz, CD_3OD): δ 7.48 (t, *J* = 7.2 Hz, 1H), 7.37 (d, *J* = 9.5 Hz, 2H), 7.19 (dd, *J* = 7.3 Hz, 1.4 Hz, 1H), 7.11-7.08 (m, 2H), 7.05 (dd, *J* = 9.6 Hz, 2.1 Hz, 2H), 6. 93 (d, *J* =2.1 Hz, 2H), 3.66 (q, *J* = 7.0 Hz, 8H), 1.30 (t, *J* = 7.0 Hz, 12H).

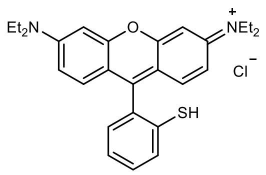

N-(6-(diethylamino)-9-(2-mercaptophenyl)-3H-xanthen-3-ylidene)-N-ethylethanaminium chloride salt (RD11[23]). Compound **RD11** was prepared from **S11** (100 mg, 1 equiv) and dilithium reagent **1** (1.3 equiv) according to general procedure B. Violet solid (165 mg) was obtained in a 70% yield by flash chromatography (CH_2Cl_2/MeOH = 12:1, v/v). ^{1}H NMR (400 MHz, CD_3OD): δ 7.80 (t, *J* = 8.0 Hz, 1H), 7.62 (t, *J* = 7.7 Hz, 1H), 7.49 (t, *J* = 7.7 Hz, 1H), 7.20 (d, *J* = 7.4 Hz, 1H), 7.08 (d, *J* = 9.5 Hz, 2H), 6. 87 (dd, *J* =9.5 Hz, 1.8 Hz, 2H), 6.76 (d, *J* = 1.8 Hz, 2H), 4.79 (s, 1H), 3.60 (q, *J* = 7.4 Hz, 8H), 1.27 (t, *J* = 7.0 Hz, 12H). ^{13}C NMR (101 MHz, $CDCl_3$) δ 157.9, 155.7, 155.0, 133.3, 132.8, 131.7, 131.2, 130.5, 130.0, 128.1, 114.5, 113.6, 96.5, 46.3, 12.7.

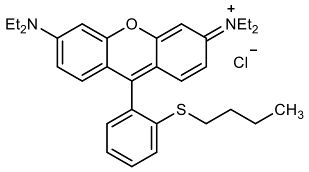

N-(9-(2-(butylthio)phenyl)-6-(diethylamino)-3H-xanthen-3-ylidene)-N-ethylethanaminium chloride salt (RD12). Compound **RD12** was prepared from **S12** (100 mg, 1 equiv) and dilithium reagent **1** (1.3 equiv) according to general procedure B. Violet solid (179 mg) was obtained in an 83% yield by flash chromatography (CH_2Cl_2/MeOH = 12:1, v/v). ^{1}H NMR (400 MHz, $CDCl_3$): δ 7.57-7.49 (m, 2H), 7.36 (t, *J* = 7.1 Hz, 1H), 7.14-7.12 (m, 3H), 6.83 (d, *J* = 9.6 Hz, 2H), 6. 78 (s, 2H), 3.59 (q, *J* = 7.4 Hz, 8H), 2.81 (t, *J* = 7.4 Hz, 2H), 1.49-1.41 (m, 2H), 1.30 (t, *J* = 7.2 Hz, 12H), 1.28-1.21 (m, 2H), 0.79 (t, *J* = 7.3 Hz, 3H); 13C NMR (101 MHz, $CDCl_3$): δ158.0, 156.6, 155.7, 136.7, 131.8, 131.6, 130.7, 129.5, 128.1, 125.8, 114.1, 113.6, 96.5, 46.2, 33.1, 30.8, 21.9, 13.6, 12.6; ESI-MS (m/z): $[M]^+$ calcd. for $C_{31}H_{39}N_2OS$, 487.2783; found 487.2782.

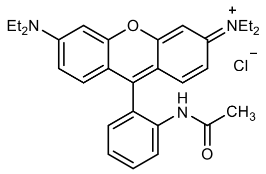

N-(9-(2-acetamidophenyl)-6-(diethylamino)-3H-xanthen-3-ylidene)-N-ethylethanaminium chloride salt (RD13). Compound **RD13** was prepared from **S13** (100 mg, 1 equiv) and dilithium reagent **1** (1.3 equiv) according to general procedure B. Violet solid (238 mg) was obtained in a 78% yield by flash chromatography (CH_2Cl_2/MeOH = 12:1, v/v). ^{1}H NMR (400 MHz, $CDCl_3$): δ 10.85 (s, 1H), 7.86 (d, J = 8.0 Hz, 1H), 7.56 (d, J = 8.0 Hz, 1H), 7.52 (d, J = 9.4 Hz, 2H), 7.30 (t, J = 7.5 Hz, 1H), 7.14 (d, J = 7.5 Hz, 1H), 6.88 (d, J = 8.2 Hz, 2H), 6.70 (s, 2H), 3.59-3.52 (m, 8H), 1.93 (s, 3H), 1.28 (t, J = 7.0 Hz, 12H); ^{13}C NMR (101 MHz, CDCl3): δ170.8, 158.4, 158.1, 155.4, 137.0, 133.3, 131.1, 130.2, 127.5, 127.0, 125.1, 113.9, 113.5, 96.0, 45.9, 29.8, 23.4, 12.7; ESI-MS (m/z): [M]+ calcd. for $C_{29}H_{34}N_3O_2$, 456.2651; found 456.2653.

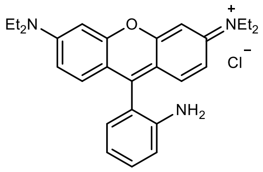

N-(9-(2-aminophenyl)-6-(diethylamino)-3H-xanthen-3-ylidene)-N-ethylethanaminium chloride salt (RD14[24]) Compound **RD14** was prepared from **RD14** (100 mg, 1 equiv) and dilithium reagent **1** (1.3 equiv) according to general procedure B. The product was then hydrolyzed with 3M HCl. Violet solid (210 mg) was obtained in a 76% yield by flash chromatography (CH_2Cl_2/MeOH = 10:1, v/v). ^1H NMR (400 MHz, $CDCl_3$): δ 7.42 (d, *J* = 9.5 Hz, 2H), 7.29 (t, *J* = 7.2 Hz, 1H), 7.05 (d, *J* = 8.2 Hz, 1H), 6.91 (dd, *J* = 7.5 Hz, 0.8 Hz 1H), 6.85 (dd, *J* = 9.5 Hz, 1.8 Hz, 2H), 6.81 (t, *J* = 7.4 Hz, 1H), 6.76 (d, *J* = 1.8 Hz, 2H), 3.60 (q, *J* = 7.0 Hz, 8H), 1.28 (t, *J* = 7.0 Hz, 12H);

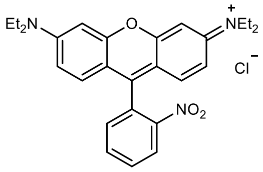

N-(6-(diethylamino)-9-(2-nitrophenyl)-3H-xanthen-3-ylidene)-N-ethylethanaminium chloride salt (RD15) Compound **RD15** was prepared from **S15** (100 mg, 1 equiv) and dilithium reagent **1** (1.3 equiv) according to general procedure B. Violet solid (182 mg) was obtained in a 69% yield by flash chromatography (CH_2Cl_2/MeOH = 12:1, v/v). ^1H NMR (400 MHz, $CDCl_3$): δ 8.36 (d, *J* = 8.2 Hz, 1H), 8.01 (t, *J* = 7.4 Hz, 1H), 7.86 (t, *J* = 7.4 Hz, 1H), 7.46 (d, *J* = 7.4 Hz, 1H), 7.01 (d, *J* = 9.5 Hz, 2H), 6.92 (dd, *J* = 9.5 Hz, 1.7 Hz, 2H), 6.77 (d, *J* = 1.7 Hz, 2H), 3.61 (q, *J* = 7.0 Hz, 8H), 1.28 (t, J = 7.0 Hz, 12H); 13C NMR (101 MHz, $CDCl_3$) δ157.8, 155.7, 154.2, 147.8, 135.1, 131.9, 131.7, 130.6, 129.0, 128.2, 127.5, 125.5, 114.9, 112.8, 96.5, 46.3, 12.7. ESI-HRMS (m/z): $[M+1]^+$ calcd. for $C_{27}H_{30}N_3O_3$, 444.2287; found 444.2285.

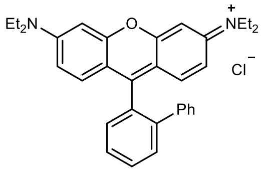

N-(9-([1,1'-biphenyl]-2-yl)-6-(diethylamino)-3H-xanthen-3-ylidene)-N-ethylethanaminium chloride salt (RD16) Compound **RD16** was prepared from **S16** (100 mg, 1 equiv) and dilithium reagent **1** (1.3 equiv) according to general procedure B. Violet solid (166 mg) was obtained in a 68% yield by flash chromatography (CH_2Cl_2/MeOH = 12:1, v/v). ^1H NMR (400 MHz, $CDCl_3$): δ 7.69 (t, *J* = 7.6 Hz, 1H), 7.61 (d, *J* = 7.4 Hz, 1H), 7.57 (t, *J* = 7.6 Hz, 1H), 7.29 (d, *J* = 7.5 Hz, 1H), 7.26 (d, *J* = 9.5 Hz, 2H), 7.14-7.13 (m, 3H), 7.07-7.06 (m, 2H), 6.85 (dd, *J* = 9.5Hz, 2.2 Hz, 2H), 6.76 (d, *J* = 2.2 Hz, 2H), 3.62 (q, *J* = 7.1 Hz, 8H), 1.31 (t, *J* = 7.1 Hz, 12H); ^{13}C NMR (101 MHz, $CDCl_3$) δ157.8, 157.8, 155.6, 141.9, 139.6, 132.1, 131.0, 130.8, 130.6, 130.4, 128.6, 128.5, 127.9, 127.8, 114.2, 113.8, 96.6, 46.3, 12.8.; ESI-MS (m/z): $[M]^+$ calcd. for $C_{33}H_{35}N_2O$, 475.2749; found 475.2750

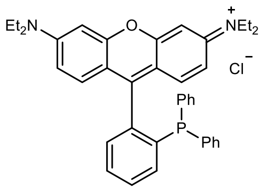

N-(6-(diethylamino)-9-(2-(diphenylphosphino)phenyl)-3H-xanthen-3-ylidene)-N-ethylethanaminium chloride salt (RD17). Compound **RD17** was prepared from **S17** (100 mg, 1 equiv) and dilithium reagent **1** (1.3 equiv) according to general procedure B. Violet solid (126 mg) was obtained in a 65% yield by flash chromatography (CH_2Cl_2/MeOH = 12:1, v/v). ^{1}H NMR (400 MHz, $CDCl_3$): δ 7.59 (t, *J* = 5.0 Hz, 2H), 7.39 (d, *J* = 6.6 Hz, 1H), 7.30-7.26 (m, 7H), 7.11 (t, *J* = 7.3 Hz, 4H), 6.88 (d, *J* = 9.4 Hz, 2H), 6.79 (s, 2H), 6.61 (d, *J* = 9.1 Hz, 2H), 3.64 (q, *J* = 6.4 Hz, 8H), 1.33 (t, J = 6.5 Hz, 12H); 13C NMR (101 MHz, $CDCl_3$) δ 157.7, 157.6, 157.5, 155.5, 138.2, 137.9, 137.8, 137.7, 135.3, 135.2, 134.5, 134.0, 133.8, 131.9, 130.3, 129.7, 129.3, 129.3, 129.2, 128.8, 128.7, 114.2, 114.2, 113.7, 96.4, 46.3, 12.8. ESI-HRMS (m/z): $[M]^+$ calcd. for $C_{39}H_{40}N_2OP$, 583.2878; found 583.2878

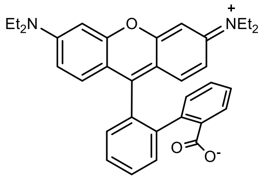

2'-(6-(diethylamino)-3-(diethyliminio)-3H-xanthen-9-yl)-[1,1'-biphenyl]-2-carboxylate (RD18) Compound **(RD18)** was prepared from **S18** (100 mg, 1 equiv) and dilithium reagent **1** (1.3 equiv) according to general procedure B. Violet solid (161 mg) was obtained in a 70% yield by flash chromatography (CH_2Cl_2/MeOH = 10:1, v/v). ^{1}H NMR (400 MHz, $CDCl_3$): δ 8.68 (s, 1H), 7.83 (d, *J* = 7.7 Hz, 1H), 7.74 (d, *J* = 7.5 Hz, 1H), 7.53 (s, 1H), 7.46 (t, *J* = 7.5 Hz, 1H), 7.33 (t, *J* = 7.4 Hz, 1H), 7.13 (d, *J* = 7.5 Hz, 1H), 6.96 (t, *J* = 7.3 Hz, 1H), 6.87 (d, *J* = 9.5 Hz, 2H), 6.71 (t, *J* = 7.4 Hz, 1H), 6.55 (s, 1H), 6.54 (d, *J* = 7.5 Hz, 2H), 3.62-3.46 (m, 8H), 1.38-1.06 (m, 12H); ^{13}C NMR (101 MHz, CDCl3) δ 173.3, 159.9, 157.7, 154.9, 143.4, 142.3, 137.2, 132.8, 130.3, 130.1, 129.9, 129.7, 128.8, 127.1, 126.4, 126.2, 114.4, 95.5, 45.8, 12.7. ESI-HRMS (m/z): $[M]^+$ calcd. for $C_{34}H_{35}N_2O_3$, 519.2648; found 519.2651.

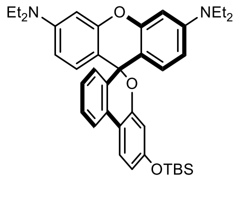

3-((tert-butyldimethylsilyl)oxy)-N3',N3',N6',N6'-tetraethylspiro[benzo[c]chromene-6,9'-xanthene]-3',6'-diamine (RD19) Compound **RD19** was prepared from **S19** (100 mg, 1 equiv) and dilithium reagent **1** (1.3 equiv) according to general procedure B. White solid (130 mg) was obtained in a 68% yield by flash chromatography (CH_2Cl_2/MeOH = 15:1, v/v). ^{1}H NMR (400 MHz, $CDCl_3$): δ 7.77 (d, *J* = 7.9 Hz, 1H), 7.67 (d, *J* = 8.5 Hz, 1H), 7.29 (t, *J* = 7.5Hz, 1H), 7.08 (t, *J* = 7.5Hz, 1H), 7.02 (d, *J* = 8.8 Hz, 2H), 6.87 (d, *J* = 7.7 Hz, 1H), 6.51 (dd, *J* = 8.5Hz, 2.2 Hz, 1H), 6.41 (d, *J* = 2.4 Hz, 2H), 6.30-6.27 (m, 3H), 3.33 (q, *J* = 7.0 Hz, 8H), 1.15 (t, *J* = 7.0 Hz, 12H), 0.93 (s, 9H), 0.14 (s, 6H); ^{13}C NMR (101 MHz, $CDCl_3$) δ 157.2, 153.9, 151.8, 148.9, 137.4, 130.7, 130.2, 129.3, 127.6, 126.9, 123.3, 120.4, 115.6, 113.6, 111.8, 110.0, 107.7, 97.5, 44.5, 25.8, 18.3, 12.8, -4.3. ESI-MS (m/z):

$[M+H]^+$ calcd. for $C_{39}H_{49}N_2O_3Si$, 621.3512; found 621.3515.

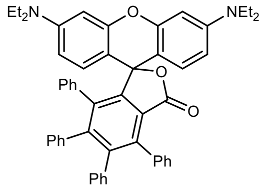

3',6'-bis(diethylamino)-4,5,6,7-tetraphenyl-3*H*-spiro[isobenzofuran-1,9'-xanthen]-3-one (RD20) Compound **(RD20)** was prepared from **S20** (100 mg, 1 equiv) and dilithium reagent **1** (1.3 equiv) according to general procedure B. Pink solid (113 mg) was obtained in a 68% yield by flash chromatography (CH_2Cl_2/MeOH = 15:1, v/v). ^1H NMR (400 MHz, $CDCl_3$): δ 7.27-7.20 (m,4H), 6.93 (d, *J* = 8.8 Hz, 2H), 6.87-6.82 (m, 7H), 6.74-6.73 (m, 3H), 6.94-6.68 (m, 2H), 6.63 (t, *J* = 7.6 Hz, 2H), 6.44 (dd, *J* = 8.8 Hz, 1.8 Hz, 2H), 6.05 (d, *J* = 7.3 Hz, 2H), 6.03 (d, *J* = 1.8 Hz, 2H), 3.36-3.29 (m, 8H), 1.15 (t, J = 7.0 Hz, 12H); ^{13}C NMR (101 MHz, $CDCl_3$) δ 169.1, 152.6, 152.4, 149.0, 148.2, 143.0, 139.4, 138.8, 138.7, 137.6, 136.0, 135.7, 131.4, 130.6, 130.6, 129.5, 128.8, 127.3, 127.1, 126.9, 126.6, 126.3, 125.9, 125.9, 122.0, 108.2, 106.3, 98.0, 44.7, 12.6. ESI-HRMS (m/z): $[M+H]^+$ calcd. for $C_{52}H_{47}N_2O_3$, 747.3587; found 747.3588

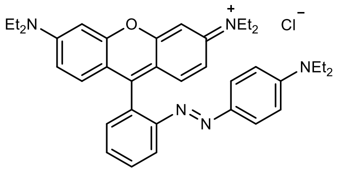

(E)-N-(6-(diethylamino)-9-(2-((4-(diethylamino)phenyl)diazenyl)phenyl)-3H-xanthen-3-ylidene)-N-ethylethanaminium chloride salt (RD21) Compound **(RD21)** was prepared from **S21** (100 mg, 1 equiv) and dilithium reagent **1** (1.3 equiv) according to general procedure B. Violet solid (149 mg) was obtained as a in a 76% yield by flash chromatography (CH_2Cl_2/MeOH = 12:1, v/v).. ^1H NMR (400 MHz, $CDCl_3$): δ 7.98 (d, *J* = 8.1 Hz, 1H), 7.68 (t, *J* = 7.6 Hz, 1H), 7.55 (t, *J* = 7.3 Hz, 1H), 7.35 (d, *J* = 9.1 Hz, 2H), 7.31 (d, *J* = 7.4 Hz, 1H), 7.23 (d, *J* = 9.5 Hz, 2H), 6.88 (d, *J* = 2.2 Hz, 2H), 6.77 (dd, *J* = 9.5 Hz, 2.2 Hz, 2H), 6.53 (d, *J* = 9.2 Hz, 2H), 3.61 (q, *J* = 7.0 Hz, 8H), 3.36 (q, *J* = 7.1 Hz, 4H), 1.30 (t, J = 7.0 Hz, 12H), 1.14 (t, J = 7.1 Hz, 6H); ^{13}C NMR (101 MHz, $CDCl_3$) δ 158.4, 157.8, 155.5, 151.2, 150.8, 142.8, 132.4, 131.2, 130.1, 129.7, 129.0, 125.9, 117.9, 114.3, 113.9, 110.9, 96.5, 46.2, 44.8, 12.8, 12.7. ESI-HRMS (m/z): $[M]^+$ calcd. for $C_{37}H_{44}N_5O$, 574.3546; found 574.3549

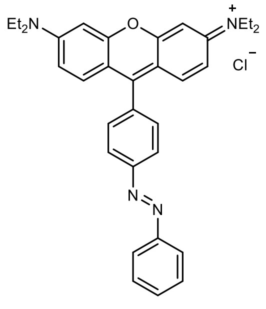

(E)-N-(6-(diethylamino)-9-(4-(phenyldiazenyl)phenyl)-3H-xanthen-3-ylidene)-N-ethylethanaminium chloride salt (RD22) Compound **RD22** was prepared from **S21** (100 mg, 1 equiv) and dilithium reagent **1** (1.3 equiv) according to general procedure B. Violet solid (176 mg) was obtained in a 79% yield by flash chromatography (CH_2Cl_2/MeOH = 12:1, v/v).. ^1H NMR (400

MHz, $CDCl_3$): δ 8.14 (d, *J* = 8.3 Hz, 2H), 7.99 (dd, *J* = 8.1 Hz, 1.8 Hz, 2H), 7.59-7.52 (m, 5H), 7.39 (d, *J* = 9.5 Hz, 2H), 6.95 (dd, *J* = 9.6 Hz, 2.2 Hz, 2H), 6.90 (d, *J* = 2.2 Hz, 2H), 3.67 (q, *J* = 7.1 Hz, 8H), 1.34 (t, J = 7.0 Hz, 12H); 13C NMR (101 MHz, $CDCl_3$) δ158.1, 156.3, 155.7, 153.6, 152.6, 134.2, 132.0, 131.9, 130.6, 129.4, 123.4, 123.3, 114.5, 113.3, 96.9, 46.4, 12.8. ESI-HRMS (m/z): $[M]^+$ calcd. for $C_{33}H_{35}N_4O$, 503.2811; found 503.2808.

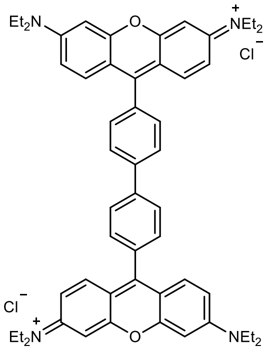

Synthesis of RD23. A solution of dilithium reagent **1** (2.6 equiv) in anhydrous THF was slowly added to a solution of compound **S23** (100 mg, 1 equiv) in anhydrous THF (20 mL) at −78 °C via syringe. The reaction mixture was allowed to warm to room temperature and stirred for another 2h. Saturated NH_4Cl solution (20 mL) was poured into the reaction flask, and the reaction mixture was stirred for another 2h before extracted with CH_2Cl_2 repeatedly (3X). The combined organic layer was dried with anhydrous $CaCl_2$ powder, filtered, and concentrated under reduced pressure to give a viscous residue, from which compounds **RD23** were obtained from a flash column over silica with a mixture of CH_2Cl_2/MeOH = 8:1, v/v. Compound **RD23** (164 mg) was obtained as a violet solid in a 56% yield. ^1H NMR (400 MHz, $CDCl_3$): δ 7.97 (d, *J* = 8.0 Hz, 4H), 7.55 (d, *J* = 8.0 Hz, 4H), 7.49 (d, *J* = 9.6 Hz, 4H), 6.96 (dd, *J* = 9.5 Hz, 1.6 Hz, 4H), 6.79 (d, *J* = 1.5 Hz, 4H), 3.63 (q, *J* = 7.0 Hz, 16H), 1.32 (t, *J* = 7.0 Hz, 24H); ^{13}C NMR (101 MHz, $CDCl_3$): δ 158.1, 157.2, 155.7, 142.1, 132.5, 131.5, 130.5, 128.1, 114.7, 113.4, 96.5, 46.4, 12.9. ESI-MS (m/z): 1/2$[M]^{2+}$ calcd. for $C_{27}H_{30}N_2O$, 398.2358; found 398.2358.

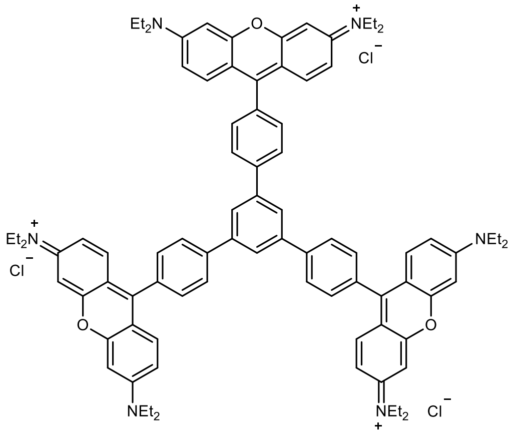

Synthesis of RD24. A solution of dilithium reagent **1** (3.9 equiv) in anhydrous THF was slowly added to a solution of compound **S24** (100 mg, 1 equiv) in anhydrous THF (20 mL) at −78 °C via syringe. The reaction mixture was allowed to warm to room temperature and stirred for another 2h. Saturated NH_4Cl solution (20 mL) was poured into the reaction flask, and the reaction mixture was stirred for another 2h before extracted with CH_2Cl_2 repeatedly (3X). The combined organic layer was dried with anhydrous $CaCl_2$ powder, filtered, and concentrated under reduced pressure to give

a viscous residue, from which compounds **RD24** were obtained from a flash column over silica with a mixture of CH_2Cl_2/MeOH = 6:1, v/v. Compound **(RD24)** (139 mg) was obtained as a violet solid in a 49% yield. ^{1}H NMR (400 MHz, $CDCl_3$): δ 8.06 (s, 3H), 8.02 (d, *J* = 7.9 Hz, 6H), 7.54 (d, *J* = 7.9 Hz, 6H), 7.50 (d, *J* = 9.6 Hz, 6H), 6.99 (dd, *J* = 9.5 Hz, 1.3 Hz, 6H), 6.77 (d, *J* = 1.2 Hz,6H), 3.60 (q, *J* = 7.0 Hz, 24H), 1.29 (t, J = 6.9 Hz, 36H); 13C NMR (101 MHz, $CDCl_3$) δ 158.0, 157.1, 155.6, 142.9, 141.7, 132.3, 131.1, 130.4, 128.1, 126.2, 114.5, 113.3, 96.3, 46.1, 12.6. ESI-HRMS (m/z): 1/3$[M]^{3+}$ calcd. for $C_{87}H_{93}N_6O_3$, 423.2436; found 423.2438

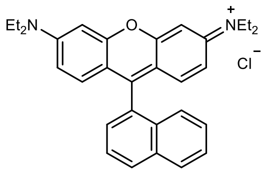

N-(6-(diethylamino)-9-(naphthalen-1-yl)-3H-xanthen-3-ylidene)-N-ethylethanaminium chloride salt (RD25[20]). Compound **RD25** was prepared from **S25** (100 mg, 1 equiv) and dilithium reagent **1** (1.3 equiv) according to general procedure B. Violet solid (206 mg) was obtained in a 79% yield by flash chromatography (CH_2Cl_2/MeOH = 15:1, v/v). ^{1}H NMR (400 MHz, $CDCl_3$) δ 8.08 (d, J = 8.2 Hz, 1H), 7.99 (d, J = 8.2 Hz, 1H), 7.56 (t, J = 7.1 Hz, 1H), 7.52 (d, J = 7.7 Hz, 1H), 7.38 (t, J = 8.0 Hz, 1H), 7.32 (d, J = 8.9 Hz, 1H), 7.05 (d, J = 9.5 Hz, 2H), 6.85 (d, J= 2.0 Hz, 2H), 6.74 (dd, J = 8.4, 2.0 Hz, 2H), 3.61–3.60 (m, 8H), 1.29–1.27 (m, 12H).

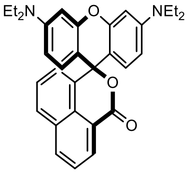

3',6'-bis(diethylamino)-3H-spiro[benzo[de]isochromene-1,9'-xanthen]-3-one (RD26). Compound **RD26** (211 mg) was prepared from **S26** (100 mg, 1 equiv) and dilithium reagent **1** (1.3 equiv) according to general procedure B. Pink solid was obtained in an 85% yield by flash chromatography (CH_2Cl_2/MeOH = 15:1, v/v). ^{1}H NMR (400 MHz, $CDCl_3$): δ 8.55 (d, *J* = 7.0 Hz, 1H), 8.20 (d, *J* = 8.2 Hz, 1H),7.89 (d, *J* = 8.2 Hz, 1H), 7.72 (t, *J* = 7.8 Hz, 1H), 7.49 (t, *J* = 8.2 Hz, 1H), 7.22 (d, *J* = 7.2 Hz, 1H), 6.71 (d, *J* = 8.9 Hz, 2H), 6.43 (d, *J* = 2.5 Hz, 2H), 6.27 (dd, *J* = 8.9 Hz, 2.5Hz, 2H) , 3.33 (q, *J* = 7.0 Hz, 8H), 1.15 (t, *J* = 7.0 Hz, 12H); ^{13}C NMR (101 MHz, $CDCl_3$): δ 163.5, 151.9, 149.1, 136.6, 133.5, 131.8, 129.9, 129.4, 128.6, 128.2, 126.9, 126.8, 126.2, 121.1, 111.4, 108.1, 97.4, 44.5, 12.6; EI-HRMS (m/z): $[M+H]^+$ calcd. for $C_{32}H_{33}N_2O_3$, 493.2491; found 493.2491.

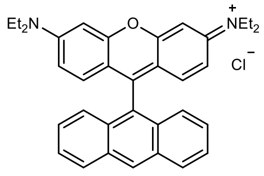

N-(9-(anthracen-9-yl)-6-(diethylamino)-3H-xanthen-3-ylidene)-N-ethylethanaminium chloride salt (RD27). Compound **RD27** was prepared from **S27** (100 mg, 1 equiv) and dilithium reagent **1** (1.3 equiv) according to general procedure B. Violet solid (172 mg) was obtained in a 76% yield by flash chromatography (CH_2Cl_2/MeOH = 12:1, v/v). ^{1}H NMR (400 MHz, $CDCl_3$): δ 8.72 (s, 1H), 8.15 (d, *J* = 8.5 Hz, 2H), 7.52 (d, *J* = 7.0 Hz, 2H), 7.41-7.34 (m, 4H), 7.02 (d, *J* = 2.0 Hz, 2H), 6.76 (dd, *J* = 9.5 Hz, 1.6 Hz, 2H), 6.67 (dd, *J* = 9.5 Hz, 2.1 Hz, 2H), 3.63 (q, *J* = 7.0 Hz, 8H), 1.30 (t, *J* = 7.0 Hz, 12H);

^{13}C NMR (101 MHz, $CDCl_3$) δ 158.0, 156.3, 155.9, 131.9, 131.0, 130.0, 129.9, 129.1, 127.7, 126.0, 125.0, 115.2, 114.5, 96.9, 46.4, 12.8. ESI-MS (m/z): $[M]^+$ calcd. for $C_{35}H_{35}N_2O$, 499.2749; found 499.2751

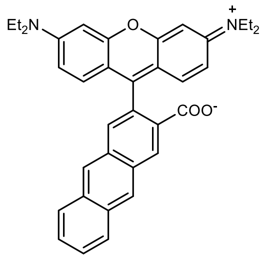

3-(6-(diethylamino)-3-(diethyliminio)-3H-xanthen-9-yl)anthracene-2-carboxylate (RD28) Compound **RD28** was prepared from **S28** (100 mg, 1 equiv) and dilithium reagent **1** (1.3 equiv) according to general procedure B. Violet solid (181 mg) was obtained in a 72% yield by flash chromatography (CH_2Cl_2/MeOH = 10:1, v/v). ^1H NMR (400 MHz, $CDCl_3$): δ 9.04 (s, 1H), 8.60 (s, 1H), 8.39 (s, 1H), 8.04 (s, 1H), 7.98 (s, 1H), 7.77 (s, 1H), 7.52 (s, 2H), 7.17 (d, *J* = 8.4 Hz, 2H), 6.66 (s, 4H), 3.50 (q, *J* = 7.8 Hz, 8H), 1.23 (t, J = 7.8 Hz, 12H); ^{13}C NMR (101 MHz, $CDCl_3$) δ 157.6, 154.8, 133.6, 133.4, 132.7, 132.0, 130.9, 130.5, 129.4, 128.8, 128.7, 128.3, 127.0, 126.8, 126.60, 114.0, 113.3, 96.1, 45.9, 12.7. ESI-HRMS (m/z): $[M]^+$ calcd. for $C_{36}H_{35}N_2O_3$, 543.2648; found. 543.2651

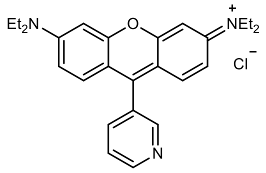

N-(6-(diethylamino)-9-(pyridin-3-yl)-3H-xanthen-3-ylidene)-N-ethylethanaminium chloride salt (RD29) Compound **RD29** was prepared from **S29** (100 mg, 1 equiv) and dilithium reagent **1** (1.3 equiv) according to general procedure B. Violet solid (243 mg) was obtained in a 76% yield by flash chromatography (CH_2Cl_2/MeOH = 12:1, v/v). ^1H NMR (400 MHz, $CDCl_3$): δ 8.86 (d, *J* = 4.0 Hz, 1H), 8.61 (s, 1H), 7.85 (d, *J* = 7.6 Hz, 1H), 7.66 (m, 1 H), 7.27 (d, *J* = 9.0 Hz, 2H), 6.97 (dd, *J* = 9.5 Hz, 1.9 Hz, 2H), 6.87 (d, *J* = 2.0 Hz, 2H), 3.66 (q, *J* = 7.0 Hz, 8H), 1.33 (t, *J* = 7.0 Hz, 12H); 13C NMR (101 MHz, $CDCl_3$): δ 157.9, 155.8, 153.1, 151.6, 149.2, 137.5, 131.5, 128.3, 124.1, 114.8, 113.5, 96.9, 46.4, 12.8. ESI-MS (m/z): $[M]^+$ calcd. for $C_{26}H_{30}N_3O$, 400.2389; found 400.2389.

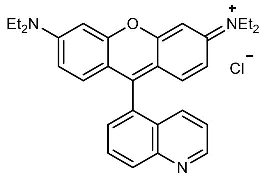

N-(6-(diethylamino)-9-(quinolin-5-yl)-3H-xanthen-3-ylidene)-N-ethylethanaminium chloride salt (RD30) Compound **RD30** was prepared from **S30** (100 mg, 1 equiv) and dilithium reagent **1** (1.3 equiv) according to general procedure B. Violet solid (205 mg) was obtained as in a 79% yield by flash chromatography (CH_2Cl_2/MeOH = 12:1, v/v). ^1H NMR (400 MHz, $CDCl_3$): δ 9.06 (d, *J* = 3.1 Hz, 1H), 8.38 (d, *J* = 8.1 Hz, 1H), 8.32 (d, *J* = 8.1 Hz, 1H), 7.97 (s, 1H), 7.66 (d, *J* = 8.0 Hz, 1H), 7.58 (m, 1 H), 7.33 (d, *J* = 9.4 Hz, 2H), 6.92 (d, *J* = 9.6 Hz, 2H), 6.87 (s, 2H), 3.65 (q, *J* = 6.9 Hz, 8H), 1.32 (t, *J* = 6.9 Hz, 12H); ^{13}C NMR (101 MHz, $CDCl_3$): δ13C NMR (101 MHz, CDCl3) δ 158.1, 156.3, 155.7, 152.4, 148.4, 137.0, 132.1, 130.4, 130.1, 129.9, 129.7, 128.1, 122.8, 114.5, 113.5, 96.7, 46.4, 12.8. ESI-MS

(m/z): [M]+ calcd. for $C_{30}H_{32}N_3O$, 450.2545; found 450.2548.

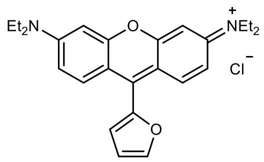

N-(6-(diethylamino)-9-(furan-2-yl)-3H-xanthen-3-ylidene)-N-ethylethanaminium chloride salt (RD31) Compound **RD31** was prepared from **S31** (100 mg, 1 equiv) and dilithium reagent **1** (1.3 equiv) according to general procedure B. Violet solid (233 mg) was obtained in a 77% yield by flash chromatography (CH_2Cl_2/MeOH = 12:1, v/v). ^1H NMR (400 MHz, $CDCl_3$): δ 8.00 (d, *J* = 9.7 Hz, 2H), 7.93 (s, 1H), 7.16 (d, *J* = 2.9 Hz, 1H), 7.04 (d, *J* = 9.5 Hz, 2H), 6.84 (s, 1H), 6.77 (s, 2H), 3.65 (q, *J* = 7.0 Hz, 8H), 1.33 (t, *J* = 7.0 Hz, 12H); ^{13}C NMR (101 MHz, $CDCl_3$) δ 158.1, 155.3, 147.4, 145.8, 143.0, 132.1, 120.5, 114.5, 113.4, 111.6, 96.7, 46.3, 12.8. ESI-MS (m/z): [M]+ calcd. for $C_{25}H_{29}N_2O_2$, 389.2229; found 389.2230.

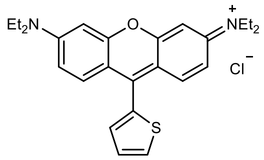

N-(6-(diethylamino)-9-(thiophen-2-yl)-3H-xanthen-3-ylidene)-N-ethylethanaminium chloride salt (RD32) Compound **RD32** was prepared from **S32** (100 mg, 1 equiv) and dilithium reagent **1** (1.3 equiv) according to general procedure B. Violet solid (216 mg) was obtained in a 76% yield by flash chromatography (CH_2Cl_2/MeOH = 12:1, v/v). ^1H NMR (400 MHz, $CDCl_3$): δ 7.76 (d, *J* = 3.8 Hz, 1H), 7.67 (d, *J* = 9.5 Hz, 2H), 7.34 (s, 2H), 6.98 (d, *J* = 9.3 Hz, 2H), 6.80 (s, 2H), 3.65 (q, *J* = 6.8 Hz, 8H), 1.32 (t, *J* = 6.8 Hz, 12H); 13C NMR (101 MHz, $CDCl_3$) δ 157.8, 155.5, 150.1, 132.2, 132.1, 131.0, 130.6, 128.4, 114.4, 113.8, 96.6, 46.3, 12.8. ESI-MS (m/z): [M]+ calcd. for $C_{25}H_{29}N_2OS$, 405.2001; found 405.2000.

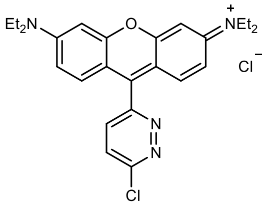

N-(9-(6-chloropyridazin-3-yl)-6-(diethylamino)-3H-xanthen-3-ylidene)-N-ethylethanaminium chloride salt (RD33) Compound **RD33** was prepared from **S33** (100 mg, 1 equiv) and dilithium reagent **1** (1.3 equiv) according to general procedure B. Violet solid (109 mg) was obtained in a 40% yield by flash chromatography (CH_2Cl_2/MeOH = 10:1, v/v). ^1H NMR (400 MHz, $CDCl_3$): δ 8.58 (d, *J* = 8.7 Hz, 1H), 7.28 (d, *J* = 8.4 Hz, 1H), 7.33 (d, *J* = 9.5 Hz, 2H), 6.95 (dd, *J* = 9.6 Hz, 1.8 Hz, 2H), 6.78 (d, *J* = 1.8 Hz, 2H), 3.63 (q, *J* = 7.0 Hz, 8H), 1.34 (t, J = 7.0 Hz, 12H); ^{13}C NMR (101 MHz, $CDCl_3$) δ 158.4, 158.2, 155.8, 154.2, 150.0, 133.3, 131.8, 130.6, 115.0, 113.2, 100.1, 96.6, 77.5, 12.8. ESI-HRMS (m/z): [M]+ calcd. for $C_{25}H_{28}ClN_4O$, 435.1952; found 435.1953

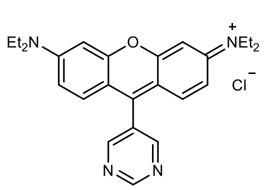

N-(6-(diethylamino)-9-(pyrimidin-5-yl)-3H-xanthen-3-ylidene)-N-ethylethanaminium chloride salt (RD34). Compound **RD34** was prepared from **S34** (100 mg, 1 equiv) and dilithium reagent **1** (1.3 equiv) according to general procedure B. Violet solid (120 mg) was obtained in a 38% yield by flash chromatography (CH_2Cl_2/MeOH = 10:1, v/v). ^{1}H NMR (400 MHz, $CDCl_3$): δ 9.48 (s, 1H), 8.85 (s, 2H), 7.21 (d, *J* = 9.2 Hz, 2H), 7.01 (d, *J* = 9.4 Hz, 2H), 6.97 (s, 2H), 3.67 (q, J = 7.0 Hz, 8H), 1.34 (t, J = 6.7 Hz, 12H); ^{13}C NMR (101 MHz, CDCl3) δ 160.1, 157.8, 156.9, 156.0, 148.5, 130.7, 115.2, 113.5, 97.5, 46.6, 12.9. ESI-HRMS (m/z): $[M]^+$ calcd. for $C_{25}H_{29}N_4O$, 401.2341; found 401.2342

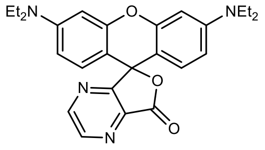

3',6'-bis(diethylamino)-7H-spiro[furo[3,4-b]pyrazine-5,9'-xanthen]-7-one (RD35) Compound **RD35** was prepared from **S35** (100 mg, 1 equiv) and dilithium reagent **1** (1.3 equiv) according to general procedure B. Violet solid (132 mg) was obtained in a 44% yield by flash chromatography (CH_2Cl_2/MeOH = 10:1, v/v). ^{1}H NMR (400 MHz, $CDCl_3$): δ 8.94 (s, 1H), 8.70 (s, 1H), 7.16 (s, 2H), 6.76 (s, 2H), 6.68 (s, 2H), 3.53 (q, *J* = 8.0 Hz, 8H), 1.25 (t, *J* = 6.6 Hz, 12H); ^{13}C NMR (101 MHz, $CDCl_3$) δ157.9, 157.9, 154.9, 131.9, 131.8, 113.9, 113.8, 112.6, 112.5, 96.3, 46.0, 12.8. ESI-HRMS (m/z): $[M]^+$ calcd. for $C_{26}H_{29}N_4O_3$, 445.2240; found 445.2242.

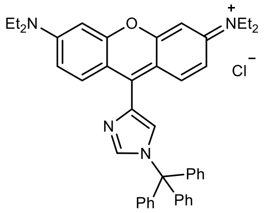

N-(6-(diethylamino)-9-(1-trityl-1H-imidazol-4-yl)-3H-xanthen-3-ylidene)-N-ethylethanaminium chloride salt (RD36). Compound **RD36** was prepared from **S36** (100 mg, 1 equiv) and dilithium reagent **1** (1.3 equiv) according to general procedure B. Violet solid (82 mg) was obtained in a 47% yield by flash chromatography (CH_2Cl_2/MeOH = 10:1, v/v). ^{1}H NMR (400 MHz, $CDCl_3$): δ 7.99 (d, *J* = 9.6 Hz, 2H), 7.80 (s, 1H), 7.42-7.41 (m, 9H), 7.30 (s, 1H), 7.22-7.20 (m, 6H), 6.88 (dd, *J* = 9.6 Hz, 1.8 Hz, 2H), 6.73 (d, *J* = 1.9 Hz, 2H), 3.88 (s, 3H), 3.63 (q, *J* = 7.0 Hz, 8H), 1.31 (t, J = 7.0 Hz, 12H); ^{13}C NMR (101 MHz, $CDCl_3$) δ 158.2, 155.3, 148.7, 141.7, 141.1, 141.0, 132.5, 129.7, 128.9, 128.7, 127.5, 114.0, 112.6, 96.6, 46.2, 12.9. ESI-HRMS (m/z): $[M]^+$ calcd. for $C_{43}H_{43}N_4O$, 631.3437; found 631.3438.

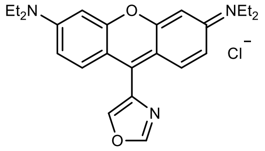

N-(6-(diethylamino)-9-(oxazol-4-yl)-3H-xanthen-3-ylidene)-N-ethylethanaminium chloride salt (RD37). Compound **RD37** was prepared from **S37** (100 mg, 1 equiv) and dilithium reagent **1** (1.3 equiv) according to general procedure B. Violet solid (84 mg) was obtained in a 28% yield by flash chromatography (CH_2Cl_2/MeOH = 10:1, v/v). ^{1}H NMR (400 MHz, $CDCl_3$): δ 8.74 (s, 1H), 8.26 (s, 1H), 8.02 (d, *J* = 9.2 Hz, 2H), 7.07 (d, *J* = 8.7 Hz, 2H), 6.78 (s, 2H), 3.64 (q, *J* = 7.0 Hz, 8H), 1.34 (t, J = 7.0 Hz, 12H); ^{13}C NMR (101 MHz, $CDCl_3$) δ 158.1, 155.6, 152.4, 143.3, 132.1, 129.4, 114.7, 112.8, 96.6, 46.3, 12.9. ESI-HRMS (m/z): $[M]^+$ calcd. for $C_{24}H_{28}N_3O_2$, 390.2182; found 390.2181.

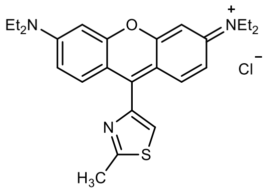

N-(6-(diethylamino)-9-(2-methylthiazol-4-yl)-3H-xanthen-3-ylidene)-N-ethylethanaminium chloride salt (RD38). Compound **(RD38)** was prepared from **S38** (100 mg, 1 equiv) and dilithium reagent **1** (1.3 equiv) according to general procedure B. Violet solid (95 mg) was obtained in a 32% yield by flash chromatography (CH_2Cl_2/MeOH = 10:1, v/v). ^1H NMR (400 MHz, $CDCl_3$): δ 7.78 (s, 1H), 7.72 (d, *J* = 9.3 Hz, 2H), 6.98 (d, *J* = 9.1 Hz, 2H), 6.73 (s, 2H), 3.62 (q, *J* = 6.8 Hz, 8H), 2.82 (s, 3H), 1.29 (t, J = 6.8 Hz, 12H); ^{13}C NMR (101 MHz, $CDCl_3$) δ 168.0, 158.1, 155.5, 149.3, 145.2, 132.3, 125.1, 114.5, 113.1, 96.4, 46.2, 29.7, 19.4, 12.8. ESI-HRMS (m/z): $[M]^+$ calcd. for $C_{25}H_{30}N_3OS^+$, 420.2110; found 420.2106

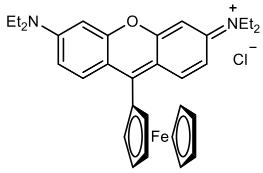

Compound **RD39** was prepared from **S39** (100 mg, 1 equiv) and dilithium reagent **1** (1.3 equiv) according to general procedure B. Violet solid (169 mg) was obtained as a violet solid in a 76% yield by flash chromatography (CH_2Cl_2/MeOH = 10:1, v/v). ^1H NMR (400 MHz, $CDCl_3$): δ 8.57 (d, *J* = 9.4 Hz, 2H), 6.91 (d, *J* = 9.2 Hz, 2H), 6.59 (s, 2H), 4.81 (d, *J* = 16.7 Hz, 4H), 4.18 (s, 5H), 3.52 (q, *J* = 7.2 Hz, 8H), 1.24 (t, *J* = 7.0 Hz, 12H); ^{13}C NMR (101 MHz, $CDCl_3$) δ157.1, 154.8, 132.6, 113.6, 113.1, 96.3, 74.7, 72.2, 71.6, 46.1, 12.8; ESI-MS (m/z): $[M]^+$ calcd. for $C_{31}H_{35}FeN_2O$, 507.2099; found 507.2100.

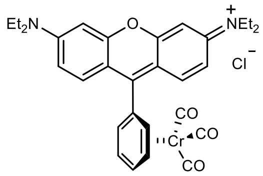

Compound **RD40** was prepared from **S40** (100 mg, 1 equiv) and dilithium reagent **1** (1.3 equiv) according to general procedure B. Violet solid (189 mg) was obtained as a violet solid in a 46% yield by flash chromatography (CH_2Cl_2/MeOH = 10:1, v/v). ^1H NMR (400 MHz, $CDCl_3$): δ 7.43 (d, *J* = 8.8 Hz, 2H), 6.52 (d, *J* = 8.6 Hz, 2H), 6.32 (s, 2H), 5.50 (d, *J* = 6.4 Hz, 2H), 5.33-5.31 (m, 1H), 4.98 (t, *J* = 6.3 Hz, 2H), 3.36 (q, *J* = 6.9 Hz, 8H), 1.18 (t, *J* = 6.9 Hz, 12H); ^{13}C NMR (101 MHz, $CDCl_3$) δ 153.3, 153.2, 148.9, 148.7, 128.7, 108.3, 108.0, 97.4, 94.4, 88.9, 44.5, 12.8; ESI-MS (m/z): $[M]^+$ calcd. for $C_{30}H_{31}CrN_2O_4$, 535.1689; found 535.1689.

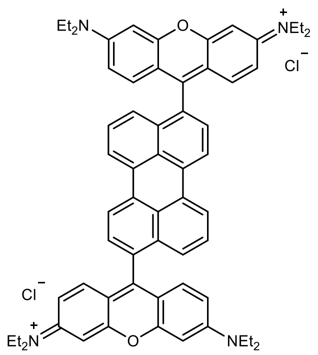

Compound **RD41** was prepared from **S41** (100 mg, 1 equiv) and dilithium reagent **1** (2.6 equiv) according to general procedure B. Violet solid (112 mg) was obtained as a violet solid in a 55% yield by flash chromatography (CH_2Cl_2/MeOH = 6:1, v/v). ^{1}H NMR (400 MHz, $CDCl_3$): δ 8.59 (d, *J* = 7.7 Hz, 1H), 8.49 (d, *J* = 7.7 Hz, 1H), 8.44 (d, *J* = 7.7 Hz, 1H), 8.36 (d, *J* = 7.6 Hz, 1H), 7.59-7.48 (m, 4H), 7.32-7.25 (m, 6H), 6.90-6.88 (m, 8H), 3.66 (q, *J* = 6.4 Hz, 16H), 1.35 (t, J = 6.4 Hz, 24H); 13C NMR (101 MHz, $CDCl_3$) δ 158.0, 156.3, 155.8, 133.1, 132.8, 132.7, 132.0, 131.3, 130.9, 129.8, 129.3, 128.7, 128.5, 128.3, 126.0, 125.5, 122.3, 122.0, 120.8, 120.5, 114.4, 114.1, 96.5, 46.2, 12.6, ESI-HRMS (m/z): 1/2$[M]^{2+}$ calcd. for $C_{62}H_{62}N_4O_2$, 894.4873 ; found 447.2434.

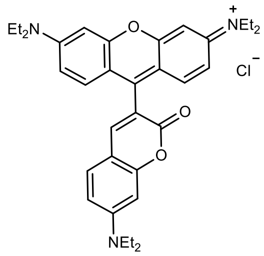

N-(6-(diethylamino)-9-(7-(diethylamino)-2-oxo-2H-chromen-3-yl)-3H-xanthen-3-ylidene)-N-ethylethanaminium chloride salt (RD42) Compound **RD42** was prepared from **S42** (100 mg, 1 equiv) and dilithium reagent **1** (1.3 equiv) according to general procedure B. Violet solid (47 mg) was obtained as a violet solid in a 23% yield by flash chromatography (CH_2Cl_2/MeOH = 10:1, v/v). ^{1}H NMR (400 MHz, $CDCl_3$): δ 8.03 (d, *J* = 9.4 Hz, 2H), 7.91 (s, 1H), 7.34 (d, *J* = 9.0 Hz, 1H), 7.04 (s, 1H), 6.81 (dd, *J* = 9.6Hz, 1.6 Hz, 2H), 6.54 (d, *J* = 1.9 Hz, 2H), 6.24 (d, *J* = 9.0 Hz, 1H), 3.53 (q, *J* = 7.0 Hz, 8H), 3.46 (q, *J* = 7.1 Hz, 4H), 1.30 (t, *J* = 7.0 Hz, 12H), 1.24 (t, *J* = 7.1 Hz, 12H); ^{13}C NMR (101 MHz, $CDCl_3$)δ 161.5, 156.68, 153.7, 130.6, 112.2, 112.0, 111.3, 105.5, 96.4, 45.5, 45.1, 13.1, 12.8.; ESI-MS (m/z): $[M]^+$ calcd. for $C_{34}H_{40}N_3O_3$, 538.3070; found 538.3072

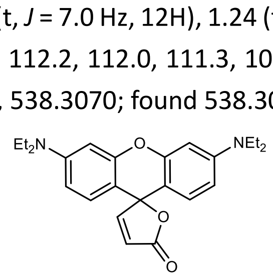

3',6'-bis(diethylamino)-5H-spiro[furan-2,9'-xanthen]-5-one (RD43). Compound **RD43** (254 mg) was prepared from **S43** (100 mg, 1 equiv) and dilithium reagent **1** (1.3 equiv) according to general procedure B. Violet solid was obtained in a 63% yield by flash chromatography (CH_2Cl_2/MeOH = 15:1, v/v). ^{1}H NMR (400 MHz, $CDCl_3$): δ 7.28 (d, *J* = 5.4 Hz, 1H), 7.99 (d, *J* = 8.8 Hz, 2H), 6.44 (dd, *J* = 8.8 Hz, 1.9 Hz, 2H), 6.39 (d, *J* = 1.9 Hz, 2H), 6.29 (d, *J* = 5.4 Hz, 1H), 3.36 (t, *J* = 7.0 Hz, 8H), 1.17 (q, *J* = 7.0 Hz, 12H); ^{13}C NMR (101 MHz, $CDCl_3$): δ 173.2, 157.8, 152.8, 149.7, 128.2, 120.4, 108.0, 102.5, 98.2, 44.6, 12.6. ESI-HRMS (m/z): $[M+H]^+$ calcd. for $C_{24}H_{29}N_2O_3$, 393.2178; found 393.2176.

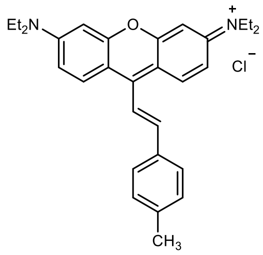

(E)-N-(6-(diethylamino)-9-(4-methylstyryl)-3H-xanthen-3-ylidene)-N-ethylethanaminium chloride salt (RD44) Compound **RD44** was prepared from **S44** (100 mg, 1 equiv) and dilithium reagent **1** (1.3 equiv) according to general procedure B. Violet solid (86 mg) was obtained as a violet solid in a 31% yield by flash chromatography (CH_2Cl_2/MeOH = 15:1, v/v). ^{1}H NMR (400 MHz, $CDCl_3$): δ 8.00 (d, *J* = 9.6 Hz, 2H), 7.63 (d, *J* = 16.2 Hz, 1H), 7.59 (d, *J* = 8.0 Hz, 2H), 7.25 (d, *J* = 8.0 Hz, 2H), 7.15 (d, *J* = 16.2 Hz, 1H), 7.02 (dd, *J* = 9.6Hz, 2.2 Hz, 2H), 6.71 (d, *J* = 2.2 Hz, 2H), 3.62 (q, *J* = 7.1 Hz, 8H), 2.38 (s, 3H), 1.31 (t, *J* = 7.1 Hz, 12H); ^{13}C NMR (101 MHz, $CDCl_3$) δ157.7, 155.3, 154.1, 145.0, 141.2, 132.4, 130.9, 130.0, 128.1, 117.7, 114.0, 112.2, 96.5, 46.1, 21.6, 12.8; ESI-MS (m/z): $[M]^+$ calcd. for $C_{30}H_{35}N_2O$, 439.2749; found 439.2749.

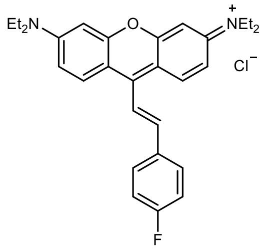

(E)-N-(6-(diethylamino)-9-(4-fluorostyryl)-3H-xanthen-3-ylidene)-N-ethylethanaminium chloride salt (RD45) Compound **RD45** was prepared from **S45** (100 mg, 1 equiv) and dilithium reagent **1** (1.3 equiv) according to general procedure B. Violet solid (76 mg) was obtained in a 28% yield by flash chromatography (CH_2Cl_2/MeOH = 15:1, v/v). ^{1}H NMR (400 MHz, $CDCl_3$): δ 8.12 (d, *J* = 9.6 Hz, 2H), 7.86-7.79 (m, 3H), 7.18-7.14 (m, 3H), 7.07 (dd, *J* = 9.6Hz, 2.5 Hz, 2H), 6.74 (d, *J* = 2.5 Hz, 2H), 3.64 (q, *J* = 7.1 Hz, 8H), 1.33 (t, *J* = 7.1 Hz, 12H); ^{13}C NMR (101 MHz, $CDCl_3$) δ 157.8, 155.5, 154.4, 143.6, 131.4, 130.5, 130.4, 116.6, 116.3, 114.2, 112.5, 96.6, 46.2, 12.8; ESI-MS (m/z): $[M]^+$ calcd. for $C_{29}H_{32}FN_2O$, 443.2499; found 443.2495.

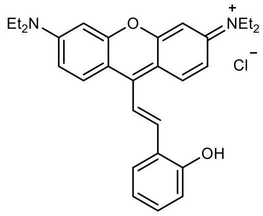

(E)-N-(6-(diethylamino)-9-(2-hydroxystyryl)-3H-xanthen-3-ylidene)-N-ethylethanaminium chloride salt (RD46). Compound **RD46** was prepared from **S46** (100 mg, 1 equiv) and dilithium reagent **1** (1.3 equiv) according to general procedure B. Violet solid (143 mg) was obtained in a 43% yield by flash chromatography (CH_2Cl_2/MeOH = 12:1, v/v). ^{1}H NMR (400 MHz, $CDCl_3$): δ 7.82 (d, *J* = 9.6Hz, 2H), 7.77 (d, *J* = 16.2 Hz, 1H), 7.63(d, *J* = 8.2 Hz, 1H), 7.37(d, *J* = 16.2 Hz, 1H), 7.36 (d, *J* = 8.2 Hz, 1H), 7.12 (t, *J* = 7.6Hz, 1H), 6.75(dd, *J* = 9.6Hz, 1.8Hz, 2H), 6.73(t, *J* = 7.6Hz, 1H), 6.55(d, *J* = 1.8Hz, 2H), 3.50 (q, *J* = 7.0 Hz, 8H), 1.23(t, *J* = 7.0 Hz, 12H);^{13}C NMR (101 MHz, $CDCl_3$) δ 158.6, 157.3, 155.1,

154.8, 143.6, 131.9, 130.8, 129.2, 122.1, 119.1, 118.3, 118.1, 113.4, 111.7, 96.3, 45.9, 12.7. ESI-HRMS (m/z): $[M]^+$ calcd. for $C_{29}H_{33}N_2O_2$, 441.2542; found 441.2542.

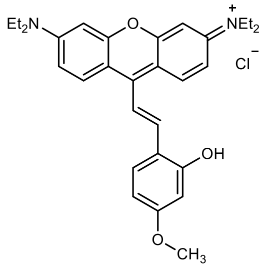

(E)-N-(6-(diethylamino)-9-(2-hydroxy-4-methoxystyryl)-3H-xanthen-3-ylidene)-N-ethylethanaminium chloride salt (RD47) Compound **RD47** was prepared from **S47** (100 mg, 1 equiv) and dilithium reagent **1** (1.3 equiv) according to general procedure B. Violet solid (119 mg) was obtained in a 41% yield by flash chromatography (CH_2Cl_2/MeOH = 12:1, v/v). ^1H NMR (400 MHz, $CDCl_3$): δ 8.02 (d, *J* = 9.5 Hz, 2H), 7.92 (d, *J* = 15.8 Hz, 1H), 7.57 (d, *J* = 15.8 Hz, 1H), 7.35 (d, *J* = 2.0 Hz, 1H), 7.30 (d, *J* = 8.7 Hz, 1H), 6.86 (dd, *J* = 9.6Hz, 1.9 Hz, 2H), 6.61 (d, *J* = 1.9 Hz, 2H), 6.39 (dd, *J* = 8.7 Hz, 2.0 Hz, 1H), 3.86 (s, 3H), 3.57 (q, *J* = 7.1 Hz, 8H), 1.31 (t, *J* = 7.1 Hz, 12H); 13C NMR (101 MHz, $CDCl_3$) δ 164.0, 161.8, 157.5, 154.7, 146.0, 131.2, 130.9, 115.9, 115.5, 113.1, 111.8, 108.4, 101.8, 96.3, 55.9, 45.8, 12.8; ESI-MS (m/z): $[M]^+$ calcd. for $C_{30}H_{35}N_2O_3$, 471.2648; found 471.2646.

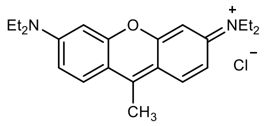

N-(6-(diethylamino)-9-methyl-3H-xanthen-3-ylidene)-N-ethylethanaminium chloride salt (**RD48**[25]). Compound **RD48** was prepared from **S48** (100 mg, 1 equiv) and dilithium reagent **1** (1.3 equiv) according to general procedure B. Violet solid (237 mg) was obtained as a red-violet solid in a 56% yield by flash chromatography (CH_2Cl_2/MeOH = 12:1, v/v). ^1H NMR (400 MHz, $CDCl_3$): δ 8.10 (d, *J* = 8.4 Hz, 2H), 7.11 (dd, *J* = 8.4 Hz, 1.2 Hz, 2H), 6.69 (d, *J* = 1.2 Hz, 2H), 3.63 (q, *J* = 7.1 Hz, 8H), 2.97(s, 3H), 1.33 (t, *J* = 7.1 Hz, 12H). ^{13}C NMR (101 MHz, $CDCl_3$): δ 157.5, 155.6, 130.4, 114.4, 113.87, 96.0, 46.1, 15.10, 12.8.

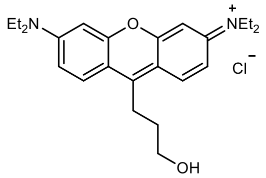

N-(6-(diethylamino)-9-(3-hydroxypropyl)-3H-xanthen-3-ylidene)-N-ethylethanaminium chloride salt (RD49) Compound **RD49** was prepared from **S49** (100 mg, 1 equiv) and dilithium reagent **1** (1.3 equiv) according to general procedure B. Violet solid (376 mg) was obtained as a violet solid in a 78% yield by flash chromatography (CH_2Cl_2/MeOH = 12:1, v/v). ^1H NMR (400 MHz, $CDCl_3$): δ 8.19 (d, *J* = 9.5 Hz, 2H), 7.03 (dd, *J* = 9.5Hz, 1.7 Hz, 2H), 6.71 (d, *J* = 1.7 Hz, 2H), 3.80 (t, *J* = 5 Hz, 2H), 3.59-3.52 (m, 10H), 1.99-1.92 (m, 2H), 1.27 (t, *J* = 7.1 Hz, 12H); ^{13}C NMR (101 MHz, $CDCl_3$) δ 162.7, 157.6, 155.4, 130.6, 114.2, 113.2, 95.8, 77.5, 77.2, 76.8, 60.8, 45.9, 34.9, 25.4, 12.7; ESI-MS (m/z): $[M]^+$ calcd. for $C_{24}H_{33}N_2O_2$, 381.2542; found 381.2542.

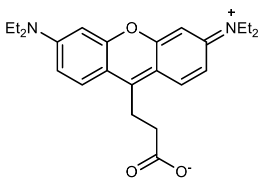

3-(6-(diethylamino)-3-(diethyliminio)-3H-xanthen-9-yl)propanoate (RD50). Compound **RD50** was prepared from **S1** (100 mg, 1 equiv) and dilithium reagent **1** (1.3 equiv) according to general procedure B. Violet solid (321 mg) was obtained in an 81% yield by flash chromatography (CH_2Cl_2/MeOH = 10:1, v/v). ^1H NMR (400 MHz, $CDCl_3$): δ 8.22 (d, *J*=9.4 Hz, 2H), 7.03 (d, *J* = 9.4 Hz, 2H), 6.64 (s, 2H), 3.72 (t, *J* = 7.4 Hz, 2H), 3.60(q, *J* = 7.0 Hz , 8H), 3.01(t, *J* = 7.4 Hz, 2H), 1.32 (q, *J* = 7.0 Hz, 12H); ^{13}C NMR (101 MHz, $CDCl_3$): δ 159.8, 157.7, 155.5, 130.5, 114.4, 113.2, 96.0, 46.0, 36.6, 24.2, 12.7; EI-HRMS (m/z): $[M]^+$ calcd. for $C_{24}H_{31}N_2O_3$, 395.2335; found 395.2333.

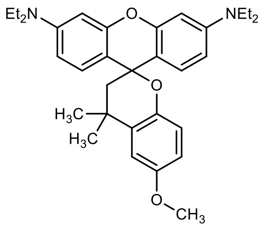

N3',N3',N6',N6'-tetraethyl-6-methoxy-4,4-dimethylspiro[chroman-2,9'-xanthene]-3',6'-diamine (RD51) Compound **RD51** was prepared from **S51** (100 mg, 1 equiv) and dilithium reagent **1** (1.3 equiv) according to general procedure B. White to pink solid (139 mg) was obtained in a 57% yield by flash chromatography (CH_2Cl_2/MeOH = 20:1, v/v).. ^1H NMR (400 MHz, $CDCl_3$): δ 7.18 (d, *J* = 8.7 Hz, 2H), 6.95 (d, *J* = 8.8 Hz, 1H), 6.85 (d, *J* = 2.9 Hz, 1H), 6.76 (dd, *J* = 8.8Hz, 2.9 Hz, 1H), 6.42 (d, *J* = 2.1 Hz, 2H), 6.36 (dd, *J* = 8.7Hz, 2.1 Hz, 2H), 3.82 (s, 3H), 3.32 (q, *J* = 7.0 Hz, 8H), 2.13 (s, 2H), 1.18 (s, 6H), 1.14 (t, *J* = 7.0 Hz, 12H); ^{13}C NMR (101 MHz, $CDCl_3$) δ 153.7, 152.7, 149.2, 148.2, 132.7, 126.7, 118.0, 116.3, 112.8, 112.0, 107.4, 98.9, 72.7, 55.9, 52.1, 44.6, 31.9, 31.8, 12.7; ESI-MS (m/z): $[M+H]^+$ calcd. for $C_{32}H_{41}N_2O_3$, 501.3117; found 501.3115

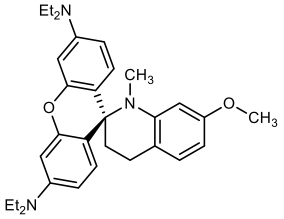

N3',N3',N6',N6'-tetraethyl-7-methoxy-1-methyl-3,4-dihydro-1H-spiro[quinoline-2,9'-xanthene]-3',6'-diamine (RD52) Compound **RD52** was prepared from **S52** (100 mg, 1 equiv) and dilithium reagent **1** (1.3 equiv) according to general procedure B. Pink solid (150 mg) was obtained in a 59% yield by flash chromatography (CH_2Cl_2/MeOH = 20:1, v/v). ^1H NMR (400 MHz, $CDCl_3$): δ 7.02 (d, *J* = 9.2 Hz, 2H), 6.93 (d, *J* = 8.1 Hz, 1H), 6.39-6.37 (m, 4H), 6.33 (d, *J* = 1.5Hz, 1H), 6.24 (dd, *J* = 8.1Hz, 1.5 Hz, 1H), 513.84 (s, 3H), 3.34 (q, *J* = 7.0 Hz, 8H), 2.68 (s, 3H), 2.65 (t, *J* = 6.1 Hz, 2H), 2.03 (t, *J* = 6.1 Hz, 2H), 1.18 (t, *J* = 7.0 Hz, 12H); ^{13}C NMR (101 MHz, $CDCl_3$) δ 159.6, 152.5, 148.1, 128.6, 127.8, 116.3, 115.0, 107.2, 99.7, 98.5, 97.6, 77.5, 77.2, 76.8, 58.6, 55.4, 44.5, 41.5, 35.4, 23.9, 12.8; ESI-MS (m/z): $[M+H]^+$ calcd. for $C_{31}H_{40}N_3O_2$, 486.3121; found 486.3121

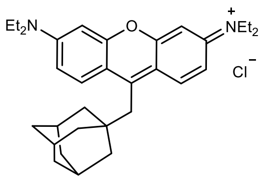

N-(9-((3r,5r,7r)-adamantan-1-ylmethyl)-6-(diethylamino)-3H-xanthen-3-ylidene)-N-ethylethanaminium chloride salt (RD53). Compound **RD53** was prepared from **S53** (100 mg, 1 equiv) and dilithium reagent **1** (1.3 equiv) according to general procedure B. Violet solid (186 mg) was obtained in a 77% yield by flash chromatography (CH_2Cl_2/MeOH = 10:1, v/v). ^{1}H NMR (400 MHz, $CDCl_3$): δ 7.87 (d, *J* = 9.6 Hz, 2H), 6.96 (dd, *J* = 9.5 Hz, 2H), 6.57 (d, *J* = 1.0 Hz, 2H), 3.53 (q, J = 7.0 Hz, 8H), 3.05 (s, 2H), 1.81 (s, 3H), 1.51-1.41 (m, 12H), 1.21 (t, J = 7.1 Hz, 12H). ^{13}C NMR (101 MHz, $CDCl_3$): δ 157.6, 156.9, 155.0, 130.9, 114.4, 113.3, 95.6, 45.7, 43.5, 40.8, 37.2, 36.1, 28.6, 12.5. EI-HRMS (m/z): [M]$^+$ calcd. for $C_{32}H_{43}N_2O$, 471.3375; found 471.3376

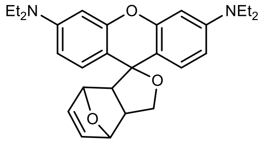

N3',N3',N6',N6'-tetraethyl-3a,4,7,7a-tetrahydro-3H-spiro[4,7-epoxyisobenzofuran-1,9'-xanthene]-3',6'-diamine (RD54) Compound **RD54** was prepared from **S1** (100 mg, 1 equiv) and dilithium reagent **1** (1.3 equiv) according to general procedure B. Pink solid (204 mg) was obtained in a 84% yield by flash chromatography (CH_2Cl_2/MeOH = 20:1, v/v). ^{1}H NMR (400 MHz, $CDCl_3$): δ 7.54 (d, *J* = 8.8 Hz, 1H), 7.25 (d, *J* = 7.4 Hz, 1H), 6.50 (dd, *J* = 8.8 Hz, 2.5 Hz, 1H), 6.45 (dd, *J* = 8.7 Hz, 2.5Hz, 1H), 6.40 (d, *J* = 2.5 Hz, 1H), 6.37 (d, *J* = 2.5 Hz, 1H), 6.31 (dd, *J* = 5.8 Hz, 1.4 Hz, 1H), 6.19 (dd, *J* = 5.8 Hz, 1.4 Hz, 1H), 4.86 (s, 1H), 4.69 (t, *J* = 8.8 Hz, 1H), 4.31 (d, *J* = 1.0 Hz, 1H), 4.19 (dd, *J* = 8.8 Hz, 4.9 Hz, 1H), 3.39-3.31 (m, 8H), 2.91-2.86 (m, 1H), 2.33 (d, *J* = 7.7 Hz, 1H), 1.17 (t, *J* = 6.9 Hz, 6H), 1.16 (t, *J* = 6.9 Hz, 6H);^{13}C NMR (101 MHz, $CDCl_3$) δ 152.3, 151.0, 148.3, 147.9, 137.7, 136.8, 129.1, 125.8, 119.2, 112.1, 107.9, 107.5, 98.6, 98.2, 82.7, 80.7, 79.9, 73.8, 62.0, 47.9, 44.5, 12.7, 12.7. ESI-HRMS (m/z): [M+1]$^+$ calcd. for $C_{28}H_{34}N_2O_3$, 447.2648; found 447.2648

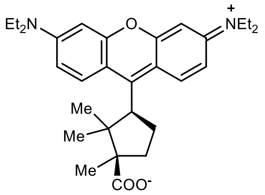

(1R,3S)-3-(3-(diethyl-l4-azanylidene)-6-(diethylamino)-3H-xanthen-9-yl)-1,2,2-trimethylcyclopentane-1-carboxylate (RD55) Compound **RD55** was prepared from **S1** (100 mg, 1 equiv) and dilithium reagent **1** (1.3 equiv) according to general procedure B. Violet solid (184 mg) was obtained in a 70% yield by flash chromatography (CH_2Cl_2/MeOH = 10:1, v/v). ^{1}H NMR (400 MHz, $CDCl_3$): δ 8.11(d, *J* = 9.9 Hz, 1H), 8.02 (d, *J* = 9.7 Hz, 1H), 7.11 (d, *J* = 9.0 Hz, 1H), 6.95 (d, *J* = 8.7 Hz, 1H), 6.67 (dd, *J* = 6.2 Hz, 1.4 Hz, 2H), 4.39 (t, *J* = 8.3 Hz, 1H), 3.61 (q, *J* = 7.8 Hz, 8H), 2.98-2.90 (m, 1H), 2.33 (s, 2H), 1.88-1.85 (m, 1H), 1.45 (s, 3H), 1.31 (t, *J* = 7.0 Hz, 12H), 1.20 (s, 3H), 0.81 (s, 3H);^{13}C NMR (101 MHz, $CDCl_3$) δ 162.4, 157.8, 157.3, 155.3, 154.6, 132.2, 130.8, 115.4, 114.6, 113.8, 113.0, 96.2, 57.1, 51.2, 48.1, 46.1, 46.0, 35.0, 30.1, 25.6, 25.2, 20.8, 12.9. ESI-HRMS (m/z): [M]$^+$ calcd. for $C_{30}H_{41}N_2O_3^+$, 477.3117; found 477.3120.

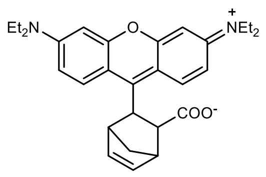

3-(6-(diethylamino)-3-(diethyliminio)-3H-xanthen-9-yl)bicyclo[2.2.1]hept-5-ene-2-carboxylate (RD56). Compound **RD56** was prepared from **S56** (100 mg, 1 equiv) and dilithium reagent **1** (1.3 equiv) according to general procedure B. Pink solid (175 mg) was obtained in a 75% yield by flash chromatography (CH_2Cl_2/MeOH = 12:1, v/v). ^1H NMR (400 MHz, $CDCl_3$): δ 7.19 (d, *J* = 8.6 Hz, 1H), 7.01 (d, *J* = 8.6 Hz, 1H), 6.48-6.42 (m, 4H), 6.33-6.31 (m, 1H), 6.17-6.15 (m, 1H), 3.57 (dd, *J* = 8.9 Hz, 5.1 Hz, 1H), 3.40-3.32 (m, 8H), 3.28 (s, 1H), 2.92 (dd, *J* = 8.9 Hz, 3.7 Hz, 1H), 2.61 (s, 1H), 1.46 (d, *J* = 8.0 Hz, 1H), 1.25 (d, *J* = 5.7 Hz, 1H), 1.19 (t, J = 7.0 Hz, 6H), 1.16 (t, J = 7.0 Hz, 6H); 13C NMR (101 MHz, $CDCl_3$) δ178.4, 153.1, 151.8, 148.8, 148.4, 136.0, 134.9, 127.4, 123.3, 117.9, 110.8, 107.4, 106.3, 99.4, 98.8, 81.6, 58.2, 53.1, 48.5, 46.0, 45.5, 44.6, 44.6, 12.7, 12.7. ESI-HRMS (m/z): $[M+1]^+$ calcd. for $C_{29}H_{34}N_2O_3$, 459.2648; found 459.2646.

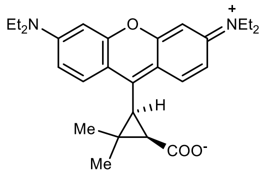

(1R,3S)-3-(3-(diethyl-l4-azanylidene)-6-(diethylamino)-3H-xanthen-9-yl)-2,2-dimethylcyclopropane-1-carboxylate (RD57). Compound **RD57** was prepared from **S57** (100 mg, 1 equiv) and dilithium reagent **1** (1.3 equiv) according to general procedure B. Violet solid (221 mg) was obtained in a 71% yield by flash chromatography (CH_2Cl_2/MeOH = 10:1, v/v). ^1H NMR (400 MHz, $CDCl_3$): δ 8.38 (s,.1H), 7.82 (s, 1H), 6.95 (d, *J* = 9.0 Hz, 2H), 6.62 (s, 2H), 3.57 (q, *J* = 7.0 Hz, 8H), 2.80 (d, *J* = 6.2 Hz, 1H), 2.23 (d, *J* = 6.2 Hz, 1H), 1.60 (s, 3H), 1.29 (t, J = 7.0 Hz, 12H); ^{13}C NMR (101 MHz, $CDCl_3$) δ 174.4, 159.5, 157.8, 155.3, 131.8, 115.3, 113.9, 95.8, 46.0, 38.6, 30.9, 26.8, 24.2, 20.3, 12.7. ESI-HRMS (m/z): $[M]^+$ calcd. for $C_{27}H_{35}N_2O_3$, 435.2648; found 435.2648.

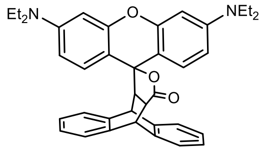

(9R,10S,11S,15R)-3',6'-bis(diethylamino)-10,11-dihydro-9H-spiro[9,10-[3,4]furanoanthracene-12,9'-xanthen]-14(15H)-one (RD58). Compound **RD58** was prepared from **S58** (100 mg, 1 equiv) and dilithium reagent **1** (1.3 equiv) according to general procedure B. White solid (140 mg) was obtained in a 68% yield by flash chromatography (CH_2Cl_2/MeOH = 20:1, v/v). ^1H NMR (400 MHz, $CDCl_3$): δ 7.38 (d, *J* = 7.2 Hz, 1H), 7.25 (d, *J* = 8.8 Hz, 1H), 7.16 (t, *J* = 7.4 Hz, 1H), 7.12-7.06 (m, 2H), 7.01-6.93 (m, 3H), 6.89 (d, *J* = 7.3 Hz, 1H), 6.55 (d, *J* = 2.1 Hz, 1H), 6.44 (d, *J* = 8.8 Hz, 1H), 6.43 (s, 1H), 6.39 (dd, *J* = 8.8 Hz, 2.2 Hz, 1H), 6.34 (dd, *J* = 8.7 Hz, 2.3 Hz, 1H), 4.78 (d, *J* = 4.2 Hz, 1H), 3.86 (d, *J* = 1.8 Hz, 1H), 3.62 (dd, *J* = 9.7 Hz, 4.2Hz, 1H), 3.44 (q, *J* = 7.0 Hz, 4H), 3.31 (q, *J* = 7.0 Hz, 4H), 2.69 (dd, *J* = 9.7 Hz, 1.9Hz, 1H), 1.25 (t, *J* = 7.0 Hz, 6H), 1.12 (t, *J* = 7.0 Hz, 6H); ^{13}C NMR (101 MHz, $CDCl_3$): δ 176.7, 153.1, 151.6, 149.0, 148.5, 145.1, 142.0, 141.9, 138.5, 128.7, 128.5, 126.6, 126.3, 126.0, 125.6, 125.1, 124.3, 123.3, 123.2, 116.8, 108.6, 107.3, 106.4, 99.2, 98.7, 82.2, 57.0, 47.8, 46.1, 45.5, 44.7, 44.6, 12.8, 12.6. EI-HRMS (m/z): $[M+H]^+$ calcd. for $C_{38}H_{39}N_2O_3$, 571.2961; found

571.2966

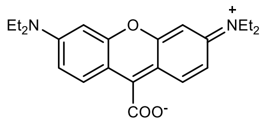

6-(diethylamino)-3-(diethyliminio)-3H-xanthene-9-carboxylate (RD59). Compound **RD59** was prepared from **S59** (100 mg, 1 equiv) and dilithium reagent **1** (1.3 equiv) according to general procedure B. Violet solid (90 mg) was obtained in a 36% yield by flash chromatography (CH_2Cl_2/MeOH = 8:1, v/v). ^{1}H NMR (400 MHz, $CDCl_3$): δ 8.13 (d, *J* = 9.4 Hz, 2H), 6.86 (dd, *J* = 9.2 Hz, 1.8 Hz, 2H), 6.58 (d, *J* = 1.8 Hz, 2H), 3.55 (q, *J* = 7.1 Hz, 8H), 1.30 (t, *J* = 7.1 Hz, 12H). ^{13}C NMR (101 MHz, $CDCl_3$): δ159.1, 155.4, 134.2, 113.2, 110.5, 100.1, 95.6, 45.8, 12.7. EI-HRMS (m/z): $[M]^+$ calcd. for $C_{22}H_{27}N_2O_3$, 367.2022; found 367.2021.

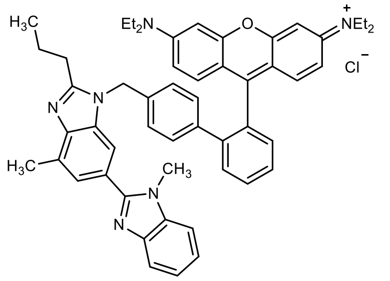

N-(6-(diethylamino)-9-(4'-((1,7'-dimethyl-2'-propyl-1H,3'H-[2,5'-bibenzo[d]imidazol]-3'-yl)methyl)-[1,1'-biphenyl]-2-yl)-3H-xanthen-3-ylidene)-N-ethylethanaminium chloride salt (RD60) Compound **RD60** was prepared from **S60** (100 mg, 1 equiv) and dilithium reagent **1** (1.3 equiv) according to general procedure B. Violet solid (81 mg) was obtained in a 53% yield by flash chromatography (CH_2Cl_2/MeOH = 10:1, v/v). ^{1}H NMR (400 MHz, $CDCl_3$): δ 7.77 (d, *J* = 6.9 Hz, 1H), 7.65 (t, *J* = 7.6 Hz, 1H), 7.56-7.50 (m, 3H), 7.41-7.40 (m, 2H), 7.29-7.19 (m, 5H), 7.01 (d, *J* = 7.9 Hz, 2H), 6.88 (d, *J* = 7.9 Hz, 2H), 6.83 (d, *J* = 9.4 Hz, 2H), 6.70 (s, 2H), 5.34 (s, 2H), 3.85 (s, 3H), 3.58 (q, *J* = 8.0 Hz, 8H), 2.72-2.68 (m, 5H), 1.68-1.62 (m, 2H), 1.27 (t, *J* = 7.8 Hz, 12H), 0.87 (t, *J* = 7.2 Hz, 3H);^{13}C NMR (101 MHz, $CDCl_3$) δ 157.5, 157.4, 156.5, 155.4, 154.5, 140.9, 139.1, 136.6, 135.8, 135.2, 131.8, 130.9, 130.6, 130.3, 130.1, 129.3, 128.8, 127.8, 126.6, 123.8, 123.6, 122.6, 122.4, 119.2, 114.2, 113.6, 109.8, 108.6, 96.3, 46.7, 46.1, 32.1, 29.6, 21.4, 16.8, 13.9, 12.6. ESI-HRMS (m/z): $[M]^+$ calcd. for $C_{53}H_{55}N_6O^+$, 791.4437; found 791.4438.

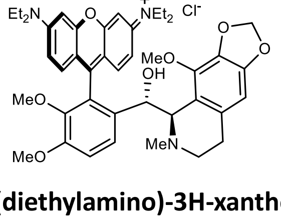

(S)-(2-(3-(diethyl-l4-azanylidene)-6-(diethylamino)-3H-xanthen-9-yl)-3,4-dimethoxyphenyl) ((R)-4-methoxy-6-methyl-5,6,7,8-tetrahydro-[1,3]dioxolo[4,5-g]isoquinolin-5-yl)methanol chloride salt (RD61) Compound **RD61** was prepared from **S61** (100 mg, 1 equiv) and dilithium reagent **1** (1.3 equiv) according to general procedure B. Purple solid (77 mg) was obtained in a 43% yield by flash chromatography (CH_2Cl_2/MeOH = 12:1, v/v). ^{1}H NMR (400 MHz, $CDCl_3$): δ 6.82 (d, *J* = 7.8 Hz, 1H), 6.67 (d, *J* = 8.7 Hz, 1H), 6.33 (s, 2H), 6.29 (s, 1H), 6.28 (d, *J* = 8.5 Hz, 1H), 6.17 (d, *J* = 5.2 Hz, 1H), 5.90 (d, *J* = 9.9 Hz, 2H), 3.75 (s, 3H), 3.70 (s, 3H), 3.64 (s, 1H), 3.35-3.27 (m, 8H), 3.06 (s, 3H), 2.67-2.60 (m, 3H), 2.54 (s, 3H), 1.34-1.25 (m, 2H), 1.17-1.10 (m, 14H); ^{13}C NMR (101 MHz, CDCl3) δ152.9, 152.1, 151.5, 148.5, 148.3, 143.4, 141.1, 134.4, 130.8, 128.7, 118.0, 113.1, 108.1, 107.3, 102.8,

100.74, 98.1, 97.6, 70.7, 62.4, 59.7, 59.0, 56.2, 44.5, 12.8. ESI-HRMS (m/z): $[M]^+$ calcd. for $C_{42}H_{49}N_3O_7$, 707.3571; found 707.3571

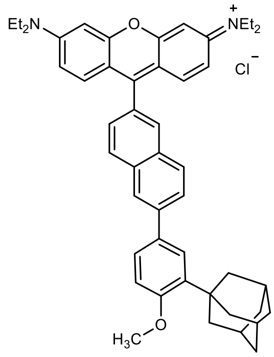

N-(9-(6-(3-((1s,3s)-adamantan-1-yl)-4-methoxyphenyl)naphthalen-2-yl)-6-(diethylamino)-3H-xanthen-3-ylidene)-N-ethylethanaminium chloride salt (RD62) Compound **RD62** was prepared from **S62** (100 mg, 1 equiv) and dilithium reagent **1** (1.3 equiv) according to general procedure B. Violet solid (114 mg) was obtained in a 68% yield by flash chromatography (CH_2Cl_2/MeOH = 10:1, v/v). ^1H NMR (400 MHz, $CDCl_3$): δ 8.11 (s, 1H), 8.09 (d, *J* = 8.6 Hz, 1H), 7.96 (d, *J* = 8.5 Hz, 1H), 7.87 (s, 2H), 7.61 (s, 1H), 7.57 (d, *J* = 8.3 Hz, 1H), 7.45 (d, *J* = 8.3 Hz, 1H), 7.41 (d, *J* = 9.5 Hz, 2H), 7.00 (d, *J* = 8.4 Hz, 1H), 6.85 (d, *J* = 9.7 Hz, 2H), 6.82 (s, 2H), 3.88 (s, 3H), 3.60 (q, *J* = 6.7 Hz, 8H), 2.17 (s, 6H), 2.08 (s, 3H), 1.77 (s, 6H), 1.30 (t, J = 6.8 Hz, 12H); 13C NMR (101 MHz, $CDCl_3$) δ 159.1, 158.1, 157.6, 155.6, 141.2, 139.2, 134.3, 132.4, 132.4, 131.5, 129.4, 129.0, 128.9, 128.8, 127.5, 126.8, 126.0, 125.9, 124.9, 114.3, 113.5, 112.3, 96.7, 55.3, 46.3, 40.7, 37.3, 37.2, 29.2, 12.8. ESI-HRMS (m/z): $[M]^+$ calcd. for $C_{48}H_{53}N_2O_2^+$, 689.4107; found 689.4106

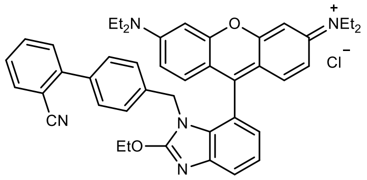

N-(9-(1-((2'-cyano-[1,1'-biphenyl]-4-yl)methyl)-2-ethoxy-1H-benzo[d]imidazol-7-yl)-6-(diethylamino)-3H-xanthen-3-ylidene)-N-ethylethanaminium chloride salt (RD63) Compound **RD63** was prepared from **S63** (100 mg, 1 equiv) and dilithium reagent **1** (1.3 equiv) according to general procedure B. Violet solid (90 mg) was obtained in a 52% yield by flash chromatography (CH_2Cl_2/MeOH = 12:1, v/v). ^1H NMR (400 MHz, $CDCl_3$): δ 7.78 (d, *J* = 7.9 Hz, 1H), 7.68-7.63 (m, 2H), 7.42 (t, *J* = 7.6 Hz, 1H),7.32-7.26 (m, 2H), 7.15 (t, *J* = 8.0 Hz, 2H), 6.92 (d, *J* = 9.5 Hz, 2H), 6.83 (d, *J* = 7.5 Hz, 1H), 6.76 (s, 2H), 6.68 (d, *J* = 9.4 Hz, 2H), 6.46 (d, *J* = 8.0 Hz, 2H), 4.72-4.66 (m, 4H), 3.56-3.51 (m, 8H), 1.46 (t, *J* = 7.1 Hz, 3H), 1.16 (t, *J* = 7.2 Hz, 12H);^{13}C NMR (101 MHz, $CDCl_3$) δ158.3, 157.0, 155.1, 153.3, 143.4, 141.2, 136.8, 136.4, 133.7, 133.3, 131.5, 130.5, 129.6, 128.5, 127.8, 125.2, 122.9, 121.7, 119.5, 118.6, 114.2, 114.0, 113.9, 109.9, 96.0, 66.9, 46.0, 45.8, 14.4, 12.3. ESI-HRMS (m/z): $[M]^+$ calcd. for $C_{44}H_{44}N_5O_2^+$, 674.3495; found 674.3494.

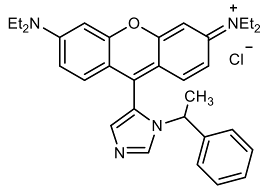

N-(6-(diethylamino)-9-(1-(1-phenylethyl)-1H-imidazol-5-yl)-3H-xanthen-3-ylidene)-N-

ethylethanaminium chloride salt (RD64) Compound **RD64** was prepared from **S64** (100 mg, 1 equiv) and dilithium reagent **1** (1.3 equiv) according to general procedure B. Violet solid (63 mg) was obtained in a 29% yield by flash chromatography (CH_2Cl_2/MeOH = 12:1, v/v). ^{1}H NMR (400 MHz, $CDCl_3$): δ 8.10 (s, 1H), 7.30 (d, *J* = 9.6 Hz, 2H), 7.09-7.02 (m, 4H), 6.98 (d, *J* = 9.5 Hz, 1H), 6.89 (s, 1H), 6.77-6.74 (m, 3H), 6.56 (dd, *J* = 9.3 Hz, 1.5 Hz, 1H), 5.01 (q, *J* = 6.8 Hz, 1H), 3.69 (q, *J* = 7.0 Hz, 4H), 3.60 (q, *J* = 7.0 Hz, 4H), 1.86 (d, J = 6.9 Hz, 3H), 1.36 (t, J = 7.0 Hz, 6H), 1.30 (t, J = 7.0 Hz, 6H); ^{13}C NMR (101 MHz, $CDCl_3$) δ157.7, 157.6, 155.8, 155.6, 144.7, 140.5, 131.4, 130.8, 129.0, 128.4, 125.8, 115.0, 114.2, 113.9, 113.8, 97.0, 96.8, 57.0, 46.4, 22.4, 12.8. ESI-HRMS (m/z): $[M]^+$ calcd. for $C_{32}H_{37}N_4O^+$, 493.2967; found 493.2968

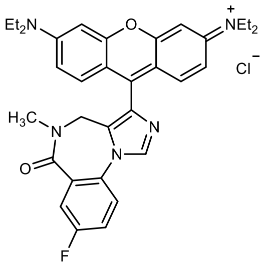

N-(6-(diethylamino)-9-(8-fluoro-5-methyl-6-oxo-5,6-dihydro-4H-benzo[f]imidazo[1,5-a][1,4]diazepin-3-yl)-3H-xanthen-3-ylidene)-N-ethylethanaminium chloride salt (RD65) Compound **RD65** was prepared from **S65** (100 mg, 1 equiv) and dilithium reagent **1** (1.3 equiv) according to general procedure B. Violet solid (39 mg) was obtained in a 20% yield by flash chromatography (CH_2Cl_2/MeOH = 12:1, v/v). ^{1}H NMR (400 MHz, $CDCl_3$): δ 8.19 (s, 1H), 7.91 (d, *J* = 7.8 Hz, 1H), 7.78 (d, *J* = 7.8 Hz, 1H), 7.74 (dd, *J* = 8.4 Hz, 2.2 Hz, 1H), 7.65-7.62 (m, 1H), 7.43-7.35 (m, 2H), 6.90 (d, *J* = 9.9 Hz, 1H), 6.79 (s, 2H), 3.72-3.55 (m, 10H), 2.93 (s, 3H), 1.34 (t, J = 7.0 Hz, 12H); ^{13}C NMR (101 MHz, $CDCl_3$) δ 165.7, 163.0, 161.0, 147.8, 137.2, 132.9, 132.6, 131.2, 131.1, 129.2, 128.6, 128.6, 124.8, 124.8, 120.5, 120.3, 119.5, 119.3, 116.4, 113.7, 96.5, 70.7, 46.4, 46.1, 43.6, 36.4, 12.8. ESI-HRMS (m/z): $[M]^+$ calcd. for $C_{33}H_{35}FN_5O_2^+$, 552.2775; found 552.2777

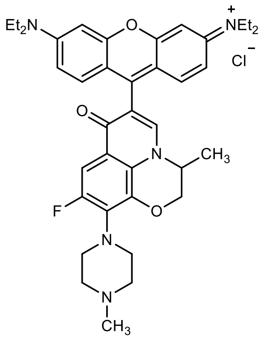

N-(6-(diethylamino)-9-(9-fluoro-3-methyl-10-(4-methylpiperazin-1-yl)-7-oxo-3,7-dihydro-2H-[1,4]oxazino[2,3,4-ij]quinolin-6-yl)-3H-xanthen-3-ylidene)-N-ethylethanaminium chloride salt (RD66) Compound **RD66** was prepared from **S1** (100 mg, 1 equiv) and dilithium reagent **1** (1.3 equiv) according to general procedure B. Violet solid (78 mg) was obtained as a violet solid in a 44% yield by flash chromatography (CH_2Cl_2/MeOH = 10:1, v/v). ^{1}H NMR (400 MHz, $CDCl_3$): δ 8.60 (s, 1H), 7.84 (d, *J* = 9.3 Hz, 1H), 7.61 (d, *J* = 12.4 Hz, 1H), 7.84 (d, *J* = 9.5 Hz, 1H), 6.92 (d, *J* = 8.8 Hz, 1H), 6.79 (dd, *J* = 9.5 Hz, 2.2 Hz, 1H), 6.10 (dd, *J* = 5.3 Hz, 2.3 Hz, 2H), 5.61 (s, 1H), 4.47(s, 2H), 3.58-3.48 (m, 8H), 3.46-3.38 (m, 4H), 2.69 (s, 4H), 2.44 (s, 3H), 1.53 (d, *J* = 6.0 Hz, 3H), 1.27-1.22 (m, 12H);^{13}C NMR (101 MHz, $CDCl_3$) δ 173.4, 173.4, 158.2, 157.9, 156.9, 155.6, 155.1, 154.4, 154.1, 142.8, 140.2,

140.1, 133.3, 132.4, 131.2, 131.1, 125.1, 122.0, 121.9, 114.6, 114.3, 114.1, 113.4, 111.7, 104.7, 104.5, 95.8, 95.7, 68.9, 55.4, 54.0, 49.9, 49.9, 45.9, 45.8, 45.8, 18.9, 12.6. ESI-HRMS (m/z): $[M]^+$ calcd. for $C_{38}H_{45}FN_5O_3^+$, 638.3506; found 638.3502.

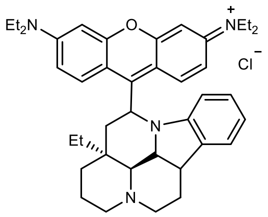

N-(6-(diethylamino)-9-((41S,13aS)-13a-ethyl-2,3,41,5,6,13a-hexahydro-1H-indolo[3,2,1-de]pyrido[3,2,1-ij][1,5]naphthyridin-12-yl)-3H-xanthen-3-ylidene)-N-ethylethanaminium chloride salt (RD67). Compound **RD67** was prepared from **S67** (100 mg, 1 equiv) and dilithium reagent **1** (1.3 equiv) according to general procedure B. Violet solid (116 mg) was obtained in a 64% yield by flash chromatography (CH_2Cl_2/MeOH = 12:1, v/v). ^1H NMR (400 MHz, $CDCl_3$): δ 7.80 (d, *J* = 9.4 Hz, 1H), 7.46 (d, *J* = 7.8 Hz, 1H), 7.27 (d, *J* = 7.5 Hz, 1H), 7.08-7.02 (m, 3H), 6.91 (s, 1H), 6.85 (t, *J* = 7.6 Hz, 1H), 6.75 (d, *J* = 8.9 Hz, 1H), 6.33 (d, *J* = 8.4 Hz, 1H), 4.69 (s, 1H), 3.73 (q, *J* = 7.0 Hz, 4H), 3.59 (q, *J* = 7.2 Hz, 4H), 3.15-3.11 (m, 1H), 2.99-2.87 (m, 3H), 2.77 (d, *J* = 16.6 Hz, 2H), 2.18-2.12 (m, 2H), 2.01-1.94 (m, 2H), 1.87 (d, *J* = 13.6 Hz, 1H), 1.64 (d, *J* = 12.7 Hz, 1H), 1.50 (t, *J* = 13.6 Hz, 12H), 1.37 (t, *J* = 7.0 Hz, 6H), 1.28 (t, *J* = 7.1 Hz, 6H), 1.01 (t, *J* = 7.0 Hz, 3H); ^{13}C NMR (101 MHz, $CDCl_3$): δ 158.3, 157.6, 156.2, 155.7, 148.2, 133.6, 131.4, 130.0, 128.7, 126.6, 123.6, 123.5, 121.2, 119.2, 115.3, 114.9, 113.7, 112.7, 110.9, 109.5, 97.6, 97.0, 56.0, 51.8, 46.6, 46.3, 45.3, 38.9, 30.0, 27.4, 19.9, 16.4, 12.8, 9.0. ESI-MS (m/z): $[M]^+$ calcd. for $C_{40}H_{47}N_4O$, 599.3750; found 599.3751

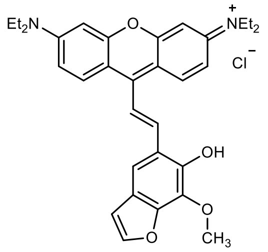

(E)-N-(6-(diethylamino)-9-(2-(6-hydroxy-7-methoxybenzofuran-5-yl)vinyl)-3H-xanthen-3-ylidene)-N-ethylethanaminium chloride salt (RD68) Compound **RD68** was prepared from **S68** (100 mg, 1 equiv) and dilithium reagent **1** (1.3 equiv) according to general procedure B. Violet solid (118 mg) was obtained in a 46% yield by flash chromatography (CH_2Cl_2/MeOH = 10:1, v/v).. ^1H NMR (400 MHz, $CDCl_3$): δ 7.97 (d, *J* = 9.5 Hz, 2H), 7.78 (t, *J* = 16.2 Hz, 1H), 7.52-7.45 (m, 3H), 6.90 (d, *J* = 9.5 Hz, 2H), 6.66 (d, *J* = 11.4 Hz, 1H), 6.65 (s, 2H), 4.13 (s, 3H), 3.54 (q, *J* = 7.0 Hz, 8H), 3.36 (q, *J* = 7.1 Hz, 4H), 1.30 (t, J = 7.0 Hz, 12H), 1.25 (t, J = 7.0 Hz, 6H); ^{13}C NMR (101 MHz, $CDCl_3$) δ 157.5, 155.0, 154.8, 147.6, 146.0, 144.8, 142.6, 133.3, 131.1, 122.4, 120.6, 118.4, 114.4, 113.7, 112.0, 107.1, 96.3, 61.1, 45.9, 12.8. ESI-HRMS (m/z): $[M]^+$ calcd. for $C_{32}H_{35}N_2O_4^+$, 511.2597; found 511.2594

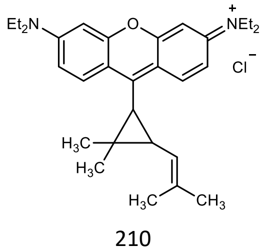

N-(6-(diethylamino)-9-(2,2-dimethyl-3-(2-methylprop-1-en-1-yl)cyclopropyl)-3H-xanthen-3-ylidene)-N-ethylethanaminium chloride salt (RD69) Compound **RD69** was prepared from **S69** (100 mg, 1 equiv) and dilithium reagent **1** (1.3 equiv) according to general procedure B. Violet solid (188 mg) was obtained as a violet solid in a 77% yield by flash chromatography (CH_2Cl_2/MeOH = 12:1, v/v). ^1H NMR (400 MHz, $CDCl_3$): δ 7.93 (s, 2H), 6.97 (s, 2H), 6.69 (s, 2H), 5.13 (d, *J* = 8.4 Hz, 1H), 3.63 (q, *J* = 8.0 Hz, 8H), 1.96 (d, *J* = 6.2 Hz, 1H), 1.85-1.77(m, 7H), 1.39(s, 3H), 1.31 (t, J = 7.8 Hz, 12H), 0.80(s, 3H); 13C NMR (101 MHz, $CDCl_3$) δ 158.8, 155.4, 136.8, 131.0, 121.2, 115.3, 113.8, 96.2, 46.1, 33.2, 32.2, 29.7, 26.4, 26.0, 24.2, 21.5, 18.6, 12.8. ESI-HRMS (m/z): $[M]^+$ calcd. for $C_{26}H_{29}N_4O_3^+$, 445.3219; found 445.3220.

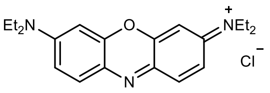

N-(7-(diethylamino)-3H-phenoxazin-3-ylidene)-N-ethylethanaminium chloride salt (RD70[26]) Compound **RD70** was prepared from **S70** (100 mg, 1 equiv) and dilithium reagent **1** (1.3 equiv) according to general procedure B. Blue-green solid (206 mg) was obtained in a 67% yield by flash chromatography (CH_2Cl_2/MeOH = 10:1, v/v). ^1H NMR (400 MHz, CD_3OD): δ7.74 (s, 2H), 7.37 (s, 2H), 6.94 (s, 2H), 3.78 (s, 8H), 1.36 (s, 12H); ESI-MS (m/z): $[M]^+$ calcd. for $C_{20}H_{26}N_3O$, 324.2076; found 324.2079.

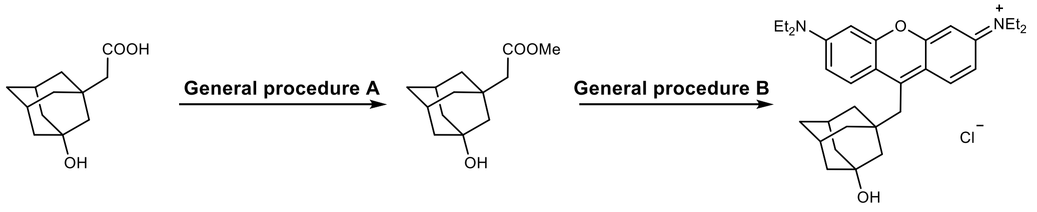

Supplementary Figure 387. Synthesis route to compound **RD71**. **S71** is prepared via the general procedure A: MeOH, cat. H_2SO_4, reflux. **RD71** is prepared via the general procedure B: compound **1**, THF, -78 °C – r. t..

methyl 2-((1r,3s,5R,7S)-3-hydroxyadamantan-1-yl)acetate (S71)
Prepared according to general procedure A using 2-((1r,3s,5R,7S)-3-hydroxyadamantan -1-yl)acetic acid to give a white solid in a yield of 98%. ^1H NMR (400 MHz, $CDCl_3$): δ 3.84 (s, 3H), 2.20 (s, 2H), 2.15 (s, 2H), 1.69-1.64 (m, 4H), 1.56-1.51 (m, 8H).

N-(6-(diethylamino)-9-(((1s,3s,5R,7S)-3-hydroxyadamantan-1-yl)methyl)-3H-xanthen-3-ylidene)-N-ethylethanaminium chloride (RD71)
Compound **RD71** was prepared from **S71** (100 mg, 1 equiv) and dilithium reagent **1** (1.3 equiv) according to general procedure B. Violet solid (161 mg) was obtained as a violet solid in a 74% yield by flash chromatography (CH_2Cl_2/MeOH = 10:1, v/v). ^1H NMR (400 MHz, $CDCl_3$): δ 7.99 (d, *J* = 9.6 Hz, 2H), 7.03 (dd, *J* = 9.5, 2.0 Hz, 2H), 6.66 (d, *J* = 2.3 Hz, 2H), 3.60 (q, *J* = 6.8 Hz, 8H), 3.25 (s, 2H), 2.13 (s, 2H), 1.63-1.56 (m, 6H), 1.46 (s, 6H), 1.32 (t, *J* = 7.1 Hz, 12H). ^{13}C NMR (101 MHz, $CDCl_3$): δ 157.8, 157.3, 155.4, 131.4, 114.9, 113.8, 95.9, 68.3, 50.7, 45.9, 43.9, 42.7, 40.3, 40.1, 35.2, 30.9, 29.7, 23.2, 12.7. EI-HRMS (m/z): $[M]^+$ calcd. for $C_{32}H_{43}N_2O_2$, 487.3319; found 487.3327.

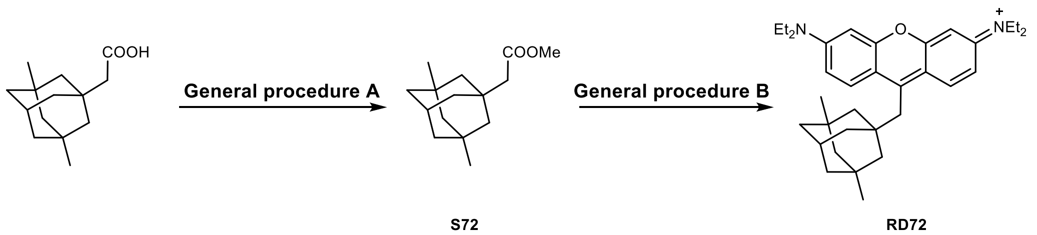

Supplementary Figure 388. Synthesis route to compound **RD72**. **S72** is prepared via the general procedure A: MeOH, cat. H_2SO_4, reflux. **RD72** is prepared via the general procedure B: compound **1**, THF, -78 °C – r. t..

methyl 2-((1r,3R,5S,7r)-3,5-dimethyladamantan-1-yl)acetate (S72)

Prepared according to general procedure A using 2-((1r,3R,5S,7r)-3,5-dimethyladamantan -1-yl)acetic acid to give a white solid in a yield of 98%. ^{1}H NMR (400 MHz, $CDCl_3$): δ 3.65 (s, 3H), 2.11 (s, 2H), 2.07-2.04 (m, 1H), 1.43 (d, *J* = 2.5 Hz, 2H), 1.30 (d, *J* = 2.5 Hz, 4H), 1.28-1.18 (m, 4H), 1.16-1.06 (m, 2H), 0.81 (s, 6H).

N-(6-(diethylamino)-9-(((1r,3R,5S,7r)-3,5-dimethyladamantan-1-yl)methyl)-3H-xanthen-3-ylidene)-N-ethylethanaminium chloride (RD72)

Compound **RD72** was prepared from **S72** (100 mg, 1 equiv) and dilithium reagent **1** (1.3 equiv) according to general procedure B. Violet solid (148 mg) was obtained as a violet solid in a 70% yield by flash chromatography (CH_2Cl_2/MeOH = 10:1, v/v). ^{1}H NMR (400 MHz, $CDCl_3$): δ 7.95 (d, *J* = 9.7 Hz, 2H), 7.04 (dd, *J* = 9.6, 2.4 Hz, 2H), 6.71 (d, *J* = 2.4 Hz, 2H), 3.64 (dd, *J* = 14.2, 7.1 Hz, 8H), 3.18 (s, 2H), 2.02-1.98 (m, 1H), 1.33-1.31 (m, 14H), 1.22-1.19 (m, 8H), 1.05 (q, *J* = 10.7 Hz, 2H), 0.73 (s, 6H). ^{13}C NMR (101 MHz, $CDCl_3$): δ157.9, 157.4, 155.4, 131.2, 114.8, 113.6, 96.1, 50.7, 50.2, 46.1, 42.6, 42.3, 40.6, 39.1, 31.9, 30.5, 29.9, 12.8. EI-HRMS (m/z): $[M]^+$ calcd. for $C_{34}H_{47}N_2O$, 499.3683; found 499.3687.

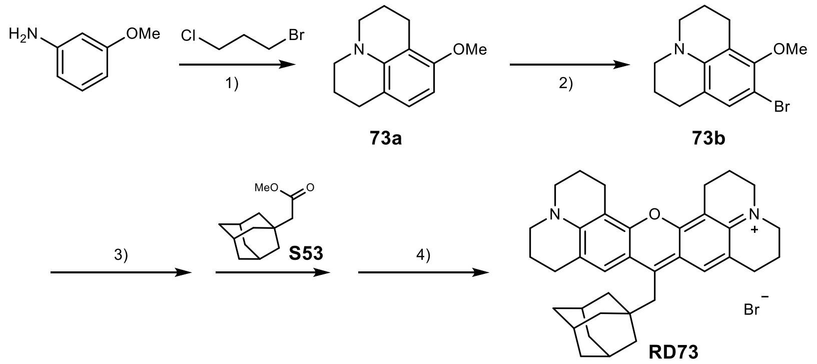

Supplementary Figure 389. Synthesis route to compound **RD73**. 1) Na_2CO_3, 150 °C; 2) Br_2, DCM, 0 °C – r. t.; 3) nBuLi, THF, -78 °C; 4) BBr_3, THF, -78 °C.

8-methoxy-2,3,6,7-tetrahydro-1H,5H-pyrido[3,2,1-ij]quinoline （73a）

3-Methoxyaniline (50.0 g, 1 equiv, 0.10 mol), excess 1-bromo-3-chloropropane (50 mL), and Na_2CO_3 (86.1 g, 2 equiv, 0.20 mol) were combined in a 500 mL round bottomed flask equipped with a condenser. The resulting mixture was heated to 70 °C for 1 h and 100 °C for 2 h and then reflux for 20 h before being cooled to room temperature. CH_2Cl_2 (20 mL) was added to dilute the viscous mixture before all solid material were filtered off. CH_2Cl_2 was removed under reduced pressure. The resulting liquid was distillated under vacuum. Compound **73a** (62 g, colorless liquid)

was obtained after 3-chlorobromopropane in a 75% yield. ^{1}H NMR (400 MHz, $CDCl_3$): δ 6.75 (d, *J* = 8.4 Hz, 1H), 6.16 (d, *J* = 8.0 Hz, 1H), 3.76 (s, 3H), 3.06-3.11 (m, 4H), 2.71 (t, *J* = 6.8 Hz, 2H), 2.66 (t, *J* = 6.8 Hz, 2H), 1.93-1.99 (m, 4H).

9-bromo-8-methoxy-2,3,6,7-tetrahydro-1H,5H-pyrido [3,2,1-ij]quinolone (73b)

A solution of dry bromine (3.93 g, 1 equiv., 24.60 mmol) in CH_2Cl_2 (20 ml) was added with vigorous stirring to a solution of **73a** (5 g, 1 equiv., 24.60 mmol) in the same solvent (50 ml) at 0 °C. A saturated solution of $NaHCO_3$ (50 mL) was added after an hour and the resulting mixture was extracted repeatedly with CH_2Cl_2. The organic layer was combined, dried with $MgSO_4$ and filtered. CH_2Cl_2 was removed under reduced pressure to yield a light purple oil (6.59 g) in a 95 % yield. ^{1}H NMR (400 MHz, $CDCl_3$): δ 6.94 (s, 1H), 3.75 (s, 3H), 3.09-3.12 (m, 4H), 2.78 (t, *J* = 6.8 Hz, 2H), 2.67-2.70 (t, *J* = 6.8 Hz, 2H), 1.92-1.96 (m, 4H).

Synthesis of RD73

A solution of n-BuLi (2.5 M in hexane) (2.5 equiv) was slowly syringed into a solution of **73b** (1.12 g, 2.5 equiv., 3.97 mmol) in THF at -78 °C, and the resulting mixture was stirred for another 30 min before a solution of **S53** (165 mg, 1 equiv., 0.79 mmol) in THF was injected at -78 °C. The reaction mixture was allowed to warm to room temperature and stirred for another 12 h. Saturated NH_4Cl solution was poured into the reaction flask, and the reaction mixture was extracted with CH_2Cl_2 repeatedly. The organic layer was combined, dried with $MgSO_4$ and filtered. Then, all CH_2Cl_2 was removed under reduced pressure to give a viscous residue. A solution of BBr_3 (16 equiv.) was added to a solution of above viscous residue in THF (20 mL) at -78 °C via syringe and stirred for 2 h. 1 M NaOH solution was poured into the reaction flask to adjust pH value to 6, and the reaction mixture was extracted with CH_2Cl_2 repeatedly. The organic layer was combined and all solvent was removed under reduced pressure to give a violet soild, which was purified by a flash column using a mixture of CH_2Cl_2 and MeOH [12:1, v/v] as an eluent to afford **RD73** (145 mg) as a vinicolor metallic luster soild in a 38% yield. ^{1}H NMR (400 MHz, $CDCl_3$): δ 7.47 (s, 2H), 3.58 (t. *J* = 5.6 Hz, 8H), 3.07 (s, 2H), 3.01 (t, *J* = 6.0 Hz, 4H), 2.92 (t, *J* = 6.0 Hz, 4H), 2.09-2.13 (m, 8H), 1.95 (s, 3H), 1.54-1.67 (m, 20H). ^{13}C NMR (400 MHz, $CDCl_3$): δ 154.2, 151.5, 150.8, 125.7, 122.6, 114.1, 104.9, 50.8, 50.3, 43.8, 40.5, 36.9, 36.3, 28.9, 27.9, 20.8, 19.9, 19.7. ESI-HRMS (m/z): $[M]^+$calcd. for $C_{36}H_{43}N_2O^+$, 519.3370; found 519.3375.

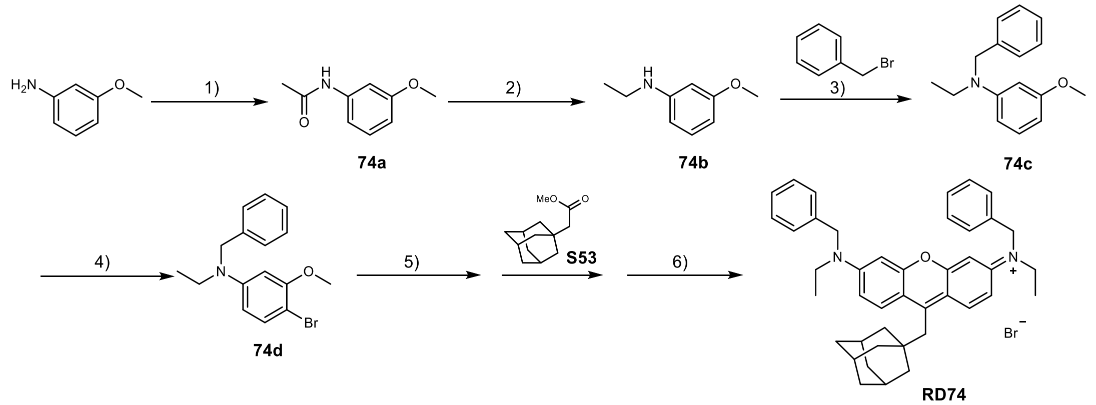

Supplementary Figure 390. Synthesis route to compound **RD74**. 1) Ethyl acetate, $AlCl_3$, Et_3N; 2) $NaBH_4$, THF, I_2, 50 °C; 3) K_2CO_3, KI, DMF, 60 °C; 4) Br_2, DCM, 0 °C – r. t.; 5) nBuLi, THF, -78 °C; 6) BBr_3, THF, -78 °C.

N-(3-methoxyphenyl)acetamide (74a)

3-Methoxyaniline (10.00 g, 1 equiv., 81.20 mmol), Et_3N (22.19 g, 2.7 equiv., 219.24 mmol), and ethyl acetate (80 mL) were combined in a 250 mL round bottomed flask. $AlCl_3$ (9.74 g, 1.0 equiv, 81.20 mmol) was added in batches, and the reaction mixture was stirred intensely for 2 h before extracted with H_2O repeatedly. The organic layer was combined, dried with $MgSO_4$ and filtered. Then, all EA was removed under reduced pressure to give a viscous yellow oil without furthere purified for next step.

N-ethyl-3-methoxyaniline (**74b**)

NaBH$_4$ was added to a solution of the above viscous oil (**74a**) in anhydrous THF (100 mL) at 0 ^oC, and the mixture was intensely stirred for 30 min. A solution of I_2 in THF was syringed into the mixtures and stired for another 6 h at 50 ^oC. H_2O was added into the reaction flask slowly, and the reaction mixture was extracted with CH_2Cl_2 repeatedly. The organic layer was combined, dried with $MgSO_4$ and filtered. Then, all CH_2Cl_2 was removed under reduced pressure to give a viscous residue, which was purified by a flash column using a mixture of petroleum ether and EtOAc [15:1, v/v] as an eluent to afford **74b** (7.77 g) as a colorless oil in an 85% yield (for two steps). ^1H NMR (400 MHz, DMSO-d_6): δ 6.99 (t, J = 8.0 Hz, 1H), 6.22 (dd, J_1 = 8.4 Hz, J_2 = 1.6 Hz, 1H), 6.17-6.19 (m, 2H), 3.67 (s, 3H), 3.03 (q, J = 7.2 Hz, 2H), 1.14 (t, J = 7.2 Hz, 3H).

N-benzyl-N-ethyl-3-methoxyaniline (**74c**)

To a stirred solution of **74b** (5 g, 1.0 equiv., 33.07 mmol) in DMF (10 mL) were added benzyl bromide (6.22 g, 1.1 equiv., 36.37 mmol) and K_2CO_3 (5.03 g, 1.1 equiv., 36.37 mmol) and KI (1.10 g, 0.2 equiv., 36.37 mmol). The mixture was heated to 80°C and stirred until the reaction was complete, as monitored by TLC. Solvent was removed under vacuum and the product was extracted with CH_2Cl_2 repeatedly. The combined CH_2Cl_2 layers were washed with H_2O, dried with $MgSO_4$ and filtered. After removal of the solvent, the product was purified by a flash column using a mixture of petroleum ether and EtOAc [100:1, v/v] as an eluent to afford N-benzyl-N-ethyl-3-methoxyaniline (**74c**) as a colorless oil (6.82 g) in an 85% yeild. ^1H NMR (400 MHz, CDCl3): δ 7.136 (t, J = 8.4 Hz, 1H), 6.36 (d, J = 6.0 Hz, 1H), 6.27 (d, J = 5.6 Hz, 1H), 3.79 (s, 3H), 3.38 (q, J = 7.2 Hz, 2H), 2.90 (s, 3H), 1.17 (t, J = 7.2 Hz, 3H).

N-benzyl-4-bromo-N-ethyl-3-methoxyaniline (**74d**)

A solution of dry bromine (3.31 g, 1 equiv., 20.72 mmol) in CH_2Cl_2 (20 ml) was added with vigorous stirring to a solution of **74c** (5 g, 1 equiv., 20.72 mmol) in the same solvent (50 ml) at 0 ^oC. A saturated solution of $NaHCO_3$ (50 mL) was added after an hour and the resulting mixture was extracted repeatedly with CH_2Cl_2. The organic layer was combined, dried with $MgSO_4$ and filtered. CH_2Cl_2 was removed under reduced pressure to yield a light purple oil (6.18 g) in a 93 % yield. ^1H NMR (400 MHz, CDCl3): δ 7.36-7.38 (m, 1H), 7.29-7.33 (m, 2H), 7.22-7.24 (m, 3H), 6.19-6.21 (m, 2H), 4.50 (s, 2H), 3.74 (s, 3H), 3.48 (q, J = 7.2 Hz, 2H), 1.22 (t, J = 7.2 Hz, 3H).

Synthesis of RD74

A solution of n-BuLi (2.5 M in hexane) (2.5 equiv) was syringed into a solution of **74c** (576.50 mg, 2.5 equiv., 1.80 mmol) in THF at -78 ^oC, and the resulting mixture was stirred for another 45 min before a solution of **S53** (150 mg, 1 equiv., 0.72 mmol) in THF inject at -78 ^oC. The reaction mixture was allowed to warm to room temperature for another 12 h. Saturated NH_4Cl solution was poured into the reaction flask, and the reaction mixture was extracted with CH_2Cl_2 repeatedly. The organic layer was combined, dried with $MgSO_4$ and filtered. Then, all CH_2Cl_2 was removed under reduced pressure to give a viscous residue, without further purification. A solution of BBr_3 (16 equiv.) was added to a solution of above viscous residue in THF (20 mL) at -78 ^oC via syringe, stirring

for 2 h, 1 M NaOH solution was poured into the reaction flask to adjust pH value to 6, and the reaction mixture was extracted with CH_2Cl_2 repeatedly. The organic layer was combined and all solvent was removed under reduced pressure to give a violet soild, which was purified by a flash column using a mixture of CH_2Cl_2 and MeOH [12:1, v/v] as an eluent to afford **RD74** (211 mg) as a vinicolor metallic luster soild in an 43% yield. ^1H NMR (400 MHz, $CDCl_3$): δ 8.07 (d, *J* = 9.2 Hz, 2H), 7.29-7.38 (m, 6H), 7.18-7.23 (m, 6H), 6.76 (s, 2H), 4.84 (s, 4H), 3.78 (q, *J* = 7.2Hz, 4H), 3.24 (s, 2H), 1.94 (s, 3H), 1.54-1.65 (m, 12H), 1.38 (t, *J* = 7.2 Hz, 6H). ^{13}C NMR (400 MHz, $CDCl_3$): δ 159.2, 157.2, 156.3, 135.2, 131.4, 129.1, 127.9, 126.2, 115.4, 114.1, 96.7, 54.3, 46.9, 43.7, 41.3, 37.6, 29.6, 28.8, 12.4. ESI-HRMS (m/z): $[M]^+$calcd. for $C_{42}H_{47}N_2O^+$, 595.3683; found 595.3688.

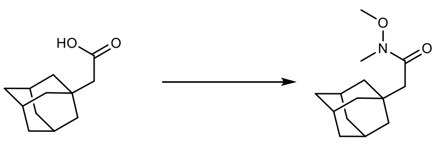

Supplementary Figure 391. Synthesis route to compound 2-((3r,5r,7r)-adamantan-1-yl) -N-methoxy-N-methylacetamide. Reaction condition: CDI, *N,O*-dimethylhydroxylamine, dry DCM, r. t..

CDI (3.34 g, 2 equiv, 0.58 mol) was added portionwise to2-((3r,5r,7r)-adamantan-1-yl)acetic acid (2 g, 1 equiv, 0.29 mol) in CH_2Cl_2 (40 mL). The mixture was stirred at room temperature for 1 h. Then *N*,*O*-dimethylhydroxylamine hydrochloride (4.02 g, 4 equiv, 1.16 mol) was added. The mixture was stirred at room temperature overnight, poured out into H_2O, and extracted with CH_2Cl_2. The organic layer was separated, washed several times with H_2O, dried with $MgSO_4$ and filtered. The solvent was evaporated to afford as a white solid (2.32g, 95%). The spectral data are in good agreement with the literature[27]. ^1H NMR (400 MHz, $CDCl_3$): δ 3.66 (s, 3H), 3.18 (s, 3H), 2.19 (s, 2H), 1.96 (s, 2H), 1.63-1.71 (m, 12H).

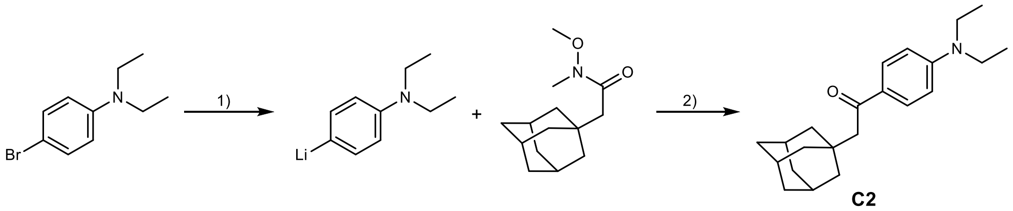

Supplementary Figure 392. Synthesis route to compound **C2**. 1) nBuLi, THF, -78 °C, 15 min; 2) THF, -78 °C – r. t..

2-((3r,5r,7r)-adamantan-1-yl)-1-(4-(diethylamino)phenyl)ethan-1-one (**C2**)

n-BuLi (2.5 M) in hexane (1.54 mL, 1.1 equiv., 3.86 mmol) was added dropwise at -78 °C to a solution of 4-bromo-N,N-diethylaniline (800 mg, 1 equiv., 3.51 mmol) in THF (30 mL) and stirred for 30 min. A solution of 2-((3r,5r,7r)-adamantan-1-yl)-N-methoxy-N-methylacetamide (832 mg, 1 equiv., 3.51 mmol) in THF (10 mL) was added at -78 °C. The mixture was stirred for 4 h while the temperature was brought to room temperature, hydrolyzed with H_2O, and extracted with EtOAc. The organic layer was separated, dried with $MgSO_4$ and filtered. The solvent was evaporated and the residue was purified by column chromatography over silica gel (eluent: PE/EtOAc: 100/1, v/v). The residue was precipitated from diethyl ether to afford 2-((3r,5r,7r)-adamantan-1-yl)-1-(4-(diethylamino)phenyl)ethan-1-one (**C2**) (457 mg, 40%). ^1H NMR (400 MHz, $CDCl_3$): δ 7.86 (d, *J* = 9.2 Hz, 2H), 6.61 (d, *J* = 9.2 Hz, 2H), 3.41 (q, *J* = 7.2 Hz, 4H), 2.60 (s, 2H), 1.94 (s, 3H), 1.60-1.69 (m, 12H).

^{13}C NMR (400 MHz, $CDCl_3$): δ 197.9, 150.9, 131.1, 126.3, 109.9, 50.7, 44.5, 43.2, 36.8, 33.9, 28.8, 12.5. ESI-HRMS (m/z): $[M]^+$calcd. for $C_{22}H_{32}NO^+$, 326.2478; found 326.2480.

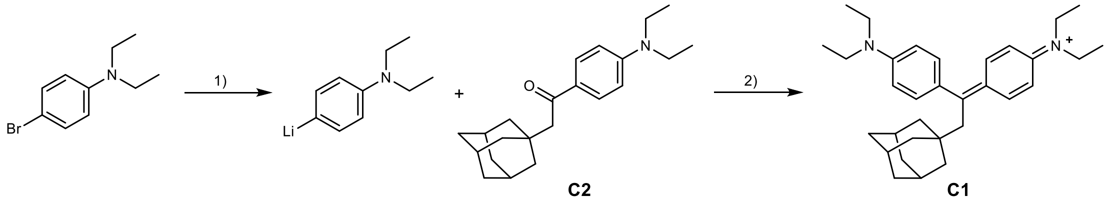

C2

C1

Supplementary Figure 393. Synthesis route to compound **C1**. 1) nBuLi, THF, -78 °C, 15 min; 2) THF, -78 °C – r. t..

4,4'-(2-((3r,5r,7r)-adamantan-1-yl)ethene-1,1-diyl)bis(N,N-diethylaniline) (**C1**)

n-BuLi (2.5 M) in hexane (1.54 mL, 1.1 equiv., 3.86 mmol) was added dropwise at -78 °C to a solution of 4-bromo-N,N-diethylaniline (800 mg, 1 equiv., 3.51 mmol) in THF (30 mL) and stirred for 30 min. A solution of **C2** (1.0 g, 1 equiv., 3.51 mmol) in THF (10 mL) was added at -78 °C. The mixture was stirred for 4 h while the temperature was brought to room temperature, hydrolyzed with dilute HCl solution (1M), and extracted with EtOAc. The organic layer was separated, and solvent was evaporated off. The residue was purified by column chromatography over silica gel (eluent: PE/EtOAc: 100/5, v/v). The residue was precipitated from diethyl ether to afford **C1** (385 mg, 24%). ^1H NMR (400 MHz, $CDCl_3$): δ 7.14 (d, *J* = 8.8 Hz, 2H), 7.05 (d, *J* = 8.4 Hz, 2H), 7.70 (d, *J* = 8.4 Hz, 2H), 6.61 (d, *J* = 8.8 Hz, 2H), 5.75 (s, 1H), 3.42 (q, *J* = 7.2 Hz, 4H), 3.36 (q, *J* = 7.2 Hz, 4H), 1.91 (s, 3H), 1.61-1.66 (m, 12H), 1.24 (t, *J* = 7.2 Hz, 6H), 1.17 (t, *J* = 7.2 Hz, 6H). ^{13}C NMR (400 MHz, $CDCl_3$): δ 149.3, 149.1, 138.5, 137.4, 131.2, 131.0, 127.7, 127.6, 112.2, 111.7, 44.4, 43.4, 40.7, 36.8, 36.0, 28.7.ESI-HRMS (m/z): $[M]^+$calcd. for $C_{32}H_{45}N_2^+$, 457.3577; found 457.3571.

Supplementary Notes

Diversity Index. Molecular fingerprints capture structural or topological features and properties of molecules in a binary bit string format. Extended-connectivity fingerprints (ECFPs) are well-established fingerprints which encodes each atom and its molecular environment within a circle with a diameter of several chemical bonds. Depending on the chosen bond radius, different numbers of structural features having different sizes are produced. For several reasons, ECFPs have become some of the most widely used similarity search tools and diversity evaluation indexes in drug discovery settings including their availability in the popular Pipeline Pilot software and their ease of use. In this study, we introduce the definition of total number of fingerprint features to quantitatively measure the diversity and novelty of our library. We first calculated the specified fingerprint for each molecule using ECFP_6 and counted the total number of unique fingerprint features collected over all the molecules. Then the diversity number fingerprint features was defined as the total number of fingerprint features divided by the number of molecules.

Supplementary References

1. Bajwa, N., & Jennings, M. P. Efficient and selective reduction protocols of the 2,2-dimethyl-1, 3-benzodioxan-4-one functional group to readily provide both substituted salicylaldehydes and 2-hydroxybenzyl alcohols. *J. Org. Chem.* **71**, 3646-3649 (2006).
2. El-Barbary, A. A., Clausen, K., & Lawesson, S. O. Studies on organophosphorus compounds 1: Thiation of 3, 1-benzoxathian-4-ones. New routes to 1, 2-benzisothiazole-3(2*H*)-thiones and 3*H*-1,2-benzodithiol-3-imines. *Tetrahedron*. **36,** 3309-3315 (1980).
3. Diener, M. E., Metrano, A. J., Kusano, S., & Miller, S. J. Enantioselective synthesis of 3-arylquinazolin-4(3*H*)-ones via peptide-catalyzed atroposelective bromination. *J. Am. Chem. Soc.* **137**, 12369-12377 (2015).
4. Huang, K. *et al.* Cascade reaction and FRET-based fluorescent probe for the colorimetric and ratiometric signaling of hydrogen sulfide. *Tetrahedron Lett.* **56**, 3769-3773 (2015).
5. Vinot, N., & Maitte, P. Synthèse de dimethyl-2, 2 chromannediones-3, 4: Condensation avec les *ortho*-diaminopyridines. *J. Heterocycl. Chem*. **26**, 1013-1021 (1989).
6. Chen, Y., Turlik, A., & Newhouse, T. R. Amide α, β-dehydrogenation using allyl-palladium catalysis and a hindered monodentate anilide. *J. Am. Chem. Soc.* **138**, 1166-1169 (2016).
7. Maier. J. M. *et al*. Measurement of silver-π interactions in solution using molecular torsion balances. *J. Am. Chem. Soc.* **137**, 8014-8017 (2015).
8. Zhu, Y. *et al.* Copper-catalyzed methyl esterification reactions via C-C bond cleavage. *J. Org. Chem.* **78**, 9898-9905 (2013).
9. Kawai, K. *et al.* A reductant-resistant and metal-free fluorescent probe for nitroxyl applicable to living cells. *J. Am. Chem. Soc.* **135**, 12690-12696 (2013).
10. Yang, L., Bai, Y., Tan, X., Wang, Z., & Zhang, X. Controllable supramolecular polymerization through host-guest interaction and photochemistry. *ACS Macro Lett.* **4**, 611-615 (2015).
11. Iannazzo, L. *et al.* Alkynylboronates and-boramides in CoI-and RhI-Catalyzed [2+2+2] cycloadditions: Construction of oligoaryls through selective suzuki couplings. *Eur. J. Org. Chem.* **18**, 3283-3292 (2011).
12. Kisic, A., Stephan, M., & Mohar, B. Asymmetric transfer hydrogenation of 1-naphthyl ketones

by an *ansa*-Ru (II) complex of a DPEN-SO_2N (Me)-$(CH_2)_2$(η^6-*p*-Tol) combined ligand. *Org. Lett.* **15**, 1614-1617 (2013).

13. Menger, F. M., & Sorrells, J. L. A non-steroidal facial amphiphile. *J. Am. Chem. Soc.* **128**, 4960-4961 (2006).
14. Moreno, R. M. *et al.* Enantiocontrolled preparation of the first stable α-ferrocenylalanine derivatives. *Eur. J. Org. Chem.* **14**, 2388-2396 (2018).
15. Yang, M. H., Orsi, D. L., & Altman, R. A. Ligand-controlled regiodivergent palladium-catalyzed decarboxylative allylation reaction to access α, α-difluoroketones. *Angew. Chem. Int. Ed.* **54**, 2361-2365 (2015).
16. Sigeev, A. S., Peregudov, A. S., Cheprakov, A. V., & Beletskaya, I. P. The palladium slow-release pre-catalysts and nanoparticles in the "phosphine-free" Mizoroki-Heck and Suzuki-Miyaura reactions. *Adv. Synth. Catal*. **357**, 417-429 (2015).
17. Fokin, A. A. *et al.* Preparative synthesis of vinyl diamondoids. *Synth. Commun.* ***43***, 1772-1777 (2013).
18. Ke, H., Chen, X., & Zou, G. N-heterocyclic carbene-assisted, bis(phosphine) nickel-catalyzed cross-couplings of diarylborinic acids with aryl chlorides, tosylates, and sulfamates. *J. Org. Chem.* **79**, 7132-7140 (2014).
19. Rusu, A. *et al.* Triprotic site-specific acid-base equilibria and related properties of fluoroquinolone antibacterials. *J. Pharm. Biomed. Anal.* **66**, 50-57 (2012).
20. Lei, Z. et al. Synthesis of Sterically Protected Xanthene Dyes with Bulky Groups at C-3′ and C-7′. *J. Org. Chem.* **80**, 11538-11543 (2015).
21. Best, Q. A., Xu, R., McCarroll, M. E., Wang, L., & Dyer, D. J. Design and investigation of a series of rhodamine-based fluorescent probes for optical measurements of pH. J. *Org. Lett*. **12**, 3219-3221 (2010).
22. Lin, W., Long, L., Chen, B., Tan, W., & Gao, W. Fluorescence turn-on detection of Cu^{2+} in water samples and living cells based on the unprecedented copper-mediated dihydrorosamine oxidation reaction. *Chem. Commun*. **46**, 1311-1313 (2010).
23. Takano, Y. *et al.* Development of a reversible fluorescent probe for reactive sulfur species, sulfane sulfur, and its biological application. *Chem. Commun*. **53**, 1064-1067 (2017).
24. Wu, C. *et al.* Ring expansion of spiro-thiolactam in rhodamine scaffold: switching the recognition preference by adding one atom. *Org. Lett.* **14**, 4198-4201 (2012).
25. Shandura, M. P., Poronik, Y. M., & Kovtun, Y. P. New heterocyclic analogues of rhodamines. *Dyes and Pigments.* **73**, 25-30 (2007).
26. Ge, J. F., Arai, C., & Ihara, M. The convenient synthesis of zinc chloride-free 3, 7-bis (dialkylamino) phenoxazinium salts. *Dyes and Pigments.* **79**, 33-39 (2008).
27. Huang, H. *et al*. Preparation of 5*H*-imidazo[5,1-a]isoindole compound as an IDO inhibitor for treating cancer. 2017. CN Patent 107176956 A. Sep 19, 2017.
